# Spliced integrated retrotransposed element (SpIRE) formation in the human genome

**DOI:** 10.1371/journal.pbio.2003067

**Published:** 2018-03-05

**Authors:** Peter A. Larson, John B. Moldovan, Naveen Jasti, Jeffrey M. Kidd, Christine R. Beck, John V. Moran

**Affiliations:** 1 Department of Human Genetics, University of Michigan Medical School, Ann Arbor, Michigan, United States of America; 2 Department of Computational Medicine and Bioinformatics, University of Michigan Medical School, Ann Arbor, Michigan, United States of America; 3 Department of Internal Medicine, University of Michigan Medical School, Ann Arbor, Michigan, United States of America; Fred Hutchinson Cancer Research Center, United States of America

## Abstract

Human Long interspersed element-1 (L1) retrotransposons contain an internal RNA polymerase II promoter within their 5′ untranslated region (UTR) and encode two proteins, (ORF1p and ORF2p) required for their mobilization (i.e., retrotransposition). The evolutionary success of L1 relies on the continuous retrotransposition of full-length L1 mRNAs. Previous studies identified functional splice donor (SD), splice acceptor (SA), and polyadenylation sequences in L1 mRNA and provided evidence that a small number of spliced L1 mRNAs retrotransposed in the human genome. Here, we demonstrate that the retrotransposition of intra-5′UTR or 5′UTR/ORF1 spliced L1 mRNAs leads to the generation of spliced integrated retrotransposed elements (SpIREs). We identified a new intra-5′UTR SpIRE that is ten times more abundant than previously identified SpIREs. Functional analyses demonstrated that both intra-5′UTR and 5′UTR/ORF1 SpIREs lack *Cis*-acting transcription factor binding sites and exhibit reduced promoter activity. The 5′UTR/ORF1 SpIREs also produce nonfunctional ORF1p variants. Finally, we demonstrate that sequence changes within the L1 5′UTR over evolutionary time, which permitted L1 to evade the repressive effects of a host protein, can lead to the generation of new L1 splicing events, which, upon retrotransposition, generates a new SpIRE subfamily. We conclude that splicing inhibits L1 retrotransposition, SpIREs generally represent evolutionary “dead-ends” in the L1 retrotransposition process, mutations within the L1 5′UTR alter L1 splicing dynamics, and that retrotransposition of the resultant spliced transcripts can generate interindividual genomic variation.

## Author summary

Long interspersed element-1 (L1) sequences comprise about 17% of the human genome reference sequence. The average human genome contains about 100 active L1s that mobilize throughout the genome by a “copy and paste” process termed retrotransposition. Active L1s encode two proteins (ORF1p and ORF2p). ORF1p and ORF2p preferentially bind to their encoding RNA, forming a ribonucleoprotein particle (RNP). During retrotransposition, the L1 RNP translocates to the nucleus, where the ORF2p endonuclease makes a single-strand nick in target site DNA that exposes a 3′ hydroxyl group in genomic DNA. The 3′ hydroxyl group then is used as a primer by the ORF2p reverse transcriptase to copy the L1 RNA into cDNA, leading to the integration of an L1 copy at a new genomic location. The evolutionary success of L1 requires the faithful retrotransposition of full-length L1 mRNAs; thus, it was surprising to find that a small number of L1 retrotransposition events are derived from spliced L1 mRNAs. By using genetic, biochemical, and computational approaches, we demonstrate that spliced L1 mRNAs can undergo an initial round of retrotransposition, leading to the generation of spliced integrated retrotransposed elements (SpIREs). SpIREs represent about 2% of previously annotated full-length primate-specific L1s in the human genome reference sequence. However, because splicing leads to intra-L1 deletions that remove critical sequences required for L1 expression, SpIREs generally cannot undergo subsequent rounds of retrotransposition and can be considered “dead on arrival” insertions. Our data further highlight how genetic conflict between L1 and its host has influenced L1 expression, L1 retrotransposition, and L1 splicing dynamics over evolutionary time.

## Introduction

Long interspersed element-1 (L1) is a non-long terminal repeat (non-LTR) retrotransposon that comprises approximately 17% of human genomic DNA [1]. Over 99.9% of human L1s cannot retrotranspose due to 5′ truncations, internal DNA rearrangements, or point mutations that inactivate the L1-encoded proteins [1–4]. However, the average diploid genome harbors approximately 80–100 full-length retrotransposition-competent L1s (RC-L1s) [5], including a small number of expressed [6–8], highly active (i.e., “hot”) L1s [5,9–11] that can retrotranspose efficiently in cultured cells or cancers. RC-L1 retrotransposition affects both intra- and interindividual human genetic variation (reviewed in [12,13]) and, on occasion, can lead to disease-producing mutations [14–16].

**Fig 1:**
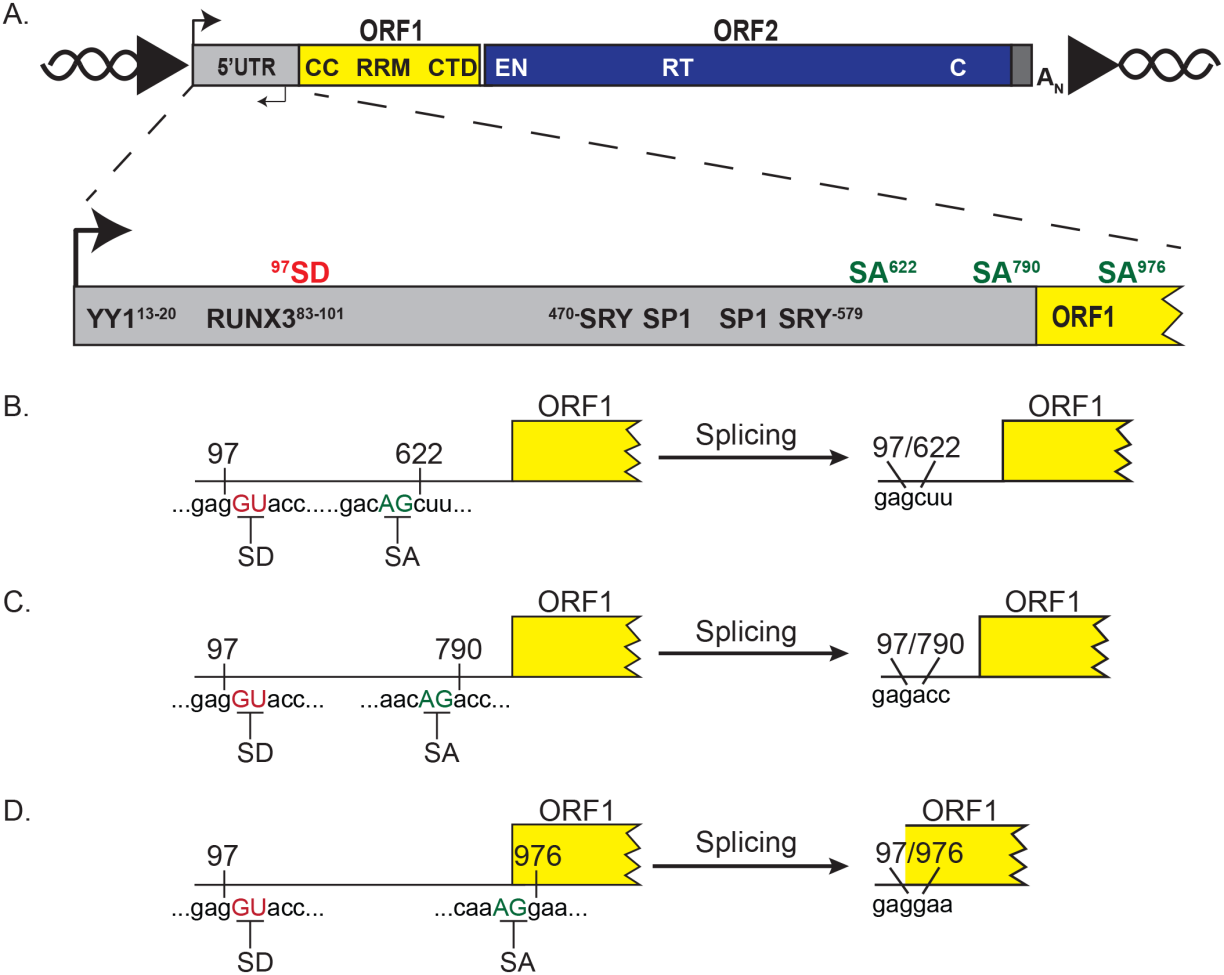
L1 mRNA contains potential SD and SA sites. (A) Schematic of a full-length retrotransposition competent genomic L1. Top: the 5′ and 3′ UTRs (gray rectangles), ORF1 (yellow rectangle), and ORF2 (blue rectangle) are indicated in the schematic. The approximate positions of sense transcription initiation and antisense transcription initiation are indicated with black arrows on the top and bottom of the 5′UTR, respectively. The approximate positions of the coiled-coil (CC), RNA recognition motif (RRM), and C-terminal domain (CTD) are indicated in black lettering in ORF1. The endonuclease (EN), reverse transcriptase (RT), and cysteine-rich (C) domain are indicated in white lettering in ORF2. The 3′UTR ends in an A_N_. The L1 is flanked by target-site duplications (black arrowheads) in genomic DNA (black helical lines). Bottom: a magnified schematic of the 5′UTR and 5′ end of ORF1. The black arrow indicates the relative position of sense transcription initiation. The SD (red) and SA (green) sequences used to generate SpIREs are indicated above the 5′UTR (gray rectangle) and ORF1 (yellow rectangle). The position of the SD and SA sequences relative to L1.3 are indicated with superscript numbers. The relative positions of *Cis*-acting transcription factor binding sequences are indicated in the 5′UTR. (B–D) Schematics of the splicing events generating SpIRE_97/622_, SpIRE_97/790_, and SpIRE_97/976_. The SD (red underlined GU nucleotides) and SA (green underlined AG nucleotides) demark the intron boundaries used to generate each class of SpIRE. The left half of the figure depicts the L1 mRNA sequence before splicing and the right half of the figure depicts L1 mRNA after splicing. A_N_, poly(A) tract; C, cysteine-rich; CC, coiled-coil; CTD, C-terminal domain; EN, endonuclease; L1, Long interspersed element-1; ORF, open reading frame; poly(A), polyadenosine; RRM, RNA recognition motif; RT, reverse transcriptase; RUNX3, runt related transcription factor 3; SA, splice acceptor; SD, splice donor; SpIRE, spliced integrated retrotransposed element; SP1, specificity protein 1; SRY, sex determining region Y; UTR, untranslated region; YY1, yin and yang 1.

Human RC-L1s are approximately six kilobases (kb) in length [17,18]. They contain a 5′ untranslated region (UTR) that harbors both sense [19] and antisense [20] RNA polymerase II promoters (Fig 1A) as well as an antisense open reading frame (ORF0) [21], which encodes a protein that may mildly enhance L1 retrotransposition efficiency. The 5′UTR is followed by two open reading frames (ORF1 and ORF2) that are separated by a 63–base pair (bp) inter-ORF spacer that contains two in-frame stop codons [18,22] (Fig 1A). L1s end with a 3′UTR, which contains a conserved polypurine motif, a “weak” RNA polymerase II polyadenylation signal, and a variable length polyadenosine (poly(A)) tract (Fig 1A) [17,23–25].

ORF1 encodes an approximately 40-kilodalton (kDa) protein (ORF1p) [26] that contains an amino-terminal coiled-coil (CC) domain required for ORF1p trimerization [27–29], a centrally located noncanonical RNA recognition motif (RRM) domain [29,30], and a carboxyl-terminal domain (CTD) harboring conserved basic amino acid residues [29–32] (Fig 1A). The RRM and CTD are critical for ORF1p nucleic acid binding [32–36]; the nucleic acid chaperone activity is postulated to play a role in L1 integration [30,36,37]. ORF2 encodes an approximately 150-kDa protein (ORF2p) [38–40] that contains conserved apurinic/apyrimidinic-like endonuclease (EN) [41,42] and reverse transcriptase (RT) domains [31,43,44] as well as a conserved cysteine-rich (C) domain [31,45] (Fig 1A). Biochemical activities contained within both ORF1p and ORF2p are required for canonical EN-dependent L1 retrotransposition in cultured human cells [31,41].

A round of human RC-L1 retrotransposition begins with the internal sense-strand promoter initiating transcription at or near the first nucleotide of the 5′UTR [13,19,46,47]. The resultant bicistronic L1 mRNA is exported to the cytoplasm, where it undergoes translation [22,46,48,49]. Following translation, ORF1p and ORF2p preferentially bind to their encoding mRNA in *cis* to form a ribonucleoprotein particle (RNP) [33,35,50–53]. The 3′ poly(A) tail of L1 mRNA is a critical *Cis*-acting determinant for recruitment of nascent ORF2p to L1 RNA [51,54]. Components of the L1 RNP gain access to the nucleus by a mechanism that does not require nuclear envelope breakdown [55, 127]. L1 integration likely occurs by target-site primed reverse transcription (TPRT) [41,53,56,57]. During TPRT, the L1 EN makes a single-strand endonucleolytic nick at a thymidine-rich sequence (e.g., 5′-TTTT/A-3′, 5′-TTTC/A-3′, etc.) present on the “bottom” strand of a target site in genomic DNA to liberate a 3′ hydroxyl (3′-OH) group [41,57,58]. Microhomology-based annealing between the L1 poly(A) tail and thymidine residues at the L1 EN cleavage site in genomic DNA enhances the ability of the L1 ORF2p RT to use the resultant 3′-OH group as a primer to initiate reverse transcription of L1 mRNA [53,59]. How TPRT is completed requires elucidation. However, as demonstrated for the related R2 non-LTR retrotransposon from *Bombyx mori* [60], it is possible that enzymatic activities associated with L1 ORF2p participate in both second-strand (i.e., “top” strand) genomic DNA cleavage and second-strand L1 cDNA synthesis.

Although retrotransposition assays and biochemical studies revealed the L1-encoded proteins preferentially retrotranspose their encoding mRNA in *cis* [50,53,61,62], L1 ORF1p and/or ORF2p can act in *trans* (*Trans*-complementation) to retrotranspose RNAs encoded by nonautonomous short interspersed elements (SINEs; e.g., Alu RNA [63,64] and SINE-R/VNTR/Alu [SVA] RNA [65–67]). Additionally, the L1-encoded protein(s) can act in *trans* to retrotranspose noncoding RNAs [12,68–73] and cellular mRNAs, with the latter process leading to the formation of processed pseudogenes [50,62,73–77].

The evolutionary success of L1 requires the faithful retrotransposition of full-length L1 mRNAs. Previous studies have revealed the presence of functional splice donor (SD), splice acceptor (SA), and premature polyadenylation signals in primary full-length RC-L1 transcripts [24,78–81]. Paradoxically, the use of these sites during posttranscriptional RNA processing leads to the production of truncated and/or internally deleted L1 mRNAs [24,78–81], which could adversely affect L1 retrotransposition. Thus, it is somewhat surprising that *Cis*-acting sequences that could negatively affect L1 retrotransposition have not been removed by negative selection during L1 evolution.

Here, we address how the retrotransposition of spliced L1 mRNAs leads to the generation of spliced integrated retrotransposed elements (SpIREs). We describe two classes of SpIREs: those that splice within the 5′UTR (intra-5′UTR SpIREs) and those that splice from within the 5′UTR into the ORF1 sequence (5′UTR/ORF1 SpIREs). Additionally, we suggest a mechanism for why some apparently deleterious *Cis*-acting splice sites within L1 mRNA are conserved throughout L1 evolution. Finally, we provide experimental evidence revealing that L1 splicing dynamics are altered by structural changes within the 5′UTR that allow L1s to evade host repression and that retrotransposition of the resultant spliced variants can lead to the generation of new classes of SpIREs. Thus, these data provide a snapshot of how an “arms race” between L1 and host repressive factors may affect the evolutionary trajectory of L1 5′UTRs. In sum, we conclude that SpIREs are deficient for retrotransposition and likely represent evolutionary “dead-ends” in the L1 retrotransposition process.

## Results

### A polymorphic L1 likely resulted from the retrotransposition of a spliced L1 mRNA

Using fosmid-based discovery methods, we previously identified a polymorphic L1 (fosmid accession #AC225317) in the human population that contains a 524-nucleotide deletion within its 5′UTR [10]. Upon closer inspection, we determined that this deletion likely resulted from the retrotransposition of a spliced L1 RNA that used a previously identified SD (G_98_U_99_) [78] and an unreported SA (A_620_G_621_) within the L1 5′UTR (numbering based on L1.3, accession #L19088; [9,82]) (Fig 1A and 1B). The structure of this element resembled previous L1s characterized by Belancio and colleagues, supporting the hypothesis that spliced L1 transcripts can complete retrotransposition in the human genome [78,79]. We named these L1s SpIREs to distinguish them from bona fide full-length genomic L1s. The three SpIREs investigated here all use the same SD (G_98_U_99_) but use different SA sequences that reside within either the L1 5′UTR (SA: A_620_G_621_ or SA: A_788_G_789_) or L1 ORF1 (SA: A_974_G_975_) (Fig 1B, 1C and 1D).

### SpIREs are present in the human genome

We used the BLAST-like alignment tool (BLAT) (https://genome.ucsc.edu) [83] (in which transposable element—derived DNAs are not masked) to search the human genome reference (HGR, GRCh38/hg38) for SpIRE G_98_U_99_/A_620_G_621_ sequences (referred to as SpIRE_97/622_). The HGR contains an annotated record of L1s that have accumulated over evolutionary time (i.e., millions of years); thus, searching the genome should reveal how SpIREs contribute to the genomic L1 repertoire. We used a 100-nucleotide in silico probe that spans the intra-5′UTR splice junction present in SpIRE_97/622_ (nucleotides 47–97 and 622–672 of L1.3) to query the HGR. We identified 116 SpIRE_97/622_ sequences, which span the youngest L1PA1 subfamily (also known as L1Hs, members of which are currently amplifying in the human population) through the older L1PA6 subfamily (which amplified approximately 27 million years ago [MYA]), but none in older (L1PA7–L1PA17, L1PB, and L1MA) L1 subfamilies (S1A Fig) [84,85]. Thus, 116 out of 6,609 (about 1.8%) of previously annotated full-length L1s in the L1PA1–L1PA6 subfamilies are actually SpIRE_97/622_ sequences (S1 Fig; S1 Data; S1 Table).

Almost half of the SpIRE_97/622_ sequences we identified belong to the L1PA3 subfamily (53 sequences, comprising about 3.4% of previously annotated full-length L1s in that subfamily) (S1A Fig; S1 Data; S1 Table). The L1PA1 subfamily harbors six SpIRE_97/622_ (comprising about 2.0% of previously annotated full-length L1s in that subfamily) and the L1PA6 subfamily contains only one SpIRE_97/622_ (comprising 0.1% of previously annotated full-length L1s in that subfamily) (S1 Fig; S1 Data; S1 Table). Seven SpIRE_97/622_ sequences could not be unambiguously assigned to a specific L1 subfamily and are classified as either L1PA2–L1PA3 or L1PA4–L1PA6 sequences (S1 Fig; S1 Data; S1 Table) [86].

Given the above data, we used BLAT to search the HGR for additional L1s containing G_98_U_99_/A_788_G_789_ and G_98_U_99_/A_974_G_975_ splicing events identified by Belancio and colleagues (referred to as SpIRE_97/790_ and SpIRE_97/976_, respectively) [78,79]. These searches confirmed the presence of four previously identified SpIRE_97/790_ sequences in the L1PA1–L1PA2 subfamilies (S1A Fig; S1 Data; S1 Table) [78]. We also discovered an additional SpIRE_97/976_ sequence in addition to the ten previously identified SpIRE_97/976_ sequences (S1A Fig; S1 Data; S1 Table) [78,79]. In total, these three classes of SpIREs comprise a small but notable (131/6,609 or about 2%) percentage of previously annotated full-length L1s from the L1PA1–L1PA6 subfamilies. The SpIRE_97/622_ sequences discovered here represent the majority (116/131 or about 89%) of identified SpIREs.

### SpIREs contain L1 structural hallmarks

We next characterized the 131 SpIRE_97/622_, SpIRE_97/790_, and SpIRE_97/976_ sequences. We first examined the post-integration (i.e., filled) site of each SpIRE in the HGR sequence. We then used the genomic sequences flanking each SpIRE to reconstruct a putative pre-integration (i.e., empty) site. Many of the SpIRE sequences, especially those from older L1 subfamilies, have degenerate poly(A) tails at their 3′ ends, which, in some cases, made it difficult to reconstruct the putative pre-integration site to bp resolution (S1 Data; S1 Table). These analyses revealed that SpIREs generally are flanked by target site duplications that ranged in size from about 6–25 bp, end in a 3′ poly(A) tract, and integrated into an L1 EN consensus cleavage site (5′-TTTT/A-3′ and variants of that sequence) (S1 Data; S1 Table). Consistent with previous studies, approximately 39% (51/131) of the SpIREs are present within the introns of RefSeq (https://www.ncbi.nlm.nih.gov/refseq/) [87] annotated genes [69,88,89], and the majority (32/51 or about 63%) of these SpIREs are present in the opposite transcriptional orientation of the annotated gene (S1 Table) [90,91]. Other structural features of the SpIREs are shown in S1 Data and S1 Table. In sum, our analyses strongly suggest that SpIREs represent a subset of genomic L1 insertions and retrotranspose by the canonical process of L1 EN-dependent TPRT.

### Intra-5′UTR splicing reduces L1 promoter activity

**Fig 2:**
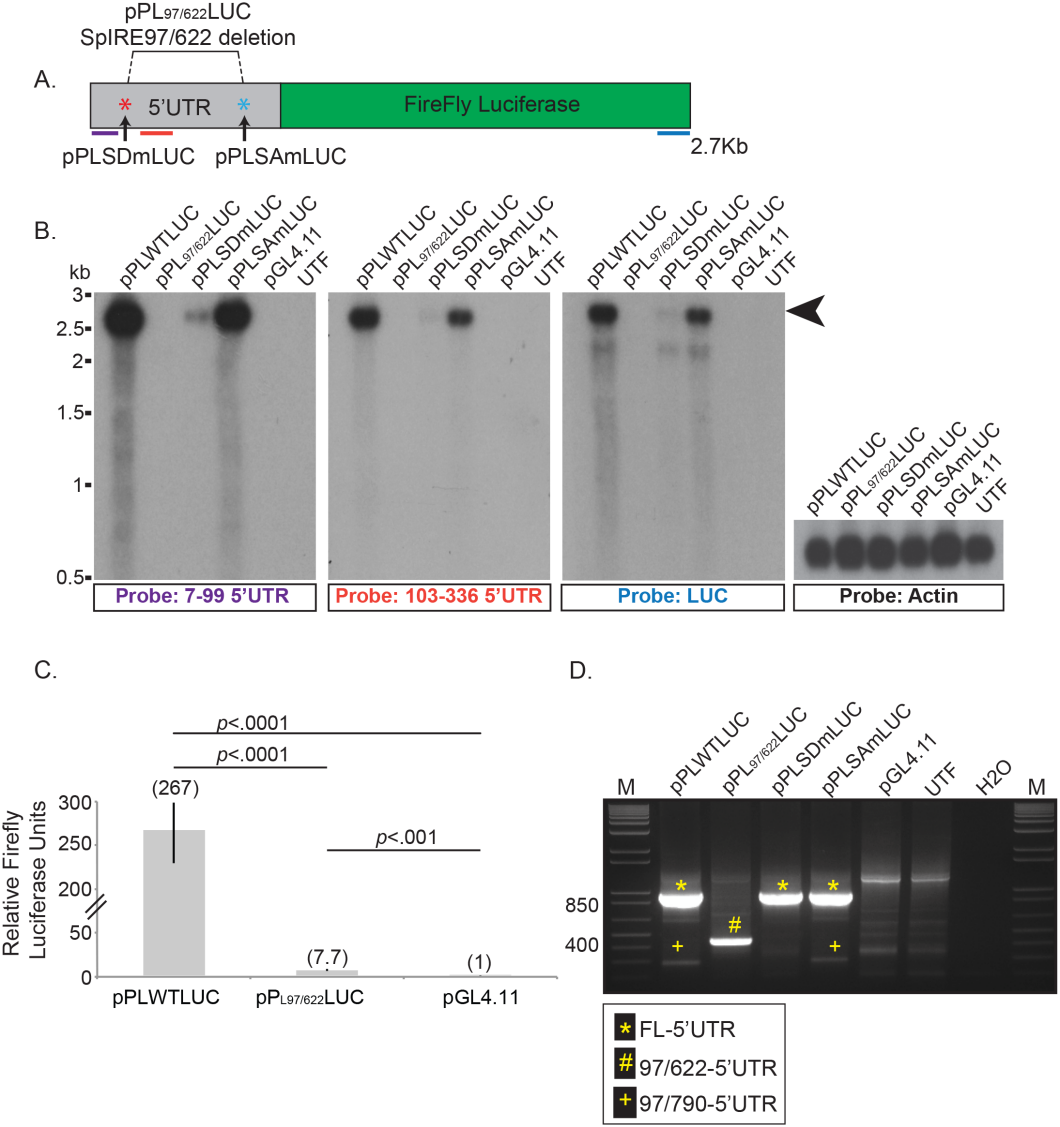
Intra-5′UTR splicing reduces L1 promoter activity. (A) Schematic of the luciferase constructs and the relative position of northern blot probes. The L1.3 5′UTR (gray rectangle) was used to drive the transcription of the firefly luciferase reporter gene (green rectangle) present in plasmid pGL4.11. The following plasmids were created: pPL_WT_LUC contains the full-length L1.3 5′UTR; pPL_97/622_LUC contains the SpIRE_97/622_ 5′UTR; pPL_SDm_LUC contains a U_99_C SD mutation (red asterisk) in the L1.3 5′UTR; pPL_SAm_LUC contains an A_620_C SA mutation (light blue asterisk) in the L1.3 5′UTR. The relative positions of complementary riboprobes used in the northern blot experiments (ribonucleotides 7–99 [purple line], ribonucleotides 103–336 [red line], and the 3′ end of the luciferase gene [blue line]) are indicated below the schematic. (B) Representative northern blots. The black arrowhead indicates the predicted size of full-length L1/luciferase mRNA (about 2.7 kb). Construct names are indicated above the gel lanes; UTF = untransfected HeLa-JVM cells. The probe used in the northern blot experiment is indicated below the autoradiograph. Actin served as an mRNA loading control (2.1 kb). RNA size standards (kb) (Millenium RNA Markers) are indicated to the left of the autoradiograph panels. (C) Results from the luciferase assays. The x-axis indicates the name of the luciferase expression plasmid. The y-axis indicates the relative firefly luciferase units normalized to a co-transfected Renilla luciferase internal control. These data represent the averages of three biological replicates (S2 Table). Each biological replicate contained six technical replicates. Error bars indicate the standard deviation between three biological replicates. *P*-values were determined using a Student one-tailed *t* test. (D) Results from RT-PCR assays: A 1.2% agarose gel depicting the results from a representative qualitative RT-PCR experiment. DNA size markers (1 kb Plus DNA Ladder) are indicated at the left of the gel. Plasmid names are indicated above the gel; UTF = untransfected HeLa-JVM cells, H2O = water control for PCR reactions. The inset to below the gel indicates the major (* and #) and minor (+) cDNA products detected in the experiments. FL, full-length; H2O, water control for PCR reactions; kb, kilobase; L1, Long interspersed element-1; M, marker; RT-PCR, reverse transcription PCR; SA, splice acceptor; SD, splice donor; SpIRE, spliced integrated retrotransposed element; UTF, untransfected HeLa-JVM cells; UTR; untranslated region; WT, wild-type.

The formation of SpIRE_97/622_ results in the deletion of five known transcription factor binding sites within the L1 5′UTR [47,92–97] (Fig 1A); thus, we hypothesized the SpIRE_97/622_ 5′UTR would have reduced promoter activity. To test this hypothesis, we subcloned the wild-type (WT) L1.3 [9,82] or SpIRE_97/622_ 5′UTR sequences upstream of a promoter-less firefly (*Photinus pyralis)* luciferase gene (vector pGL4.11), creating pPL_WT_LUC and pPL_97/622_LUC, respectively (Fig 2A). We then characterized the promoter activity of these 5′UTRs using functional assays.

We first conducted northern blot analyses using polyadenylated mRNAs isolated from untransfected HeLa-JVM cells and HeLa-JVM cells transfected with the luciferase expression vectors (Fig 2). An RNA probe complementary to ribonucleotides 7–99 of the L1 5′UTR (Fig 2A; purple line) detected a strong signal at the expected size of about 2.7 kb in mRNAs derived from HeLa-JVM cells transfected with pPL_WT_LUC, but not in mRNAs derived from HeLa-JVM cells transfected with pPL_97/622_LUC or pGL4.11 or from untransfected HeLa-JVM cells (Fig 2B, first panel). Similar results were obtained using riboprobes complementary to either ribonucleotides 103–336 of the L1 5′UTR (Fig 2A, red line; Fig 2B, second panel) or the 3′ end of luciferase (Fig 2A, blue line; Fig 2B, third panel). These data are consistent with previously published findings [47], which demonstrated that L1 transcription begins at or near the first nucleotide of the L1 5′UTR. Control experiments verified the integrity and quality of the mRNAs (Fig 2B, actin probe).

We were able to detect a faint band representing the predicted approximately 2.2 kb mRNA from HeLa-JVM cells transfected with pPL_97/622_LUC upon the prolonged exposure of the northern blots using probes complementary to ribonucleotides 7–99 of the L1 5′UTR, but not using a probe complementary to ribonucleotides 103–336 of the L1 5′UTR (S2A Fig; purple arrow). The absence of the predicted approximately 2.2-kb band in HeLa-JVM cells transfected with pPL_97/622_LUC using a probe complementary to the 3′ end of the luciferase gene is likely due to the limits of detection in our assay (S2A Fig). The origin of the approximately 2-kb transcript remains unclear (Fig 2B, S2A Fig, orange arrow); however, it could be representative of transcript initiation downstream of the canonical transcriptional start site within the 5′UTR [47,98]. These data suggest that the SpIRE_97/622_ 5′UTR retains weak promoter activity. Because the splicing events that gave rise to SpIRE_97/790_ and SpIRE_97/976_ led to larger deletions of the 5′UTR when compared to SpIRE_97/622_, we reasoned that they would lead to a similar, if not a greater, reduction in transcriptional activity; thus, they were not tested in this assay.

To corroborate the northern blot analyses, we conducted dual luciferase expression assays on whole cell lysates (WCLs) derived from HeLa-JVM cells co-transfected with firefly luciferase-based vectors (pPL_WT_LUC, pPL_97/622_LUC, or pGL4.11) and a constitutively expressed Renilla (*Renilla reniformis)* luciferase internal control plasmid (pRL-TK; Methods). Consistent with the northern blot data, HeLa-JVM cells transfected with pPL_WT_LUC exhibited an approximately 267-fold increase of normalized firefly luciferase activity when compared to cells transfected with the promoter-less pGL4.11 vector (Fig 2C; S2 Table). By comparison, HeLa-JVM cells transfected with pPL_97/622_LUC exhibited only about a 7-fold increase of normalized firefly luciferase activity when compared to cells transfected with the promoter-less pGL4.11 vector (Fig 2C; S2 Table). Together, the above data suggest that the splicing event leading to the generation of SpIRE_97/622_ severely compromises its promoter activity.

### Mutating the 5′ SD site results in decreased L1 promoter activity

Given that splicing reduces L1 promoter activity, we examined why the G_98_U_99_ SD may be conserved in the L1 5′UTR. Previous studies revealed that a RUNX3 binding site within the 5′UTR is important for maximal L1 promoter activity [96]. Interestingly, the SD site used to generate the three classes of SpIREs reported here is contained within the core sequence of a RUNX3 binding site that is conserved from the L1PA1–L1PA10 subfamilies (Fig 1A; SD: G_98_U_99_; S1B Fig) [84]. Thus, we hypothesized that this SD is retained to maintain an active RUNX3 transcription factor binding site. To test this hypothesis, we mutated the SD sequence within the WT 5′UTR (U_99_C, creating pPL_SDm_LUC) [99] and tested if this mutation affects 5′UTR promoter activity. Northern blot analyses using the previously described riboprobes detected a signal at about 2.7 kb in mRNAs derived from HeLa-JVM cells transfected with pPL_SDm_LUC. However, there is markedly less of this mRNA when compared to cells transfected with pPL_WT_LUC (Fig 2B; about 18% of pPL_WT_LUC). In contrast, mutating the SA site within the WT 5′UTR (A_620_C, creating pPL_SAm_LUC) did not drastically affect L1 promoter activity (Fig 2B). Thus, our data are consistent with previous findings [96] and suggest that the retention of the complete RUNX3 site containing the G_98_U_99_ SD is critical for L1 promoter activity.

### Reverse transcription PCR based detection of intra-5’UTR splicing events

We next sought to identify spliced L1 mRNAs that might have given rise to SpIREs. To this end, we conducted end-point reverse transcription PCR (RT-PCR) experiments using poly(A) mRNAs isolated from HeLa-JVM cells transfected with a series of L1/firefly luciferase expression vectors (S2B Fig; Methods). The REV-LUC oligonucleotide (S2B Fig, purple line) was used to initiate L1/firefly luciferase first-strand cDNA synthesis; the cDNA products then were PCR amplified using FWD-5′UTR (S2B Fig, red line) and REV-LUC (S2B Fig, purple line) oligonucleotide primers. The resultant cDNAs were separated on an agarose gel, cloned, and characterized using Sanger DNA sequencing. Control experiments conducted in the absence of RT revealed that the characterized PCR products were derived from the amplification of cDNAs (S2C Fig).

We detected the predicted full-length L1/firefly luciferase cDNA products from HeLa-JVM cells transfected with pPL_WT_LUC, pPL_SDm_LUC, and pPL_SAm_LUC (Fig 2D, yellow “*” in lanes 1, 3, and 4) as well as the shorter predicted L1/firefly luciferase cDNA product from HeLa-JVM cells transfected with pPL_97/622_LUC (Fig 2D, yellow “#” in lane 2). In agreement with our northern blot experiments (Fig 2B), we did not detect cDNAs consistent with SpIRE_97/622_ splicing in pPL_WT_LUC transfected HeLa-JVM cells (Fig 2D). However, we did detect an L1/firefly luciferase cDNA that corresponds to the SpIRE_97/790_ splicing event from cells transfected with pPL_WT_LUC and pPL_SAm_LUC (Fig 2D, yellow “+”, lanes 1 and 4; Fig 1C) [78]. Importantly, this product was not detected in HeLa-JVM cells transfected with either pGL4.11 or pPL_SDm_LUC or untransfected HeLa-JVM cells.

### Intra-5′UTR splicing does not dramatically affect L1 mRNA translation

**Fig 3:**
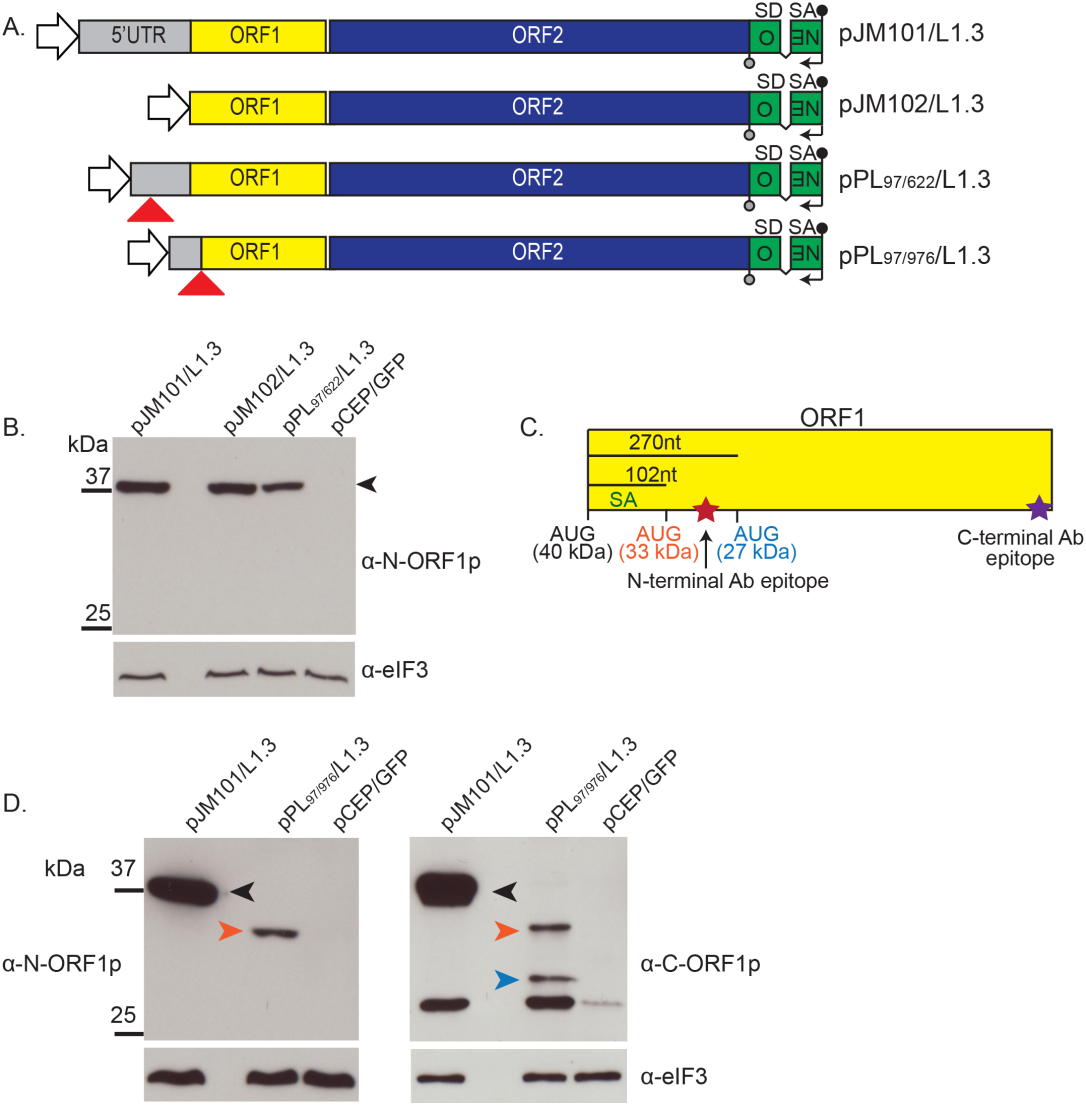
ORF1p expression from intra-5′UTR and 5′UTR/ORF1 SpIREs. (A) Schematics of the engineered L1 constructs. The L1 5′UTR (gray rectangle), ORF1 (yellow rectangle), and ORF2 (blue rectangle) are indicated in the constructs. Relative positions of the SpIRE_97/622_ and SpIRE_97/976_ deletions (red triangles) are indicated on the bottom two constructs, respectively. The CMV promoter (white arrowhead) and the *mneoI* retrotransposition indicator cassette (green rectangle = *neo* gene sequence; black “v” line = intron interrupting *neo* coding sequence, SD = splice donor site, SA = splice acceptor site) are indicated at the 5′ and 3′ ends of the constructs, respectively. The black lollipop at the 3′ end on top of the constructs indicates the sense SV40 polyadenylation signal. The black arrow and gray lollipop on the bottom of the constructs are embedded within the *mneoI* retrotransposition indicator cassette and indicate an SV40 early promoter and herpes simplex virus thymidine kinase polyadenylation signal, respectively, in the antisense orientation. (B) Representative ORF1p western blot from WCLs. Molecular weight standards (kDa) are indicated to the left of the image. The black arrowhead indicates the predicted size of full-length ORF1p (about 40 kDa). Construct names are indicated above the image; pCEP/GFP = negative control. The antibody used in the western blot experiment is indicated to the right of the gel (α-N-ORF1p). The eIF3 protein (110 kDa) served as a loading control. Western blots were performed three times, yielding similar results. (C) Schematic of ORF1 and relative location of antibody binding. Top: The relative positions in ORF1 (yellow rectangle) of the SA sequence at nucleotides 974–975 (green), the canonical ORF1 initiator methionine (AUG, black, 40 kDa), the two putative initiator methionine codons (AUG, orange, 33 kDa; AUG, blue, 27 kDa), and the N- and C-terminal epitopes recognized by the ORF1p Ab (red and purple stars, respectively) are indicated in the figure. (D) Representative western blots from WCLs: molecular weight standards (kDa) are indicated to the left of the gels. The predicted sizes of full-length ORF1p (black arrowhead) and the N-terminal truncated ORF1p variants (orange and blue arrows, respectively) are highlighted on the gel. Construct names are indicated above the image; pCEP/GFP = negative control. The antibodies used in the western blot experiments are indicated to the left (α-N-ORF1p) and right (α-C-ORF1p) of the gel images, respectively. The eIF3 protein (110 kDa) served as a loading control. The unlabeled band at about 25 kDa in the α-C-ORF1p experiment is an unknown cross-reacting product that was not detected in RNPs or with an antibody to a C-terminal ORF1p T7-*gene10* epitope tag (S4A Fig and S4B Fig). Western blots were performed three times, yielding similar results. α-C-ORF1p, C-terminal ORF1p antibody; α-elF3, eukaryotic initiation factor 3 antibody; α-N-ORF1p, N-terminal ORF1p antibody; Ab, antibody; AUG, translation initiation codon; CMV, cytomegalovirus; kDa, kilodalton; L1, Long interspersed element-1; ORF, open reading frame; SA, splice acceptor; SD, splice donor; SpIRE, spliced integrated retrotransposed element; UTR, untranslated region; WCL, whole cell lysate.

We next tested whether intra-5′UTR splicing affects L1 mRNA translation. L1 sequences were cloned into an episomal pCEP4 expression vector that contains a hygromycin B resistance gene and a cytomegalovirus (CMV) early promoter, which augments L1 expression. HeLa-JVM cells were transfected with a WT L1 (pJM101/L1.3), an L1 that contains a 5′UTR deletion (pJM102/L1.3), or an L1 containing the SpIRE_97/622_ deletion (pPL_97/622_/L1.3) (Fig 3A) [9,50].

Western blot analyses were conducted using WCLs that were derived from hygromycin-resistant HeLa-JVM cells transfected with the above constructs 9 days post-transfection. An ORF1p polyclonal antibody (α-N-ORF1p; directed against amino acids +31 to +49 in L1.3 [100] [UniProtKB accession #Q9UN81]) detected an approximately 40-kDa product in cells transfected with pJM101/L1.3, pJM102/L1.3, and pPL_97/622_/L1.3 but not in cells transfected with the pCEP/GFP control (Fig 3B). HeLa-JVM cells transfected with pPL_97/622_/L1.3 exhibited a slight reduction in the steady-state level of ORF1p when compared to HeLa-JVM cells transfected with pJM101/L1.3 or pJM102/L1.3 (Fig 3B). Because a CMV promoter augmented L1 transcription, it is unlikely that this reduction is due to reduced L1 expression. It is possible that the slight reduction in ORF1p is due to an alteration of the L1 5′UTR RNA secondary structure and/or minor changes in the stability of pPL_97/622_/L1.3 mRNA when compared to pJM101/L1.3 and pJM102/L1.3 mRNAs.

### 5′UTR/ORF1 splicing leads to amino-terminal truncated ORF1p

The splicing event yielding SpIRE_97/976_ results in an amino-terminal ORF1 deletion of 66 nucleotides, including the canonical ORF1p methionine start codon (Fig 3C, black AUG, 40 kDa). We hypothesized that ORF1p synthesis might initiate from two methionine codons (AUG) that are located in weak Kozak consensus sequences either 102 or 270 ribonucleotides downstream from the canonical AUG start codon (Fig 3C) [101]. If the downstream methionine codons are used for translation initiation, we expect to detect amino terminal truncated ORF1 proteins of about 33 kDa and 27 kDa, respectively.

Western blot analyses were conducted as above using WCLs derived from hygromycin-resistant HeLa-JVM cells transfected with pJM101/L1.3, an L1 containing the SpIRE_97/976_ deletion (pPL_97/976_/L1.3), or pCEP/GFP control vectors (Fig 3A) [22]. As predicted, the α-N-ORF1p and α-C-ORF1p antibodies detected an approximately 40-kDa protein in WCLs derived from HeLa-JVM cells transfected with pJM101/L1.3 but did not detect a protein in WCLs derived from HeLa-JVM cells transfected with the pCEP/GFP control (Fig 3D, left and right panels). The α-N-ORF1p antibody detected an approximately 33-kDa protein in WCLs derived from HeLa-JVM cells transfected with pPL_97/976_/L1.3 (Fig 3D, left panel), whereas the α-C-ORF1p antibody detected approximately 33-kDa and approximately 27-kDa proteins in the same extracts and an unknown cross-reacting protein at about 25 kDa (Fig 3D, right panel). Similar results were obtained when RNP extracts were used in western blot experiments, although western blots performed with the α-C-ORF1p antibody did not detect the cross-reacting approximately 25-kDa protein (S3A Fig). To confirm that the approximately 33-kDa and 27-kDa products were ORF1p derived, we introduced a T7-*gene10* epitope tag to the 3′ end of ORF1, creating pPL_97/976_/L1.3-T7. Western blots using a α-T7 antibody recapitulated our previous results and, similar to RNP preparations, did not identify the cross-reacting approximately 25-kDa protein (S3B Fig). Thus, the 5′UTR/ORF1 splicing event leads to the generation of an mRNA that, if translated, results in the synthesis of amino-terminal truncated derivatives of ORF1p.

### Intra-5′UTR splicing decreases subsequent rounds of L1 retrotransposition

Our data indicate that SpIRE_97/622_ contains a defective promoter and, if transcribed, SpIRE_97/622_ mRNA is translated at slightly lower levels than WT L1 mRNA. Thus, we hypothesized that an intra-5′UTR spliced L1 mRNA would be capable of undergoing an initial round of L1 retrotransposition. However, the resultant full-length retrotransposition events would contain a defective promoter, which may compromise subsequent retrotransposition.

To test the above hypothesis, we examined whether RNAs derived from a cohort of L1 expression constructs could retrotranspose using a cultured cell retrotransposition assay [31]. The 3′UTR of each construct contains a retrotransposition indicator cassette (*mneoI*). The *mneoI* cassette consists of an antisense copy of a neomycin phosphotransferase gene whose coding sequence is interrupted by an intron that resides in the same transcriptional orientation as the L1 [31,102]. This arrangement ensures that the expression of a functional neomycin phosphotransferase gene will only be activated upon L1 retrotransposition, thereby conferring cellular resistance to the drug G418 [31,102]. Retrotransposition efficiency then can be quantified by counting the resultant numbers of G418-resistant foci [31,61].

**Fig 4:**
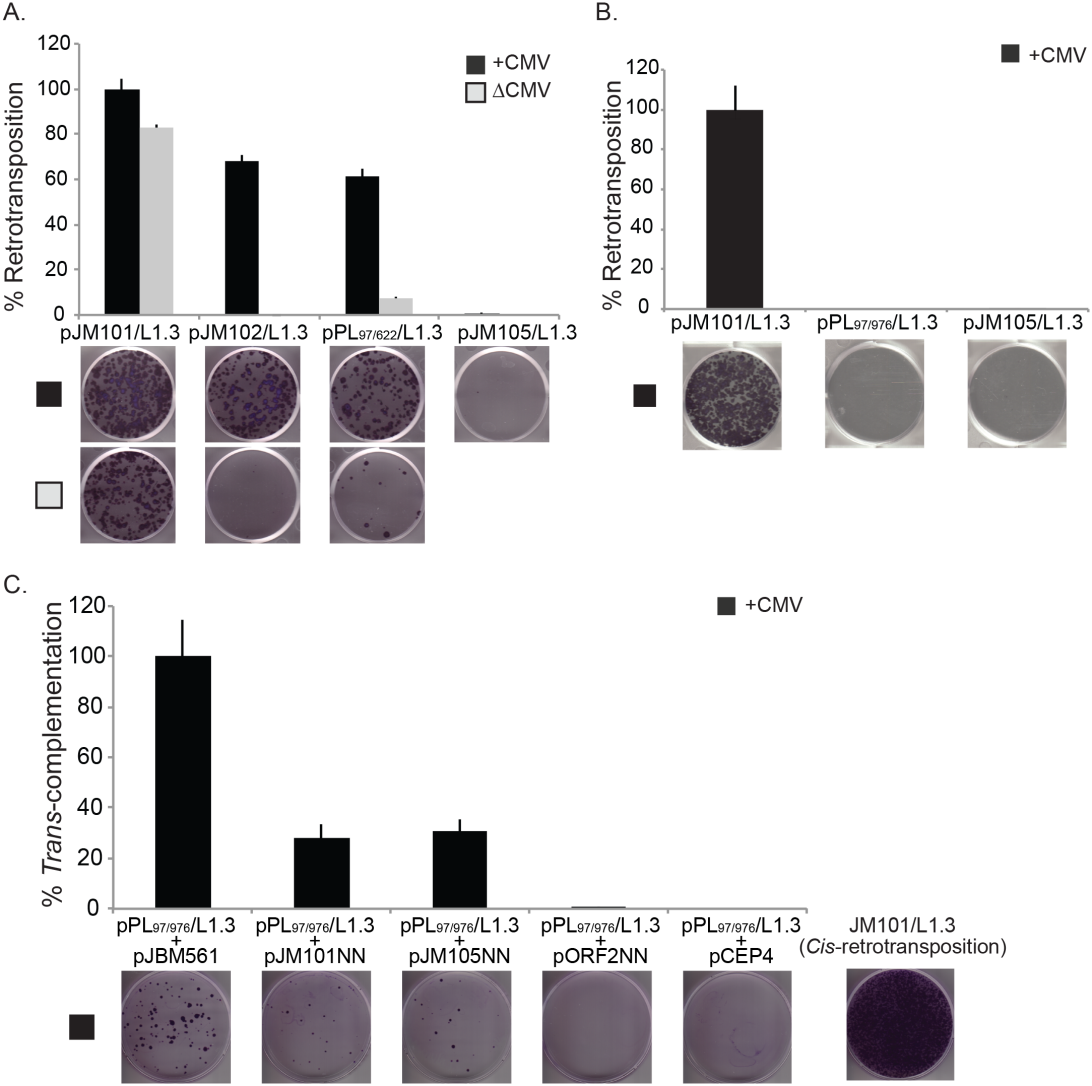
Intra-5′UTR and 5′UTR/ORF1 SpIREs are retrotransposition-defective. (A) Results from the SpIRE_97/622_ retrotransposition assay. The x-axis indicates the construct names. The y-axis indicates the relative retrotransposition efficiency (%). The CMV promoter either augments L1 expression (+CMV, black bars) or is absent (ΔCMV, gray bars) from the L1 expression construct. The relative retrotransposition efficiencies are normalized to pJM101/L1.3 (set at 100%). The pJM105/L1.3 plasmid served as a negative control. The images and data are from one representative experiment (S3 Table). Error bars represent the standard deviation of technical triplicates for the depicted assay. Each assay was repeated three times, yielding similar results. (B) Results from the SpIRE_97/976_ retrotransposition assay. The x-axis indicates the construct names. The y-axis indicates the relative retrotransposition efficiency (%). A CMV promoter augments L1 expression (+CMV, black bars). The relative retrotransposition efficiencies are normalized to pJM101/L1.3 (set at 100%). The pJM105/L1.3 plasmid served as a negative control. The images and data are from one representative experiment (S4 Table). Error bars represent the standard deviation of technical triplicates for the depicted assay. Each assay was repeated three times, yielding similar results. (C) Results from the SpIRE_97/976_ *Trans*-complementation assay. The x-axis indicates the “reporter” (top text) and the “driver” (bottom text) construct names. The y-axis indicates the relative *Trans*-complementation efficiency (%). The results of each assay were normalized to the pPL_97/976_/L1.3 “reporter” plasmid + pJBM561 “driver plasmid” co-transfection experiment, which was set at 100%. The image at the bottom right-hand side of the figure represents the efficiency of pJM101/L1.3 retrotransposition in *cis*. The pPL_97-976_/L1.3 “reporter” plasmid + pCEP4 “driver plasmid” co-transfection experiment served as a negative control. The images and data are from one representative experiment (S5 Table). Error bars represent standard deviations of technical triplicates for the depicted experiment. Each assay was repeated four times, yielding similar results. CMV, cytomegalovirus; L1, Long interspersed element-1; ORF, open reading frame; SpIRE, spliced integrated retrotransposed element; UTR, untranslated region.

Consistent with previous reports (e.g., [31,41]), mRNAs derived from RC-L1s that contain both CMV and 5′UTR (Fig 4A, pJM101/L1.3, black bar; S3 Table), CMV only (Fig 4A, pJM102/L1.3, black bar; S3 Table), or 5′UTR only (Fig 4A, pJM101/L1.3ΔCMV, gray bar; S3 Table) promoters could efficiently retrotranspose. By comparison, the pPL_97/622_/L1.3 expression construct produced mRNAs that could undergo efficient retrotransposition when a CMV promoter augmented L1 expression (Fig 4A, black bar, about 70% the activity of pJM101/L1.3; S3 Table), but not when L1 expression was driven from the 5′UTR harboring the intra-5′UTR splicing event (Fig 4A, pPL_97/622_/L1.3ΔCMV gray bar, about 7% the activity of pJM101/L1.3; S3 Table). Consistent with this observation, control experiments revealed that an L1 lacking promoter sequences (Fig 4A, pJM102/L1.3ΔCMV; S3 Table) [50] was unable to retrotranspose. Additional controls demonstrated that an L1 containing a missense mutation (pJM105/L1.3; D702A) that disrupts ORF2p RT activity [50] severely reduced L1 retrotransposition efficiency (Fig 4A; S3 Table). Thus, the data suggest that the SpIRE_97/622_ intra-5′UTR splicing event severely compromises L1 5′UTR promoter activity as well as subsequent rounds of L1 retrotransposition.

### 5′UTR/ORF1 SpIREs rely on ORF1p supplied in *trans* for mobilization

The retrotransposition of an mRNA derived from a 5′UTR/ORF1 splicing event would generate a SpIRE (e.g., SpIRE_97/976_) that contains a defective promoter and, if transcribed and translated, would produce amino-terminal truncated versions of ORF1p. If the truncated version(s) of ORF1p were nonfunctional, we reasoned that the 5′UTR/ORF1 splicing event would lead to an L1 mRNA that is compromised for an initial round of retrotransposition in *cis*. Indeed, RNAs derived from pPL_97/976_/L1.3 could not retrotranspose despite expression being driven by CMV (Fig 4B; S4 Table).

We next hypothesized that a source of WT ORF1p would be required to act in *trans* to promote the retrotransposition of L1 mRNAs containing a 5′UTR/ORF1 splicing event. To test this hypothesis, we co-transfected pPL_97/976_/L1.3 (whose expression is augmented by a CMV promoter) with a series of “driver” L1 expression plasmids that lack the *mneoI* retrotransposition indicator cassette [22,50]. The co-transfection of pPL_97/976_/L1.3 with “driver” plasmids that express WT ORF1p, pJBM561 (a monocistronic ORF1p expression vector), pJM101/L1.3NN, or pJM105/L1.3NN, resulted in low levels of pPL_97/976_/L1.3 RNA retrotransposition in *trans* (Fig 4C; columns 1, 2, and 3, respectively; S5 Table). By comparison, the co-transfection of pPL_97/976_/L1.3 with “driver” plasmids that do not express ORF1p (pORF2/L1.3NN [a monocistronic ORF2p expression vector] or pCEP4) did not support retrotransposition in *trans* (Fig 4C; columns 4 and 5, respectively; S5 Table) [22]. Thus, the expression of ORF1p, but not ORF2p, can promote low levels of retrotransposition of mRNAs derived from pPL_97/976_/L1.3 in *trans*.

### Structural changes within the 5′UTR alter L1 splicing dynamics

RT-PCR experiments using L1/firefly luciferase expression vectors uncovered evidence of SpIRE_97/790_ splicing events (Fig 2D). Intriguingly, SpIRE_97/790_ sequences are only present in the L1PA1 and L1PA2 subfamilies (S1A Fig; S1 Data; S1 Table) [84]. Indeed, the analysis of 1,000 genomes data [103] revealed that the L1PA1 SpIRE_97/790_-3 sequence (S1 Data; S1 Table) is polymorphic with respect to presence in the human population (about 41% homozygous “filled”; 35% heterozygous; 24% homozygous “empty”), whereas L1PA2 SpIRE_97/790_ sequences appear to be fixed with respect to presence in humans. Additionally, we identified four non-reference L1PA1 SpIRE_97/790_ sequences in data from the 1000 Genomes Project (S1 Data; S1 Table). Thus, SpIRE_97/790_ sequences may represent an evolutionarily younger SpIRE subfamily than the SpIRE_97/622_ and SpIRE_97/976_ sequences, which are predominantly found in older L1 subfamilies (S1 Data; S1 Table).

**Fig 5:**
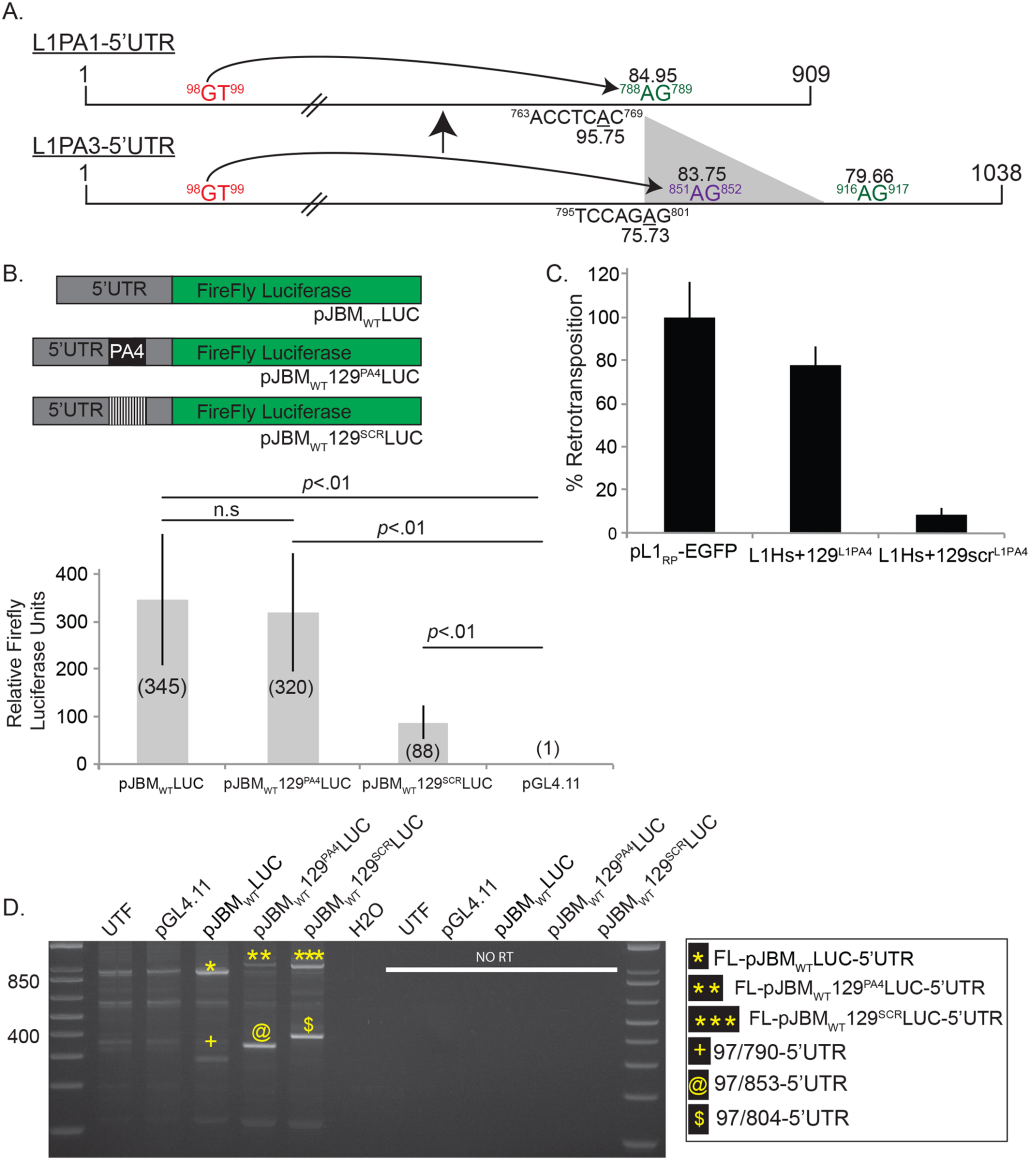
Sequence changes within the 5′UTR affect intra-5′UTR splice site choice. (A) Schematic of the L1PA1 and L1PA3 5′UTRs. Top schematic, the relative positions of the SD (red lettering), SA (green lettering), and putative branch point sequence (ACCTCAC, black lettering) in the L1PA1 5′UTR that led to the formation of SpIRE_97/790_ are indicated in the schematic. Superscript numbers indicate the first and last nucleotide of the indicated sequence. Note that nucleotide positions are indicated in the context of L1.3 (accession #L19088). Numbers below the branch point (underlined A; 95.75) and above the SA A_788_G_789_ (84.95) indicate the predicted score of those sequences for utilization in a splicing reaction, as determined using Human Splicing Finder v.3.0 (http://www.umd.be/HSF3/) [105]. Note that predicted scores above 80 are considered “strong” [105]. Bottom schematic, the relative positions of the SD (red lettering), SAs A_851_G_852_ (purple lettering), A_916_G_917_ (green lettering), and putative branch point sequence (TCCAGAG, black lettering) in the L1PA3 5′UTR are indicated in the schematic. Superscript numbers indicate the first and last nucleotide of the indicated sequence. Numbers below the branch point (underlined A; 75.73) and SAs A_851_G_852_ (83.75) and A_916_G_917_ (79.66) indicate the predicted strength of those sequences for utilization in a splicing reaction, as determined using Human Splicing Finder v.3.0 (http://www.umd.be/HSF3/) [105]. The L1PA3 5′UTR contains a 129-bp sequence (gray triangle) containing the SA A_851_G_852_ that was lost in the transition from the L1PA3 to L1PA2/L1PA1 subfamilies. The 129-bp deletion results in repositioning the SA A_916_G_917_ in L1PA3 to closer proximity of a putative branch point in the L1PA2/L1PA1 subfamilies 5′UTR (now noted as A_788_G_789_ in the top schematic), leading to a higher predicted score (84.95 in PA1 compared to 79.66 in PA3). (B) Schematic of luciferase constructs and results from luciferase assays. Top panel: the L1_RP_ 5′UTR (gray rectangle) was used to drive the transcription of the firefly luciferase reporter gene (green rectangle) present in plasmid pGL4.11. The following plasmids were created: pJBM_WT_LUC contains the full-length L1_RP_ 5′UTR; pJBM_WT_129^PA4^LUC contains the 129-bp (black box in 5′UTR) sequence derived from L1PA4 within the L1_RP_ 5′UTR; pJBM_WT_129^SCR^LUC contains a scrambled version of the 129-bp sequence (black and white striped box) within the 5′UTR. Bottom panel: luciferase assay. The x-axis indicates the name of the luciferase expression plasmid. The y-axis indicates the relative firefly luciferase units normalized to a co-transfected Renilla luciferase internal control. These data represent the averages of three biological replicates (S6 Table). Each biological replicate contained six technical replicates. Error bars indicate the standard deviation between three biological replicates. *P*-values were determined using a Student one-tailed *t* test and “n.s.” indicates that there was no statistical difference. (C) Results from the EGFP retrotransposition assay: the x-axis indicates the construct names. The y-axis indicates the relative retrotransposition efficiency (%). The relative retrotransposition efficiencies are normalized to pL1_RP_-EGFP (set at 100%). The data are from one representative experiment (S7 Table). Error bars represent the standard deviation of technical triplicates for the depicted assay. Each assay was repeated four times, yielding similar results. (D) Results from RT-PCR assays: a 2.0% agarose gel depicting the results from a representative qualitative RT-PCR experiment. DNA size markers (1 kb Plus DNA Ladder) are shown at the left of the gel. Plasmid names are indicated above the gel; UTF = untransfected HeLa-JVM cells, H2O = water control for PCR reactions. The right half of the agarose gel (“NO RT”) indicates the results from a representative experiment conducted without the addition of reverse transcriptase. The inset to the right of the gel indicates the major (*, **, and ***) and minor (+, @, and $) cDNA products detected in the experiments. The assay was repeated four times, yielding similar results. H2O, water control for PCR reactions; L1, Long interspersed element-1; RT-PCR, reverse transcription PCR; SA, splice acceptor; SD, splice donor; SpIRE, spliced integrated retrotransposed element; UTF, untransfected HeLa-JVM cells; UTR, untranslated region.

Recently, an elegant study from the Haussler laboratory demonstrated that the Krüppel-associated Box-containing Zinc-Finger Protein 93 (ZNF93) could bind within L1PA3 and L1PA4 5′UTRs to repress their expression [104]. Intriguingly, a 129-bp deletion that eliminates the ZNF93 binding site within the L1PA2 and L1PA1 5′UTRs allowed them to evade ZNF93-mediated repression [104]. This 129-bp sequence resides between a putative branch site and the SA sequence used to generate the spliced L1 RNA that gave rise to SpIRE_97/790_ sequences (Fig 5A). Thus, we hypothesized this 129-bp deletion may have altered L1 5′UTR splicing dynamics by relocating the SpIRE_97/790_ SA (A_916_G_917_ in L1PA3) to a favorable splicing context in L1PA2 and L1PA1 subfamily members (Fig 5A).

To test the above hypothesis, we generated L1/firefly luciferase expression vectors containing the 5′UTR of a “hot” L1 (L1_RP_ [accession #AF148856]) [106] or a version of the L1_RP_ 5′UTR that includes the 129-bp L1PA4 sequence containing the ZNF93 binding site [104] upstream of a promoter-less firefly luciferase gene (pGL4.11), creating pJBM_WT_LUC and pJBM_WT_129^PA4^LUC, respectively (Fig 5B, top panel). We also created a control vector that has a “scrambled” version of the 129-bp L1PA4 sequence (pJBM_WT_129^SCR^LUC) [104] (Fig 5B, top panel). Dual luciferase assays using WCLs derived from HeLa-JVM cells co-transfected with pJBM_WT_LUC, pJBM_WT_129^PA4^LUC, pJBM_WT_129^SCR^LUC, or pGL4.11 and a constitutively expressed Renilla luciferase internal control plasmid (pRL-TK; Methods) revealed that pJBM_WT_LUC and pJBM_WT_129^PA4^LUC exhibited an increase (about 345- or about 320-fold, respectively) of normalized firefly luciferase activity, when compared to the promoter-less pGL4.11 vector (Fig 5B, bottom panel; S6 Table). By comparison, pJBM_WT_129^SCR^LUC exhibited a significant, though less pronounced, increase (about 88-fold) of normalized firefly luciferase activity (Fig 5B, bottom panel; S6 Table). Thus, in general agreement with previous studies [104], the 129-bp L1PA4 insert does not negatively affect L1_RP_5′UTR transcriptional activity. As an additional control, we confirmed that the 129-bp L1PA4 sequence did not significantly affect L1 activity using an EGFP-based retrotransposition assay (Fig 5C; S7 Table) [104].

To test whether the presence or absence of the 129-bp L1PA4 sequence affects intra-L1 5′UTR splicing, we used a slightly modified version of the end-point RT-PCR strategy depicted in Fig 2D. In agreement with experiments performed with pPL_WT_LUC (Fig 2D), we detected the predicted full-length L1_RP_ 5′UTR cDNAs as well as SpIRE_97/790_ spliced cDNAs in cells transfected with pJBM_WT_LUC (Fig 5D, yellow “*” and yellow “+,” respectively, lane 3). By comparison, HeLa-JVM cells transfected with pJBM_WT_129^PA4^LUC yielded the predicted full-length 5′UTR L1 cDNA (Fig 5C, yellow “**” lane 4), but did not yield cDNAs corresponding to the SpIRE_97/790_ splicing event. Instead, we detected a new spliced cDNA that used the same G_98_U_99_ SD and a new SA that resides within the 129-bp L1PA4 sequence (A_851_G_852_), which is not present in the WT L1_RP_ sequence (Fig 5A and 5D, lane 4, yellow “@”). Finally, we detected the predicted full-length L1_RP_ 5′UTR cDNAs from cells transfected with pJBM_WT_129^SCR^LUC, as well as a biologically irrelevant product that utilized the same G_98_U_99_ SD and an SA that resides within the 129-bp L1PA4 scrambled sequence (Fig 5D, lane 5, yellow “***” and yellow “$,” respectively). Thus, our data demonstrate that the loss of the 129-bp sequence from L1PA3 resulted in a new splicing pattern that led to the emergence of SpIRE_97/790_ sequences (Fig 5A and 5D).

Finally, we examined whether the new cDNA detected from cells transfected with pJBM_WT_129^PA4^LUC corresponds to a SpIRE. Indeed, a BLAT search of the human genome using an in silico probe that spans the intra-5′UTR splice junction present in this putative SpIRE (nucleotides 47–97 and 853–903 of pJBM_WT_129^PA4^LUC) yielded nine additional SpIRE_97/853_ sequences (S1 Data; S1 Table). These additional SpIREs retain L1 structural hallmarks (S1 Data; S1 Table), indicating that canonical EN-dependent TPRT led to their generation.

## Discussion

The evolutionary success of L1 requires the continued retrotransposition of full-length L1 RNAs. Thus, it was surprising when Belancio and colleagues identified a small number of L1 retrotransposition events in the HGR that apparently were derived from spliced L1 RNAs [78,79]. Here, we confirmed and extended those findings and report a novel group of retrotransposed L1s that are derived from an L1 RNA containing an intra-5′UTR splicing event (SpIRE_97/622_; Fig 1). SpIRE_97/622_ is 10 times more prevalent than previously identified SpIREs and comprises about 1.8% of the annotated full-length L1 retrotransposition events accumulated during the past approximately 27 million years (MY) (S1 Fig).

**Fig 6:**
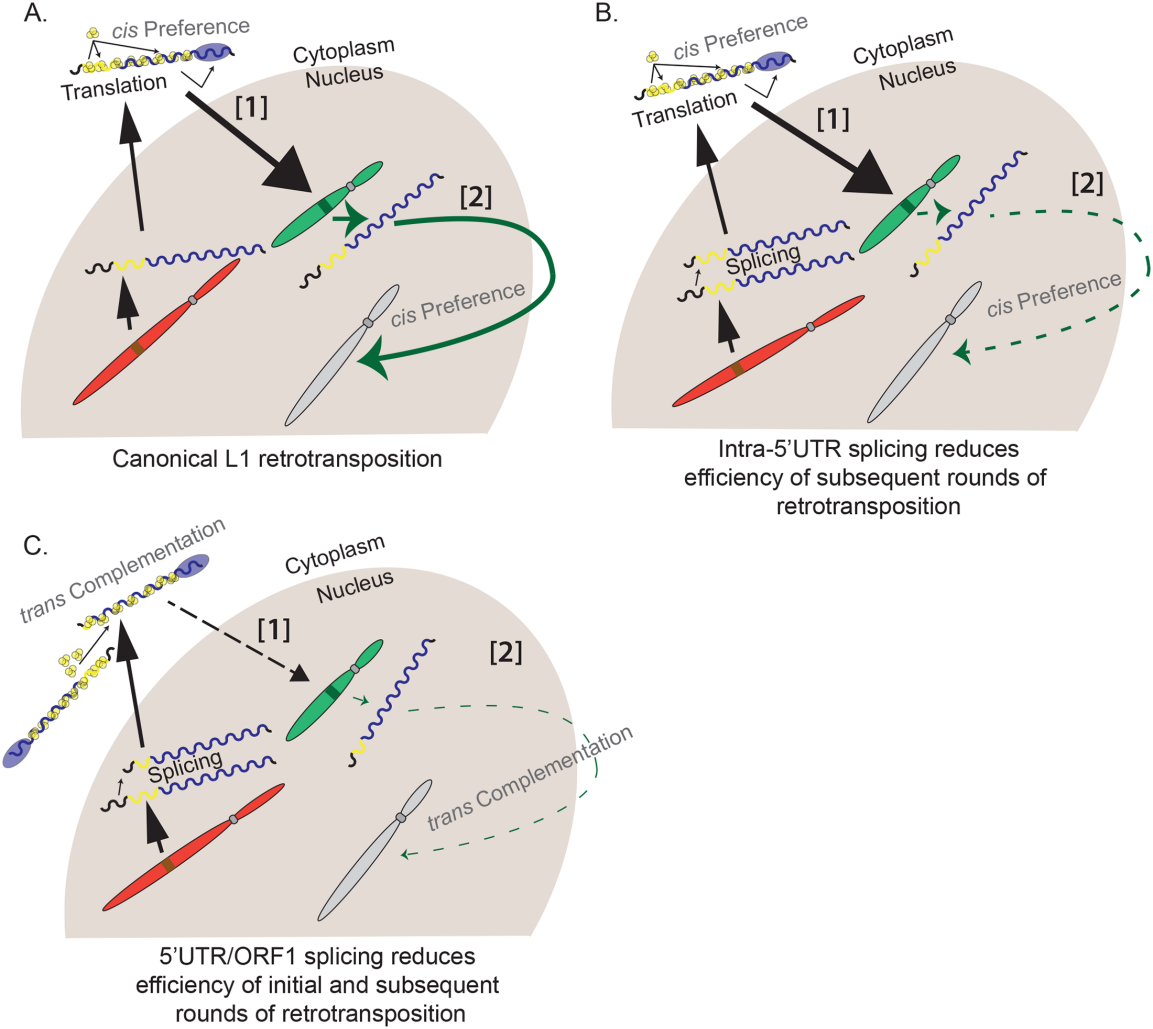
A working model for the generation of SpIREs. (A) Canonical L1 retrotransposition. An L1 is transcribed from a genomic location (red chromosome). Translation of the mRNA (multicolored wavy line) occurs in the cytoplasm and ORF1p (yellow circles) and ORF2p (blue oval) bind back onto their respective mRNA (*Cis*-preference) to form an RNP. The L1 RNP then enters the nucleus and a de novo L1 insertion occurs at a new genomic location (green chromosome) by TPRT. This insertion, if full length, could act as a source element, giving rise to new insertions (green arrow) at a new genomic location (gray chromosome). (B) Retrotransposition of intra-5′UTR spliced L1 isoform. A full-length L1 element is transcribed from its genomic location (red chromosome) and undergoes intra-5′UTR splicing. Translation of the mRNA (multicolored wavy line) occurs in the cytoplasm and ORF1p (yellow circles) and ORF2p (blue oval) bind back onto their respective mRNA (*Cis*-preference) to form an RNP. The L1 RNP then enters the nucleus and L1 mRNAs subject to intra-5′UTR splicing can undergo a single round of retrotransposition (green chromosome) by TPRT. However, because the intra-5′UTR splicing event deletes sequences required for L1 promoter activity, the resultant insertion is unlikely to undergo subsequent rounds of retrotransposition in future generations (dashed green arrow). (C) Retrotransposition of 5′UTR/ORF1 spliced L1 isoform. An L1 is transcribed from its genomic location (red chromosome) and is subject to 5′UTR/ORF1 splicing. Translation of the mRNA (multicolored wavy line) occurs in the cytoplasm; however, because translation occurs at downstream AUG codons, ORF1p (yellow circles) is truncated and nonfunctional, the 5′UTR/ORF1 spliced L1 mRNA relies on a wild-type source of ORF1p to be supplied from another L1 in *trans*. In the rare instance that *Trans*-complementation occurs (dotted arrow), it is highly unlikely that the resultant SpIRE will generate RNAs that can undergo retrotransposition in future generations (dashed thin green arrow). L1, Long interspersed element-1; ORF, open reading frame; RNP, ribonucleoprotein particle; SpIRE, spliced integrated retrotransposed element; TPRT, target-site primed reverse transcription; UTR, untranslated region.

Numerous studies have demonstrated that L1 ORF1p and L1 ORF2p exhibit *Cis*-preference and preferentially bind to their encoding mRNA to promote its retrotransposition (Fig 6A) [33,35,38,50–53]. Using a cell culture based retrotransposition assay in HeLa cells, we demonstrated that L1 mRNAs that contain intra-5′UTR splicing events can produce ORF1p and ORF2p and undergo an initial round of retrotransposition in *cis* (Fig 6B). However, the resultant SpIREs lack *Cis*-acting sequences required for efficient L1 transcription (Fig 2) [47,94,96] and, as a result, are compromised for subsequent rounds of retrotransposition (Figs 4A and 6B).

In contrast to intra-5′UTR splicing events, L1 mRNAs containing 5′UTR/ORF1 splicing events produce nonfunctional, amino-terminal truncated versions of ORF1p (Fig 3C; S3A and S3B Fig). As a result, these mRNAs are retrotransposition defective in *cis* and must rely on exogenous sources of ORF1p to promote their retrotransposition by *Trans*-complementation (Figs 4B and 4C and 6C). Notably, these experiments also provide genetic evidence that ORF2p can be translated from the 5′UTR/ORF1 spliced L1 mRNAs. In the rare cases in which *Trans*-complementation occurs, the resultant 5′UTR/ORF1 SpIRE will lack *Cis*-acting sequences required for efficient L1 transcription and, if transcribed, would produce nonfunctional versions of ORF1p. The loss of *Cis*-acting sequences and the requirement for *Trans*-complementation make it highly unlikely that the resultant 5′UTR/ORF1 SpIREs could undergo subsequent rounds of retrotransposition (Fig 6C).

The above data strongly indicate that SpIREs represent evolutionary “dead ends” in the L1 amplification process. It is possible that a small number of SpIREs could give rise to new L1 retrotransposition events. For example, the insertion of a SpIRE_97/622_ downstream of a cellular promoter could, in principle, enhance its expression and subsequent retrotransposition. However, any resultant retrotransposition event would contain a defective promoter and ultimately be compromised for subsequent rounds of retrotransposition. Thus, we conclude that splicing negatively affects L1 retrotransposition.

The SpIREs examined in this study each use a common SD site (G_98_U_99_) but different SA sites (A_620_G_621_, A_788_G_789_, A_851_G_852_, or A_974_G_975_) [78,79]. These findings raise the following question: if splicing adversely affects L1 retrotransposition, why are these splice sites retained in L1 RNA? The G_98_U_99_ SD site is about 46 MY old, is conserved in the L1PA1–L1PA10 subfamilies (S1B Fig) [84], and resides within a core binding site for the RUNX3 transcription factor [96]. Indeed, previous studies indicated that mutating U_99_ in the L1 5′UTR impairs RUNX3 binding and decreases 5′UTR transcriptional activity [96]. Consistent with these findings, we found that mutating the SD sequence leads to an approximately 5-fold reduction in L1 steady-state RNA levels (Fig 2B). Together, these data strongly suggest that the benefit conferred by the RUNX3 transcription factor binding site at the DNA level outweighs the cost of harboring the SD site (G_98_U_99_) in L1 RNA.

Despite the evolutionary conservation of the G_98_U_99_ SD, northern blotting experiments revealed that the vast majority of L1 5′UTRs are not subject to splicing (Fig 2B). SpIREs are therefore most likely formed when L1 RNAs containing rare splicing events undergo retrotransposition. The reason(s) G_98_U_99_ is not efficiently utilized as a functional SD site requires elucidation. However, it is possible that the G_98_U_99_ sequence is sequestered into a secondary structure within L1 RNA that restricts its access to U1 small nuclear RNA (snRNA) (reviewed in [107,108]). Alternatively, a cellular protein(s) might bind at or near the SD site, thereby blocking its ability to interact with U1 snRNA. Either scenario provides a plausible mechanism for how L1 maintains a functional SD sequence in its mRNA and could, in part, explain why SpIREs only represent about 2% of annotated full-length L1 retrotransposition events that occurred during the past approximately 27 MY.

SA sites within the 5′UTR might also reside in functional transcription factor binding sites or functionally conserved regions of ORF1p. For example, the A_788_G_789_ SA is about 70 MY old and is conserved through the L1PA15B subfamily (S1B Fig), suggesting that it may reside in a conserved *Cis*-acting motif. The ORF1 A_974_G_975_ SA resides at codon positions two and three of lysine 22, and any nucleotide change at codon position two would result in an amino acid substitution in ORF1p that may adversely affect its function. Thus, it is possible that some functional splice sites are embedded in sequences that are critical for 5′UTR and/or ORF1p function.

Our data reveal how host-factor—driven L1 5′UTR evolution can alter L1 splicing dynamics. We demonstrated that structural changes in the 5′UTR can lead to collateral intra-5′UTR splicing changes, which have resulted in the generation of new SpIRE_97/790_ sequences (Fig 5A and 5D). In addition to yielding insights into the evolution of human L1 5′UTR sequences, these experiments demonstrate the utility of our luciferase-based reporter constructs to prospectively detect ancestral L1 splicing events that led to the generation of an older SpIRE (SpIRE_97/853_) subfamily (Fig 5A and 5D; S1 Data; S1 Table). Although the SpIRE_97/622_ sequence is the most abundant SpIRE in the HGR, only SpIRE_97/790_ sequences were detected in our RT-PCR experiments. These data, as well as the finding that five of eight SpIRE_97/790_ sequences are polymorphic with respect to presence/absence in the human population, suggest that SpIRE_97/790_ sequences are currently amplifying in modern human genomes.

It is noteworthy that the splicing events detected from engineered L1 mRNAs in transfected HeLa cells recapitulate many splicing events that led to SpIRE formation in the human genome (Figs 2D and 5D, and [78,79]). It has recently been shown that a small number of distinct genomic L1 loci are expressed in a cell type—specific manner [6–8]. Moreover, L1 splicing and/or premature polyadenylation patterns vary among human tissues and cell types [79,80,109,110], host proteins involved in splicing and polyadenylation associate with L1 RNPs [100,111–113], and overexpression of the Epstein-Barr Virus SM protein alters L1 splicing and premature polyadenylation patterns [79]. Thus, it is tempting to speculate that L1 posttranscriptional processing may suppress expression and/or retrotransposition of full-length L1s in a developmental or cell type—specific manner.

In sum, our data strongly indicate that L1 mRNA splicing is detrimental to L1 retrotransposition and further strengthen the hypothesis that ORF1p and ORF2p predominantly retrotranspose their encoding full-length L1 RNAs to new genomic locations in *cis*. In addition, we demonstrated that despite harboring evolutionarily conserved functional SD and SA sites within their 5′UTR, the vast majority of L1 transcripts apparently evade splicing. Finally, we provide experimental evidence revealing that changes within the L1 5′UTR, which are driven by the escape from host-factor repression, lead to collateral changes in L1 splicing profiles. Together, these data provide insights into the evolutionary dynamics of the L1 5′UTR and raise the intriguing possibility that host factors that promote L1 splicing or alter L1 splicing profiles may represent a mechanism by which the cell can disrupt full-length L1 RNA to prevent unabated L1 retrotransposition.

## Methods

### E. coli and the propagation of plasmids

All plasmids were propagated in DH5α *Escherichia coli* (genotype: F- φ80*lac*ZΔM15 Δ(*lac*ZYA-*arg*F) U169 *rec*A1 *end*A1 *hsd*R17 (rk-, mk+) *pho*A *sup*E44 λ- *thi*-1 *gyr*A96 *rel*A1) (Invitrogen, Carlsbad, CA). Competent cells were generated as described previously [114]. Plasmids were prepared using the Plasmid Midi Kit (Qiagen, Germany) according to the protocol provided by the manufacturer.

### Cell lines and cell culture conditions

HeLa-JVM cells (obtained from Dr. Maxine Singer and originally cited in reference [31]) were cultured in high glucose Dulbecco’s Modified Eagle Medium (DMEM) lacking pyruvate (Invitrogen). DMEM was supplemented with 10% fetal bovine calf serum (FBS) and 1X penicillin/streptomycin/glutamine to create DMEM-complete medium, as described previously [31]. HeLa-JVM cells were grown in a humidified tissue culture incubator (Thermo Scientific, Waltham, MA) at 37°C in the presence of 7% CO_2_.

### BLAT searches and SpIRE sequence curation

BLAT [83] was used to screen build 38 (GRCh38/hg38) of the UCSC genome browser (https://genome.ucsc.edu) using 100 bp in silico probes that spanned (50 bases upstream and downstream) the splice junctions of SpIRE_97/622_, SpIRE_97/790_, and SpIRE_97/976._ The in silico probes were designed using the L1.3 sequence (accession #L19088 [9,82]) as a template. A 100-bp in silico probe that spanned (50 bases upstream and downstream) the splice junction of SpIRE_97/853_ was designed using the pJBM_WT_129^PA4^LUC sequence. Putative SpIREs shared >95% sequence identity with the in silico probes.

Putative SpIREs were downloaded from the UCSC genome browser and manually curated with the aid of repeat masker (http://repeatmasker.org). Each sequence was inspected to ensure it contained a splicing event and represented a bona fide SpIRE. For four events that were prematurely 3′ truncated, we analyzed 4 kb of genomic DNA flanking the 3′ end of the SpIRE to determine if it shared >95% sequence identity with L1.3 using the Serial Cloner alignment tool (http://serialbasics.free.fr/Serial_Cloner.html). We were unable to identify any L1 sequence in the flanking DNA; thus, we cannot determine the reason for the apparent 3′ truncation in these four SpIREs. Structural hallmarks of L1 integration events that occur by canonical TPRT (e.g., the presence of target site duplications, the presence of untemplated nucleotides at the 5′ genomic DNA/L1 junction [47,69,89,115], a 3′ poly(A) tract, and putative L1-mediated sequence transductions) [23,116,117] were determined manually by analyzing sequences flanking the 5′ and 3′ ends of each SpIRE [69,116,118]. The L1 “empty site” for all SpIREs is inferred; the 3′ TSD was considered the ancestral “empty site” and any nucleotide differences between the 5′ and 3′ TSD are annotated in the 5′ TSD only. Sequences are named based on the splicing event contained within the SpIRE (SpIRE_97/622_, SpIRE_97/790_, SpIRE_97/976_, or SpIRE_97/853_) and a corresponding number for easy referral between S1 Data and S1 Table (for example; SpIRE_97/622_-1 is the first of the analyzed 116 SpIRE_97/622_ sequences).

### Determining the conservation of SD and SA sites

Khan et al. 2006 provided full-length L1 subfamily consensus sequences of L1PA1 (L1Hs) through L1PA16 and assembled an alignment of the respective 5′UTRs [84]. We manually inspected these alignments to determine the oldest L1 subfamily that contained the 5′UTR SD/SA sequences utilized in generating the reported SpIREs. We next determined the conservation of the ORF1 SA sequence (A_974_G_975_) by aligning full-length L1 consensus sequences provided in Khan et al. 2006 using the ClustalW alignment function [84,119] from the MegAlign (http://www.dnastar.com/t-megalign.aspx) software. As with the 5′UTR, we manually inspected the resulting alignment to determine the oldest L1 subfamily that contained the ORF1 SA sequence (A_974_G_975_).

### Identification of putative branch point sequences

To identify putative splicing branch point sequences, we utilized the L1.3 5′UTR (accession #L19088) sequence and the pJBM_WT_129^PA4^LUC 5′UTR sequence and submitted them for analysis using the Human Splicing Finder v3.0 online prediction program (http://www.umd.be/HSF3/HSF.html) [105]. The resultant analyses identify potential SD, SA, and branch point sequences and assign consensus value scores for each motif [105]. Motif scores greater than 80 represent “strong” splice sites; sequences with scores less than 80 represent “weaker” splice sites. The 5′UTR sequence of each L1 was uploaded and analyzed by the general “Analyze a Sequence” function. We then selected predicted branch points that might pair with the known SA: A_788_G_789_ (L1.3) and A_851_G_852_ (pJBM_WT_129^PA4^LUC) based on their proximity to the SA sequence [120]. We identified a putative branch point (A_763_C_764_C_765_T_766_C_767_A_768_C_769_) with a score of 95.75 that could pair with the SA: A_788_G_789_ in the L1.3 5′UTR. We also identified a putative branch point (T_795_C_796_C_797_A_798_G_799_A_800_G_801_) with a score of 75.73 that could pair with the SA: A_851_G_852_ in the pJBM_WT_129^PA4^LUC 5′UTR (Fig 5A).

### Genotyping and discovery of non-reference SpIREs

We performed in silico genotyping of four SpIRE_97/790_ loci using reads from the 1000 Genomes Project [103,121]. Read pairs anchored within 600 bp of each locus were extracted from each of 2,453 samples from the 1000 Genomes Project. Extracted read pairs were aligned to reconstructed reference (insertion) and alternative (empty site) sequences and the most likely genotype for each sample was determined based on the number and mapping quality of read pairs aligned to each allele [121]. Read pairs that aligned entirely within the L1 sequence as well as read pairs that show equivalent alignments to both the reference and alternative sequences were ignored in the analysis.

We utilized an anchored read pair mapping approach to identify additional non-reference SpIRE_97/790_ insertions in the 1000 Genomes Project samples. We searched alignment files from 2,453 samples for read pairs in which one read is aligned across the splice junction in one of the four SpIRE_97/790_ sequences represented in the genome reference sequence and the other read is uniquely aligned elsewhere in the genome. We then intersected the resulting anchored locations with a recently published map of non-reference L1 insertions discovered in the same samples [122], identifying four insertions supported by multiple SpIRE-associated read pairs. To further characterize these loci, we extracted insertion-supporting read pairs for each locus and performed a de novo read assembly using the CAP3 assembler [123]. This analysis results in a collection of short contigs for each locus, which extend into the flanking edges of each inserted L1 element. The resulting contigs were filtered for repeat content, aligned to the genome reference, and annotated for characteristics indicative of bona fide SpIRE_97/790_ insertions (S1 Data and S1 Table).

### L1 expression constructs

The following L1 constructs contain a derivative of an RC-L1 (L1.3, accession #L19088 [9,82]) cloned into the pCEP4 plasmid backbone (Life Technologies), unless indicated otherwise. Cloning strategies used to create these constructs are available upon request.

pJM101/L1.3 contains a full-length version of L1.3 in the pCEP4 backbone. The 3′UTR of L1.3 contains the *mneoI* retrotransposition indicator cassette [9,31,82].

pJM101/L1.3ΔCMV is identical to pJM101/L1.3, but the CMV promoter was deleted from the pCEP4 plasmid [9,31,82].

pJM101/L1.3NN is a derivative of pJM101/L1.3 that lacks the *mneoI* retrotransposition indicator cassette [50].

pDK101/L1.3 is a derivative of pJM101/L1.3 that expresses a version of ORF1p that contains a T7 *gene10* epitope tag on its carboxyl-terminus [35].

pJM105/L1.3 is identical to pJM101/L1.3, but contains a D702A missense mutation in the ORF2p RT active site [50].

pJM105NN is a derivative of pJM105/L1.3 that lacks the *mneoI* retrotransposition indicator cassette [50].

pJM102/L1.3 is a derivative of pJM101/L1.3 that lacks the L1 5′UTR [58].

pJM102/L1.3ΔCMV is identical to pJM102/L1.3, but the CMV promoter was deleted from the pCEP4 plasmid [50].

pPL_97/622_/L1.3 is a derivative of pJM101/L1.3 that contains a 524 intra-5′UTR deletion (L1.3 nucleotides 98–621) present in SpIRE_97/622_ [10].

pPL_97/622_/L1.3ΔCMV is identical to pPL_97-622_/L1.3, but the CMV promoter was deleted from the pCEP4 plasmid.

pPL_97/976_/L1.3 is a derivative of pJM101/L1.3 that contains an 878-bp 5′UTR/ORF1 deletion (L1.3 nucleotides 98–975) present in SpIRE_97/976_.

pPL_97/976_/L1.3-T7 is a derivative of pPL_97-976_/L1.3 that expresses a version of ORF1p that contains a T7 *gene10* epitope tag on its carboxyl-terminus.

pORF2/L1.3NN is a monocistronic L1 ORF2 expression plasmid that lacks the *mneoI* retrotransposition indicator cassette [22].

pJBM561 is a monocistronic L1 ORF1 expression plasmid that lacks the *mneoI* retrotransposition indicator cassette.

pCEP/GFP is a pCEP4-based plasmid that expresses a humanized Renilla green fluorescent protein (hrGFP) from phrGFP-C (Stratagene). A CMV promoter drives the expression of the *hrGFP* gene [22].

The following L1 constructs contain a derivative of an RC-L1 (L1_RP_, accession #AF148856.1 [124]) cloned into the pCEP4 plasmid backbone (Life Technologies) lacking the CMV promoter.

pL1_RP_-EGFP contains a full-length version of L1_RP_ element. The 3′UTR contains the EGFP retrotransposition indicator cassette [124].

pL1_RP_(JM111)-EGFP: a derivative of pL1_RP_-EGFP that contains two missense mutations in ORF1 that abolish retrotransposition [31,124].

L1Hs+129^L1PA4^: a derivative of pL1_RP_-EGFP that carries a 129-bp sequence element from the L1PA4 5′UTR that is not present in L1Hs [104].

L1Hs+129scramble^L1PA4^: a derivative of pL1_RP_-EGFP that carries a scrambled version of the 129-bp sequence element from the L1PA4 5′UTR that is not present in L1Hs [104].

### Luciferase expression constructs

The following plasmids are based on the pGL4.11 promoter-less firefly luciferase expression vector (Promega, Madison, WI). Oligonucleotides and cloning strategies used to create these constructs are available upon request.

pPL_WT_LUC is a derivative of pGL4.11 that contains the WT L1.3 5′UTR upstream of the firefly luciferase reporter gene.

pPL_97/622_LUC is a derivative of pGL4.11 that contains the pPL_97-622_/L1.3 5′UTR deletion derivative upstream of the firefly luciferase reporter gene.

pPL_SDm_LUC is a derivative of pPL_WT_LUC that contains a U_99_C SD mutation in the L1.3 5′UTR upstream of the firefly luciferase reporter gene.

pPL_SAm_LUC is a derivative of pPL_WT_LUC that contains an A_620_C SA mutation in the L1.3 5′UTR upstream of the firefly luciferase reporter gene.

pRL-TK is an expression plasmid where the HSV-TK promoter drives Renilla luciferase transcription (Promega).

pJBM_WT_LUC is a derivative of pGL4.11 that contains the L1_RP_ 5′UTR from the plasmid pL1_RP_-EGFP [124] and was cloned upstream of the firefly luciferase reporter gene.

pJBM_WT_129^PA4^LUC is a derivative of pGL4.11 that contains the 5′UTR from L1Hs+129^L1PA4^ [104] and was cloned upstream of the firefly luciferase reporter gene.

pJBM_WT_129^SCR^LUC: the 5′UTR from L1Hs+129scramble^L1PA4^ [104] that was cloned upstream of the firefly luciferase reporter gene.

### RNA isolation

RNA isolation was performed as previously described with minor modifications [100]. Briefly, 8×10^6^ HeLa-JVM cells were seeded into a T-175 Falcon tissue culture flask (BD Biosciences, San Jose, CA). On the following day, transfections were conducted using the FuGene HD transfection reagent (Promega, Madison, WI). The transfection reactions contained 1 mL of Opti-MEM (Life Technologies), 120 μl of the FuGene HD transfection reagent, and 20 μg of plasmid DNA per flask. The tissue culture medium was changed 24 hours post-transfection. The cells were collected 48 hours post-transfection. Briefly, cells were washed in ice-cold 1X phosphate buffered saline (PBS) (Life Technologies). The cells then were scraped from the tissue culture flasks, transferred to a 15-mL conical tube (BD Biosciences), and centrifuged at 3,000 × *g* for 5 minutes at 4°C. Cell pellets were frozen at −20°C overnight. The frozen pellets were thawed and total RNA was prepared using the TRIzol reagent following the protocol provided by the manufacturer (Life Technologies). Poly(A) RNAs then were isolated from the total RNAs using a Oligotex mRNA Midi Kit (Qiagen), suspended in UltraPure DNase/RNase-Free distilled water (Thermo Fisher Scientific, Waltham, MA), and quantified using a NanoDrop 1000 spectrophotometer (Thermo Fisher Scientific). For the RT-PCR experiments in Fig 5D, total RNA was collected using an RNeasy kit (Qiagen), and polyadenylated RNA was isolated from total RNA using Dynabeads Oligo (dT)_25_ (Ambion).

### Northern blots

Northern blot experiments were performed as previously described [100]. Briefly, Northern blot experiments were conducted using the NorthernMax-Gly Kit (Thermo Fisher Scientific) following the protocol provided by the manufacturer. Briefly, aliquots of poly(A) RNAs (2 μg) were incubated for 30 minutes at 50°C in Glyoxal Load Dye (containing DMSO and ethidium bromide) and then were separated on a 1.2% agarose gel. The RNAs were transferred by capillary action to a Hybond-N nylon membrane (GE Healthcare, Marlborough, MA) for 4 hours and cross-linked to the membrane using the Optimum Crosslink setting of a Stratalinker (Stratagene, La Jolla, CA). Membranes were then baked at 80°C for 15 minutes. Membranes were prehybridized for approximately 4 hours at 68°C in NorthernMax Prehybridization/Hybridization Buffer (Thermo Fisher Scientific) and then were incubated overnight at 68°C with a strand-specific RNA probe (final concentration of probe, 3×10^6^ cpm/ml). The following day, the membranes were washed once with low stringency wash solution (2x saline sodium citrate (SSC), 0.1% sodium dodecyl sulfate [SDS]) and then twice with high stringency wash solution (0.1x SSC, 0.1% SDS). The washed membranes were placed in a film cassette (Thermo Fisher Scientific, Autoradiography Cassette FBCA 57) and exposed to Amersham Hyperfilm ECL (GE Healthcare) overnight at −80°C. Films were developed using a JP-33 X-Ray Processor (JPI America Inc., New York, NY).

### Preparation of northern blot probes

Northern blot probes were prepared as previously described [100]. Strand-specific αP^32^-UTP radiolabeled riboprobes were generated using the MAXIscript T3 system (Thermo Fisher Scientific). Briefly, oligonucleotide primers were used to PCR amplify portions of the L1.3 5′UTR [100] (L1.3 nucleotides 7–99 or L1.3 nucleotides 103–336) or the 3′ end of the luciferase gene (see below). The resultant PCR products were separated on a 1% agarose gel and were purified using QIAquick gel extraction (Qiagen). The labeling reaction was carried out at 37°C using the following reaction conditions: 500 ng of gel purified DNA template, 2 μL of transcription buffer supplied by the manufacturer, 1 μL each of unlabeled 10 mM ATP, CTP, and GTP, 5 μL of αP^32^-UTP (10 mCi/mL), and 2 μL of T3 RNA polymerase. The reaction components then were mixed and brought to a total volume of 20 μL using nuclease-free water in a 1.5-mL Eppendorf tube, which was incubated at 37°C for 10 minutes in a heating block. Unincorporated nucleotides were subsequently depleted using the Ambion NucAway Spin Columns (Thermo Fisher Scientific) following the protocol provided by the manufacturer. To generate a control β-actin riboprobe, the pTRI-β-actin-125-Human Antisense Control Template (Applied Biosystems) was used in T3 labeling reactions. Biological triplicates of each northern blot exhibited similar results.

Oligonucleotide sequences were used to generate northern blot probes. A T3 RNA polymerase promoter sequence was included on the antisense (AS) primer used to generate the antisense riboprobe (underlined below):

L1.3 5′UTR 7–99 Sense: 5′-GGAGCCAAGATGGCCGAATAGGAACAGCT-3′

L1.3 5′UTR 7–99 AS: 5′-AATTAACCCTCAAAGGGACCTCAGATGGAAATGCAG-3′

L1.3 5′UTR 103–336 Sense: 5′-GGGTTCATCTCACTAGGGAGTG-3′

L1.3 5′UTR 103–336 AS: 5′-AATTAACCCTCACTAAAGGGTATAGTCTCGTGGTGCGCCG-3′

Luciferase 3′ FFLuc Sense: 5′-GGCAAGATCGCCGTGAATTCTCAC-3′

Luciferase 3′ FFLuc AS: 5′-AATTAACCCTCACTAAAGGGCCTGGCGCTGGCGCAAGCAGC-3′

### Quantification of northern blots

Northern blot bands were quantified using the ImageJ software (https://imagej.nih.gov/ij/download.html) [125]. The intensity of the bands in the pPL_WT_LUC and pPL_SDm_LUC lanes were determined and normalized to the actin loading control. Three independent northern blots were subject to quantification. We then computed that average intensity of the bands and calculated a standard deviation. As reported in the text (Fig 2B), the steady-state level of pPL_SDm_LUC mRNA is about 18% the level of pPL_WT_LUC mRNA with a standard deviation of ±3.1%.

### Dual luciferase assays

Luciferase assays were performed using the Dual-Luciferase Reporter Assay System (Promega, Madison, WI) following the manufacturers protocol. Briefly, 2×10^4^ HeLa cells were plated into each well of a 6-well plate (BD Biosciences). Approximately 24 hours later, each well was transfected using a transfection mixture of 100 μl Opti-MEM (Life Technologies), 3 μl of FuGENE6 transfection reagent (Promega), and 1 μg plasmid DNA (0.5 μg of a firefly luciferase test plasmid and 0.5 μg of an internal control Renilla luciferase expression). Each transfection was performed as a technical duplicate (i.e., in two wells of a 6-well tissue culture plate). Approximately 24 hours post-transfection, the transfected cells were washed once with ice-cold 1X PBS and the cells in each well were subjected to lysis for 15 minutes at room temperature using 500 μl of the 1X Passive Lysis Buffer supplied by the manufacturer. Following homogenization of the lysate by manual pipetting, 60 μl of the lysate from each well of the 6-well tissue culture plate was distributed equally in 3 wells of a 96-well white opaque, optically transparent top plate (BD Biosciences), allowing six luminescence readings for each transfection condition (six technical replicates—3 readings per well of a 6-well plate). The 96-well plate then was subject to luciferase detection assays using a GloMax-Multi Detection System (Promega) following the manufacturer’s protocol. Luminescence readings from the six technical replicates were averaged to give a single normalized luminescence reading (NLR). This assay then was performed in biological triplicate, yielding three independent NLRs. The resultant data were subsequently analyzed using a Student one-tailed *t* test. Error bars indicate the standard deviation. Luminescence readings from lysis buffer alone and from lysates derived from untransfected cells were included used as negative controls.

### RT-PCR

Poly(A) selected mRNA from transfected HeLa-JVM cells in a T-175 tissue culture flask was collected as previously described for northern blots. The resultant mRNAs were subjected to targeted RT-PCR using SuperScript III One-Step RT-PCR System, with Platinum *Taq* DNA Polymerase (Thermo Fisher Scientific), following the manufacturer’s protocol. The REVLUC primer was used to synthesize first-strand cDNA. The FWD5′UTR and REVLUC primers then were used to amplify the resultant cDNAs (see sequences below). For RT-PCR experiments in Fig 5D, cDNA was synthesized from polyadenylated RNA with a SuperScript First-Strand Synthesis System for RT-PCR (Invitrogen) using the REVLUC primer. The resultant cDNA was then subjected to PCR using the FWD5′UTR and REVLUC primers and Platinum Taq DNA polymerase (Invitrogen) (30 cycles; annealing temp: 54°C; 1-minute extension). The RT-PCR products were separated on a 2.0% agarose gel, excised from the gel using QIAquick gel extraction (Qiagen), and cloned using the TOPO TA Cloning Kit (Thermo Fisher Scientific). Sanger DNA sequencing performed at the University of Michigan DNA Sequencing Core verified the cDNA sequences in the resultant plasmids. Biological triplicates of this experiment yielded similar results.

The following oligonucleotide sequences were used in the RT-PCR experiments:

FWD5′UTR: 5′-GGAACAGCTCCGGTCTACAGCTCCC-3′

REVLUC′ 5′-CCCTTCTTAATGTTTTTGGCATCTTCC-3′

### Protein collection

The plating and transfection of HeLa-JVM cells in T-175 tissue culture flasks was performed as detailed above in the mRNA isolation section except that HeLa-JVM cells were subjected to selection in DMEM-complete medium supplemented with 200 μg/ml of hygromycin B (Thermo Fisher Scientific) 48 hours post-transfection and the selection medium was changed every other day for 7 days. The hygromycin resistant HeLa-JVM cells were harvested 9 days post-transfection as described in the mRNA isolation section. The cell pellets were frozen at −80°C overnight. The following day, pellets were lysed for 15 minutes on ice by incubation in 0.5 mL of lysis buffer: 10% glycerol, 20 mM Tris-HCl pH 7.5, 150 mM NaCl, 0.1% NP-40 (IGPAL) (Sigma-Aldrich, St. Louis, MO), and 1X Complete Mini EDTA-free Protease Inhibitor Cocktail (Roche Applied Science, Germany). The resultant protein lysates then were centrifuged at 15,000 × *g* for 30 minutes to clear the lysate. The resultant supernatant (approximately 0.4 mL) was designated as the WCL. Alternatively, the supernatant fraction was subject to RNP collection, as previously described [35]. Briefly, 200 μL of the WCL was layered onto a sucrose solution cushion (6 mL of 17% sucrose, bottom layer, followed by 4 mL of 8.5% sucrose, top layer, overlaid by 200 μL of the WCL) and ultracentrifuged at 178,000 × *g* for 2 hours at 4°C. Following ultracentrifugation, the supernatant was aspirated and the resultant RNP pellet was suspended in 100 μL of water supplemented with 1X Complete Mini EDTA-free Protease Inhibitor Cocktail (Roche Applied Science). Bradford assays (Bio-Rad Laboratories, Hercules, CA) were used to determine protein concentrations. WCLs generally yielded 15–19 μg/μL of protein. RNP preparations yielded 6–10 μg/μL of protein. The protein samples were stored at −80°C.

### Western blots

Western blot experiments were performed as previously described, with minor modifications [100]. Briefly, protein samples were collected as described above and then were incubated with a 2X solution of NuPAGE reducing buffer (containing 1.75%–3.25% lithium dodecyl sulfate and 50 mM dithiothreitol [DTT]) (ThermoFisher Scientific). An aliquot (20 μg) of the reduced proteins were incubated at 100°C for 10 minutes and then were separated by electrophoresis on 10% precast mini-PROTEAN TGX gels (Bio-Rad Laboratories, Hercules, CA) run at 200 V for 1 hour in 1X Tris/Glycine/SDS (25 mM Tris-HCL, 192 mM glycine, 0.1% SDS, pH 8.3) buffer (Bio-Rad Laboratories). Transfer was performed using the Trans-Blot Turbo Mini PVDF Transfer Packs (BioRad Laboratories) with the Trans-Blot Turbo Transfer System (BioRad Laboratories) at 25 V for 7 minutes. The resultant membranes then were cut at the 75-kDa marker using the Precision Plus Protein Kaleidoscope marker (Bio-Rad Laboratories) as a guide. The membranes then were incubated at room temperature in blocking solution (containing 1X PBS and 5% dry low-fat milk) (Kroger, Cincinnati, OH). The eIF3 antibody (Santa Cruz Biotechnology Inc. [SC-28858]) was used at a 1:1,000 dilution to probe membranes for eIF3 at 110 kDa as a loading control. The α-N-ORF1p [100] antibody (directed against ORF1p amino acids 31–49; EQSWMENDFDELREEGFRR), α-C-ORF1p (directed against ORF1p amino acids 319–338; EALNMERNNRYQPLQNHAKM), and anti-T7 (Merck Millipore 69048 T7•Tag Antibody HRP Conjugate) antibodies were used at 1:10,000, 1:2,000, and 1:5,000 dilutions, respectively, to probe membranes for ORF1p. Antibody hybridizations were carried out overnight at 4°C in blocking solution. The blots were washed three times with 1X PBS, 0.1% Tween-20 (Sigma Aldrich) and then were incubated with a 1:5,000 dilution of secondary Amersham ECL HRP Conjugated Donkey anti-rabbit IgG Antibodies (GE Healthcare Life Sciences) for 60 minutes at room temperature blocking solution. The membranes were washed three times with 1X PBS, 0.1% Tween-20 (Sigma Aldrich). The signals then were visualized using the SuperSignal West Pico Chemiluminescent Substrate reagent (ThermoFisher Scientific) according to the protocol provided by the manufacturer. The membranes were exposed to Amersham Hyperfilm ECL (GE Healtchare) for a time that spanned 5 seconds to 5 minutes and were developed using a JP-33 X-Ray Processor (JPI America Inc.).

### Retrotransposition assays

#### The mneoI-based L1 retrotransposition assay

The cultured cell retrotransposition assay was conducted as previously described [31,61,126]. Briefly, 2×10^3^ HeLa-JVM cells/well were plated in 6-well tissue culture dishes (BD Biosciences). Approximately 24 hours post-plating, transfections were performed using a mixture containing 100 μl Opti-MEM (Life Technologies), 3 μL FuGENE6 (Promega) transfection reagent, and 1 μg L1 plasmid DNA per well of a 6-well plate. Approximately 24 hours post-transfection, the media was replaced with DMEM-complete medium to stop the transfection. Three days post-transfection, the tissue culture medium was replaced and the cells were grown in DMEM-complete medium supplemented with 400 μg/mL of G418 (Life Technologies) to select for retrotransposition events. After approximately 12 days of G418 selection, the resultant G418-resistant foci were washed with ice cold 1X PBS, fixed to the tissue culture plate by treating them for 10 minutes at room temperature in a 1X PBS solution containing 2% paraformaldehyde (Sigma Aldrich) and 0.4% glutaraldehyde (Sigma Aldrich), and stained with a 0.1% crystal violet solution for 30 minutes at room temperature to visualize the G418-resistant foci. As a transfection control, parallel 6-well tissue culture dishes of HeLa-JVM cells were co-transfected with 0.5 μg of an L1 expression plasmid and 0.5 μg of a pCEP/GFP expression plasmid (Stratagene). Three days post-transfection, the transfected HeLa-JVM cells were subjected to fluorescence detection on an Accuri C6 Flow Cytometer (BD Biosciences) to determine the transfection efficiencies (i.e., the percentage of GFP-positive cells) for each experiment [126].

#### The *Trans*-complementation retrotransposition assay

The *Trans*-complementation retrotransposition assay was performed as previously described with minor modifications [50]. Briefly, 2×10^5^ HeLa-JVM cells were plated into 60-mm dishes (BD Biosciences). Approximately 24 hours post-plating, transfections were performed using a mixture containing 93 μl of Opti-MEM (Life Technologies), 6 μl of FuGeneHD (Promega), and 2 μg plasmid DNA (i.e., 1 μg of the L1 “reporter” plasmid and 1 μg of the L1 “driver” plasmid). Subsequent steps of the retrotransposition assay were carried out as described above. As a transfection control, parallel 60-mm tissue culture dishes of HeLa-JVM cells were co-transfected with 0.5 μg of an L1 “reporter” plasmid, 0.5 μg of an L1 “driver” plasmid, and 1 μg of a pCEP/GFP expression plasmid (Stratagene). Three days post-transfection, the transfected HeLa-JVM cells were subjected to fluorescence detection on an Accuri C6 Flow Cytometer (BD Biosciences) to determine the transfection efficiencies (i.e., the percentage of GFP-positive cells) for each experiment [126]. The transfection efficiencies were used to normalize the retrotransposition efficiencies in individual transfections. At least three biological replicates were performed for each retrotransposition assay. Error bars on all retrotransposition assays represent standard deviation of technical triplicates from the indicated experiment.

#### The mEGFPI-based retrotransposition assay

EGFP retrotransposition assays were carried out as previously described [124]. Briefly, HeLa JVM cells were seeded in 6-well plates at a density of 2×10^5^ cells per well. The next day, cells were transfected with 1 μg of retrotransposition reporter plasmid using 4 μL of Fugene 6 transfection reagent. Approximately 48 hours after transfection, culture media was supplemented with puromycin (5 μg/mL) to select for transfected cells. After 4 days of puromycin selection (about 6 days post-transfection), the cells were harvested using trypsin and resuspended in PBS. The percentage of GFP positive cells was determined by flow cytometry using an Accuri C6 flow cytometer (BD Biosciences). Three wells were transfected for each plasmid (three technical replicates) and 10,000 events were analyzed from each well of transfected cells. Live gating was set using forward scatter versus side scatter profile. Events that fell within the live gate were analyzed for fluorescence. Cells transfected with the retrotransposition-dead mutant pL1_RP_(JM111)-EGFP were used as a negative control to set GFP gating.

## Supporting information

S1 Fig SpIREs in the HGR sequence. Data underlying Fig 1. (A) SpIREs present in the HGR. The class of SpIRE (SpIRE_97/622,_ SpIRE_97/790,_ and SpIRE_97/976_) is indicated at the top of each table. Column 1 indicates the L1 subfamily. Column 2 indicates the number of SpIREs present in the subfamily. Column 3 indicates the number of full-length L1s in each subfamily. Column 4 indicates SpIREs as a percentage (%) of full-length L1s. (B) Evolutionary conservation of L1 splice sites. The panels show the conservation of the SD site in the L1 5′UTR (panel 1, SD, red box) as well as SA sites in the L1 5′UTR (panel 2 and 3, SA, green box) and ORF1 (panel 4, SA, green box). Consensus sequences and alignment of those sequences that span the L1PA1–L1PA16 subfamilies were downloaded [84] and manually inspected to determine conservation of splicing sequences. HGR, human genome reference; L1, Long interspersed element-1; ORF, open reading frame; SA, splice acceptor; SD, splice donor; SpIRE, spliced integrated retrotransposed element; UTR, untranslated region.
(TIF)

S2 Fig Intra-5′UTR splicing reduces L1 promoter activity. Data underlying Fig 2. (A) A longer exposure of the northern blots depicted in Fig 2B. The black arrowhead indicates the predicted size of full-length L1/luciferase mRNA (about 2.7 kb). The purple arrowhead indicates the predicted size of pPL_97/622_/L1.3 mRNA visible in the second lane of the first blot (about 2.2 kb). The orange arrowhead indicates an mRNA that may be initiated downstream of the canonical transcriptional start site within the L1 5′UTR that is detected with the luciferase probe (about 2.0 kb). Construct names are indicated above the gel lanes; UTF = untransfected HeLa-JVM cells. The probe used in the northern blot experiment is indicated below the autoradiograph. Molecular weight standards using Millenium RNA Markers (kb) are indicated to the left of the autoradiograph panels. (B) Schematic of oligonucleotides use in RT-PCR experiments. The relative positions of the oligonucleotide primers used to reverse transcribe (REV-LUC, purple line) and then amplify (FWD-5′UTR, red line, and REV-LUC) the L1/firefly luciferase cDNA products. (C) Results from control RT-PCR experiments. A 1.2% agarose gel depicting the results from a representative experiment conducted without the addition of RT. DNA from pPL_97/622_LUC served as a positive control for PCR amplification (yellow #). The final lane contains RT but no mRNA (−mRNA). RT-PCR assays were conducted at least 3 independent times, yielding similar results. L1, Long interspersed element-1; RT-PCR, reverse transcription PCR; UTR, untranslated region.
(TIF)

S3 Fig ORF1p expression from 5′UTR/ORF1 SpIREs. Data underlying Fig 3. (A) Representative ORF1p western blots from RNP fractions. Molecular weight standards (kDa) are indicated to the left of the image. The predicted sizes of full-length ORF1p (black arrowhead), and the N-terminal truncated ORF1p variants (orange and blue arrowheads) are indicated in the image. Construct names are indicated above the image; pCEP/GFP = negative control. The antibodies used in the western blot experiments are indicated to the left (α-N-ORF1p) and right (α-C-ORF1p) of the images. The eIF3 protein (110 kDa) served as lysate loading control. Western blots were performed three times, yielding similar results. (B) Representative western blot from WCL and RNP fractions. Molecular weight standards (kDa) are indicated to the left of the image. Molecular weight standards in the “M” lanes likely cross-reacted with the α-T7 antibody. The predicted sizes of full-length ORF1p (black arrowhead), and the N-terminal truncated ORF1p variants (orange and blue arrowheads) detected by the α-T7 gene10 antibody (α-T7) in WCLs (left) and RNP preparations (right) are highlighted on the gel. Construct names are indicated above the image; pCEP/GFP and untagged pJM101/L1.3 served as negative controls. The eIF3 protein (110 kDa) served as loading controls. The bands at about 30 and 45 kDa are cross-reacting proteins. Western blots were performed three times, yielding similar results. α-C-ORF1p, C-terminal ORF1p antibody; α-N-ORF1p, N-terminal ORF1p antibody; eIF3, eukaryotic initiation factor 3; GFP, green fluorescent protein; ORF, open reading frame; RNP, ribonucleoprotein particle; SpIRE, spliced integrated retrotransposed element; UTR, untranslated region; WCL, whole cell lysate.
(TIF)

S1 Data Sequence information for the 140 SpIREs identified in the HGR sequence. Data underlying Figs 1 and 5. Each SpIRE is annotated to contain the following: (1) a clone number, (2) the L1 subfamily, (3) the class of SpIRE and the designated clone number (e.g., SpIRE_97/622_-6), and (4) a chromosomal location indicating the first and last nucleotide of the designated “filled site” containing the SpIRE and its immediate 5′ and 3′ flaking sequences in the HGR. The designation “empty site” (i.e., pre-integration) site represents the hypothetical reconstructed HGR sequence prior to SpIRE integration. The 5′ plain text/bolded text junction represents the hypothetical position of the putative L1 EN cleavage site on top-strand genomic DNA. The designation “filled site” (i.e., post-integration) represents the SpIRE sequence identified in the HGR. Bolded nucleotides in the “filled site” sequence represent putative TSDs flanking the SpIRE. Dark green shading indicates the first nucleotide of the SpIRE 5′UTR. Red shading indicates the splice junction in the SpIRE. Underscored and italicized nucleotides represent the poly(A) tract at the 3′ end of some SpIREs. Yellow shading indicates possible untemplated or putative transduced sequences before the 5′ or 3′ TSD, respectively. Light blue shading indicates nucleotides in the putative 5′ TSD that differ from nucleotides present in the 3′ TSD. Gray shading indicates possible inversion junctions within the SpIRE sequence. Pink shading indicates additional sequences that interrupt the insertion. EN, endonuclease; HGR, human genome reference; L1, Long interspersed element-1; poly(A), polyadenosine; SpIRE, spliced integrated retrotransposed element; TSD, target site duplication; UTR, untranslated region.
(DOCX)

S1 Table Additional information for each SpIRE. Data underlying Figs 1 and 5. Each tab contains information for a single SpIRE. Column 1 indicates the class of SpIRE and the designated clone number (e.g., SpIRE(_97/622_)-6). Column 2 indicates the L1 subfamily. Column 3 indicates the chromosome. Column 4 indicates the first nucleotide of the “filled site” in the HGR. Column 5 indicates the length (bp). Column 6 indicates whether the insertion resides within a gene and the name of that gene. Column 7 indicates the transcriptional orientation of the SpIRE with respect to the gene transcriptional orientation (same orientation = “Same”; opposite orientation = “Opp.”). Column 8 indicates the calculated L1 EN cleavage sequence of the insertion, where (/) indicates the location of the endonucleolytic nick. Column 9 indicates the putative size of the TSD. Column 10 indicates whether the insertion contains an L1-mediated sequence transduction. Column 11 indicates the length in bp of putative untemplated, mismatched, or 3′ transduced sequences flanking the SpIRE. Column 12 indicates additional major deletions within the SpIRE and indicates the nucleotides that have been lost in reference to L1.3 (accession #L19088). Column 13 provides additional comments about each SpIRE (See Gilbert et al. 2005). EN, endonuclease; HGR, human genome reference; L1, Long interspersed element-1; SpIRE; spliced integrated retrotransposed element; TSD, target site duplication.
(XLSX)

S2 Table Raw luciferase data underlying Fig 2C. See Methods section for complete description of assay conditions. Shown are data from three biological replicates (Assay 1, 2, and, 3) and the combined data (Fig 2C). The assay tabs display raw luciferase readings. In the tabs, the 2nd, 6th, and 10th rows indicate raw firefly luciferase counts (FFLUC Reading) for the indicated firefly luciferase expression construct. The 3rd, 7th, and 11th rows indicate raw Renilla luciferase counts (RENLUC Reading) for the co-transfected internal control Renilla expressing construct (pRL-TK). The 4th, 8th, and 12th rows indicate the ratio of firefly luciferase counts over Renilla luciferase counts (Ratio FF/REN). Columns B–G indicate the raw luciferase counts from six technical replicates. Note that a firefly and Renilla reading were taken for each technical replicate and thus each firefly count is internally controlled with a Renilla count. Column H indicates the average ratio of firefly over Renilla luciferase counts (Avg FF/REN ratio). Column I indicates the fold change of normalized firefly luciferase counts compared to the negative control pGL4.11.
(XLSX)

S3 Table Raw retrotransposition data underlying Fig 4A. See Methods section for complete description of assay conditions. The first column indicates the L1 plasmid (L1). Columns B, C, and D indicate G418 resistant foci from technical triplicates from the indicated experiment (1, 2, 3). Column E indicates the mean number of colonies across technical triplicates (Mean). Column F indicates the standard deviation across technical triplicates (Std. Dev.). Column G indicates the transfection efficiency determined using a transfection control using a pCEP/GFP expression plasmid co-transfected with the L1 expression plasmid (Trans. Eff.). Column H indicates the mean number of colonies normalized to transfection efficiency (Mean Trans. Eff.). Column I indicates the standard deviation across technical triplicates normalized to the transfection efficiency (Std. Dev. Trans. Eff.). Column J indicates the percent retrotransposition activity where the activity of pJM101/L1.3 is set to 100% (% Rtsn.). Note that standard deviations used in the indicated data are derived from the standard deviation normalized to transfection efficiency (column H). GFP, green fluorescent protein; L1, Long interspersed element-1.
(XLSX)

S4 Table Raw retrotransposition data underlying Fig 4B. See Methods section for complete description of assay conditions. The first column indicates the L1 plasmid (L1). Columns B, C, and D indicate G418 resistant foci from technical triplicates from the indicated experiment (1, 2, 3). Column E indicates the mean number of colonies across technical triplicates (Mean). Column F indicates the standard deviation across technical triplicates (Std. Dev.). Column G indicates the transfection efficiency determined using a transfection control using a pCEP/GFP expression plasmid co-transfected with the L1 expression plasmid (Trans. Eff.). Column H indicates the mean number of colonies normalized to transfection efficiency (Mean Trans. Eff.). Column I indicates the standard deviation across technical triplicates normalized to the transfection efficiency (Std. Dev. Trans. Eff.). Column J indicates the percent retrotransposition activity when the activity of pJM101/L1.3 is set to 100% (% Rtsn.). Note that standard deviations used in the indicated data are derived from the standard deviation normalized to transfection efficiency (column H). GFP, green fluorescent protein; L1, Long interspersed element-1.
(XLSX)

S5 Table Raw *Trans*-complementation data underlying Fig 4C. See Methods section for complete description of assay conditions. The first column indicates the L1 reporter plasmid (pPL97-976/L1.3) and the co-transfected L1 driver plasmid (L1). Columns B, C, and D indicate G418 resistant foci from technical triplicates from the indicated experiment (1, 2, 3). Column E indicates the mean number of colonies across technical triplicates (Mean). Column F indicates the standard deviation across technical triplicates (Std. Dev.). Column G indicates the transfection efficiency determined using a transfection control using a pCEP/GFP expression plasmid co-transfected with the L1 expression plasmid (Trans. Eff.). Column H indicates the mean number of colonies normalized to transfection efficiency (Mean Trans. Eff.). Column I indicates the standard deviation across technical triplicates normalized to the transfection efficiency (Std. Dev. Trans. Eff.). Column J indicates the percent retrotransposition activity when the activity of pPL97-976/L1.3 co-transfected with pJBM561 is set to 100% (% Rtsn.). Note that standard deviations used in the indicated data are derived from the standard deviation normalized to transfection efficiency (column H). GFP, green fluorescent protein; L1, Long interspersed element-1.
(XLSX)

S6 Table Raw luciferase data underlying Fig 5B. See Methods section for complete description of assay conditions. Data from three biological replicates (Assays 1, 2, and 3) and the combined data (Fig 5B) are shown. The assay tabs display raw luciferase readings. In the tabs, the 2nd, 6th, 10th, and 14th rows indicate raw firefly luciferase counts (FFLUC Reading) for the indicated firefly luciferase expression construct. The 3rd, 7th, 11th, and 15th rows indicate raw Renilla luciferase counts (RENLUC Reading) for the co-transfected internal control Renilla expressing construct (pRL-TK). The 4th, 8th, 12th, and 16th rows indicate the ratio of firefly luciferase counts over Renilla luciferase counts (Ratio FF/REN). Columns B–G indicate the raw luciferase counts from six technical replicates. Note that a firefly and Renilla reading were taken for each technical replicate and thus each firefly count is internally controlled with a Renilla count. Column H indicates the average ratio of firefly over Renilla luciferase counts (Avg FF/REN ratio). Column I indicates the fold change of normalized firefly luciferase counts compared to the negative control pGL4.11.
(XLSX)

S7 Table Raw retrotransposition data underlying Fig 5C. See Methods section for complete description of assay conditions. The first column indicates the L1 plasmid (L1). Columns B, C, and D indicate EGFP positive cells, as determined using flow cytometry (1, 2, 3). Column E indicates the mean number of EGFP positive cells across technical triplicates (Mean). Column F indicates the standard deviation across technical triplicates (Std. Dev.). Column G indicates the percent retrotransposition activity when the activity of pL1_RP_-EGFP is set to 100% (% Rtsn.). Column H indicates the standard deviation as a percentage of the retrotransposition efficiency (Std. Dev. % Rtsn). L1, Long interspersed element-1.
(XLSX)

## Abbreviations

AUG: translation initiation codon
BLAT: BLAST-like alignment tool
bp: base pair
C: cysteine-rich
CC: coiled-coil
CMV: cytomegalovirus
CTD: C-terminal domain
DMEM: Dulbecco’s Modified Eagle Medium
DTT: dithiothreitol
eIF3: eukaryotic initiation factor 3
EN: endonuclease
FBS: fetal bovine calf serum
HGR: human genome reference
hrGFP: humanized Renilla green fluorescent protein
kb: kilobase
kDa: kilodalton
L1: Long interspersed element-1
MY: million years
MYA: million years ago
NLR: normalized luminescence reading
non-LTR: non-long terminal repeat
ORF: open reading frame
ORF0: antisense open reading frame 0
PBS: phosphate buffered saline
poly(A): polyadenosine
RC-L1: retrotransposition-competent L1
RNP: ribonucleoprotein particle
RRM: RNA recognition motif
RT: reverse transcriptase
RT-PCR: reverse transcription PCR
RUNX3: runt related transcription factor 3
SA: splice acceptor
SD: splice donor
SDS: sodium dodecyl sulfate
SINE: short interspersed element
snRNA: small nuclear RNA
SP1: specificity protein 1
SpIRE: spliced integrated retrotransposed element
SRY: sex determining region Y
SVA: SINE-R/VNTR/Alu
TPRT: target-site primed reverse transcription
UTR: untranslated region
WCL: whole cell lysate
WT: wild-type
YY1: yin and yang 1
ZNF93: Zinc-Finger Protein 93
α-C-ORF1p: C-terminal ORF1p antibody
α-elF3: eukaryotic initiation factor 3 antibody
α-N-ORF1p: N-terminal ORF1p antibody
