# Supplementary figures and images for: Spliced integrated retrotransposed element (SpIRE) formation in the human genome
Source: PLoS Biol. 2018 Mar 5;16(3):e2003067. doi: 10.1371/journal.pbio.2003067 (PMC5860796; doi:10.1371/journal.pbio.2003067)

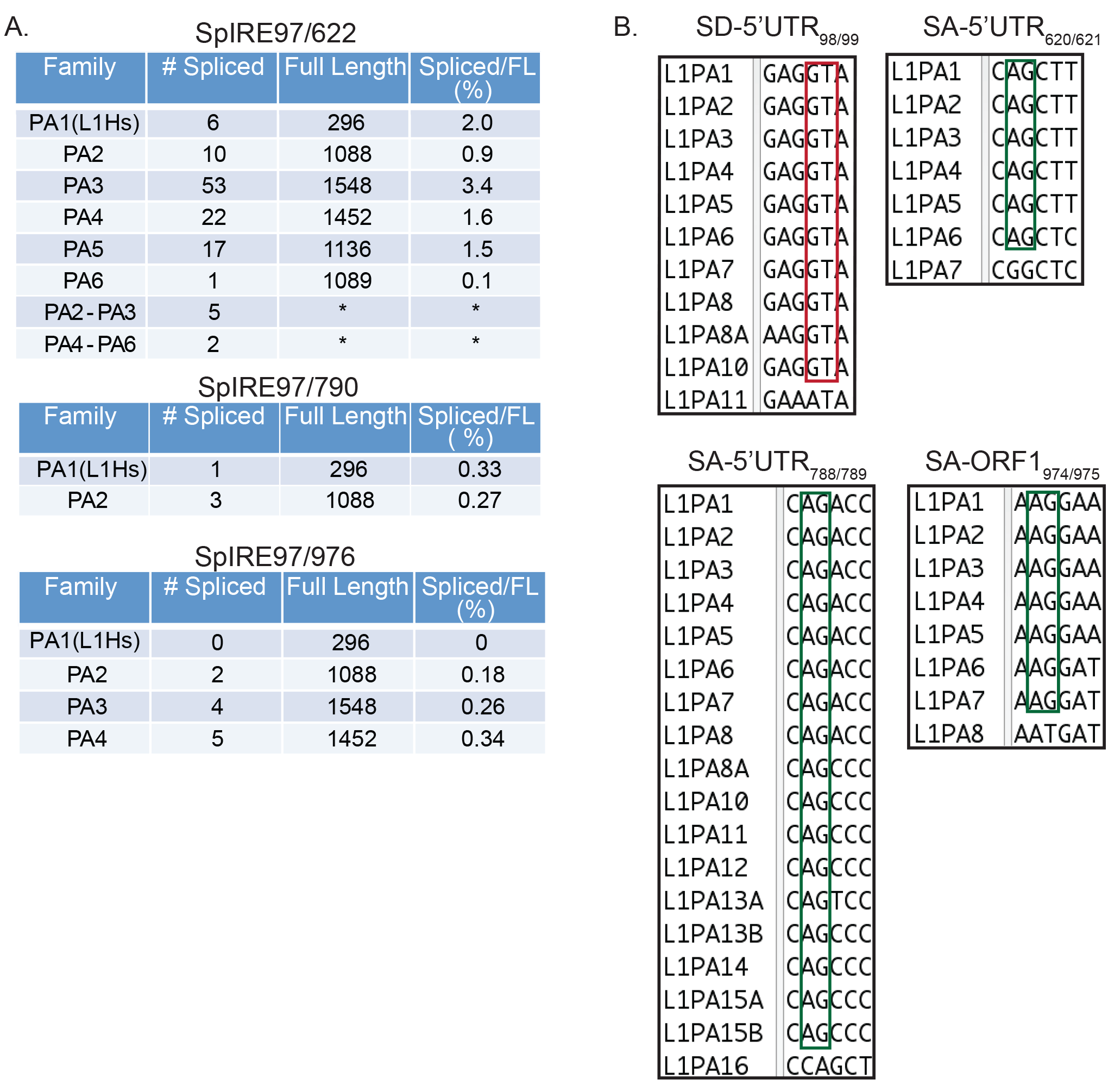

Supplement: S1 Fig — (A) SpIREs present in the HGR. The class of SpIRE (SpIRE97/622, SpIRE97/790, and SpIRE97/976) is indicated at the top of each table. Column 1 indicates the L1 subfamily. Column 2 indicates the number of SpIREs present in the subfamily. Column 3 indicates the number of full-length L1s in each subfamily. Column 4 indicates SpIREs as a percentage (%) of full-length L1s. (B) Evolutionary conservation of L1 splice sites. The panels show the conservation of the SD site in the L1 5′UTR (panel 1, SD, red box) as well as SA sites in the L1 5′UTR (panel 2 and 3, SA, green box) and ORF1 (panel 4, SA, green box). Consensus sequences and alignment of those sequences that span the L1PA1–L1PA16 subfamilies were downloaded [84] and manually inspected to determine conservation of splicing sequences. HGR, human genome reference; L1, Long interspersed element-1; ORF, open reading frame; SA, splice acceptor; SD, splice donor; SpIRE, spliced integrated retrotransposed element; UTR, untranslated region. (TIF) [file pbio.2003067.s001.tif]

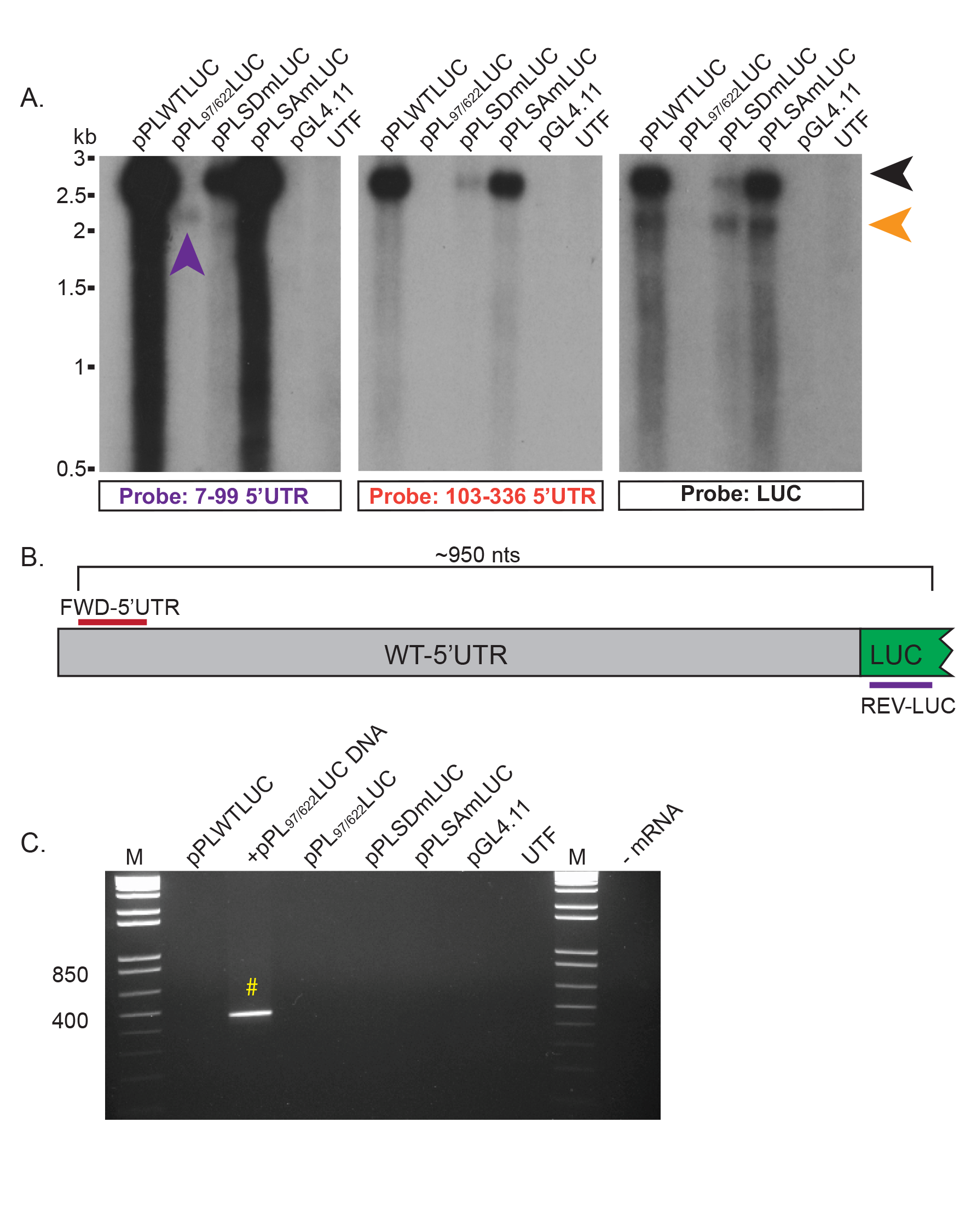

Supplement: S2 Fig — (A) A longer exposure of the northern blots depicted in Fig 2B. The black arrowhead indicates the predicted size of full-length L1/luciferase mRNA (about 2.7 kb). The purple arrowhead indicates the predicted size of pPL97/622/L1.3 mRNA visible in the second lane of the first blot (about 2.2 kb). The orange arrowhead indicates an mRNA that may be initiated downstream of the canonical transcriptional start site within the L1 5′UTR that is detected with the luciferase probe (about 2.0 kb). Construct names are indicated above the gel lanes; UTF = untransfected HeLa-JVM cells. The probe used in the northern blot experiment is indicated below the autoradiograph. Molecular weight standards using Millenium RNA Markers (kb) are indicated to the left of the autoradiograph panels. (B) Schematic of oligonucleotides use in RT-PCR experiments. The relative positions of the oligonucleotide primers used to reverse transcribe (REV-LUC, purple line) and then amplify (FWD-5′UTR, red line, and REV-LUC) the L1/firefly luciferase cDNA products. (C) Results from control RT-PCR experiments. A 1.2% agarose gel depicting the results from a representative experiment conducted without the addition of RT. DNA from pPL97/622LUC served as a positive control for PCR amplification (yellow #). The final lane contains RT but no mRNA (−mRNA). RT-PCR assays were conducted at least 3 independent times, yielding similar results. L1, Long interspersed element-1; RT-PCR, reverse transcription PCR; UTR, untranslated region. (TIF) [file pbio.2003067.s002.tif]

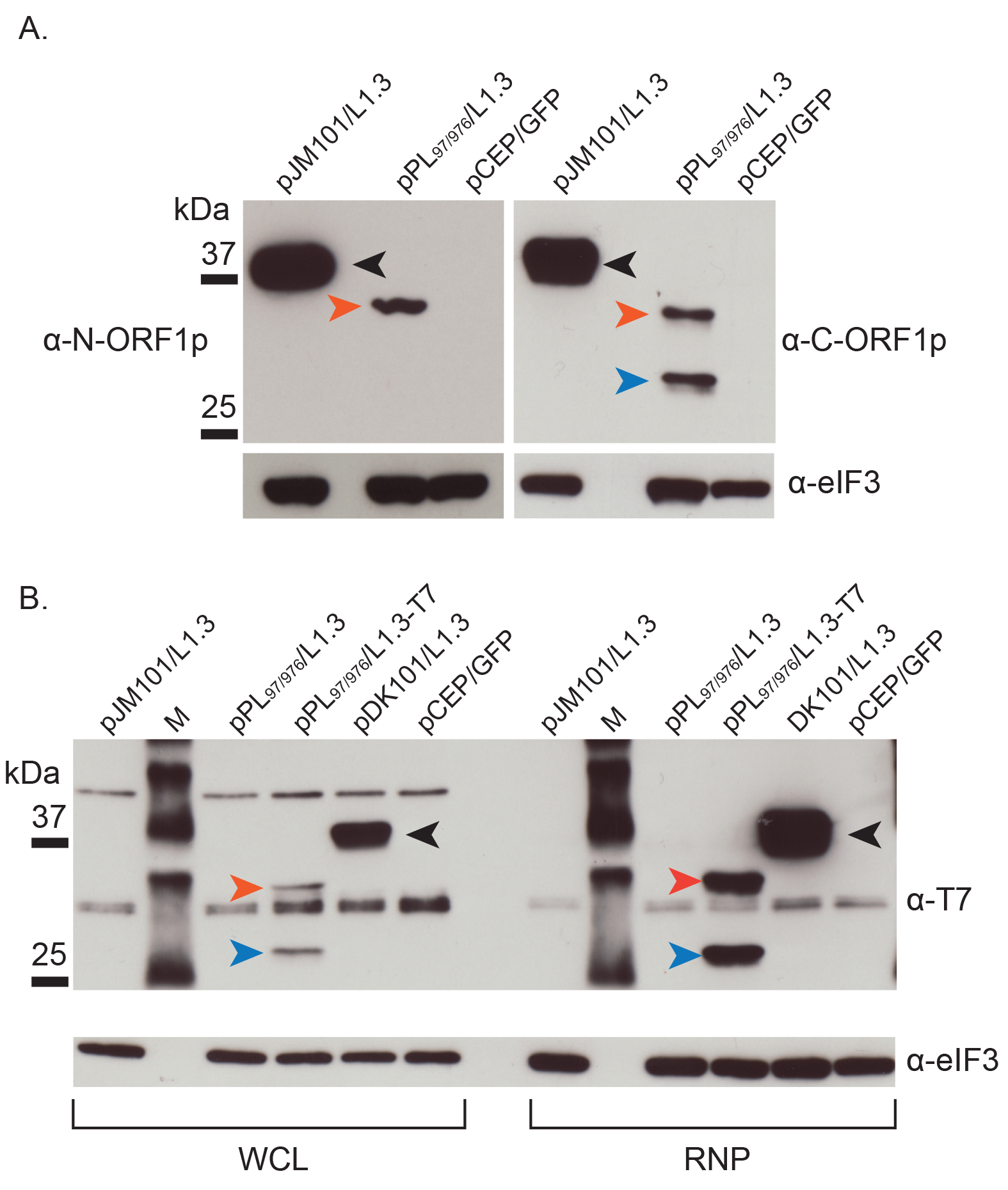

Supplement: S3 Fig — (A) Representative ORF1p western blots from RNP fractions. Molecular weight standards (kDa) are indicated to the left of the image. The predicted sizes of full-length ORF1p (black arrowhead), and the N-terminal truncated ORF1p variants (orange and blue arrowheads) are indicated in the image. Construct names are indicated above the image; pCEP/GFP = negative control. The antibodies used in the western blot experiments are indicated to the left (α-N-ORF1p) and right (α-C-ORF1p) of the images. The eIF3 protein (110 kDa) served as lysate loading control. Western blots were performed three times, yielding similar results. (B) Representative western blot from WCL and RNP fractions. Molecular weight standards (kDa) are indicated to the left of the image. Molecular weight standards in the “M” lanes likely cross-reacted with the α-T7 antibody. The predicted sizes of full-length ORF1p (black arrowhead), and the N-terminal truncated ORF1p variants (orange and blue arrowheads) detected by the α-T7 gene10 antibody (α-T7) in WCLs (left) and RNP preparations (right) are highlighted on the gel. Construct names are indicated above the image; pCEP/GFP and untagged pJM101/L1.3 served as negative controls. The eIF3 protein (110 kDa) served as loading controls. The bands at about 30 and 45 kDa are cross-reacting proteins. Western blots were performed three times, yielding similar results. α-C-ORF1p, C-terminal ORF1p antibody; α-N-ORF1p, N-terminal ORF1p antibody; eIF3, eukaryotic initiation factor 3; GFP, green fluorescent protein; ORF, open reading frame; RNP, ribonucleoprotein particle; SpIRE, spliced integrated retrotransposed element; UTR, untranslated region; WCL, whole cell lysate. (TIF) [file pbio.2003067.s003.tif]
