# Supplementary material for: Spliced integrated retrotransposed element (SpIRE) formation in the human genome
Source: PLoS Biol. 2018 Mar 5;16(3):e2003067. doi: 10.1371/journal.pbio.2003067 (PMC5860796; doi:10.1371/journal.pbio.2003067)
Supplement: S1 Data — Each SpIRE is annotated to contain the following: (1) a clone number, (2) the L1 subfamily, (3) the class of SpIRE and the designated clone number (e.g., SpIRE97/622-6), and (4) a chromosomal location indicating the first and last nucleotide of the designated “filled site” containing the SpIRE and its immediate 5′ and 3′ flaking sequences in the HGR. The designation “empty site” (i.e., pre-integration) site represents the hypothetical reconstructed HGR sequence prior to SpIRE integration. The 5′ plain text/bolded text junction represents the hypothetical position of the putative L1 EN cleavage site on top-strand genomic DNA. The designation “filled site” (i.e., post-integration) represents the SpIRE sequence identified in the HGR. Bolded nucleotides in the “filled site” sequence represent putative TSDs flanking the SpIRE. Dark green shading indicates the first nucleotide of the SpIRE 5′UTR. Red shading indicates the splice junction in the SpIRE. Underscored and italicized nucleotides represent the poly(A) tract at the 3′ end of some SpIREs. Yellow shading indicates possible untemplated or putative transduced sequences before the 5′ or 3′ TSD, respectively. Light blue shading indicates nucleotides in the putative 5′ TSD that differ from nucleotides present in the 3′ TSD. Gray shading indicates possible inversion junctions within the SpIRE sequence. Pink shading indicates additional sequences that interrupt the insertion. EN, endonuclease; HGR, human genome reference; L1, Long interspersed element-1; poly(A), polyadenosine; SpIRE, spliced integrated retrotransposed element; TSD, target site duplication; UTR, untranslated region. (DOCX) [file pbio.2003067.s004.docx]

**­Larson et al., 2017 Supplemental Dataset 1: Annotated SpIRE sequences**

**Notes:**

--Top: SpIRE name and assigned L1 subfamily

--Empty Site: Putative reconstructed L1 empty site from sequence data

--Filled Site: Sequence of SpIRE as determined using BLAT

--All sites mapped onto GRCh38/hg38

--Red Shading: SpIRE splice junction sequence

--Dark Green Shading: First nucleotide of L1

--**Bold Shading**: Putative Target Site Duplication (TSD)

--Yellow Shading: Putative untemplated nucleotides (at 5’ genomic DNA/L1 junction or putative 3’ transductions/extra nucleotides at putative L1/genomic DNA junction)

--Light Blue Shading: Putative mismatches in TSDs

--Gray Shading: Putative inversion breakpoint

--Pink Shading: Sequence interrupting insertion

--*Underline Italicize*: putative poly(A) tail in some insertions (note some are interrupted.

--Dots (.) represent putative deletions

**Please note: as older L1s are fixed in the population, we had to reconstruct the majority of Empty sites. In many cases, mutation and the degeneration of poly(A) sequences at the 3’ end of L1s made it difficult to confidently reconstruct the empty site and determine the precise L1 endonuclease cleavage position. Please see Supplemental Table 1 for additional discussion.

**SpIRE(97/622) sequences**

**Clone 9; L1Hs; SpIRE(97/622)-9:**

**Filled site spans chr1:103921941-103925794**

Empty Site:

TGGAATAATTTTAGAATTGGTACCAGCTCTTCTTTGTGCATCTGGTAGAATTTGGCTGTGAATCTATCTGCTCTGGGGCTTTTTTAGTTGGTAGTTCTTTTTTTAAAAAAATAGTAATTA

AATTTTGAAATTC**AATACTT**TACTCAGAGTGGGCTTGAAA

GTGTTCTCTCCTCTTCAAATTTTCAGAAAAGTTTGAGGAGGATGGATGTTAATTCTTCTTTAAATGTTTGAGAAAAGTTCCCTGTTAAGCTATCTAGTCCTGGGCTTTACTTTGCTGGGA

Filled Site:

TTTTTAGTTGGTAGTTCTTTTTTTAAAAAAATAGTAATTAAATTTTGAAATTC**AATACTT**GGGGGAGGAGCCAAGATGGCCGAATAGGAGCAGCTCCGGTCTACAGCTCCCAGCGTGAGCGACACAGAAGACAGGTGATTTCTGCATTTCCATCTGAGCTTTGAAGAGAGCAGTGGTTCTCCCAGTACGCAGCTGGAGATCTGAGAATGGGCAGACTGCCTCCTCAAGTGGGTCCCTGACCCCTGACCCTGGAGCAGCCTAACTGGGAGGCACCCCCCAGCAGGGGCAGACTGACACCTCACACAGCCAGGTACTCCAACAGACCTGCAGCTGAGGGTCCTGTCTGTTAGAAGGAAAACTAACAAACATAAAGGACATCCACACCAAAAACCCATCTGTACATCACCATCATCAAAGACCAAAAGTAGATAAAACCACAAAGATGGGGAAAAAACAGAGCAGAAAAACTGGAAACTCTAAAAAAGCAGAGCGCCTCTCCTCCTCCAAAGGAACGCAGGTCCTCACCAGCAACGGAACAAAGCTGGATGGAGAATGACTTTGACGAGCTGAGAGAAGAAGGCTTCAGACGATCAAATTACTCCGAGCTACAGGAGGACATTCAAACCAAAGGCAAAGAAGTTGAAAACTTTGAAAAAAATTTAGAAGAACGTATAACTAGAATAACCAATACAGAGAAGTGCTTAAAGGAGTTGATGGAGCTGAAAACCAAGGCTCGAGAACTACGTGAAGAATGCAGAAGCCTCAGGAGCCGATGCGATCAACTGGAAGAAAGGGTATCAGCGATGGAAGATGAAATGAATGAAATGAAGCGAGAAGGGAAGTTTAGAGAAAAAAGAATAAAAAGAAACGAGCAAAGCCTCCAAGAAATATGGGACTATGTGAAAAGACCAAATCTACATCTGATTGGTGTACCTGAAAGTGATGGGGAGAATGGAACCAAGTTGGAAAACACTCTGCAGGATATTATCCAGGAGAACTTCCCCAATCTAGCAAGGCAGGCCAACATTCAGATTCAGGAAATACAGAGAACGCCACAAAGATACTCCTCGAGAAGAGCAACTCCAAGACACATAATTGTCAGATTCACCAAAGTTGAAATGAAGGAAAAAATGTTAAGGGCAGCCAGAGAGAAAGGCCGGGTTACCCTCAGAGGGAAGCCCATCAGACTAACAGCGGATCTCTTGGCAGAAACTCTACAAGCCAGAAGAGAGTGGGGGCCAATATTCAACATTCTTAAAGAAAAGAATTGTCAACCTAGAATTTCATATCCAGCCAAACTAAGCTTCATAAATGAAGGAGAAATAAAATCCTTTACAGACAAGCAAATGCTGAGAGATTTTGTCACCACCAGGCCTGCCCTAAAAGAGCTCCTGAAGGAAGCGCTAAACATGGCAAGGAACAACCGGTACCAGCCACTGAAAAATCATGCCAAAATGTAAAGACCTTTGAGACTAGGAAGAAACTGCATCAACTAATGAGCAAAATAACCAGCTAACATCATAATGACAGGATCAAATTCACACATAACAATATTAACTTTAAATGTAAATGGACTAAATGCTCCAATTAAAAGACACAGGCTGGCAAATTGGATAAAGATACAAGACCCATCAGTGGGCTGTATTCAGGAAACCCATCTCACATGCAGAGACACACATAGGCTCAAAATAAAAGGAGGGAGGAAGATCTACCAAGCAAATGGAAAACAAAAAAAGGCAGGGGTTGCAATCCTAGTCTCTGATAAAACAGACTTTAAACCAACAAAGATCAAAAGAGACAAAGAAGGCCATTACATAATGGTAAAGGGATCAATTCAACAAGAAGAGCTAACTATCCTAAATATATATGCACCCAATACAGGAGCACCCAGATTCATAAAGCAAGTCCTGAGTGACCTACAAAGAGACTTAGACTCCCACACATTAATAATGGGAGACTTTAACACCCCACTGTCAACATTAGACAGATCAACGAGGCAGAAAGTCAACAAGGATACCCAGGAATTGAACTCAGCTCTGCACCAAGCGGACCTAATAGACATCTACAGAACTCTCCACCCCAAATCAATAGAATATACACTTTTTCAGCACCACACCACACCTATTCCAAAATTGACCACACAGTTGGAAGTAAAGCTCTCCTCAGCAAATGTAAAAGAACAGAAATTATAACAAACTATCTCTCAGACCACAGTGCAATCAAACTAGAACTCAGGATTAAGAATCTCACTCAAAACTGCTCAACTACATGGAAACTGAACAACCTGCTCCTGAATGACTACTGGGTACATAACGAAATGAAGGCAGAAATAAAGATGTTCTTTGAAACCAATGAGAACAAAGACACAACATACCAGAATCTCTGGGACGCATTCAAAGCAGGGTGTAGAGGGAAATTTATAGCACTAAATGCCCACAAGAGAAAGCAGGAAAGATCCAAAATTGACACCCTAACATCACAATTAAAAGAACTAGAAAAGCAAGAGCAAACACATTCAAAAGCTAGCAGAAGGCAAGAAATAACTAAAATCAGAGCAGAACTGAAGGAAATAGAGACACAAAAAACCCTTCAAAAAATTAATGAATCCAGGAGCTGGTTTTTTGAAAGGATCAACAAAATTGATAGACTGCTAGCAAGACTAGTAAAGAAGAAAAGAGAGACGAATCAAATAGACGCAATAAAAAAAGATAAAGGGGATATCACCACCAATCCCACAGAAATACAAACTACCATCAGAGAATACTACAAACACCTCTACGCAAATAAACTAGAAAATCTAGAAGAAATGGATAAATTCCTTGACACATACACCCTCCCAAGACTAAACTAGGAAGAAGCTGAATCTCTGAATAGACCAATAACAGGCTCTGAAATTGTGGCAATAATCAATAGCTTACCAACCAAAAAGAGTCCAGGACCAGAAGGATTCACAGCCGAATTCTACCAGAGGTACAAGGAGGAACTGGTACCATTCCTTCTGAAACTATTCCAATCAATAGAAAAAGAGGGAATCCTCCCTAACTCATTTTATGAGGCCAGCATCATCCTGATACCAAAGCCTGGCAGAGACACAACCAAAAAAGAGAATTTTAGACCAATATCCTTGATGAACATTGATGCAAAAATCCTCAATAAAATACTGGCAAAGCAAATCCAGCAGCACATCAAAAAGCTTATCCACCATGATCAAGTGGGTTTCATCCCTGGGATGCAAGGCTGGTTCAGTATATGCAAATCAATAAATGTAATCCAGCATACAAACAGAACCAAAGACAAAAACCATATGACTATCTCAATAGATGCAGAAAAGGCCTTCAACAAAATTCAACAACCCTTCATGCTAAAAACTCTCAATAAATTAGGTATTGATGGGACGTATCTCAAAATAATAAGAGCTATCTATGACAAACCCACAGCCAATATCATACTGAATGGGC

TTTCAGTTTTTCACCATTGTGTATGAGGTTAGCTGGGTTTTTCATACATGGCCTTTATTATGTTGAAGTGATTTCCTTCTATTCCTAGTTGGTTGATTGGTCACGTAGTAGTGTTGAATCTTATCATCTTGTCAATTTTTTTGCATCAATTGAGATGATTTGTGGTTTAGTTTCCCCTTCACTCTCTTAATGTATTACATTGATTGATTTTTATATATTAAGCCATGCTTACATTCCAGGAATAAATCCCACTGGTAATGGTGTATAATATTTTAAATGAGCTATTGAATTTAATTTGCTAATACATTTTTGGTGAGTAACTCATTTATCATGATATTCATCTACAGATTTATTTTTCTGTATTTTCTTGGTTAGGCTTTGTTATCAGTTT**AATACTT**TACTCAGAGTGGGCTTGAAAGTGTTCTCTCCTCTTCAAATTTTCAGAAAAGTTTGAGGAGGATGGATGTTAATTCTTCTTTAAATGTTTGAGAAAAGTTCCCTGTTAAGCTATCTAGTCCTGGGCTTTACTTTGCTGGGAGGTTGTTGATTATTGATTTAATCTCCTTATTAGTTATAGGTCTATTCAGATATTCTACTTTTTCATGATTCAGTGTTGGTAGGTTTTGCTTTTCTAGGAACATGTCTATTTCATCTAGACTATTAAATTTTTTGGAGTGCAATTTATCTTAGTACTCTTGTAATATTTTTGTATCTTTAGAAATAGTAGTAATGTCCTCTCTTTCATTTCTGATGTTACTTTTTTGAATCTTCTCTTTTTCTTAGTCCATCTAGCTAAAAGTTTGTCAATTTTGTTGAACTTTCCTAAGAACCAACTCTTGCCTTTATTATTTCTATTGTTTTTCTATTATTGATTTTGCTTATCTCTGTTCTAATTTTTATTATTTCCTTTTTGAGCTAGTTTTGTGTTTAGTTTGTTTTTCTATTTCTAGTATGCTAAAGTGTAATGTTAGGCTTATTGATGTGAGATCTTTCCTCCTTTTTAATATAAGCATTTATTTACAACTATAAATTTTTCTCATAACACTGTGTTTGCTGCATCCCGTAAGCTTTGGTATGTTATGCTGTAATTTTCATCTGTTTCGAGATATTCTATAATTACCCTTATGATTTCTTTTTTGATCCACTTGTTGTTTAAGAGCGTGTAGTACAGAATTCTGATAATCCTGGCCTTTGAGACGGGGGCTATATTTATTGGTGTCTTCTCTCAACTGACTCAGTCACAGCTAAACAAGTAGTCTTGTCTGAGAAGCCACTGATCTCTGAGGAGACAGATCTTATTGAGCTAACTCTACTGGAGAGCTCATGTGCCACATTGGTTCTTTGGCCTCTGTGTATCATAAGCCTCCAAATATTTTTGTGGAAGGAAGTCATGGAATCCATTGTAAATAGTTGCCAATTCATCATGGGAGTACTGATTCAGGTGACAGCCCTGTTGGTACCACTGCAACTAACCTGAAACAGCCTCAGGTTATCCCCTCATAAGGTAATCTTCTCAGGGGTCTTTTAAACCTTGACCTCAGTCAACCAGTCAATGTGCCACAGGTGTCCTCCATGCAGATGGGAGCAGTGGATCTCTTGGGAGGAGGATAAGATAGTCTGGTGGGACAATCCTTCATCCTATGATGGGTGTCTGCAACCTTTGTTTCTTCATCTACACCTGCTGTGGTCAGCAGTGGACTGAATGACCTGTTTGAACTCTCCACAGGGATAGGCATGGCACCTGGTGGGTATGTGGCTCCTAAGGCTATTTCTGGCTACCTGCATTACAGGATAAAGGCTTAGAGATTTCAGGAACATTGAGTCACCACCAAGGGCACATCTATATGGAAATGAACTTCACCAATACAGCTCTGCAGCATATGACAGATTTTGTAATCTAGTTTAACAAGAATAGCACTCCTCTGGCCATCCATACACCACTGATGCCAAACCAGAACATTGATGTCTCCCTGCCTCTCAATACTTTGGGCCTAGTCATGAAGATGACTGAATAACCTGC

**Clone 38; L1Hs; SpIRE(97/622)-38: Filled site spans: chr4:166265865-166271695**

Empty Site:

CTATTAATAAGAACTAATTTATTTAAACTGTTTCACATAACATCTTTGGCTGACTTATTCTTTGTAACATGATTTTATAAAATTTACTGGCAATATAAAGGACAAAATCATTTGATTTAATATAAAGTAAAAAACTTGATCCTCCCACCTCAGCCCCCCA

AGTAGCAAGGACTACAGTTAAGA**CTTTATTG**CAGCGCGAT

GCTCCTCTCTGGATATTTGTATGCCAATTTATTTAAGAATTATTGTTAAAATTTTTCATTCTGAAAGAAAAATAATAGCAAAAGAACTTGTCCCTGAGGACATCTAAATTGTAAATGAGC

Filled Site:

TATAAAGTAAAAAACTTGATCCTCCCACCTCAGCCCCCCAAGTAGCAAGGACTACAGTTAAGA**CTTTATTG**CAAGATGGCTGAATAGGAACAGCTGCAGTCTACAGCTCCCAGCATGAGCGACGCAGAAGATGGGTGATTTCTGCATTTCCATCTGAGCTTTGAAGAGAGCAGTGGTTCTCCCAGCATGCAGCTGGATATCTGAGAACGGGCAGACTGCCTCCTCAAGTGGGTCCCTGAACCCTGACCCCCGAGCAGCCTAACTGGGAGGCACCTCCCAGTAGGGGCAGACTGACACCTCACACGGCCCGGTACTCCTCTGAGACAAAAGTTCCAGAGGAACAATCAGACAGCAGCATTCACGGTTCATGAAAATCCACTGCTCTGCAGCCACCGCTGCTGATACCCAGGCAAACAGGGTCTGGAGTGGACCTCTAGCAAACTCCAACAGACCTGCAGCTGAGGGTTATGTCTGTTAGAAGGAAAACTAAGAAACAGAAAGGACATCCACACCAAAAACCCATCTGTACATCACCATCATCAAAGACCAAAAGTAGATAAAACCACAAAGATGGGGAAAAAACAGAGCAGAAAAACTGGAAACTCTAAAAAGCAGAGCGCCTCTCCTCCTCCAAAGGAACGCAGTTCCTCACCAGCAACGGAACAAAGCTGGATGGAGAATGACTTTGACGAATTGAGAGAAGAAGGCTTCAGACGATCAAACTATTCTGAGCTACAGGAGGAAATTCAAACCAAAGGCAAAGAAGTTAAAAACTTTGAAAAAAATTTAGACGAATGTATAACTAGAATAACCAATATAGAGAAGTGCTTAAAGGAGCTGATGGAGCTGAAAGCCAAGGCTCGAGAACTACGTGAAGAATGCAGAAGCCTCAGGAGCCGATGCGATCAACTGGAAGAAAGGGTATCAGTGATGGAAGATGAAATGAATGAAATGAAGTGAGAATGGAAGTTTAGAGAAAAAAGAATAAAAAGAAACAAAGCCTCCAAGAAATATGGGACTATGTGAAAAGACCAAATCTACGTCTGATTGGTGTACCTGAAAGTCACGGAGAGAATGGAACCAAGTTGGAAAACACCCTGCAGGATATTATCCAGGAGAACTTCCCCAATCTAGCAAGGCAGGCCAACATTCAGATTCAGGAAATACAGAGAACGCCACAAAGATACTCCTCGAGAAGAGCAACTCCAAGACACATAATTGTCAGATTCACCAAAGTTGAAATGAAGGAAAAAATGTTAAGGGCAGCCAGAGAGAAAGGTCAGGTTACCCACAAAGGGAAGCCCATCAGACTAACAGCGGATCTCTCGGCAGAAACTCTACAAGCCAGAAGAGAGTGGGGGCCAATATTCAACATTCTTAAAGAAAAGAATTTTCAACTCAGAATTTCATATCCAGCCAAACTAAGCCTCATAAGTGAAGGAGAAATAAAATACTTTACAGACAAGCAAATGCTGAGAGATTTTGTCACCACCAGGCCTGCCCTAAAAGAGCTCCTGAAGGAAGCACTAAACATGGAAAGGAACAACTGGTACCAGCTGCTGCAAAATCATGCCAAATTGTAAAGACCATCGAGACTAGGAAGAAACTGCATCAACTAACGAGCAAAATAACCAGCTAACATCATAATGACAGGATCAAATTCACACATAACAATATTAACTTTAAATGTAAATGGACTAAATGCTCCAATTAAAAGACACAGACTGGCAAATTGGATAAAGAGTCAAGACCCATCAGTGTGCTGTATTCAGGAAACCCATCTCACGTGCAGAGACACACATAGGCTCAAAATCAAAGGATAGAGGAAGATCTACCAAGCAAATGGAAAACAAAAAAAGGCAGGGGTTGCAATCCTAGTCTCTGATAACACAGACTTTAAACCAACAAAGATCAAAAGAGACAAAGAAGGCCATTACATAATGGTAAAGGGATCAATTCAACAAGAAGAGCTAACTATCCTAAATATATATGCACCCGATACAGGAGCACCCAGATTCATAAAGCAAGTCCTGAGTGATCTACAAAGAGACTTACACTCCCACACAATAATAATGGGAGCCTTTAACACCCCACTGTCAACATTAGACAGATCAATGAGACAGAAAGTTAACAAGGATACCCAGGAATTGAACTCAGCTCTGCACCAAGCAGACCTAATAGACATCTACAGAACTCTGCACCCCAAATGAACAGAATATACATTTTTTTCAGCACCACACCACACCTATTCCAAAATTGACCACATACTTGGAAGTAAAGCTCTCCTCAGCAAATGTAAAAGAACAGAAATTACAAAAAACTGTCTCTCAGACCACACTGCAATCAAACTAGAACTCGGGATTAAGAAACTCACTCAAACTGCTCAACTACGTGGAAACTGAACAACCTGCTCCTGAATGACTATTGGGTACATAACGAAATGAAGGCAGAAATAAAGATGTTCTTTGAAACCAACAAGAACAAAGACACAACATACCGGAATCTCTGGGATACATTCAAAGCAGTGTGTAGAGGGAAATTTATAGCACTAAATGCCCACAAGAGAAAGCAGAAAAGATCCAAATTGACACCCTAACATCACAATTAAAAAAACTAGAAAAGCAAGAGCAAACACATTCAAAAGCTAGCAGAAGGCAAGAAATAACCAAAATCAGAGCAGAACTGAAGGAAATAGAGACACAAAAAACCCTTCAAAAAATTAATGAATCCAGGAGCCAGTTTTTTGAAAGGATCAACAAAATTGATAGACAGCTAGCAAGACTAATAAAGAACAAAAGAGAGAACAATCAAATAGACACGAAAAAAAATGATAAAGGGGATATCACCACCGATCCCACAGAAGTACAAACTACCATCAGAGAATACTACAAACACCTCTACACAAATCAACTAGAAAATCTAGAAGAAATGGATAAATTCCTCGACACATACACTCTCCCAAGACTAAACCAGGAAGAAGCTGAATCTCTGAATAGACCAATAACAGGCTCTGAAATTGAGGCAATAATCAATAGCTTACCAACCAAAAAGAGTCCAGGACCAGATGGATTTTCAGCCGAATTCTACCAGAGGTACAAGGAGGAACTGGTACCATTCCTTCTGAAACTATTCCAATCAATAGAAAAATAGGGAATCCTCCCTAACTCATTTTATGAGGCCAGCATCATCCTGATACCAAAGCCGGGCAGAGACACAACCAAAAAAGAGAATTTCAGACCAATATTCTTGATGAACATTGATGCAAAAATCCTCAATAAAATACTGGCAAACTGAATCCAGCAGCACATCAAAAAGCTTATCCACCATGATCAAGTGGGCTTCATCCCTGGGATGCAAGGCTGGTTCAATATACACAAATCAATAAATGTAATCCAGCATATAAACAGAACCAAAGACAAAAACCACATGATTATCTCAATAGATGCAGAAAAGGCCTTTGACAAAATTCAACAACGCTTCATGCTAAAAACTCTTCAATAAATTAGGTATTGATGGGACATATCTCAAAATAATAAGAGCTATCTATGACAAACCCACAGCCAATATCATACTGAATGGGCAAAAACTGGAAGCTTTCCCTTTGAAAACTGGCAGAAGACAGGGATGCCCTCTCTCACCACTCCTATTCAACATAGTGTTGGAAGTTCTGGCCAGGGCATTTAGGCAGGAGATGGAAGTAAAGGGTATTCAATTAGGAAAAGAGAAAGTCAAATTGTCCCTGTTTGCAGATGACATGATTGTATATTTAGAAAACCCCATTGTCTCAGCCCAAAATCTCCTTAAGCTGATAAGCTACTTCAGCAAAGTCTCAGGATACAAAATCAATGTACAAAAATCACAAGCATTCTTATGCACCAATAACAAACAGAGAGCCAAATCATGAGTGAACTCCCATTCACAATTGCTTCAAAGAGAATAAAATACCTAGAAATCCAACTTAGAAGGGATGTGAAGGACGTCTTCAAGGAGAACTACAAACCACTGCTCAATGAAATAGAAGAGGATACAAACAAATGGAAGGACATTCCATGCTCATGGGTAGGAAGCATCAATATCGTGAAAATGGCCATACTGCCCGAGGTAATTTATAGATTCAATGCCATCCCCATCAAGCTACCAATGACTTTCTTCACAGAATTGGAAACAACTATTTTAAAGTTCATATGGAACCAAAAAAGAGCCTGCATCGCCAAGTCAATCTTAAGCCAAAAGAACAAAGCTGGAGGCATCACGCTACCTGACTTCAAACTATGCTGCAAGGCTACAGTAACCTAAACAGCATGCTATTGGTACCAAAACAGAGATATAGATCAATGGAACAGAACAGAGCCCTCAGAAATAACACCGCATATCTACAACTATCTGATCTTTGACAAACCTGAGAAAAACAAGCAATGGGGAAAGGATTCCCTATTTAATAAATGATGCTGGGAAAACTGGCTAGCCATATGTAGAGAGCTGAAACTGGATCCCTTCCTTACACCTTATACAAAAATTAATTCAAGATGGATTGAAGACTTAAATGTTAGACCTAAAACCATATAAAAACCCTAGAAGAAAACCTAGGCATTACCATTCAGGACATAGGCATGGGCAAGGACTTCATGTCTAAAACACCAAAAGCAATGGCAACAAAAGCCAAAATTGACAAATGGGATCTAATTAAACTAAAGACTTCTGCACAGCAAAACAAACTACCATCAGAGTGAACAGGCAACCTACAAAATGGGAGAAAATTTTCACAACCTACTCATCTGACAAACGGCTAATATTCAGAATCTACAATGAACTCAAAGAAATTTACAAGAAAAAATCAAAGAACCCCATCAAAAAGTGGGCGAAGTACATAAACAGACACTTCTCAAAAGAAGACATTTATGCAGCCAAAAAACACATGAAAAAATGCTCATCATCACTGGCCATCAGAGAAATGCAAATCAAAACCACAATGAGATACCATCTCACACCAGTTAGAATGGCAATCATTAAAAAGTCAGGAAACAACAGGTGCTGGAGAGGATGTGGAGTAATAGGAACACTTTTACACTGTTGGTGGGACTGTAAAGTAGTTCAACCATTGTGGAAGTCAGTGTGGCGATTCCTCAGGGATCTAGAACTAGAAATATCATTTGACCCAGCCATCCCATTACTAGGTATATACCCAAAGGACTATAAATCATGCTGCTATGAAGACACATGCACACGTATGTTTATTGCAGGACTATTCACAATATCAAAGACTTGGAACCAACCCAAATGTCCAACAATGATAGACTGGATTAAGAAAATGTGGCACATATACACCATGGAATACTATGCAGCCATAAAAAATGATGAGTTCATGTCCTTTGTAGGGACATGGATGAAATTGGAAATCATCATTCTCAGTAAACTATGGCAAGGAGAAAAAATCAAACACCGCATATTCTCACTCATAGGTGGGAATTGAACAATGAGATCACATGGACACAGGAAGGGGAATATCACACTCTGGGGACTGTGGTGGGGTCGGGGGAGGGGGGAGGGATAGCATTGGGAGATATACCTAATGCTAGATGACACATTAGTGGGTGCAGCGCACCAGCATGGCACCTAATGCTAAGTGACGCATTAATGGGTGCAGCATACCAGCATGGCACATGTATATATATGTAACTAACCTGCTCGTTGTGCACATGTACCCTAAAACTT*AAAGTATAATAATAATAATAATAAAAAATAAAGTAAAAAAGTTTTTAGGAAATA*CTC**CTTTATTG**CAGCGCGATGCTCCTCTCTGGATATTTGTATGCCAATTTATTTAAGAATTATTG

**Clone 52; L1Hs; SpIRE(97/622)-52:**

**5’ half of filled site spans: chr6:27943136-27944149**

**3’ half of filled site spans: chr6:27943102-27945677**

Empty Site:

ATTGAGATACAATTTCTCACTTATTAGACTGGCATTAAAAATATTAGCATGTTCTTTTAGTGAAGCTCTGTGGAAACAGGAGTTCTCATGGCCAGCAAGAGCTGAACTGAGCTGCCAGTT

GTGGGAGAAGTCAAT**TAAAA**GAACATGCTAATATTTTTAA

TGCCAGTCTAATAAGTGAGAAATTGTATCTCAATATTGTTTTAATTTGTGCCTGTCTAATTATACATGCATTTAAATTTTTTCATATGGTGAGAATAATTTTTTGTCTGGTTTGTTGATT

Filled Site:

ATTGAGATACAATTTCTCACTTATTAGACTGGCATTAAAAATATTAGCATGTTCTTTTAGTGAAGCTCTGTGGAAACAGGAGTTCTCATGGCCAGCAAGAGCTGAACTGAGCTGCCAGTTCTCATGGCCAGCAAGAGCTGAACTGAGCTGCCAGTTGTGGGAGAAGTCAAT**TAAAA**CAACAGCCAAGGAGGAGGAGCCAAGATGGCCGAATAGGAACAGCTCGGGTCTACAGCTCCCAGCGTGAGCGACACAGAAGACAGGTGATTTCTGCATTTCCATCTGAGCTTTGAAGAGAGCAGTGGTTCTCCCACCACGCAGCTGGAGATCTGAGAACGGGCAGACTGCCTCCTCAAGTGGTTCCCTGACACCTGACCCCAGAGCAGCCTAACTGGGAGGCACCCCCCCAGCAGGGGCACACTGACACCTCACAAGGCAGGGTATTCCAACAGACCTGCAGCTGAGGGCCCTGTCTCTTAGAAGGAAAACTAACAAACAGAAAGGACATCCACACCAAAAACCCATCTGTACATCACCATCATCAAAGACCAAAAGTAGATAAAACCACAAAGATGGGGAAAAAACAGAACAGAAAAACTGGAAACTCTAAAAAGCAGAGCGCCTCTCCTCCTCCAAAGGAACGCAGTTCCTCACCAGGAACGGAACAAAGCTGGAGGGAGAATGACTTTGACTAGCTGAGAGAAGAAGGCTTCAAACGATCAAATTACTCTGAACTACGGGAGGACATTCAAACCAAAGGCAAAGAAGTTGAAAACTTTGAAAAAAATTTAGAAGAATGTATAACTAGAATAACCAATACAGAGAAGTGCTTAAAGGAGCTGATGGAGCTGAAAACCAAGGCTTGAGAACTACGTGAAGAATGCAGAAGCCTCAGGAGCCGATGCGATCAACTGGAAGAAAGGGTATCAGCAATGGAAGATGAAATGAATGAAATGAAGTGAGAAGGGAAGTTTAGAGAAAAAAGAATGAAAAGAAATGAGCAAAGCCTCCAAGAAATATGGGACTATGTGAAAAGACCAAATCTACGTCTGTGGGATGCAAGGCTGGTTCAATATATGCAAATCAATAAATGTAGTCCAGCATATAAACAGAACCAAAGACAAAAACCACATGATTATCTCAATAGATGCAGAAAAGGCCTTTGACAAAATTCATCAACACTTCATGCTAAAAACTCTCAATAAATTAGGTATTGATGAGACGTATCTCAAAATAATAAGAGCTATCTATGACAAACCCACAGCCAATATCATACTGAATGGGCAAAAACTGGAAGCATTCCCTTTGAAAACTGGCACAAGACAGGGATGCCCTCTCTCACCACTCCTATTCAACATAGTGTTGGAAGTTCTGGCCAGGGCAATTAGGCAGGAGAAGGAAATAAAGAGTATTCAATTAGGAAAAGAGGAAGTCAAATTGTCCCTGTTTGCAGACGACATGATTGTATATCTAGAAAACCCCATTGTCTCAGCCCAAAATCTCCTTAAGCTGATAAGCAACTTCAGCAAAGTCTCAGGATACAAAATCAATGTACAAAAATCACAAGCATTCTTATACACCAATAACAGACAAACAGAGAGCCAAATCATGAGTGAACTCCCATTCACAATTGCTTCAAAGAGAATAAAATACCTAGGAATCCAACTTACAAGGGACGTGAAGGACCTCTTCAAGGAGAACTACAAACCACTGCTCAAGGAAATAAAAGAGGATACAAACAAATGGAAGAACATTCCATGCTCATGGGTAAGAAGAATCAATATTGTGAAAATGGCCATACTGCCCAAGGTAATTTATAGATTCAATGCCATCCCCATCAAGCTACCAATGACTTTCTTCACAGAATTGGAAAAAACTACTTTAAAGTTCTTATGGAACCAAAAAAGAGCCTGCATCGCCAAGTCAATCCTAAGCCAAAATAACAAAGCTGGAGGCATCATGCTACCTGACTTCAAACTATACTACAAGGCTACAGTAACCAAAACAGCATGGTACCGGCACCAAAACGGAGATATAGATCAATGGAACAGAACAGAGCCCTCAGAAATAACGCTGCATAGCTACAACTATCTGATCTTTGACAAACCTGAGAAAAACAAGCAATGGGGAAAGGATGATTCCCTATTTAATAAATGGTGCTGGGAAAACTGGCTAGCCATATGTAGAAAGCTGAAACTGGATCCCTTCCTTACACCTTATACAAAAACTAATTCAAGATGGATTAAAGACTTAAACGTTAGACCTAAAACCATAAAAACCCTAGAAGAAAACCTAGGCATTACCATTCAGGACATAGGCATGGGCAAGGACTTCATGTCTAAAACACCAAAAGCAATGGCAACAAAAGCCAAAATTGACAAATGGGATCTAATTAAACTCAAGAGCTTCTGCACAGCAAAAGAAACTACCATCAGAGTGAACAGGCAACCTACAAAACGGGAGAAAATTTTTGCAACCTACTCATCTGACAAAGGGCTAATATCCAGAATCTACAATGAACTCAAACAAATTTACAAGAAAAAAACAAACAACCCCATCAAAAAGTGGGCAAAGGATATGAACAGACGTAGATTTGGTCTTTTCACATAGTCCCATATTTCTTGGAGGCTTTGCTCATTTCTTTTCATTCTTTTTTCTCTAAACTTCCCTTCTCACTTCATTTCATTCATTTCATCTTCCATTGCTGATACCCTTTCTTCCAGTTGATCGCATCGGCTCCTGAGGCTTCTGCATTCTTCACGTAGTTCTCAAGCCTTGGTTTTCAGCTCCATCAGCTCCTTTAAGCACTTCTCTGTATTGGTTATTCTAGTTATACATTCTTCTAAATTTTTTTCAAAGTTTTCAACTTCTTTGCCTTTGGTTTGAATGTCCTCCCGTAGTTCAGAGTAATTTGATCGTTTGAAGCCTTCTTCTCTCAGCTAGTCAAAGTCATTCTCCCTCCAGCTTTGTTCCGTTCCTGGTGAGGAACTGCGTTCCTTTGGAGGAGGAGAGGCGCTCTGCTTTTTAGAGTTTCCAGTTTTTCTGTTCTGTTTTTTCCCCATCTTTGTGGTTTTATCTACTTTTGGTCTTTGATGATGGTGATGTACAGATGGGTTTTTGGTGTGGATGTCCTTTCTGTTTGTTAGTTTTCCTTCTAAGAGACAGGGCCCTCAGCTGCAGGTCTGTTGGAATACCCTGCCTTGTGAGGTGTCAGTGTGCCCCTGCTGGGGGGGTGCCTCCCAGTTAGGCTGCTCTGGGGTCAGGTGTCAGGGAACCACTTGAGGAGGCAGTCTGCCCGTTCTCAGATCTCCAGCTGCGTGGTGGGAGAACCACTGCTCTCTTCAAAGCTCAGATGGAAATGCAGAAATCACCTGTCTTCTGTGTCGCTCACGCTGGGAGCTGTAGACCCGAGCTGTTCCTATTCGGCCATCTTGGCTCCTCCTCCTTGGCTGTTGTTTTAATTGACTTCTCCCACAACTGGCAGCTCAGTTCAGCTCTTGCTGGCCATGAGAACTCCTGTTTCCACAGAGCTTCAC**TAAAA**GAACATGCTAATATTTTTAATGCCAGTCTAATAAGTGAGAAATTGTATCTCAATATTGTTTTAATTTGTGCCTGTCTAATTATACATG

**Clone 62; L1Hs; SpIRE(97/622)-62: Filled spans: chr7:63060743-63064765**

Filled Site: No TSD Present

GGGAGGAGCCAAGATGGCCGAATAGGAAGAGTTCCAGTCTACAGCTCCCAGCGTGAGCGACGCAGAAGACGGGTGATTTCTGCATTTCCATCTGAGCTTTGAAGAGAGCAGTGGTTCTCCCAGCACGCAGCTGGAGATCTGAGAACGGGCAGACTGCCTCCTCAAGTGGGTCCCTGACCCCTGACCCCTGGCCCCCGAGTAGCCTAACTGGGAGGCACCCCCCAGCAGGGGCAGACTGACACCTCACACGGCCAGGTACTCCAACAGACCTGCAGCTGAGGGTTCTGTCTGTTAGAAGGAAAACTAACAAACAGAAAGGACATCCACACCAAAAACCCATCTGTACATCACCATCATCAAAGACCAAAAGTAGATAAAACCACAAAGATGGGGAAAAAACAGAACAGAAAAACTGGAAACGCTAAAAAGCAGAGCGCCTCTCCTCCTCCAAAGGAATGCAGTTCCTCACCAGCAATGGAACAAAGCTGGACGGAGAATGACTTTGACAAGCTGAGAGAAGAAGGCTTCAGATGATCAAATTACTCCGAGCTATGGGAGGACATTCAAACCAAAGGCAAAGAAGTTGAAAACTTTGAAAAAAATTTAGAAGAATGTATAACTAGAATAACCAATACAGAGAAGTGCTTAAAGGAGCTGATGGAGCTGAAAACCAAGGCTCGAGAACTACGTGAAGAATGCAGAAGCCTCAGAAGCCGATGCGATCAACTGGAAGAAAGGGTATCAGCGATGGAAGATGAAATGAATGAAATGAAGTGAGAAGGGAAGTTTAGACAAAAAAGAATAAAAAGAAATGAACAAAGCCTCCAAGAAATATGGGACTATGTGAAAAGACCAAATCTACGTCTGACTGGTGTACCTGAAAGTGACGGGGAGAATGGAACCAAGTTGGAAAACACTCTGCAGGATATTATCCAGGAGAACTTCCCCAATCTAGCAAGGCAGGCCAACATTCAGATTCAGGAAATACAGAGAATGCCACAAAGATACTCCTCGAGAAGAGCAACTCCAAGACACATAATTGTCAGATTCATCAAAGTTAAAATGAAGGAAAAAATGTTAAGGGCAGCCAGAGAGAAAGGTCGGGTTACCCACAAAGGGAAGCCCATCAGACTAACAGCAGATCTCTCAGCAGAAACTCTACAAGCCAGAAGAGAGTGGGGGCCAATATTCAACATTCTTAAAGAAAAGAATTTTCAACCCAGAATTTCATATCCAGTCAAACTAAGCTTCATAAGTGAAGGAGAAATAAAATACTTTACAGACAAGCCAATGCTGAGAGATTTTGTCACCACCAGGCCTGCCCTAAAAGAGCTCCTGAAGGAAGTGCTAAACATGGAAAGGAACAACTGGTACCAGCCGCTGCAAAATCATGCCAAAATGTAAAGACCATCAAGACTAAGAAGAAACTGCATCAACTAACGAGCAAAATAACCAGCTAACATCATAATGACAGGATCAAATTCACACATAAAAATATTAACTTTAAATGTAAATGGACTAAATGCTCCAATTAAAAGACACAGACTGGCAAATTGGATAAAGATTCAAGACCCATCAGTGTGCTGTATTCAGGAAACCCATCTCACGTGCAGAGACACACATAGGCTCAAGATAAAAGGATGGAGGAAGATCTACCAAGCAAATGGAAAACGAAAAAAGGCAGGGGTTGCAATCCTAGTCTCTGATAAAACAGACTTTAAACCAACAAAGATCAAGAGACAAAGAAGGCCATTACATAATGGTAAAGGGATCAATTCAACAAGAAGAGCTAACTATCCTAAATATATATGCACCCAATACAGGAGCACCCAGATTCATAAAGCAAGTCCTGAGAGACCTACAAAGAGACTTAGACTCCCACACATTAATAATGGGAGACTTTAACACCCCACTATCAACATTAGACACATCAACGAGACAGAAATTCAACAAGGATACCCAGGAATTGAACTCAGCTCTGCACCAAGCGGACCTAACAGACATCTACAGAACTCTCCACCACAAATCAACAGAATATACATTTTTTTCAGCACCACACCACACCTATTCCAAAACTGACCACATACTTGGAAGTAAAGCTCTCCTCAGCAAATGTAAAAGAACAGAAATTATAACAAACTATCTCTCAGACCACAGTGCAATCAAACTAGAACTCAGGATTAAGAATCTCACTCAAAACCGCTCAACTACATGGAAACTGAACAACCTGCTCCTGAATGACTACTGGATACACAACGAAATGAAGGCAGAAATAAAGATGTTCTTTGAAACCAAGGAGAACAAAGACACAACATACCAGAATCTCTGGGACGCATTCAAAGCAGTGTATAGAGGAAATTTATAGCACTAAATGCCCACAAGAGAAAGCAGGAAAGATCCAAAATTGACAACCTAACATCACAATTAAAAGAACTAGAAAAGCAAGAGCAAACACATTCAAAAGCTAGCAGAAGGCAAGAAATAACTAAAATCAGAGCAGAACTGAAGGAAATAGAGACACAAAAAACCCTTCAAAAAATTAATGAATCCAGGAGCTGGTTTTTTGAAAGGATCAACAAAATTGATAGACCACTAGCAAGACTAATAAAGAAAAAAAGAGAGAGGAATCAAATAGATGCAATAAAAAATGATAAAGAGGATATCACCACCGTCCCACAGAAATACAAACTACCATCAGAGAATACTACAAACACCTCTATGCAAATAAACTAGAAAATCTAGAAGAAATGGATAAATTCCTCGACACATACACTCTCCCAAGACTAAACCAGGAAGAAGTTGAATCTCTGAATAGACCAATAACAGGATCTGAAATTGTCACAATAATCAATAGCTTACCAACACAAAAGAGCCCAGGACCAGATGGATTCACAGCCGAATTCTACCAGAGGTACAAGCAGGAACTGGTACCATTCCTTCTGAAACTATTCCAACCAATAGAAAAAGAGGGAATCCTCCCTAACTCATTTTATGAGGCCATCATCATCCTGATACCAAAGCCAGGCAGAGACACAACCAAAAAAGAGAATTTTAGACCAATATCCTTGATGAACATTGATGCAAAAATCCTCAATAAAATACTGGCAAACTGAATCCAGCAGCACATCAAAAAGCTTATCCACCATGATCAAGTGGGCTTCATCCCCGGGATGCAAGGCTGGTTCAATATACGCGAATCAATAAATGTAATCCAGCATATAAACAGAACCAAAGACAAAAACCACATGATTATCTCAATAGATGCAGAAAAGGCCTTTGACAAAATTCAACAACCCTTCATGCTAAAAACTCTCAATAAATTAGGTATTGATGGGACGTATTTCAAAATAATAAGAGCTATCTACGACAAACCCACAGCCAAATCATACTGAGTGGGCAAAAACTGGAAGCATTCCCTTTGAAAACTGGCACAAGACAGGGATGCCCTCTCTCACTACTCTTATTCAACATAGTGTTAGAAGTTCTGGCCAGGGCAATTAGGCAGGAGAAGGAAATAAAGGGTATTCAATTAGGAAAAGAGGAAGTCAAATTGTCCCTGTTTGCAGATGACATGATTGTATATCTAGAAAACCCCATTGTCTCAGCCCAAAATCTCCTTAAGCTGATAAGCAACTTCAGCAAAGTCTCAGGATACAAAATCAATGTACAGAAATCACAAGCATTCTTATACACCAACAACAGACAAACAGAGAGCCAAATCATGAGTGAACTCCCATTCACAATTGCTTCAAAGAGAATAAAATACCTAGGAATCCAACTTACAAGGGATGTGAAGGACCTCTTCAAGGAGAACTACAGACCTGGTCTAATTCTATGATCTTTATCACATGCTGCCAGATGCTGAAGAGAAATAGAAGCAACAGCAATATGGGAGTTACCCTCTCTAAAGCCCAACTGAAAATCCCTGGAGTCAGAAGTTTCACAAACAGAAGAACAACATACTTATCCTGAATCAGGGAAGGAGAAACAATTCAAACATCTAAATTTAAATTGCTATGAGAA

**Clone 90; L1Hs; SpIRE(97/622)-90: Filled site spans: chr12:10344303-10350010**

Empty Site:

AATTGGTATGCTGAAGAGATATCTGAACTCCACGTTCATTGCAACATTAATCACAATAGCCAAGATATGGAATCAGCTTAAGTGTCCATCAACAGATGAATGGATAAAGAAAATATGGTG

TATATATACAG**AAAGAAATACTATACAGC**CTTTAAAAAGA

AGGAACTTCTGTCATTCGTGACAACATAGAAACATGTGGAGGACATTATGCTAACTAAAAGAACCCAGGCACATAAGGAAAAATACTGCATGATCTCATTTATATTTGGAATCTAAAAAG

Filled Site:

TGTCCATCAACAGATGAATGGATAAAGAAAATATGGTGTATATATACAG**AAAGAAATACTATACAGC**AGGAGGAGCCAAGATGGCCGAATAGGAACAGCTCTGGTCTACAGCTCCCAGCGTGAGCGATGCAGAAGACGGGTGATTTCTCCATTTCCATCTGAGCTTTGAAGAGAGCAGTGGTTCTGCCAGCACGCAGCTGGAGATCTGAGAATGGGCAGACTGCCTCCTCAAGTGGGTCCCTGACCCCTGACCCCCGAGCAGCCTAACTGGGAGGCACCCCCCAGCAGGGGCAGACTGACACCTCACACAGCCGGGTACTCCAACAGACTGGCAGCTGAGGGTCCTGTCTGTTAGAAGGAAAACTAACAAACAGAAAGGACATCCACACCAAAAACCCATCTGTACATCACCATCATGAAAGACCAAAAGTAGATAAAACCACAAAGATGGGGAAAAAACAGAGCAGAAAAACTGGAAACTCTAAAAAGCAGAGTGCCTCTCCTCCTCCAAAGGAACGCAGTTCCTCACCAGCAACGGAACAAAGCTGGACGGAGAATGACTTTGACAAGCTGAGAGAAGAAGGCTTCAGACGATCAAATTACCCCGAGCTACGGGAGGACATTCAAACCAAAGGCAAAGAAGTTGAAAACTTTGAAAAAAAATTTAGAAGAATGTATAACTAGAATAACCAATACAGGGAAGTGCTTAAAGGAGCTGATGGAGCTGAAAACCAAGGCTCGATAACTACGTGAAGAATGCAGAAGTCTCAGGAGCCAATGTGATCAACTGGAAGAAAGGGTATCAGTGATGGAAGATGAGATGAATGAAATGAAGCGAGAAGGGAAGTTTAGAGAAAAACGAATAAAAAGAAATGAGCAAAGCCTCCAAGAAATATGGGACTATGTGAAAAGACCAAATCTACGTCTGATTGGTGTACCTGAAAGTGATGGGGAGAATGGAACCAAGTTGGAAAACACTCTGCAGGATATTATCCAGGAGAACTTCCCCAATCTAGCAAGGCAGGCCAACGTTCAGATTCAGGAAATACAGAGAACGCCACAAAGATACTCCTCGAGAAGAGCAACTCCAAGACACATAATTGTCAGATTCACCAAAGTTGAAATGAAGGAAAAAATGTTAAGGGCAGCCAGAGAGAAAGGTCGGGTTACCCTCAAAGGGAAGCCCATCAGACTAACAGTGGATCTCTCGGCAGAAACTCTACAAGCCAGAAGAGAGTGGGGGCCAATATTCGACATTCTTAAAGAAAAGAATTTTCAACCCAGAAATTCATATCCAGCCAAACTAAGCCTCATAAGTGAAGGAGAAATAAAATACTTTACAGAGAAGCAAATGCTGAGAGATTTTGTCACCACCAGGCCTGCCCTAAAAGAGCTCCTGAAGGAAGTGCTAAACATGGAAAGGAACAACCGGTACTAGCCGCTGCAAAATCATGCCAAAATGTAAAGACCTTCGAGACTAGGAAGAAACTGCATCAACTAATGAGCAAAATAACCAGCTAACATCATAATGACAGGATCAAATTCACACATAACAATATTAATTTAAATATAAATGGACTAAATGCTCCAATTAAAAGACACAGACTGGCAAATTGGATAAAGAGTCAAGACCCATCAGTGTGCTGTATTCAGGAAACCCATCTCACATGCAGAGACACACATAGGCTCAAAATAAAATGATGGAGGAAGATCTACCAAGCAAATGGAAAACAAAAAAAGGCAGGGGTTGCAATCCTAGTCTCTGATAAAACAGACTTTAAACCAACAAAGATCAAAAGAGACAAAGAAGGCCATTAAATAATGGTAAAGGGATCAATTCAAGAAGAAGAGCTAACTATCCTAAATATATATGCACCCAATACAGGAGCACCCAGATTCATAAAGCAAGTCCTGAGTGACCTACAAAGAGACTTAGACTCCCACACATTAATAATGGGAGACTTTAACACCCCACTCTCAACATTAGACAGATCAACGAGACAGAAAGTCAACAAGGATACCCAGGAATTGAACTCAGCTCTGCACCAAGCAGACCTAATAGACATCTACAGAACTCTCCACCCCAAATCAACAGAATATACATTTTTTTCAGCACCACACCACACCTATTCCAAAATTGACCACATACTTGGAAGTAAAGCTCTCCTCAGCAAATGTAAAAGAACAGAAATTATAACAAACTATCTGTCAGACCACAGTGCAATCAAACTAGAACTCAGGATTAAGAATCTCACTCAAAACTGCTCAACTACATGGAAACTGAACAACCTGCTCCTGAATGACCACTGGGTACATAACGAAATGAAGGCAGAAATAAAGATGTTCTTTGAAACCAACGAGAACAAAGACACAACATACCAGAATCTCTGGGACGCATTCAAAGCAGTGTGTAGAGGGAAATTTATAGCACTAAATGTCCACAAGAGAAAGCAGGAAAGATCTAAAATCAACACCCTGACATCACAATTAAAAGAACTAGAGAAGCAAGAGCAAACACATTCAAAAGCTAGCGGAAGTCAAGAAGTAACTAAGATCAGAGCAGAACTGAAGGACATAGAGACAGACAAAACCATTCAAAAAAATCAATGAATCCAGGAGCTGGTTTTTCGAAAAAATCAACAAAATTGACAGACCACTAGCAAGACTAATAAAGAAGAAAAGAGAGAAGAATCAAATAGATGTAATAAAAAATGATAAAGGGGATATCACCACTGATCCCACAGAAATACAAACTACCATCAGAGAATACTACAAACACCTCTATGCAAATAAACTAGAAAATCTAGAAGAAATGGATAAATTCCTTGACACATACACTTTCCCAAAACTAAACCAGGAAGAAGTTGAGTCTCTGAATAGACCAATAACAGGATCTGAAATTGTGGCAATAATCAATAGCTTACCAACCAAAAAGAGTCCAGGACCAGATGGATTCACAGCCAAATTCTACCAGAGGTACAAGGAGGAACTGGTACCATTCCTTCTGAAACTATTCCAATCAACAGAAAAAGAGGGAATCCTCCCTAACTCATTTTATGAGGCCAGCAACATCCTGATACCAAACCCGGGCAGAGACACAACCAAAAAAGAGAATTTTAGACCAATATCCTTGATGAACACTGATGCAAAAATCCTCAATAAAATACTGGCAAACTGAATCCAGCAGCACATCAAAAAGCTTATCCACCATGATCAAGTGGGCTTCATCCTTGGGATGCAAGGCTGGTTCAATATACGCAAATCAATAAATGTAATCCAGCATATAAACAGAACCAAAGACAAAAACCACATGATTATCTCAATAAATGCAGAAAAGGCCTTTGACAAAATTCAACAACCCTTAATGCTAAAAACTCTCAATAAATTAGGTATTGATGGGACGTATTTCAAAATAATAAGAGCTATTTATGACAAACCCACAGCCAATATCATGCTGAATGGACAAAAACTGGAAGCATTCCCTTTGAAAACTGGCACAAGACAGGGATGCCCTCTCTCACCACTCCTATTCAACATAGTGTTGGAAGTTCTGGCCAGGGCAATTAGGCAGGAGAAGGAAATAAAGGGTATTCAATTAGGAAAAGAGGAAGTCAAATTGTCCCTCTTTGCAGATGACATGATTGTATATCTAGAAAACCCCATTGTCTCAGCCCAAAATCTCCTTAAGCTGATAAGCAACTTCAGCAAAGTCTCAGGATACAAAATCAATGTACAAAAATCACAAGCATTCTTATACACCAGCAACAGACAAACAGAGAGCCAAATCATGAGTGAACTCCCATTCACAATTGCTTCAAAGAGAATGAAATACCTAGGAATCCAACTTACAAGGGATGTGAAGGACCTCTTCAAGGAGAACTACAAACCACTGCTCAAGGAAATAAAAGAGGATACAAACAAATGGAAGAACATTCCATGCTCATGGGTAGGAAGAATCAATATCGTGAAAATGGCCATACTGCCCAAGGTAATTTACAGATTCAATGCCATCCCCATCAAGCTACCAATGACTTTCTTCACAGAATTGGAAAAAACTACTTTAAAGTTCATATGGAACCAAAAAAGAGCCCGCATCGCCAAGTCAATCCTAAGCCAAAAGAACAAAGCTGGAGGCATCACGCTACCTGACTTCAAACTATACTACAAGGCTACAGTAACCAAAACAGCATGGTACTGGTACCAAAACAGAGATATAGATCAATGGAACAGAACAGAGCCCTCAGAAATAACACCACATATCTACAACTATCTGATCTTTGACAAACCTGAGAAAAACAAGCAATGGGGAAAGGATTCCCTATTTAATAAATGGTGCTGGGAAAAGTGGCTAGCCATATGTAGAAAGCTGAAACTGGATCCCTTCCTTACACCTTATACAGAAATCAATTCAAGACGGATTAAAGACTTAAACGTTAGACCTAAAGCCATAAAAACCCTAGTAGAAAACCTAGGCATTACCATTCAGGACATAGGCATGGGCAAGGACTTCATGTCTAAAACACCAAAAGCAATGGCAACAAAAGCCAAAATTGACAAATGGGATCTAATTAAACTCAAGAGCTTCTGCACAGCAAAAGAAACTACCATCAGAGTGAACAGGCAACCTACAAAATGGGAGAAAATTTTCACAACCTGCTCATCTGACAAAGGGCTAATATCCAGAATCTACAATGAACTCAAACAAATTTACAAGAAAAAAACAACCCCATCAAAAAGTGGGCAAAGGACATGAACAGACACTTCTCAAAAGAAGACATTTATGCAGCCAAAAAACATATGAAAAAATGCTCACCATCACTGGCCATCAGAGAAATGCAAGTCAAAACCACAATGAGATACCATCTCACACCAGTTAGAATGGCAATCATTAAAAAGTCAGGAAACAACAGGTGCTGGAGAGGATGTGGAGAAATAGGAACACTTTTACACTGTTGGTGGGACTGTAAACTAGTTCAACCATTGTGGAAGTCAGTGTGGCAATGCCTCAGGGATCTAGAACTGGAAATACCATTTGACCCAGCCATCCCATTACTGGGTATATATCCAAAGGACTATAAATCATGCTGCTATAAAGACACATGCACACGTATGTTTATTGCAGCACTATTCACAATAGCAAAGACTTGGAACCAACCCAAATGTCCAACAATGATAGACTGGATTAAGAAAATGTGGCACATATACACCATGGAATACTATGCAGCCATAAAAAATGATGAGTTCATGTCCTTTGTAGGGACATGGATGAAATTGGAAATCATCATTCTCAGTAAACTATCGCAAGAACAAAAAACCAAACACTGCATATTCTCACTCATAGGTGGGAATTGAACAATGAGAACACATGGACACAGGAAGGGGAACATCACACTCTGGGGACTGTTGTGTGGTGCGGGGAGTGGGGAGGGATAGCATTGGGAGATATACCTAATGCTAGGTGATGAGTTAGTGGGTGCAGCGCACCAGCATGTCACGTGTACACATATGTAACTAACCTGCACATTGTGCACATGTACCCTAAAATTT*AAAGTATAATAATAATAATAATAATAATAATAGTAATAGTAATAAAAACTAAGAAAAAAAAATAAAAAATAAAAAATAAAAAATGTACAAAAA****AAAGAAA*TACTATACAGC**CTTTAAAAAGAAGGAACTTCTGTCATTCGTGACAACATAGAAACATGTGG

**Clone 104; L1Hs; SpIRE(97/622)-104:Filled site spans: chr15:70729597-70735253**

Empty Site:

CTTCCGCCGCCTGTGAAGGGACCCACCGAGCTCCCAGGGCCTTTCTGCTGCTTCCTCTACCCCTGTATTTTGCTCGGCTCTGTAACTTGACTCAGCTTCAGAGAGTGGTTAAAAATTCTC

AACAAG**AAAAAG**ATGGGAACAAAAGACACTAGAGGGCAGA

CAGAGGGATGGGGGGAAGGGCTGAAAAACTGCATACTGCGTACTATGCTCAATACCTGGGTGACAAGATCAATCATACCACAAACCTCGGCAACACACAATACACCCAAGTAACAAACAT

Filled Site:

TGTAACTTGACTCAGCTTCAGAGAGTGGTTAAAAATTCTCAACAAG**AAAAAG**CTGTGGAGGGAGGAGCCAAGATGGCCGAATAGGAACAGCTCCGGTCTACAGCTCCCAGCGTGAGCGACGCAGAAGACGGTGATTTCTGCATTTCCATCTGAGCTTTGAAGAGAGCAGTGGTTCTCCCAGCACGCAGCTGGAGATCTGAGAACGGGCAGACTGCCTCCTCAAGTGGGTCCCTGACTCCTGACCCCCGAGCAGCCTAACTGGGAGGCACCCCCCAGCAGGGGCACACTGACACCTCACACGGCAGGGTATTCCAACAGACCTGCAGCTGAGGGTCCTGTCTGTTAGAAGGAAAACTAACAACCAGAAAGGACATCTACACCGAAAACCCATCTGTACATCACCATCATCAAAGACCAAAAGTAGATAAAACCACAAAGATGGGGAAAAAACAGAACAGAAAAACTGGAAACTCTAAAACGCAGAGCGCCTCTCCTCCTCCAAAGGAACGCAGTTCCTCACCAGCAACAGAACAAAGCTGGATGGAGAATGATTTTGACGAGCTGAGAGAAGAAGGCTTCAGACGATCAAATTACTCTGAGCTACGGGAGGACATTCAAACCAAAGGCAAAGAAGTTGAAAACTTTGAAAAAAATTTAGAAGAATGTATAACTAGAATAACCAATACAGAGAAGTGCTTAAAGGAGCTGATGGAGCTGAAAACCAAGGCTCGAGAACTACGTGAAGAATGCAGAAGCCTCAGGAGCCGATGCGATCAACTGGAAGAAAGGGTATCAGCAATGGAAGATGAAATGAATGAAATGAAGCGAGAAGGGAAGTTTAGAGAAAAAAGAATAAAAAGAAATGAGCAAAGCCTCCAAGAAATATGGGACTATGTGAAAAGACCAAATCTACGTCTGATTGGTGTACCTGAAAGTGATGTGGAGAATGGAACCAAGTTGGAAAACACTCTGCAGGATATTATCCAGGAGAACTTCCCCAATCTAGCAAGGCAGGCCAACGTTCAGATTCAGGAAATACAGAGAACACCACAAAGATACTCCTCGAGAAGAGCAACTCCAAGACACATAATTGTCAGATTCACCAAAGTTGAAATGAAGGAAAAAATGTTAAGGGCAGCCAGAGAGAAAGGTCGGGTTACCCTCAAAGGAAAGCCCATCAGACTAACAGCGGATCTCTCGGCAGAAACCCTACAAGCCAGAAGAGAGTGGGGGCCAATATTCAACATTCTTAAAGAAAAGAATTTTCAACCCAGAATTTCATATACAGCCAAACTAAGCTTCATAAGTGAAGGAGAAATAAAATACTTTATAGACAAGCAAATGTTGAGAGATTTTGTCACCACCAGGCCTGCCCTAAAAGAGCTCCTGAAGGAAGCGCTAAACATGGAAAGGAACAACCGGTACCAGCCACTGCAAAATCATGCCAAAATGTAAAGACCATTGAGACTAGGAAGAAACTGCATCAACTAATGAGCAAAATCACCAGCTAACATCATAATGACAGGATCAAATTCACACATAACAATATTAACTTTAAATATAAATGGACTAAATTCTGCAATTAAAAGACACAGACTGGCAAGTTGGATAAAGAGTCAAGACCCATCAGTGTGCTGTATTCAGGAAACCCATCTCACGTGCAGAGACACACATAGGCTCAAAATAAAAGGATGGAGGAAGATCTACCAAGCCAATGGAAAACAAAAAAAGGCAGGGGTTGCAATCCTAGTCTCTGATAAAACAGACTTTAAACCAACAAAGATCAAAAGAGACAAAGAAGGCCATTACATAATGGTAAAGGGATCAATTCAACAAGAGGAGCTAACTATCCTAAATATTTATGCACCCAATACAGGAGCACCCAGATTCATAAAGCAAGTCCTGAGTGACCTACAAAGAGACTTAGACTCCCACACATTAATAATGGGAGACTTTAACACCCCACTGTCAACATTAGACAGATCAACGAGACAGAAAGTCAACAAGGATACCCAGGAATTGAACTCAGCTCTGCACCAAGCAGACCTAATAGACATCTACAGAACTCTCCACCCCAAATCAACAGAATATACATTTTTTTCAGCACCACACCACACCTATTCCAAAATTGACCACATAGTTGGAAGTAAAGCTCTCCTCAGCAAATGTAAAAGAACAGAAATTATAACAAACTATCTCTCAGACCACAGTGCAATCAAACTAGAACTCAGGATTAAGAATCTCACTCAAAGCCGCTCAACTACATGGAAACTGAACAACCTGCTCCTGAATGACTACTGGGTACATAACGAAATGAAGGCAGAAATAAAGATGTTCTTTGAAACCAACGAGAACAAAGACACCACATACCAGAATCTCTGGGACGCATTCAAAGCAGTGTGTAGAGGGAAATTTATAGCACTAAATGCCTACAAGAGAAAGCAGGAAAGATCCAAAATTGACACCCTAACATCACAATTAAAAGAACTAGAAAAGCAAGAGCAAACACATTCAAAAGCTAGCAGAAGGCAAGAAATAACTAAAATCAGAGCAGAACTGAAGGAAATAGAGACACAAAAAACCCTTCAAAAAATCAATGAATCCAGGAGCTGGTTTTTTGAAAGGATCAACAAAATTGATAGACCGCTAGCAAGACTAATAAAGAAAAAAAGAGAGAAGAATCAAATAGACACAATAAAAAATGATAAAGGGGATATCACCACCGATCCCACAGAAATACAAACTACCATCAGAGAATACTACAAACACCTCTACGCAAATAAACTAGAAAATCTAGAAGAAATGGATACATTCCTCGACACATACACTCTCCCAAGACTAAACCAGGAAGAAGTTGAATCTCTGAATAGACCAATAACAGGCTCTGAAATTGTGGCAATAATCAATAGTTTACCAACCAAAAAGAGTCCAGGACCAGATGGATTCACAGCCGAATTCTACCAGAGGTACAAGGAGGAACTGGTACCATTCCTTCTGAAACTATTCCAATCAATAGAAAAAGAGGGAATCCTCCCTAACTCATTTTATGAGGCCAGCATCATTCTGATACCAAAGCCGGGCAGAGACACAACCAAAAAAGAGAATTTTAGACCAATATCCTTGATGAACATTGATGCAAAAATCCTCAATAAAATACTGGCAAACCGAATCCAGCAGCACATCAAAAAGCTTATCCACCATGATCAAGTGGGCTTCATCCCTGGGATGCAAGGCTGGTTCAATATACGCAAATCAATAAATGTAATCCAGCATATAAACAGAGCCAAAGACAAAAACCACATGATTATCTCAATAGATGCAGAAAAAGCCTTTGACAAAATTCAACAACCCTTCATGCTAAAAACTCTCAATAAATTAGGTATTGATGGGACGTATTTCAAAATAATAAGAGCTATCTATGACAAACCCACAGCCAATATCATACTGAATGGGCAAAAACTGGAAGCATTCCCTTTGAAAACTGGCACAAGACAGGGATGCCCTCTCTCACCGCTCCTATTCAACATAGTGTTGGAAGTTCTGGCCAGGGCAATCAGGCAGGAGAAGGAAATAAAGGGTATTCAATTAGGAAAAGAGGAAGTCAAATTGTCCCTGTTTGCAGACGACATGATTGTTTATCTAGAAAACCCCATCGTCTCAGCCCAAAATCTCCTTAAGCTGATAAGCAACTTCAGCAAAGTCTCAGGATACAAAATCAATGTACAAAAATCACAAGCATTCTTATACACCAACAACAGACAAACAGAGAGCCAAATCATGGGTGAACTCCCATTCACAATTGCTTCAAAGAGAATAAAATACCTAGGAATCCAACTTACAAGGGATGTGAAGGACCTCTTCAAGGAGAACTACAAACCACTGCTCAAGGAAATAAAAGAGGAGACAAACAAATGGAAGAACATTCCATGCTCATGGGTAGGAAGAATCAATATCGTGAAAATGGCCATACTGCCCAAGGTAATTTACAGATTCAATGCCATCCCCATCAAGCTACCAATGACTTTCTTCACAGAATTGGAAAAAACTACTTTAAAGTTCATATGGAACCAAAAAAGAGCCCGCATTGCCAAGTCAATCCTAAGCCAAAAGAACAAAGCTGGAGGCATCACACTACCTGACTTCAAACTATACTACAAGGCTACAGTAACCAAAACAGCATGGTACTGGTACCAAAACAGAGATATAGATCAATGGAACAGAACAGAGCCCTCAGAAATAATGCCGCGTATCTACAACTATCTGATCTTTGACAAACCTGAGAAAAACAAGCAATGGGGAAAGGATTCCCTATTTAATAAATGGTGCTGGGAAAACTGGCTAGCCATATGTAGAAAGCTGAAACTGGATCCCTTCCTTACACCTTATACAAAAATCAATTCAAGATGGATTAAAGATTTAAACGTTAAACCTAAAACCATAAAAACCCTAGAAGAAAACCTAGGCATTACCATTCAGGACATAGGCGTGGGCAAGGACTTCATGTCCAAAACACCAAAAGCAATGGCAACAAAAGACAAAATTGACAAATGGGATCTAATTAAACTAAAGAGCTTCTGCACAGCAAAAGAAACTACCATCAGAGTGAACAGGCAACCTACAACATGGGAGAAAATTTTTGCAACCTACTCATCTGACAAAGGGCTAATATCCAGAATCTACAATGAACTCAAACAAATTTACAAGAAAAAAACAAACAACCCCATCAAAAAGTGGGCGAAGGACATGAACAGACACTTCTCAAAAGAAGACATTTATGCAGCCAAAAAACACATGAAGAAATGCTCATCATCACTGGCCATCAGAGAAATGCAAATCAAAACCACTATGAGATATCATCTCACACCAGTTAGAATGGCAATCATTAAAAAGTCAGGAAACAACAGGTGCTGGAGAGGATGCGGAGAAATAGGAACACTTTTACACTGTTGGTGGGACTGTAAACTAGTTCAACCATTGTGGAAGTCAGTGTGGCGATTCCTCAGGGATCTAGAACTAGAAATACCATTTGACCCAGCCATCCCATTACTGGGTATATACCCAAAGGACTATAAATCATGCTGCTATAAAGACACATGCACACGTATGTTTATTGCGGCACTATTCACAATAGCAAAGACTTGGAACCAACCCAAATGTCCAACAATGATAGACTGGATTAAGAAAATGTGGCACATATACACCATGGAATACTATGCAGCCATAAAAAATGATGAGTTCATATCCTTTGTAGGGACATGGATGAAATTGGAAACCATCATTCTCAGTAAACTATCGCAAGAACAAAAAACCAAACACCGCATATTCTCACTCATAGGTGGGAATTGAACAATGAGATCACATGGACACAGGAAGGGGAATATCACACTCTGGGGACTGTGGTGGGGTCGGGGGAGGGGGGAGGGATAGCATTGGGAGATATACCTAATGCTAGATGACACATTAGTGGGTGCAGCGCACCAGCATGGCACATGTATACATATGTAACTAACCTGCACAATGTGCACATGTACCCTAAAACTT*AGAGTATAATAAAAAAAAAAAAAAAAAAAA****AAAAAG****A*TGGGAACAAAAGACACTAGAGGGCAGACAGAGGGATGGGGGGAAGGGCTGAAAAACTGCATACTGCGTACTATGCTCAA

**Clone 6; PA2; SpIRE(97/622)-6: Filled site spans: chr1:144965102-144970808**

Empty Site:

GTTCCTCGCCCTCGGGGAGCAACATGGATAATCTCAGTGATACCTTGAAGAAGCTGAAGATAACAGCTGTTGACAAGACTGAGGATAGTTTAGAAGGATGCTTGGATTGTCTGCTTCAAG

CCCTGGCTCAAAATAA**ATGATTCAGTGTAG**TTGTAGTGC

TAGATATATTATATGGAATTATCAGCAGTTTACCAGTTGGTTCTTTATTAGTACTCCTGATGTCTTAAACAAAAATGAATAACTGTTTATCTTTTAAAAAAATAAATAAATAAAAAATAA

Filled Site:

CTTGGATTGTCTGCTTCAAGCCCTGGCTCAAAATAA**ATGATTCAGTGTAG**GGGGGAGGAGCCAAGATGGCCGAATAGGAACAGCTCCGGTCTACAGCTCCAGCGTGAGCGACACAGAAGACGGGTGATTTCTGCATTTCCATCTGAGCTTTGAAGAGAGCAGTGGTTCTCCCAGCACGCAGCTGGAGATCTGAGAACGGGCAGACTGCCTCCTCAAGTGGGTCCCTGACCCCTGACCCCCGAGCAGCCTAACTGGGAGGCACCCCCCAGCAGGGGCACACTGACACCTCACACGGCAGGGTATTCCAACAGACCTGCAGCTGAGGGTCCTGTCTGTTAGAAGGAAAACTAACAAACAGAAAGGACATCCACACCGAAAACCCATCTGTACATCACCATCATCAAAGACCAAAAGTAGAGAAAACCTCAAAGATGGGGAAAAAACAGAACAGAAAAACTGGAAACTCTAAAACGCAGAGCGCCTCTCCTCCTCCAAAGGAACGCAGTTCCTCACCAGCAACGGAACAAAGCTGGATGGAGAATGATTTTGACGAGCTGAGAGAAGAAGGCTTCAGACGATCAAATTACTCTGAGCTATGGGAGCACATTCAAACCAAAGGCAAAGAAGTTGAAAACTTTGAAAAAAATTTAGAAGAATGTATAACTAGAATAACGAATACAGAGAAGTGCTTAAAGGAGCTGATGGAGCTGAAAACCAAGGCTCGAGAACTACGTGAAGAATGCAGAAGCCTCAGGAGCCGATGCGATCAACTGGAAGAAAGGGTATCAGCAATGGAAGATGAAATGAATGAAATGAAGCGAGAAGGGAAGTTTAGAGAAAAAAAGAATAAAAAGAAATGAGCAAAGCCTCCAATAAATATGGGACTATGTGAAAAGACCAAATCTACGTCTGATTGGTGTACCTGAAAGTGAGGCGGAGAATGGAACCAAGTTGGAAAACACTCTGCAGGATATTATCCAGGAGAACTTCCCCAATCTAGCAAGGCAGGCCAACGTTCAGATTCAGGAAATACAGAGAACGCCACAAAGACACTCCTCGAGAAGAGCAACTCCAAGACACATAATTGTCAGATTCACCAAAGTTGAAATGAAGGAAAAAATGTTAAGGGCAGCCAGAGAGAAAGGTCGGGTTACCCTCAAAGGGAAGCCCATCAGACTAACAGCAAATCTCTCGGCAGAAACCCTACAAGCCAGAAGAGAGTGGGGGCCAATATTCAACATTCTTAAAGAAAAGAATTTTCAACCCAGAATTTCATATCCAGCCAAACTAAGCTTCATAAGTGAAGGAGAAATAAAATACTTTACAGACAAGCAAATGCTGACCGATTTTGTCACCACCAGGCCTGCCCTAAAAGAGCTCCTGAAGGAAGTGCTAAACGTGGAAAGGAACAACCAGTACCAGCCACTGCAAAAACATGCCCAATTGTAAACACCATCGATGCTAGGAAGAAACTGCATCAACTAATGAACAAAATAGCCAGCTAACATCATAATGACAGGATCAAATTCACACATAACAATATTAACCTTAAATGTAAATGGGCTAAATGCTCCAATTAAAAGACACAGACTGGCAAATGGATAAAGAGTCAAGACCCATCAGTGTGCTGTATTCAAGAAACCCTTCTCACGTGCAGAGACACACATAGGCTGAAAATAAAAGGATGGAGGAAGATCTACCAAGCAAATGGAAAACAAAAAAAGGAAGGGGTTGCAATCCTAGTCTCTGATAAAACAGACTTTAAACCCACAAAGATCAAAAGAGACAAAGAAGGCCATTACATAATGGTAAAGGGATCAATTCAACAAGAGGAGCTAACTATCCTAAATATATATGCACCCAATACAGGAGCACCCAGATTCATAAAGCAAGTCCTGAGTGACCTACAAAGAGACTTAGACTCCCACACATTAATAATGGGAGACTTTAACACCCCACTGTCAACATTAGACAGATCAACGAGACAGAAAGTCAACAAGGATACCCAGGAATTGAACTCAGCTCTGCACCAAGCGGACCTAATAGACATCTACAGAACTCTCCACCCCAAATCAACAGAATATACATTTTTTTCAGCACCACACCACACCTATTCCAAAATTGACCACGTAGTTGGAAGTAAAGCTCTCCTCAGCAAATGTAAAAGAACAGAAATTATAACAAACTATCTCTCAGACCACAGTGCAATCAAACTAGAACTCAGGATTAAGAATCTCACTCAAAGCCGCTCAACTACATGGAAACTGAACAACCTGCTCCTGAATGACTACTGGGTACATAACGAAATGAAGGCAGAAATAAAGATGTTCTTTGAAACCAACGAGAACAAAGACACAACATACCAGAATCTCTGGGACGCATTCAAAGCAGTGTGTAGAGGGAAATTTATAGCACTAAATCCCCACAAGAGAAAGCAGGAAAGATCCAAAATTGACACCCTAACATCAAAATTAAAAGAACTAGAAAAGCAAGAGCAAACACATTCAAAAGCTAGCAGAAGGCAAGAAATAACTAAAATCAGAGCAGAACTGAAGGAAATAGAGACACAAAAAACCCTTCAAAAAATCAATGAATCCAGGAGCTGGTTTTTTGAAAGGATCAACAAAATTGATAGACCGCTAGCAAGACTAATAAAGAAAAAAAGAGAGAAGAATCAAATAGACACAATAAAAAATGATAAAGGGGATATCACCACCAATCCCACAGAAATACAAACTACCATCAGAGAATACTACAAACACCTCTACACAAATAAACTAGAAAATCTAGAAGAAATGGATACATTCCTCGACACATACACTCTCCCAAGACTAAACCAGGAAGAAGTTGAATCTCTGAATAGACCAATAACAGGAGCTGAAATTGTGGCAATAATCAATAGTTTACCAACCAAAAAGAGTCCAGGACCAGATGGATTCACAGCCGAATTCTACCAGAGGTACAAGGAGGAACTGGTACCATTCCTTCTGAAACTATTCCAATCAATAGAAAAAGAGGGAATCCTCCCTAACTCATTTTATGAGGCCAGCATCATTCTGATACCAAAGCCGGGCAGAGACACAACCAAAAAAGAGAATTTTAGACCAATATCCTTGATGAACATTGATGCAAAAATCCTCAATAAAATACTGGCAAACCGAATCCAGCAGCACATCAAAAAGCTTATCCACCATGATCAAGTGGGCTTCATCCCTGGGATGCAAGGCTGGTTCAATATACGCAAATCAATAAATGTAATCCAGCATATAAACAGAGCCAAAGACAAAAACCACATGATTATCTCAATAGATGCAGAAAAAGCCTTTGACAAAATTCAACAACCCTTCATGCTAAAAACTCTCAATAAATTAGGTATTGATGGGACGTATCTCAAAATAATAAGAGCTATCTATGACAAACCCACAGCCAATATCATACTGAATGGGCAAAAACTGGAAGCATTCCCTTTGAAAACTGGCACAAGACAGGGATGCCCTCTCTCACCACTCCTATTCAACATAGTGTTGGAAGTTCTGGCCAGAGCAATCAGGCAGGAGAAGGAAATAAAGGGTATTCAATTAGGAAAAGAGGAAGTCAAATTGTCCCTGTTTGCAGATGACATGATTGTTTATCTAGAAAACCCCATCGTCTCAGCCCAAAATCTCCTTAAGCTGATAAGCAACTTCAGCAAAGTCTCAGGATATAAAATCAATGTACAAAAATCACAAGCATTCTTATACACCAACAACAGACAAACAGAGAGCCAAATCATGAGTGAACTCCCATTCACAATTGCTTCAAAGAGAATAAAATACCTAGGAATCCAACTTACAAGGGATGTGAAGGACCTCTTCAAGGAGAACTACAAACCACTGCTCAAGGAAATAAAAGAGGATACAAACAAATGGAAGAACATTCCATGCTCATGGGTAGGAAGAATCAATATCGTGAAAATGGCCATACTGCCCAAGGTAATTTACAGATTCAATGCCATCCCCATCAAGCTACCAATGACTTTCTTCACAGAATTGGAAAAAACTACTTTAAAGTTCATATGGAACCAAAAAAGAGCCCGCATCGCCAAGTCAATCCTAAGGCAAAAGAACAAAGCTGGAGGCATCACACTACCTGACTTCAAACTATACTACAAGGCTACAGTAACCAAAACAGCATGGTACTGGTACCAAAACAGAGATATAGATCAATGGAACAGAACAGAGCCCTCAGAAATAATGCCACATACCTACAACTATCTGATCTTTGACAAACCTGAGAAAAACAAGCAATGGGGAAAGGATTCCCTATTTAATAAATGGTGCTGGGAAAACTGGCTAGCCATATGTAGAAAGCTGAAACTGGATCCCTTCCTTACACCTTATACAAAAATCAATTCAAGATGGATTAAAGATTTAAACGTTAGACCTAAAACCATAAAAACCCTAGAAGAAAACCTAGGCATTACCATTCAGGACATAGGCGTGGGCAAGGACTTCATGTCCAAAACACCAAAAGCAATGGCAACAAAAGCCAAAATTGACAAATGGGATCTCATAAAACTAAAGAGCTTCTGCACAGCAAAAGAAACTACCATCAGAGTGAACAGGCAAACTACAACATGGGAGAAAATTTTTGCAACCTACTCATCTGACAAAGGGCTAATATCCAGAATCTACAATGAACTCAAACAAATTTACAAGAAAAAAAACAAACAACCCCATCAAAAAGTGGGTGAAGGACATGAACAGACACTTCTCAAAAGAAGACATTTATGCAGCCAAAAAACACATGAAAAAATGCTCATCATCACTGGCCATCAGAGAAATGCAAATCAAAACCACTATTGAGATATCATCTCACACCAGTTAGAATGGCAATCATTAAAAAGTCAGGAAACAACAGGTGCTGGAGAGGATGTGGAGAAATACAAACACTTTTACACTGTTGGTGGGACTGTAAACTAGTTCAACCATTGTGGAAGTCAGTGTGGCGATTCCTCAGGGATCTAGAACTAGAAATACCATTTGACCCAGCCATCCCATTACTGGGTATATACCCAAATGACTATAAATCCTGCTGCTATAAAGACACATGCACACGTATGTTTATTGCGGCATTATTCACAATAGCAAAGACTTGGAACCAACCCAAATGTCCAACAATGATAGACTGGATTAAGAAAATGTGGCACATATACACCATGGAATACTATGCAGCCATAAAAAATGATGAGTTCATGTCCTTTGTAGGGACATGGATGAAATTGGAAACCATCATTCTCAGTAAACTATCGCAAGAACAAAAAACCAAACACCGCATATTCTCACTCATAGGTGGGAACTGAACAATGAGATCACATGGACACAGGAAGGGGAATATCACACTCTGGGGACTGTGGTGGGGTGGGGGGAGGGGGGAGGGATAGCATTGGGAGATATACCTAATGCTAGATGACGAGTTAGTGGGTGCAGCGCACCAGCATGGCACATGTATACATATGTAACTAACCTGCACAATGTGCACATGTACCCTAAAACTTAAAGTATT*AAAAAAAAAAAAAA*GC**ATGATTCAGTGTAG**TTGTAGTGCTAGATATATTATATGGAATTATCAGCAGTTTACCAGTTGGTTCTTTATTAGTACTCCTGATGTCTTAAACAAAAATGAATAACTGTTTATCTTTTAAAAAAATAAATAAATAAAAAATAAATAA

**Clone 28; PA2; SpIRE(97/622)-28: Filled site spans: chr3:12027816-12033415**

Empty Site:

TGGGAAAAAAGAGAGTCCCAGCTACATATTGTATTAGTCTCTCGTACCCTAGTCTGCCTCACACCTGGAAATTATCTAAGCTTACCACTGGTCACAGCACAGTCTGTTTCATTCCCAAAG

TAAAGAAAGCAC**AAGAATTACTTATAACC**TCTTTCCTCTA

TGTAAGTCATGAAAGTTACTCTTCTGATTAAATGTTGATTCTCCAAAGCCCCTTTCCTGACATTGATGCCTAAGGTCCTCCCTCTAAACTTTTTTCCACTGGGCCATGAAATATTAATAG

Filled Site:

AGTCTGTTTCATTCCCAAAGTAAAGAAAGCAC**AAGAATTACTTATAACC**AGCCGAATAGGAACAGCTCCGGTCTACAGCTCCCAGCGTGAGCAACGCAGAAGACGGGTGATTTCTGCATTTCCATCTGAGCTTTGAAGAGAGCAGTGGTTCTCCCAGCACGCAGCTGGAGATCTGAGAACAGGCAGACTGCCTCCTCAAGTGGGTCCCTGACCCCTGACCCCCGAGCAGCCTAACTGGGAGGCACCCCCCAGCAGGGGCACACTGACACCTCACACGGCACGGTATTCCAACAGACCTGCAGCTGAGGGTCCTGTCTGTTAGAAGGAAAACTAACAAACAGAAAGGACATCCACACCGAAAACCCATCTGTACATCACCATCATCAAAGACCAAAAGTAGATAAAACCACAAAGATGGGGAAAAAACAGAACAGAAAAACTGGAAACTCTAAAACGCAGAGCGCCTCTCCTCCTCCAAAGGAACGCAGTTCCTCACCAGCAACGGAACAAAGCTGGATAGAGAATGATTTTGACGAGCTGAGAGAAGAAGGCTTCAGACGATCAAATTACTCTGAGCTACGGGAGGACATTCAAACCAAAGGCAAAGAAGTTGAAAACTTTGAAAAAAATTTAGAAGAATGTATAACTAGAATAACCAATACAGAGAAGTGCTTAAAGGAGCTGATGGAGCTGAAAACCAAGGCTCGAGAACTACGTGAAGAATGCAGAAGCCTCAGGAGCCGATGCGATCAACTGGAAGAAAGGGTATCAGCAATGGAAGATGAAATGAATGAAATGAAGCGAGAAGGGAAGTTTAGAGAAAAAAGAATAAAAAGAAATGAGCAAAGCCTCCAAGAAATATGGGACTATGTGAAAAGACCAAATCTACGTCTGATTGGTGTACCTGAAAGTGATGCGGAGAATGGAACCAAGTTGGAAAACACTCTGCAGGATATCATCCAGGAGAACTTCCCCAATCTAGCAAGGCAGGCCAACGTTCAGATTCAGGAAATACAGAGAACGCCACAAAGATACTCCTCGAGAAGAGCAACTCCAAGACACATAATTGTCAGATTCACCAAAGTTGAAATGAAGGAAAAAATGTTAAGGGCAGCCAGAGAGAAAGGTCGGGTTACCCTCAAAGGGAAGCCCATCAGACTAACAGCGGATCTCTCGGCAGAAACCCTACAAGCCAGAAGAGAGTGGGGGCCAATATTCAACATTCTTAAAGAAAAGAATTTTCAACCCAGAATTTCATATCCAGCCAAACTAAGCTTCATAAGTGAAGGAGAAATAAAATACTTTACAGACAAGCAAATGCTGACCGATTTTGTCACCACCAGGCCTGCCCTAAAAGAGCTCCTCAAGGAAGCGCTAAACATGGAAAGGAACAACCGGTACCAGCCGCTGCAAAATCATGCCAAATTGTAAAGACCATTGAGACTAGGAAGAAACTGCATCAACTAACGAGCAAAATCACCAGCTAACATCATAATGACAGGATCAAATTCACACATAACAATATTAACTTTAAATGTAAATGGACTAAATTCTCCAATTAAAAGACACAGACTGGCAAGTTGGATAAAGAGTCAAGACCCATCAGTGTGCTGTATTCAGGAAACCCATCTCACGTGCAGAGACACACATAGGCTCAAAATAAAAGGATGGAGGAAGATCTACCAAGCAAATGGAAAACAAAAAAAGGCAGGGGTTGCAATCCTAGTCTCTGATAAAACAGACTTTAAACCAACAAAGATCAAAAGAGACAAAGAAGGCCATTACATAATGGTAAAGGGATCAATTCAACAAGAGGAGCTAACTATCCTAAATATATATGCACCCAATACAGGAGCACCCAGATTCATAAAGCAAGTCCTGAGTGACCTACAAAGAGACTTAGACTCCCACACATTAATAATGGGAGACTTTAACACCCCACTGTCAACATTAGACAGATCAACGAGACAGAAAGTCAACAAGGATACCCAGGAATTGAACTCAGCTCTGCACCAAGCGGACCTAATAGACATCTACAGAACTCTCCACCCCAAATCAACAGAATATACATTTTTTTTCAGCACCACACCACACCTATTCCAAAATTGACCACATACTTGGAAGTAAAGCTCTCCTCAGCAAATGTAAAAGAACAGAAATTATAACAAACTATCTCTCAGACCACAGTGCAATCAAACTAGAACTCAGGATTAAGAATCTCACTCAAAGCCGCTCAACTACATGGAAACTGAACAACCTGCTCCTGAATGACTACTGGGTACATAACGAAATGAAGGCAGAAATAAAGATGTTCTTTGAAACCAACGAGAACAAAGACACAACATACCAGAATCTCTGGGACGCATTCAAAGCAGTGTGTAGAGGGAAATTTATAGCACTAAATGCCCACAAGAGAAAGCAGGAAAGATCCAAAATTGACAACCTAACATCACAATTAAAAGAACTAGAAAAGCAAGAGCAAACACATTCAAAAGCTAGCAGAAGGCAAGAAATAACTAAAATCAGAGAAGAACTGAAGGAAATAGAGACACAAAAAACCCTTCAAAAAATCAATGAATCCAGGAGCTGGTTTTTTGAAAGGATCAACAAAATTGATAGACCGCTAGCAAGACTAATAAAGAAAAAAAGAGAGAAGAATCAAATAGACACAATAAAAAATGATAAAGGGGATATCACCACCAATCCCACAGAAATACAAACTACCATCAGAGAATACTACAAACACCTCTACACAAATAAACTAGAAAATCTAGAAGAAATGGATACATTCCTCGACACATACACTCTCCCAAGACTAAACCAGGAAGAAGTTGAATCTCTGAACAGACCAATAACAGGAGCTGAAATTGTGGCAATAATCAATAGTTTACCAACCAAAAAGAGTCAAGGACCAGATGGATTCACAGCCGAATTCTACCAGAGGTACAAGGAGGAACTGGTACCATTCCTTCTGAAACTATTCCAATCAATAGAAAAAGAGGGAATCCTCCCTAACTCATTTTATGAGGCCAGCATCATTCTGATACCAAAGCCGGGTAGAGACACAACCAAAAAAGAGAATTTTAGACCAATATTCTTGATGAACATTGATGCAAAAATCCTCAATAAAATACTGGGAAACCGAATCCAGCAGCACATCAAAAAGCTTATCCACCATGATCAAGTGGGCTTCATCCCTGGGATGCGAGGCTGGTTCAATATACGCAAATCAATAAATGTAATCCAGCATATAAACAGAGCCAAAGACAAAAACCACATGATTATCTCAATAGATGCAGAAAAAGCCTTTGACAAAATTCAACAACCCTTCATGCTAAAAACTCTCAATAAATTAGGTATTGATGGGACGTATTTCAAAATAATAAGAGCTATCTATGACAAACCCACAGCCAATATCATACTGAATGGGCAAAAACTGGAAGCATTCCCTTTGAAAACTGGCACAAGACAGGGATGCCCTCTCTCACCACTCCTATTCAACATAGTGTTGGAAGTTCTGGCCAGGGCAATCAGGCAAGAGAAAGAAATAAAGGGTATTCAATTAGGAAAAGAGGAAGTCAAATTGTCCCTGTTTGCAGACGACATGATTGTTTATCTAGAAAACCCCATCGTCTCAGCCCAAAATCTCCTTAAGCTGATAAGCAACTTCAGCAAAGTCTCAGGATACAAAATCAATGTACAAAAATCACAAGCATTCCTATACACCAACAACAGACAAACAGAGAGCCAAATCATGAGTGAACTCCCATTCACAATTGCTTCAAAGAGAAGAAAATACCTAGGAATCCAACTTACAAGGGATGTGAAGGACCTCTTCAAGGAGAACTACAAACCACTGCTCAAGGAAATAAAAGAGGATACAAACAAATGGAAGAACATTCCATGCTCAAGGGTAGGAAGAATCAATATCGTGAAAATGGCCATACTGCCCAAGGTAATTTACAGATTCAATGCCATCCCCATCAAGCTACCAATGACTTTCTTCACAGAATTGGAAAAAACTACTTTAAAGTTCATATGGAAGCAAAAAAGAGCCCGCATCACCAAGTCAATCCTAAGCCAAAAGAACAAAGCTGGAGGCATCACACTACCTGACTTCAAACTATACTACAAGGCTACAGTAACCAAAACAGCATGGTACTGGTACCAAAACAGAGATATAGATGAATGGAACAGAACAGAGCCCTCAGAAATAACGCCGCATACCTACAACTATCTGATCTTTGACAAACCTGAGAAAAACAAGCAATGGGGAAAGGATTCCCTATTTAATAAATGGTGCTGGGAAAACTGGCTAGCCATATGTAGAAAGCTGAAACTGGATCCCTTCCTTACACCTTATACAAAAATCAATTCAAGATGGATTAAAGATTTAAACGTTAGACCTAAAACCATAAAAACCCTAGAAGAAAACCTAGGCATTACCATTCAGGACATAGGCGTGGGCAAGGACTTCATGTCCAAAACACCAAAAGCAATGGCAACAAAAGCCAAAATTGACAAATGGGATCTAATTAAACTAAAGAGCTTCTGCACAGCAAAAGAAACTACCATCAGAGTGAACAGGCAACCTACAACATGGAAGAAAATTTTCGCAACCTACTCATCTGACAAAGGGCTAATATCCAGAATCTACAAAGAACTCAAACAAATTTACAAGAAAAAAACAAACAACCCCATCAAAAAGTGGGCGAAGGACATGAACAGACACTTCTCAAAAGAAGACATTTATGCAGCCAAAAAACACATGAAAAAATGCTTATCATCACTGGCCATCAGAGAAATGCAAATCAAAACCACTATGAGATATCATCTCACACCAGTTAGAATGGCAATCATTAAAAAGTCAGGAAACAACAGGTGCTGGAGAGGATGAGGAGAAATAGGAACACTTTTACACTGTTGGTGGGACTGTAAACTAGTTCAACCATTGTGGAAGTCAGTGTGGCGATTCCTCAGGGATCTAGAACTAGAAATACCATTTGACCCAGCCATCCCATTACTGGGTATATACCCAAATGACTATAAATCATGCTGCTATAAAGACACATGCACACGTATGTTTATTGCGGCATTATTCACAATAGCAAAGACTTGGAACCAACCGAAATGTCCAACGATGATAGACTGGATTAAGACAATGTGGCACATATACACCATGGAATACTAAGCAGCCATAAAAAATGATGAGTTCATGTCCTTTGTAGGGACATGGATGAAATTGGAAACCATCATTCTCAGTAAACTATCGCCAAGAACAAAAAACCAAACACCGCATATTCTCACTCATAGGTGGGAATTGAACAATGAGATCACATGGACACAGGAAGGGGAATATCACACTCTGGGGACTGTGGTGGGGAGGGGGGAGGGAAAGAAAAAATAAAAATAAAAAAATAAAAAAATAAAAAAAAGAAGTAGAGCACAGT*AAAAAAAAAAA****AAGAA*TTACTTATAACC**TCTTTCCTCTATGTAAGTCATGAAAGTTACTCTTCTGATTAAATGTTGATTCTCCAAAGCCCCTTTCCTGACATTGATGCCTAAGGTCCTCCCTCTAAACTTTTTTCCACTGGGCCATGAA

**Clone 35; PA2; SpIRE(97/622)-35: Filled site spans: chr4:98320362-98326069**

Empty Site:

TTTATTGGTTGATTTGGTGGCCTAAAGTATGTTTCTCTTTTTAGATTAGTGTTGATAGGTTTTGTGTTTTTTGTAGCCAGAGTATCTGGAGAAGAGCTGTTTGAAAATGTCCCCGTTACG

TGATTCTCTTTTCAGAAT**GATTCAGTGTAG**TTGTAGTGCT

AGATATATTATATGGAATTATCAGCAGTTTACCAGTTGGTTCTTTATTAGTACTCCTGATGTCTTAAACAAAAATGAATAATTGTTTATCTTTTCTCAAAGTGTCTCATCTGTTCAGTAA

Filled Site:

AGTATCTGGAGAAGAGCTGTTTGAAAATGTCCCCGTTACGTGATTCTCTTTTCAGAAT**GATTCAGTGTAG**GGGGGAGGAGCCAAGATGGCCGAATAGGAACAGCTCCGGTCTACAGCTCCCAGCGTGAGCAACGCAGAAGACGGGTGATTTCTGCATTTCCATCTGAGCTTTGAAGAGAGCAGTGGTTCTCCCAGCACGCAGCTGGAGATCTGAGAACGGGCAGACTGCCTCCTCAAGTGGGTCCCTGACCCCTGACCCCCGAGCAGCCTAACTGGGAGGCACCCCCCAGCAGGGGCACACTGACACCTCACACGGCAGGGTATTCCAACAGACCTGCAGCTGAGGGACCTGTCTGTTAGAAGGAAAACTAACAAACAGAAAGGACATCCACACCAAAAACCCATCTGTACATCACCATCATCAAAGACCAAAAGTAGATAAAACCACAAAGATGGGGAAAAAACAGAACAGAAAAACTGGAAACTCTAAAACGCAGAGCGCCTCTCCTCCTCCAAAGGAACGCAGTTCCTCACCAGCAACGGAACAAAGCTGGATGGAGAATGATTTTGACGAGCTGAGAGAAGAAGGCTTCAGACGATCAAATTACTCTGAGCTACGGGAGGACATTCAAACCAAAGGCAAAGAAGTTGAAAACTTTGAAAAAAATTTAGAAGAATATATAACTAGAATAACCAATACAGAGAAGTGCTTAAAGGAGCTGATGGAGCTGAAAACCAAGGCTCGAGAACTACGTGAAGAATGCAGAAGCCTCAGGAGCCGATGCGATCAACTGGAAGAAAGGATATCAGCAATGGAAGATGAAATGAATGAAATGAAGCGAGAAGGGAAGTTTAGAGAAAAAAGAATAAAAAGAAATGAGCAAAGCCTCCAAGAAATATGGGACTATGTGAAAAGACCAAATCTACGTCTGATTGGTGTACCTGAAAGTGAGGCGGAGAATGGAACCAAGTTGGAAAACACTCTGCAGGATATTATCCAGGAGAACTTCCCCAATCTAGCAAGGCAGGCCAACGTTCAGATTCAGGAAATACAGAGAACGCCACAAAGATACTCCTCGAGAAGAGCAACTCCAAGACACATAATTGTCAGATTCACCAAAGTTGAAATGAAGGAAAAAATGTTAAGGGCAGCCAGAGAGAAAGGTCGGGTTACCCTCAAAGGGAAGCCCATCAGACTAACAGCGGATCTCTCGGCAGAAACCCTACAAGCCAGAAGAGTGGGGGCCAATATTCAACATTCTTAAAGAAAAGAATTTTCAACCCAGAATTTCATATCCAGCCAAACTAAGCTTCATAAGTGAAGGAGAAATAAAATACTTTACAGACAAGCAAATGCTGAGAGATTTTGTCACCACCAGGCCTGCCCTAAAAGAGCTCCTGAAGGAAGCGCTAAACATGGAAAGGAACAACCGGTACCACCCGCTGCAAAATCATGCCAAAATGTAAAGACCATCGAGACTAGGAAGAAACTGCATCAACTAACGAGCAAAATCACCAGCTAACATCATAATGACAGGATCAAATTCACACATAACAATATTAACTTTAAATGTAAATGGACTAAATTCTCCAATTAAAAGACACAGACTGGCAAGTTGGATAAAGAGTCAAGACCCATCAGTGTGCTGTATTCAGGAAACCCATCTCACGTGCAGAGACACACATAGGCTCAAAATAAAAGGATGGAGGAAGATCTACCAAGCAAATGGAAAACAAAAAAAGGCAGGGATTGCAATCCTAGTCTCTGATAAAACAGACTTTAAACCAACAAAGATCAAAAGAGACAAAGAAGGCCATTACATAATGGTAAAGGGATCAATTCAACAAGAGGAGCTAACTATCCTAAATATATATGCACCCAATACAGGAGCACCCAGATTCATAAAGCAAGTCCTGAGTGACCTACAAAGAGACTTAGACTCCCACACATTAATAATGGGAGACTTTAACACCCCACTGTCAACATTAGACAGATCACCGAGACAGAAAGTCAACAAGGATACCCAGGAATTGAACTCAGCTCTGCACCAAGCAGACCTAATAGACATCTACAGAACTCTCCACCCCAAATCAGCAGAATATACATTTTTTTCAGCACCACACCACACCTATTCCAAAATTGACCACATAGTTGGAAGTAAAGCTCTCCTCAGCAAATGTAAAAGAACAGAAATTATAACAAACTATCTCTCAGACCACAGTGCAATCAAACTAGAACTCAGGATTAAGAATCTCACTCAAAGCCGCTCAACTACATGGAAACTGAACAACCTGCTCCTGAATGACTACTGGGTACATAACGAAATGAAGGCAGAAATAAAGATGTTCTTTGAAACCAACGAGAACAAAGACACAACATACCAGAATCTCTGGGACGCATTCAAAGCAGTGTGTAGAGGGAAATTTATAGCACTAAATGCCCACAAGAGAAAGGAGGAAAGATCCAAAATTGACACCCTAACATCACAATTAAAAGAACTAGAAAAGCAAGAGCAAACACATTCAAAAGCTAGCAGAAGGCAAGAAATAACTAAAATCAGAGCAGAACTGAAGTGAAATAGAGACACAAAAAACCCTTCAAAAAGTCAATGAATCCAGGAGCTGGTTTTTTGAAAGGATCAACAAAATTGATAGACCGCTAGCAAGACTAATAAGGAAAAAAAGAGAGAAGAATCAAGTAGACACAATAAAAAATGATAAAGGGGATATCACCACCGATCCCACAGAAATACAAACTACCATCAGAGAATACTACAAACACCTCTATGCAAATAAACTAGAAAATCTAGAAGAAATGGATACATTCCTTGACACATACACTCTCCCAAGACTAAACCAGGAAGAAGTTGAATCTCTGAATAGACCAATAACAGGAGCTGAAATTGTGGCAATAATCAATAGTTTACCAACCAAAAAGAGTCCAGGACCAGATGGATTCGCAGCCGAATTCTACCAGAGGTACAAGGAGGAACTGGTACCATTCCTTCTGAAACTATTCCAATCAATAGAAAAAGAGGGAATCCTCCCTAACTCATTTTATGAGGCCAGCATCATTCTGATACCAAAGCCGGGCAGAGACACAACCAAAAAAGAGAATTTTAGACCAATATCCTTGATGAACATTGATGCAAAAATCCTCAATAAAATACTGGCAAACCGAATCCAGTAGCACATCAAAAAGCTTATCCACCATGATCAAGTGGGCTTCATCCCTGGGATGCAAGGCTGGTTCAATATACGCAAATCAATAAATGTAATCCAGCATATAAACAGAGCCAAAGACAAAAACCACATGATTATCTCAATAGATGCAGAAAAAGCCTTTGACAAAATTCAACAACCCTTCATGCTAAAAACTCTCAATAAATTAGGTATTGATGGGAGGTATTTTAAAATAATAAGAGCTATCTATGACAAACCCACAGCCAATATCATACTGAATGGGCAAAAACTGGAAGCATTCCCTTTGAAAACTGGCACAAGACAGGGATGCCCTCTCTCACCACTCCTATTCAACATAGTGTTGGAAGTTCTGGCCAGGGCAATCAGGCAGGAGAAGGAAATAAAGGGTATTCAATTAGGAAAAGAGGAAGTCAAATTGTCCCTGTTTGCAGACGACATGATTGTTTATCTAGAAAACCCCATCGTCTCAGCCCAAAATCTCCTTAAGCTGATAAGCAACTTCAAAGTCTCAGGATACAAAATCAATGTACAAAAATCACAAGCATTCTTATACACCAACAACAGACAAACAGAGAGCCAAATCATGAGTGAACTCCCATTCACAATTGCTTCAAAGAGAATAAAATACCTAGGAATCCAACTTACAAGGGATGTGAAGGACCTCTTCAAGGAGAACTACAAACCACTGCTCAAGGAAATAAAAGAGGACACAAACAAATGGAAGAACATTCCATGCTCATGGGTAGGAAGAATCAATATCGTGAAAATGGCCGTACTACCCAAGGTAATTTACAGATTCAATGGCATCCCCATAAAGCTACCAATGACTTTCTTCACAGAATTGGAAAAAACTACTTTAAAGTTCATATGGAACCAAGAAAGAGCCCGCATCGCCAAGTCAATCCTAAGCCAAAAGAACAAAGCTGGAGACATCACACTACCTGACTTCAAACTATACTACAAGGCTACAGTAACCAAAACAGCATGGTACTGGTACCAAAACAGAGATATAGATCAATGGAACAGAACAGAGCCCTCAGAAATAACGCCGCATACCTACAACTATCTGATCTTTGACAAACCTGAGAAAAACAAGCAATGGGGAAAGGATTCCCTATTTAATAAATGGTGCTGGGAAAACTGGCTAGCCATATGTAGAAAGCTGAAACTGGATCCCTTCCTTACACCTTATACAAAAATCAATTCAAGATGGATTAAAGATTTAAACGTTAGACCTAAAACCATAAAAACCCTAGAAGAAAACCTAGGCATTACCATTCAGTACATAAGCGTGGGCAAGGACTTCATGTCCAAAACACCAAAAGCAATGGCAACAAAAGCCAAAATTGACAAATGGGATCTCATAAAACTAAAGAGCTTCTGCACAGCAAAAGAAACTACCATCAGAGTGAACAGGCAACCTACAAAATGGGAGAAAATTTTCGCAACCTACTCATCTGACAAAGGGCTAATATCCAGAATCTACAATGAACTCAAACAAATTTACAAGAAAAAGACAAACAACCCCATCAAAAAGTGGGCGAAGGACATGAACAGACACTTCTCAAAAGAAGACATTTATGCAGCCAAAAAACACATGAAAAAATGCTCACCATCACTGGCCATCAGAGAAATGCAAATCAAAACCACTATGAGATATCATCTCACACCAGTTAGAATGGCAATCATTAAAAAGTCAGGAAACAACAGGTGCTGGAGAGGATGTGGAGAAATAGGAACACTTTTACACTGTTGGTGGGACTGTAAACTAGGTCAACCATTGTGGAAGTCAGTGTGGCGATTCCTCAGGGATCTAGAACTAGAAATACCATTTGACCCAGCCATCCCATTACTGGGTATATACCCAAATGACTATAAATCATGCTGCTATAAAGACACATGCACATGTATGTTTATTGTGGCATTATTCACAATAGCAAAGACTTGGAACCAACCCAAATGTCCAACAGTGATAGACTGGATTAAGAAAATGTGGCACATATACACCATGGAATACTATGCAGCCATAAAAAATGATGAGTTCATGTCCTTTGTAGGGACATGGATGAAATTGGAAATCATCATTCTCAGTAAACTATCGCAAGAACAAAAAACCAAACACCGAATATTCTCACTCATAGGTGGGAATTGAACAATGAGATCACATGGACACAGGAAGGGGAATATCACACTCTGGGGACTGTGGTGGGGTGGGGGGAGGGGGGAGGGATAGCATTGGGCGATATACCTAATGCTAGATGACGAGTTAGTGGGTGCAGCGCACCAGCATGGCACATGTATACATATGTAACTAACGTGCACAATATGCACATGTACCCTAAAACTTAAAGTATT*AAAAAAAAAAA****G*ATTCAGTGTAG**TTGTAGTGCTAGATATATTATATGGAATTATCAGCAGTTTACCAGTTGGTTCTTTATTAGTACTCCTGATGTCTTAAACAAAAATGAATAATTGTTTATCTTTTCTCAAAGTGTCTCATCTGTTCA

**Clone 64; PA2; SpIRE(97/622)-64: Filled site spans: chr7:14704399-14710091**

Empty Site:

GTAGTCCCAGCTACGCGGGAGGCTGAGGCAGGAGAATGGCGCGAACCAGGGAGGCGGAGCTTGCAGTGAGCCGAGATCACGCCACTGCACTCCAGCCTGGGCGACAGAGCGAGACTCCAT

CTCAAAAAAAAAAAAA**AAAAGAAAAAAAAAAGAAAA**TGTTT

GCCAATCTTTTCTTCTCCTATTACAAAACAAAATGAAAAACAAACCAAAATAACACTGCTGGATTTCTAATGAGATTGTACTGAACCTATAGATCTGTTTGGGGGAGAATCTTTAATTTC

Filled Site:

GATCACGCCACTGCACTCCAGCCTGGGCGACAGAGCGAGACTCCATCTCAAAAAAAAAAAAA**AAAAGAAAAAAGAAA.AAAA**GgGGGAGGAGCCAAGATGGCCGAATAGGAACAGCTCCGGTCTACAGCTCCCAGCGAGACCGACGCAGAAGACGGGTGATTTCTGCATTTCCATCTGAGCTTTGAAGGGAGCAGTGGTTCTCCCAGCACGCAGCTGAAGATCTGAGAACCGGCAGACTGCCTCTTCAAGTGGGTCCCTAACCCCTGACCCCTGAGCAGCCTAACTGGGAGGCACCCTCCAGCAGGGGCACACTGACACCTCACACGGCAGGGTATTCCAATAGACCTGCAGCTGAGGGTCCTGTCTGTTAGAAGGAAAACTAACAAACAGAAAGGACATCCACACCAAAAACCCATCTGTACATCACCATCATCAAACACCAAAAGTAGATAAAACCACAAAGATGGGGAAAAAACAGAACAGAAAAACTGGAAGCTCTAAAAATCAGAGCGCCTCTCCTCCTCCAAAGGAACGCAGCTCCTCACCAGCAACGGAACAAAGCTGGATGGAGAATGACTTTGACGAGCTGAGAGAAGAAGGCTTCAGACGATCAAATTACTCTGAGCTACGGGAGGACATTCAAACCAAAGGCAAAGAAGTTGAAAACTTTGAAAAAAATTTAGAAGAATGTATAACTAGAATAACCAATACAGAGAAGTGCTTAAAGGAGCTGATGGAGCTGAAAACCAAGGCTCGAGAGCTACGTGAAGAATGCAGAAGCCTCAGGAGCCGATGCGATCAACTGGAAAAAGGGTGTCAGCAATGGAAGATGAAATGAATGAAATGAAGCGAGAAGGGAAGTTTAGAGAAAAAAGAATAAAAAGAAATGAGCAAAGCCTCCAAGAAATATGGGACTATGTGAAAAGACCAAATCTACGTCTGATTGGTGTACCTGAAAGTGATGGGGAGAATGGAACCAAGTTGGAAAACACTCTGCAGGATATTATCCAGGAGAACTTCCCCAATCTAGCAAGGCAGGCCAACGTTCAGATTCAGGAAATACAGAGAACGCCACAAAGATACTCGAGAAGAGCAACTCCAAGACACATAATTGTCAGATTCACCAAAGTTGAAATGAAGGAAAAAATGTTAAGGGCAGCCAGAGAGAAAGGTCGGGTTCCCCTCAAAGGGAAGCCCATCAGACTAACAGCGGATCTCTCGGCAGAAACCCCACAAGCCAGAAGAGAGTGGGGGCCAATATTCAACATTCTTAAAGAAAAGAATTTTCAACCCAGAATTTCATATCCAGCCAAACTAAGCTTCATAAGTGAAGGAGAAATAAAATACTTTACAGACAAGCAAATGCTAAGAGATTTTGTCACCACCAGACCTGCCCTAAAAGAGCTCCTGAAGGAAGCGCTAAATATGGAAAGGAACAACCAGTACCAGCCACTGCAAAATCATGCCAAAATGTAAAGACCATCGAGACTAGGAAGAAACTGCATCAAATAACGAGCAAAATCACCAGCTAACATCATGATGACAGGATCAAATTCACACATAACAATATTAACTTTAAATGTAAACGGACTAAATGCTCCAATTAAAAGACACAGACTGGCAAATTGGATAAAGAGTCAAGACCCATCAGTGTGCTGTATTCAGGAAACCCATCTCACGTGCAGAGACACACATAGGCTCAAAATAAAAGGATGGAGGAAGATCTACCAAGCAAATGGAAAACAAAAAAAGGCAGGGGTTGCAATCCTAGTCTCTGATAAAACAGACTTTAAACCAACAAAGATCAAAAGAGACAAAGAAGGCCATTACATAATGGTAAAGGGATCAATTCAACAAGAAGAGCTAACTATCCTAAATATACATGCACCCAATACAGGAGCACCCAGATTCATAAAGCAAGTCCTGAGTGACCTACAAAGAGACTTAGACTCCCACACAATAATAATGGGAGACTTTAACACCCCACTGTCAACATTAGACAGATCAACGAGACAGAAAGTCAACAAGGATACCCAGGAATTGAACTCAGCTCTGCACCAAGAGGACCTAATACACATCTACAGAACTCTCCACCCCAAATCAACAGAATATACATTTTTTTCAGCACCACACCACACCTATTCCAAAATTGACCACATACTTGGAAGTAAAGCTCTCCTCAGCAAATGTAAAAGAACAGAAATTATAACAAACTATCTCTCAGACCACAGTGCAATCAAACTAGAACTCAGGATTAAGAATCTCACTCAAAGCCGCTCAACTACATGGAAACTGAACAACCTGCTCCTGAATGACTACTGGGTACATAACGAAATGAAGGCAGAAATAAAGATGTTCTTTGAAACCAACGAGAACAAAGACACAACATACCAGAATCTCTGGGACGCATTCAAAGCAGTGTGTAGAGGGAAATTTATAGCACTAAATGCCCACAAGAGAAAGCAGGAAAGATCCAAAATTGACAGCCTAACATCACAATTAAAAGAACTAGAAAAGCAAGAGCAAACACATTCAAAAGCTAGCAGAAGGCAAGAAATAACTAAAATCAGAGCAGAACTGAAGGAAATAGAGACACAAAAAACCCTTCAAAAAGTCAATGAATCCAGGAGCTGGTTTTTTGAAAGGATCAACAAAATTGACAGACCGCTAGCAAGACTAATAAAGAAAAAAAGAGAGAAGAATCAAATAGACGCAATAAAAAATGATAAAGGGGATATCACCACCGATTCCACAGAAATACAAACTACCATCAGAGAATACTACAAACACCTCTATGCAAATAAACTAGAAAATCTAGAAGAAATGGATAAATTCCTCGACACATACACTCTCCCAAGACTAAACCAGGAAGAAGTTGAATCTCTTAATAGACCAATAACAGGAGCTGAAATTGTGGCAATAATCAATAGTTTACCAACCAAAAAGAGTCCAGGGCCAGATGGATTCACAGCCGAATTCTACCAGAGGTACAAGGAGGAACTGGTACCATTCCTTCTGAAACTATTCCAATCAATAGAAAAAGAGGGAATCCTCCCTAACTCATTTGATGAGGCCAGCATCATTCTGATACCAAAGCCGGGCAGAGACACAACCAAACAAGAGAATTTTAGACCAATATCCTTGATGAACATTGATGCAAAAATCCTCAATAAAATACTGGCAAACCGAATCCAGCAGCACATCAAAAAGCTTATCCACCATGATCAAGTGGGCTTCATCCCTGGGATGCAAGGCTGGTTCAATATACGCAAATCAATAAATGTAATCCAGCATATAAACAGAGCCAAAGACAAAAACCACATGATTATCTCAATAGATGCAGAAAAAGCCTTTGACAAAATTCAACAACCCTTCATGCTAAAAACTCTCAATAAATTAGGTATCGATGGGACGTATTTCAAAATAATAAGAGCTATCTATGACAAACCCACAGCCAATATCATACTGAATGGGCAAAAACTGGAAGCATTCCCTTTGAAAACTGGCACAAGACAGGGATGCCCTCTCTCACCACTCCTATTCAACATAGTGTTGGAAGTTCTGGCCAGGGCAATTAGGCAGGAGAAGGAAATAAAGGGTATTCAATTAGGAAAAGAGGAAGTCAAATTGTCCCTGTTTGCAGACGACATGATTGTATATCTAGAAAACCCCATTGTCTCAGCCCAAAATCTCCTTAAGCTGATAAGCAACTTCAGCAAAGTCTCAGGATACAAAATCAATGTACAAAAATCACAAGCATTCTTATACACCAACAACAGACAGAGAGCCAAATCATGAGTGAACTCCCATTCACAACTGCTTCAAAGAGAATAAAATACCTAGGAATCCACCTTACAAGGGATGTGAAGGACCTCTTCAAGGAGAACTACAAACCACTGCTCAAGGAAATAAAAGAGGATACAAACAAATGGAAGAACATTCCATGCTCATGGGTAGGAAGAATCAATATCGTGAAAATGGCCATACTGCCCAAGGTAATTTACAGATTCAATGCCATCCCCATCAAGCTACCAATGACTTTCTTCACAGAATTGGAAAAAACTACTTTAAAGTTCATATGGAACCAAAAGAGAGACGGCATCGCCAAGTGAATCCTAAGCCAAAAGAACAAAGCTGGAGGCATCACACTACCTGACTTCAAACTATACTACAAGGCTACAGTAACCAAAACAGCATGGTACTGGTACCAAAACAGAGATATAAATCAATGGAACAGAACAGAGCCCTCAGAAATAACGCCGCATACCTACAACTGTCTGATCTTTGACAAACCTGAGAAAAACAAGAAATGGGGAAAGGATTCCCTATTTAATAAATGGTGCTGGGAAAACTGGCTAGCCATACGTAGAAAGCTGAAACTGGATCCCTTCCTTACACCTTATACAAAAATCAATTCAAGATGGATTAAAGATTTAAACGTTAGACCTAAAACCATAAAAACCCTAAAAGAAAACCTAGGCTTTACCATTCGGGACATAGGCATGGGCAAGGACTTCATGTCCAAAACACCAAAAGCAATGGCAACAAAAGCCAAAATTGACAAATCGGATCTAATTAAACTAAAGAGCTTCTGCACAGCAAAAGAAACTACCATCAGAGTGAACAGGCAACCTACAAAATGGGAGAAGATTTTCGCAACCTACTCATCTGACAAAGGGCTAATATCCAGAATCTACAATGAACTCAAACAAATTTACAAGAAAAAATCAAACAACCCCATCAAAAAGTGGGCGAAGGACATGAACAGACACTTCTCAAAAGAAGACATTTATGCAGCCAACAGGCACATGAAAAAATGCTCATCATCACTGGCCATCAGAGAAATGCAAATCAAAACCACTATGAGATACCATCTCACACCAGTTAGAATGGCAGTCATTAAAAAGTCAGGAAACAACAGGTGCTGGAGAGGATGTGGAGAAATAGGAACACTTTTACACTGTTGGTGGGACTGTGAACTAGTTCAACCATTGTGGAAGTCAGTGTGGCGATTCCTCAGGGATCTAGAACTGGAAATACCATTTGACCCAGCCATCCCATTACTGGGTATATACCCAAATGACTATAAATCATGCTGCTATAAAGACACATGCACACGTATGTTTATTGCGGCATTATTCACAATAGCAAAGACTTGGAACCAACCCAAATGTCCAACAATGATAGACTGGATTAAGAAAATGTGGCACATATACACCATGGAACACTATGCAGCCATAAAAAATGATGAGTTCATGTCCTTTGTAGGGACATGGATGAAATTGGAAATCATCATTCTCAGTAAACTATCGCAAGAACAAAAAACCAAACACCGCATATTCTCACTCAGAGGTGGGAATTGAACAATGAGATCACATGGACACAGGAAGGGGAATATCACACTCTGGGGACTGTGGTGGGGTGGGGGGAGGGGAGAGGGATAGCATTGGGAGATATACCTAATGCTAGATGAGGAGTTAGTGGGTGCAGCGCACCAGCATGGCACATGTATACATATGTAACTAACCTGCACAATGTGCACATGTACCCTAAAACTTAAAGTATAATT*AAAAAAAAAAAA*GAAAAG**AAAAGAAAAAAAAAAGAAAA**TGTTTGCCAATCTTTTCTTCTCCTATTACAAAACAAAATGAAAAACAAACCAAAATAACACTGCTGGATTTCTAATGAGATTGTAC

**Clone 70; PA2; SpIRE(97/622)-70: Filled site spans: chr8:68362348-68368033**

Empty Site:

TGAAGATTATTTATTAAACTCATAGAGTTTATACACATTCTCAATTTGATGTTTTCATCATTAAAACCAAGATTATATTTGCTTAATATTTAGCAGAGTCCACTGTCAAT

**GAAAAAAGTACATC**ACTGAGCTGAAGAAAGATAAACTGGT

AATTTCTACTCTGCTCTGCACTAGTGAAGGTGCTATAGAATCTATTAACTATGTCTATTAGTTTACTTTCTTCTGTATTTTAAAGTATTTAAAAGT

Filled Site:

GCTTAATATTTAGCAGAGTCCACTGTCAAT**GAAAAAAGTACATC**GATTAATTCCAAAGGCCATGGAGGGAGGAGCCAAGATGGCCGAATAGGAACAGCTCCAGTCTACAGCTCCCAGCGTGAGCGACGCAGAAGACGGGTGATTTCTGCATTTCCATCTGAGCTTTGAAGAGAGCAGTGGTTCTCCCAGCACGCGGCTGGAGATCTGAGAACGGGCAGACTGCCTCTTCAAGTGGGTCCCTGACCCCCGAGCAGCCTAACTGGGAGGCACCCCCCAGCAGGGGCACACTGACACCTCACACGGCAGGATATTCCAACAGACCTGCAGCTGAGGGTCCTGTCTGTTAGAAGGAAAACTAACAAACAGAAAGGACAACCACACCAAAAACCCATCTGTACATCACCATCATCAAAGACCAAAAGTAGATAAAACCACAAAGATGGGGAAAAAACAGAACAGAAAAACTGGAAGCTCTAAAAATCAGAGCGCCTCTCCTCCTCCAAAGGAACGCAGCTCCTCACCAGCAACAGAACAAAGCTGGATGGAGAATGACTTTGACGAGCTGAGAGAAGAAGGCTTCAGACGATCAAATTACTCTGAGCTACGGGAGGACATTCAAACCAAAGGCAAAGAAGTTGAAAACTTTGAAAAAAATTTAGAAGAATGTATAACTAGAATAACCAATACAGAGAAGTGCTTAAAGGAGCTGATGGAGCTGAAAACCAAGGCTCGAGAACTACGTGAAGAATGCAGAAGCCTCAGGAGCCGATGCGATCAACTGGAAGAAAGGGTGTCAGCAATGGAAGATGAAATGAATGAAATGAAGCGAGAAGGGAAGTTTAGAGAAAAAAGAATAAAAAGAAATGAGCAAAACCTCCAAGAAATATGGGACTATGTGAAAAGACCAAATCTACGTCTGATTGGTGTACCTGAAAGTGATGGGGAGAATGGAACCAAGTTGGAAAACACTCTGCAGGATATTATCCAGGAGAACTTCCCCAATCTAGCAAGGCAGGCCAACGTTCAGATTCAGGAAATACAGAGAACGCCACAAAGATACTCCTCGAGAAGAGCAACTCCAAGACACATAATTGTCAGATTCACCAAAGTTGAAATGAAGGAAAAAATGTTAAGGGCAGCCAGAGAGAAAGGTCGGGTTACCCTCAAAGGGAAGCCCATCAGACTAACAGCGGATCTCTCGGCAGAAACCCTACAAGCCAGAAGAGAGTGGGGGCCAATATTCAACATTCTTAAAGAAAAGAATTTTCAACCCAGAATTTCATATCCAGCCAAGCTAAGCTTCATAAGTGAAGGAGAAATAAAATACTTTACAGACAAGCAAATGCTGAGAGATTTTGTCACCACCAGACCTGCCCTAAAAGAGCTCCTGAAGGAAGCGCTAAACATGGAAAGGAACAACCAGTACCAGCCGCTGCAAAATCATGCCAAAATGTAAAGACCATCGAGACTAGGAAGAAACTGCATCAACTAACGAGCAAAATCACCAGCTAACATCATAATGACAGGATCAAATTCACACATAACAATATTAACTTTAAATGTAAATGGACTAAATGCTCCAATTAAAAGACACAGACTGGCAAATTGGATAAAGAGTCAAGACCCATCAGTGTGCTGTATTCAGGAAACCCATCTCACGTGCGGAGACACACATAGGCTCAAAATAAAAGGATGGAGGAAGATCTACCAAGCAAATGGAAAACAAAAAAAGGCAGGGGTTGCAATCCTAGTCTCTGATAAAACAGACTTTAAACCAACAAAGATCAAAAGAGACAAAGAAGGCCATTACATAATGGTAAAGGGATCAATTCAACAAGAAGAGCTAACTATCCTAAATATATATGCACCCAATACAGGAGCACCCAGATTCATAAAGCAAGTCCTGAGTGACCTACAAAGAGACTTAGACTCCCACACAATAATAATGGGAGACTTTAACACCCCACTGTCAACATTAGACAGATCAACGAGACAGAAAGTCAACAAGGATACCCAGGAATTGAACTCAGCTCTGCACCAAGCGGACCTAATAGACATCTACAGAACTCTCCACCCCAAATCAACAGAATATACATTTTTTTCAGCACCACACCACACCTATTCCAAAATTGACCACATAGTTGGAAGTAAAGCTCTCCTCAGCAAATGTAAAAGAACAGAAATTATAACAAACTATCTCTCAGACCACAGTGCAATCAAACTAGAACTCAGGATTAAGAATCTCACTCAAAGCTGCTCAACTACATGGAAACTGAACAACCTGCTCCTGAATGACTACTGGGTACATAACGAAATGAAGGCAGAAATAAAGATGTTCTTTGAAACCAACGAGAACAAAGACACAACATACCAGAATCTCTGGGATGCATTCAAAGCAGTGTGTAGAGGGAAATTTATAGCACTAAATGCCCACAAGAGAAAGCAGGAAAGATCCAAAATTGACACCCTAACATCACAATTAAAAGAACTAGAAAAGCAAGAGCAAACACATTCAAAAGCAGGCAGAAGGCAAGAAATAACTAAAATCAGAGAAGAACTGAAGGAAATAGAGACACAAAAAACCCTTCAAAAAATCAATGAATCCAGGAGCTGGTTTTTTGAAAGGATCAACAAAATTGATAGACCGCTAGCAAGATTAATAAAGAAAAAAAGAGAGAAGAATCAAATAGACACAATAAAAAATGATAAAGGGAATATCACCACCGATCCCACAGAAATACAAACTACCATCAGAGAATACTACAAACACCTCTACGCAAATAAACTAGAAAATCTAGAAGAAATGGATAAATTCCTCGACACATACACTCTCCCAAGACTAAACCAGGAAGAAGTTGAATCTCTTAATAGACCAATAACAGGAGCTGAAATTGTGGCAATAATCAATAGCTTACCAACCAAAAAGAGTCCAGGACCAGATGGATTCACAGCCGAATTCTACCAGAGGTACAAGGAGGAACTGGTACCATTCCTTCTGAAACTATTCCAATCAATAGAAAAAGAGGGAATCCTCCCTAACTCATTTTATGAGGCCAGCATCATTCTGATACCAAAGCCGGGCAGAGACACAACCAAAAAAGAGAATTTTAGACCAATATCCTTGATGAACATTGATGCAAAAATCCTCAATAAAATACTGGCAAACCGAATCCAGCAGCACATCAAAAAGCTTATCCACCATGATCAAGTGGGCTTCATCCCTGGGATGCAAGGCTGGTTCAATATACGCAAATCAATAAATGTAATCCAGCATATAAACAGAACCAAAGTCAAAAACCACATGATTATCTCAATAGATGCAGAAAAAACCTTTGACAAAATTCAACAACCCTTCATGCTAAAAACTCTCAATAAATTAGGTATTGATGGGACGTATTTCAAAATAATAAGAGCTATCTATGACAAACCCACAGCCAATATCATACTGAATGTGCAAAAACTGGAAGCATTCCCTTTGAAAACTGGCACAAGACAGGGATGCCCTCTCTCACCACTCCTATTCAACATAGTGTTGGAAGTTCTGGCCAGGGCAATTAGGCAGGAGAAGGAAATAAAGGGTATTCAATTAGGAAAAGAGGAAGTCAAATTGTCCCTGTTTGCAGATGACATGATTGTATATCTAGAAAACCCCATTGTCTCAGCCCAAAATCTCCTTAAGCTGATAAGCAACTTCAGCAAAGTCTCAGGATACAAAATCAATGTACAAAAATCACAAGCATTCTTATACACCAACAACACACAAACAGAGAGCCAAATCATGAGTGAACTCCCATTCACAATTGCTTCAAAGAGAATAAAATACCTAGGAATCCAACTTACAAGGGATGTGAAGGACCTCTTCAAGGAGAACTACAAACCACTGCTCAAGGAAATAAAAGAGGATACAAACAAATGGAAGAACATTCCATGCTCATGGGTAGGAAGAATCAATATTGTGAAAATGGCCATACTGCCCAAGGTAATTTATAGATTCAATGTCATCCCCATCAAGCTACCAATGACTTTCTTCACAGAATTGAAAAAAACTACTTTAAAGTTCATATGGAACCAAAAAAGAGCCCGCATCGCCAAGTCAATCCTAAGCCAAAAGAACAAAGCTGGAGGCATCACACTACCTGACTTCAAACTTTACTACAAGGCTACAGTAACCAAAACAGCATGGTACTGGTACCAAAACAGAGATATAGATTAATGGAACAGAACAGAGCCCTCAGAAATAACACCGCATACCTACAACTGTCTGATCTTTGATAAACCTGAGAAAAACAAGAAATGGGGAAAGGATTCCCTATTTAATAAATGGTGCTGGGAAAACTGGCTAGCCATATGTAGAAAGCTGAAACTGGATCCCTTCCTTACACCTTATACAAAAATCAATTCAAGATGGATTAAAGATTTAAACGTTAGACCTAAAACCATAAAAACCCTAGAAGAAAACTTAGGCATTACCATTCAGGACATAGGCATGGGCAAGGACTTCATGTCTAAAACACCAAAAGCAATGGCAACAAAAGACAAAATTGACAAATGGGATCTAATTAAACTAAAGAGCTTCTGCACAGCAAAAGAAACTACCATCAGAGTGAACAGGCAACCTACAAAATGGGAGAAAATTTTCGCAACCTACTCATCTGACAAAGGGCTAATATCCAGAATCTACAATGAACTCAAACAAATTTACAAGAAAAAAACAAACAACCCCATCAAAAAGTGGGCGAAGGACATGAACAGACACTTCTCAAAAGAAGACATTTATGCAGCCAAAAAACACATGAAAAAATGCTCATCATCACTGGCCATCAGAGAAATGCAAATCAAAACCACAATGAGATACCATCTCACACCAGTTAGAATGGCAATCATTAAAAAGTCAGGAAACAACAGGTGCTGGAGAGGATGTGGAGAAATAGGAACACTTTTACATTGTTGGTGGGACTGTAAACTAGTTCAACCATTGTGGAAGTCAGTGTGGCGATTCCTCAGGGATCTAGAACTAGAAATACCATTTGACCCAGCCATCCCATTACTGGGTATATACCCAAAGGACTATAAATCATGCTGCTATAAAGACACATGCACATGTATGTTTATTGTGGCATTATTCACAATAGCAAAGACTTGGAACCAACCCAAATGTCCAACAATGATAGACTGGATTAAGAAAATGTGGCACATATACACCATGGAATACTATGCAGCCATAAAAAATGATGAGTTCATGTCCTTTGTAGGGACATGGATGAAATTGGAAATCATCATTCTCAGTAAACTATCACAAGAACAAAAAACCAAACACCACATATTCTCACTCATAGGTGGGAATTGAACAATGAGATCACATGGACACAAGAAAGGGAATACCACACTCTGGGGACTGTGGTGGGGTGGGGGGAGGGGGGATGGATAGCATTGGGAGATATACCTAATGCTAGATGACGAGTTAGTGGGTGCAGCGCACCAGCATGGCACATGTATACATATGTAACTAACCTGCACAATGTGCACATGTACCCTAAAACCTAAAGTATAAT*AAAAAAAGAAAAGAAAAAAAAAAAAAAAAAAAAAAAAA****GAAAAAA*GTACATC**ACTGAGCTGAAGAAAGATAAACTGGTAATTTCTACTCTGCTCTGCACTAGTGAAGGTGCTATAGAATCTATTAACTATGTCT

**Clone 72; PA2; SpIRE(97/622)-72: Filled site spans: chr8:128707281-128712802**

Empty Site:

GGAGTGAGGCTTTAGGAATCAGTCACTCATTTAACAAATATTTACTGCCAATCTTCTGTGTAGAGTCCCTGGGGATACAAGATAGAAGATATTATTTCCCCTCTAACACCACCACCCAAG

**AGAAAGAGT**ATGAGTTGGGGAAAAAAAAAAAGAAATGTGG

CACATATACCCCATGGAATACTATGCAGCCATAAAAAATGATGAGTTCACGTCCTTTGTAGGGACATGGATGAAATTGGAAATCATCTTTCTCAGTAAACTATCGCAAGAACAAAAAACC

Filled Site:

TTTACTGCCAATCTTCTGTGTAGAGTCCCTGGGGATACAAGATAGAAGATATTATTTCCCCTCTAACACCACCACCCAAG**AGAAAGAGT**TCCCATTCCAGAGGCAGGTAGAGATACATCAAAATCAAACACAAGATTCATTTTTAACAATCTCTTTAATTTTATTCCAAGATAATAAGAATTGCTTTCTTTAAAATATTTTAATTGAAATATTTCCATCGGGGGGAGGAGCCAAGACGGCCGAATAGGAAAAGCTCTGGTCTACAGCTCCCAGAGTGAGCGACGCAGAAAATGGGTGATTTCTGCATTTCCATCTGAGCTTTGAAGAGAGCAGTGGTTCTCCCAGCACGCAGCTGGAGATCTGAGAACGGGCAGACTGCCTCCTCAAGTGGGTCCCTGACCCCTGACCCCCGAGCAGCCTAACTGGGAGGCAACCCCCAGCAGGGGCACACTGACACCTCACACGGCAGGGTACTCCAACAGACCTGCAGCTGAGGGTCCTGTCTGTTAGAAGGAAAACTAACAAACAGAAAGGACATCCACACCAAAAACACATCTGTACATCACCATCATCAAAGACCAAAAGTAGATAAAACCACAAAGATGGGGAAAAAACAGAGCAGAAAAACTGGAAACTCTAAAAAGCAGAGCGCCTCTCCTCCTCCAAAGGAACACAGTTCCTCACCAGCAATGGAACAAAGCTAGAGGGAGAATGACTTTGACGAGCTGAGAGAAGAAGGCTTCAGACGATCAAATTACTCCGAGCTACGGGAGGACATTCAAACCAAAGGCAAAGAAGCTGAAAACTTTGAAAAAAATTTAGAAGAATGTATAACTAGAATAACCAATACAGAGAAGTGCTTAAAGGAGCTGATGGAGCTGAAAACCAAGGCTTGAGAACTACATGAAGAATGCAGAAGCCTCAGGAGCCAATGCGATCAACTGGAAGAAAGGGTATCAGCAATGGAAGATGAAATGAATGAAATGAAGCGAGAAGGGAAGTTTAGAGAAAAAAGAATAAAAAGAAATGAGCAAAGCCTCCAAGAAATCAAGAAATATGGGACTATGTGAAAAGACCAAATCTACGTCTGATTGGTGTACCTGAAAGTGATGGGGAGAATGGAACCAAGTTGGAAAACACTCTGCAGGATATTATCCAGGAGAACTTCCCCAATCTAGCAAGGCAGGCCAACGTTCAGATTCAGGAAATACAGAGAACGCCACAAAGATACTCCTCGAGAAGAGCAACTCCAAGACACATAATTGTCAGATTCACCAAAGTTGAAATGAAGGAAAAAATGTTAAGGGCAGCCAGAGAGAAAGGTCGGGTTACCCTCAAAGGGAAGCCCATCAGACTAACAGCGGATCTCTCGGCAGAAACCCTACAAGCCAGAAGAGAGTGGGGGCCAATATTCAACATTCTTAAAGAAAATCATTTTCAACCCAGAATTTCATATCCAGCCAAACTAAGCTTCATAAGTGAAGGAGAAATAAAATACTTTACAGACAAGCAAATGCTGAGAGATTCTGTCACCACCAGGCCTGCCCTAAAAGACCTCCTGAAGGAAGCGCTAAAGATGGAAAGGAACAACCAATACCAGCTGTGGCAAAATCATGCCAAAATTTAAAGACCATCGAGACTAGGGAGAAACTGCAGGAACTAACGATCAAAAGAACCAGCTAACATCATAATGACAGGATCAAATTCACACATAACAATATGAACTTTAAATGTAAATGGACTAAATGCTCCAATTAAAAGACACAGACTGGCAAATTGGATAAAGAGTCAAGACCCATCAGTGTGCTGTATTCAGGAAACGCATCTCACGTGCAGAGACACACACAGGCTCAAAATAAAATGATGGAGGAAGACCTACCAAGCAAATGGAAATCAAAAAAAGGCAGGGGTTGCAATCCTAGTCTCTGATAAAACAGACTTTAAACCAACAAAGATCAAAAGACACAAAGAAGGCCATTATGTAATGGTAAAGGGATCAATTCAACAAGAAGAGCTAACTATCCTAAATATATATGCACTCAATACAGGAGCACCCAGATTCATAAAGCAAGTCCTGAGCGACCTACAGAGAGACTTAGACTCCCACACATTAATAATGGGAGACTTTAACACCCCACTGTCAACATTAGACAGATCAATGAGACAGAAAGTCAACAAGGATACCCAGGAATTGAACTCAGCTCTGCACCAAGCGGACCTAATAGACATCTACAGAACTCTCCACCCCAAATCAACAGAATATACATTTTTTTCAGCACCATACCACACCTATTCCAAAATTGACCACATACTTGGAAGTAAAGCTCTCCTTAGCAAATGTAAAAGAAGAGAAATTATAATAAACTATCTCTCAGACCACAGTGCAATCAAACTAGAACTCAGGATTAAGAATCTCACTCAAAACCTCTCAACTACATGGAAACTGAACAACCTGCTCCTGAATGACTACTGGGTACATAACGAAATGAAGGCAGAAATAAAGATGTTCTTTGAAACCAACGAGAACAAAGACACAACATACCAGAATCTCTGGGACACATTCAAAGCAGTGTGTGGAGGGAAATTTATAGCACTAAATGCCCACAAGAGAAAGCAGGAAAGATCCAAAATTGACACCCTAACATCACAATTAAAAGAAATAGAAAAGCAAGAGCAAACACATTCAAAAGCTAGCAGAAGGCAAGAAATAACTATTTCCTTCAGTTCTGCTCTGATTTTAGAGACACAAAAAACCCTTCAAAAAATTAATCCAGGAGCTGGTTTTTTGAAAGGATCAACAAAATTGATAGACCGCTAGCAAGATTAATAAAGAAAAAAAGAGAGAAGAATCAAATAGATTCAATAAAAAATGATAAAGGGGATATCACCACAAATCCCACAAAAATACAAACTACCATCAGAGAATACTACAAACACCTCTACACAAATAAACTAGAAAATCTAGAAGAAATGGATAAATTCCTCGACACATACACTCTCCCAAGACTAAACCAGGAAGAAGTTGAATCTCTGAATAGACCAATAACAGGATCTGAAATTGTGGCAATAATCAATAGCTTACCAACCAAAAAGAGTCCAGGACCAGATGGATTCACAGCCGAATTCTACCAGAGGTACAAGGAGGAACTGGTACCATTCCTTCTGAAACTATTCCAATCCATAGAAAAAGAGGGAATCCTCCCTAACTCATTTTGTAAGGCCAGCATCATCCTGATACCAAAGCCTGGCAGAGACACAACCAAAAAAGAGAATTTTAGACCAATATCCTTGATGAACATTGATGCAAAAATCCTCAATAAAATACTGGCAAAATGAATCCAGCAGCACATCAAAAAGCTTATCCACCATGATCAAGTGGGCTTCATCCCTGGGATGCAAGGCTGGTTCAATATACACAAATCAATAAATGTAACCCAGCATATAAACAGAACCAAAGACAAAAACCACATGATTATCTCAATAGATGCAGAAAAGGCCTTTGACAAAATTCAACAACCCTTCATGCTAAAAACTCTCAATAAATTAGGTATTGATGGGACGTATCTCAAAATAATAAGAGCTATCTATGACAAACCCACAGCCAATATCATACTGAATGGGCAAAAACTGGAAGCATTCCCTTTGAAAACTGGCACAAGACGGGATGCCCTCTCTCACCACTCCTATTCAACATAGTGTTGGAAGTTCTGGCCAGGGCAATTAGGCAGGAGAAGGAAATAAAGGGTATTCAATTAGGAAAAGAGGAAGTCAAATTGTCCCTGTTTGCAGACGACATGATTGTATACCTAGAAAACCCCATTGTCTCAACTCAAAATCTTCTTAAGCTGATAAGCAACTTCAGCAAAGTCTCAGGATACAAAATCAATGGACAAAAATCACAAGCATTCTTATACACCAACAACAGACAAACAGCCAAATCATGAGTGAAATCCCATTCACAATTGCTTCAAAGAGAATAAAATACCTAGGAATCCAACTTACAAGGGATGTGAAGGACCTCTTCAAGGAGAACTACAAACCACTGCTCAAGGAAATAAAAGAGGATACAAACAAATGGAAAAACAAGCAATGGGGAAAGGATTCCCTATTTAATAAATGGTGCTGGGTAAACTGGCTAGCCATATGTAGGAAGCTGAAACTGGATCCCTTCCTTACACCTTATACAAAAATCAATTCAAGATGGATTAAAGATTTAAACATTAGACCTAAAACCATAAAAACCCTAGAAGAAAACCTAGGCATTACCATTCAGGATATAGGCATGGGCAAGGACTTCATGTCCAAAACACCAAAAGCAATGGCAACAAAAGACAAAATTGACAAATGGGATCTAATTAAACTAAAGAGCTTCTGCACAGCCAAAGAAACTACCATTAGAGTGAACAGTCAACCTACAAAATGGGAGAAAATTTTCGCAACCTACTCATCTGACAAAGGGCTAATATCCAGAATCTACAATGAACTCAAACAAATTTACAAGAAAAAAACAAACAACCCCATGAAAAAGTGGGCGAAGGACATGAACAGCCACTTCTCAAAAGAAGACATTTATGCAGCCAAAAAACACTTGAAAAAATGCTCATCATCACTGGCCATCAGAGAAATGCAAATCAAAACCACTATGAGATACCATCTCACACCAGTTGGAATGGCAATCATTAAAAAGTCAGGAAACAACAGGTGCTGGAGAGGATGTGGAGAAATAGGAACACTTTTACACTGTTGGTGGGACTGTAAACTAGTTCAACCATTGTGGAAGTGAGTGTGGCGATTCCTCAGGGATCTAGAACTAGAAATACCATTTGACCCAGCCATCCCATTACTGGGTATATACCCAAATGACTATAAATCATGCTACTATAAAGACACATGCACACATATGTTTATTGCGGCATTATTCACAATAGCAAAGACTTGGAACCAACCCAAATGTCCAACAATGATAGACTGGATTAAGAAAATGTGGCACATATACACCATGGAATACTATGCAGCCATAAAAAATGATGAGTTCATGTCCTTTGTAGGGACATGGATGAAATTGGAAATCATCATTCTCAGTAAACTATCGCAAGAACAAAAAACCAAACACCGCATATTCTCACTTATAGGTGGGAATTGAACTATGAGAACACATGGACACAGGAAGGGGAATATCACACTCTGGGGACTGTGGTGGGGTCGGGGGTGGGGGGAGGGATAGCATTGGGAGATATACCTAATGCTAGATGAAGAGTTAGTGGGTGCAGCACACCAGCATGGCACATGTATACATATGTAACTAACCTGCACAATGTGCACATGTACCCTAAAACTTAAAGTATAATT*AAAAAAAAAAAAAA****AGAAA*GAGT**ATGAGTTGGGGAAAAAAAAAAAGAAATGTGGCACATATACCCCATGGAATACTATGCAGCCATAAAAAATGATGAGTTCACGTCCTTTGTAGGGACATGGATGAAATTGGAAATCATCTTTCTCAGTAAACTATCGCAAGAACAAAAAAC

**Clone 75; PA2; SpIRE(97/622)-75: Filled site spans: chr10:7057937-7063606**

Empty Site:

TATAGTACTAATATTCCTGCCCATAAGTGTCAATAATAACACCCACCTCATGGGGTGTGTGTGTGTAGTATTTGAAATGCCTTTGTCATACCGATGGGGGTTGGCAGAGACTGACATGAG

AGATTCTTGTAG**AAAGAAGGCAGAGATC**TTGAACTCCTGA

CCTCAAGTGATCCACTTGCCTCGGTCTCCCAAAGTCCTTGCATTACAGGTGTGAGGCTCAGCACTTTGGGAGGTTGAGGTGGGTGGATCACCTGAGGTCAGGAGTTCAAGAACAACCTGG

Filled Site:

CTTTGTCATACCGATGGGGGTTGGCAGAGACTGACATGAGAGATTCTTGTAG**AAAGAAGGCAGAGATC**GAGGGGGAGGAGCCAAGATGGCCAAATAGGAACAGCTCCGGTCTACAGCTCCTAGCGTGAGCGACGCAGAAGACAGGTGATTTCTGCATTTCCATCTGAGCTTTGAAGAGAGCAGTGGTTCTCCCAGCATGCAGCTGGAGATCTGAGAACGGGCAGACTGCCTCCTCAAGTGGGTCCCTGACCCCTGACTCCCGAGCAGCCTAACTGGGAGGCACCCCCCAGCAGGGGCACACTGACACCTCACACGGCAGGGTATTCCAACAGACCTGCAGCTGAGGGTCCTGTCTGTTAGAAGGAAAACTAACAAACAGAAAGGACATCCACACCAAAAACCCATCTGTACATCACCATCATCAAAGACCAAAAGTAGATAAAACCACAAAGATGGGGAAAAACAGAACAGAAAAACTGGAAACTCTAAAATGCAGAGCGCCTCTCCTCCTCCAAAGGAATGCAGTTCCTCACCAGCAACGGAACAAAGCTGGACGGAGAATGATTTTGACGAGCTGAGAGAGGAAGGCTTCAGACGATCAAATTACTCTGAGCTACGGGAGGACATTCAAACCAAAGGCAAAGAAGTTGAAAACTTTGAAAAAAATTTAGAAGAATGTATAACTAGAATAACTAATACAGAGAAGTGCTTAAAGGAGCTGATGGAGCTGAAAACCAAGGCTTGAGAACTACGTGAAGAATGCAGAAGCCTCAGGAGCTGATGCGATCAACTGGAAGAAAGGGTATCAGCAAGGGAAGATGAAATGAATGAAATGAAGTGAGAAGGGAAGTTTAGAGAAAAAGGAATAAAAAGAAATGAGCAAAGCCTCCAAGAAATATGGGACTATGTGAAAAGACCAAATCTACGTCTGATTGGTGTACCTGAAAGTGATGGGGAGAATGGAACCAAGTTGGAAAACACTCTGCAGGATATTATCCAGGAGAACTTCCCCAATCTAGCAAGGCAGGCCAACGTTCAGATTCAGGAAATACAGAGAACACCACAAAGATACTCCTCGAGAAGAGCAACTCCAAGACACATAATTGTCAGATTCACCAAAGTTGAAATGAAGGAAAAAATGTTAAGGGCAGCCAGAGAGAAAGGTCGGGTTACCCTCAAAGGGAAGCCCATCAGACTAACAGCGGATCTCTCGGCAGAAACCCTACAAGCCAGAAGAGAGTGGGGGCCAATATTCAACATTCTTAAAGAAAAGAATTTTCAACCCAGAATTTCATATCCAGCCAAACTAAGCTTCATAAGTGAAGGAGAAATAAAATACTTTACAGACAAGCAAATGCTGAGAGATTTTGTCACCACCAGGCCTGCCCTAAAAGAGCTCCTGAAGGAAGTGCTAAACATGGAAAGGAACAACTGGTACCAGCTGCTGCAAAATCATGCCAAAATGTAAAGACCATCAAGACTAGGAAGAAACTGCATCAACTAACGAGCAAAATAACCAGCTAACATCATCATGACAGGATCAAATTCACACATAACAATATTAACTTTAAATGTAAATGGACTAAATGCTCCAATTAAAAGACACAGACTGGCAAGTTGGATAAAGAGTCAAGACCCATCAGTGTGCTGTATTCAGGAAACCCATCTCACCTGCAGAGACACACATAGGCTCAAAATAAAAGGATGGAGGAAGATCTACCAAGCAAATGGAAAACAAAAAAAGGCAGGGGTTGCAATCCTAGTCTCTGATAAAACAGACTTTAAACCAACAAAGATCAAAAGAGACAAAGAAGGCCATTACATAATGGTAAAGGGATCAATTCAACAAGAGGAGCTAACTATCCTAAATATATATGCACCCAATACAGGAGCACCCAGATTCATAAAGCAAGTCCTGAGTGACCTACAAAGAGACTTAGACTCCCACACATTAATACTGGGAGACTTTAACACCCCTCTGTCAACATTAGACAGATCAACGAGACAGAAAGTCAACAAGGATACCCAGGAATTGAACTCAGCTCTGCACCAAGCAGACCTAATAGACATCTACAGAACTCTCCACCCCAAATCAACAGAATATACATTTTTGTCAGCACCACACCACACCTATTCCAAAATTGACCACATAGTTGGAAGTAAAGCTCTCCTCAGCAAATGTAAAAGAACAGAAATTATAACAAACTATCTCTCAGACCACAGTGCAATCAAACTAGAACTCAGGATTAAGAATCTCACTCAAAACCGCTCAACTACATGGAAACTGAACAACCTGCTCCTGAATGACTACTGGGTACATAACGAAATGAAGACAGCAATAAAGATGTTCTTTGAAACCAACGAGAACAAAGACACAACATACCAGAATCTCTGGGATGCATTCAAAGCAGTGTGTAGAGGGAAATTTATAGCACGAAATGCCCACAAGAGAAAGCAGGAAAGATCCAAAATTGACACCCTAACATCACAATTAAAAGAACTAGAAAAGCAAGAGCAAACACATTCAAAAGCTAGCAGAAGGCAAGAAATAACTAAAATCAGAGCAGAACTGAAGGAAATAGAGACACAAAAAACCCTTCAAAAAATCAATGAATCCAGGAGCTGGTTTTTTGAAAGGATCAACAAAATTGATAGACCGCTAGCAAGACTAATAAAGAAAAAAAGAGAGAAGATTCAAATAGACGCAATAAAAAATGATAAAGGGGATATCACCACCGATCCCACAGAAATACAAACTACCATCAGAGAATACTACAAACACCTCTACGCAAATAAACTAGAAAATCTAGAAGAAATGGATACATTCCTCCACACATACACTCTCCCAAGACTAAACCAGGAAGAAGTAGAATCTGTGAATAGACCAATAACAGGAGCTGAAATTGTGGCAATAATCAATAGCTTACCAACCAAAAAGAGTCCAGGACCAGATGGATTCACAGCCGAATTCTACCAGAGGTACAAGGAGGAACTGGTACCATTCCTTCTGAAACTATTCCAATCAATAGAAAAAGAGGGAATCCTCCCTAACTCATTTTATGAGGCCAGCATCATTCTGATACCAAAGCCTGGCAGAGACACAACCAAAAAAGAGAATTTTAGACCAATATCCTTGATGAACATTGATGCAAAAATCCTCATTAAAATACTGGCAAACCGAATCCAGCAGCACATCAAAAATCTTATCCACCATGATCAAGTGGGCTTCATCCCTGGGATGCAAGGCTGGCTCAATATACGCAAATCAATGAATGTAATCCAGCATATAAACAGAGCCAAAGACAAAAACCACATGATTATCTCAATAGATGCAGAAAAAGCCTTTGACAAAATTCAACAACCCTTCATGCTAAAAACTCTCAATAAATTAGGTATTGATGGGACGTATTTCAAAATAATAAGAGCTATCTATGACAAACCCACAGCCAATATCATACTGAATGGGCAAAAACTGGAAGCATTCCCTTTGAAAACTGGCACAAGACAGGGATGCCCTCTCTCACCACTCCTATTCAACATAGTGTTGGAAGTTCTGGCCAGGGCAATCAGGCAGGAGAAGGAAATAAAGGGTATTCAATTAGGAAAAGAGGAAGTCAAATTGTCCCTGTTTGCAGACGACATGATTGTTTATCTAGAAAACCCCATTGTCTCAGCCCAAAATCTCCTTAAGCTGATAAGCAACTTCAGCAAAGTCTCAGGATACAAAATCAATGTACAAAAATCACAAGCATTCCTATACACCAACAACAGACAAACAGAGAGCCAAATCATGAGTGAACTCCCATTCACTATTGCTTCAAAGAGAATAAAATACCTAGGAATCCAACTTACAAGGGATGTGAAGGACCTCTTCAAGGAGAACTACAAACCACTGCTCAATGAAATAAAAGAGGATACAAACAAATGGAAGAACATTCCATGCTCATGGGTAGGAAGAATCAATATCGTGAAAATGGCCATACTGCCCAAGGTAATTTACAGATTCAATGCCATCCCCATCAAGCTACCAATGACTTTCTTCACAGAATTGGAAAAAACTACTTTAAAGTTCATATGGAACCAAAAAAGAGCCCGCATCGCCAAGTCAATCCTAAGCCAAAAGAACAAAGCTGGAGGCATCACACTACCTGACTTCAAACTATACTACAAGGCTACAGTAACCAAAACAGCATGGTACTGGTACCAAAACAGAGATATAGATCAATGGAACAGAACAGAGCCCTCAGAAATAATGCCCCATACCTACAACTATCTGATCTTTGACAAACCTGAGAAAAACAAGTAATGGGGAAAGGATTCCCTATTTAATAAATGGTGCTGGGAAAACTGGCTAGCCATATGTAGAAAGCTGAAACTGGATCCCTTCCTTACACCTTATACAAAAATCAATTCAAGATGGATTAAAGATTTAAACGTTAGACCTAAAACCATAAGAACCCTAGAAGAAAACCTAGGCATTACCATTCAGGACATAGGCGTGGGCAAGGACTTCATGTCCAAAACACCAAAAGCAATGGCAACAAAAGCCAAAATTGACAAATGGGATCTAATTAAACTAAAGAGCTTCTGCACAGCAAAAGAAACTACCATCAGAGTGAACAGGCAACCTACAACATGGGAGAAAATTTTCGCAACCTACTCATCTGACAAAGGGCTAATATCCAGAATCTACAATGAACTCAAACAAATTTACAAGAAAAAAACAAACAACCCCATCAAAAAGTGGGCGAGGGACATGAACAGACACTTCTCAAAAGAAGACATTTATGCAGCCAAAAAACACATGAAAAAATGCTCATCATCACTGGCCATCAGAGAAATGCAAATCAAAACCACTATGAGATATCATCTCACACCAGTTAGAATGGCAATCATTTAAAAAGTCAGGAAACAACAGGTGCTGGAGAGGATGTGGAGAAATAGGAACACTTTTACACTGTTGGTGGGACTGTAAACTAGTTCAACCATTGTGGAAGTCAGTGTGGCGATTCCTCAGGGATCTACAACTAGAAATACCATTTGACCCAGCCATCCCATTATTGGGTATATACCCAAATGACTATAAATCATGCTGCTATAAAGACACATGCACATGTATGTTTATTGCAGCATTATTCACAATAGCAAAGACTTGGAACCAACCCAAATGTCCAACAATGATAGACTGGATTAAGAAAATGTGGCACATATACACCATGGAATACTATGCAGCCATAAAAAATGATGAGTTCATGTCCTTTGTAGGGACATGGATGAAATTGGAAACCATCATTCTCAGTAAACTATCGCAAGAACAAAAAACCAAACACCGCATATTCTCACTCATAGGTGGGAATTGAACAATGAGATCACATGGACACAGGAAGGGGAATATCACACTCTGGGGACTGTGGTGGGGAGGGGGGAGGGGGGAGGGATAGCATTGGGAGATATACCTAATGCTAGATGACGAGTTAGTGGGTGCAGCGCACCAGCATGGCACATGTATACATATGTAACCTCCACAATGTGCACAGGTACCCTAAAACTTAAAGTATAATT*AAAATAATAATAATAATAATAGTAATAATAATAATAAA****AAA*GAAGGCAGAGATC**TTGAACTCCTGACCTCAAGTGATCCACTTGCCTCGGTCTCCCAAAGTCCTTGCA

**Clone 78; PA2; SpIRE(97/622)-78: Filled site spans: chr10:7137355-7143069**

Empty Site:

TATTTTTTAATCTATCAGTTTCCACTCCAGCCCTGCTGCCATCCCTATTCATCTGTACAGATCCCTATGTGAGATTTCATCAATGCACTTTCCCAGGCGCATTGCATTTGTGCATGCATA

CATCACCCTCCATT**AAAATGATCACTGAATC**CCAACCATG

AGCTTCCCTCTTGTTCACATTCACCTGTGCTCACCCTTCCTCCTTGCCTGCTCAGCATGCCCTATCTCTTGCATTCCAACCTTCAACCTGGTATCCACCTTCTCAAATCACCTTCTTGAC

Filled Site:

CATCAATGCACTTTCCCAGGCGCATTGCATTTGTGCATGCATACATCACCCTCCATT**AAAATGATCACTGAATC**ACAAAAATTCTTTATGGCGTCTTGATGGCCGAATAGGAACAGCTCCGGTCTACAGCTCCGGTCTACAGCTCCCAGCGTGAGCGACGCAGAAGACGGGTGATTTCTGCATTTCCAGCTGAGCTTTGAAGAGAGCAGTGGTTCTCCCAGCACGCAGCTGGAGATCTGAGAACGGGCAGACTGCCTCCTCAAGTGGGTCCCTGACCCCTGACCCCCGAGCAGCCTAACTGGGAGGCACCCCCCAGCAGGGGCACACTGACACCTCACACAGCAGGGTATTCCAACAGACCTGCAGCTGAGGGTCCTGTCTGTTAGAAGGAAAACTAACAAACAGAAAGGACATCCACACCAAAAACCCATCTGTACATCACCATCATCAAAGACCAAAAGTAGATAAAACCACAAAGATGAGGAAAAAACAGAAAAGAAAAACTGGAAACTCTAAAAAGCAGAGTGCCTCTCCTCCTCCAAAGGAACGCAGTTCCTCACCAGCAACGGGACAAAGCTGGATGAAGAATGACTTTGATGAGCTGAGAGAAGAAGGCGTCAGACGATCAAATTACTCTGAGCTACGGGAGGACATTCAAACCAAAGGCAAAGAAGTTGAAAACTTTGAAAAAAATTTAGAAGAATGTATAACTAGAATAACTAATACAGAGAAGTGCTTAAAGGAGCTGATGGAGCTGAAAACCAAGGCTCGAGAACTACGTGAAGAATGCAGAAGCCTCAGGAGCCGATGCGATCAACTGGAAGAAAGGGTATCAGCAATGGAAGATGAAATGAATGAAATGAAGTGAGAAGGGAAGTTTAGAGAAAAAGGAATAAAAAGAAATGAGCAAAGCCTCCAAGAAATATGGGACTGTGTGAAAAGACCAAATCTACGTCTGATTGGTGTACCTGAAAGTGATGGGGAGGATGGAACCAAGTTGGAAAACACTCTGCAGGATATTATCCAGGAGAACTTCCCCAATCTAGCAAGGCAGGCCAACGTTCAGATTCAGGAAATACAGAGAACGCCACAAAGATACTCCTCGAGAAGAGCAACTCCAAGACACATAATTGTCAGATTCACCAAAGTTGAAATGAAGGAAAAAATGTTAAGGGCAGCCAGAGAGAAAGGTCGGGTTACTCTCAAAGGGAAGCCCATCAGACTAACAGCGGATCTCTCGGCAGAAACCCTACAAGCCAGAAGAGAGTGGGGGCCAATATTCAACATTCTTAAAGAAAAGAATTTTCAACCCAGAATTTCATATCCAGCCAAACTAAGCTTCATAAGTGAAGGAGAAATAAAATACTTTACAGACAAGCAAATGCTGAGAGATTTTGTCACCACCAGGCCTGCCTTACAAGAGCTCCTGAAGGAAGCACTAAACATGGAAAGGAACAACCGGTACCAGCCGCTGCAAAATCATGCCAAAATGTAAAGACCATCAAGACTAGGAAGAAACTGCATCAACTAACGAGCAAAATCACCAGCTAACATCATAATGACTGGATCAAATTCACACATAACAATATTAACTTTAAATGTAAATGGACTTAATACTCCAATTAAAAGACACAGACTGGCAAATTGGATAAAGAGTCAAGACCCATCAGTGTGCTGTATTCAGGAAACCCATCTCACGTGCAGAGACACACATAGGCTCAAAATAAAAGGATGGAGGAAGATCTACCAAGCCAATGGGAAACAAAAAAAGGCAGGGGTTGCAATCCTAGTCTCTGATAAAAGACTTTAAACCAACAAAGATCAAAAGAGACAAAGAAGGCCATTACATAATGGTAAAGGGATCAATTCAACAAGAAGAGCTAACTATCCTAAATATATATGCACCCAATACAAGAGCACCCAGATTCATAAAGCAAGTCATGAATGACCTACAAAGAGACTTAGACTCCCACACATTAATAATGGGAGACTTTAACACCCCACTGTCAACATTAAACAGATCAACGAGACAGAAAGTCAACAAGGATACCCAGGAATTGAACTCAGCTCTGCACCAAGCAGACCTAATAGACATCTACAGAACTCTCCACCCCAAATCAACAGAATATACATTTTTTTCAGCACCACACCACACCTATTCCAAAATTGACCACATACTGGGAAGTAAAGCTCTCCTCAGCAAATGTAAAAGAACAGAAATTATAACAAACTATCTCTCAGACCACAGTGCAATCAAACTAGAACTCAGGATTAAGAATCTCACTCAAAACCGCTCAACTACATGGAAACTGAACAACCTGCTCCTGAATGACTACTGGGTACATAACGAAATGAAGACAGCAATAAAGATGTTCTTTGAAACCAACGAGAACAAAGACACAACATACCAGAATCTCTGGGACACATTCAAAGCAGTGTGTAGAGGGAAATTTATAGCACGAAATGCCCACAAGAGAAAGCAGGAAACATCCAAAATTGACACCCTAACATCACAATTAAAAGAACTAGAAAAGCAAGAGCAAACACATTCAAAAGCTAGCAGAAGGCAAGAAATAACTAAAATCAGAGCAGAACTGAAGGAAATAGAGACACAAAAAACCCTTCAAAAAATTAATGAATCCAGGAGCTGGTTTTTTGAAAGGATCAACAAAATTGATAGACCGCTAGCAAGACTAACAAAGAAAAAAAGAGAGATGAATCAAATAGATGCAATAAAAAATGATAAAGGGGATATCACCACCGATCCCACAGAAATACAAACTACCATCAGAGAATACTACAAACACCTCTACGCAAATAAACTAGAAAATCTAGAAGAAATGGATAAATTCCTGGACACATACGCTCTCCCAAGACTAAACCAGGAAGAAGTTGAATCTCTGAATAGACCAATAACAGGAGCTGAAATTGTGGCAATAATCAATAGCTTACCAACCAAAAAGAGTCCAGGACCAGATGGATTCACAGCCGAATTCTACCAGAGGTACAAGGAGGAACTGGTACCGTTCCTTCTGAAACTATTCCAATCAATAGAAAAAGAGGGAATCCTCCCTAACTCATTTTATGAGGCCAGCATCATTCTGATACCAAAGCCTGGCAGACACACAACCAAAAAAGAGAATTTTAGACCAATATCCTTGATGAACATTGATGCAAAAATCCTCAATAAAATACTGGCAAAACGAATCCAGCAGCACATCAAAAAGCTTATCCACCATGATCAAGTGGGCGTCATCCCTGGGATGCAAGGCTGGTTCAATATACGCAAATCAATAAATGTAATCCAGCATATAAACAGAGCCAAAGACAAAAACCACATGATTATCTCAATAGATGCAGAAAAAGCCTTTGACAAAATTCAACAACCCTTCATGCTAAAAACTCTCAATAAATTAGGTATTGATGGGACGTATTTCAAAATAATAAGAGCTATCTATGACAAACCCACAGCCAATATCATACGGAATGGGCAAAAACTGGAAGCATTCCCTTTGAAAACTGGCACAAGACAGGGATGCCCTCTCTCACCACTCCTATTCAACATAGTGTTGGAAGTTCTGGCCAGGGCAATTAGGCAGGAGAAGGAAATAAAGGGTATTCAGTTAGGAAAAGAGGAAGTCAAATTGTCCCTGTTTGCAGATGACATGATTGTATATCTAGAAAACCCCATTGTCTCAGCCCAAAATCTCCTTAAGCTGATAAGTAACTTCAGCAAAGTCTCAGGATACAAAATCAATGTACAAAAATCACAAGCATTCTTAGACACCAATGACAGACAAACAGAGAGCCAAATCATGAGTGAACTCCCATTCACTATTGCTTCAAAGAGAATAAAATACCTAGGAATCCAACTTACAAGGGATGTGAAGGACCTGTTCAAGGAGAACTACAAACCACTGCTCAAGGAAATAAAAGAGGATACAAACAAATGGAAGAACATTTCATGCTCATGGGTAGGAAGAATCAATATCATGAAAATGGCCATACTGCCCAAGGTAATTTACAGATTCAATGCCATCCCCATCAAGCTACCAATGACTTTCTTCACAGAATTGGAAAAAACTACTTTAAAGTTCATATGGAACCAAAAAAGAGCCCGCATCACCAAGTCAATCCTAAGCCAAAAGAACAAAGCTGGAGGCATCACACTACCTGACTTCAAACTATACTACAAGGCTACAGTAACCAAAACAGCATGGTACTGGTACCAAAACAGAGATATAGATCAATGGAACAGAACAGAGCCCTCAGAAATAACGCCGCATATCTACAACTATCTGATCTTTGACAAACCTGAGAAAAACAAGCAATGGGGAAAGGATTCCCTATTTAATAAATGGTGCTGGGAAAACTGGCTAGCCATATGTAGAAAGCTGAAACTGGATCCCTTCCTTACACCTTATACAAAAATCAATTCAAGATGGATTAAAGACTTAAACGTTAGACCTAAAACCATTAAAACCCTAGAAGAAAACCTAGGCATTACCATTCAGGACATAGGCATGGGCAAGGACTTCATGTCTAAAACACCAAAAGCAATGGCAACCAAAGACAAAATTGACAAATGGGATCTAATTAAACTAAAGAGCTTCTGCACAGCAAAAGAAACTACCATCAGAGTGAACAGGCAACCTACAAAATGGGAGAAAATTTTGGCAACCTATTCATCTGACAAAGGGCTAATATCCAGAATCTACAACGAACTCAAACAAATTTACAAGAAAAAAACAAACAACCCCATCAAAAAGTGGGCAAAGGACATGAACAGACACTTCTCAAAAGATGACATTTATGCAGCCAAAAAACACATGAAAAAATGCTCATCATCACTGGCCATCAGAGAAATGCAAATCAAAACCACAATGAGATATCATCTCACACCAGTTAGAATGGCAATCATTAAAAAGTCAGGAAACAACAGGTGCTGGAGAGGATGTGGAAAAATAGGAACACTTTTACACTGTTGGTGGGACTGTAAACTAGTTCAACCATTGTGGAAGTCAGTGTGGCGATTCCTCAGGGATCTAGAACTAGAAATACCATTTGACCCAGCCATCCCATTACTGGGTATATACCCAAAGGACTATAAATCATGCTGCTATAAAGAAACATGCACACGTATGTTTATTGCGGCATTATTCACAATAGCAAAGACTTGGAACCAACCCCAATGTCCAACAATGATAGACTGGATTAAGAAAATGTGGCACATATACACCATGGAATACTATGCAGCCATAAAAAATGATGAGTTCATGTCCTTTGTAGGGACATGGATGAAATTGGACATCATCATTCTCAGTAAACTATTGCAAGAACAAAAAACCAAACACCACATATTCTCACTCATAGGTGGGAATTGAACAATGAGATCACATGGACACAGGAAGGGGAATATCACACTCTGGGGACTGTGGTGGGGTGGGGGGAGGGGGGAGGGATAGCATCGGGAGATATACCTAATGCTAGATGACGAGTTAGTGGGTGCAGCACACCAGCATGGCACATGTATACATATGTAAGTAACCTGCACAATGTGCACATGTACCCTAAAACTTAAAGTATAAT*AAAGAAAAAAAAAGAAAAA****AAAA*TGATCACTGAATC**CCAACCATGAGCTTCCCTCTTGTTCACATTCACCTGTGCTCACCCTTCCTCCTTGCCTGCTCAGCATGCCCTATCTCTTGCATTCCAACC

**Clone 79; PA2; SpIRE(97/622)-79: Filled site spans: chr10:44482732-44488399**

Filled Site:

CCGGTTCCTCACCCTCGGGGAGCAACATGGATAATCTCAGTGATACCTTGAAGAAGCTGAAGATAACAGCTGTTGACAAGACCGAGGATAGTTTAGAAGGATGCTTGGATTGTCTGCTTC

AAGCCCTGGCTCAAAAT**AAATGATTCAGTGTAG**TTGTAGT

GCTAGATATATTATATGGAATTATCAGCAGTTTACCAGTTGGTTCTTTATTAGTACTCCTGATGTCTTAAACAAAAATGAATAATTGTTTAAAAAAAAAAAGAAAGTC

Filled Site:

TTGACAAGACCGAGGATAGTTTAGAAGGATGCTTGGATTGTCTGCTTCAAGCCCTGGCTCAAAAT**AAATGATTCAGTGTAG**GGGGAGGAGCTAAGATGGCCGAATAGGAACAGCTCCGGTCTACAGCTCCCAGCGTGAGCCACGCAGAAGACGGGTGATTTCTGCATTTCCATCTGAGCTTTGAAGAGAGCAATGCTTCTCCCAGCACGCAGCTGGAGATCTGAGAACGGGCAGACTGCCTCCTCAAGTGGATCCCTGACCCCTGACCCCCGAGCAGCCTAACTGGGAGGCACCACCCAGCAGGGGCACACTGACACCTCACACGGCAGGGTATTCCAACAGACCTGCAGCTGAGGGTCCTGTCTGTTAGAAGGAAAACTAACAAACAGAAAGGACATCCACACCGAAAACCCATCTGTACATCACCATCATCAAAGACCAAAAGTAGACAAAACCACAAAGATGGGGAAAAAACAGAACAGAAAAACTGGAAACTCTAAAACGCAGAGCACCTCTCCTCCTCCAAAGGAACGCAGTTCCTCACCAGCAACGGAACAAAGCTGGATGGAGAATGATGTTGACGAGCTGAGAGAAGAAGGCTTCAGACGATCAAATTACTCTGAGCTACAGGAGGACATTCAAACCAAAGGCAAAGAAGTTGAAAACTTTGAGAAAAATTTAGAAGAATGTATAACTAGAATAACCAATACAGAGAAGTGCTTAAAGGAGCTGATGGAGCTGAAAACCAAGGCTCGAGAACTACGTGAAGAATGCAGAAGCCTCAGGAGCCGATGTGATCAACTGGAAGAAAGGGTATCAGTGATGGAAGATGAAATGAATGAAATGAAGTGAGAAGGGAAGTTTGGAGAAAAAAGAATAAAAAGAAATGAGCAAAGCCTCCAAGAAATATGGGACTATGTGAAAAGACCAAATCTACGTCTGATTGGTGTACCTGAAAGTGAGGCAGAGAATGGAACCAAGTTGGAAAACACTCTGCAGGATATTATCCAGGAGAACTTCCCCAATCTAGCAAGGCAGGCCAACGTTCAGATTCAGGAAATACAGAGAATGCCACAAAGATACTCCTCAAGAAGAGCAACTCCAAGACACATAATTGTCAGATTCACCAAAGTTGAAATGAAGGAAAAAATGTTAAGGGCAGCCAGAGAGAAAGGTCGGGTTACCTACAAAGGGAAGCCCATCAGACTAACAGCAGATCTCTTGGCAGAAACCCTACAAGCCAGAAGAGAGTGGGGGCCAATATTCAACATTCTTAAAGAAAAGAATTTTCAACACAGAATTTCATATCCAGCCAAACTAAGCTTCATAAGTGAAGGAGAAATAAAATACTTTACAGACAAGCAAATGCTGAGAGATTTTGTCACCACCAGGCCTGCCCTAAAAGAGCCCTGAAGGAAGCGCTAAACATGGAAAGGAACAACCGGTACCAGCCGCTGCCAAATCATGCCAAAATGTAAAGACCATCGAGACTAGGAAGAAACTGCATCAACTAACGAGCAAAATAACCAGCTAACATCATAATGACAGGATCAAATTCACACATAACAATATTAACTTTAAATGTAAATGGACTAAATTCTCCAATTAAAAGACACAGACTGGCAAGTTGGATAACGAGTCAAGATCCATCAGTGTGCTGTATTCAGGAAACCCATCTCACGTGCAGAGACACACATAGGTTCAAAATAAAAGGATGGAGGAAGATCTACCAAGCAAATGGAAAACAAAAAAAGGCAGGGGTTGCAATCCTAGTCTCTGATAAAACAGACTTTAAACCAACAAAGATCAAAAGAGACAAAGAAGGCCATTACATAATGGTAAAGGGATCAATTCAACAAGAGGAGCTAACTATCCTAAATATATATGCACCCAATACAGGAGCACCCAGGTTCATAAAGCAAGTCCTGAGTGACCTACAAAGAGACTTAGACTCCCACACATTAATAATGGGAGACTTTAACACCCCACTGTCAACATTAGACAGATCAACGAGACAGAAAGTCAACAAGGATACCCAGGAATTGAACTCAGCTCTGCACCAAGCATACCTAATAGACATCTACAGAACTCTCCACCCCAAATCAACAGAATATACATTTTTTTCAGCACCACACCACACCTATTCCAAAATTGACCACATAGTTGGAAGTAAAGCTCTCCTCAGCAAATGTAAAAGAACAGAAATTATAACAAACTATCTCTCAGACCACAGTGCAATCAAACTAGAACTCAGGATTAAGAATCTCACTCAAAGCCGCTCAACTACATGGAAACTGAACAACCTGCTGCTGAATGACTACTGGGTACATAATGAAATGAAGGCAGAAATAAAGATGTTCTTTGAAACCAACGAGAACAAAGACACAACATACCAGAATCTCTGGGACGCATACAAAGCAGTGTGTAGAGGGAAATTTATAGCACTAAATGCCCACAAAAGAAAGCAGGAAAGATCCAAAATTGACACCCTAACATCACAAATAAAAGAACTAGAAAAGCAAGAGCAAACACATTCAAAAGCTAGCAGAAGGCAAGAAATAACTAAAATCAGAGCAGAACTGAAGGAAATAGAGACACAAAAAACCCTTCAAAAAATCAATGAATCCAGGAGCTGGTTTTTTGAAAGGATCAACAAAATTGATAGACCGCTAGCAAGACTAATAAAGAAAAAAAGAGAGAAGAATCAAGTAGACACAATAAATAATGATAAAGGGGATATCACCACTGATCCCACAGAAATACAAACTACCATCAGAGAATACTACAAACACCTCTACGCAAATAAACTAGAAAATCTAGAAGAAATGGATACATTCCTCATCACATACACTCTCCCAAGACTAAACCAGGAAGAAGTTGAATCTCTGAATAGACCAATAACAGGAGATGAAATTGTGGCAATAATCAATAGTTTACCAACCAAAAAGAGTCCAGGACCAGATGGATTCACAGCCAAATTCTACCAGAGGTACAAGGAGGAACCGATACCATTCCTTCTGAAACTATTCCAATCAATAGAAAAAGAGGGAATCCTCCCTAACTCATTTTATGAGGCCAGCATCATTCTGATACCAAAGCCGGGCAGAGACACAACCAAAAAAGAGAATTTTAGACCAATATCCTTGATGAACATTGATGCAAAAATCCTCAATAAAATACTGGCAAACCGAATCCTGCAGCACATCAAAAAGCTTATCCACCATGATCAAGTGGGCTTCATCCCTGGGATGCAAGGCTGGTTCAATATATGCAAATCAATAAATGTAATCCAGCATATAAACAGAACCAAAGACAAAAACCACATGATTATCTCAATAGATGCAGAAAAAGCCTTTGACAAAATTCAACAACCCTTCATGCTAAAAACTCTCAATAAATTAGGTATTGATGGGAGGTATTTCAAAATAATAAGAGCTATCTATGACAAACCCACAGCCAATATCATACTGAATGGGCAAAAACTGGAAGCATTCCCTTTGAAAACTGGCACAAGACAGGGATGCCCTCTCTCACCACTCCTATTCAACATAGTGTTGGAAGTTCTGGCCAGGGCAATTAGGCAGGAGAAGGAAATAAAGGGTATTCAATTAGGAAAAGAGGAAGTCAAATTGTCCCTGTTTGCAGACGACATGATTGTATATCTAGAAAACCCCATCATCTCAGCCCAAAATCTCCTTAAGCAGATAAGCAACTTCAGCACAGTCTCAGGATACAAAATCAATGTACAAAAATCACAAGCATTCTTATACACCAACAACAGACAAACAGAGAGCCAAATCATGAGTGAACTCCCATTCACAATTGCTTCAAAGAGAATAAAATACCTAGGAATCCAACTTACAAGGGATGTGAAGGACCTCTTCAAGGAGAACTACAAACCACTGCTCAAGGAAATAAAAGAGGATACAAACAAATGGAAGAACATTCCATGCTCATGGGTAGGAAGAATCAATATTGTGAAAATGGCCACACTGCCCAAGGTAATTTACAGATTCAATGCCATCCCCATCAAGCTACCAATGACTTTCTTCACAGAATTGGAAAAAACTACTTTAAAGTTCATATGGAACCAAAAAAGAGCCCGCATCGCCAAGTCAATCCTAAGCCAAAAGAACAAAGCTGGAGGCATCACACTACCTGACTTCAAACTATACTACAAGGTTACAGTAACCAAAACAGCATAGTACTGGTACCAAAACAGAGATATAGATCAATGGAACAGAACAGAGCCCTCAGAAATAACACCGCATACCTACAACTATCTGAACTTTGACAAACCTGAGAAAAACAAGCAATGGGGAAAGGATTCCCTATTTAATAAATGGTGCTGGGAAAACTGGCTAGCCATATGTAGAAAGCTGAAACTGGATCCCTTCCTTACACCTTATACAAAAATCAATTCAAGATGGATTAAAGATTTAAACGTTAGACCTAAAACCATAAAAACCCTAGAAGAAAACCTAGGCATTACCATTCAGGACATAAGCATGGGCAAGGACTTCATGTCCAAAACACCAAAAGCAATGGCAACAAAAGCCAAAATTGACAAATGGGATCTCATAAAACTAAAGAGCTTCTGCACAGCAAAAGAAACTACCATCAGAGTGAACAGGCAACCTACAACATGGGAGAAAATTTTCGCAACCTACTCATCTGACAAAGGGCTAATATCCAGAATCTACAATGAACTCAAACAAATTTACAAGAAAAAGACAAACAACCCCATCAAAAAGTGGGCGAAGGACATGAACAGTCACTTCTCAAAAGAAGACATTTATGCAGCCAAAAAACACATGAAAAAATGCCCATCATCACTGGCCATCAGAGAAATGCAAATCAAAACCACTATGAGATATCATCTCACACCAATTAGAATGGCAATCATTAAAAAGTCAGGAAACAACAGGTGCTGGAGAGGATGTGGAGAAATAGGAACATTTTTACACTGTTGGTGGGACTGTAAACTAGTTCAACCATTGTGGAAGTCAGTGTGGCGATTCCTCAGGGATCTAGAACTAGAAATACCATTTGACCCAGCCATCCCATTACTGGGTATATACCCAAATGACTATAAATCATGCTGCTATAAAGACACATGCACTCGTATGTTTATTGCGGCATTATTCACAATAGCAAAGACTTGGAACCAACCCAAATGTCCAACAATGATAGACTGGATTAAGAAAATGTGGCACATATACACCATGGAATACTATGCAGCCATAAAAAATGATGAGTTCATGTCCTTTGTAGGGACATGGATGAAATTGGAAACCATCATTCTCAGTAAACTATCGCAAGAACAGAAAACCAAACACCGCATATTCTCACTCATAGGTGGGAATTGAACAATGAGATCACATGGACACAGGAAGGGGAATATCACACTCTGGGGACTGTGGTGGGGTGGGGGGAGGGGGGAGGGATAGCATTGGGAGATATACCTAATGCTAGATGACGAGTTAGTGGGTGCAGCGCACCAGCATGGCACATGTATACATATGTAACTAACCTGCACAATGTGCACATGTACCCTAAAACTTAAAGTAT*AAAAAAA****AA*ATGATTCAGTGTAG**TTGTAGTGCTAGATATATTATATGGAATTATCAGCAGTTTACCAGTTGGTTCTTTATTAGTACTCCTGATGTCT

**Clone 105; PA2; SpIRE(97/622)-105: Filled site spans: chr15:51173421-51179122**

Empty Site:

AAGGGAGCCATGTGACGGGGAAGTAGAAAACCATTTTATTTCACATTTAGCATTTTCTCTGTGCAAACAGCTCTCTTAGCAGCAAACTCCAGAGGGTAGAATCTGGGCATTCATCCTAAA

CTCTCAACAGACCTTTGAGTT**AATATTT**TATCAAGGCACA

TGTTGGATATTTGCCCAATATAAGTTGTTGGTCTTTTCATCAGATCACTGTCTGAAAACACCAGCTCTCTTGCGCCTTCTCTTGTTTATGTACACGTTGGCTCTAGCGTCACTGTTACAA

Filled Site

AGCTCTCTTAGCAGCAAACTCCAGAGGGTAGAATCTGGGCATTCATCCTAAACTCTCAACAGACCTTTGAGTT**AATATTT**GGGGGAGGAGCCAAGATGGCCGAATAGGAACAGCTCCGGTCTACAGCTCCCAGCGTGAGCGACGCAGAAGACGGGTGATTTCTGCATTTCCATCTGAGCTTTGAAGAGAGCAGTGGTTCTCCCAGCACGCAGCTGGAGATCTGAGAACGGGCAGACTGCCTCCTCAAGTGGGTCCCTGACCCCTGACCCCCGAGCAGCCTAACTGGGAGGCACCCCCCAGCAGGGGCACACTGACACCTCACACGGCAGGGTATTCCAACAGACCTGCAGCTGAGGGTCCTGTCTGTTAGAAGGAAAACTAACAAACAGAAAGGACATCCACACCGAGAACCCATCTGTACATCACCATCATCAAAGACCAAAAGTAGATAAAACCACAAAGATGGGGAAAAAACAGAACAGAAAAACTGGAAACTCTAAAACGCAGAGCGCCTCTCCTCCTCCAAAGGAACGCAGTTCCTTACCAGCAACGGAACAAAGCTGGATGGAGAATGATTTTGACGAGCTGAGAGAGGAAGGCTTCAGACGATCAAATTATTCTGAGCTACGGGAGGACATTCAAACCAAAGGCAAAGAAGTTGAAAACTTTGAAAAAAATTTAGAAGAATGTATAACTAGAATAACCAATACAGAGAAGTGCTTAAAGGAGCTGATGGAGCTGAAAACCAAGGCTCGAGAACTACGTGAAGAATGCAGAAGCCTCAGGAGCTGATGCGATCAACTGGAAGAAAGGGTATCAGCAATGGAAGATGAAATGAATGAAATGAAGCAAGAAGGGAAGTTTAGAGAAAAAAGAATAAAAAGAAATGAGCAAAGCCTCCAAGAAATATGGGACTATGTGAAAAGACCAAATCTACGTCTGATTGGTGTACCTGAAAGTGATGGGGAGAATGCAACCAAGTTGGGAAACACTCTGCAGGATATTATCCAGGAGAACTTCCCCAATCTAGCAAGGCAGGCCAACAACGTTCAGATTCAGGAAATACAGAGAACGCCACAAAGATACTCCTCGAGAAGAGCAACTCCAAGACACATAATTGTCAGATTCACCAAAGTTGAAATGAAGGAAAAAATGTTAAGGGCAGCCAGAGAGAAAGGTCGAGTTACCCTCAAAGGGAAGCCCATCAGACTAACAGTGGATCTCTCGGCAGAAACCCTACAAGCCAGAAGAGAGTGGGGGCCAATATTCAACATTCTTAAAGGAAAGAATTTTCAGCCCAGAATTTCATATCCAGCCAAACTAAGCTTCATAAGTGAAGGAGAAATAAAATACTTTACAGACAAGCAAATGCTGAGAGATTTTGTCACCACCAGGCCTGCCCTAAAAGAGCTCCTGAAGGAAGCGCTAAACATGGAAAGGAACAACCAGTACCAGCCGCTGCAAAATCATGCCAAAATGTAAAGACCATCGAGACTAGGAAGAAACTGCATCAACTAACGAGCAAAATCACCAGCTAACATCATAATGACAGGATCAAATTCACACATAACAATATTAACTTTAAATGTAAATGGACTAAATTCTCCAATTAAAAGACACAGACTGGCAAGTTGGATAAAGAGTCAAGACCCATCAGTGTGCTGTATTCAGGAAACCCATCTCACGTGCAGAGACACACATAGGCTCAAAATAAAAGGATGGAGGAAGATCTACCAAGCAAATGGAAAACAAAAAAAGGCAGGGGTTGCAATCCTAGTCTCTGATAAAACAGACTTTAAGCCAACAAAGATCAAAAGAGACAAAGAAGGCCATTACATAATGGTAAAGGGATCAATTCAACAAGAGGAGCTAACTATCCTAAATATATATGCACCCAATACAGGAGCACCCAGATTCATAAAGAAAGTCCTGAGTGACCTACAAAGAGACCTAGACTCCCACACATTAATAATGGGAGACTTTAACACCCCACTGTCAACATTAGACAGATCAACGAGACAGAAAGTCAACAAGGATACCCAGGAATTGAACTCAGCTCTGCACCAAGGGGACCTAATAGACATCTACAGAACTCTCCACCCCAAATCAACAGAATATACATTTTTTTCAGCACCACACCACACCTATTCCAAAATTGACCACATAGTTGGAAGTAAAGCTCTCCTCAGCAAATGGAAAAGAACAGAAATTATAACAAACTATCTCTCAGACCACAGCGCAATCAAACTAGAACTCAGGATTAAGAATCTCACTCAAAGCCGCTCAACTACATGGAAACTGAACAACCTGCTCCTGAATGACTACTGGGTACATAACGAAATGAAGGCAGAAATAAAGATGTTCTTTGAAACCAACGAGAACAAAGACACAACATACCAGAATCTCTGGGATGCATTCAAAGCAGTGTGTAGAGGGAAATTTATAGCACTAAATGCCCACAAGAGAAAGCAGGAAAGATCCAAAATTGACACCCTAACATCACAGTTAAAAGAACTAGAAAAGCAAGAGCAAACACATTCCAAAGCTAGCAGAAGGCAAGAAATAACTAAAATCAGAGCAGAACTGAAGGAAATAGAGACACAAAAAACCCTTCAAAAAATCAATGAATCCAGGAGCTGGTTTTTTGAAAGGATCAACAAAATTGATAGACCACTAGCAAGACTAATAAAGAAAAAAAGAGAGAAGAATCAAATAGACACAATAAAAAATGATAAAGGGGATATCACCACCGATCCCACAGAAATACAAACTACCATCAGAGAATACTACAAACACCTCTATTCAAATAAACTAGAAAATCTAGAAGAAATGGATAAATTCCTCGACACATACACTCTCCCAAGACTAAACCAGGAAGAAGTTGAATCTCTGAATAGACCAATAACAGGAGCTGAAATTGTGGCAATAATCAATAGTTTACCAACCAAAAAGATTCCAGGACCAGATGGATTCACAGCCGAATTCTACCAGAGGTACAAGGAGGAACTGGTACCATTCCTTCTGAAACTATTCCAATCAATAGAAAAAGAGGGAATCCTCCCTAACTCATTTTATGAGGCCAGCATCATTCTGATACCAAAGCCGGGCAGAGACACAACCAAAAAAGAGAATTTTAGACCAATATCCTTGATGAACATTGATGCAAAAATCCTCAATAAAATACTGGCAAAACGAATCCAGCAGCACATCAAAAAGCTTATCCACCATGATGAAGTGGGCTTCATCCCTGGGATGCAAGGCTGGTTCAATATACACAAATCAATAAATGTAATCCAGCATATAAACAGAGCCAAAGACAAAAACCACATGATTATCTCAATAGATGCAGAAAAAGCCTTTGACAAAATTCAACAACCCTTCATGCTAAAAACTCTCAATAAATTAGGTATTGATGGGATGTATTTCAAAATAATAAGAGCTATCTATGACAAACCCACAGCCAATATCATGCTGAATGGGCAAAAGCTGGAAGTATTCCCTTTGAAAAGCAGCACAAGACAAGGATGCCCTCTCTCACCACTCCTATTCAACATGGTACTGAATGTTCTGGCCAGGGCAATCAGGCAAGAGAAAGCAATAAAGGGTATTCAAATAGGAAGAGAGGAAGTCAAATTGTCCCTGTTTGCAGATGACGTGATTGTTTATCTAGAAAACCCCATCGTCTCAGCCCAAAATCTCCTTAAGCTAATAAGCAACTTCAGCAAAGTCTCAGGATACAAAATCAATGTACAAAAATCACAAGCATTCCTATACACCAACAACAGACAAACAGAGAGCCAAATCATGAGTGAACTCCCATTCACAATTGCTTCAAAGAGAATAAAATACCTAGGAATCCAACTTACAAGGGATGTGAAGGACCTCTTCAAGGAGAACTACAAACCACTGCTCAAGGAAATAAAAGAGGATACAAACAAATGGAAGAACATTCCATGCTCATGGGTAGGAAGAATCAATATCGTGAAAATGGCCATACTGCCCAAGGTAATTTATAGATTCAATGCCATCCCCATCAACCTACCAATGACTTTCTTCACAGAATTGGAAAAAACTACTTTAAAGTTCATATGGAACCAAAAAAGAGCCCGCATCGCCAAGTCAATCCTAAGCCAAAAGAACAAAGCTGGAGGCATCACACTACCTGACTTCAAGCTATACTACAAGGCTACAGTAACCAAAACAGCATGGTACTGGTACCAAAACAGAGATATAGATCAATGGAACAGAACAGAGCCCTCAGAAATAATGCTGCATACCTACAACTATCTGATCTTTGACAAACCTGAGAAAAACAAGCAATGGGGAAAGGATTCCCTATTTAATAAATGGTGCTGGGAAAACTGGCTAGCCATATGTAGAAAGCTGAAACTGGATCCCTTCCTTACACCTTATACAAAAATCAATTCAAGATGGATTAAAGATTTAAACGTTAGACCTAAAACCATAAAAACCCTAGAAGAAAACCTAGGCATTACCATTCAGGACATAGGCGTGGGCAAGGACTTCATGTCCAAAACTCCAAAAGCAATGGCAACCAAAGCCAAAATTGACAAATGGGATCTAATTAAACTAAAGAGCTTCTGCACAGCGGGGGAAACTACCATCAGAGTGAACAGGCAACCTACAACATGGGAGAAAATTTTCGCAACCTACTCATCTGACAAAGGGCTAATATCCAGAATCTACAATGAACTCAAACAAATTTACAAGAAAAAAACAAACAACCCCATCAAAAAGTGGGCGAGGGACATGAACAGACACTTCTCAAAAGAAGACATTTATGCAGCCAAAAAACACATGAAAAAATGCTCATCATCACTGGCCATCAGAGAAATGCAAATCAAAACCACTATGAGATACCATCTCACACCAGTTAGAATGGCAATCATTGAAAAGTCAGGAAACAACAGGTGCTGGAGAGGATGTGGAGAAATAGGAACACTTTTACACTGTTGGTGGGACTGTAAACTAGTTCAACCATTGTGGAAGTCAGTGTGGCGATTCCTCAGGGATCTAGAACTAGAAATACCATTTGACCCAGCCATCCCATTACTGGGTATATACCCAAAGGACTATAAATCATGCTGCTATAAAGACACATGCACACGTATGTTTATTGCGGCATTATTCACAATAGCAAAGACTTGGAACCAACCCAAATGTCCAACAATGATAGACTGGATCAAGAAAATGTGGCACATATACACCATGGAATACTATGCAGCCATAAAAAATGATGAGTTCATGTCCTTTGTAGGGACATGGATGAAATTGGAAACCATCATTCTCAGTAAACTATCGCAAGAACAAAAAACCAAATACCGCATATTCTCACTCATAGGTGGGAATTGAACAATGAGATCACATGGACACAGGAAGGGGAATATCACACTCTGGGGACTGTGGTGGGGAGGGGGGAGGGGGGAGGGATAGCATTGGGAGATATACCTAATGCTAGATGACGAGTTAGTGGGTGCAGCGCACCAGCATGGCACATGTATACATATGTAACTAACCTGCACAACGTGCACATGTACCCTAAAACTTAAAGTATAATT*AAAAAAAAAATAATAATAATAATA****AA*TATTT**TATCAAGGCACATGTTGGATATTTGCCCAATATAAGTTGTTGGTCTTTTCATCAGATCACTGTCTGAAAACACCAGCTCTCTTGCGCCTTCT

**Clone 1; PA3; SpIRE(97/622)-1: Filled site spans: chrX:101540790-101546459**

Empty Site:

ACAGAAAACCAAACACCGCATATTCTTACTCATAGGTGGGAATTGAACAATGAGAACACTTGGACACTGGAAGGGGAACATCACACACAGGGGCCTGTCGTCGGGTGGGGGGAGGGGGAG

GGGTAGCATT**AGGAGATATACCTAATGTA**AATGACGAGTT

AATAGGTGCAGCACACCAACATGGCGCATGTATACATATGTAACAAACCTGCACGTTGTGCACATGTACCCTAGAACTTAAAGTATAATAAAAAAAAGAAAAAAAGTTGGTAAATTTTGT

Filled Site:

TCACACACAGGGGCCTGTCGTCGGGTGGGGGGAGGGGGAGGGGTAGCATT**AGGAGATATACCTAATGTA**GGAGGGAGGAGCCAAGATGGCCGAATAGGAACAGCTCGGGTCTACAGCTCCCAGCGTGAGCGATGCAGAAGACGGGTGATTTCTGCATTTCCATCTGAGCTTTGAAGAGAGCAGTGGTTCTCCCAGTACGCAGCTGGAGATCTGAGAACGGGCAGACTGCCTCCTCAAGTGGGTCCCTGACCCCTGACCCCTGAGCAGCCTAACTGGGAGGCACCCCCCAGCAGGGGCACACTGACACCTCACACGGCAGGGTACTCCAACAGACCTGCAGCTGAGGCTCCTGTCTGTTAGAAGGAAAACTAACAAACAGAAAGGACATCCACACCAAAAACCCATCTGTACATCACCATCATCAAAGACCAAAAGTAGATAAAACCACAAAGATGGGGAAGAAACAGAACAGAAAAACTGGAAACTCTAAAAAGCAGAGCGCCTCTCCTTCTCCAAAGGAACGCAGTTCCTCACCAGCAACAGAACAAAGCTGGATGGAGAATGACTTTGACGAGCTGAGAGAAGAAGGCTTCGGACGATCAAATTACTCTGAGCTACGGGAGGACATTCAAACCAAAGGCAAAGAAGTTGAAAACTTTGAAAAAAATTTAGAAGAATGTATAACTAGAATAACCAATACAGAGAAGTGCTTAAAGGAGCTGATGGAGCTGAAAACCAAGGTTCGAGAACTACGTGAAGAATGCAGAAGCCTCAGGAGCCGATGCAATCAACTGGAAGAAAGGGTATCAGCAATGGAAGATGAAATGAATGAAATGAAGTGAGAAGGGAAGTTTAGAGAAAAAAGAATAAAAAGAAACGAGCAAAGCCTCCAAGAAATATGGGACTATGTGAAAAGACCAAATCTACGTCTGATTGGTGTACCTGAAAGTGATGGGGAGAATGGAATCAAGTTGGAAAACACTCTGCAGGATATTATCCAGGAGAACTTCCCCAATCTAGCAAGGCAGGCCAACATTCAGATTCAGGAAATACAGAGAACGCCACAAAGATACTCCTCGAGAAGAGCAACTCCAAGACACATAATTGTCAGATTCACCAAAGTTGAAATGAAGGAAAAAATGTTAAGGGCAGCCAGAGAGAAAGGTCGGGTTACCCTCAAAGGGAAGCCCATCAGACTAACAGCGAATCTCTCGGCAGAAACCCTACAAGCCAGAAGAGAGTAGGGGCCAATATTCGACATTCTTAAAGAAAAGAATTTTCAACCCAGAATTTCATATCCAGCCAAACTAAGCTTCATAAGTTAAGGAGAAATAAAATACTTTACAGACAAGCAAATGCTGAGAGATTTTGTCACCACCAGGCCTGCCCTAAAAGAGCTCCTGAAGGAAGCGCTAAACATGGAAAGGAACATCCGGTACCAGCCGCTGCAAAATCATGCCAAAATGTAAAGACCATTGAGACTAGGAAGAAACTGCATCAACTAACGAGCAAAATCACCAGCTAACATCATAATGACAGGATCAAATTCACACATAACAATATTAACTTTAAATGTAAATGGACTAAATGCTCCAATTAAAAGACACAGACTGGCAAATTGGATAAAGAGTCAAGACCCATCAGTGCGCTGTATTCAGGAAACCCATCTCACATGCAGAGACACACATAGGCTCAAAATAAAAGGATGGAGGAAGATCTACCAAGCAAATGGGAAACAAAAAAAGGCAGGGGTTGCAATCCTAGTCTCTGATAAAACAGACTTTAAACCAACAAAGATCAAAAGAGACAAAGAAGGCCATTACTTAATGGTAAAGGGATCAATTCAACAAGAAGAGCTAACTATCCTAAATATATATGCACCCAATTCAGGAGCACCCAGATTCATAAAGCAAGTCCTAAGTGACCTACAAAGAGACTTAGACTCCCACACATTAATAATGGGAGACTTTAACACCCTACTGTCAACATTAGACAGATCAACGAGACAGAAAGTCAACAAGGATACCCAGGAATTGAACTCAGCTCTGCACAAAGTGGACCTAATAGACAACTACAGAACTCTCCACCCCAAATCAACAGAATATACATTCTTTTCAGCACCACACCACACCTATTCAAAAATTGACCACATACTTGGAAGTAAAGCACTCCTCAGCAAATGTAAAAGAACAGAAATTATAACAAACTATCTCTCAGACCACAGTGCAATCAAACTAGAACTCAGGATTAAGAATCTCACTCAAAACCGCTCAACTACATGGAAACTGAACAACCTGCTCCTGGATGACTACTGGGTACATAACGAAATGAAGGCAGAAATAAAGATGTTCTTTGAAACCAGTGAGAACAAAGGCACAACATACCAGAATCTCTGGGACACATACAAAGCAGTGTGTAGAGGGAAATTTATAGCACTAAATGCCCACAAGAGAAAGCAGGAAAGATCCAAAATTGACACTTTAACATCACAATTAAAAGAACTAGAAAAGCAAGAGCAAACACATTCAAAAGCTAGCAGAAGGCAAGAAATAACTAAAATCAGAGCAGAACTGAAGGAAATAGAGACACAAAAAACCCTTCAAAAAATTAATGAATCCAGGAGCTGGTTTTTTGAAAGGATCAACAAAATAGATAGACCGCTAGCAAGACTAATAAAGAAAAACAGAGAGAAGAATCAAATAGATGCAATAAAAAATGATAAAGGGGATATCACCACCGATCCCACAGAAATACAAACTACCATCAGAGAATACTACAAACACCTCTACGCAAATAAACTAGAAAATCTAGAAGAAATGGATAAATTCCTCGACACATACACTCTCCCAAGACTAAACCAGGAAGAAGTGGAATCTCTGAATAGACCAATAACAGGATCTGAATTTGTGGCAATAATCAATAGCTTACCAACCAAAAAGAGTCCAGGACCAGATGGATTCACAGCCGAATTCTGCCAGAGGTACAAGGAGGAACTGGTACCATTCCTTCTGAAACTATTTCAATCAATAGAAAAAGAGGGAATCCTCCCTAACTCATTTTATGAGGCCAGCATCATTCTGATACCAAAGCCAGGCAGAGACACAACCAAAAAAGAGAATTTTAGACCAATATCCTTGATGAACATTGATGCAAAAATCCTCAGTAAAATACTGGCAAAACGAATCCAGCAGCACATCAAAAAGCTTATCCACCATGATCAAGTGGGCTTCATCCCTGGGATGCAAGGCTGGTTCAATATACACAAATCAATAAATGTAATCCAGCATATAAACAGAGCCAAAGACAAAAACCACATGATTATCTCAATAGATGCAGAAAAGGCCTTTGACAAAATTCAACAACCCTTCATGCTAAAAACTCTCAATAAATTAGGTATTGATGGGACATATTTCAAAATAATAGGAGCTATCTATGACGAACCCACAGCCAATATCATACTGAATGGGCAAAAACTGGAAGCATTCCCTTTGAAAACTGGCACAAGACAGGGATGCCCTCTCTCACCACTCCTACTCAACATAGTGTTGGAAGTTCTGGCCAGGGCAATTAGGCAGGAGAAGGAAATAAAAGGTATTCAATTAGGAAAAGAGGAAGTCAAATTGTCCCTGTTTGCAGACGACATGATTGTATATCTAGAAAACCCCATTGTCTCAGCCCAAAATCTCCTTAAGCTAATAAGCAACTTCAGCAAAGTCTCAGGATACAAAATCAATGTACAAAAATCACAAGCATTCTTATACACCAACAACAGACAAACAGAGAGCCAAATCATGAGTGAACTCCCATTCACAATTGCTTCAAAGAGAATAAAATACCTGGGAATCCAACTTACAAGGGATGTGAAGGACCTCTTCAAGGAGAACTACAAACCACTGCTCAAGGAAATAAAAGAAGATACAAACAAATGGAAGAACATTCCATGCTCATGGGTAGGAAGAATCAATATTGTGAAAATGGCCATACTGCCCAAGGTAATTTACAGATTCAATGCCATCCCCATCAAGCTACCAATGCCTTTCTTCATAGAATTGGAAAAAACTACTTTAAAGTTCACATGGAACCAAAAAAGCGCCTGCATTGCCAAGTCAATCCTAAGCCAAAAGAACAAAGCTGGAGGCATCACACTACCTGACTTCAAACTATACTACAAGGCTACAGTAACGAAAACAGCATGGTACTGGTACCAAAACAGAGATATAGATCAATGGAACAGAACAGAGCCCTCAGAAATAATGCCGCATATCTACAACTATCTGATCTTTGACAAAGCTGAGAAAAACAAGCAATGGGGAAAGGATTCCCTATTTAATAAATGGTGCTGGGAAAACTGGCTAGCCATATGTAGAAAGCTGAAACTGGATCCCTTCCTTACACCTTATACAAAAATCAATTCAAAATGGGTTAAAGACTTAAACGTTAGACCTAAAACCATAAAAACCCTAGAAGAAAACCTAGGCATTACCATTCAGGACATAGGCATGGGCAAGGACTTCATGTCTAAAACACCAAAAGCAATGGCAACAAAAGCCAAAATTGACAAATGGGATCTAATTAAACTAAAGAGCTTCTGCACAGCAAAGGAAACTACCATCAGAGTGAACAGGCAACCTATAGAATGGGAGAAAATTTTCGCAACGTACTCATCTGACAAAGGGCTAATATCCAGAATCTACAATGAACTCAAATAAATTTACAAGAAAAAAACAAACAACCCCACCAAAAAGTGGGTGAAGGACATGAACAGACACTTCTCAAAAGAAGACATTTATGCAGCCAAAAAACACATGAAAAAATGCTCACCATCACTGGCCATCAGAGAAATGCAAATCAAAACCACAATGAGATACCATCTCACACCAGTTAGAATGGTGATCATTAAAAAGTCAGGAAACAACAGGTGCTGGAGAGGATGTGGAGAAATAGGAACACTTTTACACTGTTGGTGGGACTGTAAACTAGTTCAACCATTGTGGAAGTCAGTGTTGCGATTCCTCAGGGATCTAGAACTAGAAATACCATTTGCCCCAGCCATCCCATTACTGGGTATATACCCAAAGGACTATAAATCATGCTGCTATAAAGACACATGCACACGTATGTTTATTGCAGCATTATTCACAATAGCAAAGACTTGGAACCAATCCAAATGTCCAACAATTATAGACTGGATTAAGAAAATGTGGCACATATATACCATGGAATACTATGCAGCCATAAAAAATGATGAGTTCATGTCCTTTGTGGGGACATGGATGAAATTGGAAATCATCATTCTCAGTAAACTATCGCAAGAACAAAAAACCAAACACCGCATATTCTCACTCATAGGTGGGAATTGAACAATGAGAACACATGGACACAGGAAGGGGAACATCACACTCTGGGGACTGTTGTGGGGTGGGGGGAGGGGGGAGGGATAGTATTGGGAGATATACCTAATGCTAGTTGACGAGTTAGTGGGTGCAGCGCACCAGCATGGCACATGTATACATATGTAACTAACCTGCACAATGTGCACATGTACCTTAAAACTTAAAGTATAAT*AATAATAAAAAA****A*GGAGATATACCTAATGTA**AATGACGAGTTAATAGGTGCAGCACACCAACATGGCGCATGTATACATATGTAACAAACCTGCACGTTGTGCAC

**Clone 2; PA3; SpIRE(97/622)-2: Filled site spans: chrX:69458442-69464121**

Empty Site:

CCAATTCAAATTACAGCAGATTTCTCATCTGAAACCATGAAGACCAGAAGGAAGTGGTACATTTTTCAAGTGCTTTTTTTCAAGAAAAGAGCTGTCAACTGTGACTTCTATATCTGATGA

AACTATACTTCAGGAATAAAGTGG**AAATAAAGACATTGC**C

ACATGAAAGATGACTGAAAGAATTGGTCACTAGTAGACCTCCCCTTAAAGAATTGCTAAAGAGGTTTCTTCAAGTAGAAAGGAAGTGATAAAAGAAGGAATCTTAGAATTGCAGGAAAGA

Filled Site:

CAAGAAAAGAGCTGTCAACTGTGACTTCTATATCTGATGAAACTATACTTCAGGAATAAAGTGG**AAATAAAGACATTGC**TGGGGGAGGAGCCAAGATAGCTGAATAGGAACAGCTCTGGTCTACAGCTCCCAGCATGAGCGATGCAGAAGATGGGTGATTTCTGCATTTCCGTCTGAGCTTTGAAGAGAGCAGTGGTTCTCCCAGCACGCAGCTGGAGATCTGAGAACAGGCAGACTGCTTCCTCAAGTGGGTCCCTGACCCCTGACCCCCGAGCAGCCTAACTGGGAGGCACCCCCCAGCAGGGGCAGACTGACATGTCACACGGCCAGGTACTCCAACAGACCTGCAGCTGAGGGTCCTGTCTGTTAGAAGGAAAACTAACAAACAGAAAGGACATCCACAACAAAAACCCATCCGTACATCACCATCATCAAAGACAAAAAGTAGATAAAACAACAAAGATGGGAAAAAAACAGAGCAGAAAAACTGGAAACTCTAAAAAGCAGAGTGCCTGTCCTCCTCCAAAGGAACGCAGTTCCTCACCAGCAATGGAACAAAGCTGGATGGAGAATGACTTTGACGAGCTGAGAGAAGAAGACTTCAGATGATCAAATTACTCCGAGCTATGGGAGAACATTCAAACCAAAGGCAAAGAAGTTGAAAACTTTGAAAAAAATTTAGAAGAATGTATAACTAGAATAACCAATACAGAGAAGTGCTTAAAGGAGCTGATGGAGCTGAAAACCAAGGCTCGAGAACTACGTGAAGAATGCAGAAGCCTCAGGAGCCGATGTGATCAACTGGAAGAAAGGGTATCAGCGATGGAAGATGAAATGAATGAAATTAAGCGAGAAGGGAAGTTTAGAGAAAAAAGAATAAAAAGAAACGAGCAAAGCCTCCAAGAAACATGGGACTATGTGAAAAGACCAAATCTATGTCTGATTGGTGTCCCTGAAACTGACGGGGAGAATGGAACCAAGTTGGAAAACACTCTGCAGGATATTATCCAGGAGAACTTCCCCAATCTAGCAAGGCAGGCCAACATTCAGATTCAGGAAATACAGAGAAAGCCACAAAGATACTCCTCGAGAAGAGCAACACCAAGACACATAATTGTCAGATTCACCAAAGTTGAAATGAAGGAAAAAATGTTGAAGACAGCCAGAGAGAAAGGTCGGGTTACCCACAAAGGGAAGCCCATCAGACTAACAGCAGATCTCTCAGCAGAAACTCTATAAGCCAGAAGAGAGTGGGGGCCAATATTCAACATTCTTAAAGAAAAGAATTTTCAATGCAGAATTTCATATCCAGCCAAACTAAGCTTCATAAGTGAAGGAGAAATAAAATACTTTACAGACAAGGAAATGCTGAGAGATTTTGTCACCACCAGGCCTGCCCTAAAAGAGCTCCTGAAGGAAGCGCTAAACATGGAAAGGAACAACCAGTACCAGCCGCTGCAAAATCATGCCAAAATGTAAAGACCATCGAGATTAGGAAGAAACTGCATCAACTAACCAGCGAAAGAACCAGCTAACATCATAATGACAGGATCAAATTCACACATAACACTATTAACTTTAAATGTAAATGGACTAAATGCTCCAATTAAAAGACACAGACTGGCAAATTGGAAAAAGAGTCAAGACACATCAGTGTGCTGTATTCAGGAAACCCATCTCATATGCAGAGATACACATAGGCTCAAAATAAAAGGATGGAGGAAGATCTACCAAGCAAATGGAAAACAAAAAAGGCAGGGGTTGCAATCCTAGTCTCTGATAAAACAGACTATAAACCAACATAGATCAAAAGAGACAAAGAAGGCCATTACATAATGGTCAAGGGATCAATTCAACAAGAAGAGCTAACTATCCTAAATATATATGCACCCAATACAGGAGCACCCAGATTCATAAAGCAAGTCCTGAGTGACCTACAAAGAGACTTAGACTCCCACACATTAATAATGGGAAACTTTAACAGCCCACTGTCAACATTAGACAGATCAACAAGACAGAACGTCAACAAGGATACCCAGGAATTGAACTCAGCTCTGCACCAAGCGGACCTAATAGACATCTACAGAACTCTCCACCCCAAATCAACAGAATATACATTTTTTTCAGCACCACACCACACCTATTCCAAAACTGACCATATAGTTGGAAGTAAAGCACTCCTCAGCAAATGTAAAAGAACAGAAATTATAACAAACTGTCTCTCAGACCACAGCGCAATCAAACTAGAACTCAGGATTAAGAATCTCACTCAAAACCACTCAACTACATGGAAACTGAACAACCTGCTCCTGAATGACTACTGGGTACATAACGAAATGAAGGCAGAAGTAAAGATGTTCTTTGAAACCAATGAGAACAAATACACGACATACCAGAATCTCTGGGATGCATTCAAAGCAGTGTGTAGAGGGAAATTTATAGCACTAAATGCCCACAAGAGAAAGCAGGAAAGATCCAAAATTGACACCCTAACATCACAATTAAAAGAACTAGAAAAGCAAGAGCAAACACATTCAAAAGCTAGCAGAAGGCAAGAAATAACTAAAATCAGAGCAGAACTGAAGGACATAGAGACACAAAAAACTCTTCAAAAAATTAAAGAATCCAGGAGCTGGTTTTTTGAAAGGATCAACAAAACTGATAGACCACTAGCAAGACTAATAAAGGAAAAAAGAGAGAAGAATCAAATAGACGCAATAAAAAATGATAAAGGGGATATCACCACCAATCCCACAGAAATACAAACTACCATCAGAGAATACTATAAACACCTCTACGCAAATAAACTAGAAAATCAGAAATGGATAAATTCCTCGACACATACACCCTCCCAAGACTAAACCAGGAAGAAGTTGAATCTCTGAAAAGACCAATAACAGGAGCTGAAATTGTGGCAATAATCAATAGCTTACCAACCAAAAAGAGTCCAGGACCAGATGGATTCACAGCCGAATTCTACCAGAGGTATAAGGAGGAGCTGGTACCATTCCTTCTGAAACTATTCCAATCAATAGAAAAAGAGGGAATCCTCCGTAACTCATTTTATGAGGCCAGCATAATTCTGATACCAAAGCCGGGCAGAGACACAACCAAAAAAGAGAATTTTAGACCAATATCCTTGATGAACATTGATGCAAAAATCCTCAATAAAATACTGGCAAAACGAATCCAGCAGCACATCAAAAAGCTTATCCACCATGATCAAGTGGGCTTCAACCCTGGGATGCAAGGCTGGTTCAATATACGCAAACCAATAAATGTAATCCAGCATATAAACAGAGCCAAAGACAAAAACCACATGATTATCTCAATAGATGCAGAAAAGGCCTTTGACAAAATTCAACAACCCTTCATGCTAAAAACTCTCAATAAATTAGGTATTGATGGGACGTATCTCAAAATAATAAGAGGTATCTATGACAAACCCACAGCCAATATAATACTGAATGGGCAAAAACTGGAAGCATTCCCTTTGAAAACTGGCACAAGACAGGGATGCCCTCTCTCACCACTCCTATTCAACATAGTGTTGGAAGTTCTGGCCAGGGCAATCAGGCAGGAGAAGGAAATAAAGGGTATTCAATTAAGAAAAGAGGAAGTCAAATTGTCCCTGTTTGCAGATGACATGATTGTATATCTAGAAAACCCCACTGTCTCAGCCCAAAATCTCCTTAAACTGATAAGTAACTTCAGCAAAGTCTCAGGATACAAAATCAATGTACAAAAATCACAAGCATTCTTATACACCAATAACAGACAAACAAGAGAGCCAAATCATGAGAGTGAACTCCCATTCACAATGGCTTCAAAGAGAATAAAATACCTAGGAATCCAACTTACAAGGGATGTGAAGGACCTCTTCAAGGAGAACTACAAACCACTGCTCAAGGAAATAAAAGAGGATACAAACAAATGGAAGAACATTCCATGTTCATGGGTAAGAGGAATCAATATCGTGAAAATGGCCATACTGCCCAAGGTAATTTATAGATTCAATGACATTCCCATCAAGCTACCAATGACTTTCTTCACAGAATTGGAAAAAACTACTTTAAAGTTCATATGGAACCAAAAAGGAGCCCTCATCGCCAAGTCAATCCTGAGCCAAAAGAACAAAGCTGGAGGCATCACACTACCTGACTTCAAACTATACTACAAGGCTACAGTAACCAAAACAGCATGGTACTGGTACCAAAACAGAGATATAGATCAATGGAACAGAACAGAGCCTTCAGAAATAACACCGCATATCTACAACTATATGATCTTTGACAAACCTGAGAAAAACAAGCAATGGGGAAAGGATTCCCTATTTAATAAATGGTGCTGGGAAAACTGGCTAGCCATATGTAGAAAGCTGAAACCGGATCCCTTCCTTACACCTTATACAAAATCAATTCAAGATGGATTAAAGACTTATATGTTAGACCTAAAACCATAAAAACCCTAGAAGAAAACCTAGGCATTACCATTCAGGACATAGGCATGGGCAAGGACTTCATGTCTAAAACACCAAAAGCAATGGCAACAAAAGACAAAATTGACAAATGGGATCTAATTAAATTAAAGAGTTCTGCACAGCAAAAGAAACTACCATCAGAGTGAACAGGCAACCTACAAAATGGGAGAAAATTTTCGCAACCTACTCATCTGACAAAGGGCTAATATCCAGAATCTACAATGAACTCAAACAAGTTTACAAGAAAAAAACAAACAACCCCATCAAAAAGTGGGCAAAGGATATGAACAGATACTTCTCAAAAGAAGACATTTATGCAGCCAAAAAACACATGAAAAATGCTCACTGTCACTGGCCATCAGAGAAATGCAAATCAAAACCACAATGACATACCATTTCACACCAGTTAGTATGACAATCATTAAAAAGTCAGGAAACAACAGGTGCTGGAGAGGATGTGGAGAAATAGGAACACTTTTACACTGTTGGTGGGACTGTAAACTAGTTCAACCATTGTGGAAGTCAGTGTGGCGATTCCTCAGGGATCTAGAACTAGAAATACCATTTGACCCAGCCATCCCATTACTGGGTGTATACCCAAAGGACTATAAATCATGCTGCTATAAAGACACATGCACACGTATGTTTATTGCGGCACTATTCACAATAGCAAAGAATTGGAACCAACCCAAATGTCCAACAATGATAGACTGGATTAAGAAAATGTGGCACATATACACCATGGAATACTATGCAGTCTTAAAAAATGATGAGTTCATGTCCTTTGTAGGGACATGGATGAAATTGGAAACCATCATTCTCGGTAAACTATTGCAAGAACAAAAAACCAAACACCACATATTCTCACTCATAGGTGGGAATTGAACTATGAGAACACATGGACACAAGAAGGGGAACATCACACTCTGGGGACTGTTGTGGGGTTGGGGGAGGGGGTAGGGATTGCAGTGTGAGATATACCTAATGCTAGAGGACGAGTTAGTGGGTGCAGTGCACCAGCATGGCACATGTGTACATATATAACTAACCTGCACATTGTGCACATGTATCCTAAAACTTAAAGTAT*AAAAAAAAATAAAATAAAAA****AAATAAA*GACATTGC**CACATGAAAGATGACTGAAAGAATTGGTCACTAGTAGACCTCCCCTTAAAGAATTGCTAAAGAGGTTTCTTCAAGTAG

**Clone 4; PA3; SpIRE(97/622)-4: Filled site spans: chrX:113445893-113451601**

Empty Site:

ATGAAATACCACTTCACACTCACTAGGATGGCTAGAATTTTCTTTAAAATAGACAATAACAAGTTTTGTATACTATGTGGAGAAACTGAATCCCTCATGCACTGCTAGTGGGAATGCAAA

TGTTGTACTCCCTT**TAAAAGCAGTATTCCC**AATCCTGAAA

ATGTTAAACACAGAGTTATCATATTCAACTCGCAGATACATACGACAGAAAATAAAAATATATGTTCACACAGTAAGTTGTAGACAAATGTTCATAGCAGCTTTATCACAATAGCCAAAA

Filled Site:

AGAAACTGAATCCCTCATGCACTGCTAGTGGGAATGCAAATGTTGTACTCCCTT**TAAAAGCAGTATTCCC**GGGGGAGGAGCCAAGGTAGCCGAATAGGAACAGCTCTGGTCTACAGCACCCAGCGTGAGCGACGCAGAAAACgGGTGATTTCTGCATTTCCATCTGAGCTTTGAAGAGAGCAGTGGTTCTCCCAGCACGCAGCTGGAGATCTGAGAGCGGGCAGACTCCCTCCTCAAGTGGGTCCCTGACCCCTGACACCCGAGCAGCCTAACTGGGAGGCATCCCCCAGCAGGGGCAGACTGACACCTCACAGGGCCAGGTACTCCAACAGACCTGCAGCTGAGGGTCCTGTCTGTTAGAAGGAAAACTAACAAACAGAAAGGACATCCACACCAAAAACCCATCTGTACGTCACCATCATCAAAGACCAAAAGTAGATAAAACCACAAAGATGGGGAAAAAACAGAGCAGAAAAACTGGAAACTCTAAAAAGCAGAGCGCCTCTCCTCCTCCAAAGGAACACAGTTCCTCACCAGCAATGGAACAAAGCTGGACGGAGAATGACTTTGACTAGCTGAGAGAAGAAGGCTTCAGACGATCAAATTACTCCGAGCTATGGGAGGCCATTCAAACCAAAGGCAAAGAAGTTGAAAACTTTGAAAAAAGTTTAGAAGAATGTATAACTAGAATAACCAATACGGAGAAGTGCTTAAAGGAGCTGATGGAGCTGAAAACCAAGGCTCGAGAACTACGTGAAGAATGCAGGAGCCTCAGGAGCCGATGTGATCAACTGGAAGAAAGGGTATCAGCCATGAAAGATGAAGTGAATGAAATGAAGCCAGAAGGGAAGTTTAGAGAAAAAAGAATAAAAAGAAACGAGCAAAGCCTCCAAGAAATATGGGACTATGTGAAAAGACCAAATCTACATCTGATTGGTGTACCTGAAACTGACGGGGAGAATGGAACCAAGTTGGAAAACACTCTGCAGGATATCATCCAGGAGAACTTCCTCAATCTAGCAAGGCAGGCCAACATTCAGATTCAGGAAATACAGAGAACGCCACAAAGATACTCCTTGAGAAGAGCAACTCCAAGACACATAATTGTCAGATTCACCAAAGTTGAAATGAAGGAAAAAATGTTAAGGGCAGCCAGAGAGAAAGGTCGGGTTACCAACCAAGAGAAGCCCATCAGACTAACAGCGGATCTCTTGGCAGAAACTCTACAAGCCAGAAGAGAGTGGGGGCCAATATTCAACATTCTTAAAGAAAAGAATTTTCAACCCAGAATTTCATATCCAGCCAAACTAAGCTTCATAAGTGAAGGAGAAATAAAATACTTTACAGACAAGCAAATGCTGAGAGGTTTTGTCACCACCAGGCCTGCCCTAAAAGAGCTCCTAAAGGAAGCGCTAACCATGGAAAGGAACAACTGATACCAGCCACTGCAAAATCATGCCAAAATGTAAAGACCATCGAGACTAGGAAGAAACTGCATGAACTAACAAGCAAAATACCCAGCTAACATCATAATGACAGGATCAAATTCACACATAACAATATTAACTGTAAATGTAAATGGACTAAATGCTCCAATTAAAAGACACAGACTGGCAAATTGGATAAAGAGTCAAGACCCATCAGTGTGCTGTATTCAGGAAACCCATCTCATGTGCAGAGACACACATAGACCCAAAATAAAAGGAGGGAGGAAGATCTACCAAGCAAATGGAAAACAAAAAAAGGCAGAGGTTGCAATCCTAGTCTCTGATAAAACAGACTTTAAACCAACAATGATCAAAAGAGACAAAGAAGGCCATTACATAATGGTAAAGGGATCAATTCAACAAGAAGAGCTAACTATCCTAAATATATATGCACCCAATACAGGAGCACCCAGATTCATAAAGCAGTCCTGAGCAACCTACAAAGAGACTTAGACTCCCACACATTAATAATGGGAGACTTTAACACCCCACTGTCAACATTAGACAGATCAACGAGACAGAAAGTCAACAAGGATACCCAGGAATTGAACTCACCTCTGCACCAGTGGACCTAATAGACATCTACAGAACTCTCCACCCCAAATCAACAGAATATATATTTTTTTCAGCACCACACCACACCTATTCCAAAATTGACCACATACTTGGAAGTAAAGCTCTCCTCAGCAAATGTAAAAGAACAGAAATTAAAACAAACTATCTCTCAGACCACGGTGCAATCAAACTAGAACTCAGGATTAAGAATCTCACTCAAAACCACTCAACTACATGGAAACTGAACAACCTGCTCCTGAATGACTACTGGGTACATAACGAAATGAAGGCAGAAATAAAGATGTTCTTTGAAACCAATGAGAACAAAGACACAACATACCAGAATCTCTGGGACACATTCAAAACAGTGTGTAGAGGGAAATTTATAGCACTAAATGCCCACAAGAGAAAGCAGGAAAGATCCAACATCGACACCCTAACATCACAATTAAAAGAACTAGAAAAGCAAGAGCAAACACATTCAAAAGCTAGCAGAAGGCAAGAAGTAACTAAAATAAGAGCAGAACTGAAGGAAATAGAGAACCAAAAACCCTTCAAAAAATCAATGAATCCAGGAGCTGGTTTTTTGAAAGGATCAACAAAATTGATAGACTGCTAGCAAGACAAATAAAGAAAAAAAGAGAGAAGAATCAAAGAGACGCAATAAAAAATGATAAAGGGGATATCACCACCGATCCCACAGAAATACAAAGTACCATCAGAGAATACTACAAACACCTCTACGCAAATAAACTAGAAAATCTAGAAGAAATGGATAAATTCCTCGACACATACACTCTCCCAAGACTAAACCAGGAAAAAGTTGAATCTCTGAATAGACCAATAACAGGCTCTGAAATTATGGCAATAATCAATAGCTTACCAACCAAAAAGAGTCCAGGACCAGATGGATTCACAGCCGAATTCTACCAGAGGTAAAAGGAGGAACTGGTACCATTCCTTCTGAAACTATTCCAATCAATAGAAAAAGAGGGCATCCTCCCTAACTCATTTTATGAGGCCAGCATCATCCTGATACCAAAGCCGGGCAGAGACACAACAGAAAAAGATAATTTTAGACCAATAACCTTGATGAACATTGATGCAAAAATCCTCAATAAAATACTGGCAAAACGAATCCAGCAGCACATCAAAAAGCTTATCCACCATGATCAAGTGGGCTTCATCCCTGGGATGCAAGGCTGTTTCAATATATGCAAATCAATAAATGTAATCCAGCATACAAAGAGAAGCAAAGACAAAAACCACGATTATCTCAATAGATGCAGAAAAGGCCTTTGACAAAATTCAACAGCCCTTCATGCTAAAAACTCTCAATAAATTAGGTATTGATGGGATGTATCGCAAAATAATAAGAGCTATCTATGACAAACCCACAGCCAATATCATACTGAATGGGCAAAAACTGGAAGCATTCCCTTTGAAAACTGGCACAAGACAGGGATGCCCTCTCTCACCACTCCTATTCAACATAGTGTTGGAAGTGCTGGCCAGCGCAATTAGGCAGGAGAAGGAAATAAAGGGTATTCAATTAGGAAACGAGGAAGTCAAATTGTCCCTGTTTGCAGATGACATGACTGTATATCTAGAAAACCCCATTGTCTCAGCCCAAAATGTCCTTAAGGTGATAAGCAACTTCAGCAAAGTCTCAGGATACAAAATCAATGTGCAAAAATCACAAGCATTCTTATACACCAACAACAGACAAACAGAGAGCCAAATCATGAGTGAACTCCCATTCACAATTGCTTCAAAGAGAATAAAATACCTAGGAATCCAGCTTACAAGGAATGTGAAGGACCTCTTCCAGGAGAACTACAAACCACTGCTCAAGGAAATAAAAGAGGATACAAACAAATGGAAGAACATTCCATGCTCATGGGTAGGAAGAATCAATATCGTGAAAATGGCCATACTGCCCAAGGTAATTTACAGATTCAACGCCATCCCCATCAAGCTACCAATGACTTTCTTCACAGAATTGGAAAAAACTACTTTAAAGTTCATATGGAACCAAAAAAGAGCCCGCATTGCCAAGTCAATCCTAAGCCAAAAGAACAAAGCTGGAGGCATCACACTACCTGACTTCAAACTATGCTACAAGGCTACAGTAACCAAAACAGCATGGTACTGGTACCAAAACAGAGATATAGATCAATGGAACAGAACAGAGCCCTCAGAAATAACGCCACATATCTACAACTATCTGATCTTTGACAAACCTGACAAAAACAAGAAATGGGGAAACGATTCCCTATTTAATAAATGGTGCTGGGAAAACTGGCTAGCCATATGTAGAAAGCTGAAACTGGATCCCTTCCTTACACCTTATACAAAAATCAATTCAAGATGGATTAAAGACTTAAATGTTAGACCTAAAACCATAAAAACCCTAGAAGAAAACCTAGGCATTACCATTCAGGACATAGGCACGGGCAAGGACTTCATGTCTAAAACACCAAAAGCAATGGCAACAAAAGCCAAAATTGACAAATGGGATCTAATTAAACTAAAGAGCTTCTGCACAGCAAAAGAAACTACCATCAGAGTGAACAGGCAACCTACCAAATGGGAGAAAATTTTCACAACCTACTCATCTGACAAAGGGCTAATATCCAGAATCTACAATGAACTCCAACAAATTTACAAGAAAAAAACAAACAACCCCATCAAAAAGTGGGCGAAGGACATGAAAAGACACTTCTCAAAAGAAGACATTTATGCAGCCAAAAAACACATGAAAAAATGCTCACCATCACTGGCCATCAGAGAAATGCAAATCAAAACCACAATGAGATACCATCTCACACCAGTTAGAATGGTGATCATTAAAAAGTCAGGAAACAACAGGTGCTGGAGAGGATGTGGAGAAATAGGAACACTTTTACACTGTTGGTGGGACTGTAAACTAGTTCAACTATTGTGGAAGACAGTGTGGCTATTCCTCAGGGATCTAGAACTAGAAATACCATTTGACCCAGCCATCCCATTACTGGGTATATACCCAAAGGACTATAAATCATGCTGCTATAAAGACACATGCACACATATGTTTATTGCGGCATTATTCACAATAGCAAAGACTTGGAACCAAGCCAAATGTCCAACAATGAGAGACTGGATTAAGAAAATGTGGCACATATACACCATGGAATACTATGCAGCCATAAAAAATGATGAGTTCACGTCCTTTGTAGGGACATGGATGAAATTGGAAATCATCATTCTCAGTAAACTATCGCAAGAACAAAAAACCAAACACCGCATATTCTCACTCATAGGTGGGAATTGAACAATGAGAACACATGGACACAGGAAGGGGAACATCACACTCTGGGGACTGTTGTGGGGTGGGGGGAGGGGGGAGGGATAGCATTGGGAGATATACCTAATGCTAGATGACGAGTTAGTGGGTGCAGCGCACCAGCATGGCACATGTGTACATATGTAACTAACCTGCACATTGTGCACATGTACCCTAAAACCTAAAGTATAATAATAAT*AAATTTTAAAAAAAATTTTAAAAAGTTTCCAAAAAATAAAAAATAAATAAATAAA****TAAAA*GCAGTATTCCC**AATCCTGAAAATGTTAAACACAGAGTTATCATATTCAACTCGCAGATACATACGACAGAAAATAAAAATATA

**Clone 8; PA3, SpIRE(97/622)-8: Filled site spans: chr1:92232048-92237799**

Empty Site:

ATGAAAAGGGGCACTTAACAGTTGATTAGAAAATTTAAAAAATGACAACATGAAACTTTTCACCACAGAAATGTAAATTTCATAAAGTTCAACTTAACACATTTAAAAGCCTCTATAAAA

TGGGCAAATGTTTGT**AAAAATATA**TATAATTTAGAAAATGGAC

TCACAACAAAGTATAAAACAAAATACAGCAAAAAGCATAAAACCACACGTTACTATGGAACAATCATTTCTTGCCCCTTCATTCCAGACATCAGCTGGTGTCCGTAAGATAAATATCAGG

Filled Site:

CATAAAGTTCAACTTAACACATTTAAAAGCCTCTATAAAATGGGCAAATGTTTGT**AAAAATATA**ATTTATGGGGGAGGAGCCAAGATGGCCAAATAGGAACAGCTCCTGTCTACAGCTCCCAGCATGAGCAACGCAGAAGACAGGTGATTTCTGCATTTCCATCTGAGCTTTGAAGAGAGCAGTGGTTCTCCCAGCACGCAGCTGGAGATCTGAGAACAGGCAGACTGCCTCCTCAAGTGGATCCCTGACCCCTGAGCAGCCTAACTGGGAGGCACCCCCCAGTAGGGGCAGACTGACACCTCATACGGCCGGGTACTCCTCTGAGACAAAACTTCCAGAGGAATGATCAGACAGCAGCATTCGTGGTTCACAAAAAACCACTGTTCTGCAGACACCACTGCTGATACACAGGCAAACAGGGTCTGGAGTGGACCTCTAGCAAACTTCAACAGACCTGCAACTGAGGGTCCTGTCTGTTAGAAGGAAAACTAACAAACAGAAAGGACATCCACACCAAAAACCCATCTGTACATCACCATCATCAAAGACCAAAAGTAGATAAAACCACAAAGATGGGGAAAAAACAGAGCAGAAAAAATGGAAACTCTAAAAAGCAGAGCACCTCTCCTCCTCCAAAGGATCACAGTTCCTCACCAGCAATGGAACAAAGCTGGACAGAGAATGACTTTAACGAGTTGAGAGAAGAAGGCTTCAGACAATCAAACTACGAGCTACAGGAGGAAATTCAAACCAAAGGCAAAGAAGTTAAAAACTTTGAAAAAAATTTAGGCGAACGTATAACTAGAATAAACAATACAGAGAAGTGCTTAAAGGAGCTGATGGAGCTGAAAGCCAAGGCACGAGAAGTACGTGAAGAATGCAGAAGCCTCAGGAGCCAACGCGATCAACTGGAAGAAAGGGTATCAGCGATGGAAGATGAAATGAATGAAATGAAGCGAGAAGGGAAGTTTAGAGAAAAAAGAATAAAAAGAAACGAACAAATCCAGCAAGAAATATGGGACTATGTGAAAACACCAAATCTACGTCTGATTGGTGTACCTGAAAGTGACGGGGAGAATGGAACCAAGTTGGAAAACACTCTGCAGCATATTATCCAGGAGAACTTCCCCAATCTAGCAAGGCAGGCCAACATTCAGATTCAGGAAATACAGAGAACGCCACAAAGATACTCCTCAAGAAGAGCAACTCCAAGACACATAATTGTCAGATTCACCAAAGTTGAAATAAAGGAAAAAATGTTAAGGGCAGCCAGAGAGAAAGGTCGGGTTACCCACAAAGGGAAGCCCATCAGACTAACAGCAGATCTCTAGGCAGAAACTCTACAAGCCAGAAGAGAGTGGGGGCCAATATTCGACATTCTTAAAGAAAAGAATTTTCAACCCAGAATTTCATATCCAGCCAAACTACGCTTCATAAGTGAAGGAGAAATAAAATCCTTTACAGACAAGCAAATGCTGAGAGATTTTGTCACCAGCAGGCCTGCCCTAAAAGAGCTCCCAAAGGAAGCACTAAACATGGAAAGGAACAACCGGTACCAGCCGCTGCAAAATCATGCCAAAATGTAAAGACCATCGAGACTAGGAAGAAACTGCATCAACTAACGAGCAAAATAACCAGCTAACATCATAATGACAGGATCAAATTCACACATAACAATATTAACCTTAAATGTAAATGGGCTAAATGCTCCAATTAAAAGACACAGAATGGCAAATTGGATAGAGTCAAGACCCATCGGTGTGCTGTATTCAGGAAACCCATCTCACATGCAGAGACACACATAGGCTCAAAATAAAAGGATGGAGGAAGATCTACCAAACAAATGGAAAACAAAAAAAGGCAGGGGTTGCAATCCTAGTCTCTGATAAAACAGACTTTAAACCAACAAAGATCAAAAGAGACAAAGAAGGCCATTACATAATGGTAAAGGGATCAATTCAACAAGAGGAGCTAACTATCCTAAATATATATGCACCCAATACAGGAGCATCCAGATTCATAAAGCAAGTCCTGAGTGACCTACAAAGAGACTTAGACGCCCACACACTAATAATGGGAGACTTTAACACCCCACTGTCAACATTAGACAGATCAACGAGACAGAAAGTCAACAAGGATACCCAGGAATTGAACTCAGCTCTACACCAAGCAGACATAACAGACATCTACAGAACTCTCCACCCCAAATCAACAGAATATACATTTTCTTCAGCACCACACCACACCTATTCCAAAACTGACCACATAGTTGGAAGTAAAGCTCTCCTCAGCAAATGTAAAAGAACAGAAATTATAACAAACTATCTCTCAGACCACAGTGCAATCAAACTAGAACTCAGGATTAAGAAACTCACTCAAAACTGCTCAACTACATGGAAACTGAACAACCTGCTCCTGAATGACTACAGGGTACATAATGAAATGAAGGCAGAAATAAAGATGTTCTTTGAAACCAATGAGAACAAAGACGCAACATACCAGAATCTCTGGGACACATTCAAAGCAGTGTGTAGAGGGAAATTTATAGCACTAAATGCCCACAAGAGAAAGCAGGAAAGATCCAAAACTGACACCCTAACATCACAATTAAAAGAACTAGAAAAGCAAGAGCAAACACATTCAAAAGCTAGCAGAAGGCAAGAAATAACTAAAATCAGAGCAGAAGTGAAGGAAATAGAGACACAAAAAACCCTTCAAAAAATTAATGAATCCAGGAGCTGGTTTTTTGAAAGGATCAACAAAATTGATAGACCGCTAGCAAGACTAAAAGAAAAAAAGAGAGAAGAATCAAATAGACACAATAAAAAATGATAAAGGGGATATCACCACTGATCCCACAGAAATACAAACTACCATCAGAGAATACTACAAACACCTCTATGCAAATAAACTAGAAAATCTAGAATAAATGGATAAATTCCTCGACACATACACTCTCCCAAGACTAAACCAGGAAGAAGTTGAATCTCTGAATAGACCAATAACAGGAGCTGAAATTGTGGCAATAATCCATAGCTTACCAACCAAAAAGAGTCCAGGACCAGATGGATTCACAGCCTAATTCTACCAGAGGTAAAAGGAGGAGATGGTACCATTCCTTCTGAAACTATTCCAATCAATAGAAAAAGAGGGAATCCTCCCTAACTCATTTTATGAGGCCAGCATCATCCTGATACCAAAGCCTGGCAGAGAAACAACAAAAAAAGAGAATTTTAGACCAATATCCTTGATGAACATTGATGCAAAAATCCTCAATAAAATACTGGCAAACCGAATGCAGCAGCACATCAAAAAGCTTATCCACCATGATCAAGTGGGCCTCATCCCTGGGATGCAAGGCTGGTTCAATATACGCAAATCAATAAATGTAATCCAGCATATAAACAGAACCAAAGACAAAAACCACATGATTATCTCAAAAGATGCAGAAAAGGTCTTTGACAAAATTCAACAACCCTTCATGCTAAAAACTCTCAATAAATTAGGTATTGATGGGACGTATCTCAAAATAATAACAGCTATCTATGACAAACCCACAGGCAATATCATACTCAATGGGCAAAAACTGGAAGCATTCCCTTTGAAAACTGGCACAAGACAGGGATGCCCTCTCTCACCACTCCTATTCAACATAGTGTTGGAAGTTCTGGCCAGGGCAATTAGGCAGGAGAAGGAAATAAAGGCTATTCAATTAGGAAAAGAGGAAGTCAAATTGTCCCTGTTTGCAGATGACATGATTGTGTATCTAGAAAACCCCATCGTCTCAGCCCAAAATCTCCTTAAGCTGATAAGCAACTTCAGCAAAGTCTCAGGATACAAAATCAATGTACAAAAATCACAGGCATTCTTACACACCAATAACAGACAAACAGAGAGCCAAATCATGAGCAAACTCCCATTCACAATTGCTACAAAGAGAATAAAATACCTAGGAATCCACCTTACAAGGGATGTGAAGGACCTCTTCAAGGAGAACTACAAACCACTGCTCAATGAAATTAAAGAAGATACAAACAAATGGAAGAACATTCCATGCTCATGGGTAGGAAGAATCAATATCGGGAAAATGGCCATACTGCCCAAGGTAATTTATAGATTCAATGCCATCCCCATCAAGCTACCAATGACTTTCTTCACAGAATTGGAAAAAACTACTTTAAAGTTCACATGGAACCAAAAAAGAGCCCTCATTGCCAAGTCAATCCTAAGCCAAAAGAACAAAGCTGGAGGCATCACACTACCTGACTTCAAACTATACTACAAGGCTACAGTAACCAAAACAGCATGGTACTGGTACCAAAACAGAGATATAGATCAACGGAACAGAACACAGCCCTCAGAAATAACGCCGCATATCTACAACTATCTGATCTTTGACAAACCTGAGAAAAACAAGCAATGGGGAAAGGATTCCCTATTTAATAAATGGTGCTGGGAAAACTGGCTAGCCATATGTAGAAAGCTGAAACTGGATCCCTTCCTTACACCTTATACAAAAATCAATTCAAGATGGATTAAAGACTTAAACGTTAGACCTAAAATCATAAAAACCCTAGAAGAAAACCTAGGTATTACCACCCAAGACATAGGCATGGGCAAGGACTTCACGTCTACAATACCAAAAGCAATGGCAACAAAAGCCAAAATTGACAAATGGGATCTAATTAAACTAAAGAGCTTCTGCACAGTAAAAGAAACTACCATCAGAGTGAACAGGCAACCCACAAAATGGGAGAAAATTTTCACAACCTACTCATCTGACAAAGGGCTAATATCCAGAATCTACAATGAACTCAAACAAATCTACAAGAAAAAAACAAACAACCCCATCAAAAAGTGGGTGAAGGACATGAACAGACACTTCCCAAAAGAAGACATTTATGCAGCCAAAAAACACATGAAAAAATGCTCACCATCACTGGCCATCAGAGAAATGCAAATCAAAACCACTATGAGATATCATCTCACACCAGTTAGAATGGCAATCATTAAAAAGTCAGGAAACAACAGGTGCTGGAGAGGATGTGGAGAAATAGGAACACTTTTACACTGTTGGTGGGACTGTAAACTAGTTCAACCATTGTGGAAGACAGTGTGGTGATTCCTCAGGGATCTAGAACTAGAAATACCATTTGACCCAGCCATCCCATTACTGGGTAATACCCAAAGGACTATAAATCATGCTGCTATAAAGACACATGTACACGTATGTTTACTGCGGCACTATTCACAATAGCAAAGACTTGGAACCAACCCAAATGTCCAACAATGATAGACTGGATTAAGAAAATGTGGCACATATACACCATGGAATACTATGCAGCCACAAAAAATGATGAGTTCATGTCCTTTGTAGGGACATGGATGAAGGTGGAAACCATCATTCTCAGCAAACTATCGCAAGGACAAAAAACCAAACACCGCATGTTCTCACTCATAGGTGGAAATTGAACAATGAGAACACATGGACGCAGGAAGGGGAACATCACACTCTGGGGACTGTTGTGGGGTGGGGGGAGGGAGGACGGATAGCATTGGGAGATATACCTAATGCTAGATGACGAGTTAGTGGGTGCAGCGCACCAGCATGTCACATATATATGTATGTAACTAATCTGCACACTGTGCACATGTACCCTAAAACTTAAAGTATAATAAT***AAAAATATA****TATAATTTAT*GAAAATGGACTCACAACAAAGTATAAAACAAAATACAGCAAAAAGCATAAAA

**Clone 10; PA3; SpIRE(97/622)-10: Filled site spans: chr1:100676811-100682600**

Empty Site:

AGAGCTAGTAGCGGGACCTAAAGTGAATAAAGAGCGCAGAAAGGTTTTATTCATAGCTATCTTGTTTTGGTTATAAATCCTCAATAAGACCATAGTGTTATAATGAAAGTTTAGTGGAAT

TAAACTTCGGCATCTC**AAAAATATGTTATA**AATTGCAT

TCACCTTCAATCAAAAGGGCCTGACAACTGAAGGCTGTCAATTAAACAATAGCAGTGGCAGCAGATAATGGTGCACGTCAAGTGTGCGGACCAAAAATTTTGAATGGCAAAGA

Filled Site:

TAAACTTCGGCATCTC**AAAATTATGTTATA**GAGCCAAGATGGCCAAATAGGAACAGCTCTGGTCTACGGCTCCCAGCGTGAGCGACGCAGAAGACGGGTGAGCGACGCAGAAGACGGGTGACTTCCGCATTTCCATCTGAGCTTTGAAGAGAGCAGTGGTTCTCCCAGCACGCAGCTGGAGATCTGAGAACGGGCAGACTGCCTCCTGAAGTGGGTCCCTGACCCCTGACCCCAGAGAAGCCTAACTGGGAGGCACCCCCAGTAAGGACAGACTGACACCTCACATGGCCAGGTACTCCTCTGAGACAAAACTTCCAGAGGAATGATCAGACAGCAGCATTCGTGGTTCATGAAAATCCGCAGTTCTGCAGCCACCACTGCTGTTACCCATGCAAACAGGGTCTGGAGTGGTCCTCTAGCAAACTCCAACAGACCTGCAGCTGAGGGTGCTGTCTGTTAGAAGGAAAACTAACAAACAGAAAGGACATCCACACCAAAAACCCATCTGTACATCACCATCATCAAAGACCAAAAGTAGATAAAACCACAAAGACAGGGAAAAAAACAGAGCAGAAAAACTGGAAACTCTAAAAAGCAGAGTGGCTCTCCTCCTCCAAAGGAACGCAGTTCCTCACCAGCAACGGAACAAAGCTGGATGGAGAATGACTTTGATGAGTTGAGAGAAGAAGTCTTCAGACGATCAAACTACTCCGAGCTACAGGAGGAAATTCAAACCAAAGGCAAAGAAGTTGAAAACTTTGAAAAAAATTTAGACGAATGTATAACTAGAATAACCAATACAGAGAAGTGCTTAAAGGAGCTGATGGAGCTGAAAGCCAAGGCTCGAGAACTACGTGAAGAATGCAGAAGCCTCAGGAGCCGATGCGATCAACTGGAAGAAAGGGTATCAGTGATGGAAGATGAAATGAATGAAATGAAGTGAGAAGGGAAGTTTAGAGAAAAAAGAATACAAAGAAATGAACAAAGCCTCCAAGAAATATGGGACTATGTGAAAAGATCAAATCTACGTCTGATTGGTGTACCTGAAAGTGACGGGGAGAATGGAACCAAGTTGGAAAACACTCTGCAGGATATTATCCAGGAGAACTTCCCCAGTCTAGCAAGGCAGGCCAACATTCAGATTCAGGAAATACAGAGAACGCCACAAAGATACTCCTCGAGAAAAGCAACTCCAAGACACATAATTGTCAGATTCACCAAAGTTGAAATGAAGGAAAAAATGTTAAGGGCAGCCAGAGAGAAAGGTCGGGTTACCCACAAAGGGAAGCCCATCAGACTAACAGCAGATCTCTCAGCAAAAACCCTGCAAGCCAGAAGAGAGTGGGGGCCAATATTCAACATTCTTAAAGAAAAGAATTTTCAACTCAGAATTTCATTTCCAGCCAAACTAAGCTTCATAAGTGAAGGAGAAATAAAATCCTTTACAGACAAGCAAATGCTGAGAGATTTTGTCACCACCAGGCCTGCCCTAAAAGAGCTTCTGAAGGAAGCGCTAAACATGGAAAGGAACAACTGGTACCAGCCACTGCAAAATCATGCCAAATTGTAAAGACCATCAAGGCTAGGAAGAAACTGCATCAACTAATGAGCAAAATAACCAGCTAACATCATAATGACAGGATCAAATTCACACATAACAATATTAACTTTAAATGTAAATGGACTAAATGCTCCAATTAAAAGACACAGACTGGCAAATTGGATAAAGAGTCAAGACCCATCAGTGTGCTGTATTCAGGAAACCCATCTCACGTGCAGAGACACACATAGGCTCGAAATAAAGGGATGGAGGAAGATCTACCAAGCAAATGGAAAACAAAAAAAGGCAGAGGTTGCAATCCTAGTCTCTGATTAAACAGACTTTAAACCAACAAAGATCAAAAGAGACAAAGAAGGCCATTCCATAATGGTAAAGGGATCAACTCAACAAGAAGAGCTAACTATCCTAAATATATATGCACACAATACAGGAGCACCCAGTTTCATAAAGCAAGTCCTGAGTGACCTACAAAGAGACTTAGACTCCCACACAATAATAATGGGAGACTTTAACACCCCACTGTCAACATTAAACAGATCAACGAGACAGAAAGTTAATAAGGATACCCAGGAATTGAACTCAGCTCTGTACCAAGTGGACCTAATAGACATCTACAGAACTCTCCACCCCAAATCAACAGAATATACATTTTTTTCAGCACCACACCACACCTATTCCAAAATTGACCACATACTTGGAAGTAAAGCTCTCCTCAGCAAATGGAAAAGAACAGAAATTATAACAAACTGTCTCTCAGACGACCATGCAATCAAACTAGAACTCAGGATTAAGAAACTCACTCAAAACCGCTCAGTTACATGGAAACTGAACAACCTGCTCCTGAATGACTACTGGGTACGTAACGAAATGAAGCAGAAATAAAGATGTTCTTTGAAACCACGAGAACTAAGACACAACATACCAGAATCTCTGGGACACATTCAAAGCAGTGTGTAGAGGGAAATTTATAGCACTAAATGCCCACAAGAGAAAGCAGGAAAGATCCAAAATTGACACCCTAACATCACAATTAAAAGAACTAGAAAAGCAAGAGCAAACACATTCAAAAGCTAGCAGAAGGCAAGAACTAATTAAAAACAGAGCAGAACTGAAGGAAATAGAGACACAAAAAACCCTTCAAAAAATTAATGAATCCAGGAGCTGGTTTTTTGAAAGGATCAACAAAATTGATAGACCGCTAGCAAAACTAATAAAGAAGAAAAGAGAGAAGAATCAAATAGACGCAATAAAAAATGATAAAGGGGATATCACCACTGATCCCACAGAAATACAAACTACCATCAGAGAATATTACAAACACCTCTATGCAAATAAACTAGAAAATCTAGAAGAAATGGATACATTCCTCGACACATACACATTTCCAAGACTAAACCAGGAAGAATTTGACTCTCTGAATAGACCAATAACAGGCTCTGAAATTGTGGCAATAATCAATAGCTTACCAACCAAAAAGAGTCCAGGACCAGACGGATTCACAGCCAAATTCTACCAGAGGTACAAGGAGGAATTGTTACCATTCCTTCTGAAACTATTCCAATCAATAGAAAAAGAGGGAATCCTCCCTAACTCATTTTATGAGCCCAGCATCATCCTGATACCAAAGCTGGGCAGAGACACAACCAAAAAAGAGAATTTTAGACCAATATCCTTGATGAACATTAATGCAAAAATCCTCAATAAAATACTGGCAAACCAAATCCAGCAGCACATCAAAAACTTATCCACCATGATCAAGTGGACTTCATCCCTGGCATGCAAGGCTGGTTCAATATATGCAAATCAATAAATATAATCCAGCATATAAACAGAACCAAAGACAAAAACCACATGATTATCTCAATAGATGCAGAAAAGGCCTTTGACAAAATTCAACAACTCTTCATGCTAAAAACTCTCAATAAATTAGGTATTGATGGGACATATCTCAAAATAATAGGAGCTATCTATGACAAACACACAGCCAATATCATACTGAATGGGCAAAAACTGGAAGCATTCCCTTTGAAAACGGGCACAAGACAGGGATGCCCTCTCTCACCACTCCTATTCAACATAGTGTTGGAAGTTCTGGCCAGGGCCATTAGGCAGGAGAAGGAAATAAAGGGTATTCAATTAGGAAAAGAGGAAGTCAAATTGTCCCTGTTTGCAGATGACATGATTGTATTATCTAGAAAACCCCATTGTCTCAGCCCAAAATCTCCTTAAGCTGATAAGCAACTTCAGCAAAGGCTCAGGATACAAAATTGATGTGCAAAAATCACAAGCATTCTTATACACCAATAGCAGACAAACAGAGAGCCAAATCATGAGGGAACTCCCATTCAAAATTGCTTCAAAGAGAATAAAATACCTAGAAATCCAACTTACAAGGGATGTGAAGGACCTCTTCAAGAAGAACTACAAAACACTGCTCAATGAAATAAAAAAGGATACAAACAAATGGAAGAACATTCCATGCTCATGGTTAGGAAGAATCAATATCGTGAAAATGGCCATACTGCCCAAGGTAATTTATAGATTCAATGCCATCCCCATCAAGCTACCAATGACTTTTTTCACAGAATTGGAAAAAACTACTTTAAAGTTCATATGGAACCAAAAAAGAGCCCATATCGCCAAGTCAATCCTAAACCAAAAGAACAAAGCTGGAGGCATCAGGCTACCTGACTTCAAACTATACTACAAGGCTACAGTAACCAAAACAGCATGGTACTTGTACCAAAACAGAGATATAGACCAATGGAACAGAACAGAGCCCTCAGAAATAACGTCGCATATCTACAACTGTCTGATCTTTGACAAACCTGACAGAAACAAGAAATGGGGAAAGGATTCCCTATTTAATAAATGGTGCTGGGAAAACTGACTAGCCATATGTAGAAAGCTGAAACTGGATCCCTTCCTTACACCTTATACAAAAATTAATTCAAGATGAATTAAAGACTTAAACGTTAGACCTAAAACCATAAAAACCCTAGAAGAAAACCTAGGCAATACCATTCAGGACATAGGCATGGGCAAGGACTTCATGTCTAAAACACCAAAAGCAATGGCAACAAAAGCCAAAATTGACAAATGGGATCGAATTAAACTAAAGAGCTTCTGCACAGCAAAAGAAACTACCATCAGAGTGAACAGGCAACCTACAGAATGGGAGAAAATGTTTACAATCTATCCATCTGACAAAGGGCTAATATCCAGAATCTACAATGAACTCAAACAGATTTACAAGAAAAAAACAAACAACCCCATCAAAAAGTGGGCGAAGGACATGAACAGACACTTCTCAAAAGAAGACATTTATGCAGCCAAAAAACACATGAAAAAATGCTCACCATCACTGGCTGTCAGAGAAATGCAAATCAAAACCACAATGAAATGCCATCTCACACCAGTTAGAATGGCAATCATTAAAAAATCAGGTAACAACAGGTGCTGGAGAGCATGTGGAGAAATAGGAACACTTTGACACTGTTGGTGGGACTGTAAACTAGTTCAACCATTGTGGAAGTCGGTGTGGTGATTCCTCAGGGATCTAGAACTAGAAATACCATTTGACCCAGCCATCCCATTACTGGATATATACCCAAAGGACTATAAATCATGCTGCTATAAAGACACATGCACACGTATGTTTATTGCGGCACTATTCACAAGAGCAAAGACTTGGAACCAACCCAAATGTCCGACAATGATAGACTGGATTAAGAAAATGTGGCACATATACATCATGGAATACTATGCAGCCATAAAAAAGGGTGAGTTCATGTCCTTTGTAGGGACATGGATGAAACTGGAAATCATCATTCTCAGTAAACTATCACAAGAACAAAAAACCAAACACTGCATATTCTCACTCATAGGTGGGAATTGAACAATGAGAACACATGGACACAGGAAGGGGAACATCACACTCTGGGGACTGTTGTGGGGTGGGGGGAGGGGGGAGGGATAGCATTAGGAGATATACCTAATGCTAAATGATGAGTCAATGGGTGCAGCACACCATCATGGCACTTGTATACATATGTAACTAACCTGCACATTGTGCACATGTACCCTAAAACTTAAAGTATAAT*AATAATAAAATAAAATAAAAATTTAAAAAAAGAAAATAACAAAA****AAAAA*TATGTTATA**AATTGCATTCACCTTCAATCAAAAGGGCCTGACAACTGAAGGCTGTCAATTAAACAATAGCAGTGGCA

**Clone 11; PA3; SpIRE(97/622)-11: Filled site spans: chr1:228707260-228712921**

Empty Site:

AAAATATATATATATACATATATATACATATATATATACATATATATATACATATGTATATATATATATATATATAGTGTGTGTGTGTGTGTGTGTGTGTGTGTGTGTGTGTGTGTGTGT

GT**ATAAAATACCCAAAGC**TGGAGTTTGCATCTCCTGCA

ATGTTGCCATATACTGCCAGGGTCTGCCTGACCTAGTCGGATATCTGAGTCTGACTATATATATATAAAGGTATCGATTAATTTCTTTTTTCTTCCTTTTTTTTTTAACCAGGA

Filled Site:

GTGTGTGTGTGTGTGTGTGTGTGTGTGTGTGTGTGTGTGTGT**ATAAAATACCCAAAGC**AGAATGGTCTCGCAAGATGGCCGAATAGGAACAGCTCCGGTCTACAGCTCCCAGCGTGAGCGACACAGAAGATGGGTGATTTCTGCATTTCCATCTGAGCTTTGAAGAGAGCAGTGGTTCTCCCAGCACGCAGCTGGAGATCTGAGAACGGGCAGACTGCCTCCTCAAGTGGGTCCCTGACCCCTGACCCCCGAGCAGCCTAACTGGGAGGCACCCCCCAGCAGGGGCAGACTGACACCTCACACGGCCGGGTACTCCAGCAGACCTGCAGCTGAGGGTCTTGTCTGTTAGAAAGAAAACTAGCAAACAGAAAGGACATCCACACCAAAAACCCATCTGTACATCACCATCATCAAAGACCAAAAGTAGATAAAACCACAAAGATGGAGAAAAACCAGAGCAGAAAAACTGGAAACTCTAAAAAGCAGAGTGCCTCTCCTCCTCCAAAGGAATGCAGTTCCTCACCAGCAACGGAACAAAGCTGGACAGAGAATGACTTTGACAAGCTGAGAGAAGAAGGCTTCAGACAATCAAATTACTCCGAGCTATGGGAAGACATTCAAACCAAAGGCAAAGAAGTTGAAAACTTTGAAAAAAATTTAGAAGAATATATAACTAGAATCACCAATACAGAGAAGTGCTTAAAGGAGCTGATGGAGCTGAAAACCAAGGCTCGAGAACTACATGAAGAATGCAGAAGCCTCAGGAGCTGATGTGATCAACTGGAAGAAAGGGTATAAGTGATGGAAGATGAAATGAATAAAATGAAGGGAGAAGGGAAGTTTAGAGAAAAAAGAATAAAAAGAAACGAGCAAAGCGTCCAAGAAATATGGGACTATGTGAAAAGACCAAATCTACGTCTGATTGGTGTACCTGAAAGTGACGGGGAGAATGGAACCAAGTTGGAAAACACTCTGCAGGGTATTATCCAGGAGAACTTCCCCAATCTAGCAAGGCAGGCCAACATTCAGATTCAGGAAATACAGAGAACGCCACAAAGATACTCCTCGAGAAGAGCAACACCAAGACACATAATTGTCAGATTCACCAAAGTTGAAATGAAGGAAAAAATGTTAAGGGCAGCCAGAGAGAAAGGTCGGGTTACCCTCAAAGGGAAGCCCATCAGACTAACAGCAGATCTCTCGGCAGAAACTCTACAAGCCAGAAGAGAGTGGGGGCCAATATTCAACATTCTTAAAGAAAAGAATTTTCAACCCAGAATTTCATATCCAGCCAAACTAAACTTCATAAGTAAAGGAGAAATAAAATACTTTACAGACAAGCAAATACTGAGAGATTTTGTCACCACCAGGCCTGCCCTAAAAGAGCTCCTGAAGGAAGCACTAAACATGGAAAGGAACAACCGGTACCAGCCGCTGCAAAATCATGCCAAAATGTAAAGACCATCGAGACTAGGAAGAAACTGCATCAACTAACAAGCAAAACAACCAGCTAACATCATAATGACAGGATCAAATTCACACATAACAATATTAACTTTAAGTGTCAATGGACTAAATGCTCCAATTAAAAGACACAGACTGTCAAATTGGATAAAGAGTCAAGACCCATCAGTGTGCTGTATTCAGGAAACCCATCTCATGGGCAGAGACACACATAGGCTCAAAATAAAAGGATGGAGGAAGATCTACCAAGAAAATGGAAAACAAAAAAAGGAAGGGGTTGCAATCCTAGTCTCTGATAAAACAGACTTTAAACCAACAAAGATCAAAAGAGACAAAGAAGGCCATTACTTAATGGTAAAGGGATCAATTCAACAAGAAGAGCTAACTATCCTAAATATATATGCACCCAATACAGGAGCACCCAGATTCATAAAGCAAGTCCTGAGTGACCTATAAGGAGACTTAGACTCCCACACATTAATAATGGGAGACTTTAACACCCCACTGTCAACATTAGACAGATCAACGAGACAGAAAGTCAACAAGGATACCCAGGAATTGAACTCAGCTCTGCACCAAGCAGACCTAATAGACATCTACAGAACTCTCCACCCCAAATCAACAGAATATACATTTTTTTCAGCACCACACCACACCTATTCCAAAATTGACCACATAGTTGGAAGTAAAGCTCTCCTCAGCAAATGTAAAAGAACAGAAATTATAACAAACTATCTCTCAGACCACAGTGCAATCAAACTGGAACTCAGGATTAAGAATCTCACTCAAAACCACTCAACTACATGGAAACTGAACAACCTGCTCCTGAATGACTACTGGGTACATAACGAAATGAAGGCAGAAATAAAGATGTTCTTTGAAACCAACGAGAACAAAGACACAACATACCAGAATCTCTGGGACACATTCAAAGCAGTGTGTAGAGGGAAATTTATAGCACTAAATGCCCACAAGAGAAAGCAGGAAAGATCCAAAATTGACACCCTAACATCACAATTAAAAGAACTAGAAAAGCAAGAGCAAACATATTCAAAAGCTAGCAGAAGGCAAGAAATAACTAAGATCAGAGCAGAACTGAAGGAAATAGAGACACAAAAAACCCTTCAAAAAATTAACGAATCCAGGAGCTGGTTTTTTGAAAGGATCAACAAAATTGATAGACCGCTAGCAAGACTAATAAAGAAAAAAAGAGAGAAGAATCAAATAGGAGCAATAAAAAATGATAAAGGGGATATCACCACCAATTCCACAGAAATACAAACTACCATAAGAGAATACTACAAACACCTCTATGCAAATAAACTAGAAAATCTAGAAGAAATGGATAAATTCCTTGACATATACACTCTCCCAGGACTAAACCAGGAAGAAGTTGAATCTCTGAATAGACCAATAATAGGAGCTGAAATTGTGGCAATAATCAATAGCTTACCAACCAAAAAGAGTCCAGGACCAGATGGATTCACAGCCAAATTCTACCACAGGTACAAGGAGGAACTGGTACCATTCCTTTTGAAACTATTCCACTCAATAGAAAAAGAGTAAATCCTCCCTAACTCATTTTATGAGGCCAGCATCATCCTGATACCAAAGCCGGGCAGAGACACAACCAAAAAAGAGAATTTTAGACCAATATCCTTGATGAACATTGATGCAAAAATCCTCAATAAAATACTGGCAAACTGAATCCAGCAGCACATCAAAAAGCTTATCCACCATGATCAAGTGGGCTTCATCCCTGGGATGCAAGCCTGGTTCAATATACGCAAATCAATAAATGTAATCCAGCATATAAACAGAACCAAAGATAAAAACCACATGATTATCTCAACAGATGCAGAAAAGGCCTTTGACAAAATTCAACAACCCTTCATGCTAAAAACTCTCAATAAATTAGGTATTGATGGGACATATCTCAAAATAATAAGAGCTATCTATGACAAACCCACAGCCAATATCATACTGAATGGGCAAAAACTGGAAGCATTCCCTTTGAAAACTGGCACAAGACAGGGATGCCCTCTCTCACCACTCCTATTCAACATAGTGTTGGAAGTTCTGGCCAGGGCAATGAGGCAGGAGAAGGAAATAAAGGGCATTCAATTAGGAAAAGAGGAAGTCAAATTGTACCTGTTTGCAGACGACATGATTGTATATCTAGAAAACCCCATTGTCTCAGCCCAAAATCTCCTTAAGCTGATAAGCAACTTCAGCAAAGTCTCAGGATACAAAATCAATGTACAAAAATCACAAGCATTCTTATACACCAACAGACAAACAGAGAGCCAAATCATGAGTGAACTCCCATTCACAATTGCTTCAAAGAGAATAAAATACCTAGAAATCCAACTTACAAGGGATGTGAAGGACCTCTTCAAGGAGAACTAAAAACCACTGCTCAAGGAAATAAAAGAGGATACAAACAAATGGAAGAACATTCCATGCTCATGGGTAGGAAGAATCAATATCGTGAAAATGGCCATACTGCCCAAGGTAATTTACAGATTCAGTGCCATCCCCATCAAGCTACCAATGACTTTCTTCACAGAATTGGAAAAAACTACTTTAAAGTTCATATGGAACCAAAAAAGAGCCCGCATCGCCAAGTCAATCCTAAGCCAAAAGAACAAAGCTGGAGGCGTCACACTACTTGACTTCAAACTATACTACAAGGCTACAATAACCAAAAAAGCATGGTACTGGTACCAAAACAGAGATATAGATCAATGGAACAGAACAGAGCCCTCAGAAATAACGCCGCATATCTACAACTATCTGATCTGTGACAAACCTGACAAAAACAAGCAATGGGGAAAGGATTCCCTATTTAATAAATGGTGCTGGGAAAACTGGCTAGCCATATGTAGAAAGCTGAAACTGGATCCTTTCCTTACACCTTATACAAAAATTAATTCAAGATGGGTTAAAGACTTAAACGTTAGACCTAAAACCATAAAAACCCTAGAAGAAAACCTAGGCATTACCATTCAGGACATAGGCATGGGCAAGGACTTCATGTCTAAAACACCAAAAGCAATGGCAACAAAAGCCAAAATTGACAAATGGGATCTAATTAAACTAAAGAGCTTCTGCACAGCAAAAGAAACTACCATCAGAGTGAACAGGCAACCTACAAAATGGGAGAAAATTTTCACAACCTACTCATCTGACAAAGGGCTAATATCCAGAATCTACAATGAACTCAAACAAATTTACAAGAAAAAAACAAACAACCCCATCAAAAAGTGGGCAAAGGACATGAACAGACACTTCTCAAAAGAAGACATTTATGCAGCCAAAAAACACATGAAAAAATGCTCACCATCACTGGCCATCAGAGAAATGCAAATCAAAACCACAATGAGATACCATTTCACACCAGTTAGAATGGCAATCATTAAAAAGTCAGGAAATAACAGGTGCTGGAGAGGATATGGAGAAATAGGAACACTTTTACACTGTTGGTGGGACTGTAAACTAGTTCAACCATTGTGGAAGTCAGTGTGGCGATTCCTCAGGGATCTAGAACTAGAAATACCATTTGACCCAGCCATCCCATTACTGGGTATATACCCAAAGGACTATAAATCATGCTGCTATAAAGACACATGCACATGTATGTTTATTGCGGCATTATTCACAATAGCAAAGACTTGGAACCAACCCACACGTCCAACAATGATAGACTGGATTAAGAAAATGTGGCACATATACACCATGGAATACTATTCAGCCATACAAAATGATGAGTTCATGTCCTTTGTAGGGACATGCATGAAATTGGAAATCATCATTCTCAGTAAACTATCGCAAGAACAAAAAACCAAAAACCGCATATTCTCACTCATGGGTGGGAGTTGAACAATGAGAACACATGGACACAGGAAGGGGAACATCACACTCTGGGGACTGTTGTAGGGTGGGGGGAGGGGGGAGGGATAGCATTGGGAGATATACCTAATGCTAGATGACGAGTTAGTGGGTGCAGCGCACCACCATGGCACATGCATACATATGTAACTAACCTGCACATTGTGCACATGTACCCTAAAACTTAAAGTATAAT*AATAATAAATAAATAAATAAATAAAATAAAATAAAATAAAATAAAATAAAATAAAATAAA****ATAAAATA*CCCAAAGC**TGGAGTTTGCATCTCCTGCAATGTTGCCATATACT

**Clone 12; PA3; SpIRE(97/622): Filled spans: chr1:71215791-71221424**

Empty Site:

TCTTGTCTTCTAAGATACTGCTTCTATCTCTGTGTGTCCTCTTTAATATCTAATAAAACTAAAGTGGATCAGATGATCAACAGCATTACATAAATTCTATATATCAGATGAGGAAAAGTG

AAGGTAAACCCAAAAAGCT**AAGAAACAATGTAA**TGCAG

ATTAGCACCAGATGGGCCCTCCAAAGTTAGAAAAATGAAGGGTGTTAGAAGGTACATTGAAAAGGGAAATGAAAAAAGGAGAGAGGCACAAATGAAAGCAAACTAACTCCAAGTTTC

Filled Site:

AAAGTGAAGGTAAACCCAAAAAGCT**AAGAAACAATGTAA**GGGAGGAGCTAAGATGGCCGAAAAGGAACAGCTCCGGTCTACAGCTCCCAGCGTGAGCGACGCAGAAGATGGGTGATTTCTGCATTTCCATCTGAGCTTTGAAGAGAGCAGTGGTTCTCCCAGCACGCAGCTGGAGATCTGAGAATGGGCAGACTGCCTCCTCAAGTGGGTCCCTGACCCCTGACCCCCGAGCAGCCTAACTGGGAGGCACCCCCCAGCAGGGGCAGACTGACACCTCACATGGCTGGGTACTCCAACACACCTGCAGCTGAGGGTCCTGTCTGTTAGAAGGAAAACTAACTAACAGAAAGGACATCCACACCAAAAACCCATCTGTACATCACCATCATCAAAGACCAAAAGTAGATAAAACCACAAAGATGGGGAAAAAACAGAGCAGAAAAACTGGAAACTCTAAAAAGCAGAGCGCCTCTCCTCCTCCAAAGGAACGCAGTTCCTCACCAGCAACGGAACAAAGCTGGACGGAGAATGACTTTGACGAGCTGAGAGAAGAAGGCTTCAGACGATCAAATTACTCTGAGCTACGGGAGGAAATTCAAACCAAAGGCAAAGAAGTTGAAAACTTTGAAAAAAGTTTAGAAGAATGTATAACTAGAATAACCAATACAGAGAAGTGCTTAAAGGAGCTGATGGAGCTGAAAACCAAGGCTCGAGAACTACCTGAAGAATGCAGAAGCCTCAGGAGCCGATGCGATCAACTGGAAGAAAGGGTATCAGCGATGGAAGATGAAGTGAATGAAATGAAGTGAGAAGGGAAGTTTAGAGAAAAAAGAATAAAAAGAAATGAGCAAAGCCTTCAAGAAATATGGGACTAGTGAAAAGACCAAATCTACGTCTGATTGGTGTACCTGAAAGTGACGGGGAGAATGGAACCAAGTTGGAAAACACTCTGCAGGATATCATCCAGGAGAACTTCCCCAATCTAGCAAGGCAGGCCAACATTCAGATTCAGGAAATACAGAGAACTCCACAAAGATACTCCTCGAGAAGAGCAACTCCAAGACACATAATTGTCAGATTCACCAAAGTTGAAATGAAGGAAAAAATGTTAAGGGCAGCCAGAGAGAAAGGTCGGGTTACCCACAAAGGGAAGCCCATCAGACTAACAGCGGATCTCTCGGCAGAAACTCTACAAGCCAGAAGAGAGTGGGGGCCAATATTCAACATTCTTACAGAAAAGAATTTTCAACCCAGAATTTCATATCCAGCCAAACTAAGCTTCATAAGTGAAGGAGAAATAAAATCCTTTACAGACAAGCAAATGCTGAGAGATTTTGTCACCACCAGGCCTGCCCTAAAAGAGCTCCTGAAGGAAGCACTAAACATGGAAAGGAAAAACCGGTACCAGCCGCTGCAAAATCATGCCAAAATGTAAAGACCATCGAGACTAGGAAGAAACTGCATGAACTAACGAGCAAAATAACCAGCTAACATCATAATGACAGGATCAAATTCACACATAACAATATTAACTTTAAATGTAAACAGACTAAATGCTCCAATTAAAAGACACAGACTGGCAAATTGGATAAAGAGTCAAGACCCATCAGTGTGCTGTATTCAGGAAAGCCATCTCACGTGCAGAGACACACATAGGCTCAAAATAAAAGGATGGAGGAAGATCTACCAAGCAAATGGAAAACAAAAAAAGGCAGGGGTTGCAATCCTAGTCTCTGATAAAAGAGACTTTAAACCAACAAAGATCAAAAGAGACAAAGAAGGCCATTACATAATGGTAAAGGGATCAATTCAAGAAGAAGAGATAACTATCCTAAATATATATGCACCCAATACAGGAGCACCCAGATTCATAAAGCAAGTCCTGAGCGACCTACAAAGAGACTTAGACTCCCACACATTAATAATGGGAGACTTTAACACCCCACTGTCAACATTAGACAGATCAACGAGACAGAAAGTCAACAAGGATACCCAGGAATTGAACTCAGCTCTGCACCAAGCAGACCTAATAGACATCTACAGAACTCTTCACCCCAAATAAACAGAATATACATTTTTTTCAGCACCACACCACACCTATTCCAAAATTGACCACATACTTGGAAGTAAAGCTCTCCTCAGCAAATGTAAAAGAACAGAAATTATAACAAACTATCTCTCAGACCACAGTGCAATCAAACTAGAACTCAGGATTAAGAATCTCACTCAAAACCGCTCAACTACATGGAAACTGAACAACCTGCTCCTGAATGACTACTGAGTACATAACGAAATGAAGGCAGAAATAAAGATGTTCTTTAAAACCAATGAGAACAAAGACACAACATACCAGAATCTCTGGGACACATTCAAAGCAGTGTGTAGAGGGAAATTTATAGCACTAAATGCCCACAAGAGAAGGCAGAAAAGATCCAAAATTGACACCCTAACATCACAATTAAAAGAACTAGAAAAGCAAGAGCAAACACATTCAAAAGCTAGCAGAAGGCAAGAAATAACTAAAATCAGAGCAGAACTGAAGGAAATAGAGACACAAAAAACCCTTCAAAAAATTAATGAATCCAGGAGCTGGTTTTTTGAAAGGATCGACAAAATTGATAGACCGCTAGCAAGACTAATAAAGAAAAAAAGAGAGAAGAATCAGATAGACGCAATAAAAAATGATAAAGGGGATATCACCACCGATCCCACAGAAATACAAACTACCATCAGAGAATACTACAAACACCTCTATGCAAATAAACTAGAAAATCTAGAAGAAATGGATAAATTCCTGGACACATACACCCTGCCAAGACTAAACCAGGAAGAAGTTGAATCTCTGAATAGACCAATAACAGGATCTGAAATTGTGGCAATAATCAATAGCTTACCAACCAAAAAGAGCCCAGGACCAGATGGATTCACAGCCGAATTCTACGAGAGGCACAAGGAGGAACTGGTACCATTCCTTCTGAAACTATTCCAATCAATAGAAAAAGAGGGAATCATCCCTAACTCATTTTATGAGGCCAGCATCATCCTGATACCAAAGCCGGGCAGAGACACAACAAAAAAAGAGAATTTAGACCAATATCCTTGATGAACATTGATGCAAAAATCCTCAATAAAGTACTGGCAAACCGAATCCAGCAGCACATCAAAAAGCTTATCCACCATGATCAAGTGGGCTTCATCCCTGGGATGCAAGGCTGGTTCAATATATGCAAATCAATAAATGTAATCCAGCATATAAACAGAACCAAAAACAAAAACCACATGATTATCTCAATAGATGCAGAAAAGGCCTTTGACAAAATTCAACAACCCTTCATGCTAAAAACTCTCAAGAAATTACGTATTGATGGGACGTATCTCAAAATAATAAGAGCTATCTATGACAAACCCACAGCCAATATCATACTGAATGGGCAAAAACTGGAAGCATTCCCTTTGAAAACTGGCACAAGACAGGGATGCCCTCTCTCACCACTCCTATTCAACATAGTGTTGGAAGTTCTGGCCAGGGCAGTTAGGCAGGAGAAGGAAATAAATGGTATTCAATTAGGAAAAGAGGAAGTCAAATTGTCCCTGTTTGCAGACGACATGATTGTATATCTAGAAAACCCCATTGTCTCAGCCCAAAATCTCCTTAAGCTGATAAGCAACTTCAGCAAAGTCTCAGGATACAAAATCAATGCAGAAAAATCACAAGCCTTCTTATACACCAACAACAGACAAACAGAGAGCCAAATCATGAGTGAACTCCCATTCACAATTGCTTCAAAGAGAATAAAATACCTAGGAATCCAACTTACAGGGGATGTGATGGACCTCTTCAAGGAGAACTACAAACCACTGCTGAAGGAAATAAAAGAGGATACAAACAAATGGAAGAACATTCCATGCTCATGGGTAGGAAGAATCAATATCGTGAAAATGGCCATACTGTCCAAGTAACTTATAGATTCAATGCCATCCCCATCAAGCTACCAATGACTTTCTTCACATAATTGGAAAAAACTACTTTAAAGTTCATATGGAACCAAAAAAGAGCCTGCATCGCCAAGTCAATCCTAAGCCAAAAGAACGAAGCTGGAGGCATCACGCTACCTGACTTCAAACTATACTACAAGGCTACAGTAACCAAAACAGCATGTTACTGGTACCAAAACAGAGTTATAGATCAATGGAACAGAACAGAGCCCTCAGAAATAACGCCACATATCACAACTATCTGATCTTTGACAAACCTGAGAAAAACAAGCAATGGGAAAGGATTCCCTATTTAATAAATGGTGCTGGGAAAACTGGCTAGCCATATGTAGAAAGCTGAAACTGGATCCCTTCCTTACACCTTATACAAAAATCAATTCAAGATGGATTAAAGATTTAAACGTTAGACCTAAAACCATAAAAACCCTAGAAGAAAACCTAGGCATTACCATTCAGGACATAGGCATGGGCAAGGACTTAATGTCTAAACCACCAAAAGCAATGGCAACAAAAGCCAAAATTGACAAATGGGATCTAATTAAACTAAAGAGCCTCTGCACAGCAAAAGAAACTACCATCAGAGTGAACAGGCAACCTACAAAATGGGAGAAAATTTTCGCAACCTACTCATCTGACAAAGGGCTAATATCCAGCATCTACAATGAACTCAAACAAATTTACAAGAAAAAAACAAACAACGCCATCAAAAAGTGGGCGAAGGACATGAACAGACACTTCTCAAAAGAAGACATTTATGCAGCCAAAAACCACATGAAAAAATGCTCACCATCACTGGCCATCAGGGAAATGCAAATCAAAACCACAATGACATACCATCTCACACCAGTTAGAATGGCAATCATTAAAAAGTCAGGAAACAACAGGTGCTGGAGAGGATGTGGAGAAATAGGAACACTTTTACACTGTTGGTGGGACTGTAAACTAGTTCAATCATTGTGGAAGTCAGTGTGGCGATTCCTCAGGGATCTGGAACTAGAAATACCATTTGACCCAGCCATCCCATTACTGGGTATATACCCAAAGGACTATAAATCATGCTGCTATAAAGACACATGCACACATATGTTTATTGCGGCATTATTCACAATAGCAAAGACTTGGAACCAAGCCAAATGTCCAACAATGATAGACTGGATTAAGAAAATATGGCACATATACACCATGGAATACTATGCAGCCATAAAAAATGATGAGTTCACATCCTTTGTAGGGACATGGATGAAATTGGAAATCATCATTCTCAGTAAACTATCACAAGAACAAAAAACCAAACACCACATATTCTCACTCATAGGTGGGAATTGAACAATGAGAACACGTGGACACAGGAAGGGGAACATCACACTCTGGGGACTGTTGTGGGGTGGTGGGAGGGGGGAGGGATAGCATTGGGAGATATACCTAATGCTAGATGACGAGTTAGTGGGTGCAGTGCACCAGCATGGCACATGTATACATATGTAACTAACCTGCACATTGTGCACATGTACCCTAAAACTTAAAGTATAATAAT*AATAAATTAAAAAAATAAAAAAAAAGA****AAGAAACAATGTAA***TGCAGATTAGCACCAGATGGGCCCTCCAAAGTTAGAAAAATGAAGGGTGTTAGAAGGTACATT

**Clone 13; PA3; SpIRE(97/622)-13: Filled spans: chr1:37207908-37214758**

Empty Site:

TTTTCAGTCAATTGGCTTTTGACAAAGGTGCCAAGAACACAATTGGAAGAGAAAGGACAGTATCTTCAATAAATGGTGCTGGAAAAACTGAATATACACATGCAGAGGAATGAAAATGCA

CTCATATCTCACCCTTTATAC**AAGAATCAATTCA**AAATGA

ATTATACAAAAATTAGCTGGGCATGGTGACAGTTTCCTGTAATCCCAGCTACTGGAGAGGCTGAGGCAGGAGAATTGTTTAAACCCTGGAGGCAGAGGTTGCAGTGAGCTGAGATCGTCC

Filled Site:

CTCATATCTCACCCTTTATAC**AAGAATCAATTCA**GAGGAGCCAAGATGGCCGAATAGGAACAGCTCCGGTCTACAGCTCCCAGCATGAGCAACGCAGAAGATGGGTGATTTCTGCATTTCCATCTGAGCTTTGAAGAGAGCAGTGGTTCTCCCAGCACGCAGCTGGAGATCTGAGAATGGGCAGACTGCATCCTCAAGTGGGTCTCTGACCCCTGACCCCTGAGCAGCCTAACTGGGAGGCACCCCCCAGTAGCGGCAGACTGACACCTCACACAGCCAGGTACTCCTCAGAGACAAAACTTCCAGAGGAACGATCAGACAGCAGCATTCACGGTTCACAAAAATCCGCTGTTCTGCAGACACTGCTGCTGATACCCAGGCAAACAGCGTCTGGAGTGGACCTCTAGCAAACTCCAACAGACCTGCAGCTGAGGGTCCTGTCTGTTAGAAGGAAAACTAACAAACAGAAAGGACATCCACACCAAAAACCCATCTGTACATCACCATCATCAAAGACCAAAAGTAGATAAAACCACAAAGATGGGGAAAAAACAGAGCAGAAAAACTGGAAACTCTAAAAAGCAGAGCGCCTCTCCTCCCCCAAAGGAACGCAGTTCCTCACCAGCAACAGAACAAAGCTGGACGGAGAATGACTTTGACGAGTTGAGAGAAGAAGTTTTCAGACGATCAAACTACTCCGAGCTACAGGAGGAAATTCAAACCAAAGGCAAAGAAGTTGAAAACTTTGAAAAAAAAAATTTAGACAAATGTATAACTAGAATAACCAACACAGAGAAGTGCTTAAAGTTGCTGATGGAGCTGAAAGCCAAGGCTCGAGAACTACGTGAAGAATGCAGAAGCCTCAGGAGCTGATGCAATCAACTGGAAGAAAGGGTATCAGTGATGGAAGATGAAATGAATGAAATGAAGCAAGAAGGGAAGTTTAGAGAAAAAAGAATAAAAAGAAACGAACAAAGCCTCCAAGAAATATGGGACTATGTGAAAAGACCAAATCTGCGTCTGATTGGTGTACCTGAAAGTGACGGGGAGAATGGAACCAAGTTGGAAAACACTCTGCAGGATATTATCCAGGAGAACTTCCCCAATCTAGCAAGGCAGACCAACATTCAGATTCAGGAAATACAAAGAATGCCACAAAGATACTCCTCGAGAAGAGCAACTCCAAGACACATAATTGTCAGATTCACCAAAGTTGAAATGAAGGAAAAAATGTCAAGGGCAGCCAGAGAGAAAGGTTAGGTTACCCACAAAGGGAAGCCCATCAGACTAACAGTGGATCTCTCGGCAGAAACTCTACAAGCCAGAAGAGAGTGAGGGCCAATATTCAACATTCTTAAAGAAAAGAATTTTCAACCCAGAATTTCATATCCAGACAAACTAAGCTTCATAAGTGAAGGAGAAATAAAATCCTTTACAGACAAGCAAATGCTGAGAGATTTTGTCACCACCAGGCCTGCCCTAAAAGAGCTCCTGAAGGAAGCACTAAACATGGAAAGGAACAACCAGTACCAGCCGCTGCAAAATCATGCCAAAATGTAAAGACCATCGAGACTAGGAAGAAACTGCATGAACTAACGAGCAAAATAACCAGCTAACATCATAATGACAGGATCAAACTCACACATAACAATATTAACTTTAAATGTAAATGGACTAAATGCTCCAATTAAAAGACACAGACTGGCAAATTGGATAAAGAGTCAAGACCCGTCAGTGTGCTGTATTCAGGAAACCCATCTCATGTGCAGAGACAAACATAGGCTCAAAATTAAAGTATGGAGGAAGATCTACCATGCAAATGGAAAACAAAAAAGGCAGGGGTTGCAATCCTAGTCTCTGATAAAACAGACTTTAAACCAACAAAGATCAAAAGGGACAAAGAAGGCCATTACATAATGGTAAAGAGATCAATTCAACAAGAAGAGCTAACTATCCTAAATATATATGCACCCAATACAGGAGCACCCAGATTCATAAAGCAAGTCCTGAGTGACCTACAAAGAGACTTAGACTCACACACAGTAATAATGGGAGACTTTAACACCCCACTGTCAACATTAGACAGATCAACGAGACAGAAAGTTAACAAGGATACCCAGGAATTGAACTCAGCTCTGCACCAAGCAGACCTAATAGACATCTACAGAACTCTTCACCCCAAATCAACAGAATATACATTTTTTTCAGCACCACACCACACCTATTCCAAAATTGACCACATAGTTGGAAGTAAAGCTCTCCTCAGCAAATGTAAAAGAACAGAAATTATAACAAACAGTCTCTCAGACCACAGTGCAATCAAACTAGAACTCAGGATTAAGAAACTCACTCAAAACCACTCAACTACATGGAAACTGAACAACCTGCTCCTGAATGACTACTGGGTACATAACGAAATGAAGGCAGAAATAAAGATGTTCTTTGAAACCAATGAGAACAAAGACACAACATACCAGAATCTCTAGGACGCATTCAAAGCAGTGTGTAGAGGGAAATTTATAGCACTAAATGCCCACAAGAGATAGCAGGAAAGATCCAAAATTGACACCCTAACATCACAATTAAAAGAACTAGAAAAGCAAGAGCAAACACATTCAAAAGCTAGCAGAAGGCAAGAAATAACTAAAATCAGAGCAGAACTGAAGGAAATAGAGACACAAAAAACCCTTCAAAAAATTAATGAATCTAGGAGCTGGTTTCTTGAAAGGATCAACAAAATTGATAGACCGCTAGCAAGACTAATAAAGAAAAAAAGAGAGAAGAATCAAATAGACACAATAAAAAATGATAAAGGGGATATCACCACCAATCCCACAGAAATACAAACTACCATCAGAGAATACTACAAACACCTCTACGCAAATAAACTAGAAAATCTAGAAGAAATGGATAAATTCCTCGACACATGCACTCTCCCAAGACTAAACCAGGAAGAAGTTGAATCTCTGAATAGAACAGTAACAGGATCTGAAATTGTGGCAATAATCAATAGCTTACCAACCAAAAAGAGTCCAGGACCAGATGGATTCACAGCCGAATTCTACCAGAGGTACAAGGAGGAACTGGTACCATTCCTTCTGAAACTATTCCAATCAATAGAAAAAGAGGGAATCCTCCCTAACTCATTTCATGAGGCCAGCATCATCCTGATACCAAAGCCAGGCAGAGACACAACCAAAAAAGAGAATTTTAGACCAATATCCTTGATGAACATTGATGCAAAAATCCTCAGTAAAATACTGGCAAACCAAATCCAGCAACACATCAAAAAGCTTATCCACCATGATCAAGTGGGCTTCATCCCTGGGATGCAAGGTTGGTTCAATATACACAAATCAATAAATGTAATCCAGCATATAAACAGAACCAAAGACATGATTATCTCAATAGATGCAGAAAAGGCCTTTGACAAAATTCAACAACGCTTCATGCTAAAAACTCTCAATAAATTAGGTATTGATGGGACGTATCTCAAAATAATAAGAGCTATCTATGACAAACCCACAGCCAATATCATACTGAATGGGCAAAAACTGGAAGCATTCCCTTTGAAAACTGGCACAAGACAGGGATGTCCTCTCTCACCACTCCTATTCAACATAGTGTTGGAAGTTCTGGCCAGGGCAATTAGGCAGGAGAAGGAAATAAAGGGTATTCAATTAGGAAAAGAGGAAGTCAAATTGTCCCTGTTTGCAGACGACATGATTGTATATCTAGAAAACTCCATTGTCTCAGCCCAAAATCTCCTTAAGCTGATAAGCAACTTCAGCAAAGTCTCAGGATACAAAATCAATGCACAATAATCACAAGCATTCTTATACACCAATAACAGACAATCAGAGAGCCAAATCATGAGTGAATTCCCATTCACAATTGCTTCAAAGAGAATAAAATACCTAGGAATCCAACTTACAAGGGATGTGAAGGATCTCTTCAAGGAGAACTACAAACCACTGCTCAATGAAATTAAAGAGGATACAAAGAAATGGAAGAACATTCCATGCTCATGGGTAGGAAGAATCAATATCGTGAAAATGGCCATACTGCCCAAGGTAATTTATACATTCAATGCCATCCCCATCAAGCTACCAATGACTTTCTTCACAGAATTGGAAAAAACTACTTTCAAGTTCATATGGAATCAAAAAAGAGCCCACGTCGCCAAGTCAATCCTAAGCCAAAAGAACAAAGCTGGAGGCATCACGCTACCTGACTTCAAACTATACTACAAGGCTACAGTAACCAAAACAGCATGGTACTGGTACCAAAACAGAGATATAGATCAATGGAACAGAACAGAGCCCTCAGAAATAATGCTGCATATCTACAGCTATCTGATCTTTGACAAACCTGAGAAAAACAAGCAATGGGGAAAGGATTCCCTATTTAATAAATGGTGCTGGGAAAACTGGCTAGCCATATGTAGAAAGCTGAAACTGGATCCCTTCCTTACACCTTATACAAAAATTAATTCAAGATGGATTAAAGACTTAAACATTAGACCTAAAACCATAGAAACCCTAGAAGAAAACCTAGGCATTACCATTCAGGACATAGGCATGGGCAAGGACTTCATGTCTAAAACATCAAAAGCAATGTCAACAAAAGCCAAAATTGACAAATGGGATCTAATTAAACTAAAGAGCTTCTGCACAGCAAAAGAAACTACCATCAAAGTGAACAGGCAACCTACAAAATGGGAGAAAATTTTCGCAACCTACTCATCTGACAAAGGGCTAATATCCAGAATCTACAATGAACTCAAACAAATTTACAAGAAAAAAAACAAACAACCCCATCAAAAAGTGGGTGAAGGATATGAACAGACACTTCTCAAAAGAAGACATCTATGCAGCCAAAAGACACATGAAAAAATGCTCATCATCACTGGCCATCAGAGAAATGCAAATCAAAACCACAATGAGATACCATCTCACACCAGTTAGAATAGCAATCATTAAAAAGTCAGGAAACAACAGGTGCTGGAGAGGATGTGGAGAAATAGGAACACTTTTACACTGTTGGTGGGACTGTAAACTAGTTCAACCATTGTGGAAGTCAGTGTGGCAATTCCTCAGGGATCTAGAACTAGAAATACCATTTGACCCAGCCATCCCATTACTTGGTATATATCCAAAGGACTATAAATCATGCTGCTATAAAGACACATGCACACGTATGTTTATTGCGGCACTATTGACAATAGCAAAGACTTGGAACCAACCCAAATGTCCAACAATGATAGACTGGATTAAGAAAATATGGCACATATACACCATGGAATACTATGCAGCCATAAAAAATGATGAGTTCATGTCCTTTGTAGGGACATGAATGAAATTGGAAATCATCATTCTCAGTAAATTATCTCAAGGACAAAAAGCCAAACATCACATGTTCTCACTCATAGATGGGAATTGAACAATGAGAACACATGGACACAGGAAGGGGAACATCACTTTCATGTGCGTCCGTGTGAAAAGACCACCAAACAGGCTTTGTGTGAGCAACATGGCTATTTATTTCACCTGGGTGCAGGTGGGCTGAGTCTGAAAAGAGTCAGCAAAGGGAGATAGGGGTGGGGCTGTTTTATAGGATTAGGGAAGGTAATGGAAAATTACAGTCAAAGGGGGTTGTTCTCTGGTGGGCAGGGGCGGGGGTCACAAGATGCTCAGTGGGGGAGCTTCTGAGCCAGAAGGAAATTCACAGGGTTAATCACTCAGTTAAGGTGGGGCAGGAACAAATCACAATGGTGGAATGTCATCAGTTAAGGTGGGGCAGGGCCTTTTCACTTCTTTTGTGATTCTTCAGTTACTTCAGGCCATCTGGGCGTATATGTACAAGTCACAGGGGATGTGATGGCTTGGCTTGGGCTCAGAGGCCTGACAATCACACTCTGGGGTCTGGTGGGGGGACAGGGGAGAGATAGCATTAGGAGATATACCTAATGCTAAATGACGAGTTGATGGGTGCAGCACACCAGCATGGCACATGTATACATATGTAACTAACCTGCACATTGTGCACATGTACCCTAAAACTTAAAGTATAAT*AATAATAATAAAAGAAAA*CCCTGCTCTTAGAGGGTTCATCATCTAGAAGTGAGAGGAAGATGTGAAAACAAAATTTTGTCATATGCTGTGGTCAAGTGTTTAAATGGAAGTATTAATAGGAATCTAAAGGAAATACATATGACTAAAATAAAAGAAAGTGAATTCAGCCCTGGGAGTAACAGAGACGTGCAAACAAGATGAGAGGCATTGTCTTGGAGCATAAACAATGTGGTCCTGGTTAGATACTGTACTTAAAATAATATTGTAAATATTGCAGATTTATGGGTTAAAGACTTTGCTGTTGTGGGCTAAGAATCTAACCAGTTCCAGACGACCAACTTTTAAACAGACTTTTGGAGTAGAGTTTCTAAATGTTCTTAAAGGTTCCTGTCAGGCCTTTGAGCCCAAGCCTGTATGTATACATGCAGATGGCCTGAAGCAAGTGAAGAATCACAAAAGAAGTGAAAATGGCCAGTTCCTGCCTTAACTGATGACATTACCTTGTGAAATTCCTTCTCCTGGCTCAGACACTCCCCCACTGAGCACCTCGTGACCCCCGCCCCTGCCTGCCGGAGAACAACCCCTTTGACTGTAATTTTCCACTACCTACCCAACTCCTATAAAATGGCCCTACCCCTATCTCCCTTTGCTGACTCTTTTTGGACTCAGCCCGCCTGCACCCAGGTGATTAAAAAGCTTTATTGCTC*AAAAAAAAAAAAAAAA***AAGAATCAATTCA**AAATGAATTATACAAAAATTAGCTGGGCATGGTGACAGTTT

**Clone 14; PA3; SpIRE(97/622)-14: Filled site spans: chr1:80945988-80951611**

Empty Site:

TTTAAAAAAATTAAAAAATGAAAAAGAAGACAGGACTACACAACTGAAAAATTTTAAAAGAATTGTAAGAGAAGGTTATGAAAACTATATTTCAACAACTTAAGACAACTTTGATGAACT

GTATTTCCTT**AAAGAAAAAATTTATA**AAAACTAC

CTCAAGAACAAGCCTGAATAGATCTCTAATAATGAGATTGAATTATTAATTACAAATCTTCTTACAAAGAAAAGCCCAGAGCTCAATGGCTTGATCCAAAAAAGATATTCTATGTAATCC

Filled Site:

AAAACTATATTTCAACAACTTAAGACAACTTTGATGAACTGTATTTCCTT**AAAGAAAAAATTTATA**GAGTGGGAGGAGCTGAGATGGCCGAATAGGAACAGCTCCAGTCTACAGCTCCCAGCATGAGCAACGCAGAAGATGGGTGATTTCTGCATTTCCATCTGAGCTTTGAAGAGAGCACTGGTTCTCCCATCACGCAGCTGGAGATCTGAGAACGGGCAGACTGCCTCCTCAAGTGGGTCCCTGACCCCTGACCCCCGAGCAGCCTAACTGGGAGGCACCCCCCAGCAGGGGCACACTGACACCTCACACGGCCGGGTACTCCAACAGACCTGCAGCTGAGGGTCCTGTCTGTTAGAAGGAAAACTAACAAACAGAAAGGACATCCACACCAAAAACTCATCTGTACATCACCATCATCAAAGACCAAAAGTAGATAAAACCACAAAGATAGGGAAAAAACAGAGCAGAAAAACTGGAAACTCTAAAAAGCAGAGCACCTCTCCTCCTCCAAAGGAACGCAGTTCCTCACCAGCAACGGAACAAAGCTGGATGGAGAATGACTTTGACGAGCTGAGAGAAGAAGGCTTCAGACGATCAAATTACTCCGAGCTACGGGAGGAAACTCAAACCAAAGGCAAAGAAGTTGAAAACTTTGAAAAAAGTTTAGAAGAATGTATAACTAGAATAACCAATACAGAGAAGTGCTTAAAGGAGCTGATGGAGCTGAAAACCAAGGCTCGAGAACTACGTGAAGAATGCAGAAGCCTCAGGAGCCCATGCGATCAACTGGAAGAAAGGGTATCAGCGATGGAAGATGAAATGAATGAAATGAAGTGAGAAGGGAAGTTTAGAGAAAAAAGAATAAAAAGAAACGAGCAAAGCCTCCAAGAAATATGGGACTATGTGAAAAGACCAAATCTACGTCTGATTGGTGTACCTGAAAGTGACGGGGAGAATGGAACAAGTTGGAAAACACTCTGCAGGATATTATCCAGGAGAACTTCCTCAATCTCACAAGGCAGGCCAACACTCAGATTCAGGAAATACAGAGAACGCCACAAAGATACTCCTCGAGAAGAGCAACTCCAAGACACATAATTCACCAAAGTTGAAATGAAGGAAAAAATGTTAAGGGCAGCCAGAGAGAAAGGTTGGGTTACCCACAAAGGGAAGCCCATCAGACTAACAGCAGATCTCTCGGCAGAAACTCTACAAGCCAGAAGAGAGTGGGGGCCAATATTCAACATTCTTAAAGAAAAGAATTTTCCACCCAGAATTTCATATCCAGCCAAACTAAGCTTCATAAGTGAAGGAGAAATAAAATACTTTACAGACAAGCAAATGCTGAGAGATTTGGTCACCACCAGGCCTGCCCTAAAAGAGCTCCTGAAGGAAGCACTAAACATGGAAAGGAACAACCGGTACCAGCTGCTGCAAAATCATGCCAAAATGTAAAGACCATCGAGACTAGGAAGAAACTGCATCAACTAACGAGCAAAATAACCAGCTAACATCATAATGACAGGATCAAATTCATACATAACAATATTAACTTTAAATGTAAATGGACTAAATGCTCCAATTAAAAGACACGGACTGGCAAATTGGATAAGGAGTCAAGACCCATCAGTGTGCTGTATTCAGGAAACCCATCTCACATGCAGAGACACACATAGGCTCAAAATAAAAGGATGGAGGAAGATCTACCAAGCAAATGGACAACAAAAAAAGGCAGGGGTTGCAATCCTAGTCTCTGATGAAACAGACTTTAAACCAACAAAGATCAAAAGAGACAAAGAAGGCCATTACATCATGGTAAAGGGATCAATTCAACAAGAAGAGCTAACTATCCTAAATATATATGCACCCAATACAGGAGCACACAGATTCATAAAGCAAGTCCTGAGTGACCTACAAAAAGACTTAGACTCCCACACATTAATAATGGGAGACTTTAACACCCCACTGTCAACATTAGACAGATCAATGAGACAGAGAGTCAACAAGGATACCCAGGAATTGAACTCAGCTCTGCACCAAGCAGACCTAATAGACATCTACAGAACTCTCCACCCCAAATCAACAGAATATACATTTTTTTCAGCACCACACCACACCTATTCCAAAATTGACCACATACTTGGAAGTAAAGCTCTCCTCCGCAAATGTAAAAGAACAGAAATTATAAAAAACTATATCTCAGACCACAGTGCAATCAAACTAGGACTCAGGATTAAGAATCTCACTCAAAGCCGCTCAACTACATGGAAACTGAACAACCTGCTCCTGAATGACTACTGGGTACATAACGAAATGAAGGCAGAAATAAAGATGTTCTTTGAAACCAACAAGAACAAAGACACAACATACCAGAATCTCTGGGACACATTCAAAGCAGTGTGTAGAGGGAAATTTACAGCACTAAATGCCCACAAGAGTAAGCAGGAAAGATCCAAAATTGACACCCTAACATCACAATTAAAAGAACTAGAAAAGCAAGAGCAAACACATTCAAAAGCTAGCAGAAGGCAAGAAATAAGTAAAATCAGAGCAGAACTGAAGGAAATAGAGACACAAAAAACCCTTCAAAAAATTAATGAATCCAGGAGCTGGTTTTTTGAAAGGATCAACAAAATTGATAGACCACTAGCAAGACTAATAAAGAAAAAAAGAGAGAAGAATCAAATAGATGCAATGAAAAATGATAAAGGGGATATCACCACTGATCTCACAGAAATACAAACTACCATCAGAGAATACTACAAACACCTCTATGCAAATAAACTAGAAAATCTAGAAGAAATGGATAAATTCCTTGACACATACACTCTCCCAAGACTAAACCAGGAAGAAGTTGACTCTCTGAATAGACCAATAACGGGAGCTGAAAATGTGGCAATAATCAATAGCTTACCAACCAAAAAGAGTCCAGGACCATATGGATTCACAGCTGAATTCTACCAGAGGTACAAGGAGGAACTGGTATCATTCCTTCTGAAACTATTCCAATCAATAGAAAAAGAGGGAATCCTCCCTAACTCATTTTATGAGGCCAGCATCACCCTGATACCAAAGCCGGGCAGAGACACAACCAAAAAAGAGAATTTTAGACCAATATCCTTGATGAACATTGATGCAAAAATCCTCAAGAAAATACTGGCAAACCAAATCCAGCAGCACATCAAAAAGTTTATCCACCATGATCAAGTGGGCTTCATCCCTGGGATGCAAGGCTGGTTCAATATACGCAAATCAATACATGTAATCCAGCATATAAACAGAACCAAAGACAAAAACCACATGATTATCTCAATAGATGCAGAAAAGGCCTTTGACAAAATTCAGCAACCCTTCATGCTAAAAACTCTCAATAAATTAGGTATTGATGGGACGTATCTCAAAATAATAAGAGCTATCTATGACAAACCCACAGCCAATATCATACTGAATGGGCAAGAACTGGAAGCATTCCCTTTGAAAACTGGCACAACACAGGGATGCCCTCTCTCACCACTCCTATTCAACATAGTGTTGGAAGTTCTGGCCAGGGCAATTAGGCAGGAGAAGGAAATAAAGGGTATTCAATTAGGAAAAGAGGAAGTCAAGTTGTCCCTGTTTGCAGACGACATCATTGTATATCTAGAAAACCCCATTGTCTCAGCCCAAAATCTCCTTAAGCTGATAAGCAACTTCAGCAAAGTCTCAGGATACAAAATCAATGTACAAATATCACAAGCATTCTTATACACCAACAACAGACAAACAGAGAGCCAAATCATGAGTGAACTCCCATTCACAATTGCTTCAGAGAATAAAATACCTAGGAATCCAACTTACAAGGGATGTGAAGGACCTCTTCTAGGAGAACTACAAACCACTGCTCAAGGAAATAAAAGAGGATACAAACAAATGGAAGAACATTCCATGCTCATGGGTAGGAAGAATCAATATTGTGAAAATGGCCATACTGTCCAAGGTAATTTACAGATTCAATGCCATCCCCATCAAGCTACCAATGACTTTCTTCACAGAATTGGAAAAAACTACTTTAAAGTTCATATGGAACCAAAAAAGAGCCCACATCGCCAAGTCAATCCTAAGCCAAAAGAACAAAGCTGGAGGCATCACACTACCTGACTTCAAACTATACTACAAGGCCACAGTAACCAAAACAGCATGGTACTGGTACCAAAACAGAGTTATAGATCAATGGAACAGAACAGACCCCTCAGAAATAACACCGCATATCTACAACTATGTGATCTTTGACAAACCTGAGAAAAACAAGCAATGGGGAAAGGATTCCCTATTTAATAAATGGTGCTGGGAAACTGGCTAGCCATATGTAGAAAGCTGAAACTGGATCCCTTCCTCACACCTTACACAAAAATCAATTCAAGCTGGATTAAAGACTTCAACGTTAGACCTAAAACCATAAAAACCCTAGAAGAAAACCTAGGCATTACCATTCAGGACATAGGCATGGGCAAGGACTTCATGTCTAAAACACCAAAAGCAATTGCAACAAAAGACAAAATTGACAAATGGGATCTAATTAAACTAAGGAACTTCTGCACAGCAAAAGAAACTACCATCAGAGTGAACAGGCAACCTACAGAATGGGAGAAAATTTTTGCAACCTACTCATCTGACAAAGGGCTAATATCCAGAATCTACAATGAACTCAAACAAATTTACAAGAAAAAAACAAACAACCCCATCAAAAAGTGGGCAAAGGACAGGAACAGACACTTCTCAAAAGAAGACATTTATGCAGCCAAAAAACACATGAAAAAATGCTCACCATCACTGGCCATCAGAGAAATGCAAATCAAAACCACAATGAGATACCATCTCACACCAGTTAGAATGGCAATCATTAAAATGTCAGGAAACAACAGGTGCTGGAGAGGATGTGGAGAAATAGGAACACTTTTACACTGTTGGTGGGACTGTAAACTAGTTCAACCATTGTGGAAGTCAGTGTGGCGATTCCTCAGGGATCTAGAACTAGAAATACCATTTGACCCAGCCATCCCATTACTGGGTATATACCCAAAGGACTATAAATCATGCTGCTATAAAGACACATGCACACGTATGTTTATTGCGGCACTATTCACAATAGCAAAGACTTGGAACCAATCCAAATGTCCAACAATGATAGACTGGTTTAAGAAAATGTGGCACATATACACCATGGAATACTATGCAGCCATAAAAAAGGATGAGTTCATGTCCTTTGTAGGGACATGGATGAAATTGGAAATCATCATTCTCAGTAAACTATCCCAAGAACAAAAACCAAGCACCGCATATTCTCACTCATAGGTGGGAATTGAACAATGAGAACACATGGACACAGGAAGGGGAACATCACACTCTGGGGACTGTTGTGGGGTGGGGGCAGGGGGGAAGGATAGCATTGGGAGATATACCTAATGCTAGATGACGAGTTAGTGGGTGCAGCGCACCAGCATGGCACATGTATACATATGTAACTAACCTGCACATTGTGCACATGTACCCTAAAACTTAAAGTATAAT*AATAATCATAAAAA****AAAGAAAAAA*TTTATA**AAAACTACCTCAAGAACAAGCCTGAATAGATCTCTAATAATGAG

**Clone 18; PA3; SpIRE(97/622)-18: Filled site spans: chr2:193032462-193038083**

Empty Site:

TCAAGAAAGGTATATTTTCTTTATTACATTAAACTATCCAGGATGTGTAATAAAGAGTGAATTAATTATAGAAAAAATTCATGAGCAATATTTGAAAAATAGACTTTTCATTCAGCCTTT

TTTTAATTGAAAGCAATC**AGAATGATACATGTATT**AAG

TTGTATTTTAGTTGATAAAATAATCTTATGCATCACCTTAATATTGTTTAATTCTTATCTTCTAAAATAACTCCAAATTATTAAAACTTAATGTGACAATAATAATAAC

Filled Site:

ATGAGCAATATTTGAAAAATAGACTTTTCATTCAGCCTTTTTTTAATTGAAAGCAATC**AGAATGATACATGTATT**GGGGAGAAGCCAAGATGGCTGAATAGGAAGAGCTCCGGTCTACAGCTCCCAGCATGAGCAACGCAGAAGACGGGTGATTTCTGCATTTCCATCTGAGCTTTGAAGAGAGCAGTGGTTCTCCCAGCACGCAGCTGGAGATCTGAGAATGGGCAGACTGCCTCCTCAAGTGGGTCCCTGACCCCTGACCCCTGAGCAGCCTAACTGGGAGGCACCCCCCAGCAGGGGCAGACTGACACCTCACACAGCCGGGTACTCCAACAGACCTGCAGCTGAGGGTCCTGTCTGTTAGAAGGAAAACTAACAAACAGAAAGGACATCCTCACCAAAAACCCATCTGTACATCAGCATCATCAAAGACCAAAAGTAGATAAAACCACAAAGATGGGGAAAAAACAGAGCAGAAAAACTGGAAACTCTAAAAAGCAGAGTGCCTCTCCTCCTCCAAAGGAACGCAGTTCCTCACCAGCAACGGAACAAAGCTGGACGGAGAATGACTTTGACGAGCTGAGAGAAGAAGGCTTCAGACGATCAAATTACTCCGAGCTATGGGAGGACATTCAAACCAAAGGCAAAGAAGTTGAAAACTTTGAAAAAAATTTAGAAGAATATATAACTAGAATAACCAATATAGAGAAGTGCTTAAAGGAGCTGATGGAGCTGAAAACCAAGGCTCGAGAACTACGTGAAGAATGCAGAAGCCTCAGGAGCCGATGCGATCAACTGGAAGAAAGGGTATCAGCAATGGAGGATGAAATGAATGAAATGAAGCGAGAAGGGAAGTTTAGAGAAAAAAGAATAAAAAGAAACGAGCAAAGCCTCCAAGAAATATGGGACTATGTGAAAAGACCAAATCTATGTCTGATTGGTGTACCTGAAACTGACAGGGAGAATGGAACCAAGTTGGAAAACACTCTGCAGGATATTATCCAGGAGAACTTCCCCAATCTAGCAAGGCAGGCCAACATTCAGATGCAGGAAATACAGAGAATGCCACAAAGATACTCCTCGAGAAGAGCAACTCCAAGACACACAATTGTCAGATTCACCAAAGTTGAAATGAAGGAAAAAATGTTAAGGGCAGCCAGAGAGAAAGGTCGGGTTACCCTCAAAGGGAAGCCCATCAGACTAACAGCAGATCTCTCGCAGAAACTCTACAAGCCAGAAGAGAGTGGGGGCCAATATTCAACATTCTTAAAGAAAAGAATTTTCAACACAGAATTTCATATCCAGCCAAACTAAGCTTCATAAGTGAAGGAGAAATAAAATACTTTACAGACAAGCAAATGCTGAGAGATTTTGTTACCACCAGGCCTGCCCTAAAAGAGCTCCTGAAGGAAGTGCTAAACATGGAAAGGAACAACCGGTACCAGCCGCTGCAAAATCATGCCAAAATGTAAAGACCATCGAGACTAGGAAGAAACTGCATCAACTAATGAGCAAAATAACCAGCTAACATCATAATGACAGGATCAAATTCACACATAACAATATTAACTTTAAATGTAAATGGATTAAATGCTCCAATTAAAAGACACAGACTGGCAAATTGGACAAAGAGTCAAGACCCATCAGCGTGCTGTATTCAGGAAACCCATCTCACATGCAGAGACACACATAGGCTCAAAATAAAAGGATGGAAGAAGATCTACCAAGCAAATGGAAAACAAAAAAAGGCAGGGGTTGCAATCCTAGTCTCTGATAAAACAGACTTTAAACCAACAAAGATCAAAAGAGACAAAGAAGGCCATTACATAATGGTAAAGGGATCAATTCAACAAGAAGAGCTAACTATCCTAAATATATATGCACCCAATACAGGAGCACCCAGATTCATAAAGCAAGTCCTAAGTGACCTACAAAGAGACTTAGACTCCCGCACATTAATAATGGGAGACTTTAACACCCCACTGTCAACATTAGACAGATCAAAGAGACAGAAAGTTAACAAGGATACCCAGGAATTGAACTCAGCTCTGTGCCAAGCGGACCTAATAGACATCTACAGAACTCTCCACCCCAAATCAACAGAATATACATTTTTTTCAGCACCACACCACACCTATTCCAAAACTGACCACATACTTGGAAGTAAAGCTCTCCTCAGCAAATGTAAAAGAACAGAAATTATAACAAACTATCTCTCAGACCACAGTGCAATCAAACTAGAACTCAGGATTAAGAATCTCACTCAAAACTGCTCAACTACATGGAAACTGAACAACCTGCTCCTGAATGACTATTGGGTACATAACGTAATGAAGGCAGAAATAAAGATGTTCTTTGAAACCAATGAGAACAAAGACACAACATACCAGAATCTCTGGGACTCATTCAAAGCAGTGTGTAGAGGGAAATTTATAGCACTAAATGCCCACAAGAGAAAGCAGGAAAGATCCAAAATTGACAACCTAGCATCACAATTAAAAGAACTAGAAAAGCAAGAGCAAACATATTCAAAAGCTAGCAGAAAGCAAGAAATAACTAAAATCAGAGCAGAACTGAAGGAAATAGAGACACAAAAAACTCTTCAAAAAATTAATGAATCCAGGAGCTGGTTTTTTGAAAGGATCAACAAAATTGATAGACCGCTAGCAAGACTAATAAAGAAAAAGAGAGAAGAACCAAATAGATGCAATAAAAATTGATAAAGGGGATATCACCACCGATCCCACAGAAATACAAACTACCATCAGAGAATACTACAAACACCTCTACACAAATAAACTAGAAAATCTAGAAGCAATGGATAAATTCCTCGACACATCCACTCTCCCAAGACTAAACCAGGAAGAAGTTGAATCTCTGAATAGACCAATAACAGGAGCTCAAATTGTGGCAATAATCAATAGCTTACCAACCAAAAAGAGCCCAGGACCAGATGGATTCACAGCCGAATTCTACCAGAGGTACAAGGAAGAACTGGTACCATTCCTTCTGAAACTATTCCAATCAATAGAAAAAGAGGGAATCCTCCCTAACTCATTTTATGAGGCCAGCATCATCCTGATACCAAAGCCGGGCAGAGACACAACCAAAAAAGAGAATTTTAGACCAATATCCTTGATGAACATTGATGCAAAAATCCTCAATAAAATACTGGCAAACCGAATCCAGCAGCACATCAAAAAGCTTATTCACCATGAGCAAGTGGGCTTCATCCCTGGGATGCAAGGCTGGTTCAATATACGCAAATCAATAAATATAATCCAGCATATAAACAGAACCAAAGACAAAAACCACATAATTATCTCAATAGATGCAGAAAAGGCCTTTGACAAAATTCAACAACGCTTCATGCTAAAAACTCTCAATAAATTAGGTATTGATGGGACGTATTTCAAAATAATAAGAGCTATCTATGACAAACCCACAGCCAATATCATACTGAATGGGCAAAAACTGGAAGCATTCCCTTTGAAAACTGGCACAAGACAGGGATGCCCTCTCTCACCACTCCTATTCAACACAGTGTTGGAAGTTCTGGCCAGGGCAATTAGGCAGGAGAAGGAAATAAAGAGTATTCGATTAGGAAAAGAGGAAGTCAAATTGTCCCTGTTTGCAGACGACATGACTGTATATCTAGAAAACCTCATTGTCTCAGCCCAAAATCTCCTTAAGCTGATAAGCAACTTCAGCAAAGTCTCAGGATACAAAATCAATGTACAAAAATCACAAGCATTCTTATACACCAACAATAGACAAACAGAGCACCAAATCATGAGTGAACTCCCATTCACATTGCTTCAAAGAGAATAAAATACCTAGGAATCCAACTTACAAGGGATGTGAAGGACCTCTTCAAGGAGAACTACAAACCACTGCTCAAGGAAATAAAAGAGGATACAAATGGAAGAATATTCCATGCTCATGGGTAGGAAGAATCAATATCGTGAAAATGCCCATACTGCCCAAGGTAATTTATAGATTCAATGCCATCCCCATCAAGCTACTAATGACTTTCTTCACAGAATTGGAAAAAACTACTTTAAAGTTCATATGGAACCAAAAAAGAGCCCGCATCGCCAAGTCAATCCTAAGCCAAAAGAACAAAGCCGGAGGCATCACACTACCTGACTTCAAACTATACTACAAGGCTACAGTAACCAAAACAGCATGGTACTGGTACCAAAACAGAGATATAGATCAATGGAACAGAACAGAGCCCTCAGAAATAACGCCGCATATCTACAACTATCTGATCTTTGACAAACCTGACAAAAACAAGGAATGGGGAAAGGATTCCCTATTTATTAAATGGTGCTGGGAAAACTGGCTAGCCATATGTAGAAAGCTGAAACTGGATCCCTTCCTTACACCTGATACAAAAATCAATTCAAGATGGATTAAAGACTTAAATGTTAGACCTAAAACCATAAAAACCCTAGAAGAAAACCTAGGCGTTACCATTCAGGACATAGGCATGGGCAAGGACTTCATGTCTAAAACACCAAAAGCAATGGCAACAAAAGCCAAAATTGACAAATAGGATCTCATTAAACTAAAGAGCTTCTGCACAGCAAAAGAAACTACCATCAGAGTGAACAGGGAATCTACAAAATGGGAGAAAATTTTCTCAACCTACTCATCTGACAAAGGGCTAATATCCAGAATCTACAATGAACTCAAACAAATTTACAAGAAAAAAACAAACAACCACATCAAAAAGTGGGCGAAGGACATGAACAGACACTTCTCAAAAGAAGACATTTATGCAGCCAAAAAACACATGAAAAAATGCTCATCATCACTGGCCATCAGAGAAATGCAAATCAAAACCACAATGAGATACCATCTCACACCAGTTAGAATGGCAATCATTAAAAAGTCAGGAAACAACAGGTGCTGGAGAGGATGAGGAGAAATAGGAACACTTTTACACTGTTGGTGGGACTGTAAACTAGTTCAACCCTTGTGGAAGTCAGTGTGGCGATTCCTCAGGGATCTAGAACTAGAAATACCATTTGACCCAGCCATCCCATTATTGGGTATATACCCAAAGGACTATAAATCATGCTGCTATAAAGACACATGCACACGTATGTTTATTGCGGCACTATTCACAATAGCAAAGACTTGGAACCAACCCAAATGTCCAACAATGATAGACTGGATTAAGAAAATGTGGCACATATACACCATGGAATACTATGCAGCCATAAAAAATGATGAGTTCATGTCCTTTATAGGGACATGGATGAAATTGGAAATCATCATTCTCAGTAAACTATCACAAGAACAAAAAAGCAAACACCGCATATTCTCACTCATAGGTGGGAATTGAACAATGAGAACACATGGACACAGGAAGGGGAACATCACACTCTGGGGACTGCTGTGGGGTGGGGGGAGGGGGGAGGGATAACATTGGGAGATATACCTAATGCTAGATGACGAGTTAGTGGTTGCAGCGCACCAGCATGGCACATGTATACATATGTAACTAACCTGCACATTGTGCACATGCACCCTAAAACTTAAAGTATAATACT*AATAAAAAA****AGAA*TGATACATGTATT**AAGTTGTATTTTAGTTGATAAAATAATCTTAT

**Clone 19; PA3; SpIRE(97/622)-19: Filled site spans: chr2:161536144-161541762**

Empty Site:

ATGGTAACCACAAATCAAAAATCTACAGTAGATGCACAAGACAAAAATAGAAGGTTTCAAAGCACACCACTACAGAAAACCATCAAACCATAAAGGAAGGCAGCAAGAGAGGAAGAAAG

AAACAAAGTATCTATAAAAC**AACCAGAAAATG**ATTTAAAAT

GGCAGTAATAAGTGTTTTCTATAAATAATTGCCTTGAATCTAAATGGATTAAATTATTTAATAAAAAGAATGCCTGAATGGAGAGAAAACAAGATCCAACTATATGCAGCCT

Filled Site:

TCAAACCATAAAGGAAGGCAGCAAGAGAGGAAGAAAGAAACAAAGTATCTATAAAAC**AACCAGAAAATG**AGCTCCGGTCTACAGCTCCCAGCGTGAGTGACACAGAAGACGGCTGATTTCTGCATTTCCATCTGAGCTTTGAAGAGAGCAGTGGTTCTCCCAGTACGCAGCTGGAGATCTGAGAACAGGCAGACTGCCTCCTCAAGTGAGTCCCTGACCCCTGACCCCCGAGCAGCCTAACTGGTAGGCACCCCCCAGCAGGGGCAGACTGACACCTCACAGGGCCGGGTACTCCAACAGACCTGCAGCTGAGGGTCCTGTCTGTTAGAAGGAAAACTAACAAACAGAAAGGTCATCCACACCAAAAACCCATCTGTACATCACCATCATCAAAGACCAAAAGTAGATAAAACCACAAAGATGGGGAAAAAACAGAGCAGAAAAACTGGAAACTCTAAAAAGCAGAGCACCTCTCCTCCTCCAAAGGAACGCAGTTCCTCACCAGCAACGGAACAAAGCTGGACGGAGAATGACTTTGACGAGCTGAGAGAAGGCTTCAGATGATCAAATTACTCAAATTACTCTGAGCTACGGGAGGACATTCAAACCAAAGGCAAAGAAGTTGAAAACTTTGAAAAAAATTTAGAAGAATGTATAACTAGAATAACCAATACAGAGAAGTGCTTAAAGTAGCTGATGGAGCTGAAAACCAAGGCTCGAGAACTACGTGAAGAATGCAGAAGCCTCAGGAGCCGATGCGATCAACTGGAAGAAAGGGTATCAGCGATGGAAGATGAAATGAATGAAATGAATCGAGAAGGGAAGTTTAGAGAAAAAAGAATAAAAAGAAACGAGCAAAGCCTCCAAGAAATATGGGACTATGTGAAAAGACCAAATCTTCATCTGATTGGTGTACCTGAAAGTGACGGGGAGAACGGAACCAAGTTGGAAAACATTCTGCAGGATATTATCCAGGAGAACTTCCCCAATCTAGCAAGGCAGGCCAACATTCAGATTCAGGAAATACAGAGAACGCCACAAAGATACTCCTTGAGAAGAGCAACACCAAGACACATAATTGTCAGATTCACCAAAGTTGAAATGAAGGAAAAAATGTTAAGGGCAGCCAGAGAGAAAGGTCGGGTTACCCTCAAAGGGAAGCCCATCAGACTAACAGCAGATCTCTCAGCAGAAACTCTACAAGCCAGAAGAGAGTGGGGGCCAATATTCAACATTCTTAAAGAAAAGAATTTTCAACCCAGAATTTCATATCCAGCCAAACTAAGCTTCATAAGTGAAGGAGAAATAAAATCCTTTACAGACAAGCAAATGCTGAGAGATTTTGTCACCACCAGGCCTGCCCTAAAAGAGCTCCTGAAGGAAGCACTAAACATGGAAAGGAACAACTGGTACCAGCCGCTGCAAAATCATGCCAAAATGTAAAGACCATCGAAACCAGGAAGAAACTGCATCAACTAACGAGCAAAATAACCAGCTAACATCATAATGACAGGATCAAATTCACACATAACAATATTAACTTTAAATGTAAGTGGACTAAATGCTCCAATTAAAAGACACAGACTGGCAAATTGGATAAAGAGTCAAGACCCATCAGTGTGCTGTATTCAGGAAACCCATCTCACGGGCAGAGACACACATAGGCTCAAAATAAAAGGATGGAGGAAGATCTACCAAGCAAATGGAAAACAAAAAAAGGCAGATGTTGCAATCCTAGTCTCTGATAAAACAGACTTTAAACCAACAAAGATCAAAAGAGACAAAGAAGGCCATTACTTAATGGTAAAGGGATCAATTCAACAAGAAGAACTAACTATCCTAAATATATATGCACCCAATACAGGAGCACCCAGATTCATAAAGCAAGTCCTGAGTGACCTACAAAGAGACTTAGACTCCCACACATTAATAATGGGAAACTTTAACACCCCACTGTCAACATTAGACAGATCAATGAGACAGAAAGTCAACAAGGATACCCAGGGATTGAACTCAGCTCTGCACCAAGTGGACCTAATAGACATCTACAGAACTCTGCACCCCAAATCAACAGAATATACATTTTTTTCAGCACCACACCACACCTATTCCAAAATTGGCCACATACTTGGAAGTAAAGCTCTCCTCAGCAAATGTAAAAGAACAGAAATTATAACAAACTATCTCTCAGACCACAGTGCAATCAAACTAGAACTCAGGATTAAGAATCTCACTCAAAACCGCTCAACTACATGGAAACTGAACAACCTGCTCCTGAATGACTACTGGGTACATAACGAAATGAAGGCAGAAATAAAGATGTTCTTTGAAACTAACGAGAACAAAGACACAACATACCAGAATCTCTGGGACGCATTCAAAGCAGTGTGTAGAGGGAAATTTATAGCACGAAATGCCCACAAGAGAAAGCAGGAAAGATCCAAAATTGACACCCTAACATCACAATTGAAAGAACTAGAAAAGCAAGAGCAAACACATTCAAAAGCTAGCAGAAGGCAAGAAATAACTAAAATCAGAGCAGAACTGAAGGAAATAGGGACACAAAAAAACCTTCAAAAAATTAATGAATCCAGGAGCTGGTTTTTTGAAAGGATCAACAAAATTGATAGACCGCTAGCAAGACTAATAAAGAAGAAAAGACAGAAGAATCAAATAGGCGCAATAAAAAATGATAAAGGGGATATCACCACCGATCCCACAGAAATACAAACTACCATCAGAGAATACTACAAACACCTCTATGCAAATAAACTAGAAAATCTAGAAGAAATGGATAAATTCCTCGACACATACACTCTCCCAAGACTAAACTAGGAAGAAGCTGAATCTCTGAATAGACCAATAACAGGATCTGAAATTGTGGCAATAATCAATAGCTTACCAACGAAAAAGAGTCCAGGACCAGATGGATTCACAGCCGAATTCTACCAGAGGTACAAGGAGGAACTGGTACCATTCCTTCTGAAACTATTCAAATCAATAGAAAAAGATGGTATCCTCCCTAACTCATTTTATGAGGCCAGCATCATCCTGATACCAAAGCCGGGCAGAGACACAACCAAAAAAGAGAATTTTAGACCAATATCCTTGATGAACATTGATGCAAAAATCCTCAATAAAATACTGGCAAACCGAATCCAGCAGCACATCAAAAAGCTTATCCACCATGATCAAGTGGGCTTCATCCCTGGGATGCAAGGCTGGTTCAATATACGCAAATCAATAAATGTAATCCAGCATATAAACAGAACCAAACACAAAAACCACATGATTATCTCAATAGATGCAGAAAAGGCCTTTGACAAAATTCAACAACCCTTCATGCTAAAAACTCTCAATAAATTAGGTATTGATGGGACGTATCTCAAAATAATAAGAGCTATCTATGACAAACCCACAGCCAATATCATACTGAATGGGCAAAAACTGGAAGCATTCCCTTTGAAAACTGGCACAAGACTAGGATGCCCTCTCTCACCACTCCTATTCAACATAGTGTTGGAAGTTCTGGCCAGGGCAATTAGGCAGGAAAAGGAAATAAAAGGTATTCAATTAGGAAAACAGGAAGTCAAATTGTTCCTGTTTGCAGATTACATGATTGTATATCTAGAAAACCCCATTGTCTCAGCCCAAAATCTCCTTAAGCTGATAAGCAACTTCAGCAAATCTTAAGATACAAAATCAATGTACAAAAATCACAAGCATTCTTATACACCAATAACAGACAAACAGAGAGCCAAATCATGAGTGAACTCCCATTCACAATTGCTTCAAAGAGAATAAAATACCTAGGAATCCAACTTATAAGGGACGTGAAGGACCTCTTCAAGGAGAACTACAAACCACTGCTGAAGGAAATAAGAGAGGATACAAACAAATGGAAGAACATTCCATGCTCATGGGTAGGAAGAATCAATATTGTGAAAATGGCCATAGGGTCCAAGGTAATTTACAGATACAATGCCATCCCCATCAAGCTACCAATGACTTTCTTCACAGAATTGGAAAAAACTACTTTAAAGTTCATATGGAACCAAAAAAGAGCCTGCATCGCCAAGTCAATCCTAAGCCAAAAGAACAAAGCTGGAGGCATCACACTACCTGACTTCAAACTATACTACAAGGCTACAGTAACCAAAACAGCATGGTACTGGTACCAAAACAGAGATATAGATCAATGGAACAGAACAGAGCCCTCAGAAATAACGCCACATATCTACAACTATCTGATCTTTGACAAACCTGAGAAAAACAAGTAATGGGGAAAGGATTCCCTATTTAATAAATGGTGCTGGGAAAACTGGCTAGCCATATGTAGAAAGCTGAAACTGGATCCCTTCCTTACACCTTATACAAAAATTAATTCAAGATGGATTAAAGACTTAAACGTTAGACCTAAAACCATAAAAACCCTAGAAGAAAACCTAGGCATTACCATTCAGGACATAGGCATGGGCAAGGACTTCATGTCTAAAACACCAAAAGCAATGGCAACAAAAGCCAAAATTGACAAATGGGATCTAATTAAACTAAAGAGCTTCTGCACAGCAAAAGAAACTACCATCAGAGTGAACAGGCAACCTACAACATGGGAGAAAATTTTCGCAACCTACTCATCTGACAAAGGGCTAATATCCAGAATCTACAATGAACTCAAACAAATTTACAAGAAAAAAACAAACAACCCCATCAAAAAGTGGGCAAAGGACATGAACAGACATTTCTCAAAAGAAGACATTTATGCAGCCAAAAAACACATGAGAAAATGCTCATCATCACTGGCCATCAGAGAAATGCAAATCAAAACCACAATGAGATACTATTTCACACCAGTTAGAATGGCAATCATTAAAAAGTCAGGAAACAACAGGTGCTGGAGAGGATGTGGAGTAATAGGAACACTTTTACACTGTTGGTGGGACTGTAAACTAGTTCAACCATTGTGGAAGTCAATGTGGCAATTCCTCAGGGATCTAGAACTAGAAATACCATTTGACCCAGCCATCCCATTACTGGGTATATACCCAAAGGACTATAAATCATGCTGCTATAAAGACACATGCACACGTATCTTTATTGCGGCATTATTCACAATAGCAAAGACTTGGAACCACCCCAAATGTCCAACAATGATAGACTGGATTAAGAAAATGTGGCACATATACACCATGGAATACTATGCAGCCATAAAAAATGATGAGTTCATGTCCTTTGTAGGGACATGGATGATATTGGAAATCATCATTCTCAGTAAACTATCGCAAGAACAAAAAACCAAACACCGCATATTCTCACTCATAGGTGGGAACTGAACAATGAGAACACATGGACACAGGAAGGGTAACATCGCACTCTGGGGACTTTTGTGGGGTGGGGGGATTGGGGAGGGATAGCATTCGGAGATATACCTAATGCTAGATGACGAGTTAGTGGGTGCAGTGCACCAGCATGGCACATGTATACGTATGTAACTAACCTGCACATTGTGCACATGTACCCTAAAACTTAAAGTATAAT*AATAATAAAAAAA****AA*CCAGAAAATG**ATTTAAAATGGCAGTAATAAGTGTTTTCTATAAATAATTGCCTTGAATCTAAATGG

**Clone 20; PA3; SpIRE(97/622)-20: Filled site spans: chr2:131373741-131379521**

Empty Site:

AATTTAGGTTATGCACATAGTTTAAGTCATATGTGTTGGAAATTGAAACTAGTAAGGCAAGGCCCACTGCGGCTTTGCCGGCAGCAAATACTAGTAGGGTGATGGGTACTACGGTTGCTA

GGGTAAAATGTATATT**TAAAGTTATAAGG**GTATTTATGATGAATAATGATAATATTATTCCTTCTAGGCATAGTAGGAAAAAAGTTGTATTTATATATAGCAATGCTGGCAGCTAGGACTGTGAGAGAAAGGAGTAGAAGAACTGCTGATA

Filled Site:

GCAGCAAATACTAGTAGGGTGATGGGTACTACGGTTGCTAGGGTAAAATGTATATT**TAAAGTTATAAGG**AGGGGTGGAGCCAAGATGGCCAAATAGGAACAGCTCCGGTCTACAGCTCCCAGCATGAGCGATGCAGAAGACGGGTGATTTCTGCATTTTCATCTGAGCTTTGAAGAGAGCAGTGGTTCTCCCAGCATGCAGCTGGAGATCTGAGAACGGGCAGACTGCCTCCTCAAGTGGGTCCCTGACCCCTGACCCCCGAGCAGCCTAACTGGGAGGCACCCCCAAGTAGGGGCAGACTGACACCTCACACGGCCAGGTACTTCTCTGAGACAAAACTTCCAGAGGAACGATCAGACAGCAGCATTTGCAGTTCATGAAAATCTGCTGTTCTGCAGCCACTGCTGCGGGTACCCAGGCAAACAGGGTCTGGAGTGGACCTCTAGCAAACTCCAACAGACCTGCAGCTGAGGGTCCTATCTGTTAGAAGGAAAACTAACAAACAGAAAGGACATCCACACCAAAAACCCATCTGTACATCACCATCATCAAAGACCAAAAGTAGATAAAACCACAAAGATGGGGAAAAAACAGAGCAGAAAAACTGCAAACTCTAAAAAGCAGAGCGCCTCTCCTCCTCCAAAGGAATGCAGTTCCTCACCAGCAACGGAACAAAGCTGGACGGAGAAGGACTTTGACGAGTTAAGAGAAGAAGGCTTCAGATGATCAAACTACTCTGAGCTACAGGAGGAAATTCAAACCAAAGGCAAAGAAGTTAAAAACTTTGAAAAAAATTTAGACAAATGTATAACTAGAATAACCAATGCACAGAAGTCCTTAAAGGAGCTGATGGAGCTGAAAGCCAAGGATCGAGAAATACGTGAAGAATGCAGAAGCCTCAGGAGCCAATGCAATCAACTGGAATAAAGGGTATCAGTGATGGAAGATGAAATGAATGAAATGAAGTGAAAAGGGAAGTTTAGAGAAAAAGAATAAAAAGAAATGAACAAAGCCTCCAAGAAATATGGGACTATGTGAAAAGACCAAATCTATGTCTGATTGGTGTACCTGAAAGTGACGGGGAGAACAGAACCAAGTTGGAAAACACTCTACAAGATATTATCCAGGAGAACTTCCCCAATCTAGCAAGGCAGGCCAACATTCAAATTCAGGAAATACAGAGAATGCCACAAAGATACTCCTCGAGAAGAGCAACTCCAAGACACATAATTGTCAGATTCACCAAAGTTGAAATGAAGGAAAAAACGTTAAGGGCAGCCAGAGAGAAAGGTCGGGTTACCCACAAAGGGAAGCCCATCAGACTAACAGCAGATCTCTTGGCAGAAACTCTACAAGCCAGAAGAGAGTGGGGGCCAATATTCAACATCCTTAAAGAAAAGAATTTTCAACCCAGAATTTCCTATCCAGCCAAACTAAGCTTCATAAGTGAAGGAGAAATAAAATACTTTACAGACAAGCAAATGCTGAGAGATTTTGTCACCGCCAGGCTTGCCCTAAAAGAGCTCCTGAAGGAAGCACTAAACATGGAAAGGAACAACCGGTACCAGCCACTGCAAAATCATGCCAAATTGTAAAGACCATCCAGGCTAGGAAGAAACTGCATCAACTAACGAGCAAAATAACCAGCTAACATCATAATGACAGGATCAAATTCACACATAACAATATTAACTTTAAATGTAAATGGTCTACATGCTCCAATTAAAAGACACAGACTGGCAAATTGGATAAAGACTCAAGACCCATCAGTGTGCTGTATTCAGGAAACCCATCTCACGTGCAGAGACACACATAGGCTCAAAATAAAGGGATGGAGGAAGATCTACCAAGCAAATGGAAAACAAAAAAAGGCAGGGGTTGCAATCCTACTCTTTGATAAAACAGAGTTTAAACCAGCAAAGATCAAAAGAGACAAAGAAGGCCATTACATAATGGTAAAGGGATCAATTCAACAAGAAGAGCTAACTATCGTAAATATATATGCACCCAATACAGGAGCACCCAGATTCATAAAGCAAGTCCTGAGTGACCTACAAAGAGACTTAGACTCCCACACAATAATAATGGGAGACTTTAACACCCCACTGTCAACATTAGACAGATCAACGAGACAGAAAGTTAACAAGGATACCCAGGAATTGAACTCAGCTCTGCACCAAGCGGACCTAGTAGACATCTATAGAACTCTCCACCCCAAATCAACAGAATCTACATTTTTTCAGCACCACACCACACCTATTCCAAAATTGATCACATAGTTGGAAGTAAAGCCCTCCTCAGCAAATGTAAAAGAACAGAAATTATAACAAACTATCTCTCAGACCACAGTGCAATCAAACTAGAACTCAGGATTAAGAAACTCACTCAAAACCACTCAACTATATGGAAACTGAACAACCTGCTCCTGAATGACTACTGGGTACATAACAAAATGAAGGCAGAAATAAAGATGTTCTTTGAAACCAATGAGAACAAAGACACAACATACCAGAATCTCTGGGACACATTCAAAGCAGTGTGTAGAGGGAAATTTATGGCACTAAATGCCCACAAGAGAAAGCAGGAAAGATCCAAAATTGACACCCTAACATCACAATTAAAAGAACTAGAAAAGCAAGAGCAAACACATTCAAAAGCTAGCAGAAGGCAAGAAATAACTAAAATCAGAGCAGAACTGAAGGAAATAGAGACACAAAAAACCCTTCAAAAAATTAATGAATCCAGGATCTGGTTTTTTGAAAGGATCAACAAAATTGATAGACCGCTAGCAAGACTAATAAAGAAGAAAAGTGAGAAGAATCAAATAGACGCAATAAAAAATGATAAAGGGGATATCACCACCAATCCCACAGAAATACAAACTACCATCAGAGAATATTACAAACACCTCTACGCAAATAAACTAGAAAATCTAGAAGAAATGGATAAATTCCTCGACACATACATCCTCCCAAGACTAAACCAGGAAGAAGTTGAATCTCTGAATAGATCAATAATAGGCTCTGAAATTGTGGCAATAATCAATAGCTTACCAACCAAAAAAAGTCCAGGACCAGATGGATTCACAGCCGAATTCTACCAGAGGTACAACGAGGAGCTGGTACCATTCCTTCTGAAACTATTCCAATCAATAGAAAAAGGAATCCTCCCTAACTCATTTTATGAGGCCAGCATCATCCTGATACCAAAGCTGGGCAGAGACATAACCAAAAAAGGGAATTTTAGACCAATATCCTTGATGAACATTGATGCAAAAATCCTCAATAAAATACTGGCAAACTGAATCCAGCAGCACATCAAAAAGCTTATCCACCATGATCAAGTGGGCTTCATCCCTGGGATGCAAGGCTGGTTCAACATATGCAAATCAATAAATGTAATCCAGCATATAAACAGAACCAAAGACAAAAACCACATGATTATCTCAATAGATGCAGAAAAGGCCTTTGACAAAATTCAACAACCCTTCATGCTAAAAACTCTCAATAAATTAGGTATTGATGGGACGTATCTCAAAATACTAAGAGCTACCTATGACAAACCCACAGCCAATATCACACTGAATGGGCAAAAACTGGTAGCATTCTCTTTGAAAACTGGCATGAGACAGGGATGCCCTCTCTCACCACTCCTATTCAACATAGTGTTGGAAGTTCTGGCCAGGGCAATCAGGCAGGAGAAGGAAATAAAGGGTATTCAATTAGGAAAAGAGGAAGTCAAATTGTCCCTGTTTGCAGATGACATGATTGTATATCTAGAAAACCCCATTGTCTCAGCCCAAAACCTCCTTAAGCTGATAAGCAACTTCAGCAAAGTCTCAGGATACAAAATCAATGTACAAAAATCACAAGCATTCTTATACACCAATAACAGACAAACAGAGAGCCAAATCATGAGTGAACTCCCATTCACAATTGCTTCAAAGAGAATAAAATACCTAGGAATCCAACTTACAAGGGCTGTGAAGGACCTCTTCAAGGAGAACTACAAACCACTGCTCAGTGAAATAAAAGAGGATACAAAGAAATGGAAGAACATTCCATGCTCATGGGTAGGAAGAATCAATATCGTGAAAATGGCCATACTGCCCAAGGTCATTTATAGATTCAATGCCATCCCCATCAAGCTACCAATGACTTTATTCACAGAATTGGAAAAAACTACTTTAAAGTTCATATGAAACCAAAAAAGAGCCCGCATCGCCAAGTCAATCCTGAACCAAAAGAACAAAGCCAGAGGCATCATGCTACGTGACTTCAAACTATACTACAAGGCTACAGTAACCAAAACAGCATGGTACTGGTACCAAAACAGAGATATAGATAAATGGAACAGAACAGAGCCCTCAGAAATAACACCACACATCTACAACCATCTGATCTTTGACAAACCTGGGAAAAACAAGCAATGGGGAAAGGATTCCCTATTTAATAAATGGTGCTGGGAAAACTGGCTAGCCATATGTAGAAAGCTGAAACTGGATCTCTTCCTTACACCTTATACAAAAATTAATTCAAGATGGATTAAAGACTTAAACGTTAGACCTAAAACCATAAAAACCCTGAAACCCAAGAAAACATAGGCATTACCATTCAGGACATAGGCATGGGCAAGGACTTCATGTCTAAAACACCAAAAGCAATGGCAAGAAAAGCCAAAATTGACAAATGGGATGTAATTAAACTAAAGAGCTTCTGCACAGCAAAAGAAACTACCATCAGAGTGAACAGGCAACCTACAAAATGGGAGAAAATTTTCGCAACCCACTCATCTGACAAAGGGCTGATATCCAGAATCTACAATGAACTCAAACAAATTTACAAGAAAAAAACAAACAAGCCCATGAAAAAGTGGGCGAAGGACATGAACAGACACTTCTCAAAAGAAGACATCTATGCAGCCAAAAAACACATGAAAAAATGCTCACCATCACTGGCCATCAGAGAAATGCAAATCAAAACCACAATGAGATACCATCTCACACCAATTAGAATGGCGATCATTAAAAAGTCAGGAAACAACAGGTGCTGGAGAGGATGTGGAGAAATAGGAACACTTTTACACTGTTGGTGGGACTGTAAACCCATTCAACCATTGTGGAAGTCAGTGTGGTGATTCCTCAGGGATCTAGAACTAGAAATACCATTTGACCCAGCCATCCCATTACTGGGTATATACCCAAAGGACAATAAATCATGCTGCTATAAAGACACATGCACACGTATGTTTATTGAGGCACCATTCACAATAGCAAAGACTTGGAGCCAACCCAAATGTCCAACAATGATAGACTGGATTAAGAAAATGTGGCACATATACACCATGGAATACTATGCATCCATAAAAAATGATGAGTTCATGTCCTTTGTAGGGACATGGATGAAATTGGAAATCATCATTCTCAGAAACTATCACAAGGACAAAAAACCAAACACCGCATGTTCTCTCTCATAGGTGGGAATTGAACGATGAGAACACATGGACACAAGAAGGGGAACATCACACTCTGGGGTCTGTTGTGGGGTGGGGGGAGGGGGGAGGGATAGCATTAGGAGATATACCTAATGCTAAATGACGAGTTAATGGGTGCAGCACACCAGCATGGCACATGTATACATATGTAACTAACCTGCACATTGTGCACATGTACCCTAAAACTTAAAGTATAAT*AATAATAAAATAAATAAATAAATAAGTAAA****TAAA*GTTATAAGG**GTATTTATGATGAATAATGATAATATTATTCCTTCTAGGCATAGTAG

**Clone 23; PA3; SpIRE(97/622)-23: Filled site spans: chr2:76904808-76910549**

Empty Site:

GTCAAGTTTTGTATGCCAGAGCAACATCAAATGTAACATATGTAAAAAATGAAAGGGGAGGTATAATATATAAGAATACCTACCACAAGGTGTTATGGTAAAAATAACATGAGCTATTCA

CTTGTCAAAAGTGTTTT**GAAAAGGCATCATTCTT**ATAG

CACCATTATTACGAAGAAATATTTGTTTCTCATTATGTCAGGGATTGATGGAATCTTTCCCATAAAGAATGTATGTATCCAAAAATAGAGCTAGTATTAAATTTATATCA

Filled Site:

TACCACAAGGTGTTATGGTAAAAATAACATGAGCTATTCACTTGTCAAAAGTGTTTT**GAAAAGGCATCATTCTT**GGGGGAGGAGCCAAGATGGCCAAATAGGAACAGCTTCGGTCTACAGCTCCCAGTGTGAGCGACGCAGAAGATGGGTGATTTCTGCATTTCCATCTGAGCATTGAAGAAAGCAGTGGTTCTCCCAGAACGCAGCTGGAGATCTGAGAACAGGCAGACTGCCTCCTCAAGTGGGTCTCTGACCCCCTGACCACTGAGCAGCCTAACTAGGAGGCACCCCCCAGTAGGGGCAGACTGACACCTCACATGGCCGGGTACTCCTCTGAGACAAAACTTCCAGAGGAACGATCAGACAGCAGCATTTGCAGTTCACGAAAAACCACTGTTCTGCAGACACCGCTGCTGATACCCAGGCAAACAAGGTCTGGAGTGGACCTCTAGCAAATTCCAACAGACCTGCGGCTGAGGGTCCTGTCTGTTAGAAGGAAAACTAACAAACAGAAAGGACATCCACACCAAAATCCCATCTGTACATCACCATCATCAAAGACCAAAAGTAGATAAAACCACAAAGATGGGGAAAAAACAGAGCAGAAATACTGGAAACTCTAAAAACCAGAGCACCTCTCCTCCTCCAAAGGATCGCAGTTCCTCACCAGCAATGGAAAAAAGCTGGACGGAGAATGACTTTGACGAGTTGAGAGAAGAAGGCTTCAGACGATCAAACTACGAGCTACAGGAGGAAATTCAAACCAAAGGCAAAGAAGTTAAAAACTTTGAAAAAAATTTAGACGAATGTATAACTAGAATAACCAATACAGAGAAGTGCTTAAAGGAGCTGACTGAGCTGAAACCAAGGCTCCAGAACTACGTGAAGAATGCAGAAGCCTCAGGAGCCGATGCGATCAACTGGAAGAAAGGGTATCAGTGATGGAAGATGAAATGAATGAAATGAAGCGAGAAGGCAAGTTTAGAGAAAAAAGAAAAAAAAGAAACGAACAAAGCCTCCAAGAAATATGGGACTTTGTGAAAAGACCAAATCTACATCTGATTGGTGTACCTGAAAGTGACAGGGAGAATGGAACCAAGTTGGAAAACACTCTGCAGGATATTATCCAAGAGAACTTCCCCAATCTAGCAAGGCAGGCCAACATTCAGATTCAGGAAATACAGAGAACGCCACAAAGATACTCCTCGAGAAGAGCAACTCCAAGGCACATAATTGTCAGATTCACTAAAGTTGAAATGAAGGAAAAAATGTTAAGGGCAGCCAGAGAGAAAGGTCGGGTTACCCACAAAGGGAAGCCCATCAGACTAACAGCTGATCTCTCAGCAGAAACTCTACAAGTCAGAAGAGACTGGGGGCCAATATTCAACATTCTTAAAGAAAAGAATTTTCAACCCAGAATTTCATATCCAGCCAAACTAAGCTTCATAAGTGAAGGAGAAATAAAATACTTTACAGACAAGCAAATGCTCAGAGATTTTGTCACCACCAGGCCTGCCCTAAAAGAGCTCCTGAAGGAAGCGCTAAACGTGGAAAGGAACAACCAGTACCAGATGCTGCAAAGTCATGCCAAATTGTAAAGACCATCGAGACTAGGAAGAAACTGCATCAACTAACGAGCAAAATAACCAGCTAACATCATCAAGACAGGATCAAATTCACACATAAGAATATTAACTTTAAATGTAAATGGACTAAATGCTCCAATCAAAAGACACAGACTGGCAAATTGGATAAAGAGTCAAGACCCATCAGTCTGTTGTATTCAGGAAAAGCATCTCACATGCAGAGACACACATAGGCTCAAAATAAAAGGATGGAGGAAGATCTACCAAGCCAATGGAAAACAAAAAAAGGCAGGGGTTGCAATCCTAGTCTCTGATAAAACAGACTTTAAACCAACAAAGATCAAAAGAGACAAAGAAGGCCATTACATAATGGTAAAGGGATCAATTCAACAAGAAGAGCTAACTATCCTAAATATATATGCACCCAATACAGGAGCACCCAGATTCATAAAGCAAGTCCTGAGTGACCTACAAAGAGACTTAGACTCCCACACAATAATAATGGGAGACTTTAACACCCCACTGTCAACATTAGACAGATCAATGAGACAGAAAGTTAACAAGGATACCCAGGAATTGAACTCAGCTCTGCACCAAGCAGAATAGACATCTACAGAACTCTCCACCCCAAATCAACACAATATACATTTTTTTCAGCACCAAACCACACCTATTCCAAAATTAACCACATAGTTGGAAGTAAAGCTCTCCTCAGCAAATGTAAAAGAACAGAAATTATAACAAACTATCTCTCAGACCACAGTGCAATCAAACTAGAACTCAGGATTAAGAAACTCACTCAAAACTGCTCAACTACATGGAAACTGTACAACCTGCTCCTGAATGACTACCAGGTACATAATGAAATGAAGGCAGAAATAAAGATGTTCTTTGAAACCACGAGAACAAAGACACAACATACCAGAATCTCTGGGACGCATTCAAAGTAGTGTGTAAAGGGAAATTTATAGCACTAAATGCCCACAAGAAAAAGCAGGAAAGATCCAAAATTGACACCCTAACATCACAATTAAAAGAACTAGAAAAGCAAGAGCAAACACATTCAAAAGCTAGCAGAAGGCAAGAAATAACTAAAATCAGAGCAGAACTGAAGGAAATAGAGACACAAAAAACCCTTCAAAAAGTTAATGAATCCAGGAGCTGGTTTTTTGAAAGGATCAACAAAATTGATAGACCACTAGCAAGACTAATAAAGAAAAAAAGAGAGAAGAATCAAATAGACGCAATAAAAAATGATAAAGGGGATATCACCATCGATACCACAGAAATACAAACTACCATCAGAGAATACTACAAACACCTCTACGCAAATAAACTAGAAAATCTAGAAGAAATGGATAAATTCCTCGACACATACACTCTCCCAAGACTAAACCAGGAAGAAGTTGAATCTCTGAATAGACCAATAACAGGAGCTGAAATTGTGGCAATAATCAATAGCTTACCAACCGAAAAGAGTCCAGGACCAGATGGATTTACAGCTGAATTCTACCAGAGGTACAAGGAGGAACTGGTACCATTCCTTCTGAAACTATTCCAATCAATAGAAAAAGAGGGAATCCTCCCTAACTCATTTTATGAGGCCAGCATCATCCTGATACCAAAGCTGGGCAGAGACACAGCCAAAAAACAGAATTTTAGACCAATATCCTTGATGAACATTGATGCAAAAATCCTCAATAAAATACTGGCAAACCGAATCCAGCAGCACATCAAAAAGCTTATCCACCATGATCAAGTGGGCTTCATCCCTGGGATGCAAGGCTGGTTCAATATACGCAAATCAATAAATGTAATCCAGCATATAAACAGAACCAAAGACAAAAACCACATGATTATCTCAATAGATGCAGAAAAGGCCTTTGACAAAATTTAACAACCTTCATGCTAAAAACTCTCAATAAATTAGGTATTGATGGGCCATATCTCAAAATCATAAGAGCTATCTATGACAAACCCACAGCCAATATCATACTGAATGGGCAAAAACTGGAAGCATTCCCTTTGAAAACTGGCACAAGACAGGGATGCCCTCTCTCACCACTCCTATTCAACATAGTGTTGGAAGTTCTGGCCAGGGCAATTAGGCAGGAGAAGGAAATAAAGGGTATTCAATTAAGAAAAGAGGAAGTCAAAGTGTCCCTGTTTGCAGATGACATGATTGTATATCTAGAAAACCCCATTGTCTCAGCCCAAAATCTCCTTAAGCTGATAAGCAACTTCAGCAAAGTTTCAGGACACAAAATCTCTGTACAAAAATCACAAGCATTCTTATACACCAATAAGAGACAAACAGAGAGCCAAATCATGAGTGAACTCCCATTCACAATTGCTTCAAAGAGAATAAAATACCTAGGAATCCACCTTACAAGGGATGTGAAGGACCTCTTCCAGGAGAACTACAAACCACTGCTCAAGGAAATAAAAGAGGATACAAACAAATGGAAGAACATTCCATGCTCATGGGTAGGAAGAGTCAATATTGTGAAAATGGCCATACTGCCCAAGGTAATTGATAGATTCAATGCCATCCCCATCAAGCTACCAATGCCTTTCTTCACAGAATTGGAAAAAACTACTTTAAAGTTCACATGGAAGCAAAAAAGAGCCCTCATCGCCAAGTCAATCCTAAGCCAAAAGAACAAAGCTGGAGGCATCACACTACCTGACTTCAAACTATACTACAAGGCTACAGTAACCAAAACAGCATGGCACTGGCACCAAAACAGAGATATAGATCAATGGAACAGAACAGAGCCCTCAGAAATAATGCTGCTTATCTACAACTATCTGATCTTTGACAAACCTGAGAAAAACAAGCAATGGGGAAAGGATTCCCTGTCTAATAAATGGTGCTGGGAAAACTGGCTAGCCATATGTAGAAAGCTGAAACTGGATCCCTTCCTTACACCTTATACAAAAATCAATTCAAGATGGATTAAAGACTTAAACCTTAGACCTAAATCCATAAAAACCCTAGAAGAAAACCTAGGCATTACCATTCAGGACATAGGCATGGGCAAGGACTTCATGTCTAAAACACCAAAAGCAATGGCAACAAAAGACAAAATTGACAAATGGGATCTCATTAAACTAAAGAGCTTCTGCACAGCAAAAGAAACTACCATCAGAGTGAACAGGCAACCTACAAAATGGGAGAAAATTTTCGCAACCTACTCATTTGACAAAGGGCTAATATCCAGAATCTACAATGAACTCAAACAAATTTACAAGAAAAAAACAAACAACCCCATCAAAAAGTGGGCGAAGGACATGAACAGACACTTCTCAAAAGAAGACATTTATGCAGCCAAAAAAACACATGAAAAAATGCTCACCATCACTGACTATCAGAGAAATGCAAATCAAAACCACAGTGAGATATCATCTCACACCAGTTAGAATGGCAATCATTAAAAAGGCAGGAAACAACAGGTGCTGGAGAGGATGTGAAGAAATAGGAACACTTTCACATTGTTGGTGGGACTGTCAACTAGTTCAACCATTGTGGAAGTCAGTGTGGTGATTCCTCAGGGATCTAGAACTAGAAATACCATTTGACCCAGCCATCCCATTACTGCGTATATACCCAAAGGATTGTAAATCATGCTGCTATAAAGACACATGAACACGTATGTTTATTGTGGCACTATTCACAATAGCAAAGACTTGGAACCAACCCAAATGTCCAACAATGATAGACTGGATTAAGAAAATGTGGCACATAGACACCATGGAATACTATGCAGCCATAAAAAATGATAAGTTTATGTCCTTTGTAGGGACATGGATGAAATTGGAAATCATCATTCTCAGCAAACTATCGGAAGAACAAAAAACCAAACACTGCATATTCTCACTCATAGGTGGGAATTGAACAATGAGAACACATGGACACAGGAAGGGGAACATCACACTCTGGGGACTGTTGTGGGGTGGGGGGAGGGTGGAGGGATAACATTGGGAGATATACCTAATGCTAGATGACGAGTTAGTGGGTGCAGAGCACCAGCATGGCACATGTATACATATGAAACTAACCTGCACATTGTGCACATGTACCCTAAAACTTAAAGTATAAT*AATAAAAAAAA****GAAAA*GGCATCATTCTT**ATAGCACCATTATTACGAAGAAATA

**Clone 25; PA3; SpIRE(97/622)-25: Filled site spans: chr2:96441023-96446702**

Empty Site:

GCCATCCCATTACTGAGTATATACACAAAGGATTATAAATCATGCTGCTATAAAGACACATGCACACGTATGTTTATTGCGGCACTATTCACAATAGCAAAGACTTGGAACCAACCCAAATG

TCCATCAATGATAGACTGGATT**AAGAAAATGTGACAC**ATA

TACACTGTGGAATACTATGCAGCCATAGAAAAGGATGAGTTCATGTCCTTTGTAGGGACATGGATGAAGCTGGAAACCATCATTCTGAGCAAACTATTGCACTGACAGAAAACCAAACAC

Filled Site:

CACTATTCACAATAGCAAAGACTTGGAACCAACCCAAATGTCCATCAATGATAGACTGGATT**AAGAAAATGTGACAC**GGTGGGGAGGAGCCAAGATGGCCAAATAGGAACAGCTCCGGTCTACAGCTCCCAGCGTGAGCGACGCAGAAAAGACAGCCGATTTCTGCATTTCCATCTGAGCTTTGAAGAGAGCAGTGGTTCTCCCAGCACGCAGCTGGAGATCTGAGAACGGGCAGACTGCCTCCTTAAGTGGGTCCCTGACCCCTGACCCCTGAGCAGCCTAACTGGGAGGCACCTCCCAGCAGGGGCAGACTGACACCTCACACGGCCGGGTACTCCAACAGACCTGCAGCTGAGGGTCCTGTCTGTTAGAAGGAAAACTAACAAACAGAAAGGACATCCACACCAAAAAGCCATCTGTACATCACCATCATCAAAGACCAAAAGTAGATAAAAACACAAAGATGGGGAAAAAACAGAGCAGAAAAACTGGAAACTCTAAAAAGCAGAGTGCCTCTCCTCCTCCAAAGGAATGCAGTTCCTCACCAGCAACGGAACAAAGCTGGACGGAGAATGACTTTGACGAGTTGAGAGAAGAAGGCTTCAGATGATCAAATTACTCCGAGCTACGGGAGGACATTCAAACCAAAGGCAAAGAAGTTGAAAACTTTGAAAAAAATTTAGAAGAATGTATAACTAGAATAACCCATACAGAGAAGTGCTTAAAGGAGCTGATGGAGCTGAAAACCAAGGCTCGAGAACTACGTGAAGAATGCAGAAGCCTCAGGAGCCGATGAGATCAACTGGAAGAAAGGGTATCAGGGATGGAAGATGAAATGAATGAAAGGAAGCAAGAAGGGAAGTTTAGAGAAAAAAGAATAAAAAGAAATGAGCAAAGCCTCCAAGAAATATGGGACTATGTGAAAAGACCAAATCTACATCTGATTGGTGTACCTGAAAGTGATGGGGAGAATGGAACCAAGTTGGAAAACACTGTGCAGGATATTATCCAGGAGAACTTCCCCAATCTAGCAAGGCAGGCCAACATTCAGATTCAGGAAATACAGAGAACACCACAAAGATACTCCTCAAGAAGAGCAACTCCAAGACACATAATTGTCAGATTCACCAAAGTTGAAATGAAGGAAAAAATGTTAAGAGCAGCCAGAGAGAAAGGTCAGGTTACCCTCAAAGGGAAGCCCATTAGGCTAACAGCGGATCTCTCAGCAGAAACTCTACAAGCCAGAAAGAGTGGGGGCCAATATTCAACATTCTTAAAGAAAAGAATTTTCAGCCCAGAATTTCATATCCTGCCAAACTAAGCTTCATAAGTGAAGGAGAAATAAAATACTTTACAGACAAGCAAATGCTGAGAGATTTTGTCACCACCAGGCCTGCCCTAAAAGAGCTCCTGAAGGAAGCGCTAAACATGGAAAGGAACAACCGGTACCAGCCACTGCAAAATCATGCCAAAATGTACAGACCATTGAGACTAGGAAGAAACTGTATCAACTAACGAGCAAAATAACCAGCTAACATCATAATGCCAGGATCAAATTCACACATAACAATATTAACTTTAAATGTAAATGGACTAAATGCTCCAATTAAAAGATACAGACTGGCAAATTGGATAAAGAGTCAAGACCCATCAGTGTGCTGTATTCAGGAAACCCATCTCAGGTGCAGTGACACACATAGGCTCAAAATAAAAGGATGGAGGAAGATCCACCAAGCAAATGGAAAACAAAAAAAGGCAGGGGTTGCAATCCTAGTCTCTGATAAAACAGACTTTAAACCAACAAAGATCAAAAGAGACAAAGAAGGCCATTACATAATGGTAAAGAGATCAATTCAACAAGAAGAGCTAACTATCCGAAATATATATGCACCCAATACAGGAGCATCCAGATTCATAAAGCAAGTCCTGAGTGACCTACAAAGAGACTTAGACTCCCACACATTAATAATGGGAGACTTTAACACCCCACTGTCAACATTAGACAGATCAACGAGACAGAAAGTCAACAAGGATACCCAGGAATTGAACTCAGCTCTGCACCAAGTGGACCTAATAGACATCTACAGAACTCTCCACCCCAAATCAACAGAATATACATTTTTTTCAGCACCACACCACACCTATTCCAAAATTGACCACATACTTGGAAGTGAAGCTCTCCTCAGCAAATATAAGAGAACAGAAATTATAACAAACTGTCTCTCAGACCACAGTGCAATCAAACTAGAACTCAGGATTAAGAAACTCACTCAAAACCACTCAATTACATGGAAACTGAACCAACCTGCTCCTGAATGACTACTGGGTGCATAACGAAATGAAGGCAGAAATAAAGATGTTCTTTGAAACCAATAAGAACAAAGACACAACATACCAGAATCTCTGGGATGCATTCAAAGCAGTGTGTAGAGGGAAACTTATAGCACTAAATGCCCACAAGAGAAAGCAGGAAAGATCCAAAATTGACACCCTAACATCACAGTTGAAAGAACTAGAAAAGCAAGAGCAAACTCATTCAAAAGCTAGCAGAAGGCAAGAAATAACTAAAATCAGAGCAGAACTGAAGGAAATAGAGACACAAAAAACCCTTCAAAAAATTAATGAATCCAGGAGCTGGTTTTTTGAAACGATCAACAAAATTGATAGACCACTAGCAAGACTAATAAAGAAGAAAATAGAGAAGAATCAAATAGACACAATAAAAAATGATAAAGGGGATATCACCACCGATCCCACAGAAATACAAACTACCATCAGAGAATACTACAAACACCTCTACGCAAATAAACTAGAAAATCTAGAAGAAATGGATAAATTCCTGGACACATACACCCTCCCAAGACTAAACCAGGAAGAAGTTGAATCTCTGAATAGACCAATAACAGGCTCTGAAATTGTGGCAATAATCAATAGCTTACCAACCAAAAAGAGTCCAGGACCAGATGGATTCACAGCCGAATTCTACCAGAGGTACAAGGAGGAACTGGTATGCTTCCTTCTGAAACTATTCCAATTAATAGAAAAAGAGGGAATCCTCCCTAACTCATTTTATGAGGCCAGCATCATCCTGATACCAAAGCCGGGCAGAGACACAACCAAAAAAGAGACTTTTAGACCAATATCCTTGATGAACATTGATGCAAAAATCCTCAATAAAATACTGGCAAACCAAATCCAGCAGCACATCAAAAAGCTTATCCACCATGATCAAGTGGGCTTCATCCCTGGGATGCAAGGCTGGTTCAATATACGCAAATCAATAAATGTAATCCAGCATATAAACAGAACCAAAGACAAAAACCACATGATTATCTCAATAGATGCAGAAAAGGCCTTTGACAAAATTCAACAACCCTTCATGCTAAAAACTCTCAATAAATTAGGTATTGATGGGACGTATCTCAAAATAATAAGAGCTATCTATGACAAACCCACAGCCAATATCATACTGAATGGGCAAAAACTGGAAGCATTCCCTTTGAAAACTGGCACAAGACAGGGATGCCCTCTCTCACCACTCCTATTCAACATAGTGTTGGAAGTTCTGGCCAGGGCAATTAGGCAGGAGAAGGAAATAAAGGGTATTCAATTAGGAAAAGAGGAAGTCAAATTGTCCCTGTTTGCAGATGACATGATTGTATATCTAGAAAACCCCATTGTCTCAGCCCAAAATCTCCTTAAGCTGATAAGCAACTTCAGCAAAGTCTCAGGATACAAAATCAATGTACAAAAATCACAAGCATTCTTATACACCAACAACAGACAAACAGAGAGCCAAATCGTGAGTGAACTCCCATTCACAATTGCTTCAAAGAGAATAAAATACCTAGGAATCCAACTTACAAGGGATGTGAAGGACCTTGTCAAGGAGAACTACAAACCAGTGCTCAAGGAAATAAAAAAGGATACAAACAAATGGAAGAACATTCCATGCTCATGGATAGGAAGAATCAATATTGTGAAAATGGCCATACTGCCCAAGGTAATTTACACGTTCGATGCCATCCCCATCAAGCTACCAATGACTTTCTTCACAGAATTGGAAAAAACTACTTTAAAGTTCATATGGAACCAAAAAAGAGCCCGCATCGCCAAGTCAATCCTAAGCCAAAAGAACAAAGCTGGCGGCATCACACTACCTGACTTCAAACTATACTACAAGGCTACAGTAACCAAAACAGCATGGTACTGGTACCAAAACAAAGATATACATCAATAGAACAGAACAGAGCCCTCAGAAATAACGCCGCATATCTACAACTATCTGATCTTTGACAAACCTGAGAAAAACAAGCAATGGGGAAAGGATTCCCTATTTAATAAATGGTGCTGGGAAACCTGGCTAGCCATAGGTAGAAAGCTGAAACTGGATCCCTTCATTACACCTTATACAAAAATTAATTCAAGATGGATTAAAGACTTAAATGTTAGACCTAAAACCATAAAAACCCTAGAAGAAAACCTAGGCTTTGCCATTCAGGACATAGGCATGGACAAGGACTTCATGTCTAAAACACCAAAAGCAATGGCAACAAAAGACAAAATTGACAAATGGGATCTAATTAAACTAAAGAGTTCTGCACAGCAAAAGAAACTACCATCAGAGTGAACAGGCAACCTACAAAATGGGAGAAAATTTTTGCAACCTACTCATCTGACAAAGGGCTAATATCCAGAATCTACAATGAACTCAAACAAATTTACAAGAAAAAAACAACCCCATCAAAAAGTGGGCGAAGGACATGAACAGACACTTCTCAAAAGAAGACATTTATGCAGCCAAAAAACACATGAAAAAATGCTCATCATCACTGGCCATCAGAGAAATGCAAATCAAAACCACAATGAGATACCATCTCACACCAGTTAGAATGGCAATCATTAAAAAGTCAGGAAACAACAGGTGCTGGAGAGGATGTGGAGAAATAGCAACACTTTTACACTGTTGGTGGGACTGTAAACTAGTTCAACCATTGTGGAAGTCAGTGTGGCAATTCCTCAGGGATCTAGAACTAGAAATACCATTTGACCCAGCCATCCCATTAATGGGTATATACCCAAAGGACTATAAATCATGCTGCTATAAAGACACATGTACACATATGTTTATTGCGGCATTATTCACAATAGCAAAGACTTGGAACCAACCCAAATGTCCAACAATGATAGACTGGATTAAGAAAATGTGGCACATATACACCATGGAATACTATACAGCCATAAAAAATGATGAGTTCATGTCCTTTGTAGGGACATGGATGAAATTGGAAATCATCATTCTCAGTAAACTATTGCAAGGACAAAAAATCAAACACTGCATATTCTCACTCATAGGTGGGAATTGAACAATGAGAACACATGGACACAGGAAGGGGAACATCACACTCTGGGGCCTGTTGTGGGGTCGGGGGAGGGGGGAGGGATAGCATTGGGAGATATACCTAATGCTAGATGACGAGTTAGTGGGTGCAGCGCACCAGCATGGCACATGTATACATATGTAACTAACCTGCACATTGTGCACATGTACCCTAAAACTTAACGTATAAT*AATAAAAAATAATAAAAAATAAAAAAAAGAAA****AAGAAAA*TGTGTCAC**ATATACACTGTGGAATACTATGCAGCCATAGAAAAGGATGAGTTCATGTCCTTTGTAGGGAC

**Clone 27; PA3; SpIRE(97/622)-27: Filled site spans: chr3:159084831-159090510**

Empty Site:

CCTCCAAGTTTTCAGCCCTGAACAAGTCTGACTTCCCTCCCATGGTCCACTGACTCCATGAATAGACCTGGACCAGCCCCACCCACCAACTCTACGGACGATGTGCTTCTCAGAACTGAC

CCAGCATCCAACAATCTTGTACAAAC**AGAACTTATG**CATT

CCCCTTGATAACAAGTAACTAGGATGTGCCTCCAAGACCCGAAAGACAGGACTAGAGTAAATCCCCATCAAGACTCAGGATATAAAACCTGGGTCTTAATGGGGTTACCTCCTCTGTCAT

Filled Site:

ACCCACCAACTCTACGGACGATGTGCTTCTCAGAACTGACCCAGCATCCAACAATCTTGTACAAAC**AGAACTTATG**TGAGGGTGGAGGAGCCAAGATGGCCAAATAGGAACAGCTCCGGTCTACAGCTCCCAGCGTGAGCGACGCAGAAGACGGGTGATTTCTGCATTTCCATCTGAGCTTTGAAGAGAGCAGTGGTTCTCCCAGCACGCAGCTGGAGATCTGAGAACGGGCAGACTGCCTCCTTAAGTGGGTCCCTGACCCCTGACCCCTGACCCCCCGAGCAGCCTAACCGGGAGGCACCCCACAGCAGGGGCAGACTGACACCTCACAGGGCCCAGTACTCCAACAGACCTGCAGCTGAGGGTCCTGTCTGTTAGAAGGAAAACTAACAAACAGAAAGGACATCCACACCAAAAACCCATCTTTACATCACCATCATCAAAGACCAAAAGTAGATAAAACCACAAAGATGGGGAAAAAACCGAGCAGAAAAACTGGAAACTCTAAAAAGCAGAGCACCTCTCCTCCTCCAAAGGAATGCAGTTCCTCAACAGCAACAGAACAAAGCTGGATGGAGAATAACTTTGACGAGCTGAGAGAAGAAGGCTTCAGATGATCAAATTACTCTGAGCTACAGGAGGACACTCAAACCAAAGGCAAAGAAGTTGAAAACTTTGAAAAAAATTTAGAAGAATGTATAACTAGAATAACCAATACAGAGAAGTGCTTAAAGGAGCTGATGGAGCTGAAAACCAAGGCTCTAGAACTACGTAAAGAATGCAGAAGCCTCAGGAGCCGGTGAGATCAACTGGAAGAAAGGGTATCAGCAATGGAAGATGAAATGAATGAAATGAAGCGAGAAGGGAAGTTTAGAGAAAAAAGAATAAAAAGAAATGAGCAAAGCCTCCAAGAAATATGGGACTATGTGAAAAGACCAAATCTTCATCTGATTGGTGTACCTGAAAGTGACGGGGAGAATGGAACCAAGCTGGAAAACACTCTGCAGGATATTATCCAGGAGAACTTCCCCAATCTAGCAAGGCAGGCCAACATTCAGATTCAGGAAATACAGAGAACACCACAAAGATACTCCTCGAGAAGAGCAACTCCAAGACACATAATTGTCAGATTCACCAAAGTTGAAATGAAGGAAAAAATGTTAAGGGCAGCCAGAAAGAAAGGTCAGGTTATCCTCAAAGGGAAGCCCATCAGACTAACAGCGGATCTTTCGGCAGAAACGCTACAAGCCAGAAGAGAGTGGGGGCCAATATTCAACATTCTTAAAGAAAAGAATTTTCAACCCAGAACTTCATATCCAGCCAAACTAAGCTTCATAAGTGAAGGAGAACTAAAATACTTTACAGACAAGCAAATGCTGAGAGATTTCGTCACCACCAGGCCTGCCCTAAAAGAGCTCCTGAAGGAAGCACTAAACATGTAAAGGAACAACCGGTACCAGCCACTGCAAAATCATGCCAAAATGTGAAGACCATCGAGACTAGGAAGAAACTGCATCAACTAATGAGCAAAATAACCAGCTAACATCATAATGACAGGATCAAATTCACACATAACAATATTAACTTTAAATGTAAGTGGACTAAATGCTCCAATTAAAAGACACAGACTGGCAAATTGGATAAAGAGTCAAGACCCATCAGGGTGCTGTATTCAGGAAACCCATCTCACGTGCAGAGACACACATAGGCTCAAAATAAAAGGATGGAGGAAGATCTACCAAGCAAATGGAAAACAAAAAAAGGCAGGGGTTGCAATCCTAGTCTCTGATAAAACAGACTTGAAACCAACAAAGATCAAAAGAGACAAAGAAGGCCATTACATAATGGTAAAGGGATCAATTCAACAAGAAGAGCTAACTATCCTGCATATATATGCACCCAATACAGGAGCACCCAGATTCACAAAGCAAGTCCTTAGAGACCTACCAAGAGACTTAGACTCCCACACATTAATAATGGGAGACTTTAGCACCCCACTGTCAACATTAGACAGATCAATGAGACAGAAAATCAACAAGGATACCCAGGAATTGAACTCAGCTCTGCACCAAGTGGACCTAATAGACATCTACAGAACTCTCCACCCCAAATCAACAGAATATACATTTTTTTCAGCACCACACCACACATATTCCAAAATTGACCACATACTTGGAAGTAAAGCTCTCCTCAGCAAATGTAAAAGAACAGAAATTATAACAAACTGTCTCTCAGACCACAGTGCAATCAAACTAGAACTCAGGATTAAGAATCTCACTCAAAACCGCTCAACTACATGGAAACTGAACAACCTGCTCCTGAATGATTACTGGGTACATAAGGAAATGAAGGCAGAAATAAAGATGTTCTTTGAAACCAACGAGAACAAAGACACAACATACCAGAATCTCTGGGACATATTCAAAGCAGTGTGTAGAGGGAAATTTATAGCATTAAATGCCCACAAGAGAAAGCAGGAAAGATCTAAAATTGACACCCTAACATCACAATTAAAAGAACTAGAAAAGCAAGAGCAAACACATTCAAAAGCTAGCAGAAGGCAAGAAATAACTAAAATCAGAGCAGAACTGAAGGAAATAGAGACACAAAAAACCCTTCAAAAAATTAATGAATCCAGGAGCTGGTTTTTTGAAAGGATCAACAAAATTGATAGACCACTAGCAAGACTAATAAAGAAAAAAAGAGAGAAGACTCAAATAGATGCAATAAAAAATGATAAAGGGGATATCACCACCGATCCCACAGAAATACAAACTACCATCAGAGAATACTACAAACACCGCTATGCAAATAAACTAGAAAATCTAGAAGAAATGGATAAATTCCTTGACACATACACTCTCCCAAGACTAAACCAGGAAGAAGTTGAATCTCTGAATAGACCAATAACAGGATCTGAAATTGTGGCAATAATCAATAGCTTACCAACCAAAAAGAGTCCAGGACCAGATGGATTCACAGCTGAATTTTACCAGAGGTACAAGGAGGACTTGGTACCATTCCTTCTGAAACTATTCCAATCAATAGAAAAAGAGGAAATCCTCCCTAACTCATTTTATGAGGCCAGCATCATCCTGATACCAAAGCCAGGCAGAGACACAACCAAAAAAGAGAATTTTAGACCAATATCCTTGATGAACATTGATGCAAAAATCCTCAATAAAATACTGGCAAAACAAATCCAGCAGCACATCAAAAAGCTTATCCACCATGATCAAGTGGGCTTCATCCCTGGGATGCAAGGCTGGTTCAATATATGCAAATCAATAAATGTAATCCAGCATATAAACAGAACCAAAGACAAAAACCACACGATTATCTCAATAGATGCAGAAAAGGCCTTTGGCAAAATTCAACAACCCTTCATGCTAAAAACTCTCAATACATTAGGTATTGATGGGACATATTTCAAAATAATAAGAGCTATCTATCACAAACCCACAGCCAATATCATACTGAATGGGCAAAACCTGGAAGCATTCCCTGTGAAAACTGGCACAAGACAGGGATGCCCTCTCTCACCACTCCTATTCAACATAGTGTTGGAAGTTTTGGCCAGGGCAATTAGGCAGGAGAAGGAAATAAAGGGTATTCAATTAGGAAAAGAGGAAGTCAAATTGTCCCTGTTTGCAGATGACATGATTGTATATCTAGAAAACCCCATTGTCTCAGCCCAAAATCTCCTTAAGCTGATAAGCAACTTCAGTAAGGTCTCAGGATACAAAATCAATGTACAAAAATCACAAGCATTCTTATACACCAATAACAGACAAACAGACAGCCAAATCATGGGTGAACTCCCATTCACAATTGCTTCAAAGAGAATAAAATACCTAGGAATCCAACTTACAAGGGATGTGAAGGACCTCTTCAAGGAGAACTACAAACCACTGCTCAAGGAAATAAAAGAGGATACAAACAAATGGAAGAATATTCCATGCTCACGGGTAGAAAGAATCAATATCGTGAAAATAGCCATACTGCCCAAGGTAATTTACACATTCAATGCCATCCCCATCAAGCTACCAATGACTTTCTTCAAAGAATTGGAAAAAACTACTTTAAAGTTCATATGGAACCAAAAAAGAGCACGCATCGCCAAGTCAATCCTAAGCCAAAAGAACAAAGCTGGAGGCATCACACTACCTGACTTCAAACTATACTACAAGGCTACAATAACCAAAACAGCATGGTACTGGTACCAAAACAGACATATAGATCAATGGAACAGAACAGAGCCCTCAGAAATAACGACACATATCTACAACTATCTGATCTTTGACAAACCTGAGAAAAACAAGCAATGGGGAAAGGATTCCCTATTTAATAAATGGTGCTGGGAAAACTGGCTAGCCATATGTAGAAAGCTGAAACTGGATCCCTTCTTTACACCTTATACAAAAATCAATTCAAGATGGATTAAAGGCTTAAATGTTAGATCTAAAACCATAAAAACCCTAGAAGAAAACCTAGGCATTACCATTCAGGACATAGGCATGGGCAAGGCTTCATGTCTAAAACACCAAAAGCAATAGCAACAAAAGCCAAAATTGACAAATGGTATCTAATTAAACTAAAGAGCTTCTGCACAGCAAAAGAAACTACCATCAGAGTGAACAGGCAACCTATAAAATGGGAGAAAATTTTCACAACCTACTCATCTGACAAAGGGCTAATATCCAGAATCTACAATGAACTCAAACAAATTTACAAGAAAAAAAAAACAACCCCATCAAAAAGTGGGCGAAGGACATGAACAGACACTTCTCAAAAGAAGACATTTATGCAGCCAAAAAACACATGAAAAAATGCTTATCATCACTGGCCATCAGAGAAATGCAAATCAAAACCACATTGAGATACCATCTCACACCAGTTAGAATGGCAATCATTAAAAAGTCAGGAAACAACAGGTGCTGGAGAGGATGTGGAGAAATAGGAACACTTTTACACTGTTGGTGGGACTGTAAACCAGTTCAACCATTGTGGAAGTCAGTGTGGCGATTCCTCAGGGATCTAGAACTAGAAATACCATTTGACCCAGCCATCCCATTACTGGGTATATACCCAAATGACTATAAATCATGCTGCTTTAAAGACACATGCACATGTATGTTTATTGTGGCATTATTCACAATAGCAAAGACTTGGAACCAACCCAAATGTCCAACAATGATAGACTGGATTAAGAAAATGTGGCACATATACACCATGGAATACTATGCAGCCATAAAAAATGATGAGTTCATGTCCTTTGTAGGGACATGGATGAAATTGGAAACCATCATTCTCAGTAAACTATCGCAAGAACAAAAACCCAAACACTGCATATTCTCACTAATAGGTGGGAATTGAACAATGAGAACACATGGACACAGGAAGGGGAACATCACACTCTGGGGACTGTTGTGGGGTGGGGGGAGAGGGGAGGGATAGCATTGGGAGATATACCTAATGCTAGATGACGAGTTAGTGGGTGCAGCACAGCAGCATGGCACATGTATACATATGTAACTAACCTGCACATTGTACACATATACCCTAAAACTTAAAGTATAAT*AATAATAAAAAAAAA****AGAA*CTTATG**CATTCCCCTTGATAACAAGTAACTAGGATGTGCCTCCAAGACCCGAAAGACAGGACTAGAGTAAATCCCCATCA

**Clone 30; PA3; SpIRE(97/622)-30: Filled site spans: chr3:135822428-135828227**

Empty Site:

AGTTAATATGCTTCTTCATGGCCAGGTGCAGTGGTTCACGCCTGTAATCCCAGCACTTTGGGAGGCCAAGGCGGGTGGATCACGAGGTCAGGAGTTCGAGACCAGCCTGATCAACATGGT

GAAACCCTGTCTCTACTA**GAAATACAAAAATT**AGCCGGGC

ATTGGTGGCATGTGCCTGTAATCCCAGCTACTCAGGAGGCTGAGGCAGGAATCGCTTGAACCTGGGATGCAGAGGTTGCAGTGAGCCAAGATCGCGCCACTGCACTCTAGTCTGGGCGAC

Filled Site:

CACGAGGTCAGGAGTTCGAGACCAGCCTGATCAACATGGTGAAACCCTGTCTCTACTA**GAAATACAAAAATT**GAGAAGACAGCCAAGATGGCCAAATAGGAACAGCTCTGGTCTACAGCTCCCAGCGTGAGCGACGCAGAAGATGGGTGATTTCTGCATTTCCATCTGAGCTTTGAAGAGAGCAGTGATCCTCCCAGCACGCAGCTGGAGATCTAAGAATGGGCAGGCTGCCTCCTCAAGTGGGTCCCTGACCCCTGACCCCCGAGCAGCCTAACTGGGAGGCACCCCTCAGTAGGGGCAGACTGACACCTCACACGGCCAGGTACTCCTCTGAGACAAAACTTCCAGAGGAACAATCAGACAGCAGCATTCACGGTTCACGAAAATCTGCTGTTCTGCAGCCACCGCTGCTAATACCCAGGCAAACAGGGTCTGGAGTGGACCTCTAGCAAACTCCAACAGACCTGCAGCTGAGGGTCCTGTCTGTTAGAAGGAAAACTAACAAACAGAAAGGACATCCACACCAAAAACCCATCTGTACATCACCATCATCAAAGACCAAAAGTAGATAAAACCACAAAGATGGGGGAAAAACAGAGCAGAAAAACTGGAAACTCTAAAAAGCAGAGTGCCTCTCCTCCTCCAAAGGAACGCAGTTCCCCACCAGCAACGGAACAAAGCTGGACGGAGAATGACTTTGACGACTTGAGAGAAGAAGGCTTCAGACGATCAAACTACTCCGAGCCACAGGAGGAAATTCAAACCAAAGGCAAAGAAGTTGAAAACTTTGAAAAAAATTTAGACGAATGTATAACTAGAATAACCAATACAGAGAAGTGCTTAAAGGAGCTGATGGAGCTGAAAGCCAAGGCTCCAGAACTACGTGAAGAATGCAGAAGCCTCAGGAGCCGATGCAATCAACTGGAAGAAAGGGTATCAGTGAAGGAAGATGATATGAATGAAATGAAGTGAGAAGGGAAGTTTAGAGAAAAAAGAATAAAAAGAAATGAACAAAGCCTCCAAGAAGTATGGGACTATGTGAAAAGACCAAATCTACGTCTGATTGGTGTACCTGAAAGTGATGGGGAGAATGGAACCAAGTTGGAAAACACTCTGCAGGATATTATCCAGGAGAACTTCCCCAATCTAGCAAGGCAGGCCAACATTCAGATTCAGGAAATACAGAGAACGCCACAAAGATACTCCTCGAGAAGAGCAACTCCAAGACACATAATTGTCAGATTCAACAAAGTTGAAATGAAGGAAAAAATGTTAAGGGCAGCCAGAGAGAAAGGTCGGGTTACCCACAAAGGGAAGCCCATCAGACTAACAGCGGATCTCTTGGCAGAAACTCTACAAGCTAGAAGAGAGTGGGGACCAGTATTCAACATTCTTAAAGAAAAGAATTTTCAACCCAGAATTTCATATCCAGCCAAACTAAGCTTCATAAGTGAAGGAGAAATAAAATCCTTTACAGACAAGCAAATGCTGAGAGATTTTGTCACCACCAGGCCTGCCCTAAAAGAGCTCCTGAAGGAAGCACTAAACATGGAAAGGAACAACTGGTACCAGCCGCTGCAAAATCATGCGAAAATGTAAAGACCATCGAGACTAGGAAGAAACTGCATCAACTAACGAGCAAAATAACCAGCTAACATCATAATGACAGGATCAAATTCACACATAACAATATTAACTTTAAATGTAAATGGACTAAATGTTCCAATTAAAAGACACAGACTGCCAAATTGGATAAAGAGTCAAGACCCATCAGTGTGCTGTATTCAGGAAACCCATCTCACGTGCAGAGACACACATAGGCTCAAAATAAAAGGATGGAGGAAGATCTACCAAGCAAATGGAAAACAAAAAAAGGCAGGGGTTGCAATCCTAGTCTCTGATAAAACAGACTTTAAACCAACAAAGATCAAAAGAGACAAAGAAGGCCATTACATAATGGTAAAGGGATCAATTCAACAAGAAGAGCTAACTATCCTAAATATATATGCACCCAATACAGGAGCACCCAGATTCATAAAGCAAGTCCTGAGTGACCTACAAAGAGACGTAGACTCCCACACAATAATAATAAGAGACTAACACCCCACTGTCAACATTAGACAGATCAACGAGACAGATAGTTAACAAGGATACCCAGGAATTGAACTCAGCTCTGCACCAAGTGGACCTAATAGACATCTACAGAACTCTCCACCCCAAATCAACAAAATATACATTTTGTTCAGCACCACACCACACCTATTCCAAAATTGACCACATAGTTGGAAATAAAGCTCTACTCAGCAAATGTAAAAGAACAGAAATTATAACAAACTGTCTCTCAGACCACAGTGCAATCAAACTAGAACTCAGGATTAAGAAACTCACTCAAAACCGTTCTTGAAACCAACGAGAACAAAGACACAACATACCAGAATCTCTGGGACACATTCAAAGCAGTGTGTAGAGGGAAATTTATAGCACTAAATGCCCAAAAGAGAAAGCAGGAAAGACCCAAAATTGATACCCTAACATCACAATTAAAAGAACTAGAAAAGCAAGAGCAAGCACATTCAAAAGCTAGCAGAAGGCAAGAAATAACTAAAATCAGAGCAGAACTGAAGGAAATAGAGATGCAAAAAAACCCTTCAAAAAATTAATGAATCTAGGAGCTGGTTTTTTGAAAGGATCAACAAAATTGATAGACTGCTAGCAAGACTAATAAAGACAAAAAGAGAGAAGAATCAAAAACATGCAATAAAAAATGATAAAGGGGATATCACCACCGATCCCACAGAAATACAAACTACCATCAGAGAATACTACAAACACCTCTACGCAAATAAACTAGAAAATCTAGAAGAAATGGATAAATTCCTCGACACATACACCCTCCCAAGACTAAACCAGGAAGAAGTTGACTCTCTGAATAGACCAATAACAGTCTCTGAAATTGTGGCAATAATCAATAGCTTACCAACCAAAAAGAGTCCAGGACCAGATGGATTCACAGCCGAATTCTACCAGAGGTACAAGGAGGAACTGGTACCATTCCTTCTGAAACTACTCCAATCAATAGAAAAAGAGGGAATCCTCCCGAACTCATTACATGAGGCCAGCATCATCCTGATACCAAAGCCGGGCAGAGACACAAGCAAAAAAGAGAATTCTAGACCAATATCCTTGATGAACATTGATGCAAAAATCCTCAATAAAATACTGGCAAACCGAATCCAGCAGCACATCAAAAAGCTTATCCACCATGATCAAGTGGGCTTCATCCCTGGGATGCAAGGCTGGTTCAATATATGCAAGTCAATAAATGTAATCCAGCATATAAACAGAGCCAAAGACAAAAGCCACGTGATTATCTCAACAGATGCAGAAAAGGCCTTTGACAAAATTCAACAACTCTTCACGCTAAAAACTCTCAATAAATTAGGTATTGATGGGACGTATCTCAAAATCATAAGAGCTATCTATGACAAACCCACAGCCAATATCATACTGAATGGGCAAAAACTGGAAGCATTCCCTTTGAAAACTGGCACAAGACAGGGATGCCCTCTCTCACCACTCCTATTCAACATAGTGTTGGAAGTTCTGGCCAGGGCAATTAGGCAGGAGAAGGAAATAAAGAGTACTCAATTAGGAAAAGAGGAAGTCAAATTTTCCCTGTTTGCAGACGACATGATTGTATATCTAGAAAACCCCATTGTCTCAGCCCAAAATCTCCTTAAGCTGATAAGCAACTTCAGCAAAGTCTCAGGATACAAAATCAATGTACAAAAATCACAAGCATTCTTATACACCAATAACAGACAAACAGAGAGCCAAATCATGAGTGAACTCCCATTCACAATTGCTTCAAAGAGAATAAAATACCTAGGAATCCAACTTACAAGGGACATGAAGGACCTCTTCAAGGAGAACTACAAACCACTTCTCAAGGAAATAAAAGAGGATACAAACAAATGGAAGAACATTCCATGCTCATGGGTAGGAAGAATCAATATTGTGAAAATGGCCATACTGCCCAAGGTAATTTATAGATTCAATGCCATCCCCATCAAGCTACCAATGACTTTCTTCACAGAATTGGAAAAAACTACTTTAAAGTTCATATGGAACCAAAAAAGAGCCCGCATCGCCAAGTCAATCCTAAGCCAAAAGAACAAAGCTGGAGGCATCACGCTACCTGACTTCAAACTATACTACAAGGCTACAGTAACCAAAACAGCATGGTACTGGTACCAAAACAGAGATATAGATCAATGGAATAGAACAGAGCCCTCAGAAATAATACCACATATCTACAACTATCTGATCTTTGACAAACCTGAGAGAAACAAGCAACGGGGAAATGATTCCCTATTTAATAAATGGTGCTGGGAAAACTGGCTAGCCATATGTAGAAAGCTGAAACTGGATCCCTTCCTTACACCTTATACAAAAATTAATTCAAGATGGATTAAAGACTTAAACGTTAGACCTAAAACCATAAAAACCCTAGAAGAAAACCTAGGCATTACCATTCAGGACATAGGCATGGGCAAGGACTTCATGTCTAAAACACCAAAAGCAATGGCAACAAAAGCCAAAATTGACAAATGGGATCTAATTAAACTAAAGAGCTTCTGCACAGCAAAAGAAACTACCATCAGAGTGAACAGGCAACCCAAAAAATGGGAGAAAATTTTCACAACCTACTCATCTGACAAAGGGCTAATATCCAGAATCTACAATGAACTCAAACAAATTTACAAGAAAAAAACAAACAATCCCATCAAAAAGTGGGCGAAGGATATGAACAAACAGTTCTCAAAAGAAGACATTTATGCAACCAAAAGACACATGAAAAAATGCTCATCATCAACTGGCCATCAGAGAAATGCAAATCAAAACCACAATGAGATACCATCTCACACCAGTTAGAATGGCAATCATTAAAAAGTCAGGAAACAACAGGTGCTGGAGAGGATGTGGAGAAATAGGAACGCTTTTACACTGTTGGTGGGACTGCAAACTAGTTCAACCATTGTGGAAGTCAGTGTGGCGATTCCTCAGGGATCTAGAACTAGAAATACCATTTGACCCAGCCATCCCATTACTGGGTATATACCCAAAGGACTATAAATCATGCTGCTATAAAGACACATGCACACATATGTTTATTGCGGCACTATTCATAATAGCAAAGACTTGGAACCAACACAAATGTCCAACAATGATAGACTGGATTAAGAAAATGTGGCACATATACACCACGGAATACTATGCAGCCATAAAATGATGAGTTCATGTCCTTTGTAGGGACATGGATGAAATTGGAAATCATCATTCTCATTAAACTATCGCAAGGACAAAAAACCAAACACCGCATGTTCTCACTCATAGGTGGGAATTGAACAATGAGAACACATGGACACAGGAAGGGGAACATCACACTCTGAGGACTGTTGTGGGGTTGGGGGAGTGGGGAGGGATAGCATTAGGAGTTATACCTAATGCTAAATGACGAGTTAATGGGTGCAGCACACCAGCATGGCACATGTATACATATGTAACTAATCTGCACATTGTGCACATGTACCCTAAAACTTAAAGTATCATAATAAT*AAAAGAAAAAAAAG****GAAATACAAAAA*TT**AGCCGGGCATTGGTGGCATGTGCCTGTAATCCCAGCTACTCAGGAGGCTGAGGCAGGAATCGCTTGAACCTGG

**Clone 34; PA3; SpIRE(97/622)-34: Filled site spans: chr3:24280028-24285641**

Empty Site:

TTACACATTTAAAAGAATTATTCTGTGAAGGGGTCCATAGACTTCACCAGATAGTTAATAGGGTCCAGCGCACAAAAATGGTTACAAACACTTGGCTGCCTTAGCTCAAAGATAATTATT

TATTATCTTTCCTTCAT**AAGCCATAGAACC**AATTTCTGAT

GCCATATTAATCTTATTTTCTCCCAAGAATACTGGCCATTTAACCCTAACTACCTGGTAACTTCCTATGTCTCAAATTTAACTAAAATGCCCAAGAAAACAGTGCTTCT

Filled Site:

GTTACAAACACTTGGCTGCCTTAGCTCAAAGATAATTATTTATTATCTTTCCTTCAT**AAGCTATAGAACC**GGGGGAGGAGCCAAGATGGCCAAATAGGAACAGCTCTGGTCTACAGCTCCCAGCCTGAGCGACGCAGAAGATGGGTGATTTCTGCATTTCCATCTGAAATTTGAAGAGAGCAGTGGTTCTCCCAGCACGCAGCTGGAGATCTGAGAATGGGCAGACTGCTTCCTCAAGTGGGTCCCTGACTCCTGACCCCCGAGCAGCCTAACTGGGAGGCACACCCCAGCAGGGACAGACTGACACCTCACATGGCCAGGTACTCCAACAGACCTGCAGCTGAGGGTCCTCTCTGTTAGAAGGAAAACTAACAAACAGAAAGGACATCCACACCAAAAACCCATCTGTACATCACCATCATCAAAGACCAAAAGTAGGTAAAACCACAAAGATGGGGAAAAAACAGAGCAGAAAAACTGGAAACTCTAAAAAGCAGAGCGCCTCTCCTCCTCCAAAGGAATGCAGTTCCTCACCAGCAACAGAACAAAGCTGGACGGAGAATGACTTTGACAAGCTGAGAGAAGAAGGCTTCAGACGATCAAATTACTCCAAGCTATGGGAGGACATTCAAACCAAAGGCAAATAAGTTGAAAACTTTGAAAAAAACTTAGAAGAATGTATAACTAGAATAACCAATACAGAGAAGTGCTTAAAGGAGCTGATGGAGCTGAAAACCAAGGCTCGAGAACTACGTGAAGAATGCAGAAGCCTCAGGAGCCCATGCGATCAACTGGAAGAAAGGGTATCAGTGATGGAAGATGAAATGAATGAAATGAAGCGAGAAGGGAAGTTTAGAGAAAAAGGAATAAAAAGAAATGAACAAAGCCTCCAAGAAATATGGGACTATGTGAAAAGACCAAATCTACGTCTGATTGGTGTACCTGAAAGTGATGGGGAGAATGGAACCAAGTTGGAAAACACTCTGCAGGATATCATCCAGGAGAACTTCCCCAATCTAGCAAGGCAGGCCAACGTTCAGATTCAGGAAATACAGAGAACGCCACAAAGATACTCCTCGAGAAGAGCAACACCAAGACACATAATTGTCAGATTCACCAAAGCTGAAATGAAGGAAAAAATGTTAAGGGCAGCCAGAGACAAAGGTCGGGTTACCCTCAAAGGGAAGCCCATCAGACTAACAGCAGATCTCTCGGCAGAAACTCTACAAGCCAGAAGAGAGTGGGGGCCAATATTCAACATTCTTAAAGAAAAGAATTTTCAACCCAGAATTTCATATCCAGCCAAACTAAGCTTCATAAGTGAAGGAGAAATAAAATACTTTACAGACAAGCAAATGCTGAGAGATTTTGTCACCACCAGGCCTGCCCTAGAAGAGCTCCTGAAGGAAGTGCTAAACATGGAAAGGAACTGGTACCAGCCGCTGCAAAATCATGCCAAAATGTAAAGACCATCGAGACTAGGAGGAAACCGCATCAACTAACGAGCAAAATCACCAGCTAACATCATAATGACAGGATCAAATTCACACATAACAATATTAACTTTAAATGTAAATGGACTAAATGCTCCAATTAAAAGACACAGACTGGCGAAATGGATAAAGAGTCAAGACCCATCAGTGTGCTGTATTCAGGAAACCCATCTCACGTGCAGAGACACACATAGGCTCAAAATAAAAGGATGGAGGAAGATCTACCAAGCAAATGGAAAACAAAAAAAGGCAGGGGTTGCAATCCTAGTCTCTGATAAAACAGACTTTAAACCAACAAAGATCAAAAGAGACAAAGAAGGCCATTATTTAATGGTAAAGGGATCAATTCAACAAGAAGAGCTACCTATCCTAAATATATATGCACCCAATACAGGAGCACCCAGATTCATAAAGCAAGTCCTGAGTGACCTACAAAGAGAGTTAGACTCCCACACATTAATAATGGGAGACTTTAACACCCCACCATCAACATTAGACAGATCAACGAGACAGAAAGTCAACAAGGATACCCAGGAATTGAACTCAGCTTTGCACCAAGCAGACCTAATAGACATCTACAGAACTCTCCACCCCAAATCAACAGAATATACATTTTTTTCAGCACCACACCACACCTATTCCAAAATTGACCACATACTTGGAAGTAAAGCTCTCCTCAGCAAATGTAAAAGAACAGAAATTATAAAAAACTATCTCTCAGACCACAGTGCAGTCAAACTAGAACTCAGGATTAAGAATCTCACTCAAAACCGCTCAACTACATGGAAACTGAAAAACCTGCTCCTGAATGACTACTGGGTACATAACGAAATGAAGGCAGAAATGAAGATGTTCTTTGAAACCAACGAGAACAAAGACACAACATACCAGAATCTCTGGGACGCATTCAAAGCAGTGTGTAGAGGGAAATTTATAGCACTAAATGCCCACAAGAGAAAGCAGGAAAGATCCAAAATTGACACCCTAACATCACAATTAAAAGAACTAGAGAAGCAAGAGCAAACACATTCAAAAGCTAGCAGAAGGCAAGAAATAACTAAAATCAGAGCAGAACTGAAGGAAATAGAGACACAAAAACCCTTCAGAAAGTTAACGAATCCAGGAGCTGGTTTTTTGAAAGGATCAACAAAATTGATAGACCGCTAGCAAGACTAATAAAGAAAAAAAGAGAGAACAATCAAATAGACGCAATAAAAAATGATAAAGAGGATATCACCACCGATCCCACAGAAATACAAACTACCATCAGAGAATACTACAAACACCTCTACGCAAATAAACTAGAAAATCTAGAAGAAATGGATAAATTCCTTGACACATACACCCTCCCAAGACTAAACCAGGAAGAAGTTGAATCTCTGAATAGACCAATAACAGGATCTGAAATTGTGGCAATAATCAATACCTTACCAACGAAAGAGTCCAGGACCAGATGGATTCACAGCCGAATTCTACCAGAGGTACAAGGAGGAACTGGTACCATTCCTTCTGAAACTATTCCAATCAATAGAAAAAGAGGGAATCCTCCCTAACTCATTTTATGAGGCCAGCATCATCCTGATACCAAAGCCGGGCAGAGACACAACCAAAAAAGAGAATTTTAGACCAATATCCTTGATGAACATTGATGCAAAAATCCTCAGTAAAATACTGGCAAACCAAATCCAGCAGCACATCAAAAAGCTTATCCACCATGATCAAGTGGGCTTCATCCCTGGGATGCAAGGCTGGTTCAATATATGCAAATCAATAAACATAATCCAGCATATAAACAGAACCAAAGACAAAAACCACGTGATTATCTCAATAGATGCAGAAAAGGCCTTTGACAAAATTCAACAACACTTCATGCTAAAAACTCTCAATAAATTAGGTATTGATGGGACGTATCTCAAAATAATAAGAGCTATTTATGACAAACCCACAGCCAATATCATACTGAATGGGCAAAAACTGGAAGCATTCCCTTTGAAAACTGGCACAAGACAGGGATGCCCTCTCTCACCACTCCTATTCGACATAGTGTTGGAAGTTCTGGCCAGGGCAATTAGGCAGGAGAAGGAAATAAAGGGTATTCAATTAGGAAAAGAGGAAGTCAAATTGTCCCTGTTTGCAGATGACATGATTGTATATCTAGAAAACCCCATTGTCTCAGCCCAAAATCTCCTTAAGCTGATAAGCAACTTCAGCAAAGTCTCAGGATACAAAATCAATGTACAAAAATCACAAGCATTCTTATACACCAGTAACAGACAGAGAGCCAAATCATGAGTGAACTCCCATTCACAATTGCTACAAAGAGAATAAAATACCTAGGAATCCAACTTACAAGGGATGTGAAGGACCTCTTCAAGGAGAACTACAAACCACTGCTCAAGGAAATAAAAGAGGATACAAACAAATGGAAGAACATTCCATGCTCATGGGTAGGAAGAATCAATATCATGAAAATGGCCATACTGCCCAAGGTAATTTACAGATTCAATGCCATCCCCACCAAGCTACCAATGACTTTCTTCACAGAATTGGAAAAAACTACTTTAAAGTTCATATGGAACCAAAAAAGAGCCCGCATCGCCAAGTCAATCCTGAGCCAAAAGAACAAAGCTGGAGGCATCACACTACCTGACTTCAAACTATACTACAAGGCTACAGTAACCAAAACAGCATGGTACTGGTACCAAAACAGAGATATAGATCAACGGAACAGAACAGAGCCCTCAGAAATAACGCCACATATCTACAACTATCTGATCTTTGACAAACCTGAGAAAAACAAGCAATGGGGAAAGGATTCCCTATTTAATCAATGGTGCTGGGAAAACTGGCTAGCCATATGTAGAAAGCTGAAACTGGATCCCTTCCTTACACCTTATACAAAAATCAATTCAAGATGGATTAAAGATTTAAACGTTAGACCTAAAACCATAAAAACCCTAGAAGAAAACCTAGGCATTGCCATTCAGGACATAGGCATGGGGAAAGACTTCATGTCTAAAACACCAAAAGCAATGTCAACAAAAGCCAAAATTGACAAATGGGATCTAATTAAACTAAAGAGCTTCTGCACAGCAAAAGAAACTACCATCAGAGTGAACAGGCAACCTACAGAATGGGAGAAAATTTTTGCAACCTACTCATCTGACAAAGGGCTAATATCCAGAATCTACAATGAACTCAAACAGATTTACAAGAAAAAAACAAACAACCCCATCAAAAAGTGGGCAGAGGACATGAACAGACACTTCTCAAAAGAAGACATTTATGCAGCCAAAAAACACATGAAAAAATGCTCACCATCACTGGCCATCAGAGAAATGCAAATTAAAACCACAAAGAGATACCATTTCACACCAGTTAGAATGGCAATCATTAAAAAGTCAGGAAACAACAGGTGCTGGAGAGGATGTGGAGAAATAGGAACACTTTTACACTGTTAGTGGGACTGTAAACTAGTTCAACCATTGTGGAAGTCAGTGTGGTGATTCCTCAGGGATCTAGAACTAGACATACCATTTGACCCAGCCATCCCATTACTGGGTATATACCCAAAGGACTATAAATCATGCTGCTATAAAGACACATGCACACATATGTTTATTGCGGCATTATTCACAATAGCAAAGACTTGGAACCAACCCAAATGTCCAACAATGATAGACTGGATTAAGAAAATGTGGCACATATACACCATGGAATACTATGCAGCCTTAAAAAAGGATGAGTTCATGTCCTTTGTAGGGACATGGATGAAATTGGAAACCATAATTCTCAGTAAACTATCACAAGAACAAAAAACCAAACACCGCATATTCTCACTCATAGGTGGGAATTGAACTATGAGAACACATGGACACAGGAAGGGGAACATCACACTCTGGGGACTGTTGTGGGGTGGGGGGAGGGGGGAGGGATAGCATTGGGAGATATACCTAATGCTAGAGGACGAGTTAGTGGGTGCAGCACACCAGCATGGCACATGTATACATATGTAACTAACCTGCACATTGTGCACATGTACCCTAAAACTTAAAGTATAAT*TAAAAAAAA****AA*GCCATAGAACC**AATTTCTGATGCCATATTAATCTTATTTTCTCCCAAG

**Clone 36, PA3; SpIRE(97/622)-36: Filled site spans: chr4:77513740-77519509**

Empty Site:

GAAAAAAACAACCCCATCAAAAAGTAGGCAAAGGATATGAACAGACACTTCTCAAAAGAGGACATTTATGCAGCCAACAGACACATGAAAAAATGCTCATCATCACTGGTCATCAGAGAA

ATGCACATCAAACCACAAT**GAGATACCATCTCA**CACCAGT

TAGAATGGAAATAGTTAAAAAGTCAGGAAACAGGCCAGGTGTGGTGGCTCATGCCTGTAATCCCAGCATTTTGGGAGGCTGAGTTGGGTGGATCACAAGGTCAGGAGATGGAGGCCATCCTG

Filled Site:

TTTATGCAGCCAACAGACACATGAAAAAATGCTCATCATCACTGGTCATCAGAGAAATGCACATCAAACCACAAT**GAGATACCATCTCA**TGGGGGAGGAGCCAAGATGGCCGAATAGGAACAGCTCCGGTCTACAGCTCCCAGCGTGAGTGACGCAGAAGACGGGTGATTTCTGCATTTCCATCTGAGCTTTGAAGAGAACAGTGGTTCTCCCAGCACGCAGCTGGAGATCTGAGAACGGGCAGACTGCCTCAAGTGGGTCCCTGACCCCTGACCCCTGAGCAGCCTAACTGGGAGGCATCCCCCAGTAGGGGCAGACTGACACCTCACATGGCCAGGTACTCCTCTGAGACAAAACTTCCAGAGGAACGATCAGACAGCAGCATTCACGGTTCACAAAAAACCACTGCTCTGCAGACACCGCTGCTGATACCCAGGCAAACAGGATCTGGAGTGGACCTCTAGCAAACTCCAACAGACTTGCAGCTGAGGGTCCTCTCTGTTAGAAGGAAAACTAACAAACAGAAAGGACATCCACACCAAAAACCTATCTGTACATCACCATCATCAAAGTCCAAAACTAGATAAAACCACAAAGATGGGGAAAAAACAGAGCAGAAACACTGGAAACTCTAAAAAGCAGAGCACCTCTCCTTCTCCAAAGGATCGCAGTTCCTCACCAGCAATGGAACAAAGCTGGATGGAGAATGACTTTGAAGAGTTGAGAGAAGAAGGCTTCAGACAATCAAACTATGAGCTACAGGAGGAAATTCAAACCAAAGGCAAAGAAGTTAAAAACTTAGAAAAAAATTTAGACGAATGTATAACTAGAATAACCAATATAGAGAAGTGCTTAAAGGAGCTGATGAAGCTGAAAGCCAAGGCTCGAGAACGACGTGAAGAATGCAGAAGCCTCAGGAGCTGATGCGATCAACTGGAAGAAAGGGTATCAGCAATGGAAGATGAAATGAATGAAATGAAGCAAGAAGGGAAGTTTAGAGAAAAAAGAATAAAAAGAAATGAACAAAGCCTCCAAGAAATATGGGACTATGTGAAAAGACCAAATCTACATCTCATTGGTGTACCTGAAAGTGACAGGGAGAATGGAACCAAGTTGGAAAACACTCTGCAGGATATTATCCAGGAGAACTTCCCCAATCTAGCAAGGCAGGCCAACATTCAGATTAGGGAAATACAGAGAACGCCACAAAGATACTCCTCCAGAACAGCAACTCCAAGACACAGAATTGTCAGATTTACCAAAGTTGAAATGAAGGAAAAAATGTTAAGGGCAGCCAGAGAGAAAGGTCGGGTTACCCACAAAGGGAAGCCCATCAGACTAACAGCGGATCTCTCAGCAGAAACTCTACAAGCCAGAAGAGAGTGGGGGCCAATATTCACCATTCTTAAAGAAAAGAATTTTCAACCCAGAATTTCATCTCCAGCCAAACTAAGCTTCATAAATGAAGGAGAAATAAAATCCTTTACAGACAAGCAAATGTCACCACCAGGCCTGCCCTTAAAGAGCTCCTGAAGGAAGCACTAAACGTGGAAAGGAACAACCGGTACCAGCCGCTGCAAAATCATGCCAAAATGTAAAGACCATTGAGACTAAAAAGAAACTGCATCAACTGACGAGCAAAATAACCAGCTGACATCATAATGACTGGTTCAAATTCACACATAACAATATTAACTTTAAATGTAAATGGACTCAATGCTCCAATTAAAAGACACAGACTGGCAAATTGGATAAAGAGTCAAGACCCATCAGTGTGTTGTATTCGGGAAACCCATCTCACATGCAGAGACACACATAGGCTCAAAATAAAGGGATGGAGGAAGATCTACCAAGCAAATGGAAAACAAAAAAAGGCAGGGGCTGCAATCCTAGTCTCTGGTAAAACAGACTTTAAACCAACAAAGATCAAAAGAGACAAAGAAGGCCATTACATAGTGGTAAAGGGATCAATTCAACAAGAAGAACTAACTATCCTAAATATATATGCACCCAATACAGGAGCACCCAGATTCATAAAGCAAGCCCTGAGTGACCTACAAAGAGACTTAGACTCCCACACATTAATAATGGGAGACTTTAACACCCCACTGTCAACATTAGACAGATCAATGAGACAGAAAGTCAACAAGGATACCCAGGAATTGAACTCAGCTCTGCACCAAGTGGACCTAACAGACATCTACAGAACTCTCCACCTCAAATCAACAGAATATACATTTTTTTCAGCACCACACCACACCTATTCCAAAATTGACCACATAGTTGGAAGTAAAGCTCTCCTCAGCAAATGTAAAAGAACAGACATTATAACAAACTATCTCTCAGACCACAGTGCAATCAAACTAGAACTCAGGATTAAGAAACTCACTCAAAACCACTCAACTATATGGAAAATGAACAACCTGCTCCTGAATGACTACTGGGTACATAACAAAATGAAGGCAGAAATAAAGATGTTCTTTGAAACCAACGAGAACAAAGACACAAGATACCAGAATCTCTGGGACACATTCAAAGCAGTGTGTAGAGGGAAATTTATAGCACTAAATGCCCACAAGAGAAAGCAGGAAAGATCCAAAATTGACACCCTAACATCAAATTAAAAGAAGTAGAAAAGCAAGTGCAAACACATTCAAAAGCTAGCAGAAGGTAAGAAATAACTAAGATCAGAGCAGAACTGAAGGAAATAGAGACACAAAAACCCTTCAAAAAATTAATGAATCCAGGAGCTGGTTTTTTGAATGGATCAACAAAATTGATAGACCACTAGCTAGACTAATAAAGAAAAAAAAGAGAGAAGAATCAAATAGACACAATAAAAAATGATAAAGGGGATATCACCACTGATCCCACAGAAATACAAACTACCATCAGAGAATACTACAAACACCTCTATGCAAATAAACTAGAAAATCTAGAAGAAATGGATAAATTCCTCCACACATACACTCTCCCAAGACTAAACCAGGAAGAAGTTGAATCTCTGAATAGACCAATAACAGGAGCTGAAATTGTGGCAATAATCAATAGCTTACCCACTAAAAGGAGTCCAGGACCAGATGGATTCACAGCCGAATTCTACCAGAGGTACAAGGAGGAACTGGTACCATTCCTTCTGAAACTATTCCAATCAATAGAAAAAGAGGGAATCCTCCCTAACTCATTTATGAGGCCAGCATCATCCTGACACCAAAGCCGGGCAGAGACACAACCAAAAAAGAGAATTTTAGACCAATATCCTTGATGAACATTGATGCAAAAATCCTCAATAAAAAACTGGCAAACTGAATCCAGCAGCACATCAAAAAGCTTATCCACCATGATCAAGTGGGCTTCATCTCTGGGATGCAAGTCTGGTTCGATATATGCAAATCAATAAATGTAATCCAGCATATAAACAGAACCAAAGACGAAAACCACATGATTATCTCAATAGATGCAGAAAAGGCCTTTGACAAAATTCAACAGCACTTCACGCTAAAAACTCTCAATAAATTAGGTACTCAAGGGACATATCTCAAAACAATAAGAGCTATCTATGACAAACCCACAGTCAATATCATACTGAATGTGCAAAAACTGGAAGCATTCCCTTTGAAAACTGGTGCAAGACAGAGATGCCCTCTCTCACCACTCCCATTCAACATAGTGTTGGAAGTTCTGGCCAGGGCAATTAGTCAGGAGAAGGAAATAAAGGGTATTCAGTTAGGAAAAGAGGAAGTCAAATTGTCCCTGTTGGCAGATGACATGATGGTATATCTAGAAAATCCCAATGTCTCAGCCCAAAATCTTCTTAAGCTGATAAGCAACTTCAGCAAAGTCTCAGGATACAAAATCAATGTGCAAAAATCACAAGCATTCTTATACACCAATAACAGACAAACAGAGAGCCAAATCATGAGTGAACTCCCATTCACAATTGCTTCAAAGAGAATAAAATACCTAGGAATCCACCTTATAAGGGATGTGAAGGACCTCCTCAAGGAGAACTACAAACCACTGCTCAATGAAATAAAAGAGGATACAAACAAATGAAAGAACCTTCCATGCTCATGGGTAGGAAGAATCAATATCGTGAAAATGGCCATACTGCCCAAGGTAATTTATAGATTCAATGCCATCCCCATCAAGCTACCAATGACTTTCTTCACAGAATTGGAAAAAACTACTTTAAAGTTCATATGGAACCAAAAAAGAGCCCACATCGCCATGTCAATCCTAAGCCAAAAGAACAAAGCTGGAGGCATCACGCTACCTGACTTCAAACTATACTACAAGGCTACAGTAACCAAAACAGCATGGCACTGGTACTAAAACAGAGATATAGATCAATGGAACAGAACAGAGCCCTCAGAAATAACTCCGCATATCTACAACCATCTGATCTTTGACAAACCTGACAAAAACAAGCAATGGGGAAAGGATTCCCTATTTAATAAATGGTGCTGGGAAAACTGGCTAGCCATATGTAGAAAGCTGAAACTGGATCCCTTCCTTACACCTTATACAAAAATCAATTCAAGATGGATTAAAGACTTAAACGTTAGACCTAAAACCATAAAAATCCTAGAAGAAAACCTAGACATTACCATTCAGGACATAGGCACGGGTAAGGACTTCATGTCTAAAACACCAAAAGCAATGGCAACAAAAGCCAAAATTGACAAATGGGATCTAATTAAACTAAAGAGCTTCTGCACAGCAAAAGAAACTACCATCAGAGTGAACAGGCATCCCACAAAATGGGAGAAAATTTTCGCAACTTACTCATCTGACAAAGGGCTAATATCCAGAATCTACAATGAACTCAAACAAATTTACAAGAAAAAAACAAACAACCCCATCAAAAAGTGGGCGAAGGACATGAACAGACACTTCTCAAAAGAAGACATTTATGCAGCCAAAAAACACATGAAAAAATGCTCACCATCACTGGCCATCAGAGAAATGCAAATCAAAACCACAATGAGATATCATCTCACACCAGTTAGAATGGCAATCATTAAAAAGTCAGGAAACAACAGGTGCTGGAGAGGATGTGGAGAAATAGGAACACTTTTACACTGTTGGTGGGACTGTAAACTAGTTCAACCATTGTGGAAGTCAGTGTGGTGATTCCTCAGGGATCTAGAACTAGAAATACCATTTGACCCAGCCATCCCATTACTGGGTATATACCCAAAGGACTATAAATCATGCTGCTATAAAGACACATGCACACATATGTTTATTGCAGCACTATTCACAATAGCAAAGACTTGGAACCAACCCAAATGTCCAACAATGACAGACTGGATTAAGAAAATGTGGCACATATACACCATGGAATACTATGCAGCCATAAAAAATGATGAGTTCATGTCCTTTGTAGGGACATGGATGAAATTGGAAATCATCATTCTCAGTAAACTATTGCAAGAACAAAAAACCAAACACCACATATTCTCGCTCATAGGTGGGAATTGAACAATGAGAACACATGGACACAGGAAGGGGAACATCACACTCTGGGGACTGTTGTGGGGTGGGGGTAGTGGGGAGCGATAGCACTGGGAGATATACCTAATGCTAGATGACGAGTTAGTGGTTGCAGCACACCAGTATGTCACATGTATACATATGTAACTAACCTGCACATTGTGCACATGTACCCTAAAACTTAAAGTAT*AAAAAAAAAAAAAAAA***GAGATACCATCTCA**CACCAGTTAGAATGGAAATAGTTAAAAAGTCAGGAAACAGGCCAGGTGTGG

**Clone 39; PA3; SpIRE(97/622)-39: Filled site spans: chr4:115172286-115177975**

Empty Site:

CCTGCTTATGATACCCACTGTTGTTTTTATTTTGCTTTTTTTAATACCAGAGAAAAAATATTTTACAATTTGTTATGTTTTCTTCACTAGCGTCTAAACTCTGGAGGAGACTATATTTTG

TTCACTCTCATCTTCCCATTGCCTGAAAT**AGTGCTT**TCCA

TGTAATAGTTTTCTAATTAATGTGTTGAATGAATTAATAAATTTGTTATTCCTTACAAAACCCATAGGTTAGATGATGTCATCAAAATAAAATGTTTTTAGAGAAAAGAGCTGAAGCTGA

Filled Site:

TTATGTTTTCTTCACTAGCGTCTAAACTCTGGAGGAGACTATATTTTGTTCACTCTCATCTTCCCATTGCCTGAAAT**AGTGCTT**GGGGTCGGGGGGAGCCAAGATGGCCAAATAGGAACAGCTCCGGTCTACAGCTCCCAGCGTGAGCGACGCAGAAGATGGGTGATTTCTGCATTTCCATCTGAGCTTTGAAGAGAGCAGTGGTTCTCCCAGCACGCAGCTGGAGATCTGAGAACGGTCAGACTGCCTCCTCAAGTGGGTCCCTGACCCCTGACCCCCGAGCAGCCTAACTGGGAGGCACCCTCCAGCAGGGGCAGACTGACACCTCACACGGCCGGGTACTCCAACAGACCTGCAGCTGAGGGTCCTGTCTGTTAGAAGGAAAACTAACAAACAGAAAGGACATCCACACCAAAAACCCATCTGTACATCACCATCATCAAAGACCAAAAGTAGATAAAACCACGAAGATGGGGAAAAAACAGAGCAGAAAAACTGGAAACTCTAAAAAGCAGAGCACCTCTCCTCCTCCAAAGGAACGCAGTGCTTCACCAGCAATGGAACAAAGCTGCACGGAGAATGACTTTGACGAGCTGAGAGAAGAAGGCTTCAGATGATCAAATTACTCTGAGCTATGGGAGGAAATTCAAACCAAAGGCAAAGAAGTTGAAAACTTTGAAAAAAGTTTAGAAGAATGTATAACTAAAATAACCAATACAGAGAAGTGCTTAAAGGAGCTGATGGAGCTGAAAACCAAGGCTGGAGAACTACGTGAGGAATGCAGAAGCCTCAGGAGCCGATGCGATCAACTGGAAGAAAGGGTATCAGTGCTGGAAGATGAAGTGAATGAAATGAAGCGAGAAGGGAAGTTTAGAGAAAAAAGAATAAAAAGAAATGAGCAAAGCCTCCAAGAAATATGGGACTATGTGAACAGACCAAATCTACGTCTGATTGGTGTATCTGAAAGTGACGGGGAGAATGGAACCAAGTTGGAAATCACTCTGCAGGATATTATCCAGGAGAACTTCCCCAATCTAGCAAGGCAGGCCAACATTCAGATTCAGGAAATACAGAGAACACCACAAAGATACTCCTCGAGAAGAGCAGCTCCAAGACACATAATTGTCAGATTCACCAAAGTTGAAATGAAGGAAAAAATGTTAAGGGCAGCCAGAGAGAAAGGTTGGGTTACCCACAAAGGGAAGCCCATCAGACTCACAGCAGATCTCTCGGCAGAAACTCTACAACCCAGAAGAAAGTGGGGGCCAATATTCAACAGTCTTAAAGAAAAGAATTTTCAACCCAGAATTTCATATCCAGCCAAACTAAGCTTCATAAGTGAAGGAGAAATAAAATACTTTACAGACAAGCAAATGCTGAGAGATTTTCTCACCACCAGGCCTGCCCTAAAAGAGCTCCTGAAGGAAGCGCTAAACATGGAAAGGAACAACCAGTACCAGCCGCTGCAAAATCATGTCAAAATGTAAAGATCATCGAGACTAGGAAGAAACTGCATCAACTATCGAGCAAAATAACCAGCTAACATCATAATGACAGGATCGAATTCACACATAACAATATTAACTTTAAATGTCAATGGACTAAATGCTCCAATTAAAAGACACAGACTGGCAAATTGGATACGGAGTCAAGACCCATCAGTGTGCTGTATTCAGGAAACCCATCTCACGTGCAGAGACACACATAGGCTCAAAATAAAAGGATGGAGGAAGATCTACCAAGCAAATGCAAAACAAAAAAAGGCAGGGGTTGCAATCCTAGTCTCTGATAAAACAGACTTTAAACCAACAAAGATCAAAAGAGACAAAGAAGGCCATTACATAATGCTAAAGGGATCAATTCAACAAGAAGAGCTAACTATCCTAAATATAAATGCACCCAATACAGGAGCACCCAGATTCATAAAGCAAGTCCTGAGTGACCTACAAAGAGACTTAGACTCCCACACATTAATAATGGGAGACTTTCACACCCCACTGTCAACATTAGACAGATCAACGAGACAGAAAGTCAACAAGGATACCCAGGAATTGAACTCAGCTCTGCACCAAGCGGACCTAATAGACATCTACAGAACTCTCCATCCCAAATCAACAGAGTATACATTTTTTTTCAGCACCACACCACACCTATTCCAAAATTGACCACATACTTGGAAGTAAAGCTCTCCTCAGCAAATGTAAAAGAACAGAAATTATAACAAAGTATCTCTCAGACCACAATGCAATCAAACTAGAACTCAGGATTAAGAATCTCACTCAAAACCGCTCAACTACATGGAAACTGAACAACCTGCTCCTGAATGACTACTGGGTACATAACGAAATGAAGGCAGAAATAAAGATGTTCTTTGAAACCAACGAGAACAAAGACACAACATACCAGAATCTCTGGGACACATTCAAAGCAGTGTGTAGAGGGAAATTTATAGCACTAAATGCCCACAAGAGAAAGCAGGAAAGATCCAAAATTGACACCCTAACATCACAATTAAAAGAACTAGAAAACCAAGAGCAAACACATTCAAAAGCTAGCAGAAGGCAAGAAATAAGTTAAACCAGAGCAGAACTGAAGGAAATAGAGACACAAAAAACCCTTCAAAAAATTAATGAATCCAGCAGCTGGTTTTTTGAAAGGATCAACAAAATTGATAGACCGCTAGCAAGACTAATAAAGAAAAAAAGAGAGAAGAATCAAATAGACGCAATAAAAAATGATAAAGGGGATATCACCACCGATCCCACAGAAATACAAACTACCATCAGAGAATACTACAAACACCTCTACGCAAATAAACTAGAAAATCTAGAAGAAATGGATACATTCCTCTACACATACACTTTCCCAAGACTAAACCAGGAAGAAGTTGAATCTCTGAATAGACCAATAACAGGAGCTGAAATTGTGGCAATAATCAATAGCTTACCAACCAAAAAGAGTCCAGGACCAGATGGATTCACAGCATAATTCTACCAGAGGTACAAGGAGGAACTGGTACCATTCCTTCTGAAACTATTCCAATCAATAGAAAAAGAGGGAATCCTCCCTAATTCATTTTATGAGGCCAGCATCATCCTGATACCAAAGCCGGGCAGAGACACAACCAAAAAAGAGAATTTTAGACCAATATCCTTGATGAACATTGATGTAAAAATCCTCAATAAAATACTGGCAAACCGAATCCAGCAGCACATCAAAAAGCTTATCCACCATGATCAAGTGGGCTTCATCCCTGGGATGCAAGGCTGGTTCAATATACGCAAATCTATAAATGTAATCCAGCATATAAACAGAGCCAAAGACAAAAACCACATGATTATCTCAATAGATGCAGAAAAGGCCTTTGACAAAATTCAACAACCCTTCATGCTAAAAACTCTCAATAAATTAGGTATTGATGGGACGTATCTCAAAATAATAAGAGCTACCTATGACAAACCCACAGCCAACATCATACTGAATGGGCAAAAACTGGAAGCATTCCCTTTGAAAACTGGCACAAGACAGGGATGCCCTCTCTCACCACTCCTATTCAACATAGTGTTGGAAGTTCTGGCCAGGGCAATTAGGCAGGAGAAGGAAATAAAGGGTATTCAATTAGGAAAAGAGGAAGTCAAATTGTCCCTGTTTGCAGACGACATGATTGTGTATCTAGAAAACCCCATTGTCTCAGCCCAAAATCTCCTTAAGCTGATAAGCAACTTCAGCAAAGTCTCAGGATGCAAAATCAAGAACAAAAATCACAAGCATTCTTATACACCAACAACAGACAAACGGAGAGCCAAATCATGAGTGAACTCCCATTCACAATTGCTTCAAAGAGAATAAAATACCTAGGAATCCAACTTACAAGGGATGTGAAGGACCTCTTCAAGGAGAACTACAAACCACTGCTCAAGGAAATAAAAGAGGATACAAAGAAATGGAAGAACATTCCATGCTCATGGGTAGGAAGAATCAATATCGTGAAAATGGCCATACTGCCCAAGGTGATTTACAGATTCAATGCCATCCCCATCAAGCTACCAATGACTTTCTTCACAGAATTGGAAAAAACTACTTTAAAGTTCATAGGGAACCAAAAAAGAGCCCACATCGCCAAGTCAATCCTAAGCCAAAAGAACAAAGCTGGAGGCATCACACTACCTGACTTCAAACTATACTACAAGGCTACAGTAACCAAAGCACCATGGTACTGGTACCAAAACAGAGATATAGATCAATGGAACAGAATGGAGCCCTCAGAAATAACGCCACATATGTACAACTATCTGATCTTTGACAAACCTGAGAAAAACAAGCAATGGGGAAAGGATTCCCTATTTAACAAATGGTGCTGGGAAAACTGGCTAGCCATATGTAGAAAGCTGAAACTGGATCCCTTCCTTACATCTTATACAAAAATCAATTCAAGATGGATTAAAGACTTAAACGTTAGACCTAAAACCATAAAAACCCTAGAAGAAAACCTAGGCATTACCATTCAGGACATAGGCATGGGCAAGGACTTCATGTCTAAAACACCAAAAGCAATGGCAACAAAAGCCAAAATTGACAAATGGGATCTCATTAAACTAAAGAGCTTCTGCACAGCAAAAGAAACTACCATCAGAGTGAACAGGCAACCTACAAAATGGGAGAAAATTTTTGAAACCTACTCATCTGACAAAGGGCTAATATCCAGAATCTACAATGAACTCAAACAAATTTACAAGAAAAAAACAAACAACCCCATCAAAAAGTGGGCAAAGGACATGAACAGACACTTCTCAAAAGAAGACATTTATGCAGCCAAAAAACACATGAAAAAATGCTCACCATCACTGGCCATCAGAGAAATGCAAATCAAAACCACAATGAGATACCATCTCACACCAGTTAGAATGGCAATCATTAAAAAGTCAGGAAACAACAGGTGCTGGAGAGGATGTGGAGAAATAGGAACACTTTTACACTGTTGGTGGGACTGTAAACTAGTTCGACCATTGTGAAAGTCAGTGTGGCGATTCCTCAGGGATCTAGAACTAGAAATATCATTTGACCCAGCCATCCCATTATTGGGTATATACCTAAAGGACTATAAATCATGCTGCTATAAAGACACATGCACACGTATGTTTATTGCAGCATTATTCACAATAGCAAAGACTTGGAACCAACCCAAATGTCCAACAATGATAGACTGGATTAAGAAAATGTGGCACATATACACCATGGAATACTATGCAGCCATAAAGAATGATAAGTTCATGTCCTTTGTAGGGACATGGATGAAATTGGAAATCATCATTCTCAGTAAACTATCGCAAGGACAAAAAACCAAACACCGCATATTCTCACTCATAGGTGGGAACCGAACAATGAGAACACATGGACACAGAAAGGGGAACATGACACTCCCGGGACTGTTGTGCAGTGGGGGGAGGGGGGAGGGATAGCATTGGGAGATATACCTAATGCTAGATGACGAGTTAGTGGGTGCAGCGCACCAGCATGGCACATGTATACATATGTAACTAACCTGCACAATGTGCACATGTACCCTAAAACTTAAAGTATAAT*AATAAGTTAAACATTAAAAAATTAAAAAAAA*G**AGTGCTT**TCCATGTAATAGTTTTCTAATTAATGTGTTGAATGAATTAATAAATTTGTTATTCCTTACAAAACCCA

**Clone 42; PA3; SpIRE(97/622)-42: Filled site spans: chr4:127860001-127866130**

Empty Site:

CTAACAGAGTCCAATGGCATATCAAACAGATAATCCACTACGATCAAGTGGGTTTCATACCAGGAATGCAGGGATGATTTAATATCCACAATTCAATAAATGTGATACACCACATAAACA

GAATTAAAAACGAA**AGTCA**TATGATCATCTCAATAGATGC

AGAAAAAGCATCTGATAAAATCCAGCATTGCTTTATGATTAAAACCCTCAGCAAAATCAGCATAAAAGGGACATACTGAAAGGTAATAAAAGCCATCTATGACAAACCATAGCCAACAAT

Filled Site:

AATATCCACAATTCAATAAATGTGATACACCACATAAACAGAATTAAAAACGAA**AGTCA**GGAGGAGCCAAGACGGCCAAATAGGAACAGCTCCAGTCTACAGCTCCCAGCGTGAGCGACGCAGAAGACGGGTGATTTCTGCATTTCCAACTGAGCTTTGAAGAGAGTAGTGGTTCTCCCAGCACGCAGCTTGAGATCTGAGAACACGCAGACTGCCTCCTCAAGTGGGTCCCTGACCCCTGACCCCCGAGTAGCCTAACTGGGAGGCACTCCCCAGTAGGGGCAGACGGACACCTCACACGGCCGGGTACTTCTCTGAGACAAAACTTCCAGAGGAACGATCAGACAGCAGCATTCGCGGTTCATGAAAATCCGCTGTTCTGCAGACACTGCTGCTGATAACCAGGCAAACAGGGTCTGGAGTGGACCTCCAGCAAACTCTAACAGACCTGCAGCTGAGGGTCCTGTCTGTTAGAAGGAAAACTAACAAACAGAAAGGACATCCACACCAAAAACCCATCTGTACATCACCATCATCAAAGACCAAAAGTAGATAAAGCCACAATGATGGAGAAAAAACAGAGCAGAAAAACTGGAAACTCTAAAAAGCAGAGCGCCTCTCCTCCTCCAAAGGAACACAGTTCCTCTCCAGCAACGGAACAAAGCTGGACAGAGAATGACTTTGACGAGTTGAGAGAAGAAGGCTTCAGACGATCAAACTACTCCGAGCTACAGGAGGAAATTCAAACCAAAGGCAAAGAAATTGAAAACTTTGAAAAAAATTTAGATGAATGTATAACTAGAATAACCAATACAGAGAAGTGCTTAAAGGAGCTGTTGGAGCTCAAAGCCAAGGCTCGAGAACTACGTGAAGAATGCAGAAGCCTCAGGAGCCAATGCGATCAACTGGAAGAAAGGGTATCAGTGATGGAAGATGAAATGAATGAAATGAAGCAAGAAGGGAAGTTTAGAGAAAAAAGAATAAAAGGAAACGAACAAAGCCTCCAAGAAATATGGGACTATGTGAACAGACCAAATCTACGTCTGATTGGTGTACCTGAAAGTGATGGAGAGAATGGAACCAAGCTGGAAAACACTCTGCAGGATATTATCCAGGAGAACTTCCCCAATCTAGCAAGGCAGGTCAACATTCAGGTAATACAGAGAATGCCACAAAGATACTCCTCGAGAAGAGCAACTCCAAGACACATAATTGTCAGATTCACCAAAGTGGAAATGAAGGAAAAAATGTTAAGGGCAGCCAGAGAGAAAGGTCGGGTTACTCACAAAGGGAAGCCCATCAGACTAACGGTGGATCTCTCGGCAGAAACTCTACAAGCCAGGAGAGGGTGGGGGCCAATATTCAACATTCTTAAAGAAAAGAATTTTCAACCCAGAATTTCATATCCAGCCAAACTAAGCTTCACAAGTGAAGGAGGAATAAAATACTTTACAGACAAGCAAATGCTGAGAGATTTTGTCACCACCAGGCCTGCCCTAAAAGAGCTCCCGAAGGAAGCACTAAACATGGAAAGGAACAACCGGTACCAGCCGCTGCAAAATCATGCCAAAATGTAAAGACCATCGAGACTAGGAAGAAACTGCATCAACTAATGAGCAAAATAACTACCTAACATCATAATGACAGGATCAAATTCACACATAACAATATTAACTTTAAATGTAAATGGACTAAATGCTCCAATTAAAAGACACAGACTGGCAAATTGGATAAAGAGTCAAGACACATCAGTGTGCTGTATTCAGGAAACCCATCTCACGTGCAGAGACACACATAGGCTCAAAATAAAAGGATGGAGGAAGATCTACCAAGCAAATGGAAAACAAAAAAAAGGCAGGGGTTGCAATCCTAGTCTCTGATAAAACAGACTTTAAACCAACAAAGATCAAAAGAGACAAAGAAGGCCATTACATAATGGTAAAGCGATTAATTCAACAAGAAGAGCTAACTATCCTAAATATATATGCACCCAATACAGGAGCACCCAGATTCATAAAACAAGTCCTTAGTGACCTACAAAGAGACTTAGACTCCAACACAATAATAATGGGAGACTTTAACACCCCACTGTCAACATTAGACACATCAACAAGACAGAAAGTTAACAAGGATACCCAGGAATTGAACTCAGCTCTGCACCAAGCGGACCTAACAGACATCTACAGAACTCTCCACCCCAGATCAACAGAATATACATTTTTTTTAGCACCACACCACACCTATTCCAAAATTGACCACATACTTGGAAGTAAAGCTCTCCTCAGCAAATGTAAAAGGACAGAAATTATAACAAACTGTCTCTCAGACCACAGTGCAATCAAACTACAACTCAGGATTAAGAAACTCACTCAAAACTGCTCAACTACATGGAAACTGAACAACCTGCTACTGAATGACTACTGGGTACAGAACAAAATGAAGGCAGAAATAAAGATGTTCTTTGAAACCAACGAGAACAAAGACACAACATACCAGAATCTCTGGGACACATTCAAAGCAGTGTGTAGAGAGAAATTTATAGCACTAAATGCCCACAAGAGAAAGCAGGAAAGATCCAAAATTGACACCCTAACATCACAACTAAAAGAACTAGAAAAGCAAGAGCAAACACATTCAAAAGCTAGCAGAAGGCAAGAAATAACTAAAATCAGAGCAGAACTGAAGGAAATAGAGACACAAAAAAAACCCTTCAAAAAATTAATGAATCCAGGAGCTGATTTTTTGAAAGGATCAACAAAATTGATAGACCGCTAGCAAGACTATTAAAGAAGAAAAGAGAGAAGAATCAAATAGACACAATAAAAAATGATAAAGGGGATATCACCACCGATCCCACAGAAATACAAACTACCATCAGAGAATACTACAAACACCTCTATGCAAATAAACTAGAAAATCTAGAAGAAATGGATAAATTCCTCGACACATACATCCTCCCAAGACTAAACCAGGAAGAAGTTGAATCTCTGAATAGACCAATAACAGGCTCTGAAATTGTGGCAATAATCAATAGCTTACCAACCAAAAAGAGTCCAGGACCAGATGGATTCACAGCTGAATTCTACCAGAGGTACAAGGAGGAACTGGTACCATTCCTTCTGAAACTATTCCAATCAATAGAAAAAGAGGGAATCCTCCCTAACTCATTTTATGAGGCCAGCATCATCCTGATACCAAAGCCAGGCAGAGACACAACCAAAAAAGAGAATTTTAGACCAATATCCTTGATGAACATTGATGCAAAAATCCTCAATAAAATACTGGCAAACCGAATCCAGCAGCACATCAAAAAGCTTATCCACCATGATCAAGTGGGCTTCATCCCTGGGATGCAAGGCTGGTTCAACATATGCAAATCAATAAATGTAATCCAGCATATAAACAGAACCAAAGACAAAAACCACATGATTATCTCCATAGATGCAGAAAAGGCCCTTAATAAAATTCAGCAGCCCTTCATGCTAAAAACTCTCAATAAATTAGGTATCGATGGGATGTATCTCAAAATAATAAGAGTTATCTATGACAAACCCACAGCCAATATCATACTGAATGGACAAAAACTGGAAGCATTCCCTTTGAAAACTGGCACAAGACAGGGATGCCCTCTCTCACCACTCCTATTCAACATAGTGTTGGAAGATCTGGCCAGGGCAATTAGGCAGGAGAAGGAAATAAAGGGTATTCAATTAGGAAAAGAGGAAGTCAAATTGTCCCTGTTTGCAGATGACATGATTGTATATCTAGAAAACTCCATTGTCTCAGCCCAAAATCTCCTTAAGCTGATCAGCAACTTCAGCAAAGTCTCAGGATACAAAATCAATGTACAAAAATCACAAGCATTCTTATACACCAATAACAGACAAACAGAGAGCCAAATCATGAGTGAACTCCCATTCACAATTGCTTCAAAGAGAATAAAATACCTAGGAATCCAACTTGCAAGGGATGTGAAGGACCTCTTCAAGGAGAACTACAATCCACTGCTCAATGAAATAAAAGAGGATACAAACAAATGGAAGAACATTCCATGCTCATGGGTAGAAAGAATCAATATCGTGAAAATGGCCATACTGCCCAAGGTAATTTATAGATTCAATGCCATCTCCATCAAGCTACCAATGACTTTCTTCACAGAATTGGAAAAAACTACTTTAAAGTTCATATGGAACCAAAAAAGAGCCCGCATTGCCAAGGCAATCCTAAGCCAAAAGAACAAAGCTGGAGGCATCACGCTACCTGACTTCAAACTATACTACAAGGCTACAGTAACCAAAACAGCATGGTACTGGTACCAAAACAGAGATATAGATCAATGGAACAGAACAGAGCCCTCAGAAATGACGCCGCATATCTACAACTATCTGATCTTTGACAAACCTGACAAAAACAAGAAATGGGGAAAGGATTCCCTATTTAATAAATGGTGCTGGGAAAACTGGCTAGCCATATGTAGAAAGCTGAAACTGGATCCCTTCCTTACACCTTACACAAAAATTAATTCAAGATGGATTAAAGACTTAAACGTTACACCTAAAACCATAAAAACCCTAGAAGAAAACCTAGGCATTACCATCCAGGACATAGGCATGGGCAAGGACTTCATGTCTAAAACACCAAAAGCAATGGCAACAAAAGACAAAATTGACAAATGGGATCTAATTAAACTAAAGAGCTTCTGCACAGCAAAAGAAACTACTATCAGAGTGAACAGGCAACCTACAAAATGGGAGAAAATTTTCGCAACCTACTCATCTGACAAAGGGCTAATATCCAGAATCTACAATGAACTCAAACAAATTTACAAGAAAAAAACAAACAACCCCATCAAAAAGTGGGCAAAGGACATGAACAGATACTTCTCAAAAGAAGACATTTATGCAGCCAAAAAAAACATGAAAAAATGCTCACCATCACTGGCCATCAGAGAAATGCAAATCAAAACCACAATGAGATACCATCTCAGCAGTTAGAATGGCAATCATTAAAAAGTCAGGAAACAACAGGTGCTGGAGAAGATGTGGAGAAATAGGAACACTTTTACACTGTTGGTGGGACTGTAAACTAGTTCCACCATTGTGGAAGTCAGTGTGGCGATTCCTCAGGGATCTAGAACTAGAAATACCATTTGACCCAGCCGTCCCATTACTGGGTATATACCCAAAGGACTATAAATCATGCTGCTATAAAGACACATGCACACGTATGTTTATTGCGGCACTATTCACGATAGCAAAGACTTGGAACCAATCCAAATGTCCAACAATGATAGACTGGATTAAGAAAATGTGGCACATATACACCATGGAATACTATGCAGCCATAAAAAATGATGAGTTCATGTTCTTTGTAGGGACATGGATGAAATTGGAAATCATCATTCTCAGCAAACTATCGCAAGGACAAAAAACCAAACACCGCATGTTCTCACTCATAGGTGGGAATTGAACAATGAGAACACATGGACAGAGGAAGGGGAACATCACACTCTGGGGACTGTTGTGGGGTGGGGGGAGGGGGGAGGGGGGAGGGATAGCATTAGGAGATATACCTAACGCTAAATGATGAGTTAATGGGTGCAGCACACCAGCATGGCACACATATACATATGTAACTAACCTGCACATTGTGCACATGTAGCCTAAAACTTAAAGTATAAT*AATAATTTTAAAATAAATAAATAAATAA*ATGATGAGTTCATGTCCTTTGTAGGGACATGGATGAAACTGGAAACCATCGTTCTCAGCAAACTATCGCAAGGACAAAAAACCAAACACCACATGTTCTCACTCATAGGTGGGAATTGAACAATGAGAACACATGGACACAGGAAGGGGAACATCACACACAGGGAACTGTTGTGGGGTGGGGGAAGAGGGAGGGATAGCATTAGGAGATATACCTAATGCTAAATGACGAGTTAATGGGTGCAGCACACCAACATGGCACATGTATACATATGTAAAAAACCTGCACATTGTGCACATGTACCCTAAAACTTAAAGTATAATAATAATAAA*ATTTTAAAAAG****A*GTCA**TATGATCATCTCAATAGATGCAGAAAAAGCATCTGATAAAATCCAGCATTGCTTTATGATT

**Clone 43; PA3; SpIRE(97/622)-43: Filled site spans: chr5:133180623-133186302**

Empty Site:

TAACAGAGTGAGACCCCATCTAAAAAAAAAAAAAAAAAAGAAAGTTTGGGAGGACTTACTCTATCAAATACAAGACTTAATATAAAGTTATAGTAATTAAGACAGTATGTAACTGAGCAA

CATTAGACAAACAGGTCAATGAAACA**ATATAGGAATC**AGA

AATAAATATATGTATATATGAAAATGTTATCTATAGCAGAGGTAGCACTCCAGATTGGTGGGGAAAAGACAAACTTTTTCAATAAATGTTGCTGGAATACATACACCAGGGTACATGTT

Filled Site:

TATAAAGTTATAGTAATTAAGACAGTATGTAACTGAGCAACATTAGACAAACAGGTCAATGAAACA**ATATAGGAATC**GATGGGAGGAGCCAAGATGGCCGAATAGGAACAGCTCCGGTCTACAGCTCCCAGCGTGAGCGACGCAGAAGACAGATGATTTCTGCATTTCCATCTGAGCTCTGAAGACAGCAGTGGTTCTCCCAGTACGCAGCTGGAGATCTCAGAACGGGCAGACTGCCTCCTCAAGTGGGTCCCTGACCCCTGACCCCCGAGCAGCCTAACTGGGAGGCACCCCCCAGCAGGGGCACACTGACACCTCACATAGCAGGGTACTCCAACAGACCAGCAGCTGAGGGTCCTGTCTGTTAGAAGGAAAACTAACAAACAGAAAGGATATCCACACCAAAAACCCATCTGTACATCACCATCATCAAAGACCAAAAGTAGATAAAACCACAAAGATGGGGAAAAAACAGAACAGAAAAACTGGAAACTCTAAAAAGCAGAGCGCCTCTCCTCCTCCAAAGGAACGCAGTTCCTCACCAGCAACGGAACAAAGCTGGACGGAAAACGACTTTGACGAGCTGAGAGAAGAAGGCTTCAGACGATCAAATTACTCTGAGCTACGGGAGTACATTCAAACCAAAGGCAAAGAAGTTGAAAACTTCGAAAAAAATTTAGAAGAATGTATAACTAGAATAACCAATACAGAGAAGTGCTTAAAGGAGCTGATGGAGCTGAAAACCAAGGCTCGAGAACTACGTGAAGAATGCAGAAGCCTCAGGAGCCGATGCAATCAACTGGAAGAAAGGGTATCAGCAATGGAAGATGAAATGAATGAAATGAAGCGAGAAGGGAAGTTTAGAGAAAAAAGAATAAAAAGAAATGAGCAAAGCCTCCAAGAAATATGGGACTATGTGAAAAGACCAAATCTACGTCTGATTGGTGTACGTGAAAGTGACGAGGAGAATGGAACCAAGTTGGAAAACACTCTGCAGGATATTATCCAGGAGAACTTCCCCAATCTAGCAAGGCAGGCCAACGTTCAGATTCCGGAAATACAGAGAATGCCACAAAGATACTTCTCGAGAAGAGCAACTCCAAGACACATAATTGTCAGATTCACCAAAGTTGAAATGAAGGAAAAAATGTTAAGGGCAGCCAGAGAGAAAGGTCGGGTTACCCTCAAAGGGAAGCCCATCAGACTAACAGCGGATCTCTCGGCAGAAACCCTACAAGCCAGAAGAGAGTGGGGGCCAATATTCAACATTCTTAAAGAAAAGAATTTTCAACCCAGAATTTCATATCCAGCCAAACTAAGCTTCATAAGTGAAGGAGAAATAAAATACTTTACAGACAAGCAAATGCTGAGAGATTTTGTCACCACCAGGCCTCCCCTAAAAGAGCTCCTGAAGGAAGCGCTAAACATGGAAAGGAACAACCGGTACCAGCCGCTGCAAAATCATGCCAAAATGTAAAGACCATCAAGACTAGGAAGAAACTGCATCAACTAACGAGCAAAATAGCCAGCTAACATCATCATGACAGGATCAAATTCACACATAACAATATTTACTTTAAATGTAAATGGACTAAATGCTCCAATTAAAAGACACAGACTGGCAAATTGGATAAAGAGTCAAGACCCATCAGTGTGCTGTATTCAGGAAACCCATCTCACGTGCAGAGACACACATAGGCTCAAAATAAAAGGATGGAGGAAGATCTACCAAGCAAATGGAAAACAAAAAAAGGCAGCGGTTGCAATCCTAGTCTCTGATAAAACAGACTTTAAACCAACAAAGATCAAAAGAGACAAAGAAGGCCATTACATGATGGTAAAGGGATCAATTCAATAAGAAGAGCTAACTATCCTAAATATATATGCACCCAATACAGGAGCACCCAGATTCATAAAGCAAGTCCTGAGTGACCTACAAAGAGACTTAGACTCCCACACATTAATAATGGGAGACTTTAACACCCCACTGTCAACATTAGACAGATCAACGAGACAGAAAGTCAACAAGGATACCCAGGAATTGAACTCAGCTCTGCACCAAGCAGACCTAATAGACATCTGCAGAACTCTCCACCCCAAATCAACAGAATATACATTCTTTCAGCACCACACCACACCCATTCCAAAATTGACCACATACTTGGAAGTAAAGCTCTCCTCAGCAAATGTAAAAGAACAGAAATTATAACAAACTATCTCTCAGACCACAGTGCAATCAAACTAGAACTCAGGATTAAGAATCTCACTCAAAACCGCTCAACTACATGGAAACTGAACAACCTGCTCCTGAATGACTACTGGGTACATAACGAAATGAAGGCAGAAATAAAGATGTTCTTTGAAACCAACGAGAACAAAGACACAACATACCAGAATCTCTGGGACACATTTAAAGCAGTGTGTAGAGGGAAATTTATAGCACTAAATGCCCACAAGAGAAAGCAGGAAAGATCCAAAATTGACACCCTAACATCACAATCAAAAGAACTAGAAAAGCAAGAGCAAACACATTGAAAAGCTAGCAGAAGGCAAGAAATAACTAAAATCAGAGCAGAACTGAAGGAAATAGAGACACAAAAAACCCTTCAAAAAATTAATGAATCCAGGAGCTGGTTTGTTGAAAGGATCAACAAAATAGATAGACCGCTAGCAAGACTAATAAAGAAAAAAAGAGAGAAGAATCAAAAAGACGCAATAAAAAATGATAAAGGGCATATCACCACTGATCCCACAGAAATACAAACTACCATCAGAGAATACTACAAACACCTCTACACAAATAAACTACAAAATCTAGAAGAAATGGATAAATTCCTCGACACATACACTCTCCCAAGACTAAACCAGGAAGAAGTTGAATCTCTGAATAGACCAATAACAGGATCTGAAATTGTGGCAATAATCAATAGCTTACCAACAAAAAAGAGTCCAGGACCAGATGGATTCACAGCTGAATTCTACCAGAGGTACAAGGAGGAACTGGTACCATTCCTTCTGAAACTATTCCAATCAATAGAAAAAGAGGGAATCCTCCCTAACTCATTTTATGAGGCCAGCATCATTCTGATACTAAAGCCTGGCAGAGACACAACCAAAAAAGAGAATTTTAGACCAATATCCTTGATGAACATTGATGCAAAAATCCTCAATAAAATACTGGCAAAACGAATCCAGCAGCACATCAAAAAGCTTATCCACCATGATCAAGTGGGCTTCATCCCTGGGATGCAAGGCTGGTTCAATATACACAAGTCAATAAATGTAATCCAGCATATAAACAGAACCAAAGACAAAAACCACATGATTATCTCAATAGATGCAGAAAGGGCCTTTGACAAAATTCAACAACGCTTCATGCTAAAAACTCTCAATAAATTAGGTATTGATGGGACGTATTTCAAAATAATAAGAGCTATCTATGACAAACCCACAGCCAATATCATACTGAATGGGCAAAAACTGGAAGCATTCCCTTTGACAACTGGCACAAGACAGGGATGCCCTCTCTCACCACTCCTATTCAACATAGTGTTGGAAGTTCTGGCCAGGGCAATTAGGCAGGAGAAGGAAATAAAGGGTATTCAATTAGGAAAAGAGGAAGTCAAATTGTCCCTCTTTGCAGATGACATGATTGTATAGCTAGAAAACCCCATTGTCTCAGCCCAAAATCTCGTTAAGCTGATAAGCAACTTCAGCAAAGTCTCAGGATACAAAATCAATGTGCAAAAATCACAAGCATTCTTATACACCAACAACAGACAAACAGAGAGCCAAATCATGAGTGAACTCCCATTCACAATTGCTTCAAGGAGAATAAAATACCTAGGAATCCAACTTACAAGGGATGTGAAGGACCTCTTCAAGGAGAACTACAAACCACTGCTCAAGGAAATAAAAGAGGATACAAACAAATGGAAGAACATTCCATGCTCATGGGTAGGAAGAATCAATATCATGAAAATGGCCATACTGCCCAAGGTAATTTACAGATTCAATGCCATCCCCATCAAGCTAGCAATGCCTTTCTTCACAGAATTGGAAAAAACTACTTTAAAGTTCATATGGAACCAAAAAAGAGCCCGCATTGCCAAGTCAATCCTAAGCCAAAAGAACAAAGCTGGAGGCATCACGCTACCTGACTTCAAACTATACTACAAGGCTACAGTAACCAAAACAGCATGGTACTGATACCAAAACAGAGATATAGATCAATGGAACAGAACAGAGCCCTCAGAAATAACGCCGCATATCTACAACTATCTCATCTTTGACAAACCTGAGAAAAACAAGCAATGGGGAAAGGATTCCCTGTTTAATAAATGGTGCTGGGAAAACTGGCTAGCCATATGTAGAAAGCTGAAACTGGATCCCTTCCTTACACATTATACAAAAATCAATTCAAGATGGATTAAAGACTTAAATGTTAGACCTAAAACCATAAAAACCCTAGAAGAAAACCTAGGCAATACCATTCAGGACATAGGCATGGGCAAGGACTTCATGTCTAAAGCACCAAAAGCAATGGCAACAAAAGACAAAATTGACAAATGGGATCTAATTAAACTAAAGAGCTTCTGCACAGCAAAAGAAACTACCATCAGAGTGAACAGGCAACCTACAAAATGGGAGAAAATTTTCGCAACCTACTCATCTGACAAAGGGCTAATATCCAGAATCTACAATTAACTCAAACAAATTTACAAGAAAAAAACAAACAACCCCATCAAAAAGTGGGCAAAGGACATGAACGGACACAGACACTTCTCAAAAGAAGACATTTATGCAGCCAAAAAACACATGAAAAAATGCTCACCATCACTGGCCATCAGAGAAATGCAAATCAAAACCACAGTGAGATACCATCTCACACTAATTAGAATGGCAATCATTAAAAAGTCAGGAAGCAACAGGTGCTAGAGAGGATGTGGAGAAATAGGAACACTTTTACACGGTTGGTGGGACTGTAAACTAGTTCAACCACTGTGGAAGTCCGTGTGGCGATTCCTCAGGGATCTAGAACTAGAAATACCATTTGACCCAGCCATCCTATTACTGGGTATATACCCAAAGGACTATAAATCATGCTGCTATAAAGACACATGCACACGTATGTTTATTGCAGCACTATTCACAATAGCAAAGACTTGGAACCAACCCAAATGTCCAACAATGATAGACTGGATTAAGAAAATGTGGCACATATATACCATGGAACACTATGCAGCCATAAAAAATGATGAGTTCATGTCCTTTGTAGGGACATGGATGAAATTGGAAATCATCATTCTCAGTAAACTATCGCAAGAACAAAAAATCAAACACTGCATATTCTCACTCATAGGTGGAAATTGAACAATGAGATCACATGGACACAGGAAGGGGAACATCACACTCTGGGGACTGTTGTGGGGTGGGGGGAGGGGGGAGGGATAGCATTGGGAGATATACCTAATGCTAGATGACGAGTTAGTGGGTGCAGCGCACCAGCATGGCACATGTATACATAAGTAACTAACCTGCACAATGTGCACATGTACCCTAAAACTTAAAGTATAAT*AATAAAAGAAAAAAA*TAT**ATATAGGAATC**AGAAATAAATATATGTATATATGAAAATGTTATCTATAGCAGAGGTAGCACTCCAGATTGGTGGGGAAAAGAC

**Clone 44; PA3; SpIRE(97/622)-44: Filled site spans: chr5:140515388-140521011**

Empty Site:

AATGACATCATTACTGCCTAAGTGGTTTTAACATTGGTCTGTGAAGAAATTTAGTAAAGCATTGGAAATCATTTGTTTATTGTTAAGATCTGGGTTACAAATGGTTTATTAGATTCCCAA

TTAAAGACTATCCAG**AAAAAAGAC**TATCCAGTATCCTAGG

ATGTGTGCTGTAATGGAAAAATTAAAACAAACAAACTAAAACATTGCCTTTCTGAGGCTCAGTCTGCTTACCTGGAAAATGACAGTGTTGATATACTGAGTTTGGAAATTATATTAAAAG

Filled Site:

TGTTAAGATCTGGGTTACAAATGGTTTATTAGATTCCCAATTAAAGACTATCCAG**AAAAAAGAC**GGGAGGAGCCAAGGTGGCCGAATAGGAGCAGCTCCGGTCTACAGCTCCCAGCGTGAGCGACGCAGAAGACGGGTGATTTCTGCATTTCCATCTGAGCTTTGAAGAGAGCAGTGGTTCTCCCAGCACGCAGCTGGAGATCTGAGAACGGGCAGACTGCCTCCTCAAGTGGGTCCCTGACCCCTGATCCCTGACCCCTGAGCAGCCTAACTGGGGGGCACCCCCCAGCAGGGTCAGACTGACACCTCACACGGCCGGGTACTTCAACAGACCTGCAGCTGAGGGTCCTGTCTGTTAGAAGGAAAACTAACAAACAGAAAGGACATCCACACCAAAAACCCATCTGTACATCACCATCATCAAAGACCAAAAGTAGATAAAACCAAAAAGATGGGGAAAAAACAGAGCAGAAAAACTGGAAACTCTAAAAAGCAGAGCGCCTCTCCTCCTCCAAAGGAACAAAGTTCCTCACCAGCAACGGAACAAAGCTGGACGGAGAATGACTTTGACGAGCTGAGAGAAGAAGGCCTCAGACGATCAAATTACTCTGAGCTACGGGAGGAAATTCAAACCAAAGGCAAAGAAGTTGAAAACTTTGAAAAAAATTTAGAAGATTGTATAACTAGAATAACCAATACAGAGAAGTGCTTAAAGGAGCTGATGGAGCTGAAAACCAAGGCTCGAGAACTACGTGAAGAATGCAGAAGCCTCAGGAGCCCATGCGATCAACTGGAAGAAAGGGTATCAGCGATGGAAAATGAAATGAATAAAATGAAGCGAGAAGGGAAGTTTAGACAAAAAAGAATAAAAAGAAACGAGCAAAGCCTCCAAGAAATATGGGACTATGTGAAAAGACCAAATCTACGTCTGATTGGTGTACCTGAAAGTGACAGGGAGAATGGAACCAAGTTGGAAAACACTGCAGGATATTATCCAGGAGAACTTCCCCAATCTAGCAAGGCAGGCCAACATTCCGATTCAGGAAATACAGAGAACGCCACAAAGATACTCCTCGAGAAGAGCGACTCCAAGACACATAATTGTCAGATTCACCAAAGTCGAAGGAAAAAATGTTAAGGGCAGCCAGCGAGAAAGGTCGGGTTACCCTCAAAGGGAAGCCCATCAGACTAACAGCAGATCTCTCGGCAGAAACTCTACAAGCCAGAAGAGAGTGGGGGCCAATATTCAACATTCTTAAAGAAAAGAATTTTCAACCCAGAATTTCATATCCAGCCAAACTAAGCTTTGTAAGTGAAGGAGAAATAAAATACTTTACAGAGAAGCAAATGCTGAGAGATTTTGTCACCACCAGGCCTGCCCTAAAAGAGCTCCTGAAGGAAGCACTAAACATGGAAAGGAACAACCGGTACCAGCCGCTGCAAAATCATGCCAAAATGTAAAGACCATCGAGACTAGGAAGAAACTGCATCAACTAACGAGCAAAATAAACAGCTAACATCATAATGACAGGATCAAATTCACACATAACAATATTAACTTTAAATGTAAACGGATTAAATGCTCCAATTAAAAGACACAGACTGGCAAATTGGATAAAGAGTCAAGACCCATCAGTGTGCTGTATTCAGGAAACCCATCTCACGTGCAGAGACACACATAGGCTCAAAATAAAAGGATGGAGGAAGATCTACCAAGCAAATGGAAAACAAAAAAAGGCAGGGGTTGCAATCCTAGCCTCTGATAAAACAGACTTTAAACCAACAAAGATCAAAAGAGACAAAGAAGGCCATTACATAATGGTAAAGGGATCAATTCAACAAGAAGAGCTAACTATCCTAAATATATATGCACCCAATACAGGAGCACCCAGATTCATAAAGCAAGTCCTGAGTGACCTACAAAGAGACTTAGACTCCCACACATTAATAATGGGAGACTTTAACACCCCACTGTCAACATTAGACAGATCAACGAGACAGAAAGTCAGCAAGGATACCCAGGAATTGAACTCAGCTCTGCACCAAGCGGACCTAATAGACATCTACAGAACCCTCCACCCCAAATCAACAGAATATACATTTTTTTCAGCACCACACCACACCTATTCCAAAATTGACCACATACTTGGAAGTAAAGCTCTCCTCAGCAAATGTAAAAGAACAGAAATTATAACAAACTATCTCTCAGACCACAGTGCAATCAAACTAGAACTCAGGATTAAGAATCTCACTCAAAACCGCTCAACTACATGGAAACTGAACAACCTGCTCCTGAATGACTACTGGGTACATAACGAAATGAAGGCAGAAATAAAGATGTTCTTTGAAACCAACGAGAACAAGGACACAACATACCAGAATCTCTGGGACACATTCAAAGCAGTGTGTAGAGGGAAATTTATAGCACTAAATGCCCACAAGAGAAAGCAGGAAAGATCCAAAATTGACACCCTAACATCACAATTAAAAGAACTAGAAAAGCAAGAGCAAACACATTCAAAAGCTAGCAGAAGGCAAGAAATAACTAAAATCAGAGCAGAACTGAAGGAAATAGAGACACAAAAAAACCCTTCAAAAAATTAACGAATCCAGGAGCTGGTTTTTTGAAAGGATCAACAAAATTGATAGACCACTAGCAAGACTAATAAAGAAAAAAAGAAGAATCAAATAGATGCAATAAAAAATGATAAAGGGGATATCACCACTGATCCTACAGAAATACAAACTACCGTCAGAGAATACTACAAACACCTCTACGCAAATAAACTAGAAAATCTAGAAGAAATGGATAAATTCCTCGACACATACACTCTCCCAAGACTAAACCAGGAAGAAGTTGAATCTCTGAATAGACCAAGAACAGGAGCTGAAATTGTGGCAATAATCAATAGCTTACCAACCAAAAAGAGTCCAAGACCAGATGGATTCACAGCCGAATTCTACCAGAGGTACAAGGAGGAGCTGGTACCATTCCTTCTGAAACTATTCCAATCAATAGAAAAAGAGGGAATCCTCCCTAACTCATTTTATGAGGCCAGCATCATCCTGATACCAAAGCCGGGCAGAGACACAACCAAATAAGAGAATTTTAGACCAATATCCTTGATGAACATTGATGCAAAAATCCTCAATAAAATACTGGCAAACCGAATCCAGCAGCACATCAAAAAGCTTATCCACCATGATCAAGTGGGCTTCATCCCTGGGATGCAAGGCTGGTTCAGTATACGCAAATCAATAAAAGTAATCCAGCATATAAACAGAACCAAAGACAAAACCACATGATTATCTCAATAGATGCAGAAAAGGCCTTTGACAAAATTCAACAACCCTTCATGCTAAAAACTCAATAAATTAGGTATTGATGGGACGTATCTCAAAATAATAAGAGCTATCTATGACAACCCCACAGCCAATATCATACTGAATGGGCAAAAACTGGAAGCGTTCCCTTTGAAAACGGGCACAAGACAGGGATGCCCTCTCTCACCACTCTTATTCAACATAGTGTTGGAAGTTCTGGCCAGGGCAAATAGGCAGGAGAAGGAAATAAAGGGTATTCAATTAGGAAAAGAGGAAGTCAAATTGTCCCTGTTTGCAGACGACATGATTGTATATCTAGAAAACCCCATTGTCTCAGCCCAAAATCTCCTTAAGCTGATAAGCAACTTCAGCAAAGTTTCAGGATACAAAATCAATGTACAAAAATCACAAGCATTCTTATACACCAACAACAGAGAGCCAAATCATGAGTGAACTCCCATTCACAATTGCTTCAAAGAGAATAAAATACCTAGGAATCCAACTTACAAGGGATGTAAAGGACCTCTTCAAGGAGAACTACAAACCACTGCTCAGTGAAATAAAAGAGGATACAAACAAATGGAAGAACATTCCATGCTCATGAGTAGGAAGAATCAGTATCGTGAAAATGGCCATACTGCCCAAGGTAATTTACCGATTCAATGCCATCCCCATCAAGCTACCAATGACTTTCTTCACAGAATTGGAATAACTACTTTAAAGTTCATATGGCACCAAAAAAGAGCCCACATCGCCAAGTCAGTCCTACGCCAAAAGAACAAAGCTGGAGGCATCACACTACCTGACTTCCAACTATACTACAAGGCTACAGTAACCAAAACAGAATGTTACTGGTACCAAAACAGAGATATAGATCAATGGAACAGAACAGAGCCCTCAGAAATAACGCCGCATGTCTACAACTATCTGATCTTTGACAAACCTGAGAAAAACAAGCAATGGGGAAAGGATTCCCTATTTAATACATGGTGCTGGGAAAACTGGCTAGCCATATGTAGAAAGCTGAAACTGGATCCCTTCCTTACACCTTATACAAAAATCAGTTCAAGATGGATTAAAGACTTAAACGTTAGACCTAAAACCATAAAAACCCTAGAAGAAAACCTAGGCATTACCATTCAGGACATAGGCATGGGCAAGGACCTCATGTCTAAAACACCAAAAGCAATGGCAACAAAAGCCAAAATTGACAAATGGGATCTAATTAAACTAAAGAGCTTCTGCACAGCAAAAGAAACTACTATCAGAGTGAACAGGCAACCTACAAAATGGGAGAAAATTTTCGCAACCTACTCATCTGACAAAGGGCTAATATCCAGAATCTACAATGAACTCAAACAAATTTACAAGAAAAAAACAAACAACCCCATCAAAAAGTGGGCAAAGGACATGAACAGACACTTCTCAAAAGAAGACATTTATGCAGCCAAAAAACACATGAAAAAATGCTCCTCATCACTGGCCATCAGAGAAACGCAAATCAAAACCACAATGAGATACCATCTCACACCAGTTAGAATGGCGATCATTAAAAAGTCAGGAAACAACAGGTGCTGGAGAGGATGTGGAGAAATAGGAACACTTTTACACTGTTGGTGGGACTCTAAACTAGTTCAACCATTGTGGAAGTCAGTGTGGCGATTCCTCAGGGATCTAGAACTAGAAATACCATTTGACCCAGCCATCCTATTACTGGGTATATACCCAAAGGACTATAAATCATGCTACTATAAAGACACATGCACACGTATGTTTATTGCGGCATTATTCACAATAGCAAAGACTTGGAACCAACCCAAATGTCCAGCAATGATAGACTGGATTAAGAAAATGTGGCACATATACACCATGGAATACTATGCAGCCATAAAAAATGATGAGTTCATGTCCTTTGTGGGGACATGGATGAAATTGGAAATCATCATTCTCAGTAAACTATTGCAAGAACAAAAAACCAAACACCGCATATTCTCACTCATAGGTGGGAATTGAACAATGAGAACACATGGACACAGGAAGGGGAACATCACACTCTGGGGACTGTTGTGGGGTGGGGGGGAGGGGGGAGGGATAGCATTGGGAGATATACCTAATGCTAGATGACGAGTTAATGGGTGCAGCGCACCAGCATGGCACATGTATACATATGTAACTAACTGCACATTGTGCACATGTACCCTAAAACTTAAAGTATAAT*AAAAAAAAAA****AAAAAA*GAC**TATCCAGTATCCTAGGATGTGTGCTGTAATGGAAAAATTAAAACAAACAAACTAAAACATTGC

**Clone 45; PA3; SpIRE(97/622)-45: Filled site spans: chr5:8158993-8165702**

Empty Site:

TAGTCTTTGAAAACTTATTGCCTAGAAATACAGGCCTTCTTCATTTTCCCTTTCTGTGTGCTACACTCGCATGCACACACATGCGTAAGAAATACAGCTGCACACATCAGTATGAACCAC

CCTGTTAAGAAGCAGG**TTAAGAACCATGGC**TGGGGAGCTA

GTGTGGTGATTTGGAGGCAAGAAGACACAGTGACTTTCTGAGTTGCCAGAGTTTCTGCACTAGTTCTTTCTCATCTGTGTGGGTGACTGTTCCTTTCATCTTTGGAGTTACTGTCCTTTG

Filled Site:

ATGCGTAAGAAATACAGCTGCACACATCAGTATGAACCACCCTGTTAAGAAGCAGG**TTAAGAAGTCTGGC**GGGGAGGAGCCAAGATGGCTGAATAGGAACAGCTCTGGTCTACAGCTCCCAGCGTGAGCGAAACAGAAGACGGGTGATTTCTGCATTTCCATCTGAGCTTTGAAGAGAGCAGTGGTTCTCCCAGCACGCAGCTGGAGATCTGAGAACGGGCAGACTGCCTCCTCAAGTGGGTCCCTGACCCCTGACCCCCGAGCAGCCTAACTGGGAGGCACCCCCCAGCAGGGGCAGACTGACACCTCACACGGCAGGGTACTCCAACAGACCTGCAGCTGAGGGTCCTGTCTGTTAGAAGGAAAACTAACAAACAGAAAGGACATCCACACCAAAAACCCATCTGTACATCACCATCATCAAAACCAAAAGTATATAAAACCACAAAGATGGGGAAAAAACAGAGCAGAAAACTGGAAACTCTAAAAAGCAGAGCGACTCTCCTCCTCCAAAGGACCGCAGTTCCTCACCAGCAACGGAACAAAGCTGGATGGAGAATGACTTTGACGAGCTGAGAGAAGAAGGCTTCAGACAATCAAATTACTCCGAGCTCCGGGAGGACATTCAAACCAAAGGCAAAGAAGTTGAAAACTTTGAAAAAAATTTAGATGAATGTATAACTAGAATAACCAATACAGAGAAGTGCTTAAAGGAGCTGATTGAGCTGAAAACCAAGGCTCGAGAACTACGTGAAGAATGCAGAAGCCTCAGGAGCCGATGCGATCAACTGGCAGAAAGGGTATCAGCGATGGAAGATGAAATGAATGAAATGAAGCGAGAAGGGAAGTTTAGAGAAAAAAGAATAAAAAGAAATGAGCAAAGCCTCCAAGAAATATGGGACTATGTGAAAAGACCAAATCTACGTCTGATTGGTGTACCTGAAAGTGACGGGGAGAATGGAACCAAGTTGGAAAATACTCTGCAGGATATTATCCAGGAGAACCTCCCCAGTCTAGCAAGGCAGGCCAACATTCAGATTCAGGAAATACAGAGAACACCACAAAGATACTCCTCGAGAAGAGCAACTCCAAGACACATAATTGTCAGATTCACCAAAGTTGAAATGAAGGAAAAAATGTTAAGGGCAGCCAGAGAGAAAAGTTGGGTTACCCTCAAAGGGAAGCCCATCAGACTAACAGCAGATCTATCAGCAGAAACTCTACAAGCCAGAAGAGAGTGGGGGCCAATATTCAACATTCTTAAAGAAAAGAATTTTCAACCCAGAATTTCATATCCAGCCAAACTAAGCTTCATAAGTGAAGGAGAAATAAAATACTTTTCAGACAAGCAAATCCTGAGAGATTTTGTCACCACCAGGCCTGCCCTAAAAGAGCTCCTGAAGGAAGCGCTAAACATGGAAAGGAACAACCGGTACCAGCCACTGCAAAATCATGCCAAAATGTAAAGACCATCGAGACTAGGAAGAAACTGCATCAACTAACGAGCAAAATAACCAGCTAACATCATAATGACAGGATCAAATTCACACATAACAATATTAACTTTAAATGTAAAGGGACTAAATGCTCCAATTAAAAGACACAGACTGGCAAATTGGATAAAGAGTCAAGACCCATCAGTGTGCTGTATTCAGGAAACCCATCTCATGTGCAGAGACACACACAGGCTCAAAATAAAAGGATGGAGGAAGATCTACCAAGCAAATGGAAAACAAAAAAAGGCAGGGGTTGCAATCCTAGTCTCTGATAAAACAGACTTTAAGCCAACAAAGATCAAAAGAGACAAAGAAGGCCATTACATAATGGTAAAGGGATCAATTCAACAAGAAGAGCTAACTATCCTAAATATATATGCACCCAATACAGGAGCACCCAGATTCATAAAGCAAGTCCTGAGTGACCTACAAAGAGACTTAGAGTCCCACACATTAATAATGGGAGACTTTAACACCCTACTGTCAACATTGGACAGATCAACGAGACAGAAAGTCAACAAGGATATCCAGGAATTGAAATCAGCTCTGCAACAAGCGGACCTAATAGACATCTACAGAGCTCTCCACCCCAAATCAACAGAATATACATTTTTTTCAGCACCACACCACACCTATTCCAAAAGTGACCACATACTTGGAAGTAAAGCTCTCCTCAGCAAATGTAAAAGAACAGAAATTATAACAAACTATCTCTCAGACCACAGTGCAATCAAACTAGAACTCAGGATTAAGAAACTCACGCAATACCGCTCAACTACATGGAAACTGAACAACCTGCTCCTGAATGACTACTGGGTACATAACGAAATGAAGGCAGAAATAAAGATCTTCTTTGAAACCAAGGAGGACAAAGACACAACATACCAGAATCTCTGGGACACATTCAAAGCAGTGTGTAGAGGGAAATCTATAGCACTAAATGCCCACAAGAGAAAGCAGGAAAGATCCAAAATTGACACCCTAACATCACAATTAAAGGAACTAGAAAAGCAAGAGCAAACACATTCAAAAGCTAGCAGAAGTCAAGAAATAACTAAAATCAGAGCAGAACTGAAGGAAATAGAGACACAAAAAACCCTTCAAAAAATTAATGAATCCAGGAGCTGGTTTTTTGAAAGGATCAACGAAATTGATAGACCGCTAGCAAGACTAATAAAGAAAAAAAGAGAGAAGAATCAAATAGACGCAATAAAAAATGACAAAGGGGATATCACCACCGATCCCACAGAAATACAAACTACCATCAGAGAATACTACAAACACCTCTACGCAAATAAACTAGAAAATCTAGAAGATATGGATAAATTCCTTGACACATACACTCTCCCAAGACTAAACCAGGAAGAAGTTGAATCTCTGAATAGACCAATAACAGGATTTGAAATTGTGGCAATAATCAATAGCTTACCAACCAAAAAGAGTCCAGGACCAGATGGATTCACAGCCGAATTCTACCAGAGGTACAGGGAGGAACTGGTACCATTCCTTCTGAAACTATTCCAGTCAATAGAAAAAGAGGGAATCCTCCCTAACTCATTTTATGAGGCCAACATCATTCTGATACCAAAGCCAGGCAGAGACACAACCAAAAAAGAGAATTTTAGACCAATATCCTTGATGAACATTGATGCAAAAATCCTCAATAAAATACTGGCAAACCGAATCCAGAAGCACATCAAAAAGCTTATCCACCATGATCAAGTGGGCTTCATCCCAGGGATGCAAGGCTGGTTCAATATACGCAAATCAATAAATGTAATCCAGCATATAAACAGAGACAAAGACAAAAACCACATGATTATCTCAATAGATGCAGAAAAGGCCTTTGACAAAATTCAACAACCCTTCATGCTAAAAACTCTCAATAAATTAGGTATTGATGGGACATATTTCAAAATGATAAGAGCTATCTATGACAAACCCACAGCCAATATCATACTGAATGGGCAAAAACTGGAAGCATTCCCTTTGAAAACTGGCACAAGATAGGGATGACCTCTCTCACCACTCCTATTCAACATAGTGTTGGAAGTTCTGGCCAGGGCAATTAGGCAGGAGAAGGAAATAAAGGGTATTCAAATAGGAAAAGAGGAAGTCAAATTGTCCCTGTTTGCAGATCACATGATTGTATATCTAGAAAACCCCATTGTCTCAGCCCAAAATCTCCTTAAGCTGATAAGCAACTTCAGCAAAGTCGCAGGATACAAAATCAATGTGCAAAAATCACAAGCATTCCTATACACCAACAACAGACAAACAGAGAGCCAAATCATGAGTGAACTCCCATTCACAATTGCTTCAAAGAGAATAAAATACCTAGGAATCCAACTTACAAGGGATGTGAATGACCTCTTCAAGGAGAACTACAAACCACTGCTCAAGGAAATAAAAGAGGATACAAACAAATGGAAGAACATTCCATGCTCATGGGTAGGAAGAATCAATATCGTGAAAATGGCCATACTGCCCAAGGTAATTTACAGATTCAATGCCATCCCCATCAAGCTACCAATGCCTTTCTACACAGAATTGGAAAAAACTACTTTAAAGTTCATATGGAACCAAAAAAGAGCCCACATCACCAAGTCAATCCTAAGCCAAAAGAACAAAGCTGGAGGCATCATGCTACCTGACTTCAAACTATACTACAAGGCTACAGTAACTAAAACAGCATGGTACTGGTACCAAAACAGAGATATAGATCAATGGAACAGAACAGAGCCCTCAGAAACAATGCCGCATATCTCCAACTATCTGATCTTTGACAAACCTGAGAAAAACAAGCAATGGGGAAAGGATTCCCTATTTAATAAATGGTGCTGGGAAAACTGGCTAGCCATATGTAGAAAGCTGAAACTGGATCCCTTCCTTACACCTTATACAAAAATCAATTAAAGATGGATTAAAGGCTTACATGTTAGACCTAAAACCATAAAAACCCTAGAAGAAAACCTAGGCATTACCATTCAGGACATAGGCATGGGCAAGGACTTCATGTCTAAAACACCAAAAGCAACGGCAACAAAAGCCAAAATTGACAAATGGGATCTAATTAAACTAAAGAGCTTCTGCACAGCAAAAGAAACTACCATCAGAGTGAACAGGCAACCTACAAAGTGGGAGAAAATTTTCGCAACCTACTCATCTGACAAAGGGCTAATATCCAGAATCTACAATGAACTCAAACAAATTTACAGGAAAAAAACAAACAACCCCATCAAAAAGTGGGCAAAGGACATGAACAGACATTTCTCAAAAGAAGACATTTATGCAGCCAAAAAACACATGAAAAAATGCTCACCACCACTGGCCATCAGAGAAATGCAAATCAAAACCACAATGAGATACCATCTCACACCAGTTAGAATGGCAATCATTAAAAAGTCAGGAAACAACAGGTGCTGGAGAGGATGTGGAGAAATAGGAACACTTTTACACTGTTGGTGGGACTGTAAACTAGTTCAACCATTGTGGAAGTCAGTGTGGCGATTCCTCAGGGATCTAGAACTAGAAATACCATTTGACCCAGCCATCCCATTACTGGGTATATACCCAAAGGACTATAAATCATGCTGCTATAAAGACACATGCACACGTATATTTATTGCGGCACTATTCACAATAGCAAAGACTTGGAACCAACCCAAATGTCCAACAATGATAGACTGGATTAAGAAAATGTGGCACATATACACCATGGAATACTATGCAGCCATAAAAAAATGATGAGTTCATGTCCTTTGTAGGGACATGGATGAAATTGGAAACCATCATTCTCAGTAAACTATTGCAAGAACAAAAAACCAAACACCACATATTCTCACTCATAGGTGGGAATTGAACAATGAGATCACATGGACACAGGAAGGGGAACATCACACTCTGGGGACTGTTGTGGGGTGGTGGGAGGGGGGAGGGATAACATTGGGAGATATACCTAATGCTAGATGATGAATTAGTGGGTGCAGCACACCAGCATGGCACATGTATACGTATGTAACTAACCTGCACAATGGGCACATGTACCCTGAAACTTAAAGTATAA*TAATAAATAAATAAATAAATAAATAAAAAATTAAAAAGAAA*CTCTTGGATATTTATTTGACATGTAAATGTTTGAATTGGAAACTACAAATTAATACTGATATGTGTGTTTGATTATGTCATCATGTCATTAGCTGGTTACTATGCAGAGTTGATTGTGCAGTTGTTTTATAATATCAATGCTTTATGTACTTCAGTGTTTTTGTGGTAACTGGTAATGGTCTTTCCTTTCTATATTTAGCACTCCCTTAAGGACTTCTTGTAAGACAGGTCTAGTGATAATTAACTCTCTTAGCATTTGCTTTTCTGAAAAGGATCTTATTTCTCCTTCACCTGTGAAGATTACTTTGACTGGATATGAAATTCTTGGTTGGAATTTCTTTTCTTCAAGAATGCTGGATATAGGCCGTTAATCTCTTCTGGTTAGTAGAGTTTCTGCTGAAAGATCCATCATTAACCTGATGAGGTTCCCTTTGTAGGTGACCTTTTCTCTGTAGCTGCCTTTAACATTTTTTCTTTCATTTCAACCTTGGAAGATCTGAAGACTATGTGTTTTGGAGATGGTCATCATGTATAGTATTATGCAAGAATTTTCTGCATTTCCTGAATTTGAATGTTGGCCTCTCACAGGCTTGGGACAATTTTCATGGATGAAATCCTGAAATATGTTTTTTGCTTACTTTCTCCCCATCTCTTTCAAAGAGTTGTAGATTTGGTCTCTTTACATGATCCCATGTTTCTTGGAGGTTTTATTCATTATTTTTTATTTGTTTTCCTTTTTTCTCCATCTGATTGAGTTATTTCAAAGACCATGTCTTCAAACTCTGAGATTCTTTCCTCAGTTTGGTCAATTCTGCTGTCATTACCTGTGATTATACTATAAAATTCTTGAAGTGAATTTTTCAGCTCAGTCAGATCACTTTGATTCTTTCTTAAAATGGCCATTTCATCTTTTACCTCCTGTGTCATTTTACTGTATTCTTTAGATTGGGTTTCAACCTTCTCTTGAACGTTGATGATCTTTGTTTCTATCCATATTCTGAATTCTACTTCTGACATTTCAGCCATTTCAACCTGT**TTAAGAACCATGGC**TGGGGAGCTAGTGTGGTGATTTGGAGGCAAGAAGACACAGTGACTTTCTGAGTTGCC

**Clone 46; PA3; SpIRE(97/622)-46: Filled site spans: chr5:148610121-148615827**

Empty Site:

TCATTGGAAGCCAGGGCATTTCAGAACAATTCTAATATGATGTTGTCCTTCCAATAAAACATTGCTCATCACAACTATCTGAGAAAAAGAGTGAGTGGAGGCCTTCTTCAGCCCTCATAG

TCTTGTGGATAG**AAAGCTCCTAAAGGT**ATGTTAAGTGGTT

AAGTTTCTGGATCCTCTTCTTGCAGACTGCTCAGGTAAAATGCTGATTCAGGACTTTCACTTGTCAATCAATTTCTCCAGCGGGAGCCAAAATACTTTTTTGAAAAATGTTGTCCTGTCT

Filled Site:

GAGAAAAAGAGTGAGTGGAGGCCTTCTTCAGCCCTCATAGTCTTGTGGATAG**AAAGCTCCTAAAGGT**GGTGGAGGAGCCAAGATGGCCAAATAGGAACAGCTCCGGTCTACAGCTCCCAGCCTGAGCGACACAGAAGATGCGTGATTTCTGCATTTCCATCTGAGCTTTGAAGAGAGCAGTGGTTCTCCCAGTACGCAGCTGGAGATCTGAGAACGGGCAGACTGCCTCCTCAAGTGGGTCCCTGACCCGTGACCCCCGAGCAGCCTAACTGGGAGGCACCCTCCAGCAGGGGCACACTGACACCTCACACTGCAGGGTACTCCAACAGACCTGCAGCTGAGGGTCCTGTCTGCTACAAGGAAAACTAACAAACAGAAAGGACATCCACACCAAAAACCCATCTGTACATCACCATCATCAAAGACCAAAAGTAGATAAAACCACAAAGATGGGGAAAAAACAGAACAGAAAAACTGGAAACTCTAAAAATCAGAGTGCCTCTCCTCCTCCAAAGGAACGCAGCTCCTCACCAGCAACGGAACAAAGCTGGACGGAGAATGACTTTGATGAGCTGAGAGAAGAAGGCTTTAGACGATCAAATTACTCTGAGCTACGGGAGGACATTCAAACCAAAGGCAAAGAAGTTGAAAACTTTGAAAAAAATTTAGAAGAATGTATAACTAGAATAACCAATACAGAGAAGTGCTTAAAGGAGCTGATGGAGCTGAAAACCAAGGCTCCAGAACTATGTGAAGAATGCAGAAGCCTCAGGAGCCGATGCGATCAACTGGAAGAAAGGGTATCAGCAATGGAAGATGAAATGAATGAAATGAAGCAAGAAGGAAAGTTTAGAGAAAAAAGAATAAAAAGAAATGAGCAAAGCCTCCAAGAAATATGGGACTATGTGAAAAGACCAAATCTACGTCTGATTGGTGTACCTGAAAGTGATGGGGAGAATGGAACCAAGTTGGAAAACACTCTGCAGGATATTATCCAGGAGAATTTCCCCAATCTAGCAAGGCAGGCCAACGTTCAGATTCAGGAAATACAGAGAACGCCACAAAGATACTCCTCGAGAAGAGCAACTCCAAGACACATAATTGTCAGATTCACCAAAGTTGAAATGAAGGAAAAAATGTTAAGGGCAGCCAGAGAGAAAGGTCGGGTTACTCTCAAAGGGAAGCCCATCAGACTAACAGCGGATCTGTCGGCAGAAACTCTACAAGCCAGAAGAGAGTGGGGGCCAATATTCAACATTCTTAAAGAAAAGAATTTTCAACCCAGAATTTCATATCCAGCCAAACTAAGCTTCATAAGTGAAGGAGAAATAAAATACTTTACAGACAAGCAAATGCTGAGAGATTTTGTCACCACCAGGCCTGCCCTAAAAGAGCTCCTGAAGGAAGCGCTAAACATGTAAAGGAACAACCGGTACCAGCCACTGCAAAATCATGCCAAAATGTAAAGACCATCGAGACTAGGAAGAAACTGCATCAACTAACGAGCAAAATCACCAGCTAACATCATAATGACAGGATCAAATTCACACATAACACTATTAACTTTAAATGTAAATGGACTAAATGCTCCAATTAAAAGACACAGACTGGCAAATTGGGTAAAGAGTCAAGACCCATCAGTGTGCTGTATTCAGGAAACCCATCTCACGTGCAGAGACACACATAGGCTCAAAATAAAAGGATGGAGGAAGATCTACCAAGCAAATGGAAAACAAAAAAAGGCAGGGGTTGCAATCCTAGTCTCAGATAAAACAGACTTTAAACCAACAAAGATCAAAAGAGACAAAGAAGGCCATTACATAATGGTAAAGGGATCAATTCAACAAGAAGAGCTAACTATTCTAAATATATATGCACCCAATACAGGAGCACCCAGATTCATAAAGCAAGTCCTGAGTGACCTACAAAGAGACTTAGACTCCCACGCATTAATAATGGGAGAATTTAACACCCCACTGTCAACATTAGACAGATCAACGAGACAGAAAGTCAACAAGGATACCCAGGAATTAAACTCAGCTCTGCACCAAGCGGACCTAATAGACATCTACAGAACTCTCCACCACAAATCAACAGAATATACATTTTTTTCAGCACCACACCACACCTATTCCAAAATTGACCACATACTTGGAAGTAAAGCTCTCCTCAGCAAATGTAAAAGAACAGAAATTATAACAAACTGTCTCTCAGACCACAGTGCAATCAAACTAGAACTCAGGATTAAGAATCTCACTCAAAACCACTCAACTACATGGAAACTGAACAACCTGCTCCTGAATGACTACTGGGTACATAACGAAATGAAGGCAGAAATAAAGATGTTCTTTGAAACCAACAAGAACAAAGACACAACATACCAGAATCTCTGGGACGCATTCAAAGCAGTGTGTAGAGGGAAATTTATAGCACTAAATGCCCACAAGAGAAAGCAGGAAAGATCCAAAATTGACACCCTAACATCACAATTAAAAGAACTAGAAAAGCAAGAGCAAACACATTCAAAAGCTAGCAGAAGGCAAGAAATAACTAAAATCAGAGCAGAACTGAAGGAAATAGAGACACAAAAAAACCCTTCAAAAAATTAATGAATCCAGGAGCTGGTTTTTTGAAAGGATCAACAAAATTGATAGACCGCTAGCAAGACTAATAAAGAAAAAAAGAGAGAAGAATCAAATAGACGCAATAAAAAATGATAAAGGGGATATCACCACCGATCCCACAGAAATACAAACTACCATCAGAGAATACTACAAACACCTCTACGCAAATAAACGAGAAAATCTAGAAGAAATGGATAAATTCCTTGACACATACACTCTCCCAAGACTAAACCAGGAAGAAGTTGAATCTCTGAATAGACCAATAACAGGATCTGAAATTGTGGCAATAATCAATAGCTTACCAACCAAAAAGAGTCCAGGACCAGATGGATTCACAGCCGAATTCTACCAGAGGTACAAGGAGGAACTGGTACCATTCCTTCTGAAACTATTCCAATCAATAGAAAAAGAAGGAATCCTCCCTAACTCATTTTATGAGGCCAGCATCATTCTGATATCAAAGCCGGGCAGAGACACAACCAAAAAAGAGAATTTTAGACCAATATCCTTGATGAACATTGATGCAAAAATCCTCAATAAAATACTGGCAAACCAAATCCAGCAGCACATCAAAAAGCTTATCCACCATGATCAAGTGGGCTTCATCCCTGGGATGCAAGGCTGGTTCAATATACGCAAATTAATAAATGTAATCCAGCATATAAACAGAGCCAAAGACAAAAACCACATGATTATCTCAATAGATGCAGGAAAGGCCTTTGACAAAATTCAACAACCCTTCATGCTAAAAACTCTCAATAAATTAGGTATTGATGGGATGTATCTCAAAATAATAAGAGCTATCTATGACAAACCCACAGCCAATATCATACTGAATGGGCAAAAACTGGAAGCATTCCCTTTGAAAACTGGCACAAGACAGGGTTGCCCTCTCTCACCACTCCTATTCAACATAGTGTTGGAAGTTCTGGCCAGGGCAATTAGGCAGGAGAAGGAAATAAAGGGTATTCAATTAGGAAAAGAGGAAGTCAAATTGTCCCTGTTTGCAGATGACATGATTGTATATCTAGAAAACCCCATTGTCTCAGCCCAAAATCTCCTTAAGCTGATAAGCAACTTCAGCAAAGTCTCAGGATACAAAATCAATGTGCAAAAATCACAAGCATTCCTATATACCAACAACAGACAAACAGAGAGCCAAACCATGAGTGAACTCCCATTCACAATTGCTTCAAAGAGAATAAAATACCTAGGAATCCAACTTACAAGGGATGTGAAGGACCTCTTCAAGGAGAACTACAAACCGCTGCTCAAGGAAATAAAAGAGGATACAAACAAATGGAAGAACATTCTATGCTCATGGGTAGGAAGAATCAATATCGTGAAAATGGCCATACTGCCCAAGGTAATTTACAGATTCAATGCCATCCCCATCAAGCTACCAATGCCTTTCTTCACAGAATTGGAAAAAACTACTTTCAAGTTCATATGGAACCAAAAAAGAGCCTGCATTGCCAAGTCAATCCTAAGCCAAAAGAACAAAGCTGGAGGCATCACACTACCTGACTTCAAACTATACTACAAGGCTACAGTAACCAAAACAGCATGGTACTGGTACCAAAACAGAGATATAGATCAATGGAACAGAACAGAGCCCTCAGAAATAATGCCGCATATCTACAACTATCTGATCTTTGACAAACTTGAGAAAAACAAGCAATGGGGAAGGATTCCCTATTTAATAAATGGTGCTGGGAAAACTGGCTAGCCATATGTAGAAAGCTGAAACTGGATCCCTTCCTTACACCTTATACAAAAATTAATTCAAGGTGGATTAAAGACTTAAACGTTAGACCTAAAACCATAAAAACCCTAGAAGAAAACCTAGGCATTACCATTCAGGACATAGGCATGTGCAAGGACTTCATGTCTAAGACACCAAAAGCAATGGCAACAAAAGACAAAATTGACAAATGGGATCTAATTAAACTAAAGAGCTTCTGCACAGCAAAAGAAACTACCATCAGAGTGAACAGGCAACCTACAAAATGGGAGAAAATTTTCACAACCTACTCATCTGACAAAGGGCTAATATCCAGAATCTACAATGAATTCAAACAAATTTACAAGAAAAAGCAAACAACCCCATCAAAAAGTGGGCAAAGGACATGAACAGACACTTCTCAAAAGAAGACATTTATGTAGCCAAAAAACACATGAAAAAATGCTCACCATCACTGGCCATCAGAGAAATGCAAATCAAAACCACAATGAGATACCATCTCACACCAGTTAGAATGGCAATCATTAAAAGTCAGGAAACAACAGGTGCTGGAGAGGATGTGGAGAAATAGGAACACTTTTACACTGTTGGTGGGACTGTAAACTAGTTCAACCATTGTGGAAGTCAGTGTGGCGATTCCTCAGGGATCTAGAACTAGAAATACCATTTGACCCAGCCATCCCATTACTGGGTATATACCCAAAGGACTATAAATCATGTTGCTATAAAGACACATGCACACGTATGTTTATTGCAGCATTATTCACAATAGCAAAGACTTGGAACCAACCCAAATGTCCAACATTGATAGACTGGATTAAGCAAATGTGGCACATATACACCATGGAATACTATGCAGCCATAAAAAATGATGAGTTCATGTCCTTTGTAGGGACATGGATGAAATTGGAAATCATCATTCTCAGTAAACTATCACAAGAACAAAAAACCAAACACTGCATATTCTCACTCATAGGTGGGAATTGAACAATGAGATCACATGGACACAGGAAGGGGAACATCACACTCTGGGGACTGTTGTGGGGTGGGGGGAGGGGGGAGGGATAGCATTGGGAGATATACCTAATGCTAGATGACGAGTTAGTGGGTGTAGCACACCAGCATGGCACATGTATACGTATGTAACTAACCTGCACAATGTGCACATGTACCCTAAAACTTAAAGTATAA*TAATTAAAAAAATAAAGAAAGAAAGAAAGAAATAAAATAAAATAAAATAAAATAAAATAAAAT****AAA*GCTCCTAAAGGT**ATGTTAAGTGGTTAAGTTTCTGGATCCTCTTCTTGCAGACTGCTCAGGTAAAATGCTGATTCAGGACTTT

**Clone 47; PA3; SpIRE(97/622)-47: Filled site spans: chr5:109621602-109627306**

Empty Site:

GATACTTCAACCAGGGTACAATCTCCCAGGTTTAAACTGCAAAGGACAAGGAAAAGGGCCTACAATTCCTGAGGCTGTAGGTCTCCCTCTTCTTGAGGCTTCACTGTGAAGTAA

CATTGT

TTGTCCTAAATCTAAAAGAA**AAAAAAAAG**AAAAAGTATAAAGTATCC

AAAACCCATCTCTGCTAAAAATACAAAAAATTAGCCAGGCGTGGTGGTGAGCACCTGTAAATCCAGCTACTCAGGGGGCTGAGGCAGGAGAATCACTTGAACCTGGAGGGAGAGGTTGGA

Filled Site:

GTCTCCCTCTTCTTGAGGCTTCACTGTGAAGTAACATTGTTTGTCCTAAATCTAAAAGAA**AAAAAAAAG**GAGGGAGGAGCCAAGATGGCTGAATAGGAACAACTCCGGTCTACAGCTCCCAGTGTGAGCGACGCAGAAGATGGGTGATTTCTGCATTTCCATCTGAGCTTTGAAGGGAGCAGTGGTTCTCCCAGCACGCAGCTGGAGATCTGAGAACGGGCAGACTGCCTCCTCAAGTGGGTCCCTGACCGCTGACCCCCGAACAGCCTAACTGGGAGGCACCCCCCAGTGGGGGCAGACTGATGTCTCACATGGCCGGGTACTCCTCTGAGACAAAACTTCCAGAGGAACAATCAGACAGCAGCATTCGCGGTTCATGAAAAACCGCTGTTCTGCAGACACCGCTGCTGATACCCAGGCAAACAGGGTCTGGAGTGGACCTCTAGCAAACTCCAACAGACCTGCAGCTGAGGGTCCTGTCTGTTAGAAGGAAAACTAACAAACAGAAAGGACATCCACACCAAAAGCCCATCTGTACATCACCATCATCAAACACCAAAAGTAGATAAAACCACAAAGATGGGGAAAAAACAGAACAGAAAAACTGGAAACTCTAAAAAGCAGAGCGCCTCTCCTCCTCCAAAGGAACGCAGTTCCTCACCAGCAACGGAACAAAGCTGGATGGAGAATGACTTTGATGAGCTGAGAGAAGAAGGCTTCAGATGATCAAACTACTCCGAGCTATAGGAGGAAATTCAAACCAAAGGCAAAGAAGTTGAAAACTTTGAAAAAAATTTAGAAGAATGTATAACTAGAATAACCAAGACAGAGAAGTGCTTAAAGGAGCTGATGGAGCTGAAAGCCAAGGCTTGAGAAATACGTGAAGAATGCAGAAGCCTCAGGAGCTGATTCGATCAACTGGAAGAAAGGGTATCAGCAATGGAAGATGAAATGAATGAAATGAAGCGAGAAGCGAAGTTTAGAGAAAAAGGAATAAAAAGAAATGAACAAAGCCTCCAGGAAATATGGGACTATGTGAAAAGACCAAATCTACGTCTGATTGGTGTACCTGAAAGTGACGGGGAGAATGGAACCAAGTTGGAAAACGCTCTGCAGGATATTAGCCAGGAGAACTTCCCCAATCTAGCAAGGCAGGCCAACGTTCAGATTCAGGAAATACAGAGAACGCCACAAAGATACTCCTCAAGAAGAGCAAATCCAAGACACATAATTGTCAGATTCACCAAAGTTGAAATGAAGGAAAAAATGTTAAGGGGAGCCAGAGAGAAAGGTCGGGTTACCCACAAAGGGAAGCCCATCAGACTAACAGCGGATCTCTCGGCAGAAACTCTACAAGCTAGAAGAGAGTGGGGGCCAATATTCAACATTCTTAAAGAATTTTCAACCCAGAATTTCATATCCAGCCAAACTAAGCTTCATAACTGAAGGAGAAATAAAATACTTTACAGACAAGCAAATGCTCAGAGATTTTGTCACCACCAGGCCTGCCCTAAAAGAGCTCCTGAAGGAAGCACTCAATATGGAAAGGAACAACCGGTAACAGCCGCTGCAAAATCATGTCAAAATGTAAAGACCATCGAGACTAGGAAGAAACTGCATCAACTAATGAGCAAAATAACCAGCTAATATCAAAATGACAGGATCCAATTCACACATAACAATATTAACTTTAAATGTAAATGGACTAAATGCTCCAATTAAAAGACACAGACTGGAAAATTGGATAAAGAGTCAAGACCCATCAGTGTGCTGTATTCAGGAAACTCATCTCACATGCAGAGACACACATAGGCTCAAAATAAAAGGATGGAGGAAGATCTACCAAGCAAATGGAAAAGAAAAAAAGGCAGGGGTTGCAATCCTAGTCTCTGATAAAACAGACTTTAAACCAACAAAGATCAAAAGAGACAAAGAAGGCCATTACATAATGGTAAAGGGATCAATTCAACAAGAAGAGCTAACTATCCTAAATATATATGCACCCAATACAGGAGCACCCAGATTCATAAAGCAAGTCCTGAGTGACCTACAAAGAGACTTAGACTCCCACACATTAATAATGGGAGACTTTAACACCCCACTGTCAACATTAGACAGATCAACGAGACAGAAAGTTAACAAGGATACCCAGGGATTGAACTCAGCTCCGCACCATGCAGACCTAATAGACATCTACAGAACTCTCCACCCCAAATCAACAGAATATACATTTTTTTCAGCACCACACCACACCTATTCCAAAATTGACCATACAGTTGGAAGTAAAGCTCTCCTCAGCAAATGTAAAAGAACAGAAATTATAACAAACTATCTCTCAGACCACTATGCAATCAAACTAGAACTCAGGATTAAGAAACTCACCCAAAACCACTCAACTACATGGAAACTGAACAACCTGCTCCTGAATGACTACTGGGTACATAACGAAATGAAGGCAGAGATAAAGATGTTCTTTGAAACCAATGAGAAGAAAGACACAACATACCAGAATCTCTGGGATACATTCAAAGCAGTGTGTAGAGGGAAATTTATAACACTAAATGCCCACAAGAGAAAGCAGGAAAGATCCAAAATTGACACCCTAACACCACAATTAAAAGAACTAGAAAAGCAAGAGCAAACACATTCAAAAGCTAGCAGAAGGCAAGAAATAACTAACATCAGAGCAGAACTGAAGAAAATAGAGACACAAAAAACCCTTCAAAAAATTAATGAATCCAGGAGCTGGTTTTTTGAAAGGATCAACAAAATTGACAGACTGCTAGCAAGACTAATAAAGAAAAAAAGAGAGAAGAATCAAATAGATGCAATAAAAAATGATAAAGGGGATATCACCACCGATCCCACAGAAATACAAACTACCATCAGAGAATACTATAAACACCTCTATGCAAATAAACTAGAAAATCTAGAAGAAATGGATAAATTCCTGGACACATACACTCTCCCAAGACTAAACAAGGAAGAAGTTGAATCTCTGAATAGACCAATAACAGGAGCTGAAATTGTGGCAATAATCAATAGCTTACCAACCAAAAAGAGTCCAGGACCAGATGGATTCACAGCTGAATTCTACCAGAGGTACAAGGAGGAACTGGTACCATTCCTTCTGAAACTATTCCAATCAATAGAAAAAGAGGGAATCCTCCCTAACTCATTTTATGAGGCCAGCATCATCCTGATACCAAAGCTGGGCAGAGACACAACCAAAAAAGAGAATTTTAGACCAATAACCTTGATGAACATTGATGCAAAAAATCCTCAATAAAATACTGGCAAACTGAATCCAGCAGCACATCAAAAAGCTAAAAACTCTCAATAAATTAGGTATTGATGGGACGTATCTCAAAATAATAAGAGCTATCTATGACAAACCCACAGCCAATATCATACTGAATGGGCAAAAACTGGAAGCATTCCCTTTGAAAACTGGCACAAGACAGGGATGCCCTCTCTCACCCCTCCTATTCAACATAGTGTTGGAAGTTCTGGCCAGGGCAATCAGGCAGGAGAAGGAAATAAAGGGTATTCAATTAGGAAAAGAGGAAGTCAAATTGTCCCTGTTCGCAGACGATATGACTGTATATCTAGAAAACCCCATCGTCTCAGCCCAAAATCTCCTTAAGCTGATAAGCAACTTCAGCAAAGTCTCAGGATACAAAATCAATGTACAAAAATCACAAGCATTCTTATACACCAACAACAGACAAACAGAGAGCCAAATCATGAGTGAACTCCCATTCACAATTGCTTCAAAGAGAATAAAATACCTAGGAATCCACCTTACAAGGGACGTGAAGGACCTCTTCAATGAGAACTACAAACCACTGCTCAAGGAAATAAAAGAGGATAAAAACAAATGGAAGAAGATTCCATGCTCATGGGTAGGAAGAATCAATATCGTGAAAATGGCCATATGCCCAAGGTAATTTATAGATTCAATGCCATCCCCATCAAGCTACCAATGACTTTCTTCACAGAATTGGAAAACACTACTTTAAAGTTCATATGGAACCAAAAAAGAGCCCGCATTGCCAAGTCAATCCTAAGCCAAAAGAACAAAGCTGGAGGCATCACACTACCTGACTTCAAACTATACTACAAGGCTACAGTAACCAAAACAGCATGGGACTGGTACCAAAACAGAGATATAGATCAATGGAACAGAACAGAGCCCTCAGAAATAATGCCGCATATCTACAACTATCTGATCTTTGCCAAACCTGAGAAAAACAAGCAATGGGGAAAGGATTCCCTATTTAATAAATGGTGCTGGGAAAACTGGCTAGCCATATGTAGAAAGCTGAAACTGGATCCCTTCCTTAAACCTTATACAAAAATCAATTCAAGATGGATTAAAGACCTAAACGTTAGACCTAAAACCATAAAAACCCTAGAAGAAAACCTAGGCATTACCATTCAGGACATAGACATGGGCAAGGACTTCATGTCTAAAACACCAAAAGCAATGGAAACAAAAGTCAAAATTGACAAATGGGATCTAATTAAACTAAAGAGCTTCTGCACAGCAAAAGAAACTACCATCAGAGTGAACAGGCAACCTACAAAATGGGAGAAAATTTTTGCAACCTACTTATCTGACAAAGGGCTAATATCCAGAATCTACAATGAACTCAAACAAATTTACAAGAAAAAAACAAACAACCCCATCAAAAAGTGGGTGAAGGACATGAACAGACACTTCTCAAAAGAAGCATTTATGCAGCCAAAAAACACATGAAAAAATGCTCACCATCACTGGCCATCAGAGAAATGCAAATCAAAACCACAGTGAGATACCATCTCACACCAGTTAGAATGGCAGTCATTAAAAAGTCAGGAAGCAGCAGGTGCTGGAGAGGATGTGGAGAAATGGGAACACTTTTACACTGTTGGTGGGACTGTAAACTAGTTCAACCGTTGTGGAAGTCAGTGTGGCGATTCCCCCAGGGATCTGGAACTAGAGGTACCATTTGACCTAGACATCCCATTACTGGGTATAAATCATGCTGCTATAAAGACACATGCACACGTATGTCTGTTGCGGCACTGTTCACAATAGCAAAGACTGGGAACCAACCCAAATGTCCAACAATGATAGACTGGATTAAGAAAGTGTGGCACATGTATACCATGGAATACTATGCAGCCATAAAAAATGATGAGTTCATGTCCTTTGTAGGGACATGGATGAAATTGGAAATCATCATTCTCAGTAAACTATCGCAAGAACAAAAAACCAAACACTGCATATTCTCACTCATAGGTGGGAACTGAACAACGAGAACACATGGACACAGGAAGGGGAACATCACACTCTGGGGACTGCTGTGGGGTGGGGGGAGGGGGAGGGATAGCATTGGGAGATATACCTAATGCTAGATGATGAGTTAGTGGGTGCAGTGCACCAGCATGGCACATGTATACATATGTAACTAACCTGCACATTGTGCACATGTACCCTAAAACTTAAAGTATAA*TAAT****AAAAAAAAG****AAAAA*GTATAAAGTATCCCATGAACCCACTCCAACAAGTTTCCATACAAATTCTACCTGACAAAACTTCACCCATGTCTGGGCACGGTGGCTCATGCCTGTAATCTCAGCACTTTGGGAGGCCAAGGCAGGTGGATCACCAGTCAGGAGTTTGAGACCAGCCTGGCCAACATGGTGAAAACCCATCTCTGC

**Clone 48; PA3; SpIRE(97/622)-48: Filled site spans: chr5:104717691-104723449**

Empty Site:

AAGTCAACTCAAAAGAGAAAAACTTTGTAGAAAAAAAAGTAAGATGATTTACATGTGAATGACACAATTGTATGCCCGGAAGTCCCAAGAGAATCAGCTAAAACACTAATCCCAAAATAA

AATGATGTTAT**AAGACTTTGTTA**TGTAAAATTACTGTATA

TTAAAATAGATCATAATTATTTTTTAGGTAATTCATAAATTTAATATGATACCCATGAAAATACCAAACACTAGTTGGCTGTTTATTTATTTTACTAAACAAACTAATTCTAAATTTTAT

Filled Site:

AGTCCCAAGAGAATCAGCTAAAACACTAATCCCAAAATAAAATGATGTTAT**AAGACTTTGTTA**GGGGAGGAGCCAAGATGGCCGAATAGGAACAGCTCCCGTCTACAGCTCCCAGCATGAGCGACGCAGAAGACGGGTGATTTCTGCATTTCTATCTGAGCTTTGAAGAAAGCAGTGGTTCTCCCAGTACGCAGCTGGAGATCTGAGAACGGGCAGACTGCCTCCTCATGTGACCCCCGAGCAGCCTAACTGGGAAGTACCCCCCAGGAGGGGCACACTGACACCTCACTTGACAGGGTACTCCAACAGACCTGCAGCTGAGGGTCCTGTCTGTTAGAAGGAAAACTAACAAACAGAAAGGACATCCACACCAAAAACCCATCTGTACATCACCATCATCAAAGACCAAAAGTAGATAAAACCACAAAGATGGGGAAAAAACAGAACAGAAAAACTGGAAACTCTAAAAAGCAGAGCGCCTGTCCTCCTCCAAAGGAACGCAGTTCCTCACCAGCAACAGAACAAAGCCGGTCGGAGAACGACTTTGACGAGCTGAGAGAAGAAGGCTTCAGACGATCAAATTACTCTGAGCTACGGGAGGACATTCAAACCACAGGCAAAGAAGTTGAAAACTTTGAAAAAAATTTAGAAGAATGTATAACTAGAATAACCAATACAGAGAAGTGCTTAAAGGAGCTGATGGAGCTGAAAACCAAGGCTTGAGAACTATGTGAAGAATGCAGAAGCCTCAGGAGCCGATGCGATCAACTGGAAGAAAAGGTATCAGCAATGGAAAATGAAATGAATGAAATGAAATGAGAAGGGAAGTTTAGAGAAAAAAGAATAAAAAGAAATGAGCAAACCCTCCAAGAAATATGGGACTATGTGAAAAGACCAAATCTACGTCTGATTGGTGTGCCTGAAAGTGATGGGGAGAATGGAACCAAGTTGGAAAACACTCCGCAGGATATTATCCAGGAGAACTTCCCCAATCTAGCAAGGCAGGCCAATGTTCAGATTCAGGAAATACAGAGAACACCACAAAGATACTCCTCGAGAAGGGCAACTCCAACACACATAATTGTCAGATTCACCAAAGTTGAAATGAAGGAAAAAATGTTAAGGGCAGCCAGAGAGAAAGGTCGGGTTACCCTCAAAGGGAAGCCCATCAGACTAACAGTGGATCTCTCGGCAGAAACCCTACAAGCCAGAAGAGAGTGGGGGCCAACATTCAACATTCTTAAAGAAAAGAATTTTCAACCCAGAATTTCATATCCAGCCAAACTAAGCTTCATAAGTGAAGGGGAAATAAAATACTTTACAGACAAGCAAATGCTGAGAGATTTTGTCACCACCAGGCCTGCCCTAAAAGAGCTCCTGAAGGAAGCGCTAAACATGGAAAGGAACAACTGGTACCAGCCGCTGCAAAATCATGCCAAAATGTAAAGACCATCGAGACTAGGAAGAAACTGCATCAACTAACGAGCAAAATCACCAGCTAACATCATCATGAGAGGATCAAATTCACACATAACAATATTAACTTTAAATGTAAATGGCTAAATGCTCCAATGAAAAGACACAGACTGGCAAATTGGATAAAGAGTCAAGACCCATCAGTGTGCTGTATTCAGGAAACCCATCTCACATGCAGAGACACACATAGGCTCAAAATAAAAGGATGGAGGAAGATCTACCCAGCAAATGGAAAACAAAAAGGCAGGGGTTGCAATCCTAGTCTCTGATAAAAGAGACTTTAAACCAACAAAGATCAAAAGAGACAAAGAAGGCCATTACATAATGGTAAAGGGATCAATTCAACAAGAAGGGCTAACTATCCTAAATATATATGCACCCAATACAGGAGCAACAAGATTCATAAAGCAAGTCCTGAGTGACCTACAAAGAGACTTAGACTCCCACACATTAATAATGGGAGACTTTAACACCCCACTGTCAACATTAGACAGATCAACGAGACAGAAAGTCAACAAGTATACCCAGGAATTGAACTCAGCTCTGCAACAAGCGGACCTAATAGACATCTACAGAACTCTCCACCCCAAATCAACAGAATATACATTTTTTTCAGCACCACACCACACCTATTCCAAAATTGACCACATACTTGGAAGTAAAGCTCTCCTCAGCAAATGTAAAAGAACAGAAATTATAACAAACTATCTCTCAGACCACAGTGCAATCAAACTAGAACTCAGGATTAAGATTCTCACTCAAAACCGCTCAACTACATGGAAACTGAACAACCTGCTCCTGAATGACTACTGGGTACATAACGAAATGAAGGCAGAAATAAAGATGTTCTTTGAAATCAACGAGAACAAAGATACAACATACCAGAATCTCTGGGACGCATTCAAAGCAGTGTGTAGAGGGAAACTTATAGCTCTAAATGCCCACAAGAGAAAGCAGGAAAGATCCAAAATTGACACCCTAACATCACAATTAAAAGAACTAGAAAAGCAAGAGCAAACACATTCAAAAGCTAGCAGAAGGCAAGAAATAACTAAAATCAGAGCAGAACTGAAGGAAATAGAGACACAAAAAACTCTTCCAAAAATTAATGAATCCAGGAGCTGGTTTTTTGAAAGGATCAACAAAATAGATAGACCGCTAGCAAGACTAATAAAGAAAAAAAGAGAGAAGAATCAAATAGACACAATAAAAAATGATAAAGGGGATATCACCACCGATCCCACAGAAATACAAACTACCATCAGAGAATACTACAAACACCTCTACACAAATAAACTAGAAAATCTAGAAGAAATGGATAAATTCCTCGACACATACACTCTCCCAAGACTAAACCGGGAAGAAGTTGAATCTCTGAATAGACCAATAACAGGATCTGAAATTGTGGCAATAATCAATAGCTTACCAACAAAAAAGAGTCCAGGACCAGATGGATTCACAGCTGAATTCTACCAGAGGTACAAGGAGGAACTGGTACCATTCCTTCTGAAACTATTCCAATCAATAGAAAAAGAGGGAATCCTCCCTAACTCATTTTATGAGGCCAGCATCATTCTGATACCAAAGCCTGGCAGAGACACAACAAAAAAAGAGAATTTTAGACCAATATCCTTGATGAACATTGATGCAAAAATCCTCAATAAAATGCTGGCAAAATGAATCCAGCAGCACATCAAAAAGCTTATCCATCATGATCAAGTGGGCTTCATCCCTGGGATGCAAGGCTGGTTCAATATACACAAATCAATAAATGTAATCCAGCATATAAACAGAGCCAAAGACAAAAACCACATGATTATCTCAATAGATGCAGAAAAGGCCTTTGACAAAATTCAACAACCTTTCATGCTAAAAACTCTCAATAAATTAGGTATTGATGAGACGTTTTTCAAAATAATAAGAGCTATCTATGACAAACCCACAGCCAATATCATATAGAATGGGCAAAAACTGGAAGCATTCCCTTTGAAAACTGGCACAACACAGGGATGCCCTCTCTCACCACTCCTATTCAACACAGTGTTGGAAGTTCTGGCCAGGGCAATTAGGCAGGAGAAGGAAATAAAGGGTATTCAATTAGGAAAAGAGGAAGTCAGATTGTTCCTGTTTGCAGACGACATGATTGTATATCTAGAAAACCCCATTGTCTCAGCCCAAAATCTCCTTAAGCTGATAAGCAACTTCAGCAAAGTCTCAGGATACAAAATCAATGTACAAAAATCACAAGCATTCTTAGAAACCAACAACAGACAAACAGAGAGCCAAATCATGAGGGAACTCCCATTCACAATTGCTTCAAAGAGAATAAAATACCTAGGAATCCAACTTACAAGGGATGTGAAGGACCTCTTCAAGGAGAACTACAAGCCACTGCTCAAGGAAATAAAGGAGGATACAAACAAATAGAAGAACATTCCATGCTCATGGGTAGGAAGAATCAATATCGTGAAAATGGCCATACTGCCCAAGGTAATTTACAGATTCAATGCCATCCCCATCAAGCTACCAATGCCTTTCTTCACAGAATTGGAAAAAACTACTTTAAAGTTCATATGGAACCAAAAAAGAGCCCGCATCGCCAAGTCAATCCTAAGCCAAAAGAACAAAGCTGGAGGCATCGTGCTACCTGACTTCAAACTATACTACAAGGCTACAGTAACCAAAACAGCATGGTACTGGTACCAAAACAGAGATATAGACCAATGGAACAGAACAGAGCCCTCAGAAATAACGCCACATATCTACAACTATCTGATCTTTGACAAACCTGAGAAAAACAAGCAATGGGGAAAGGATTCCCTATTTAATAAATGGTGCTGGGAAAACTGGCTAGCCATATGTAGAAAGCTGAAACTGGATCCCTTCCTTACACCTTATACAAAAATCAATTCAAGATGGATTAAAGACTTAAATGTTAGACCTAAAACCATAAAAACCCTAGAAGAAAACCTAGGCAATACCATTCAGGACATAGGCGTGGGCAAGGACTTCATGTCTAAAACACCAAAAGCAATGGCAACAAAGACAAAAGTGACAAATGGGATCTAAAGAAACTAAAGAGCTTCTGCACAGCAAAAGAAACTACCATCAGAACGAACAGGCAACCTACAAAATGGGAGAAAATTTTCGCAACCTACTCATCTGACAAAGGGCTAATATCCAGAATCTACAATGAACTCAAACAAATTTACAAGAAAAAAACAAACAACCCCATCAAAAAGTGGGCAAAGGACATGAACAGACACTTCTCAAAAGAAGACATTTATGCAGCCAAAAAACACATGAAAAAATGCTCATCATCACTGGCCATCAGAGAAATGCAAATCAAAACCACAATGAGATACCATCTCACACCAGTTAGAATGGCAATCATTAAAAAGTCAGGAAACAACAGGTGCTGGAGAGGATGTGGAGAAATAGGAACACTTTTACACTGTTGGTGGGACTGTAAACTAGTTCAACCATTGTGGAAGTCAGTGTGGAGATTCCTCAGGGATCTAGAACTAGAAATACCGTTTGACCCAGCCATCCCATTACTGGGTATATACCCAAAGGACTATAAATCACGCTGCTATAAAGACACATGCACACGTATGTTTATTGCGGCACTATTCACAATAGCAAAGACTTGGAACCAACCCAAATGTCCAACACTGATAGACTGGATTAAGAAAATGTGGCACATATACACCATGGAATACTATGCAGCCATAAAAAATGATGAGTTCATGTCCTTTGTAGGGACATGGATGAAATTGGAAATCATCATTCTCAGTAAACTGTCGCAAGAACAAAAAACCAAACACCGCATATTCTCACTCATAGGTGGGAATTGAACAATGAGATCACATGGACACAGGAAGGGGAACATCACACTCTGGGGACTGTTGTGGGGTGGGGGGAGGGGGGAGGGATAGCATTGGGAGATATACCTAATGCTAGATGACGAGTTAGTGGGTGCAGCGCACCAGCATGGCACATGTATACATATGTAACTAACCTGCACAATGTGCACATGTACCTTAAAACTTAAAGTATAA*TAATAAAAGAAAAA****AAGA*CTTTGTTA**TGTAAAATTACTGTATATAAATTAATAATTTCCACATACAATATACTGCAAGAAATTTCCAACTTAAAATATTAATGACATATTTAGGAGTGTGTACAACATGAAGAATTCTCACTAAAACAAAAGAAATCTTGAAAAAAACAAGACGTTCTTGAAAAGAAAGACATATTAAAATAGATCATAATTATTTTTTAGG

**Clone 53; PA3; SpIRE(97/622)-53: Filled site spnas: chr6:76745423-76751192**

Empty Site:

AAAATTGAAAGAATATGGGTGTGCTCAATTTAGCAACTCTAACTAGATCCCTCAACCATAATTTTAATGGTTATATAGCACTTGCCTGTGGTAATAAATAGTAATTGACCATACTAATTTCACCTTTA

GAAAC**TTTAGTTTA**TTTTTTATTGATAATTGGGTGTTTCT

CACAGAGGGGGATTTGGCAGGGTCATAGGACAATAGTGGAGGGAAGGTCAGCAGATAAACAAGTGAACAAAGGTCTCTGGTTTTCCTAGGCAGAGGACCCTGCGGCCTTCTG

Filled Site:

AATAAATAGTAATTGACCATACTAATTTCACCTTTAGAAAC**TTTAGTTTA**GGGGGAGCAGCCAAGATGGCCAAATAGGAACAGCTCCCGTCTACAGCTCCCAGCGTGAGCGAGGCAGAAGATGGGTGATTTCTGCATTTCCATCTGAGCTTTGAAGAGAGCAGTGGTTCTCCCAGCATGCAGCTGGAGATCTGAGAATGGGCAGACTGCCTCCTCAAGTGGGTCCCTGACCCCTGACCCCCGAGCAGCCTAACTGGGAGGCATCCCCTAGCAGGGGCAGACTGACACCTCACACGGCCAGGTACTCCAAAAGACCTGCAGCTGAGGGTCCTGTCTGTTAGAAGGAAAACTAACAGAAAGGACATCCACACCAAAAATCTATCTGTACATCACCATCATCAAAGACCAAAAGTAGATAAAACCACAAAGATGGGAAAAAAACAGAACAGAAAAACTGGAAACTCTACAGAGCACCTCTCCTTCTCCAAAGAAACACAGTTCCTCACCAGCAACGGAACAAAGCTGGACGGAGAATGACTTTGACAAGCTCAGAGAAGAAGCCTTCAGACAATCAAATTACTCCGAGCTATGGGAGGACATTCAAATCAAAGGCAAAGAACTTGAAAACTTTGAAAAAAATTAAGAAGAATGTATAACTAGAATAACCAATACAGAGAAGTGCTTAAAGGAGCTGATGGAGCTGAAAACCAAGGCTCGAGAACTACGTGAAGAATGCAGAAGCCTCAGGAGCCGATGCGATCAACTGGAAGAAAGGGTATCAGCGATGGAAGATGAAATGAATGAAATGAAGTGAGAAGGGAAGTTTAGAGAAAAAAGAATAAAAAGAAATGAGCAAAGCCTCCAAGAAATATGGGACTATGTGAAAAGACCAAATCTATGTCTGATTGGTGTACCTGAAAGTGACAGGGAGAATGGAACCAAGTTGGAAAACACTCTGCAGGATATTATCCAGGAGAACTTCCCCAATCTAGCAAGGCAGGCCAACATTCACATTCAGGAAATACAGAGAATGACACAAAGATACTCCTCGAGAAGAGCAACTCCAAGACACATAATTGTCAGATTCACCAAAGTTGAAATGAAGGAAAAAATGTTAAGGGCAGCCAGAGAGAAAGGTCGGGTTACCCTCAAAGAGAAGCACATCAGACTAACAGCAGATCTCTCAGTAGACACTCTACAAGCCAGAAGAGAGCGGGGGCCAATATTCCACATTCTTAAAGAAAAGAATTTTCAACCCAGAATTTCATATCCAGCCAAACTAAGCTTCATAAGTGAAGGAGAAATAAAATACTTTACAGATAAGCAAATGCTGAGAGATTTTGTCACCACCAGGCCTGCCTTACAAGAGCTCCTGAAGGAAGCACTAAACATGGAAAGGAAAAACCGGTACCAGCCCCTGCAAAATCATGCCAAAATGTAAAGCGCATCGAGACTAGGAAGAAACTGCATCAACTAACCAGCAAAATAACCAGCTAACATCATAATGACCAGATCAAATTCACACATAACAATATTAACTTTAAATGTAAATGGATTAAATGCTCCAATTAAAAGACACAGACTGGCAAATTGGATAAAGAGTCAAGACCCATCAGTGTGCTGTATTCAGGAAACCCGTCGCACGTGCAGAGATGCACATAGGCTCAAAATAAAAGGATGGAGGAATATCTACCAAGCAAATGGAAAATAAAAAAAGGCAGGAGTTGCAATCCTAGTCTCTGATAAAACAGACTTTAAACCAACAAAATCAAAAGAGACAAAGAAGGCCATTACTTAATGGTAAAGGGATCAATTCAACAAGAAGAGCTAACTATCCTAAATATATATGCACCCAATACAGGAGCACCCAGATTCATAAAATAAGTCCTGAGTGACCTACAAAGAGACTTAGACTCCCACACATTAATAATGAGAGACTTTAACACCCGACTGTCAACATTAGACCAATCAACGAGACACAAAGTCAACAAGGATACCCAGGAATTGTACTCAGCTCTGCACTAAGCATACCTAATAGACATCTACAGAACTCTCCACCCCAAATCAACAGAATATACATTCTTTGCAGCACCACACCACACCTATTCCAAAAGTGACCACATACTTGCAAGTAAAGCTCTCCTCAGCAAATGTAAAAGAACAGAAATTATAACAAACTATCTCTCAGACCACAGTGCAATCAAACTAGAACTCAGGATTAAGAATCTCACTCAAAACCACTCAACTACATGGAAACTGAACAACCTGCTCCTGAATGACTACTAGGTACATAACGAAATGAAGGCAGAAATAAAGATGTTCTTTGAAACGAATGAGAACAAAGACACAACATATCAGAATCTCTGGGACACATTCAAAGCAGTGTGTAGAGGGAAATTTATAGCACTAAATGCCCACAAGAGAAAGCAGGAAAGATCCAATATTGACACCCTAACATCACAATTAAAAAAACTAGAAAAGCAAGAGCAAACACATTCAAAAGCTAGCAGAAGGCAAGAAATAACTAAAATCAGAGCAGAACTGAAGGAAATAGAGACACAAAAAACCCTTCAAAAAATTAACAAATCCAGGAGCTGGTTTTTTGAAAGGATCAACAAAATTGATAGACCGCTAGCAAGACTAATAAAGAAAAAGAGAAGAATCAAATAGACGCAGTAAAAAATGATAAGGGGGATATCACCACCCATCCCACAGAAATACAAACTACCATCAGAGAATATAACAAACACCTCTATGCAAATAAACTAGAAAATCTAGAAGAAATGGATAAATTCCTCGACACATACACTCTCCCAAGACTAAACCAGGAAGAAGCTGAATCTCTGAATAGACCAATAACAGGAGCTGAAATTGTGGCAATAATCAATAGCTTGCCAACGAAAAAGAGTCCAGGACCACATGGATTCACAGCCGAATTCTACCAGAGGTACAAGGAGGAACTGGTACCATTCCGTCTGAAACTATTCCAATCAATAGAAGAAGAGGGAATCCTCCCTAACTCATTTTATGAGGCCAGCATCATCCTGATACCAAAGCCTGGCAGAGACACAACAAAAAAAGAGAATTTTAGACCAATATCCTTAATGAACATTGATGCAAAAATCCTCAATAAAGTACTGGCAAACCGAAACCAGCAGCACATCAAAAAGCTTATCCACCATGATCAAGTGGGCTTCATCCCTGGGGTGCAAGGCTGGTTCAATATATGCAAATCAATAAATGTAATCCAGCATATAAACAGAACCAAAGACAAAAAACACATGATTATCTCAATAGATGCAGAAAAGGCCTTTGACAAAATTCAACAGCCCTTCATGCTAAAAACTCTCAAAAAATTAGGTATTGATGGGATGTATTTCAAAATAATAAGAGCTATCTATGACAAACCCACAGCCAATGTCGTACTGAATGGGCAAAACCTGGAAGCATTCCCTTTGAAAACTGGCACAAGACAGGGATGCCCTCTCTCACCACTCCTATTCAACATAGTGTTGGAAGTTCTGGCCAGGGCAATAAGGCAGGAGAAGGAAATAAAGGGTATTAAATTAGGAAAAGAGGAAGTCAAATTGTCCCTGTTTGCAGACGACATGACTGTATATCTAGAAAACCCCATTGTCTCAGCCCAAAATCTCCTTAAGCTGATAAGCAACTTCAGCGAAATCTCAGGATACAAAATCAATGTACAAAAATCACAAGCATTCTTATACACCAACAACAGACAAACAGAGAGCCAAATCATGAGTGAACTCCCATTCACAATTGCTTCAAAGATAATAAAATACCTAGGAATTCAACTTACAAGGGATGTGAAGGACCTCTTCAAGGAGAACTACAAACCACTGCTCAAGGAAATAAAAGAGGATACAAACAAATGTAAGGATATTCCATGCTCATGGGTAGGAAGAATCAATATCATGAAAATGGCCATAATGCCCAAGGTAATTTACAGATTCAATACTATCCCCATCAAGCTACAAATGACTTTCTTCACAGAATTGGAAAAAACTACTTTAAAGTTCATATGGAAACAACAAAGAGCCTGCATTGCCAAGTGAATCCTAAGCCAAAAGAACAAAGCTGGAGGCATCACACTACCTGACTTCAAACTATACTACAAGGCTACAGTAACCAAAACAGCATGGTACTGGTACCAAAACAGAGATATAGATCAATGGAACAGAACAGAGCCCTCAGATATAACACCACATATCTACAAGTATCTGATCTTTGACAAACCTGAGAAAAACAAGCAATGGGGAAAGGATTCCCTATTTAATAAATGGTGTTGGGAAAACTGGCTAGCCATATGTAGAAAGCTGAAACTGGATCCCTTCCTTACACCTTACACAAAAATCAATTCAAGATGGATTAAAGACTTAAACGTTAGACCTAAAACCATAAAAACCCTAGAAGAAAACCTAGGCATTACCATTCAGGACATAGGCATGGGCAAGGACTTCATGTCTAAAACACCAAAAGCAATGGCAACAAAAGCCAAAATTGACAAATTGGATCTAATTAACCTAAAGAGCTTCTGCACAGCAAAAGAAACTATCATCAGAGTAAACAGGCAACCTACAAAATGGGAGAAAATTTTCACAACCTACTCATCTGACAAAGGGCTAATATCCAGAATCTACAATGAACTCAAACAAATTTACAAGAAAAAAACAAACAACCCCATCAAAAAGTGGGCGAAGGACATGAACAGACACTTCTCAAAAGAAGACATTTATGCAGCCAAAAGACACATGAAAAAATGCTCACCATCACTGGTCATCAGAGAAATGCAAATCAAAACCATAATGAGATACCATCTCACACCAGTTAGAATGGCGATCATTAAAAAGTCAGGAAACAACAGGTGCTGGAGAGGATGTGGAGAAATACGAACACTTTTACACTGTTGGTGGGACTGTAAACTAGTTCAACCATTGTGGAAGTCAGTGTGGCGATTCCTCAGGGATCTAGAACTAGAAATATCATTTGACCCAGCCATCCCATTACTGGGTATATGCCCAAAGGACTATAAATCATGCTGCTATAAAGACACATGCACACGTATATTTATTGCGGCACTATTCACAATAGCAAAGACTTGGAACCAACCCAAATGTCCAACAATGATAGACTGGATTAAGAAAATGTGGGACATATACACCATGGAATATTATGCAGCCATAAAAAATGATGAGTTCATGTCCTTTATAGGGACATGGATGAAATTGGAAATCATCATTCTCAGTAAACTATCGCAAGAACAAAAAACCAAACACCGCATATTCTCACTCATAGGTGGGAATTGAACAATGAGAACACATGGACACAGGAAGGGGAACATCACACTCTGGGGACTGTTGTGGGGTGGGGGACGGGGGAGGGATAGCATTGGGAGATATACCTAATGCTAGATGACGAGTTAGTGGGTGCAGCGCACCAGCATGGCACATGTATACATATGTAACTAACCTGCACATTGTGCACATGTACGCTAAAACTTAAAGTATAAT*AATAATAAATAAATAAATAAATAAATAAATAAAAACAAATAAAATAAAATAAAATAAAATAAAAATAATAATAAAAAGAAATGTTATTACCTTATATTAACAATTATAAATAATATGTTATACATGCATCCTTATATAATAATTTTCTTTTTTTTT****TTTAATTTA***TTTTTTATTGATAATTGGGTGTTTCTCACAGAGGGGGATTTGGCAGGGTCATAGGACAATAGTGGAGGGAAGGTC

**Clone 54; PA3; SpIRE(97/622)-54: Filled site spans:chr6:115008902-115014586**

Empty Site:

AAATTTAATATACAGTAATATACTTAATCTATTAGTACATAATTTTTTCTTTAATAATACAGTTCTTTTTTTCAGCAATAACTAACTTTTGGATTAGTCATCATCAAGTAAAATACAAAT

ACTCCAATAGAAACATAGATATC**ATTTTCTG**ATCTCAAAA

AAAAAAAAAAAAAAAAGAAATATAGCTATCATTCAGAAATGGGCAATTTACAAAAGAAAACAAATTTCTACTAATGATATCACAATTACAAATTCCATGTGTAAATATGAGCATATTAA

Filled Site:

ACTAACTTTTGGATTAGTCATCATCAAGTAAAATACAAATACTCCAATAGAAACATAGATATC**ATTTTCTG**GCAGGGGAGGAGCCAAGATGGCCAAATAGGAACAGCTCCGGTCTACAGCTCCCAGCCTGAGCGACACAGAAGATGGGTGATTTCTGCATTTCCATCTGAGCTTTGAAGAGAGCAGTGGTTCTCCCAGTACGCAGCTGGAGATCTGAGAACGGGCAGACTGCCTCCTCAAGTGGGTCCCTGACTCCTGACCCCCGAGCAGCCTAACTGGGAGGCACCCTCCAGCAGGGGCACACTGACACCTCACACTGCAGGGCATTCCAACAGAACTGCAGCTGAGGGTCCTGTCTGTTAGAAGGAAAACTAACAAACAGAAAGGACATCCACACCAAAAACCCATCTGTACATCACCATCTTCAAAGACCAAAAGTAGATAAAACCACAAAGATGGGGAAAAAACAGAACAGAAAAACTGGAAACTCTAAAAATCAGAGCGCCTCTCCTCCTCCAAAGGAACGCAGCTCCTCACCAGCAATGGAACAAAGCTGGACGGAGAATGACTTTGACGAGCTGAGAGAAGAAGACTTCAGACGATCAAATGACTCTGAGTTACGGGAGGACATTCAAACCAAAGGCAAAGAAGTTGAAAACTTTGAAAAAAATTTAGAAGAATATATAACTAGAATAAACAATACAGAGAAGTGCTTAAAGGAGCTGATGGAGCTGAAAATCAAGGCTCAAGAACTACGTGAAGAATGCAGAAGCCTCAGGAGCCAATGTGATCAACTGGAAGAAAGGGTATCAGTAATGGAAGACGAAATGAATGAAATGAAGCGAGAAGGAAAGTTTAGAGAAAAAAGAATAAAAAGAAATGAGCAAAGCCTCCAAGAAACATGGGACTATGTGAAAAGACCAAATCTACGTCTGATTGGTGTACCTAAAAGTGATGGGGAGAATGGAACCAAGTTGGAAAACACTCTGCAGGATATTATCCAGGAGAACTTCCCCAATCTAGCAAGGCAGGCCAACGTTCAGATTCAGGAAATACAGAGAATACCACAAAGATACTCCTCGAAAAGAGCAACTCCAAGACACATAATTGTCAGATTCACCAAAGTTGAAATGAAGGAAAAAATGTTAAGGGCAGCCAGAGAGAAAGGTTGGGTTACCCTCAAAGGGAAGCCCATCACACTAACAGTGGATCTCTTGGCAGAAACCCTACAAGCCAGAAGAGAGTGGGGGCCAATATTCAACATTCTTAAAGAAAAGAATTTTCAACCCAGAATTTCATATCCAGCCAAACTAAGCTTCATAAGTGAAGGAGAAATAAAATCCTTTACAGACAAGCAAATGCTGAGAGATTTTGTCACCACTAGGCCTGCCCTAAAAGAGCTCCTGAAGGAAGCACTAAACATGGAAAGGAACAACCAGTACCAGCCGCTGCAAAATCATGCCAAAATGTAAAGACCGTCGAGACTAGGAAGAAACTGCATCAACTAACGAGCAAAATAACCAGCTAACATCATAATGACAGAATCAAATTCACACATAACACTATTAACTTTAAATGTAAATGGACTAAATGCTCCAATTAAAAGACACAGACTGGCAAATTGGATAAAGAGTCAAGACCCATCAGTGTGCTGTATTCAGGAAACCCATCTCACATGCAGAGACACACATAGCCTCAAAATAAAAGGATGGAGGAAGATCTACCAAGCAAATGGAAAACAAAAAAAGGCAGGGGTTGCAATCCTAGTCTCTGATAAAACAGACTTTAAACCAACAAAGATCAAAAGAGACAAAGAAGGCCATTACATAATGGTAAAGGGATCAATTCAACAAGAAGAGCTAACTATCCTAAATATATATGCACCCAATACAGGAGCACCCAGATTCATAAAGCAAGTCCTGAGTGACCTACAAAGAGACTTAGACTCCCACACATTAATAATGGGAGACTTTAACACCCCACTGTCAACATTAGACAGATCAACAAGACAGAAAGTCAACAAGGATACCCAGGAATTGAACTCAGCTCTGCACCAAGCGGACCCAATAGACATCTAAAGAACTCTCCACCCCAAATCAACAGAATATACATTTTTTTCAGCACCACACCACACCTATTCCAAAATTGACCACACAGTTGGAAGTAAAGCTCTCCTCAGCAAATGTAAAAGAACAGAAATTACAATAAACTATCTCTCAGACCACAGTGCAATCAAACTAGAACTCAGGATTAAGAATCTCACTCAAAACCGCTCAACTACATGGAAACTGAACAACCTGCTCATGAATGACTACTGGGTACATAACGAAATGAAGGCAGAAATAAAGATGTTCTTTGAAACCAACGAGAACAAAGACACAACATACCAGAATCTCTGGGATGCATTCAAAGCAGTGTGTAGAGGGAAATTTATAGCACTACATGCCCACAAGAGAAAGCAGGAAAGATCCAAAATTGACACCCTAACATCACAATTAAAAGAACTAGAAAAGCAAGAGCAAACACATTCAAACGCTAGCAGAAGGCAAGAAATAACTAAAATCAGAGCAGAACTGAAGGAAATAGAGACACAAAAAACCCTTCAAAAAATTAATGAATCCAGGAGCTGGTTCTTTGAAAGGATCAACAAAATTGATAGACCACTAGCAAGACTAATAAAGAAAAAAAGAGAGAAGAATCAAATAGATGCAATAAAAAATGATAAAGGGGATATCAGCACCGATCCTACAGAAATACAAACTACCATCAGAGAATACTACAAACACCTCTACGCAAATAAACTAGAAAATCTAGAAGAAATGGATAAATTCCTTGACACATACACTCTCCCAAGACTAAACCACGAAGAAGTTGAATCTCTGAATAGACCAATAACAGGATCTGAAATTGTGGCAATAATCAATAGCTTACCAACCAAAAAGAGTCCAGGACCAGATGGATTCACAGCTGAATTCTAACAGAGGTACAAGGAGGAACTGGTACCATTCCTTCTGAAACTATTCCAATCAATAGAAAAAGAGGGAATCCTCCCTAACTCATTTTATGAGGCCAGCATCATTCTGATACCAAAGCCGGGCAGAGACACAACCAAAAAAGAATTTTAGACCAATATCCTTGATGAACATTGATGCAAAAATCCTCAATAAAATACTGGCAAAACGAATCCAGCAGCACATCAAAAAGCTTATCCACCATGATCAAGTGGGCTTCATCCCTGGGATGCAAGGCTGGTTCAATATACGCAAAGCAATAAATGTAATCCAGCATATAAACAGAGCCAAAGACAAAAACCACATGATTATCTCAATAGATGCAGAAAAGGCCTTTGACGAAATTCAACAACCTTTCATGCTAAAAACTCTCAATATATTAGGTATTGATGGGACATATTTCAAAATAATAAGAGCTATCTATGACAAACCCACAGCCAATATCATACTGAATGGGCAAAAACTGGAAGCATTCCCTTTGAAAACTGGCACAAGACAGGGATGCCCTCTCTCACCACTCCTATTCAACATAGTGTTGGAAGTTCTGGCCAGGGCAATTAGGCAGGGGAAGGAAATAAAGGGTATTCAATTAGGAAAAGAGGAAGTCAAATTGTCCCTGTTTGCAGATGACATGATTGTATATCTAGAAAACCCCATTGTCTCAGCCCAAAATCTCCTTAAGCTCATAAGCAACTTCAGCAAAGTCTCAGGATACAAAATCAATGTGCAAAAATCACAAGCATTCCTATACACCAACAACAGACAAACAGAGAGCCAAATCATGAGTGAACTCCCATTCACAATTGCTTCAAAGAGAATAAAATACCTAGGAATCCAACTTACAAGGGATGTGAAGGACCTTTTCAAGGAGAACTACAAACCGCTGCTCAAGGAAATAAAAGAGGATACAAACAAATGGAAGAACATTCCATGCTGATGAGTAGGAAGAATCAATATCGTGAAAATAGCCATATTGCCCAAGGTAATTTATAGATTCAATGCCATCCCCATCAAGCTACCAATGACTTTCTTCACACAATTGGAAAGAACTACTTTAAAGTTCATATGGAACCAAAAAAGAGCCCGCATCGCCAAGTCAATCCTAAGCCAAAAGAACAAAGCTGGAGGCATCACACAATCTGACTTCAAACTATACTACAAGGCTACAGTAACCAAAACAGCATGGTACTGGTACCAAAACACAGATATAGATCAATGGAACAGAACAGAGCCCTCAGAAATAACGCCGCAAATCTACAGCTATCTGATCTTTGACAAACCTGAGAAAAACAAGCAATGGGGAAAGGATTCCCTATTTAATAAATGGTGCTGGGAAAACTGGCTAGCCATGTGTAGAAAGCTGAAACTGGATCCCTTCCTTACACCTTATACAAAAATCAATTCAAGATGGATTAAAGACTTACATGTTAGACCTAAAACCATAAAAATCCTAGAAGAAAACCTAGGCATTACCATTCAGGACATAGGCATGGGCAAGGACTTCATGACTAAAACACCAAAAGCAATAGCAACAAAAGACAAAATTGACAAATGGGATCTAATTAAACTAAAGAGCTTCTGCACAGCAAAATAAACTACCATCAGAGTGAACAGGCAACCTACAAAATGGGAGAAAATTTTTGCAACCTAATCATCTGACAAAGGGCTAATATGCAGAATCTACAATGAACTCAAACACATTTACAAGAAAAAAACAAACCCCATCAAAAAGTGGGCGAAGGACATGAACAGAGATTTCTCAAAAGAAGACATTTATGCAGCCAAAAAGCACATGAAAAAATGCTCACCATCACTGGCCATCAGAGAAATGCAAATCAAAACCACAATGAGATACCATCTTACACCAGTTAGAATGGCAATCATTAAAAAGTCAGGAAACAACAGGTGTTGGAGAGGATGTGGAGAAATAGGAACACTTTTACACTGTTGGTGGGACTGTAAACTAGTTCAACCATTATGGAAGTCAGTGTGGTGATTCCTCAGGGATCTAGAACTAGAAATACCATTTGACCCAGCCATCCCATTACTGGGTATATACCCAAAGGACTATAAATCATGCTGCTATAAAGACACACGCACATGTATGTTTATTGCGGCATTATTCACAATAGCAAAGACTTGGAACCAACCCAAATGTCCAACAGTGATAGACTGGATTAAGAAAATGTGGCACATATACACCATGGAATACTATGCAGCCATAAAAAATGATGAGTTCATGTCCTTTGTAGGGACATGGATGAAATTGGAAACCATCATTCTCAGTAAACTATTCCAAGAACAAAAAACCAAATGCTGCATATTCTCACTGATAGGTGGGAATTGAACAATGAGATCACATGGACACAGGAAGGGGAACATCACACTCTGGGGACTGTTGTGGGTTGGGGGGAGGGGGGAGGGATAGCATTGGGAGATATACCTAATGCTAGATGACGAGTTAGTGGGTGCAGGACACCAGCATGGCACATGTATGCGTATGTAACTAACCTGCACAATGTGCACATGTACCCT*AAAACTTAAAGTATAATAATAAAAAAAATATAAATAAATACAACTCT****ATTTTCTG****ATCTCAAAAAAAAAAAAAAAAAAAAGAAA*TATAGCTATCATTCAGAAATGGGCAATTTACAAAAGAAAACAAATTTCTACTAAT

**Clone 56; PA3; SpIRE(97/622)-56: Filled site spans: chr6:82632714-82638294**

Empty Site:

GTTCATTGATGGGAAAAATAATAAAGAAAATGTGGTATATACATGCAATGGAATAGTATTCAGTCTTTAGAAGAAAGAAATCTTGCCATATACATCAACATGGATAAACCTGGAAGATAC

TAACCT**AAGTAAAAGAAGCC**AGTGGCAGAAGGCCACAGGC

CACATGAACCCAGTTATATGCAATGTCCATCAAATCAGAAAGCAGATCTGTGGACGCCGAGGATGGGGAGTGGGGGAAGAGAGGGGCTGACTGCTAATGGGTACAGCGTTTCTTTTTTGC

Filled Site:

TCTTGCCATATACATCAACATGGATAAACCTGGAAGATACTAACCT**AAATAAAATAAGCC**GCAAGATATCCAAATAGGAACAGCTCCGGTCTACAGCTCCCAGCGTGAGCGACGCAGAAGACGGGTGACTTCTGCATTTCCATCTGAGCTTTGAAGAGAGCAGTGGTTCTCCCAGCACGCAGCTGGAGATCTGAGAACGGGCAGACTGCTTTCTCAAGTGGGTCCCTGATCCCTGACACCCGAGCAGCCTAACTGGGAGGAACCCCCCAGCAGGGGCAGACTGACACCTCACATGGCCCAGTACTCCAACAGACCTGCAGCTGAGGGCCCTGTCTGTTAGAAGGAAAACTAACAAACAGAAAGGACATCCACACCAAAAACCCATTTGTACATCACCATCATCAAAGACCAAAAGTAGATTAAACCACAAAGATGGGGAAAAACAGAGCAGAAAAACTGGAAACTCTAAAAAGCAGAGCACCTCTCCTCCTCCAAAGGAACACAGTTCATCATCAGTAATGGAACAAAGCTGGACGGAGAATGACTTTGACGAGCTGAGAGAAGAAGGCTTCAGATGATCAAATTACTCTGAGCTACGGGAGGACATTCAAACCAAAGGAAAAGAAGTTGAAAACTTTTTAAAAAATTTAGAAGAATGTGTAACTAGAATAACCAATACAGAGAAGTGCTTAAAGGAGCTGGTGGAGCTGAAAACCAAGGCTCAAGAACTACGTGAAGAATGCAGAAGCCTCAGGAGCCGATGCGATCAACTGGACGAAAGGGTATCAGCAATGGAAGATGAAATGAATGAAATGAAGTGAGAAGGGAAGTTTAGAGAAAAAAGAATAAAAAGAAAAGAGCAAAGCCTCCAAGAAATATGGGACTAAGTGAAAAGACCAAATCTACGTCTGATTGGTGTACCTGAAAGTGACGGGGAGAATGGAACCAGGCCAACATTCAGATTCAGGAAATACAGAGAACACCACAAAGATACTCCTCGAGAAGAGCAACTCCAAGACACATAATTGTCAGATTCACAAAAGTTGAAATGAAGGAAAAAATGTTAAGGGCAGCCAGAGAGAAAGGTCGGGTTACCCTCCAAGGGAAGCCCATCAGACTAACAGCGGATCTTTCAGCAGAAACTCTACAAGCCACAAGAGAGTGGGGGCCAATATTCAACATTCTTCAACAGAAGAATTTTCAACCCAGAATTTCATATCCAGCCAAACGAAGCTTCATAAGTGAAGGAGAAATAAAATCCTTTACAGACAAGCAAATGCTGAGAGATTTTGTCACCACCAGGCCTGCCCTAAAAGAGCTCCTGAAGGAAGCGCTAAACATGGAAAGGAACAACCGGAACCAGCCACTGCAAAATCATGCCAAAATGTAAAGACCATTGAGACTAGGAAGAAACTGCATCAACTAATGAGCAAAATAACCAGCTAACATCATAATAATGGGATCAAATTCACACATAACAATATTAACCTTAAATGTAAATGGACTAAATGCTCCAATTAAAAGACACAGACTGGCAAATTGGATAAAGAGTCAAGACCCATCAGCCTGCTGTATTCAGGAAACCCATCTTATATGCAGAGACACACATAGGCTCAAAATAATAGGATGGAGGAAGATCTACCAAGCAAATGGAAAACAAAAAAAGGCAGGGGTTGCAATCCTAGTCTCTGATAAAAGAGACTTTAAACCAACAAAGATCAAAAGAGACAAAGAAGGCCATTACATAATGGTAAAGGGATCAATTCAACAAGAAGAGCTAACTATCCTAAATATATATGCACCCAATACAGGAGCACCCAGATTCATAAAGCAAGTCCTGAGTGACCTACAAAGAGACTTAGACTCCCACGCAATAATAATGGGAGACTTTAACACCCCACTGTCAACATTAGACAGATCGAGACAGAAAGTCAACAAGGATACCCAGGAATTGAACTCAGCTCTGCACCAAGCAGACCTAATAGACATCTACAGAACTCTCCACCCCAAATCAATAGAATATACATTTTTTTCAGCACCACACCACACCTATTCCAAAATTGACCACATAGTTGGAAGTAAAGCACTCCTCAGCAAATGTAAAAGAACAGAAATTATAACAAACTGTCTGTCAGACCACAGTGCAATCAAACTAGAACTCAAGATTAAGAATCTCACTCAAAACTGCTCAACTACATGGAAACTGAACAACCTGCTCCTGAATGACTACTGGGTAAAGAACGAAATGAAGGCAGAAATAAAGATGTTCTTTGAAACCAACGAGAACAAAGACACAACATACCAGAATCTCTGGGACGCATTCAAAGCAATGTGTAGAGGGAAATTTATAGCACTAAATGCCCACAAGAGAAAGCAGGAAAGATGTAAAATTGACACCCTAACATCACAATTAAAAGAACTAGAAAAGCAAGAGCAAACACATTCAAAAGCTAGCAGAAGGCAAGAAATAACTAAAATCAGAGCAGAACTGAAGGAAATAGAGACACAAAAAACCCTTCAAAAAATTAATGAATCCAGGAGCTGGTTTTTTGAAAGGATCAACAAAATTGATAGACCGCTAGCAAGACTAATAAAGAAAAAAAGAGAGAAGAATCAAATAGATGCAATAAAAAATGATAAAGGGGATATCACCACCGATCCCACAGAACTACAAACTACCATCAGAGAATACTACAAACACCTCTATGCAAATAAAATAGAAAATCTAGAAGAAATGGATAAATTCCTTGACACATACACTCTCCCAAGACTAAACCAGGAAGAAGTTGAATCTCTGAATAGACCAATAACAGGATCTGAAATTGTGGCAATAATCAATAGCTTACCAACCAAAAAGAGTCCAGGACTAGATGGATTCACAGCCGAATTCTACCAGAGGTACAAGGAGGAACTGGTACCATTCCTTCTGAAACTATTCCAATCAATAGAAAAAGAGGGAATACTCCCTAATTCATTTTATGAGGCCAGCATCATCCTGATACCAAAGCCTGGCAGAGACACAACCAAAAAGGAGAATTTTAGACCAATATCCTTGATGAACATTGATGCAAAAATCCTCAATAAAATACTGGCAAACCGAATCCAGCAGCACATCAAAAAGTTTATCCACAATGATCAAGTGGGCTTCATCCCTGGGATGCAAGGCTGGTTCAATATACGCAAATCAATAAATGTAATCCAGCATATAAACAGGGCCAAAGACAAAAACCACATGATTATCTCAGTAGATGCAGAAAAGGCCTTTGACAAAATTCAACAATCCTTCATGCTAAAAACTCTCAATAAATTAGGTATTGATGGGATGTATCTCAAAATAATAAGAGCTATCTATGACAAACCCACAGCCAATATCATACCGAATGGGCAAAAACTGGAAGCATTCCCTTTGAAAACTGGCACAAGACAGGGATGCCCTCTCTCACCACTCCTATTCAACATAGTGTTGGAAGTTCTGGCCAGGGCAATTAGGCAGGAGAAGGAAATAAAGAGTATTCAATTAGGAAAAGAGGAAGTCAAATTGTCCCTGTTTGCAGATGACATGACTGTATATCTAGAAAACTCCATTGTCTCAGCCCAAAATCTCCTTAAGCTGATAAGCAACTTCAGCAAAGTCTCAGGATACAAAATCAATGTACAAAAATCACAAGCATTCTCATACACCAATAACAGACAAACACAGAGCCAAATCATGAGTGAGCTCCCATTCACAATTGCTTCAAAGAGAATAAAATACCTAGGAATCCAACTTACAAGGGACGCGAAGGACCTCTTCAAGGACAACTACAAACCACTGCTCAATGAAATAAAACAGGACACAAACAAATGGAAGAACATTCCATGCTCATGGGTAGGAAGAATCAATATCGTGAAAATGGCCATACTGCCCAAGGTAATTTATAGATTCAATGCCATCCCCAACAAGCTACCAATGACTTTCTTCACAGAATTGGAAAAAACTACTTTAAAGTTCATATGGAACCAAAAAAGAGTCCACATCAACAAGTCAATCCTAAGCCAAAAGAACAAAGCTGGAGGCATCATGCCACCTGACTTCAATCTATACTACAAGGTTACAGTAACCAAAACAGCATGGTACTGGTACCAAAACAGAGATATAGATCAATGGAACAGAACAGAGCCCTCAGAAATAACGCCACATATCTACAACTATCTGATCTTTGACAAACCTGAGAAAAACAAGCAATGGGGAAAGGATTCCCTATTTAATAAATGGTGCTGGGAAAACTGGCTAGCCATATGTAGAAAGCTGAAACTGGATCCCTTCCTTACACCTTCTACAAAAATTAATTCAGGATGGACTAAAGACTTAAATCGTAGACCTAAAACTATAAAAACCCTAGAAGAAAACCTAGGCATTACCATTCAGGACATAGGCATGGGCTAGGACTTCATGTCTAAAACACCAAAAGCAATGGCAACAAAAACCAAAATTGACAAATGGCATCTCATTAAACTAAAGAGCTTCTGCACAGCAAAAGAAACTACCATCAGAGTGAACAGGCAACCTACAAAATGGGAGAAAATTTTTGCAACCTACTCATCTGACAAAGGGCTAATATCCAGAATCTACAATGAACTCAAACAAATTTACAAGAAAAAAACAAACAACCCCATCAAAAAGTGGGCGAAGGACATGAACAGACACTTCTCAAAAGAAGACATTTATGCAGCCAAAAAACACATGAAAAAATGCTCATGATCACTGGCCATCAGAGAAATGCAAATCAAAACAAAATGAGATACCATTTCACACCGGTTAGAATGCTGATCATTAAAAAGTCAGGAAACAACAGGTGCTGGAGAGGATGTGGAGAAATAGGAACACTTTTACAGTGTTGGTGGGACTGTAAGCTAGTTCAACCATTGTGGAAGTCAGTGTGGCGATTCCTCAGGGATCTAGAACTAGAAATACCAATTGACCCAGCCATCCCATTACTGGGTATATACCCAAAGGACTATAAATCATGCTGCTATTAGGACACATGCACACGTATATTTATTGCGGCACTATGCACAATAGCAAAGACTTGGAACCAACCCAAATGTCCAACAACGATAGACTGGATTAAGAAAATGTGGCACATATACAGCATGGAATACTATGCAGCCATAAAAAATGATGAGTTCATGTCCTTTGTAGGGACATGGATGAAATTGGAAATCATCATTCTCAGTAAACTTTCGCAAGGACAAAAAACCAAACACCGCATGTTCTCACTCATAGATGGGAATTGAACAATGAGAACACGTGGACACAGGAAGGGGAACATCTCACTCTGGGGACTGTTGTGGGGTGGGGGGAGGGGGGAGGGATAACATTAGGAGATATACCTAATGTTAAATGACGAGTTAATGGGTGCAGCACACCAGCATGGCACATGTATACATATGTAACTAACCTGCACATTGTGCACATGTACCCTAAAACTTAAAGTATAA*TAATAATAAAATAAAATAAAAACATTACAC****AAGTAAAAGAA*GCC**AGTGGCAGAAGGCCACAGGCCACATGAACCCAGTTATATGCAATGTCCATCAAATC

**Clone 57; PA3; SpIRE(97/622)-57: Filled site spans: chr6:80820688-80826243**

Empty Site:

TGTAGTATTATAAATAAAAAATATCTCTGATGAATGTGCTTCTCTTATTATTCCACTTCTCTCTTAAACCCTTTCTCTAAGTCTTGGTTGCCTCTTGTAGCAGTGAAGAACCTGTACTCT

AGAGTCAGTTGACCTGCGTT**AAAATTTTGACTT**TTCTATT

TTTCATCTGTTTGACCTGGGAAAAATTACTTAATTGTTGTGTAGATCACAATAGTACCTCCTCCATGGTAGTTAGTTGAAATAATTGATTAATAACATTTACACTATTCCTGGTATACAG

Filled Site:

GTCTTGGTTGCCTCTTGTAGCAGTGAAGAACCTGTACTCTAGAGTCAGTTGACCTGCGTT**AAAATTTTGACTT**CTTGAATAGGAACAGCTCCGGTCTACAGCTCCCAGCATGAGCGACGCAGAAAACAGGTGATTTCGGCATTTCTATCTGAGCTTTGAAGAGAGCAGTGGTTCTCCCAGGACGCAGCTGGAGATCTGAGAACAGGCAGACTGCCTCCTCAAGTGGGTCCCTGACCCCTGACCCCCAAGCAGCCTAACTGGGAGGCACCCCCCAGCAGGGGCAGACTGACACCTCACATGGCCAGGTACTCCAACAGACCTGCAGCTGAGGGTCCTCTCTGTTAGAAGGAAAACTAACAAACAGAAAGGACATCCACACCAAAAACCCATTTGTACATCACCATCATCAAAGACCAAAAGTAGATAAAACCACACAGATGGGGAAAAAACAGAGCAGAAAAACTGGAAATTCTAAAAAGCAGAGTGCCTCTCCTCCTCCAAAGGAACACAGTTCCTCACCAGCAACGGAACAAAGCTGGACGGAGAATGACTTTGACGAGCTGAGAGAAGAAGGCTTCAGATGATGAAATTACTCCGAGCTACGGGAGGACATTCAAACCAAAGGCAAAGAAGTTGAAAACTTTGAAAAAAATTTAGAAGAATGTATAACTAGAATAATCAATACCGAGAAGTGCTTAAAGGAGCGGATGGAGCTGAAAACCAAGGCTCGAGAACGATGTGAAGAATGCAGAAGCCTCAGGAGCTGATGTGATCAACTGGAAGAAAGGATATCAGCGATGGAAGATGAAATGAATGAAATGAAGCGAGAAGGGAAGTTCAGAGAAAAAAGAATAAAAAGAAACAAACAAAGCCTCCAAGAAATATGAGACTATGTGAAAAGACCAAATCTATGTCTGATTGGTGTACCTGAAAGTGACGGGGAGAATGGAACCAAGTTGGAAAACACTCTGCAGGATATTATCCAGGAGAACTTCCCCAATCTAGCAAGGCAGGCCAACATTCAGATTCAGGAAATACAGAGAACGCCACAAAGATACTCCTCGAGAAGAGCAACTCCAAGACACATAATTGTCAGATTCACCAAAGTTGAAATGAAGGAAAAAATGTTAAGGGCAGCCAGAGAGAAAGGTCGGGTTACCCTCAAAGGGAAGCCCATCAGACTAACAGCGGATCTCTTGGCAGAAACTCTACAAGCCAGAAGAGAGTGGGGGCCAATATTCAACATTCTTCAAGAGAAGAATTTTCAACCCAGAATTTCATATCCAGCCAAACTAAGCTTCATAAGTGAAGGAGAAATAAAATCCTTTACAGACAAGCAAATGCTGAGAGATTTTGTCACCACCAGGCCTGCCCTAAAAGAGCTCCTGAGGGAAGCGCTAAACATGGAAAGGAACAACCAGTACCAGCCGCTGAAAAAGCATGCCAAAATGTAAAGACCATCGAGACTAGGAAGAAACTGCATCAACTAACGAGCAAAATAACCAGCTAACATCATAATGACAGGATCAAATTCACACATAACAATATTAACTTTAAATGTAAATGGACTAAATGCTCCAATTAAAAGACACAGACTGGCAAATTGGATAAAGAGTCACGACCCATCAGTGTGCTGTATTCAGGAAACCCATCTCATGGGCAGAGACACACATAAGCTCAAAATAAAAGGATGGAGGAAGATCTACCAAGCAAATGGAAAACAAAAAAATGCAGGGATTGCAATCCTAGTCTCTGAAAAAACAGACTTTAAACCAACAAAGATCAAAAGAGACAAAGAAGGCCATTACTTAATGGTAAAGGGATCAATTCAACAAGAAGAGCTAACTGTCCTAAATATACATGCCCCCAATACAGGAGCACCCAGATTCATAAAGCAAGTCCTGAGTGACCTACAAAGAGACTTAGACTCCCACACAATAATAATTGGAGACCTTAACACCCCACTGTCAACATTAGACAGATCAACGAGACAGAAAGTCAACAAGGATACCCAGGAATTGAACTCAGCTCTGCACCAAGCAGACCTAATAGACATCTACAGAACTCTCCACTCCAAATCAACAGAATATACATTTTCTTCAGCACCACACCACACCTATTCCAAAACTGACCACATACTTGGAAGTAAAGCTCTCGTCAGCAAATGTAAAAGAACAGAAATTATAACAAACTATCTCTCAGACCACAGTGCAATCAAACTAGAACTCAGGATTAAGAATCTCACTCAAAACTGCTCAACTACATGGAAACTGAACAACCTGCTCCTGAATGACTACTGGGTACATAACGAAATGAAGGCAGAAATAAAGATGTTCTTTGAAACCAACGAGAACAAAGATACAATGTACCAGAATCTCTGGGATGCATTCAAAGCAGTGTGTAGAGGGAAATTTATAGCACTAAATGCCCACAAGAGAAAGCAGGAAAGATCCAAAATTGACACCCTAACATCACAATTAAAAGAACTAGAAAAGCGAGAGCAAACACATTCAAAAGCTAGCAGAAGGCAAGAAATAACTAAAATCAGAGCAGAACTGAAGGAAATAGACACAAAAAACACTTCAAAAAATTAACGAATCCAGGAGCTGGTTTTTTGAAAGGATCAACAAAATTGATAGACCACTAGCAAGACTAATAAGGAAAAAAAAGAGAGAAGAATCAAATAGACACAATAAAAAATGATAAAGGGGATATCACCACTGATCCCACAGAAATACAAACTACCATCAGAGAATACTACAAACACCTCGACCAATAACAGGAGCTGAAATTGTGGCAATAATCAATAGCTTACCAACGAAAAAGAGTCCAGGACCAGATGGATTCACAGCCGAATTCTACCAGAGGTACAAGGAGGAACTAGTACCATTCCTTCTGAAACTATTGCAATCAATAGAAAAAGAGGGAATCCTCCCTAACTCATTTTATGAGGCCAGCATCACCCTGGTACCAAAGCTGGGCAGAGACACAACCAAAAAAGAGAATTTTAGACCAATATTCTTGATGAACATTGATGCAAAAATCCTCAATAAAATACTGGCAAACCGAATCCAGCAGCACATCAAAAAGCTTATCCACCATGATCAAGTGGGCTTCATCCCTGGGATGCAAGGCTGGTTCAATATACGCAAATCAATAAATGTAATCCAGCATATAAACAGAACCAAAGACAAAAACCACATGTTTATCTCAATAGATGCAGAAAAGGCCTTTGACAAAATTCAACAGCCCTTCATGCTAAAAACTCTCAATAAGTTAGGTATTGATCAGACGTATCTCAAAATAATAAGAGCTATCTATGACAAACCCACAGCCAATATCATACTGAATGGGCAAAAACTGGAAGCATTCCCTTTGAAAACTGGCACAAGACAGGGATGCCCTCTCTCACCACTCCTATTCAACATAGTGTTGGAAGTTCTGGCCAGAGCAATTAGGCAGGAGAAGGAAATAAAGGTTATTCAATTAGGAAAAGAGGAAGTCAAATTGTCCCTGTTTGCAGACGACATGATTGATTGTATATCTAGAAAACCCCATTGTCTCAGCCCAAAATCTCCTTAAGCTGATAAGCAACTTCACCAAAGTCTCAGGATACAAAATCAATGTACAAAAATCACAAGCATTCTTATACACCAATAACAGACAAACAGAGAGCCAAATCATGAGTGAACTCCCATTCACAATTGCTTCAAAGAGAATAAAATACCTAGGAATCCAACTTACAAGGATGTGAAGGACCTCTTCAAGGAGAACTACAAACCACTGCTCAATGAAATAAAAGAGGATACAAACAAACGGAAGAACATTCCATGCTCATGGGTAGGAAAAATCAATATCGTGAAAATGGCCATACTGCCCAAGGTAATTTACAGATTCAATGCCATCCCCATCAAGCTACCAATGACTTTCTTCACAGAAGTGGAAAAAACGACTTTAAAGTTCATATGGAACCAAAAAACAGCCCACATCACCAAGTCAATCCTGAGCCAAAAGAACAAAGCTGGAGGCATCACACTACCTGACTTCAAACTATACTACAAGGCTACAGTAACCAAAACAGCATGGTACTGGTACCAAAACAGAGATATAGATCAATGGAACAGAACAGAGCCCTCACAAATAACGCTGCATATCTACAACTATCTGATCTTTGACAAATCTGAGAAAAACAAGCAATGGGGAAAGGATTCCCTATTTAATAAATGGTGCTGGGAAAACTGGCTAGCCATATGTAGAAAGATGAAACTGGATCCCTTCCTTACACCTTATACAAAAATCAATTCAAGATGGATTAAAGACTTAAACGTTAGACCTAAAACCATAAAAACCCTAGAAGAAAACCTAGGCATTACCATTCAGGACATAGGCATGGGCAAGGACTTCATGTCTAAAACACCAAAAGCAATGGCAACAAAAGCCAAAATTGACAAATGGGATCTAATTAAACTAAAGAGCTTCTGCACAGCAAAAGAAACTACCATCAGAGTGAACAGGCAACCTACAAAATGGGAGAAAATTTTCACAACCTACTCATCTGACAAAGGGCTAATATCCAGAATCTACAATGAACTCCAACAAATTTACAAGAAAAAAACAACCCCATCAAAAAGTGGGCATAGGACATGAACAGACACTTCTCAAAAGAAGACATTTATGCAGCCAAAAAACACAAGAAAAAATGCTCACCATCACAGGCCATCAGAGAAATGCAAATCAAAACCATAATGAGATACCATCTCACACCAGTTAGAATGGCAATCATTAAAAAGTCAGGAAATAACAGGTGCTGGAGAGGATGTGGAGAAATAGGAACACATTGACACTGTTGGTGGGACTGTAAACTAGTTCAACCATTGTGGAAGTCAGTGTGGCGATTCCTCAGGGATCTAGAACTAGAAATACCTTTTGACCCAGCCATCCCATTACTGGGTATATACCCAAAGGACTATAAATCATGCTGCTATAAAGACACATGCACACGTATGTTTATTGCGGCATTATTCACAATAGCAAAGACTTGGAACCAACCCAGATGTCCAACAATGATAGACTGGGTTAAGAAAATGTGGCACATATACACCATGGAATACTAGCAGCCATAAAAAATGATGAGTTCATGTCCTTTGTAGGGACATGGATGAAATTGGAAATCATCATTCTCAATAAACTATTGCAAGAACAAAAAACCAAACACTGCATATTCTCACTCATAGGTGGGAATTGAACAATGAGGACACATGGACACAGGAAGGGGAACATCACACTCTGGGTACTGTTGTGGGGTGGGGAGAGCGGGGAGGGATAGCACTGGGAGATATACCTAATGCTAGATGACGAGTTAGTGGGTGCAGCGCACCAGCATGGCACATGTATACATATGTAACTAACCTGCACATTGTGCACATGTACCCTAAAACTTAAAGTATAAT*AATAATAATAATAAAAGAAAAA****AAAA*TTTTGACTT**TTCTATTTTTCATCTGTTTGACCTGGGAAAAATTACTTAATTGTTGTGTAGATCACAATAGTACCTCCTCC

**Clone 58; PA3; SpIRE(97/622)-58: Filled site spans: chr6:74484391-74490993**

Empty Site:

GACATTTTTTACTTTTGCTTTTCTGAGGTTTACAAATAATATACCTAGGTATGGGGGATTTTTTTGGCATCAATTTTTCTTGGTGTAGTCCAACCTTCCTGAATAACTGGTTTGGTATCT

GACATT**AATTTGGGGGCAAAATC**CTCAAGCATTATTGTTT

CAAATTTTTTTTTCTTTTCCTCTTTCTTCCCTTCTTCTATTCTGTTATGCATATGTTATACCTTTTGTATACTGTCCTTGAATATTCTGTTATTTTCAGTCTTTTTTTCTATTTGCTTTT

Filled Site:

GACATTTTTTACTTTTGCTTTTCTGAGGTTTACAAATAATATACCTAGGTATGGGGGATTTTTTTGGCATCAATTTTTCTTGGTGTAGTCCAACCTTCCTGAATAACTGGTTTGGTATCTGACATT**AATTTGGG..CAAAATC**GGGGAGGAGCCAAGATGGCCGAATAGGAACAGCTCCGGTCTACATCTCCCAGCGTGAGCAACGCAGAAGACGGGTGATTTCTGCATTTCCATCTGAGCTTTGAAGAGAGAAGTGGTTCTCCCGGCACGCAGCTGGAGATCTGAGAATGGGCAGACTGACTACTCAAGTGGGTCCCTGACCCCTGATCCCCGAGCAGTCTAAATGGGAGGCACCCCCCAGCAGGGGCAGACTGACACCTCACATGGCTGGGTACTCCAACAGACCTGCAGCTGAGGGTCCTGTCTGTTAGAAGGAAAACTAACAAACAGAAAGGACATCCACACCAAAAACCCATCTACATCACCATCATCAAAGACCAAAAGTAGATAAAACCGTAAAGGTGGGGAAAAAACAGAGCAGAAAAACTGGAAACTCTAAAAAGCAGAGTGCCTCTCCTCCTCCAAAAGAATGCAGTTCCTCACCAGCAATGGAGCAAAGCTGGATGGAGAATGACTTTGATGAGCTGAGAGAAGAAGGCTTCAGACAATCAAATTACTCCGAGCTACAGGAGGGAATTCAAACCAAAGGCAAAGAAGTTGAAAACTTTGGAAAAAATTTAGAAGAATGTATAACTAGAATAACCAATACGGAGAAGTGCTTAAAGGAGCTGATGGAACTGAAAACCAAGGCTCGAGAACTACGTGAAGAATGCAGAAGCCTCAGGAGCCCATGCAATCAACTGGAAGAAAGGGTATCAGTGATGGAAGATGAAATGAATGAAATGAAGCAAGAAGGGAAGTTTAGAGAAAAAAGAATAAAAAGAAACGAACAAAGCCTCCAAGAAATATGGGACTATGTAAAAAGACCAAATCTACGTCTGATTGGTGTACCTGAAAGTGATGGGGAGAATGGAACCAAGTTGGAAAACACCCTGCAGGATATTATCCAGGAGAACTTCCCCAATCTAGCAAGGCAGGCCAACATTCAGAGTCAGGAAATACAGAGAACGCCACAAAGATACTCCCCGAGAAGAGCAACTCCAAGACACATAATTGTCAAATTCACCAAAGTTGAAATCAAGGAAAAAATGTTAAGGGCAGCCAGAGAGAAAGGTCGGGTTACCCTCAAAGGGAAGCCCATCAGACTAACTGCGGATCTCTTGGCAGAAACTCTACAAGCCAGAAGAGAGTGGGGGCCAATATTCAACATTCTTAAAGAAAAAAATTTCAACCCAGAATTTCATATCCAGCCAAACTAAGCTACATAAGTGAAGGAGAAATAAAATACTTTACAGACAAGCAAATGCTGAGAGATTTTGTCACCACCAGGCCTGCCCTAAAAGAGCTCCTGAAGGAAGCGCTAAACATGGAAAGGAACAACCGGTACCAGCCGCTGCAAAATCATGCCAAAATGTAAAGACCATCGAGACTAGGAAGAAACTGCATCAACTAATGAGCAAAATCACCAGCTAACATCATAATGACAGGATCAAATTCACACACAACAATATTAACTTGAAATGTTAATGGACTAAATGCTCCAATTAAAATACACAGACTGGCAAATTGGATAAAGATTCAAGACCCATCAGTGTGCTGTATTCAGGAAACCCATCTCACGTGCAGAGACACACATAGACTCAAAATAAAAGGATGGAGGAAGATCTACCAAGCAAATGGAAAACAAAAAAAGGCAGGGGTTGCAATCCTAGTCTCTGATAAAACAGACTTTAAACCAACAAAGATCAAAAGAGACAAAGAAGGCCATTACATAATGGTAAAGGGATCAATTCAAGAAGAAGAGCTAACTATCCTAAATATATATGCACCCAATACAGGAGCACCCAGATTCATAAAGCAAGTCCTGAGTGACCTACAAAGAGACTTAGACTCCCACACATTAATAATGGGAGACTTTAACACCCCACTGTCAACATTAGACAGATCAACGAGACAGAAAGTCAACAAGGATACCCAGGAATTGAACTCAGCTCTACACCAAGTGGACCTAATAGATATCTACAGAACTCTCCACCCAAATCAACAGAATATACATTTTTTTCAGCACCACACCTATTCCAAAATTGACCACATACTTGGGAGTAAAGCTCTCCTCAGCAAATGTAAAAGAACAGAAATTATAACAAACTTTCTCTGAGACCACAGTGCAATCAAACTTGAACTCAGGATTAAGAATCTCACTCAAAACCGCTCAACTACATGGAAACTGAATAACCTGCTCCTGAATGATTACTGGGTACATAACGAAATGAAGGCAGAAATAAGGATGTTCTTTGAAACCATCGAGAACAAAGACAAAACAAACCAGAATCTCTGGGACGCATTCAAAGCAGTGTGTAGAGGGAAATTTATAGCACTAAATGCCCACAAGAGAAAGCAGGGAAGATCCAAAATTGACACCCTAACATCACAATTAAAAGAACTAGAAAAGCAAGAGCAAACACATTCAAAAGCTAGCAGAAGGCAAGAAGTAACTAAAATCAGAGCAGAACTGAAGGAAATAGAGACACAAAAAACCCTTCAAAAAATTAATGAATCCAGGAGCTGGTTTTTAGAAAGGATCAACAAACTTGATAGACCACTAGCAACACTAATAAAGAAAAAAAGAGAGAAGAATCAAATAGACGCAATAAAAAATGATAAAGGGGATATCACCACCAATCCCACAGAAATACAAACTACCATCAGAGAATACTACAAACACCTCTACTCAAATAAACTAGAAAATCTAGAAGAAATGGATAAATTCCTCAACACACACACTCTCCCAAGACTAAACCAGGAAGAAGTTGAATCTCTGAATAGACCAATAACAGGAGATGAAATTGTGGCAATAATCAGTATCTTACCAACCAAAAAGAGTCCAGGACCAGATTGATTCACAGCCCAATTCTACCAGAGGTACAAGGAGGAACTGGTACCATTCCTTCTGAAACTATTCCAACCAATAGAAAAAGAGGGAATCCTCCCTAACTCATTTTATGAGGCCAGCATCATCCTGATACCAAAGCCTGGCACAGACACAACAAAAAAAGAGAATTTTAGACCAATATCCTTGATGAACATTGATGCAAAAATCCTCAATAAAATACTGGCAAACTGAATCCAGCAGCACATCAAAAAGCTTATCCACCATGATCAAGTGGGCTTCATCCCTGGGATGCAAGGCTGGTTCAATATATGCAAATCAATAAATGTAATCCAGCATATAGACAGAGCCAAAGACAAAAACCACATGATTATCTCAATAGATGCAGAAAAGGCCTTTGACAAAATTCAACAACCCTTCATGCTAAAAACTCTCAATAAATTCGGTATTGATGGGATGTATCTCAAAATAATAAGAGCTATCTATGACAAACCCACAGCCAGTATCATACTGAATGGGCAAAAACTGGAAGCATTCCCTTTGAAAACTGGCACAAGACAGGGATGTCCTCTCTCACCACTCCTATTCAACATAGTGTTGGAAGTTCTGGCCAGGGCAATTAGGCAGGAGAAGGAAATAAAAGGTATTCAATTAGGAAAAGAGGAAGTCAAATTGTCCCTCTTTGCAGACGACATGATTGTATATCTACAAAACCCCATTGTCTCAGCCCAAAATCTCCTTAAGCTGATAAGCAACTTCAGCAAAGTCTCAGGATACAAAATCAATGTACAAAAATGACAAGCATTCTTATACACCAACAACAGACAAACAGAGAGCCAAATCATGAGTGATTTTCCATTCACAATTGCTTCAAAGAGAATAAAATACCTAGGAATCCAACTTACAAGGGATGTGAAGGACCTCTTCAAGGAGAACTACAAACCACTGTTCAATGAAATAAAAGACGATACAAACAAATGGAAGAACATTCCATGCTCATGGGCAGGAAGAATCAATATCGTGAAAATGGCCATACTGCCCAAGGTAATTTACAGATTCAATGCCATCCCCATCAAGCTACCAATGACTTTCTTCACAGAATTGGATAAAACTACTTTAAAGTTCATATGGAACCAAAAAAGAGCCCACATCTCCAAGTCAATCCTAAGCCAAAAGAACAAAGCTGTAGGCATTATGCTACCTGACTACAAATTATAGTACAAGTCTACAGTAACCAAAACAGCACGGTACTGGTACCAAAACAGAGATATAGATCAATGGAACAGAACAGAGCCCTCAGAAATAATGCTGCATGTCTACAACTATCTGATCTTTGACAAACCTGAGAAAAACAAGCAATGGGGAAAGGATTCCCTATTTAACAAATGGTCAGCCCCCCGCCTGGCTAGCCGCCCCATCCAGGAGGTGAGGGGCGCCTCTGCCCGGCCGCCCCTACTGGGAAGTGAGGAGCCCCTCTGCCCAGCCAGCTGCCCCGTCCGGGAGGGAGGTGGGGGGGTCAGCCCCCCGCCCGGCCAGCTGCCTCATCCGGGAGGTGAGGGGTGCCTCTGCCTGGCCACCCCTACTGGGAAGTGAGGAGCCCCTGTGCCCGGCCACCACCCTGTCTGGGAGGTGTACACAACAGCTCATTGAGAACGGGCCATGATGACAATGGTGGTTTTGTGGAATAGAAAGGGGGGAAAGGTGGGGAAAAGATTGAGAAATCGGATGGTTGCCATGTTTGTGTAGAAAGAAGTAGACATGGGAGACTTTTCATTTTGTTCTGTACTAAGAAAAATTCTTCTGCCTTGGGATCCTGTTGATCTGTGACCTTACCCCCAACCCTGTGCTCTCTGAAACATGTGCTGTGTCCACTCAGGGTTAAATGGATTAAGGGTGATGCAAGATGTGCTTTGTTAAACAGATGCTTGAAGGCAGCATGCTCCTTAAGAGTCATCACCACTCCCTAATCTCAAGTACCCAGGGACACAAACACTGCGGAAGGCCGCAGGGTCCTCTGCCTAGGAAAACCAGAGACCTTTGTTCACTTGTTTATCTGCTGACCTTCCCTCCACTATTGTCCTATGAGGCTGCCAAATCCCCCTCTGCGAGAAACACCCAAGAATGATCAATAAGAAAACAAACAAACAAAAACAAAACAAAACAAAACAAACAAACAAAACAAAACAAACAAATGGTGCTGGGAAAACTGGCTAGCCATATGTAGAAAGCTGAAACTGGATCCCTTCCTTACACCTTAAACAAGAATCAATTCAAGACGGATTAAAGACTTAAACATTAGACCTAAAACCATAAAAACCCTAGAAGAAAACCTAGGCACTACCATTCAGGACATAGGCATGGGCAAGGACTTCAGGTCTAAAACACCAAAAGCAATGGCAACGAAAACCAAAATTGACAAATGGGATCTAATTAAACTAAAGAGCTTCTGCACAGCAAAAGAAACTACCATCAGAGTGAACAGGCAACCCACAAAATGGGAGAAAATTTTTGCAACCTACTTGTCTGACAAAGGGCTAATATCCAGAATCTACAATGAACTCAAACAAATTTACAAGAAAACAACAAAAAACCCCATCAAAAAGTGGGCAAAGGACATGAACAGACACTTCTCAAAAGAAGACATTTATGCAGCCAAAAAACACATGAAAAAAATGCTCACCATCACTGGCCATCAGAAAAATGCAAATCAAAACCACAATGAGATACCATCTCACACCAGTTAGAATGGCAATCATTAAAAAAGTCAGGAAACAACAGGTGCTGGAGAGGATGTGGAGAAATAGGAACACTTTGACACTGTTGGTGGGACTGTAAACTAGTTCAACCATTGTGGAAGTCAGTGTGGCGATTCCTCAAGGATCTAGAACTAGAAATACCATTTGACCCAGCCATCCCATTACTGGGTATATGCCCAAAGGACTATAAATCATGCTGCTATAAAGACACATGCATACGTATGTTTATTGCAGCATTATTCACAATAGCAAAGACTTGGAACCAACCCAAAAGTCCAACAATGACAGACTGGATTAAGAAAATGTGGCACATATACACCATGGAATACTATGCAGCCATAAAAAATGATGAGTTCATGTCCTTTATAGGGACATGGATGAAATTGGAAATCATCATTCTCAGTAAACTATCGCAAGAACAAAAAACCAAACACCGCATATTCTCACTAATAGGTGGGAATTGAACAATGAGAACACATGGACACAGGAAGGGGGACATCACACTCTGGGGACTGTTGTGGGGTGGAGGGAGGGGGGAGGGATAGCACTGGGAGATATACCTAATGCTAGATGACGAGTTAGTGGGTGCAGTGCACCAGCATGGCACATGTATACATATTTAACTAACCTGCACATTGTGCACATGTACCCTAAAACTTTAAGTATAAT*AATAATAATTAAAAA****AA*TTTGGGGGCAAAATC**CTCAAGCATTATTGTTTCAAATTTTTTTTTCTTTTCCTCTTTCTTCCCTTCTTCTATTCTGTTATGCATATGTTATACCTTTTGTATACTGTCCTTGAATATTCTGTTATTTTCAGTCTTTTTTTCTATTTGCTTTT

**Clone 59; PA3; SpIRE(97/622)-59: Filled site spans: chr6:117996247-118002735**

Empty Site:

TACATTTATAAATTTTAAGGTAAAAATCTCAAAAACAAACTTTAGTGTTAAGATTCTGCAGTGACTTTAGCACAAACTTTTGAAATCCTAGGGTACTAGATTAGTACAAAAGGAAAAACT

TGCACAATTGTGC**TAAATAAGAATTTTCT**CAAGGTCATAT

CGACCCTTTCTCAGGTGACTGTTTGCTATCAGAGATAATTTTCAGACATATGCAAAAACTTAACCTTTTTAATGATATGAGAATTTCTAACCTGTCTGGAAGAAAAGGCATAAGAGATCA

Filled Site:

TGAAATCCTAGGGTACTAGATTAGTACAAAAGGAAAAACTTGCACAATTGTGC**TAAATAAGAATTTCTT**GAGTGGCAGCCAAGGTGGCCGAATAGGAACAGCTCCGGTCTACAGCTCCCAGCCTGAGTGATGCAAAAGACAGGTGATTTCTGCATTTCCGTCTGAGCTTTGAAGAGAGCAGTGCTTCTCCCAGCATGCAGCTGGAGATCTGAGAACGGGCAGACTGCCTCCTCAAGTGGGTCCCTGACCCCTGATCCCTGAGCAGCCTAACTGGGAGGCACCCCCCAGTAGGGGCAGACTGACACCTCACACGGCCGGGTACTCCTCTGAGACAAAACTTCCAGAGGAACAATCAGACAGCAGCATTCGCGGTTCATGAAAATCTGCTGTTCTGCAGCCACAGCTGCTGATACCCAGGCACACAGGGTCTGGAGTGGACCTCTAGCAAACTCCAACAGACCTGCAGCTGAGGGTCCTGTGTGTTAGAAGGAAAACTAACAAACAGAAAGGACATCCACACCAAAAACCCATCTGTACATCACCATCATCAAAGTAGATAAAACCACAAAGATGGGGAAAAAACAGAGCAGAAAAACTGGAAACTCTAAAAAGCAGAGTGCCTCTCCTCCTCCAAAGGAACACAGTTCCTCACCAGCAACGAAACAAAGCTGGACGGAGAATGACTTTGACGAGTTGAGAGAAGAAGGCTTCAGACGATCAAACTACTCCGAGCTACAGGAGGAAATTCAAACCAAAGGCAAAGAAGTTAAAAACTTTGAAAAAAATTTAGATGAATGTATAACTAGAATAACCAATACAGAGAAGTGCTTAAAGGAGCTGATGGAGCTGAAAGCCAAGGCTCGAGAACTACGTGAAGAATGCAGAAGCCTCAGCAGCTGATGCGATCAACTGGAAGAAAGGGTATCAGTGATGGAAGATGAAATGAATGAAATGAAGCAAGAAGGGAAGTTTAGAGAAAAAAGAATAAAAAGAAACAAACAAAGCCTCCAAGAAATATGGGACTATGTGAAAAGATCAAATCTACGTCTGATTGGTGTACCTGAAAGTGACTGGGAGAATGGAACCAAGTTGGAAAACACTCTGCAGGATATTATCCAGGAGAACTTCCCCCATCTAGCAAGGCAGGCCAACATTCAGATTCAGGAAATACAGAGAACGCCACAAAGATACTCCTCGAGAAGAGCAACTCCAAGACACATAACTGTCAGATTCACCAAAGTTGAAATGAAGGAAAAAATGTTAAGGGCAGCCAGAGAGAAATGTCAGGTTACCCACAAAGGGAAGCCCATCAGACTAACAGTGGATCTCTCAGCAGAAACTCTACAAGCCGGAAGAGAGTGGGGGCCAATATTCAACATTCTTAAAGAAAAGAATTTTCAACCCAGAATTTCATATCCAGCCAAGCTAAGCTTCATAACTGAAGGAGAAAGAAAATCCTTTACAGACAAGCAAATGCTGAGAGATTTTGTCACCACCAGGCCTGCCCTAAAACAGCTCCTGAAGGAAGTACTAAACATGGAAAGGAACAACCGGTACCAGCCACTGCAAAATCATACCAAATTGTAAAGACCATCAAGGCTAGGAAGAAACTGCATCAACTAACGAGCAAAATAACCAGCTAACATCATAATGACAGGATCAAATTCACACACAACAATATTAACTTTAAATGTAAATGGACTAAATGCTCCAATTAAAAGACACAGACTGGCAAATTGGATAAAGAGTCAAGACCCATCAGTGTGCTGTATTCAGGAAACCCATCTCACGTGCAGAGACACACATAGGCTCAAAATAAAAGGATGGAGGAAGATCTACCAAGCAAATGGAAAACAAAAAAAGGCAGGGGTTGCAATCCTATTCTCTGATAAAACAGACTTTAAACCAACAAAGATCAAAAGAGACAAAGAAGGCCATTACATAATGGTAAAGGGATCAATTCAACAAGAAGAGCTAACTATCCTAAATATATATGCACCCAATACAGGAGCACCCAGATTCATAAAGCAAGTCCTGAGTGACCTACAAAGAGACTTAGACTCCCACACAATAATAATGGGAGACTTTAACACCCCACTGTCAACATTAGACAGATCAACGAGACAGAAAGTTAACAAGGATACCCAGGAATTGAACTCAGCTCTGCACCAAGTGGACCTAATAGACATCTAAAGAACTCTCCACCCCAAATCAACAGAATATACATTTTTTTCAGCACCACACCACACCTATTCCAAAATTGACCACATACTTGGAAGTAAAGCTGTCCTCAGCAAATGTAAAAGATCAGAAATTATAACAAACTATCTCTCAGACCACAGTGCAATCCAACTAGAACTCAGGATTCAGAAACTCACTCAAAAACTACATGGAAACTGAACAACCTGCTCCTGAATGACTACTGGATACATAAGGAAATGAAGGCAGAAATAAAGATGTTCTTTGAAACCAATGAGAACAAAGACACAACAGACCAGAATCTCTGGGACACATTCAAAGCAGTGTGTAGAGGGAAATTTATAGCACTATATGCCCACAAGAGAAAGCAGGAAAGATCCAAAATTGACACCCTAACATCACAATTAAAAGAACTAGAAAAGCAAGAGCAAACACATTCAAAAGCTAGCAGAAGGCAAGAAATACCTAAAATCAGCTCTTCCGGTTCTAGGCACTTCGGGAGCCGCGGCTTATGGTGCAGACATGGCCAAGTCCAAGAACCACACCACACACAACCAGTCCCGAAAATGGCACAGAAATGGTATCAAGAAACCCCGATCACAAAGATACGAATCTCTTAAGGGGGTGGACCCCAAGTTCCTGAGGAACATGCGCTTTGCCAAGAAGCACAACAAAAAGGGCCTAAAGAAGATGCAGGCCAACAATGCCAAGGCCATGAGTGCACGTGCCGAGGCTATCAAGGCCCTCGTAAAGCCCAAGGAGGTTAAGCCCAAGATCCCAAAGGGAGTCAGCTGCAAGCTCGATCGACATGCCTACGTTGCCCACCCCAAGCTTGGGAAGCGTGCTCTTGCCCGTATTGCCAAGGGGCTCAGGCTGTGCCGGCCAAAGGCCAAGGCCAAGGCCAAGGATCAAACCAAGGCCCAGGCTGCAGCTCCAGCTTCAGTTCCAGCTCAGGCTCCCAAAGGTACCCAGGCCCCTACAAAGGCTTCAGAGTAGATATCTCTGCCAACATGAGGACAGAAGGACTGGTGCGACCCCCCACCCCCACCCCTGGGCTATCATCTGCATGGGGCTGGGGTCCTCCTGTGCTATTTGTACAAATAAACCTGAGGCAGGAAAAAAAAAAAAAAGAAATACCTAAAATCAGAGCAGAACTGAAGGAAATAGAGACCCAAAAAAACCCTTCAGAAAATTAATGAATCCAGGAGCTGGTTTTTTGAAAGGATCAACAAAATTGATAGACCACTAGCAAGACTAATAAAGAAGAAAAGAGAGAAGAATCAAATAGACACAATAAAAAATGATAAAGGGGATATCACCACCGATCCCACAGAAATACAAACTACCATCAGAGAATACTACAAACACCTCTACGCAAATAAACTAGAAAATCTAGAAGAAATGGATAAATTCCTCGACACATATACCCTCCCAAGACTAAACCAGGAAGAAGTTGAATCTCTGAATAGACCAATAACAGGCTCTGAAATTGTGGCAATAATCAATAGCTTACCAACCAAAAAGAGTCCAGGACCAGATGGATTCACAGCCGAATTCTACCAGAGGTACAAGGAGGAACTGGTACCATTCCTTCTGAAACTATTCCAATCAATAGAAAAAGAGGGAATCCTCCCTAACTCATTTTACGAGGCCAGCATCATCCTGATACCAAAGCCTGGCAGAGATACAACCAAAAAAGAGAATTTTAGACCAATATCCTTGATGAACATTGATGCAAAAATCCTCAATAAAATACTGGCAAACGGAATCCAGCAGCACATCAAAAAGCTTATCTACCATGATCAAGGGGGCTTCATCCCTGTGATGCAAGGCTGGTTCAATATATGCAAATCAATAAATGTAATCCAGCATATAAACAGAACCAAAGACAAAACCACATGATTATCTCAATAGATGCAGAAAAGGCCTTTGACAAAATTCAACAACGCTTCATGCTAAAAGCTCTCAATAAATTAGGCATTGATGGGACATATCTCAAAATAATAAGAGCTATCTATGACAAACCCACGGCCAATATCATACTGAATGGGCAAAAACTGGAAGCATTCCCTTTGAAAACTGGCACAAGACAGGGATTCCCTCTCTCACCACTCCTATTCAACATAGTGTTGGAAGTTCTGTCCAGGGCAATTAGGTAGGAGAAGGAAATAAAGGGTATTCGATTAGGAAAAGAGGAAGTCAAATTGTCCCTGTTTGCAGATGACATGATAGTATATCTAGAAAACCCCATTGTCTCAGCTCAAAATCTCCTTAAGCTGATAAGCAACTTCAGCTAAGTCTCAGGATACAAAATCAATGTACAAAAATCACAAGCATTCTTATACACCAGTAACAGACAAACAGAGAGCCAAATCATGAGTGAACTCCCATTCACAATTGCTTCAAAGAGAATAAAATACCTAGGAATCCAACTTACAGGGGACATGAATGACCTCTTCAAGGAGAACTACAAACCACTGCTCAATGAAATAAAAGAGGATACAAACAAATGGAAGAACATTCCATGCTCATCGGTAGGAAGAATCAATATTGTGAAAATGGCCATACTGCCCAAGGTAATTTATAGATTCAATGCCATCCCCATCAAGCTACCAATGACTTTCTTCACAGAATTGGAAAAAACTATTTTAAAGTTCATATGGAACCAAAAAAGAGCCTGCATCGCCAAGTCAATCCTAAGCCAAAAGAACAAAGCTGGAGGCATCACACTACCTGACTTCAAACTATACTTCAAGGCTACAGTAACCAAAACAGCATGGTACTTGTTTCAAAATAGAGATATAGATCAATGGAACAGAGCAGAGCCCTCAGAAATAACGCCACATATCTACAACTATCTGATCTTTGACAAACCTGACAAAAACAAGCAATGGGGAAAGGATTCCCCATTTAATAAATGGTGCTGGGAAAACTGGCTAGCCATATGTAGAAAGCTGAAACTGGATCCCTTCCTTACACCTTATACAAAAAGTAATTCAAGATGGATTAAAGACTTAAACGTCAGATCTAAAACCATAAAAATCCTAGAAGAAAACCCAGGCATTACCATTCAGGACATAGGCATGGGCAAGGACTTCATGTCTAAAACACCAAAAGCAATGGCAACAAAAGCCAAAATTGACAAATGGGATCTAATTAAACTAAAGAGCTTCTGCACAGCAAAAGAAACTACCATCAGAGTGAACAGGCAACCTACAAAATGGAAGAAAATTTTCACATCCTACTTATCTGACAAAGGGCTAATATCCAGAATGTACAATGAACTCAAACAAATTTACAAGAAAAAAACAAACAACCCCATCAAAAAGTGGGCGAAGGATATGAACAGACGCTTCTCAAAAGAAGACATTTATGCAGCCAAAAAACATATGAAAAAATGCTCACTATCACTGGCCATCAGAGAAATGCAAATCAAAACCACAATGAGATACCATCTCACACCAGTTAGAATGGCAATCATTAAAAAGTCAGGAAACAACAGGTGCTGGAGAGGATGTGGAGAAATAGGAACACTTTTACACTGTTGGTGGGACTGTAAACTAGTTCAACCATTGTGGAAGTCAGTGTGGCGATTCCTCAGGGATCTAGAACTAGAAATACCATTTGACCCAGCCATCCCATTACTGGGTATATACCCAAAGGACTATAAATCATGCTGCTATAAAGACACATGCACACGTATGTTTATTGCGGCACTATTCACAATAGCAAAGACTTGGAACCAACCCAAATGTCCAACATTGATAGACTGGATTAAGAAAATGTGGCACATATACACCATGGAATACTATGCAGCCATAAAAAATGAAGAGTTTGTGTCCTTTGTAGGGACATGGATGAAATTGGAAATCATCATTCTCAGCATACTATCGCAAGGACAAAAAACCAAACACCGCATGTTCTCACTCATAGGTGGGAACTGAACAATGAGAACACATGGACACAGGAAGGGGAACGTCAAACTCTGGGGATTGTTGTGGGGTAGGGGGAGGGGGAGGGATAGCATTAGGAGATATACCTAATGTTAAATGACGAGTTAATGGGTGCAGCACACCAGCATGGCACATGTATACATATGTAACTAACCTGCACATTGTGCACATGTACCCTAAAACTTAAAGTATAA*TAATAATAATAAAATTTAAAAAAAGAAGATAAAAAAAGAA*TTTCTTTCTTTCTAGTT**TAAATAATAATTTTTT**CAAGGTCATATCGACCCTTTCTCAGGTGACTGTTTGCTATCAGAGATAATTTTCAGACATATG

**Clone 61; PA3; SpIRE(97/622)-61: Filled site spans: chr7:87238596-87244258**

Empty Site:

GGAAAGCAGACTTTGTTAAGTCTACTTGTGAAAATAGAATTGCTAGGTATAATGATAAGTATATTTTTAAGGGGTTTTAAAATTTTTGCTAAATTATCCATCTGGAAGGACCCTTTACCA

CCAGTGATTG**AGAGGCC**GGGCGCGGTGGCTCACGCCTGTA

ATCCCAGCACTTTGGGAGGCCGAGGCGGGCGGATCACGAGGTCAGGAGATCGAGACCATCCCGGCTAAAACGGTGAAACCCCGTCTCTACTAAAAATACAAAAAAATTAGCCAGGCGTAG

Filled Site:

AATTTTTGCTAAATTATCCATCTGGAAGGACCCTTTACCACCAGTGATTG**AAAGGCC**AAACTCGGAGGAGGAGCCAAGATGGCCGAATAGGAACAGCTCCGGTCTACAGCTCCTAATGTGAGCGACACAGAAGACGAGTGATTTCTGCATTTCCATCTGAGCTTTGAAGAGAGCAGTGGTTCTCCCAGCACGCAGCTGGAGATCTGAGAACGGGCAGACTGCCTCCTCAAGTGGGTCCCTGACCCCTGACCCCCGAGCAGCCTAACTGGGAGGCACCCCCCAGCAGGGGCACACTGACACCTCACACGGCCGGCCGGGTACTCCAACAGACCTGCAGCTGAGGGTCCTGTCTGTTAGAAGGAAAACTAACAAACAGAAAGGACATCCACAACAAAAACCCATCTGTACATCACCATCATCAAAGACCAAAAGTAGATAAAACCACAAAGATGGGGAAAAAACAGAGCAGAAAAACTGGAAACTCTAAAAAGCAGAGCGCCTCTCTTCCTCCAAAGGAACGCAGTTCCTTATCAGCAACAGAACAAAGCTGGGCGGAGAATGACTTTGATGAGCTGAGAGAAGAAGGCTTCAGACGATCAAATTACTCCGAGCTATGGGAGGACATTCAAACCAAAGGCAAAGAAGTTGAAAACTTTGAAAAAAATTTAGAGGAATGTATAACTAGAATAACCAATACAGAGAAGTGCTTAAAGGAGCTGATGGAGCTGAAAACCAAGGCTCGAGAACTACGTGAAGAATGCAGAAGCCTCAGGAGCCGATGTGATAAACTGGAAGAAAGGGTATCAGTGATGGAAGTTGAAAGGAATGAAATGAAGCGAGAAGGGAAGTTTAGAGAAAAAAGAATAAAAAGAAACGAGCAAAGCCTCCAAGAAATATGGGACTATGTGAAAAGACCAAATCTACGTCTGATTGGTGTACCTGAAAGTGACGGGGAGAATGGAACCAAGTTGGAAAACACTCTGCAGGATATTATCCAGGAGAACTTCCCCAATCTAGCAAGGCAGGCCAACATTCAGATTCAGGAAATACAGAGAATGCCACAAAGATACTCCTCGAGAGGAGCAACACCAAGACACATAATTGTCAGATTCACCAAAGATGAAATGAAGGAAAAAATGTTAAGGGCAGCCAGAGAGAAAGGTCGGGTTACCCTCAAAGGGAAGCCCATCAGACTAACAGCAATTCTCTCAGTAGAAACTCTACAAGCCAGAAGAGAGTGGGGGCCAATATTCAACCTTCTTAAAGAAAAGAATTTTCAACCCAGAATTTCATATCCAGCCAAACCAAGCTTCATAAGTGAAGGAGAAATAAAATACTTTACAGACAAGCAAATGCTGAGAGATTTTGTCACCACCAGGCCTGCCCTAAAAGAGCTCCTGAAGGAAGCGCTAAACATGGAAAGGAACAACCGGTACCAGCCACTGCAAAATCATGCCAAAATGTAAAGACCATCGAGACTAGGAAGAAACTGCATCAACTAACGAGCAAAATAACCAGCTAACATCAAAATGACAGGATCAAATTCACACATAACAATATTAACTTTAAACGTAAATGGACTAAATGCTCCAATTAAAAGACACAGTCTGGCAAATTGGATAAAGAGTCAAGACCCATCAGTGTGCTGTATTCAGGAAACCCATCTCACGGGCAGAGACACACATAGGCTCAAAATAAAAGGATGGAGGAAGATCTACCAAGCAAATGGAAAACAAAAAAAGGCAGGGGTTGCAATCCTAGTCTGTGATAAAACAGACTTTAAACCAACAAAGATCAAAAGAGACGAAGAAGGCCATTACATAATGGTAAAGGGATCAATTCAACAAGAAGAGCTAAGTATCCTATATATATATATGCACCCAATACAGGAGCACTGAGATTCATAAAGTAAGTCCTGAGTGACCTACAAAGAGACTTAGACTCCCACACTAAGTGGGAGACTTTACCCCACTGTCAACATTAGACAGATCAACAAGACAGAAAGTCAACAAGGATACCCAGGAATTGAACTCAGCTCTGCACCAAGTGGACCTAATAGACATCTACAGAACTCTCCACCCCAAATCAACAGAATATACATTTTTTTCATCACCACACCACACCTATTCCATAATTGACCACATACTTGGAAGGAAAGCTCTCCTCAGCAAATGTAAAAGAACAGAAATTAGAACAAACTATCTCTCAGACCACAGTGCAATCAAACTAGAACTCAGGATTAAGAATCTCACTCAAAACCGCTCAACTACATGGAAACTGAACAACCTGCTCCTGAATGACTACTGGGTACATAACGAAATGAAGGCAGAAATAAAGATGTTCTTTGAAACCAATGAGAACAAAGACACAACATACCAGAATCTCTGGGACGCATTCAAAGCAGTGTGTAGAGGGAAATTTATAGCACTAAATGCCCATAAGAGAAAGCAGGAAAGATCCAAAATTGACACCCTAACATTACAATTAAAAGAACTAGAAAAGCAAGAGCAAACACATTCAAAATCTAGCAGAAGGCAAGAAATAACTAAAATCAGAGCAGAACTGAAGGAAATAGAGACACAAAAAACCCTTCAAAAAATTAACGAATCCAGGAGCTTGTTTTTTGAAAGGATCAACAAAATTGATAGACCGCTAGCGAGACTAATAAAGAAAAAAAGAGAGAAGAATCAAATAGACGCAACAAAAAATGATAAAGGGGATATCACCACCGATCCCGCAGAAATACAAACTACCATCAGAGAATACTACAAACACCTCTACGCAAATAAACTAGAAAATCTAGAAGAAATGGATAAATTCCTTGACACATACACTCTCCCAACACTAAATGAGGAATAAGTTGAATCTCTGAATAGACCAATAACAGGAGCTGAAATTGTGGCAATAATCAATAGCTTACCAACCAAAAAGTTTCCAGGACCAGATGGATTCACAGCCGAATTCTACCAGAGGTACAAGGAGGAACTGGTACCATTCCTTCTGAAACTATTTCCATCAATAGAAAAGGAGGGAATCCTCCCTAACTCATTTTATGAGGCCAGCATCATCCTGATACCAAAGCTGGGCAGAGACACAACCTAAAAAGAGAATTTTAGACCAATATCCTTGATGAACATTGATGCAAAAATCCTCAATAAAATACTGGCAAACGGAATCCAGCAGCACATCAAAAAGCTTATCCACCATGATCAAGTGGGCTTCATCCCTGGGATGCAAGGCTGGTTCAATATATGCAAATCAATAAATGTAATCCAGCATATAAAGAGAACCAAAGACAAAACCCACATGATCATCTCAATAGATGCAGAAGAGGCCTTTGACAAAATCCAACAACCTTCATGCTAAAAAACTCTCAATAAATTAGGTATTGATGGGACATATCTCAAAATAATAAGAGCTATCTATGACAAACCCACAGCCAATATCATACTGAATGGGCAAAAACTGGAAGCATTCCCTTTGAAAACTGGCACAAGACAGGGATGCCCTCTCTCACCACTCCTATTCAAAATAGTGTTGAAAGTTCTGGCCAGGGCAATTAGGCAGGAGAAGGAAATAAAGGGTATTCAATTAGGAAAAGAGGAAGTCAAATTGTCCCTGTTTGCAGATGACATGATTGTATATCTAGAAAACCCCATTGTCTCAGCCCAAAATCTCCTTAAGCTGATAAGCAACTACAGCAAAGTCTCAGGATACAAAATCAATGTACAAAAATCACAAGCATTCTTATACACCAACAACAAACAGAGAGCCAAATCATGAGTGAACTCCCATTCACAATTGCTTCAAAGAGAATAAAATACCTAGGAATCCAACTTACAAGGGATGTGAAGGACCTCTTCAAGGAGAACTACAAACCACTGCTCAAGGAAATAAAAGAGGATACAAACAAATGAAAGAACCTTTCATGCTCATGGGTAGGAAGAATCAATATCGTGAAAATGGCCATACTGCCCAAGGTAATTTACAGATTCAATGCCATCCCCATCAAGCTACCAATGACTTTCTTCACAGAATTGGAAAAAACTACTTTAAAGTTCATATGGAACCAAAAAAGAGCCCGCATCACCAAGTCAATCCTGAGCCAAAAGAACAAAGCTGGAGACATCACACTACCTGACTTCAAACTATACTACAAGGCTACAGTAACCAAAACAGCATGGTACTGGTACCAAAACAGAGATATAGATCAATGGAACAGAACAGAGCCCTCAGAAATAACGCCGCATATCTACAACTATCTGATCTTTGACAAACCTGAGAAAAACAAGCAATGGGGAAAGGATTCCCTATTTAATAAATGGTGCTGGGAAAACTGGCTAGCCATATGTAGAAAGCTGAAACTGGATCCCTTCCTTACACCTTATACAAAAATCAATTCAAGATGGATTAAAGACTTAAACGTTAGACCTAAAACCATAAAAACCCTAGAAGAAAACCTAGGCAATACCATTCAGGACATAGGCATGGGCAAAGACTTCATGTCTAAAACACCAAAAGCAATGGCAACAAAAGCCAAAATTGACAAATGGGATCTAATTAAACTAAAGAGCTTCTGCACAGCAAAAGAAACGACCATCAGAGTGAACAGGCAACCTACAAAATGGGAGAAAATTTTTGCAACCTACTCATCTGACAAAGGGCTAATATCCAGAATCTACAATGAACTCAAACAAATTTACAAGAAAAAAACAAACAACCCCATCAAAAAGTGGGCAAAGGACATGAACAGACACTTCTCAAAAGAAGACATTTATGCAGCCAAAAGACACATGAAAAAATGCTCACCATCACTGGCCATCAGAGAAATGCAAATCAAAACCACAATGAGATACCATCTCACACCAGTTAGAATGGCAATCATTAAAAAGTCAGGAAACAACAGGTGCTGGAGAGGATGTGGAGAAATAGGAACACTTTTACACTGTTGGTGGGAATGTAAACTAGTTCAACCATTGTGGAAGTCAGCGTGGTGATTCCTCAGGGATCTAGAACTAGAAATACCATTTGACCCAGCCATCCCATTACTTGGTATATACCCAAAGGACTATAAATCATGCTGCTATAAAGACACATGCACACATATGTTTATTGTGGCATTATTGACAATAGCAAAGACTTGGAACCAACCCAAATGTCCAACAATGATTGACTGGATTAAGAAAATGTGGCACATATACACCCTGGAATACTATGCAGCCATAAAAAATGATGAGTTCATGTCCTTTGTAGGGACATGGATGAAATTGGAAATCATCATTCTCAGTAAACTATTGCAAGAACAAAAAACCAAACACCGCATATTCTCACTCATAGGTGGGAATTGAACAATGAGAACACATGGACACAGGAAGGGGAACATCACACTCTGGGGCCTTTTGTGGGGTGGGGGGAGGGGGGAGGGATAGCATTGGGAGATATACCTAATGCTAGATGACGAGTTAGTGGGTGCAGTGCACCAGCATGGCACATGTATACATATGTAACTAACCTGCACATTGTGCACACGTACCCTAAAACTTAAAGTATAAT*AATAATAAATAAATTAAAAAAAAAAA****AGA*GGCC**GGGCGCGGTGGCTCACGCCTGTAATCCCAGCACTTTGGGAGGCCGAGGCGGGCGGATCACGAGGTCAGGAGATCGAG

**Clone 63; PA3; SpIRE(97/622)-63: Filled site spans: chr7:89896800-89902458**

Empty Site:

ATATACCCAAAGGATTATAAATCATGCTGCTATAAGACACATGCACACGTATGTTTATTGTGTCACTATTCCCAATAGCAAAGACTTGGAACCAAGATAAATGTCCAACAACGATAGACT

GGATT**AAGAAAATGTGGCA**CATATACACCATGGAATACTA

TGCAGCTATAAAAAAGGATGAGTTCATGTCCTTTGTAGGGACATGGATGAAGCTGGAAACCATCATTCTCTGCAAACTATCACAAGGACATAAAACCAAACACCGCATGTTCTCACTCAT

Filled Site:

AAGACTTGGAACCAAGATAAATGTCCAACAACGATAGACTGGATT**AAGAAAATGTGGCA**GGGGAAGGAGCCAAGATGGCCGAATAGGAACAGCTCTGGTCTACAGCTCCTAGCGTGAGCGACTCAGAAGATGGGTGATTTCTGCATTTCCATCTGAGCTTTGAAGAGAGCAGTGGTTCTCCCAGCACGCAGCTGGAGATCTGAGAACGGGCAGACTGCCTCCTCAAGTGGGTCCCTGACCCCTGACCCCTGACCCCTGAGCAGCCTAACTGGGAGGCACCCCCCAGCAGGGGCATACTGACACCTCACAAGGCGGGTACTCCGACAGACCTGCAGCTGAGGGTCCTGTCTGTTAGAAGGAAAACTAACAAACAGAAAGGACATCCACACCAAAAACCCATCTGTACATCACCATCATCAAAGACCAAAAGTAGATAAAACCACAAAGATGGGGAAAAAACAGAGCAGAAAAACTGGAAACTCTAAAAAGCAGAGCGCCTCTCCTCCTCCAAAGGAACACAGTTCCTCACTAGCAACGGAACAAAGCTGGACGGAGAACGACTTTGACAAGCTGAGAGAAGAAGGCTTCAGACGATCAAATTACTCCGAGCTACGGGAGGACATTCAAACCAAAAGCAAAGAAGTTGAAAACTTTGAAAAAATTTAGAAGAATGTATAACTAGAATAACCAATACAGAGAAGCGCTTAAAGGAGCTGATGGAGCTGAAAACCAAGGCTCGAGAACTATGTGAAGAATGCAGAAGCCTTAGGAGCTGATGCGATCAACTAGAACAAAGGGTATCAGCTATGGAGGATGAAATGAATGAAATGAAGTGAGAAGGGAAGTTTGGAGAAAAAAGAATAAAAAGAAATGAACAAAGCCTCCAAGAAATATGGGACTATGTGAAAAGACCAAATCTACGTCTGATTGGTGTACCTGAAAGTGATGGGGAGAATGGAACCAAGTTGGAAAACACTCTGCAGGATATATCCAGGAGAACTTCCCCAATCTAGCAAGGTAGGCCAACATTCAGATTCAGGAAATACAGAGAACACCACAAAGATACTCCTCGAGAAGAGCAACTCCAAGACACATAATTGTCAGATTCACCAAAGTTGAAATGAAGGAAAAAATGTTAAGGGCAGCCAGAGAGAAAGGTCGGGTTACCCTCAAAGGGAAGCCCATCAGACTAACAGCTGATCTCTCGGCAGAAACTCTACAAGCCAGAAGAGAGTGGGGGCCAAAATTCAACATTCTTAAAGAAAAGAATTTTCAACCCAGAATTTCATATCCAGCCAAACTAAGCTTCATAAGTGAAGGAGAAATAAAATCCTTTACAGACAAGCAAATGCTGAGAGATTTTGTCACCACCAGGCCTGCCCTAAAAGAGCTCCTGAAGGAAGTGCAAAACATGGAAAGGAACAACCGGTACCAGCCACTGCAAAATCATGCCAAAATGTAAAGACCATCCAGACTAGGAAGAAACTGCATCAACTAACGAGCAAAATAACCAGCTAACATCATAATGATAGGATCAAATTCACACATAACAATATTAACTTTAAATGTAAACGGACTAAATGCTCCAATTAGAAGACACAGACTGGCAAATTGGATAAAGAGTCAAGACCCATCAGTGTGCTGTATTCAGGAAACCCATCTCACGTGCAGAGACACACATAGGCTCAAAATAAAAAGATGGAGGAAGATCTACCAAGCAAATGGAGAACAAAAAAAGGCAGGGGTTGCAATCCTAGTCTCTGATAAAACAGACTTTAAACCAACAAAGATCAAAAGAGACAAAGAAGGCCATTACATAATGGTAAAGGGATCAATTCAACAAGAAGAGCTAACTATCCTAAATATATATGCACCCAATACAGGAGCACCCAGATTCATAAGGCAAGTCCTAAGTGACCTACAAAGAGACTTAGACTCCCACACATTAATAATGGGAGACTTTAACATCCCACTGTCAACATTAGACAGATCAACGAGACAGAAAGTCAACAAGGATACCCAGGAATTGAACTCAGCTCTGTACCAAGCGGACCTAATAGACATCTACAGAACTCTCTACCCCAAATCAACAGAATATACATTTTTTTCAGCACCACACCACACCTATTCCAAAATTGACCACATAGTTGGAAGTAAAGCTCTCCTCAGCAAATGTAAAAGAACAGAAATTATAACAAACTATCTCTCAGACCACAGTGCAATCAAACTAGAACTCAGGATTAAGAATCTCACTCAAAACCGCTCAACTACATGGAAACTGAACAACCTGCTCCTGAATGACTACTGGGTACATAACGAAATGAAGGCAGAAATAAAGATGTTCTTTGAAACCAATGAGAACAAAGACACAACATACCAGAACCTGTGGGACGCATTCAAAGCAGTGTGTAGAGGGAAATTTATAGCACTAAATGCCCACAAGAGAAAGCAGGAAAGATGTAAAATTGACAGCCTAACATCACAATTAAAAAAACTAGAAAAGCAAGAGCAAACACATTCAAAAGCTAGCAGAAGGCAAGAAACAACTAAAATCAGAGCAGAACTGAAGGAAATAGAGACACAAAAAACCCTTCAAAAAATCAATGAATCCAGGAGCTGGTTTTTTGAAAGGATCAACAAAATTGATAGACCGCTAGCAAGACCAATAACGAAGAAAAGAGGGAAGAATCAAATAGACACAATAAAAAATGATAAAGGGGATATCACCACTGATCCCACAGAAATACAAACTACCATCAGAGAATACTACAAACACCTCTATGCAAACAAACTAGAAAATCTAGAAGAAATGGATACATTCCTCGACACATACACTCTCCCAAGACTAAACCAGGAAGAAGTTGAATCTCTGAATAGACCAATAACAGGCTCTGAAATTGTGGCAATAATCAATAGCTTACCAACCAAAAAGAGTCCAGGACCAGATGGATTCACAGCCGAATTCTACCAGAGGTACAAGGAGGAACTGGTACCATTCCTTCTGAAACTATTCCAATCAACAGAAAAAGAGGGAATCCTCCCTAACTCATTTTATGAGGCCAGCATCATCCTGATACCAAAGCCGGGCAGAGACACAACCAAAAAAGAGAATTTTAGACCAATATCCTTGATGAACATTGATGCAAAAATCCTCAATAAAATACTGGCAAACCGAATCCAGCAGCACATCAAAAAGCTTATCCACTATGATCAAGTGGGCTTCATCCCTGGGATGCAAGGCTGGTTCAAAATATGCAAATCAATAAATGTAATCCAGCATATAAACAGAACCAAAGACAAAAACCACATGATTATCTCAATAGATGCAGAAAAGGCCTTTGACAAAATTCAACAACCCTTCATGCTAAAAACTCTCAATAAATTAGGTATTGATGGGACGTATTTCAAAATAATAAGAGCTATCTATGACAAACCCACAGCCAATATCATACTGAATGGGCAAAAACTGGAAGCATTCCCTCTGAAAACGGGCACAAGACAGGGATGCCCTCTCTCACCACTCCTATTCAACATAGTGTTGGAAGTTCTGACCAGGACAATTAGGCAGGAGAAGGAAATAAAGTGTATTCAATTAGGAAAAGAGGAAGTCAAATTGTCCCTGTTTGCAGACGACATGATTGTATACCTAGAAAACCCCATTGTCTCAGCCCAAAATCTCCTTAAGCTGATAAGCAAATTCAGCAGTCTCAGGATACAAAATCAATGTACGAAAATCACAACCATTCTTATACACCAACAACAGAGAAACAGAGAGCCAAATCATGAGTGAACTCCCATTCACAATTGCTTCAAAGAGAATAAAATACCTAGCTTACAAGGGATGTGAAGGACCTCTTCCAGGAGAACTACAAAACACTGCTCAGGAAAATAAAAGAGGATACAAACAAATGGAAAAACATTCCATGCTCATGTGTAGGAAGAATCAATATCATGAAAATGGCCCTACTGCCCAAGGTAATTTATAGATTCAATGCCATCCCCATCAAGCTACCAATGCCTTTCTTCACAGAATTGGAAAAAACTACTTTAAAGTTCATATGGAACCAAAAAAGAACCCACATCACCAAGTCAATCCTAGGCCAAAAGAACAAAGCTGGAGGCATCACACTACCTGACTTCAAACTACAGTACAAGGCTACAGTAACCAAAACAGCATGGTACTGGTACCAAAACAGAGATATAGATCAATGGAACAGAACAGAGCCCTCAGAAATAACACCGCATATCTACAACTATCTGATCTTTGACAAACCTGAGAAAAAGAAGCAATGGGGAAAGGATTCCCTATTTAATAAATGGTGCTGGGAAAACTGGCTAGCCATATGTAGAAAGCTGAAACTGGATCCCTTCCTTACACCTTATACAAAAATCAATTCAAGATGTATTAAAGAATTAAACGTTAGACCTAAAACCATAAAAACCCTAGAAGAAAACCTAGGCATTACCATTCAGGACATAGGCATGGGCAAGGACTTCATGTCTAAAACACCAAAAGCAATGGCAACAAAAGCCAAAATTGACAAATGGGATCTAATTAAACTAAAGAGCTTCTGCACAGCAAAAGAAACTGCCATCAGAGTGAACAGGCAACCTACAAAATGGGAGAAAATTTTTGCAACCTACTCATCTGACAAAGGGCTAATATCCAGAATCTACGATGAACTCAAACAAATTTACAAGAAAAAAACAACCCCATCAAAAAGTGGGCAAAGTACATGAACAGACACTTCTCAAAAGAAGACATTTATGCAGCCAAAAAACACATGAAAAAATGCTCACCATCACTGGCCATCAGAGAAACGCAAATCAAAACCACAATGAGATATCATCTCACACCAGTTAGAATGGCAATCATTAAATGTCAGGAAACAACAGGTGCTGGAGAGGATGTGGAGAAATAGGAGCACTTTTACATTGTTGGTGGGACTGTAAACTAGTTCAACCATTGTGGAAGTCAGTGTGGCGATTCCTCAGGGATCTAGAACTAGAAATACCATTTGACCCAGCCATCCCATTACTGGGTATATACCCAAAGGACTATAAATCATGCTGCTATAAAGACACATGCACATGTATGTTTATTGTGGCATTATTCACAATAGCAAATACTTGGAACCAACCCAAGTGTCCAAGAATGATAGACTGGATTAAGAAAATGTGGCATATATACACCATGGAATACTATGCAGCCATAAAAAATGATGAGTTCATGTCCTTTGTAGGGACATGGATGAAATTGGAAATCATCATTCTCAGTAAACTATTGCAAGAACAAAAAACCAAACAACACATATTCTCACTTATAGGTGGGAATTGAACAATGAGAACACATGGACACAGGAAGGGGAACATCACACTCTGGGGACTGTTGTGGGGTGGGGGGAGGGGGGAGGGATAGCACTGGGAGATATATCTAATGCTAGATGACGAGTTAGTGGGTGCAGCGCACCAGTATGGCACATGTATACATATGTAACTAACCTGCACATTGTGCACATGTACCCTAAAACTTAAAGTATAAT*AATAATGATAATAATAATAATAATAATAATAAAAGTAAACCTAAAAAAA****AAGAAAA*TGTGGCA**CATATACACCATGGAATACTATGCAGCTATAAAAAAGGATGAGTTCATGTCCT

**Clone 66; PA3; SpIRE(97/622)-66: Filled site spans: chr7:83743719-83749370**

Filled Site: No clear TSD present

AGCCAAGATGGCCGAATAGGAACAGCTCCGGTCTACAGCTCCCAGCGTGAGTGACGCAGAAGACGGGTGATTTCTGCATTTCCATCTGAGGTTTGAAGAGAGCAGTGGTTCTCCGAGCACGCAGCTGGAGATCTGAGAATGGGCAGACTGCCTCCTCAAGTGGGTCCCTGACCCCTGACCCCCGAGCAGCCTAACTGGGAGGCACCCCCCAGTAGGGGCAGACTGACACCTCACACGGCCGGGTACTCCTCTGAGACAAAAATTCCAGAGGAACGATCAGACAGCAGCATTCACGGTCCATGAAATTCCGCTGTTCTGCAGCCACCGCTGCTGATAACCAGGCAAACAGGGTCTGGAGAGGACCTCTAGCAAACTCCAACAGACCTGCAGCTGAGGGTCCTGTCTGTTAGAAGGAAAACTAACAAACAGAAAGGACATCCACACCAAAAACCCATCTGTACATCACCATCATCAAAGACCAAAAGTAGATAAAACCACAAAGATGGGGAAAAAACAGAGCAGAAAAACTGGAAACTCTAAAAAGCAGAGCACCTCTCCTCCTCCAAAGGAACACAGTTCCTCACTAGCAATGGAACAAAGCTGGACAGAGAATGACTTTGACGAGTTGAGAGAAGAAGGCTTCAGATGACCAAACTACTCTGAGCTACGGGAGGAAATTCAAACCAAAGGCAAAGAAGTTAAAAACTTTGAAAAAATTTAGACAAATGTATAACTAGAATAACCAATACAGAGAAGTGCTTAAAGGAGCTGATGGAGCTGAAAGCCAAGGCTCAAGAACTACATGAAGAATGCAGAAGCCTCAGGAGCCGATGCGATCAACTGGAAGAAAGGGAATCAGTGATGGAAGATGAAATGAAATGAAGTGAGAAGGGAAGTTTAGAGAAAAAAGAATAAAAAGAAACGAACAAAGCCTCCAAGAAATATGGGACTATGGGAAAAGACCAAATCTACGTCTGATTGGTGTACCTGAAAGTGACGGGGAGAATGGAACCAAGTTGGAAAACACTCTGCAGGATATTATCTGGGAGAACTTCCCCAATCTAGCAAGGCAGGCCAACATTCAAATTCAGGAAATACAGAGAACGCCACAAAGAAACTCCTCAAGAAGAGCAACTCCAAGACACATAATTGTCAGATTCACCAAAGTTGAAATGAAGGAAAAAATGTTAAGGGCAGCCAGAGAGAAAGGTCGGGTTACCCACAAAGGGAAGCCCATCAGACTAACAGTGGATCTCTCGGCAGAAACTCTACAAGCCAGAAGAGAGTGGGGGGCAATATTCAACATTCTTAAAGAAAAGAATTTTCAACCCAGAATTTCATATCTAGCCAAACTAAGCTTCATAAGTGAAGGAGAAATAAAACACTTTACAGACAAGCAAATGCTGAGAGATTTTGTCACCACCAGGCCTGCCCTAAAAGAGCTCCTGAACGAAGCCCTAAACATGGAAAGGAACAACCAGTACCAGCCACTGCAAAATCATGCCAAATTGTAAAGACCATCGAGGCTAGGAAGAAACTCCATCAACTAACGAGCAAAATAACCAGCTAACATCATAATGACAGGATCAAATTCACACATAACAATATTAACTTTAAATGTAAATGGACTAAATGCTCCAATTAAAAGACACAGACTGGCAAATTGGATAAAGAGTCAAGACCCATCAGTGTGCTGTATTCAGGAAACCCATCTCACGTGCAGAGACACACATAGGCTCAAAATAAAAGGATGGAGGAAGATCTACCAAGCAAATGGAAAACAAAAAAAGGCAGGGGTTGCAATCCTAATCTCTGATAAAACAGACTTTAAACCAACAAAGATCAAAAGAGACAAAGAAGGCCATTACATAATGGTAAAGGGATCAATTCAACAAGAAGAGCTAACTATCCTAAATATATATGCACCCAATACAGGAGCACCCAGATTCATAAAGCAAGTCCTGAGTGACCTAAAAAGAGACTTAGACTCCCACACAATAATAATGGGAGACTTTAACACCCCACTGTTAACACTAGACCGATCAACAAGACAGAAAGTTAACAAGGATATCCAGGAATTGAAGTCAGCTCTGCACCAAGCAGACCTAATAGACATCTACAGAACTCTCCACCCCAAATCAACAGAATATACATTTTTTTCAGCACCACACCACACCTATTCCAAAATTGACCACATAGTTGGAAGTAAAGCGCTCCTCAGAAAATGTAAAAGAACAGAAATTATAACAAACTGTCTCTCAGAACACAGTGCAATCAAACTAGAACTCAGGATTAAGAAACTCACTCAAAATCGCTCAACTACATGGAAACTGAACAACCTGCTCCTGAATGACTACTGGGTACATAAGGAAATGAAGGCAGAAATAAAGATGTTCTTTGAAACCAACGAGGACAAAGACACAACATACCAGAATCTCTGGGACACATTCAAAGCAGTGTGTAGAGGGAAATTTATAGCACTAAATGCCCACAAGAGAAAGCAGGAAAGATCCAAAATTGACACCTAACATCACAATTAAAAGAACTAGAAAAGCAAGGGCAAACACATTCAAAAGCTAGCAGAAGGCAAGAAATAACTAAAACCAGAGCAGAACTGAAGGAAATAGAGACACAAAAAACCCTTCAAAAAATTAATGAATCCAGGAGCTGGTTTTTTGAAAAGATCAACAAAATTGATAGACCCCTAGCAAGACTAATAAAGAAGAAAAGAGAGAAGAATCAAATAGATGCAATAAAAAATCATAAAGGGGATATCACCACCAATCCCACAGAAATACAAACTACCATCAGAGAATACTATAAACACCTCTATGCAAATAAACTAGAAAATCTAGAAGAAATGGATAAATTCCTCGACACATACACCCTCCCAAGACTAAACCAGGAAGAAGTTGAATATCTGAATAGACCAATAACAGGCTCTGAAATTGTGGCAATAATCAATAGCTTACCAACCAAAAAGAGTCCAGGACCAGTTGGATTCACAGCCGAATTCTAACAGAGGTACAAGGAGGAACTGGTACCATTCCTTCTGAAACTATTCCAATCAATAGAAAAAGAGGGAATCCTCCCTAACTCATTTTATGAGGCCAGCATCATCCTGATACCAAAGCCAGGCAGAGACACAACCAAAAAAGAGAATTTTAGACCAATATCCCTGATGAACATTGATGCAAAAATCCTCAATAAAATACTGGCAAACCGAATCCAGCAGCACATCAAAAAGCTTATCCACCATGATCAAGTGGGCTTCATCCCTGGGATGCAAGGCTGGTTCAATACACGCAAATCAATAAATGTAATCCAGCATATAAACAGAACCAAAGACAAAAACCACATGATTATCTCAATAGATGCAGAAAAGGCCTTTGACAAAATTCAACAATGCTTCATGCTAAAAACTCTCAATAAATTAGGTATTGATGGGACGTATCTCAAAATAATACGAGCTATCTATGACAAACCCACAGCCAATATCATACTGAATGGGCAAAAACTGGAAGCATTCCCTTTGAAAACTGGCACAAGACAGGGATGCCCTCTCTCACCACCCTTATTCAACATAGTGTTGGAAGTTCTGGCCAGGGCAATTAGGCAGGAGAAGGAAATAAAGGGTATTCAATTAGGAAAAGAGGAAGTCAAATTGTCCCTGTTTGCAGAAGACATGATTGTATATCTAGAAAACCCCATTGTCTCAGCCCAAAATCTCCTTAAGCTGATAAGCAACTTCAGCAAAGTCTCAGGATACAAAATCAATGTACAAAAATCACAAGCATTCTTATACACCAACAACAGACAAACAGAGAGACAAATCATGAGTGAACTCCCATTCACAATTGCTTCAGAGAATAAAATACCTAGGAATCTAACTTACAAGGGACGTGAAGGACCTCTTCAAGGAGAACTACAAACCACTGCTCAATGAAATAAAAGAAGATACAAAGAAATGGAAGAACATTCCATGCTCATGGGTAGGAAGAATCAATATCATGAAAATGGCCATACTGCCCAAGGTAATTTATAGATTCAATGCCATCCCCATCAAGCTACCAATGACTTTCTTCACAGAATTGGAAAAAACTACTTTGAAGTACATATGGAACCAAAAAAAAGCCTGCATCGCCAAGTCAATCCTAAGCCAAAAGAACAAAGCTGGAGGCATCATGCTACCTGACTTCAAACTATACTACAAGGCTACAGTAACCAAAACAGCATGGTACTGGTACCAAAACAGAGATATAGACCAATGGAACAGAACAGAGCCCTCAGAAATAATGCCGCATACCTACAACTATGTGATCTTTGACAAACCTGACAAAAACAAGCAATGGGGAAAGGATTCCCTATTTAATACATGGTGCTGGGGAAACTGGCTAGCCATATGTAGAAAGCTGATACTGGATTCCTTCCTTACACCTTATACAAAAATTAATTCAAGATGGATTAAAGACTTAAACGTTAGACCTAAAACCATAAAAACCCTAGAAGAAAACCTAGGCATTACCATTCAGGACATAGGCATGGGCAAGGACTTCATGTCTAAAACACCAAAAGCAATGACAACAAAAGCCAAAATTGAAAAATGGGATCTAACTAAACTAAAGAGCTTCTGCACAGCAAAAGAAACTACCATCAGAATGAACAGGCAACCTACAAAATGGGAGAAAATTTTCACAACCTACTCATCTGACAAAGGGCTAATATCCAGAATCTACAATGAACTCAAACAAATTTACAAGAAAAAAACAAACAACCCCATCAAAAAGTGGGCCAAGGACATGAACAGACACTTCTTAAAAGAAGACATTTATGCAGCCAAAAAACACATGAAAAAATGCTCATCATCACTGGCCATCAGAGAAATGCAAATCAAAACCACAATGAGATACCATCTCACACCAGTTAGAATGGCAATCATTAAAAAGTCAGGAAACAACAGGTGCTGGAGAGGATGTGGAGAAATAGGAACACTTTTACTCTGTTGGTGGGACTGTAAACTAGTTCAACCACTGTGGAAGTCAGTGTGGCGATTCCTCAGGCATCTAGAACTAGAAATACCATTTGACCCAGCCATCCCATTACTGGGTATATACCCAAAGGACTATAAATCATGCTGCTATAAAGACACATGCACATGTATGTTTATTGCGGCACTATTCACAATAGCAAAGACTTGGAACCAACCCAAATGTCCAACAATGATAGACTGGATTAAGAAAATGTGGCACATATACACCATGGAATACTATGCAGCCATAAAAAATGATGAGTTCATGTCCTTTGTAGGGACATGGATGAAATTGGAAACCATCATTCTCAGTAAACTATCGCAAGGACTAAAAACAAAACACCGCATGTTCTCACTCATAGGTGGGAATTGAACAATGAGAACACATGGACACAGGAAGGGGAACATTACACTCTGGGGACTGTTGTGGGGTGGGGGGAGGGGGGAGGGATAGTATTAGGAGATATACCTAATGCTAAATGATGAACTCATCATTTAGCTGCTGGTGCAGCACACCAGCATGGCACATGTATACATATGAAACTAACCTGCACATTGTGCACATGTACCCTAAAACTTAAAGTATGATAAT*AATAAAATTAAAAAAAATAATACTGATTAAA*

**Clone 71; PA3; SpIRE(97/622)-71: Filled site spans: chr8:3270822-3276494**

Empty Site:

CAGTGGGAGGTAATCGAATCATGGGAGCAGGTCTTTCTTCTGCTGTTCTCATGATAGTGAATAAGACTCATGAGATCTCATGGTTTTATAAATGGGAGTTTCCCTGCACAAGCTCTCTCT

TCTCTGCCACCATGT**AAGATGTGACTTGC**TCCTGCTTACC

TTCTGCCATAATTGTGAGGCCTCCCCAGCCATGTGAAACTGTGAGTCAATTAAAACTCTTTTCTTTATAAATTACCCAGTCTTGGGTATGTCTTTATCAGCAGCATGAAAACAGACTAAT

Filled Site:

TGGTTTTATAAATGGGAGTTTCCCTGCACAAGCTCTCTCTTCTCTGCCACCATGT**AAGATGTGACTTGC**AGTCGGAGAAGCCAAGATGAACGAATAGGAACAGCTCCGGTCTACAGCTCCCAGCGTGAACGACACAGAAGATGGGTGATTTCTGCATTTCCATCTGAGCTTTGAAGAGAGCAGTGGTTCTCCCAGCATGCAGCTGGAGATCTGACAACAGGAAGACTGCCTCCTCAAGTGGGTCCCTGACCCCCAAGAAGCCTAACTGAGAGGCACCTCCCAGCAGGGGTAGACTGACACCTCACACGGCCGAGTACTCCAACAGACCTGCATCTGAAGGTCCTGACTGTTAGAAGGAAAACTAACAAACAGAAATGACATCCACACCAAAAACCCATCTGTACATCAACAGCATCAAAGACCAAAAGTAGATAAAACCACAAAGATGGGGAAAAAACAGAGCAGAAAAACTGGAAACTCTAAAAAGCAGAGTGCCTCTCCTCCTCCAAAGGAACACAGTTCCTCACCAGCAACGGAACAAAGCTGAACAGAGAAAGAGTTTGACAAGCTGAGAGAAGAAGGCTTCAGACAATCAAATTACTCTGAGCTACAGGAGGACATTCAAACCAAAGGCAAAGAAGTTGAAAACTATGAAAAAAGTTTAGAAGAATGTATAACTAGAATAACCAATACAGAGAAGTGCTTAAAGGAGCTGATGGAGCTGAAAACCAAAGCTCCAGAACTACGTGAAGAATGCAGAAGCCTCAGGAGCCGATGCGATCAACTGGAAGAAATGGTATCAGTGATGGAAGATGAAATGAATGAAATGAAGCGAGAAGGGAAGTTTAGAGAAAAAAGAATAAAAAGAAACGAGCAAAGCCTCCAAGAAATATGGGACTATGTGAAAAGACTGAATCTACGTCTGATTGTCGTACCTGAAAGAGACAGGGACAATGGAACCAAGTAGGAAAACACTCTGCAGGATATTATCCAGGAGAACTTCCCCAATCTAGCAAGGCAGGTCAACATTCACATTCAGGAAATACAGAGAATGCCACAAAGATACTCCTCGAGAAGAGCAACACCAAGACACATAATTGTCAGATTCACCAAAGGTGAAATGAAGGAAAAAATGTTAAGGGCAGCCAGAGAGAAAGGTTGGGTTACCCTCAAAGGGAAGCCCATCAGACTAACAGCAGATCTCTTGACAGAAACTCTACAAGCCAGAAGAGAGCGGTGGCCAATATTCAACATTCTTAAAGAAAAGAATTTTCAACCCAGAATTTCATATCCAGCCAAACTAAGCTTCATAAGTGAAGGAGAAATAAAATACTTTACAGACAAGCAAATGCTGAGAGATTTTGTCACTACCAGGCCTTCACTAACAGAGCTCCTGAAGGAAGCGCTAAACATGGAAAGGAACAAAAGGTACCAGGCACGGCAAAATCATGCCAAATTGTAAAGACCATCAAGGCTAGGAAGAAACTGCATCAACTAACGAGCAAAATAACCAGCTAACATCATAATGACAGGATCAAATTCACACATAACAATATTAACTTTAAATGTAAATGGACTAAATGCACCAATTAAAAGACACAGACTGGCAAACTGGACACAGAGTCAAGACCCATCAGTGTGCACTATTCAGGAAACACATCTCACGGGTACAGACACACATAGGCTCAAAATGAAAGGATGGAGGAAGATCTACCAAGCAAATGGAAAACAAAAAAAGGCAGGGGTTGCAATCCTAGTCTCTCATAAAACAGACTTTAAACCAACAAAGATCAAAAGAGACAAAGAAGGCCATTACATAATGGTAAAGGGATCAATTCAACAAGAAGGGCTAACTATCCTAAATATATATGCACCCAATACAGGAGCACCCAGATTCATAAAGCAAGTCCTGAGTGACCTACAAAGAGACTTAGACTCCCACACATTAATAATGGGAGACTTTAACACCCCACTGTCAACTTTAGACAGATCAACAAGACAGAAAGTCAACAAGCATACCCAGGAACTTAACTCAGCTCTGCACCAAGCGGACCTAATAGACATCTACAGAACTCTCCACCCCAAATCAACAGAATATACATTTTTTTCAGCACCACACCACACCTACTCCAAAATTGACCACATAGTTGGAAGTAAAGCACTCCTCAGCAAATATAAAAGAACAGAAATTGTAACAAACTGTCTCTCAGACCACAGTGCAATCAAACTAGAACTCAGGATTAAGAATCTCACTCAAAACCGCTCAACTACATGGAAACTGAACAACCTGCTCCTGAATGACTACTGGGTACATAACGAAATGAAGGCAGAAATAAAGATGTTCTTTGAAACCAACGAGAACAAAGACACAACATACCAGAGTCTCTGGGACACATTCAAAGCAGTGTGTAGAGGGAAATTTATAGCACTAAATGCCCACAAGAGAAAGCAGGAAAGATCCAAAATTGACACCCTAACATCACAATTAAAAGAACTAGAAAAGCAAGAGCAAACACATTCAAAAGCTAGCAGAAGGCAAGAAATAACTAAAATCAGAGTAGAACTGAAGGAAATAGAGACACAAAAAACCCTTCAAAAAATTAACGAATCCAGGAGCTGGTTTTTTGAAAGGATCCACAAAATTGGTAGACCACTAGCAAGACTAAGAAAAAAACAGAGAGGAATCAAATAGATACAATAAAAATGATAAAGGGGATATCACCACCAATCCCACAGAAATACGAACTGCCATCAGAGAATACTACAAACACCTCTATGCAAATAAACTAGAAAATCTAGAAGAAATGGATAAATTCCTGGACACATACACTCTCCCAAGACTAAACCAGGAAGAAGTTGAATCTCTGAATAGACCAAAAACAGGAGCTGAAATTGTGGCAATAATCAATAGCTTACCAACGAAAAAGAGTGCAGGACCAGATGGACTCACAACCGAATTCTACCAGAGGTACAAGGAGGAATTTGTACCATTCCTTCTGAAACTATTCCAATCAATAGAAAAAGAGGGAATCCTCCCTAACTCATTTTATGAGGCCAGCATCATCCTGATACCAAAGCCTGGCAGAGACACAACCAAAAAAGAGAATTTTAGACCAATATCCTTGATGAACATTGATGTAAAAATCCTCAATAAAATACTGGCAAACCGAATCCAGCAGCACATCAAAAAGCTTATCCACCATGATCAAGTGGGCTTCATCCCTGGGATGCAAGGCTAGTTCAATATACGCAAATCTATAAATGTAATCCAGCATATAAACAGAACCAAAGACAAAAACCACATGATTATCTCAATAGACGCAGAAAAGGCCTTTGACAAAATTCAACAACCTTTCATACTAAAAACTCTCAATAAATAAGGTATTGATGGGACGTATCTCAAAATAATAAGAGCTATCTATAACAAACCCACAGCCAATATCATACCGAATGGGCAAAAACTAGAAGCATTCCCTTTGAAGACTGGCACAAGACAGGGATGCCCTCTCTCACCACTCCTATTCAACATAGTGTTGGAAGTTCTGGCCAGGGCAATTAGGCAGGAGAAGGAAATAAAGGGTATTCAATTAGGAAAAGAGGAAGTCAAATTGTCCCTGTTTGCAGACGACATGATTGTATATCTGGAAAACCCCACTGTCTCAGCCCAAAATCTCCTTAAGCTGATAAGCAACTTCAGCAAAGTCTCAGGATACAAAATCAATGTACAAAAATCACAAGCATTCTTATACACCAACAACAGACAAACAGAGAGCCAAATCAGGAGTGAACTCCCATTCACAATTGCTTCGAAGAGAATAAAATACCTAGGAATCCAACTTACAAGGGATGTGAAGGACCTCTTCAAGGAGAACTACAAACCACTGCTCAAGGAAATAAAAGAAGATACAAACAAATGGAAGAACATTCCATGCTCATGGGTAGGAAGAATCAATATCGTGAAAATGGCCATACTGCCCAAAGTAATTTACAGATTCAATGCCATCCCCTTCAAGTTACCAATGACTTTCTTCCCAGAATTGGAAAAAACTACTTTAAAGTTCATATGGAACCAAAAAGGAGCCCACATTGCCAAGTCAGTCCTGAGCCAAAAGAACAAAGCTGGAGGCATCAGACTACCTGACTTCAAACTATGCTACAAGGCTACCGTAACCAAAACAGCATGGTACTGGTACCAAAACAGAGATATAGATCAATGGAATAGAACAGAGCCCTCAGAAATAACGCCGCATATCTACAACTATCTGATCTTTGACAAACCTGAGTAAAACAAGCAATGGGGAAAGCATTCCCTATTTAATAAATGGTGCTGGGAAAACTGGCTAACCATATGCAGAAAGCTGAAACTGGATCCCTTCCTTACACCTTATACAAAAATCAATTCAAGATGGATTAAAGACTTAAACATTAGACCTAAAACCATAAAAACCCTAGAAGAAAACCTAGGCATTACCATTCAGGACATAGGCATGGGCAAGGACTGCATGTCTCAAACACAAAAAGCAAAGGCAACAAAAGCCAAAATTGACAAATGGGATCTAATTAAACTGAAGAGCTTCTGCACAGCAAAAGAAACTACCATCAGAGTGAACAGGCAACCTACAAAATGGGAGAAAATTTTCACAACCTACTCATCTGACAAAGGGCTAATATCCACAATCTATAATGAACTCAAACAAATTTACAAGAAAAAAACAAACAACCCCATCAAAAAGTGGGCAAAGGACATGAACAGACACTTCTCAAAAGAAGACATTTATGCAGCCAAAAAACACATGAAAAAATGCTCATCATCACTGGCTATCAGAGAAATGCAAATTGAAAACCACAATGAGATACCATCTCACACCAGTTGGAATGGCAATCATTAAAAAGTGAGGAAACAACAGGTGCTGGAGAGGATGTGGAGAAATAGGAACACTTTTACACTGTTGCTGGGACTATAAACTAGTTCAACCATTGTGGAAGTCAGTGTGGACATTCGTCAGGGATCTAGAACTAGAAATACCATTTGACCCAGCCATCCCATTACTGGGTATATACCCAAAGGAATATAAATCATGCTGCTATAAAGACACATGTACACGTATGTTTATTGTGGCATTATTCACAATAGCAAAGACTTGGAACCAACCCAAATGTCCAACAATGATAGACTGGATTAAGAAAATGTGGCACATATACACCATGGAATACTATGCAGCCTTAAAAAATGATGAGTTCATGTGCTTTGTAGGGACATGGAAGAAATTGGAAATCATCATTCTCAGTAAACTATCGCAAGAACAAAAAACCAAACACCGCATATTCTCACTCAAAGGTGGGAACTGAACAATGAGAACACGTGAACACAGGAAGGGGAACATCACACTCTGGGGACTGTTGTGGGGTGGGGGGAGGGGGGAAGGACAGCATTGGGAGATATACCTATTGCTAGATGACAAGTTAGTGGGTGCAGCGCACCAGCATGGCACATGTATACATATGTAACTAACCTGTACGTTGTGCACATGTACCCTAAAACTTACAGTATAAT*AATAATAAATAAAAAATTAAAAAAAAAAAGAAAAA****AA*GATGTGACTTGC**TCCTGCTTACCTTCTGCCATAATTGTGAGGCCTCCCCAGCCATGTGAAACTGTGAGTCAATTAAAACTCT

**Clone 73; PA3; SpIRE(97/622)-73: Filled site spans: chr8:129324237-129329905**

Empty Site:

AAAAAGTTTAGGCTTTATGGACCAAAAGACAATATTTAGGATACTTATATAACAACTCATATAACAAGTTAAAGGACTTAAGTACCTTATATAGCAATTTGTATGTCTTAGAGTACTTAT

AAAATAGCCATTTT**AAAATGTAAAAATCA**TTCTTAGCTTG

CAGGATGCAGAACATAAAAACAAACAGGTAGAAAGCCAGATTTGGCCCATGAGCTGTCATTTACCAAACTCTTGTCTATCTAAATATGTGGTATTATAAAACACATATTTTGGCTCTCAG

Filled Site:

AGTACCTTATATAGCAATTTGTATGTCTTAGAGTACTTATAAAATAGCCATTTT**AAAATGTAAAAATCA**AGCCAAGATGGCCGAATAGGAACAGCTCCGGTCTACAGCTCCCAGCGTGAGCGACGCAGAAGACGGGAGATTTCTGCATTTCTATCTGAGCTTTGAAGAGAAGAGTGGTTCTCCCAGCACACAGCTGGAGATCTGAGAACGGGCTGACTGCCTCCTCAAGTGGGTCCCTGATCCCTGACCCCCGAGCAGCCTAACTGGGAGGCACCCCCCAGCAGGGGCACACTGACACCTCACACGGCCAGGTACCCCAACAGACCTGCAGCTGAGGGTCCTGTCTGTTAGAAGGAAAACTAACAAACAGAAAGGACATCCACACCAAAAACCCATCTGTACATCACCATCATCAAAGACCAAAAGTAGATAAAACCACAAAGATGGGGAAAAAACAGAGCAGAAAAACTGGAAACTCTAAAAAGCAGAGCCCCTCTCCTCCTCCAAAGGAACGCAGTTCCTCACCAGCAACGGAACAAAGCTGGACAGAGAATGACTTTGACGAGCTGAGAGAAGAAGGCTTCAGACGATCAAATTACCCCGAGCTACGGGAGGACATTCAAACCAAAGGCAAAGAAGGTGAAAACTTTGAAAAAAACTTAGAAGAATGTATAACTAGAATAACCAATACAGAGAAGTGCTTAAAGGAGCTGACGGAGCTGAAAACCAAGGCTCGAGAACTACGTGAAGAATGCAGAAGCCTCAGGAGCCGATGCGATCAACTGGAAGAAAGGGTATCAGCGATGGAAGATGAAATGAATGAAATGAAGCGAGAAGGGAAGTTTAGAGAAAAAAGAATAAAAAGGAACGAGCAAAGCCTCCAAGAAATATGGGACTATGTGAAAAGACCAAATCTACATCTGATTGGTGTACCTGAAAGTGACGGGGAGAATGGAACCAAGCTGGAAAACACTCTGCAGGATATTATCCAGGAGAACTTCCCCAATCTAGCAAGGCAGGCCAACGTTCAGATTCAGGAAATACAGAGAACACCACAAAGATACTCCTCAAGAAGAGCAACTCCAAGACACATAATTGTCAGATTCACCAAAGTTGAAATGAAGGAAAAAATGTTAAGGGCAGCCAGAGAGAAAGGTCGGGTTACCCTCAAAGGGAAGCCCATCAGACTAACAGCGGATCTCTCGGCAGAAACTCTACAAGCCAGAAGAGAGTGGGGGCCAATATTCAACATTCTTAAAGAAAAGAATTTTCAACCCAGAATTTCATATCCAGCCAAACTAAGCTTCATAAGTGAAGGAGAAATAAAATACTTTACAGAGAAGCAAATGCTGAGAGATTTTGTCACCACCAGGCCTGCCCTAAAAGAGCTCCTGAAGGAAGCGCTAAACATGGAAAGGAACAACCGATACCAGCCACGGCAAAATCATGCCAAAATGTAAAGATCATCGAGACTAGGAAGAAACTGCATCAACTAATGAGCAAAATAACCAGCTAACATCATAACGACATGATCAAATTCACACATAACAATATTAACTTTAAATGTAAATGGACTAAATGTTCCCATTCAAAGACACAGACTGGCAAATTGGATAAAGAGTCAAGACCCATCAGTGTGCTGTATTCAGGACACCCATCTCACGTGCAGAGACACACATAGGCTCAAAATAAAAGGATGGAGGAAGTTCTACCAAGCAAATGGAAAAAAAAAAAAAAAAGGCAGGGGTTGCAATCCTAGTCTCTGATAAAACAGACTTTAAACCAACAAAGATCAAAAGAGACAAAGGCCATTACATAATGGTAAAGGGATCAATTCAACAAGAAGAGCTAACTATCCTGAATATATATGCACCCAATACAGGAGCACCCAGATTCATAAAGCAAGTCCTGAGTGACCTACAAAGAGACTTAGACTCCCACACATTAATAATGGGAGACTTTAACACCCCACTGTCAACATTAGACAGATCAATGAGACAGAAAGTCAACAAGGATACCCAGGAATTGAACTCAGCTCTGCACCAAGCAGACCTAATAGACATCTATAGAACTCTCCACCCCAAATCAACAGAATATACATTTTTTTCAGCACCACACCACACCTATTCCAAAATTGACCACATACTTGGAAGTAAAGCTCTCCTCAGCAAATGTAAAAGAACAGAAGTTATAACAAACTATCTCTCAGACCACAGTGCAATCAAACTAGAACTCAGGATTAAGAATCTCACTCAAAACCGCTCAACTACATGGAAACTGACAACCTGCTCCTGAATGACTACTGGGTACATAACAAAATGAAGGCAGAAATAAAGATGTTCTTTGAAACCAAAGAGAACAAAGACAGAACATACCAGAATCTCTGGGACGCATTCAAAGCAGTGTGTAGAGGGAAATTTATAGCACTAAATGCCCACAAGAGAAAGCAGGAAAGATCCAAAATTGACACCCTAACATCACAATTAAAAGAACTAGAAAAGTAAGACCAAACACATTCAAAAGCTAGCAGAAGGCAAGAAATAACTAAAATCAGAGCAGAACTGAAGGAAATAGAGACATAAAACACCCTTCAAAAAATTAATGAATCCAGGAGCTGGTTTTTTGAAAGGATCAACAAAATTGATAAACCGCTAAAAAGACTAATAAAGAAAAAAAGAGAGAAGAATCAAATAGACGCAATAAAAAATGATAAAGGGGATATCACCACTGATCCTACAGAAATACAAACTACCATCAGAGAATACTACAAACACCTCTATGCAAATAAACTAGAAAATCTAGAAGAAATGGATAAATTCCTCAACACATACACTCTCCCAAGACTAAACCAGGAAGAAGTTGAATCTCTGAATAGACCAATAACAGGATCTGAAATTGTGGCAGTAATCAATAGCTTACCAACCAAAAAGAGTCCAGGACCAGATGGATTCACAGCCGAATTCCACCAGAGGTACAAGGTGGAACTGGTACCATTCCTTCTGAAACTATTCCAATCAATAGAAAAAGAGGGAATCCTCCCTAACTCATTTTATGAGGCCAGCATCATCCTGATACCAAAGCCGGGCAGAGACACAATCAAAAAAGAGAATTTTAGACCAATATCCTTGACGAACATTGATGCAAAAATCCTCAATAAAATACTGGCAAACCGAATCCAGCAGCACATCAAAAAGCTTATCCACTGTGATCAAGTGGGCTTCATCCCTGGGATGCAAGACTGGTTCAATATACGCAAATCAATAAATGTAATCCAGCATATAAACAGAACCAAAGTCAAAAACCACATGATTATCTCAATAGATGCAGAAAAAGCCTTTGACAAAATTCAACAACGCTTCATGCTAAAAACTCTCAATAAATTAGGTATTGATGGGACGTATTTCAAAATAATAAGAGCTATCTATGACAAACCCACAGCCAATATCATACTGAATGGGCAAAAACTGGAAGCATTCCCTTTGAAAACTGGCACAAGACAGGGATGCCCTCTCTCACCACTCCTATTCAACATAGTGTTGGAAGTTCTGGTCAGGGCAATTAGGCAGGAGAAGGAAATAAAGGGTATTCAATTAGGAAAAGAGGAAGTCAAACTGTCCCTGTTTGCAGACGACATGATTGTATATCTAGAAAACCCCACTGTCTCAGCCCAAAATCTCCTTAAGCTGATAAGCAACTTCAGCAAAGTCTCAGGATACAAAATCAATGTACAAAAATCACAAGCATTCTTATACACCAACAACAGACAAACAGAGAGCCAAATCATGAGTGAACTCCCATTCACAATTGCTTGAAAGAGAATAAAATACCTAGGAATCCAACTTACAAGGGATGTGAAGGACCTCTTCAAGGAGAACTACAAACCACTGCTCAAGGAAATAAAAGAGGATACAAACAAATGGAAGAACATTCCATGCTCATGGGTAGGAAGACTCAATATCGTGAAAATGGCCATACTGCCCAAGGTAATTTACAGATTCAATGCCATCCCCATCAAGCTACCAATGACTTTCTTCACAGAATTGGAAAAAACTACTGTAAAGTTCATATGGAACCAAAAAAGAGCCTGCATCACCAAGTCAATCCTAAGCCAAAAGAACAAAGCTGGAGGCATCACACTACCTGACTTCAAACTATACTACAAGTCTACAGTAACCAAAACAGCATGGTACTGGTACCAAAACAGAGATATAGATCAATGGAACAGAACAGAGCCCTCAGAAATAATGCCACATATCTACAACTATCTGATCTTTGACAAACCTGAGAAAAACAAGCAATGGGGAAAGGATTCCCTATTTAATAAATGGTGCTGGGAAAACTGGCTAGCCATATGTAGAAAGCTGAAACTGGATCCCTTCCTTACACCTTATACAAAAATCAATTCAAGATGGATTAAAGACTTAAACGTTAGACCTAAAACCATAAAAACTCTAGAAGAAAACCTAGGCATTACCATTCAGGACATAGGCGTGGACAAGGACTTCATGACTAAAACACCAAAAGCAATGGCAACAAAACCCAAAATTGACAAATGGGATCTAATTAAACTAAAGAGCTTCTGCACAGCAAAAGAAACTACCATCAGAGTGAACAGGCAACCTACAAAATGGGAGACAATTTTCGCAATCTACTTATCTGACAAAGGACTAATATCCAGAATCTATAATGAACTCAAACAAATTTACAAGAAAAAAACAAACAACCCCATCAAAAAGTGGGTGAAGGACATGAACAGACACTTCTCAAAAGAAGACATTTATGCAGCCAAAAAACACATGAAAAAATGCTCATCATCACTGGCCATCAGAGAAATGCAAATCAAAACCACAATGAGATACCATCTCACACCAGTTAGAATGGTGATCATTAAAAAGTCAGGAAACAACAGGTGCTGGAGAGGATGTGGAGAAATAGGAACACTTTTACACTGTTGGTGGGACTGTAAACTAGTTCAACCATTGTGGAAGTCAGTGTGGCGATTCCTCAGGGATCTAGAACTAGAAATACCATTTGACCCAGCCATCCCATTACTGGGTATATACCCAAAGGACTATAAATCATGCTGCTATAAAGACACATGCACACGTATGTTTATTGCAGCATTATTCACAATAGCAAAGACTTGGAACCAACCCAAATGTCCAACAATGATAGACTGGATTAAGAAAATGTGGCACATATACACCATGGAATACTATGCAGCCATAAAAAATGATGAGTTCATATCCTTTGTAGGGACATGGATGAAATTGGAAATCATCATTCTCAGTAAACTGTTGCAAGAACAAAAAACCAAACACATATTCTCACTCATAGGTGGGAATTGAACAATGAGAACACATGGACACAGGAAGGGGAACATCACACTCTGGGTACTGTTGTGGGGTGGGGGGAGGGGGGAGGGATAGCACTGGCAGACATACCTAATGCTAGATGATGAGTTAGTGGGTGCAGCGCACCAGCATGGTACATGTATACGTATGTAACTAACATGCACATTGTGCACATGTACCCTAAAACTTAAAGTATAA*TAATAATAAATAAATAAATAAATAAATAAATAAATAAATAAAAAGAAA****AAAATGTAAAAA*TCA**TTCTTAGCTTGCAGGATGCAGAACATAAAAACAAACAGGTAGAAAGCCAGATTT

**Clone 76; PA3; SpIRE(97/622)-76: Filled site spans: chr10:53792286-53797903**

Empty Site:

GAGAAAAATAATAGTATTTATCCTATGGGCTATTATGAGGATTAAATTAGTTAATTTTTGTAAAGCTTTTAGAACAACTCTTGCATATAATAGACACTACATAAGTGTGCAGGGGGAAAA

TCACTTCAC**GAAAGAAAGG**GGTTTGGGTGCAGTGGATCAG

GCTTATAATCCCATATACCAGGGAGGCTGAAGAGAGAGGATCCCTTGAGCCCAGAAATTGGAGGCTGCTGTGAGTCTTGTTCATGCCACTGCACTTCAGCCTGGGTGACAGAGTGAGACT

Filled Site:

TTGCATATAATAGACACTACATAAGTGTGCAGGGGGAAAATCACTTCAC**GAAAGAAAGG**GGTGGAGGAGCCAAGATGGCCCAAGAGGAACAGCTCCGGTCTACAGCTCCTAGCGTGAGCGACGCAGAAGACGGGTGATTTCTGCATTTCCATCTGAGCTTCGAAGAGAGCAGTGGTTCTCCCAGCACGCAGCTGGAGATCTGAGAACGGGCAGACTGTTTCCTCAAGTGGGTCCCTGACCCCTGACCCCCGAGCAGCCTAACTGGGAGGCACCCCTCAGCAGGAGCAGACTGACACCTCACACGGCCGGGTACTCCAACAGTCCTGCAGCTGAGGGTCCTATCTGTTAGAAGGAAAACTAACAAACAGAAAGGACATCCACACCAAAAACCCATCTGTACATCACCATCATCAAACACCAAAAGTAGATAAAACCACAAAGATGGGGAAAAAACAGAGCAGAAAAACTGGAAACTCTAAAAAGGAGAGTGCCTCTCCTCCTCCAAAGGAACGCAGTTCCTCACCAGCAATGGAACAAAGCTAGACGGAGAATGACTTTGACGAGCTGAGAGAAGAAGGCTTCAGACGATCAAATTACTCCGAGCTACAGGAGGAAATTCAAACCAAAGGCAAAGAAGTTGAAAACTTTGAAAAAAGTTTAGAAGAATGTATAACTAGAATAACCAATCCAGAGAAGTGCTTAAAGGAGCTGATGGAGCTGAAAGACAAGGCTCGAGAACTACGTGAAGAATGCAGAAGCCTCAGGAGCCGATGCGATCAACTGGAAGAAAGGGTATCAGCGATGGAAGATGAAGTGAATGAAATGAAGCGAGAAGGGAAGTTTAGAGAAAAAAGAATAAAAAGAAACGAGCAAAGCCTCCAAGAAATATGGGACTATGTGAAAAGACCAAATCTACGTCTGACTGGTGTACCTGAAAGTGACAGGGAGAATGGAACCAAGTTGGAAAACACTCTGCAGGATATTATCCAGGGGAACTTCCCCAATCTAGCAAGGCAGGCCAACATTCAGATTCAGGAAATACAGAGAATGCCACAAAGATACTCCTCGAGAAGAGCAACTCCAAGACACATAATTGTCAGATTCACCAAAGTTGAAATGAAGGAAAAAATGTTAAGGACAGCCAGAGACAAAGGTCGGGTTACCCACAAAGGGAAGCCCATCAGACTAACAGCGGATCTCTCAGCAGAAACTCTACAAGCCAGAAGAGAGTGGGGGCCAATATTCTACATTCTTAAAGAAAAGAATTTTCAACCCAGAACTTCATATCCAGCCAAACTAAGCTTCATAATTGAAGGAGAAATAAAATACTTTACAGACAAGCAAATGCTGAGAGATTTTGTCACCACCAGGCCTGCCCTAAAAGAGCTCCTGAAGGAAGCGCTAAACATGGAAAGGAACAACCGGTACCAGCTGCTGCAAAAACATGCCAAAGTGTAAAAACCATCGAGACTAGGAAGAAACTGCATGAACTAACGAGCAAAATAACCAGCTAACATCATAATGACAGGATCAAATTCACACATAACAATATTAACTTTAAATGTAAATGTGATAAATGCTCCAATTAAAAGACACAGACTGGCAAATTGGATAGAGTCAAGACCCATCAGTGAGCTGTATTCAGGAGACACATCTCACATGCAGAGACAGAGATAGGCTCAAAATAAAGGTATGGAGGAAGATCTACCAAGCAAATGGAAAAAAAAAATAGTGGTTGCAATCCTAGTCTCTGATAAAACAGACTTTAAACCAACAAAGATCAAAAGAGACAAAGAAGGGCATTACATAATGGTAAAGGGTTCAATTCAACAAGAAGAGCTAACTATCCTAAATATATATGCACCCAATACAGGAGCACCCAGATTCATAAAGCAAGTCCTGAGTGAACTACAAAGAGACTCAGACTCTCACACATTAATAATGGGAGACTTTAACACCCCACTGTCAACATTTGACAGATCAATGAGACAGAAAGTCAACAAGGATACCCAGGAATTGAACTCAGCTCTGCACCAAGCAGACCTAATAGACATCTACAGAAGTCTCCACCCCACATCAACAGAATATACATTTTTTTTCAGCACCACACCACACCTACTCCAAAATTGATCACATAATTGGAAGTAAAGCTCTCCTCAGCAAATGTAAAAGAACAGAAATTATAACAAACTATCTCTCAGACCACAGTGCAATCAAACTAGAACTCAGGATTAAGAATCTCACTCAAAACCGCTCAACTACATAGAAACTGAACAACCTGCTCCTGAATGACTACTGGGTACATAATGAAATGAAGGCAGAAACAAAGATGTTCTTTGAAACCAACGAGAACAAAGACACAACATACCAGAATCTCTGGGACGCATTCAAAGCAGTGTGTAGGGGGAAATTTATAGCACTAAATGCCCACAAGAGAAAGCAGGAAAGATCCAAAATTGACACCCTAACATCACAATTAAAAGAACTAGAAAAGCAAGAGCAAACACATTCAAAAGCTAGCAGAAGGCAAGAAGTAACTAAAATCAGAGCAGAACTGAAGGAAATAGAGACACAAAAAACCCTTCAAAAAATTAATGAATCCAGGAGCTGGTTTTTTGAAAGGATCAACAAAATTGATAGACCACTACCAAGACTAATAAAGAAAAAAAGAGAGAAGAATCAAATAGACACAATAAAAAATGATAAAGGGGATATCACCACCGATCCCACAGAAATACAAACTACCATCAGAGAATACGACAAACACCTCTACGCAAATAAACTAGAAAATCTAGAATAAATGGATAAATTCCTCGACACATACACTCTCCCAAGACTAAACCAGGAAGAAGTTGAATCTCTGAATAGACCAATAACAGGAGCTGAAATTGTGGCAATAATCAATAGCTTACCAGACAAAAAGAGTCCAGGACCAGATGGATTCACAGCCGAATTCTACCAGAGGTACAAGGAGGAACTGGTACCATTCCTTCTGAAACTATTCCAATCAACAGAAAAAGAGGGAATCCTCCCTAACTCATTTGATGAGGCCAGCATCATCCTGATACCAAAGCGGGGCAGAGACACAACCAAAAAAGAGAATTTTAGACCAATATCCTTGATGAACACTGATGCAAAAATCCTCAATAAAATACTGGCAAACCGAATCCAGCAGCACATCAAAAAGCTTATCCACCATGATCAAGTGGGCTTCATCCCTGGGATGCAAGGCTGGTTCAATATACACAAATCAATAAATGTAATCCAGCATATAAACAGAACCAAAGACAAAAACCACATGATTATCTCAATAGATGCAGAAAAGGCATTTGACAAAATTCAACAACCCTTCATGCTAAAAACTCTCAATAAATTAGGTATTGATGGGACGTATCTCAAAATAATAAGAGCTATCTATGACAAACCCACAGCCAATATCATACTGAATGGGCAAAAACTGGAAGCATTCCCTTTGAAAACGGGCACAAGACAGGGATGCCCTCTCTCACCACTCCTATTCAACATAGTGTTGGAAGTTCTGGCCAGGGCAATTAGGCAGGAGGAGGAAATAAAGGGTATTCAATTAGGAAAAGAGGAAGTCAAATTGTCCCTGTTTGCAGATAACATGATTGTATATCTAGAAAACCCCACTGTCTCAGCCCAAAATCTCCTTAAGCTGATAAGCAACTTCAGCAAAGTCTCAGGATACAAAATCAATGTACAAAACTCACAAGCATTCTTATACACCAATAACAGACAAACAGAGAGCCAAATCATGAGTGAACTCCCATTCACAATTGCTTCAAAGAGAATAAAATACCTAGGAATCCAACTTACAAGGGATGTGAAGGAACTCTTCAAGGAGAACTACAAACCACTGCTCAAGGAAATAAAAGAGGATACAAACAAATGGAAGAACATTCCATGCTCATGGGTAGGAAGAATCAATATCGTGAAAATGGCCATACTGCCCAAGGTAATTTACAGATTCAATGCCATCCCCATCAAGCTACCAATGACTTTCTTCACAGAATTGGAAAAAACTACTTTAAAGTTCATATGGAACCAAAATAGAGCCCACATCGCCAAGTCAATCCTAAGCCAAAAGAACAAAGCTGGAGGCATCACACTCCCTGACTTCAAACTATACTACAAGGCTACGGTAACCAAAACAGCATGGTACTGGTACCAAAACAGATATAGATCAATGGAACAGAACAGAGCCCTCAGAAATAACGCCGCATATCTACAACTATCTGATCTTTGACAAACCTGAGAAAAACAAGCAATGGGGAAAGGATTCCCTATTTAATAAATGGGGCTGGGAAAACTGGCTAGCCATATGGAGAAAGCTGAAACTGGATCCCTTCCTTACACCTTACACAAAAATCAATTCAAGATGGATTAGAGATTTAAACGTTAGACCTAAAACCATAAAAACCCTAGAAGAAAACCTAGGCATTACCATTCAGGACATAGGCATGGGCAAGGACTTCATGTCTAAAACACCAAAAGCAATGGCAACAAAAGCCAAAATTGACAAATGGGATCTAATTAAACTAAAGAGCTTTTGCACAGCAAAAGAAACTACCATCAGAGTGAACAGGCAACCTACAAAATGGGAGAAAATTTTCGCAACCTACTCATCTGACAAAGGCTAATATCCAGAATCTACAATGAACTCAAACAAATTTACAAGAAAAAAACAAACAACCCCATCAAAAAGTGGGCAAAGGATATGAACAGACACTTCTCAAAAGAAGACATTTATGCAGCCAAAAAAACACATGAAAAAATGCTCACCATCACTGGCCATCAGACAAATGCAAATCAAAACCATAATGAGATACCATCTCACACTAGTTAGAATAGCAATCATGAAAAAGTCAGGAAACAACAGGTGCTGGAGAGGATGTGGAGAAATAGGAGCACTTTTACACTGTTGGTGGGACTGTAAACTAGTTCAACCATTGTGGAAGTCAGTGTGGCGATTCCTCAGGGATCTAGAACTAGAAATACCATTTGACCCAGCCATCCCATTACTGGGTATATACCCAAAGGACTAGAAACCATGCTGCTAAAAAACACATGCACACGTATGTTTACTGTGGCACTATTCACAATAGCAAAGACTTGGAACCAACTCAAATGTCCAACAATGATAGACTGGATTAAGAAAATGTGGCACATATACACCATGGAATACTATGCAGCCACAAAAAATGATGAGTTCATGTCCTTTGTAGGGACATGGATGAAATTGGAAATCATCATTCTTAGTAAACTATCGCAAGAACAAAAAACCAAACACTGCATATTCTCACTCATAGGTGGGAATTGAACAATGAGAACACATGGACACAGGAAGGGGAACATCACACTCTGGGGACTGTTGTGGGGTGGGGGGAGGGGGGAGGGATAGCACTGGGAGATATACCTAATGCTAGATGACGAGTTAGTGGGTGCGGCTTACCAGCATGGCACATGTATACGTATGTAACTAACCTCCACATTGTGCACATGTACCCTAAAACTTAAAGTATAAT*AATAATAAATTTAAAAAAAA****GAAAGAAA*GG**GGTTTGGGTGCAGTGGATCAGGCTTATAATCCCATATACCAGG

**Clone 77; PA3; SpIRE(97/622)-77: Filled site spans: chr10:109605382-109611046**

Empty Site:

ATTTTATGTCCTTTTATTTATCAAGGTCTTAGTTGTTTATCCATTTGTACAAACTCTTGTAAGAAAAGTTTAAAAACCTTTTGACATACCTCCTGGAAAGAGTCTTCCCAGTCTGCCTTA

TAATTTGATGAAAT**AAGATATTAAA**TTTTACAAAGCAAAT

CCCTGTAGTTTTTTTCCCATTATTTCTAACTTACATATTACTCTCTGTTTTCTTTTAATTTTGCTTTTTTTCTATGTTTTCATGGTTCTCATATTTTCAATGACAACTCTGTATATTAAC

Filled Site:

TTGACATACCTCCTGGAAAGAGTCTTCCCAGTCTGCCTTATAATTTGATGAAAT**AAGATATTAAA**GGGGGAGGAGCCAAGATGGCCGAATAGGAACAGCTCCGGTCTACAGCTCCCAGCGTGAGCAATGCAGAAGAGGGGTGATTTCTGCATTTCCATCTGAGCTTTGAAGAGAGCAGTGGTTCTCCCAGCACGCAGCTAGAGATATGAGCAGCTGGAGATCTGAGAACGGGCAGACTGCCTCCTCAAGTGGGTCCCTGACCCCTGACCCCTAAGCAGCCTAAATGGGAAGCACCCGCCAGCAGGGGCAGACTGACAACTCACACGGCTGGGTACTCCAACAGACCTGCAGCTGAGGGTCCTGTCTGTTAGAAGGAAAACTAACAAACAGAAAGGACATCCACACCAAAAACCCATCTGTACATCACCATCATCAAAGACCAAAAGTAGATAAAACCACAAAGAGGGGGAAAAAACAGAACAGAAAAACTGGAAACTCTAAAAAGCAGAGCGCCTCTCCTCCTCCAAAGGAACGCAGTTCCTCACCAGCAACGGAAAAAAGCTGGATGGAGAATGACTTTGACGAGCTGAGAGAAGAAGGCTTCAGACGATCAAATTACTCTGAGCTACGGGAGGAAATTCAAACCAAAGGCAAAGAAGTTGAAAACTTTGAAAAAAGTCTAGAAGAATGTATAACTAGAATAACCAATACAGAGAAGTGCTTAAAGGAGCTGATGGAGCTGAAAACCAAGGCTCAAGAACTATGTGAAGAATGTAGAAGCCTCAGGAGCTGATGTGATCAACTGGAAGAAAGGGTATCAGCGATGGAAGATGAAGTGAATGAAATGAAGTGAGAAGGGAAGTTTAGAGAAAAAAGAATAAAAAGAAAAGAGCAAAGCCTCCAAGAAATATGGGACTATGTGAAAAGACCAAATCTACGTGTGATTGGTGTACCTGAAAGTGACGGGGAGAATGGAACCAAGTTGAAAAACACTCTGCAGGATATTATCCAGGAGAACTTCCCCAATCTAGCAAGGCAGGCCAACATTCAGATTCAGGAAATACAGAGAATGCCACAAAGATACTCCTCGAGAACAGCAACTCCAAGACACATAATTGTCAGATTCACCAAAGTTGAAATGAAGGAAAAAATGTTAAGGGCAGCCAGAGAGAAAGGTCGGGTTACCCTCAAAGGGAAGCCCATGAGACTAACAGCGGATCTCTCAGCAGAAACTCTACAAGCCAGAAGAGAGTGGGGGCCAATATTCAACATTCTTAAAGAAAAGAATTTTCAACCCAGAATTTCATATCCAGCCAAACTAAGCTTCATAAGGGAAGGAGAAATAAAATACTTTACAGACAAGCAAATGCTGAGAGATTTTGTCACCACCAGGCCTGCCCTAAAAGAGCTCCTGAAGGAAGCACTAAACATGGAAAGGAACAACTGGTACCAGCCACTGCAAAATCATGCCAAAATGTAAAGACCATCGAGACTAGGAAGAAACTGCATCAACTAACGAGCAAAATAACCAGCTAACATCATAATGACAGGATCAAATTCACACATAACAATATTAACTTGAAATGTAAATGGACTAAATGCACCAATTAAAAGGCACAGATGGGCAAATTGGATAAAGAGTCAAGACCCATCAGCGTGCTGTATTTAGGAAACCCATCTCACATGCACAGACACACATAGGCTCAAAATAAAAGGATGGAGGAAGATCTAACAAGCAAATGGAAAACAAAAAAAGGCAGGGGTTGCAATCCTAGTCTCTGATAAAACAGACTTTAAACGAACAAAGATCAAAAGAGATAAAGAAGGCCATTACACAATGGTAAAGGGATCAATTCAACAAGAAGAGCTAACTATCCTAAATATATATGCACCCAATACAGGAGCATCCAGATTCATAAAGCAAGTCCTGAGTGACCTACAAAGGGACTTAGACTCCCACACAGTAACAATGGGAGACTTTAACAGCCCACTGTCAACATTAGACAGATAAACGAGACAGAAAGTCAACAAGGATACCCAGGAATTGAACTCAGCTCTGCACCAAGTGGACCTAATACACATCTACAGAACTCTTCACCCAAAATCAACAGAATATACATTTTTTTTCAGCACCACACCACCCCTATTCCAAAATTGACCGCATAGTTGGAAGTAAAGCACTCCTCAGCAAATGTAAAAGAACAGAAATTATAACAAACTGTCTCTCAGACCACAATGCAATCAACCTAGAACTCAGGATTAAGAATCTCACTCAAAACCACTCAACTACATGGAAACTGAACAACCTGCTCCTGAATGACTACTGGGTACATAACAAAATGAAGGCAGAAATAAAGATGTTCTTTGAAACCAATGAGAACAAAGACACAACATACCAGAATCTCTGGGATGCATTCAAAGCAGTGTGTAGAGGGAAATTTATAGCACTAAATGCCCACAAGAGAAAGCAGAAAAGATCCAAAATTGACATCCTAACATCACAATTAAAAGAACTAGAAAAGCAAGAGCAAACACATTCAAAAGCTAGCAGAAGGCAAGAAATAACTAAAATCAGAGCAGAAATGAAGGAAATAGAGACACAAAAAACCCTTCAAAAAATTAATGAATCCAGGAGCTGGTTTTTTGAAAGGATCAACAAAATTGATAGACCGCTAGCAAGACTAATAAAGAAAAAAAGAGAGAAGAATCAAATAGACACAATAAAAAATGATAAAGGGGATATCACCACCGATCCCACAGAAATACAAACTACAATCAGAGAATACTACAAACACCTCTACGCAAATAAACTAGAAAATCTAGAAGAAATGGATAAATTCATCAACACATACACTCTCCCAAGACTAAACCAGGAAGAAGTTGAATCTCTGAATAGAACAATAACAAGATCTGAAATTGTGACAATAATCAATAGCTTACCAACCAAAAAGAGTCCAGGACCAGATGCATTCACAGCCGAATTCTACCAGAGGTACAAAGAGGAACTGGTACCATTCCTTCTGAAACTATTCCAATCAATAGAAAAAGAGGGAATCCTCCCTAACTCATTTTATGAGGCCAGCATCATCCTGATACCAAAGCCGGGCAGAGACACAACCAAAAAAGAGAATTTTAGACCAATATCCTTGATGAACATTGATGCAAAAATCCTCAATAAAATACTGGAAAACCCAATTCAGCAGCACATCAAAAAGCTTATCCACCATGATCAAGTGGGCTTCATCCCTGGGATGCAAGGCTGGTTCAATATACGCAAATCAATAAATGTAATCCAGCATATAAACAGAACCAAAGAAAAAAACTACATGATTATCTCACTAGATGCAGAAAAGGCCTTTCACAAAATTCAACAACCCTTCATGCTAAAAACTCTCAATAAATTAGGTATTGATGGGACATATCTCAAAATAATAAGAGCTATCTATGACAAACCCACAGCCAATATCATACTGAATGGGCAAAAACTGGAAGCATTCCCTTTGAAAACTGGCACAAGACAGGGATGCCCTCTCTCACCACTCCTATTCAACATAGTGTTAGAAGTTCTGGCCAGGGCAATTAGGCAGGAGAAGGAAATAAAAGGTATTCAATTAGGAAAAGAGGAAGTCAAATTGTCCCTGTTTGCAGATGACATGATTGTATATCTAGAAAACCCCATTGTCTCAGCCCAAAATCTCCTTAAGCTGATAAGCAACTTCAGCAAAGTCTCAGGATACAAAATCAATGTGCCAAAATCACAAGCATTCTTATACACCAACAACAGACAGAGAGCCAAATCATGAGTGAACTCCCATTCACAATTGCTTCAAAGAGAATAAAATACCTAGGAATCCAACTTACAAGGGATGTGAAGGACCTCTTCAAGGAGAACTACAAACCACTGCTCAAGGAAATAAAAGAGGATACAAACAAACGGAAAAACATTCCATGCTCATGGGTAGGAAGAATCAATATCGTGAAAATGGCCATACTATCCAAGCTAATTTACAGATTCAATGCCATTCCCATCAAGCTACCAATGACTTTCTTCACAGAATTGGAAAAAAACTACTTTAAAGTTCATATAGAACCAAAAAAGAGCCCGCATTGCCAAGTCAATCCTAAGCCAAAAGAACAAAGCTGGAGGCATCATGCTACCTGACTTCAAACTACACTACAAGGCTACAGTAACCAAAACAGCATGGTACTGGTACCAAAATAGAGATATAGATCAATGGAACAGAACAGAGCCCTCAGAAATAATGCTGCATATCTACAACTATCTGATCTTTGACAAACCTGAGAAAAACAAGCAATGGGGAAAGGATGCCCTATTTAATAAATGGTGCTGGGAAAACTGGCTAGCCATATGTAGAAAGCTGAAACTGGATCCCTTCCTTACACCTTATACAAAAATCAATTCGAGATGAATTAAAGACTTAAACATTAGACCTAAAACCATAAAAACCCTAGAAGAAAACCTAGGCATTACCATTCAGGACATAGGCATGGGCAAGTACTTCATGTCTAAAACACCAAAAGCAATGGCAACAAAAGCCAAAATTGACAAATGGGATCTAATTAAACTAAAGAGCTTCTTCACAGCAAAAGAAACTACCATCAGAGTGAACAGGCAACATACAAAATGGGAGAAAATTTTCGCAACCTACTCATCTGACAAAGGGCTAATATCCAGAATCTACAATGAACTCAAACAAATTTACAAGAAAAAAACAAACAAACCCATCAAAAAGTGGGTGAAAGACATGAACAGACACTTCTCAAAAGAAGACATTTATGCAGCCAAAAAACATATGAAAACATGCTCACCATCACTGGCCATCAGAGAAATGCAAATCAAAACCACAATGAGATACCATCTCACACCAGTTAGAATGGCAATCATTAAAAAGTCAGGAAACAACAGGTGCTGGAGAGGATGTGGAGAACTAGGAACACTTTTACACTGTTGGTGGGACTGTAAACTAGTTCAACCATTGTGGAAGTCAGTGTGGCGATTCCTCAGGGATCTAGAACTAGAAATACCATTTGACCCAGCCATCCCATTACTGGGTATATACCCAAAGGACTATAAATCATGCTGCTATAAAGACACATGCACACGTATGTTTATTGCGGCATTATTCACAATAGCAAAGACTTGGAACCAACCCAAATGTCCCACAATGATAGACTGGATTAAGAAAATGTGGCACATATACACCATGGAGTACTATGCAGCCATAAAAAATGATGAGTTCATGCCTTTGTAGGGACATGGATGAAATTGGAAATCATCATTCTCAGTAAACTATCACGAGAACAAAAAACCAAACAGTGCATATTCTCACTCATAGGAGGGAATTGAACAATGAGAACACATGGACACAGGAAGGGGAACATCACACTCTGGGGACTGTTGTGGGGTGGGGGGAGGGGGGAGGGATAGCACTGGGAGATATACCTAATGCTAGATGACGAGTTAGTGGGTGCAGCGCACCAGCATGGCACATGTATACATATGTAACTAACCTGCACATTGTGCACATGTACCCTAAAACTTAAAGTATAAT*AATAAATTTTTTAAAAA****AAGATTTTAAA***TTTTACAAAGCAAATCCCTGTAGTTTTTTTCCCATTATTTCTAACTTACATATTACTCTCTGT

**Clone 82; PA3; SpIRE(97/622)-82: Filled site spans: chr10:49246072-49251736**

Empty Site:

AGGGGAATGGAATAGATTAGTCATCAAATGGTATTTTTATTTATAAAGGAAACCTTAGATAAAAGAAGTAAATACAATTCAATAATTACTGATACTGGGACAGAGGAGGCACCTCTAAGA

TTAGCAAT**AAAAATACCACAAAG**GAAAATACTGATAAGCCA

TATAATCTGTGTCCATATATCACATATAGATTATATCAAAAATGCCACATTCAAAAATAGAAAGGACAAACTGGAAAGGAATATTTTCAACAAATATGATAAGAGGGCAATAGTCTTATT

Filled Site:

AATAATTACTGATACTGGGACAGAGGAGGCACCTCTAAGATTAGCAAT**AAAAATACCACAAAG**AGGGGGAGGAGCCAAGATGGCTGAATACGAACAGCTCCAGTCTACAGCTCCCAGCATGAGCGACGCAGAAGATGGGTGGTTTCTGCATTTCCATCTGAGCTTTGAAGAGAGCAGTGGTTCTCCCAGCATGCAGCTGGAGATCTGAGAACGGGCAGACTGCCTCCTCAAGTGGGTCCCTGACCCCTGACCCCCGAGGAGCCTAACTAGGAAGCACCCCCCAGCAGGGGCAGACTGACACCTCACACGGCTCCAACAGACCTGCAGCTGAGGGTCCTGTCTGTTAGAAGGAAAACTAACAAACAGAAAGGACATCCACACCAAAAACCCATCTGTACATCACCATCATCAAAGACCAAAAGTAGATAAAACCACAAAGATGGAGAAAAAACAGAGCAGAAAAACTGGAAACTCTAAAAAGCAGAGCACCTCTCCTCCTCCAAAGGAACGCAGTTCCTCACCAGTAACAGAACAAAGCTGGACGAAGAATGACTTTGACGAGCTGAGAGAAGAAGGCTTCAGACGATCAAATTACTCCGAGCTACAGGAGGACATTCAAACCAAAGGCAAAGAAGTTGAAAACTTTGAAAAAAATTTAGAAGAATGTATAACTAGAATAACCAATACAGAGAAGTGCTTAAAGGAGCTGATGGAGCTGAAAACCAAGGCTCGAGAACTACATAAAGAATGCAGAAGACTCAGGAGCTGATGCAATCAACTGGAAGAAAGGGTATCTGTGATAGAAGATGAAATGAATGAATGAAATGAAGCGAGAAGGGAAGTTTAGAGAAAAAAGAATAAAAAGAAACGAACAAAGCCTCCAAGAAATATGGGACTATGTGAAAAGACCAAATCTACATCTGATTGGTGTACCTGAAAGTGACAGGGAGAATAGAACCAAGTAGGAAAACACTCTGCAGGATATTATCCAGGAGAACTTCCCCAATCTAGCAAGGCAGGCCAACATTCAGATTCAGGAAATACAGAGAACGCCACAAAGATACTCCTCGAGAAGAGCAACTCCAAGACACATAATTGTCAGATTCACCAAAGTTGAAATGAAGGAAAAAATGTTAAGGGCAGCCAGAGAGAAAGGTTGGGTTACCCTCAAAGGGAAGCCCATCAGACTAACAGCGGATCTGTTGGCAGAAACTCTACAAGCCAGAAGAGAGTGGGGGCCAATATTCAACATTCTTAAAGAAAAGAATTTTCAACCCAGAATTTCATATCCAGCCAAACTAAGCTTCATAAGTGAAGGAGAAATAAAATACTTTACAGACAAGCAAATGCTGAGAGATTTTGTCACCACCAGGCCTGTTCTAAAAGAGCTCCTGAAGGAAGCACTAAACATGGAAAGGAACAACCAGTACCAGCCGCTGCAAAATCATGCCAAAATGTAAAGACCATCGAGACTAGGAAGAAACTGCATCAACTAATGAGCAAAATAACCAGCTAACATCATAATGACAGGATCAAATTCACACATAACAATATTAACTTTAAATGTAAATGGACTAAATGCTCCAATTAAAAGACACAGACTGGCAAATTGGATAAAGAGTCAAGACCCATCAGTGTGCTGTATTCAGGAAACCCATCTCACGTGCAGAGACACATATAGGCTCAAAATAAAAGGATGGAGGAAGATCTACCAAGCAAATGGAAAACAAAAAAAGGCAGGGGTTGCAATCCTAGTCTCTGATGAAACAGACTTTAAACCAACAAAGATCAAAAGAGACAAAGAAGGCCATTACATAATGGTAAAGGGATCAATTCAACAAGAAGAGCTAACTATCCTAAATATATATGCACCCAATACAGGAGCACCCAGATACATAAAGCAAGTCCTGAGTGACCTACAAAGAGACTTAGACTCCCACACATTAATAATGGGAGACTTTAACACCCCACTGTCAACATTAGACAGATCAAAGATACAGAAAGTCAACAAGGATACCCAGGAATTGAACTCAGCTCTGCACCAAGTGGACCTAATAGACATCTACAGAACTCTCCACCCCAAATCAACAGAATATACATTAGTTTCAGCACCACACCACACCTATTCCAAAATTGACCACATACTTGGAAGTAAAGCTCTCCTCAGCAAATGTAAAAGAACATAAACTATAACAAACTGTCTCTCAGACCACAGTGCAATCAAACTAGAACTCAGGATTAAGAATCTCACTCAAAACTGCTCAACTACATGGAAACTGAACAACCTGCTCCTGAATGACTACTGGGTACATAATAAAATGAAGGCAGAAATAAAGATGTTCTTTGAAACCAATGAGAACAAACACACAACATACCAGAATCTTTGGGACACATTCAAAGCAGTGTGTAGAGGGAAATTAATAGCACTAAATGCCCACAAGAGAAAGCAGGAAAGATCCAAAATTGACACCCTAACATCACAATTAAAAGAACTAGAAAAGCAAGAGCAAACACATTCAAAAGCTAGCAGAAGGCAAGAAATAACTAAAATCAGAGCAGAACTGAAGGAAATAGAGACACAAAAAACCTTTCAAAAAATTAATGAATCCAGGAGCTGATTTTTGAAAGGATCAACAAAATTGATAGATCGCTAGCAAGAAAAATAAAGAAAAAAAGAGAGAAGAATCAAATAGGTGTAATAAAAAATGATAAAGGGGATATCACCACCAATCCCACAGAAATACAAACTACCATCAGAGAATACTACAAACACCTCTACTCAAATAAACTAGAAAATCTAGAAGAAATGGATAAATTCCTTGACACATACACTCTCCCAAGACTAAACCAGGAAGAAGTTGAATCTCTGAATAGACCAATAACAGGAGCTGAAATTGTGGCAATAATCAATAGCTTGCCAATCAAAAAGAGTCCAGGACCAGATGGATTCACAGCTGAATTCTACCAGAGGTACAAGGAGGAAATGGTACCATTCTTTCTGAAACTATTCTTATCAATAGAAAAAGAGGGAATCCTCCCTAACTCATTTTATGAGGCCAGCATCATGCTGATACCAAAGCCGGGCAGAGACACAACCAAAAAAGAGAATTTTAGACCAATATCCTTGAGGAACATTGATGTAAAAATCCTCAATAAAATACTGGCAAACCGAATCCAGCAGCACATCAAAAAGCTTATCCACCATGATCAAGTGGGCTTCATCCCTGGGATGCAAGGCTGGTTCAACATATGCAAATCAATAAAGGTAATCCAGCATATAAAGAGAACCAAACACAAAAACCACATGATTATCTCAATAGATGCAGAAAAGGCCTTTGACAAAATTCAACAACTCTTCATGCTAAAAACTCTCAATAAATTAGGTATTGATGGGACGTATTTCAAAATAATAAGAGCTATCTATGACAAACCCACAGCCAATATCATACTGAATGGGCAAAAACTGGAAGCATTCCCATTGAAAACTGGCACAAGACAGGGATGCCCTCTCTTACCACTCCTATTCAACATAGTGTTGGAAGTTCTGGCCAGGGCAATTAGGCAGGAGAAGGAAATAAAGGGTATTCAATTAGGAAAAGAGGAAGTCAAATTGTCCCTGTTTGCAGATGACATGATTGTATATCTAGAAAACCCCATCGTCTCAGCCCAAAATCTCCTTAAGCTGATAAGCAACTTCAGCAAAGTCTCAGGATACAAAATCAATGTACAAAAATCACAAGCATTCTTATACACCAATAACAGACAAACAGAGAGCCAAATCATGAATGAACTCCCATTCACAATTACTTCAAAGAGAATAAAATACCTAGGAATCCAACTTACAAGGGATGTGAAGGACCTCTTCAAGGAGAACTACAAACCACTGCTCAAGGAAATAAAAGAGGATACAAACAAATGGAAGAACATTCCATGCTCATGGGTAGGAAGAATCAATACATGAAAATGGCCATACTGCCCAAGGTAATTTACAGATTCAATGCCATCCCCATCAAGCTACCAATGATTTTCTTCACAGAATTGGAAAAAACTACTTTAAAGTTCATATGGAACCAAAAAAGAGCCCGCATCACCAAGTCAATCCTAAGCCAAAAGAACAAAGCTGGAGGCATCACACTGCCTGACTTCAAACTATACTACAAGGCTACAGTAACCAAAACAGCATGGTACTGGTACCAAAACAGAGATATAGATCAATGGAACAGAACAGAGCCCTCAGAAATAATGCTGCATATCTACAACTATCTGATCTTTGACAAACCTGAGAAAAACAAGCAATGGGGAAATGATTCCCTATTTAATAAATGGTGCTGGGAAAACTGGCTAGCCATATGTAGAAAGCTGAAACTGGATCCCTTCCTTACACCTTATACAAAAATCAATTCAAGATGGATTAAAGACATAAACGTTAGACCTAAAACCATAAAAACCCTAGAAGAAAATCTAGGCTTTACCATTCAGGACATAGGCATGGGCAAGGACTTCATGTCTAAAACACCAAAAGCAATGGCAACAAAAGCCAAAATTGACAAATGGGATCTAATTAAACTAAAGAGCTTCTGCACAGCAAAAGAAACTACCATCAGAGTGAACAGGCAACCTACAAAATGGGAGAAAATTTTCACAACCTACTCATCTGACAAAGGGCTAATATCCAGAATCTACAAAGAACTCAAACAAATTTACAAGAAAAAAACAAACAACCCCATCAAAAAGTGGGCAAAGGACATGAACAGACACTTCTCAAAAGAAGACATTTATGCAGCCAAAAAACACATGAAAAAATGCTCACCATCACTGGCCATCAGAGACATGCAAATCAAAACCACAATGAGATACCATCTCACACCAGTTAGAATGGCAATCATTAAAAAATCAGGAAACAACAGGTGCTGGAGAGGATGTGAAGAAATAGGAACACTTTTACGCTGTTGGTGGGACTGTAAACTAGTTGAACCATTGTGGAAGTCAGTGTGGCGATTCCTCAGGGATCTAGAACTAGAAATACCATTTGACCCAGCCATCCCATTACTGGGTATATACCCAAAGGACTATAAATCATGCTGCTATAAAGACACATGCACACGTATGTTTATTGCGGCATTATTCACAATAGCAAAGACTTGGAACCAACCCAAATGTCCAACAATGATAGACTTGATTAAGAAAATGTGGCACATACACACCATGGAATACTATGCAGCCATAAAAAATGATGAGTTCATGTCCTTTATAGGGACATGGATGAAATTGGAAATCATCATTCTCAGTAAACTATCGCAAGAACAAAAAACCAAACACCGCATATTCTCACTCATAGGTGGGAATTGAACAATGAGAACACATGGACCCAGGAAGGGGAATATdCACACTCTGGGGACTGTTGTGGGGTGGGGGGAGTGGGGAGGGATAACATTGGGAGATATACCTAATGCTAGATGATGAGTTAGTGGGTGCAGCACACCAGCATGTCACATGTATACATATGTAACTAACTTGCACATTGTGCACATGTACCCTAAAACTTAAAGTATAAT*AATAAAAATAAATAAATAAATTAATTAATTAATTAA****AAAAATA*CCACAAAG**GAAAATACTGATAAGCCATATAATCTGTGTCCATATATCACATATAGATTATATCAAAAA

**Clone 83; PA3; SpIRE(97/622)-83**

**Filled site spans: chr11:13793733-13799387**

Empty Site:

TTTCAGTCTGATGCTTTCTTTCAAACTATCTTCCAATGTATTAATTCTCTCATTAGCTGTTAAACCCATTCACTGAATCCTTAATTTTGGTTTTGATATCCAAAATACCCACTATTGTTT

TTA**AAAATAGCTTTC**AATTCTCTGCTTAAGCTCAAGAAAT

TGCATTTTATTTCCTTCAATATCATAATCACCATTATTTAAGTGTCTATATCTGATAACTCCAATATATGAAGCCCTATCAGTTTTGCTTCTAGTAGCTTTCATTCTTATTGTTTTGTCT

Filled Site:

TTAATTTTGGTTTTGATATCCAAAATACCCACTATTGTTTTTA**AAAATAGCTTTC**GGAGTCAAGATGGCCGAATAGGAACAGCTCCGCTCTACAGCTCCCGGCCTGAGCAATGCAGAAGACGGGTGATTTCTGCATTTCCATCTGAGCTTTGAAGAGAGCAGTGGTTCTCCCAGTACGCAGCTGGAGATCTGAGAATGGGCACACTGCCTCCTCAAGTGGGTCCCTGATCCCTGATCCCTGACCCCCGAGCAGCCTAACTGGGAGGCACCCTCCAGCAGGGGCACACTGACACCTCACACTGCAGGGTACTCCAACAGACCTGCAGCTGAGGGTCCTGTCTGTTAGAAGGAAAACTAACAAACAGAAAGGACATCCACACCAAAAACCCGTCTGTACATCACCATCATCAAAGACCAAAAGTAGATAAAACCACAAAGATGGGGAAAAAACAGAGCAGAAAAACTGGAAACTCTAAAAATCAGAGCGCCTCTCCTCCTCCAAAGGAACGCAGCTCCTCACCAGCAACGGAACAAAGCTGGATGGAAAATGACTTTGATGAGCTGAGAGAAGAAGGCATCAGACGATCAAATTACTCTGAGCTACGGGAGGACATTCAAACCAAAGGCAAAAAAGTTGAAAACTTTGAAAAAAATTTAGAAGAATGTATAACTAGAATAACCAATACAGAGAAGTGCTTAAAGGAGCTGATGGAGCTGAAAACCAAGGCTCGAGAACTACCTGAAGAATGCAGAAGCCTCAGGAGCCGATGTGATCAACTGGAAGAAAGGGTATCAGCAATGGAAGATGAAATGAATGAAATGAAGCCAGAAGGAAAGTTTAGAGAAAAAAGAATAAAAAGAAACAAACAAAGCCTCCAAGAAATATGGGACTATGTGAAAAGACCAAATCTACATCTAACTGGTGTACCTGAAAGTGATGGGGAGAATGGAACCAAGTTGGAAAACACTCTGCAGGATATTATCCAGGAGAATTTCCCCAATCTAGCAAGGCAGGCCAACGTTCAGATTCAGGAAATACAGAGAACGCCACAAAGATACTCCTCGAGAAGAGCAACTCCAAGACACATAATTGTCAGATTCACCAAAGTTGAAATGAAGGAAAAAATGTTAAGGGCAGCCAGAGAGAAAAGTTGGGTTACCCTCAAAGGGAAGCCCATCAGACTAACAGCGGATCTCTCGGCAGAAACCCTACAAGCCAGAAGAGAGTGGGGGCCAATATTCAACATTCTTAAAGAAAAGAATTTTCAACCCAGAATTTCATATCCAGCCAAACTAAGCTTCATAACTGAAGGAGAAATAAAATCCTTTACAGACAAGCAAAGGCGGAGAGATTTTGTCATCACCAGGCCTGCCCTAAAAGAGCTGCTGAAGGAAGCACTAAACATGGAAAGGAACAACCGGTACCAGCCACTGCAAGATCATGCCAAAATGTAAAGACCATCGAGACTAGGAAGAAACTGCATCAACTAACGAGCAAAATCACCAGCTAACATCATAATGACAGGATCAAATTCACACATAACAATATTAACTTTAAATGTAAATGGACTAAATGCTCCAATTAAAAGGCACAGACTGGCAAATTGGATAAAGAGTCAAGACCCATCAGTGTGTTGTATTCAGGAAACCCATCTCATGTGCAGAGACATACATAGGCTCAAAATAAAGGGATGGAGGAAGATCTACCAAGCAAATGGAAAACAAAAAAAGGCAGGGGTTGCAATCCTAGTCTCTGATAAAACAGACTTTAAACCAACAAAGATCAAAAGAGACAAAGAAGGCCATTACATAATGGTAAAGGGATCAATTCAACAAGAAGAGCTAACTATCCTAAATATATATGCACCCAATAGAGGAGCACCCAGATTCATAAAGCAAGTCCTGAGTGACCTACAAAGAGACTTAGACTCCCACACATTAATAATGGGAGACTTTAACACCCCACTGTCAACATTAGACAGATCAATGAAACAGAAAGTCAACAACGATACCCAGGAATTGAACGCATCTCTGCACCAAGCAGACCTAATAGACATCTACAGAACTCTCCACTCCAAATCAACAGAATATACATTTTTTTCAGCACCACACCACACCTATTCCAAAATTGACCACATACTTGGAAGTAAAGCTCTCCTCAGCAAATGTAAAAGAACAGAAATTATAACAAACTATCTCTCAGACCACAGTGCAATCAAACTAGAACTCAGGATTAAGAATCTCACTCAAAACCGCTCAACTACATGGAAACTGAACAACCTGCTCCTGAATGACTACTGGGTACATAATGAAGTGAAGGCAGAAATAAAGATGTTCTTTGAAACCAATGAGAACAAAGACACAACATACGAGAATCTCTGGGATGCATTCAAAGCAGTGTGTAGAGGGAAATTTACACCACTAAATGCCCACAAGAGAAAGCAGGAAAGATCCAAAATTGACACCCTAACATCACAATTAAAAGAACTAGAAAAGCAAGAGCAAACACATTCAAAAGCTAGCAGAAGGCAAGAAATAACTAAAATCAGAGCAGAACTGAAGGAAATAGAGACACAAAAAAACCCTTCAAAAAATTAATGAATCCAGGAGCTGGTTTTTTGAAAGGATCAACAAAATTGATAGACCACTAGCAAGACTAATAAAGAAAAAAAGAGAGAAGAATCAAATAGATGCAATAATAAATGATAAAGGGGATATCACCACCGATCCCACAGAAATACAAACTACCATCAGAGAATACTACAAACACCTCTATGCAAATAAACTAGAAAATCTAGAGGAAATGGATAAATTCCTTGACACATACACTCTACCAAGACTAAACCAGGAAGAAGTTGAATCTCTGAATAGACCAATAACAGGATCTGAAACTGTGGCAATAATCAATAGCTTACCAACCAAAAAGAGTCCAGGACCAGATGGATTCACAGCCGAATTCTACCAGAGGTACAAGGAGGAACTGGTACCATTCCTTCTGAAACTATTCCAATCAATAGAAAAAGAGGGAATCCTCCCTAACTCATTTTATGAGGCCAGCATCATTCTGATACCAAAGCCGGGCAGAGACACAACCAAAAAAGAGAATTTTAGACCAATAACCTTGATGAACATTGATGCAAATATCCTCAATAAAATACTGGCAAACCGAATCCAGCAGCACATCAAAAAGCTTATCCACCATGATCAAGTGGGCTTCATCCCTGGGATGCAAGGCTGGTTCAAGATACGCAAATCAATAAATGTAATCCAGCATATAAACAGAGCCAAAGACAAAAACCAGATGATTATCTCAATAGATGCAGAAAAGGCCTTTGACAAAATTCAACAACCCTTCATGCTAAAAACTCTCAATAAATTAGGTACTGATGGGACATATTTCAAAATAATAAGAGCTATTTATGACAAACCCACAGCCAATATCATACTGAATGGGCAAAAACTGGAAGCATTCCCTTTGAAAACTGGCACAAGACAAGGATGCCCTCTCTCACCACTCCTATTCAACATAGTGTTGGAAGTTCTGGCCAGGGCAATTAGGCAGGAGAAGGAAATAAACGGTATTCAATTAGGAAAAGAGGAAGTCAAATTGTCCCTGTTTGCAGATGACATGATTATATATCTAGAAAACCCCATTGTCTCAGCACAAAATCTCCTTAAGCTGATAAGCAACTTCAGCAAAGTCTCAGGATACAAAATCAATGTGCAAAACTCACAAGCATTCTTATACACCAACAACAGACAGAGAGCCAAATCATGAGTGAACTCCCATTCATAATTGCTTCAAAGAGAATAAAATACCTAGGAATCCAACTTACAAGGGATGTGAAGGACCTCTTCAAGGAGAACTACAAACCACTGCTCAAGGAAATAAAAGAGGATACAAACAAATGGAAGAACATTCCATGCTCATGGGTAGGAAGAATCAATATCGTGAAAATGGCCATACTGCCCAAGGTAATTTATAGATTCAATGCCATCCCCATCAAGCTACCAATGCCTTTCTTCACAGAATTGGAAAAAACTACTTTAAAGTTCATATGGAACCAAAAAAGAGCCCGCATTGCCAAGTCAATCCTAAGCCAAAAGAACAAAGCTGGAGGCATCACACTACCTGACTTCAAACTATACTACAAGGCTACAGTAACCAAAACAGCATGGTACTGGTACCAAAACAGAGATATAGATCAATGGAACAGAACAGAGCCCTCAGAAATAATGCCGCATATCTACAACTATCTGATCTTTGACAAACCTGAGAAAAACAAGCAATGGGGAAAGGATTCCCTATTTAATAAATGGTGCTGGGAAAACTGGCTAGCCATATGTAGAAAGCTGAAACTGGATCCCTTCCTTACACCTTATACAAAAATCAATTCAAGATGGATTAAAGACTTACATGTTAGACCTAAAACCATAAAAACCCTAGAAGAAAACCTAGGCATTACCATTCAGGACATAGGCATGGGCAAGGGCTTCATGTCTAAAACACCATAAGCAATGGCAACAAAAGCCAAAATTGACAAATGGGATCTAATTAAACTCAAGAGCTTCTGCACAGCAAAAGAAACTATCATCAGAGTGAACAGGCAACCTACAAAATGGGAGAAAATTTTCGCAACCTACTCATATGACAAAGGGCTAATATCCAGAATCTACAATGAACTCAAACAAATTTACAAGAAAACAACAAACAACCCCATCAAAAAGTGGGCGAAGGACATGAACAGACACTTCTCAAAAGAAGACATTTATGCAGCCAAAAAACACATGAAAAAATGCTCATCATCACTGGCCATCAGAGAAATGCAAATCAAAACAACAATGAGATATCATCTCACACCAGATAGAATGGCAATCATTAAAAAGTCAGGAAACAACAGGTGCTGGAGAGGATGTGGAGAAATAGGAACACTTTTACACTGTTGGTGGGACTGTAAACTAGTTCAACCATTGTGGAAGTCAGTGTGGCGATTCCTCAGGGATCTAGAACTGGAAATACCATTTGACCCAGCCATCCCATTACTGGGTATATACCCAAAGGACTATAAATCATGCTGCTATAAAGACACAAGCACATGTATGTTTATTGCGGCATTATTCACAATAGCAAAGACTTGGAACCAACCCAAATGTCCAACAATGATAGACTGGATGAAGAAAACGTGGCACATATACACCATGGAATACTATGCAGCCATAAAAAATGATGAGTTCATGTCCTTTGTAGGGACATGGATGAAATTGGAAACCATCATTCTCAGTAAACTCTCGCAAGAACAAAAAACCAAACACCGCATATTCTCACTCATAGGTGGGAATTGAACAGTGAGATCACACGGACACAGGAAGGGGAACATCACACTCTGGGGACTGTTGTGGGGTGGGGGGAGGGGGGAGGGATAGCATTGGGAGATATACCTAATGCTAGATGACGAGTTAGTGGGTGCAGCACACCAGCATGGCACATGTATACGTATGTAACTAACCTGCACAATGTGCACATGTACCCTAAAACTTAAAGTATAAT*AATAAAAAAATAATAATAAATAAAAAATAAATAAAT****AAAA*TAGCTTTC**AATTCTCTGCTTAAGCTCAAGAAATTGCATTTTATTTCCTTCAATATCATAATCACCAT

**Clone 84; PA3; SpIRE(97/622)-84: Filled site spans: chr11:100652962-100658622**

Empty Site:

TACAATAGCCACCAAAAAACAAACAAACAAACAAAAACTTAGAAATATACCTAACCAAGGATGTTAAAGACCTCTGCAAGGAAAACTACAAAACACTGCTGAAATAAATCATAGAAGACA

CAAACAAAT**GAAAGCATAAC**CCATGCTCATGGATAGGTAG

AATCAATATTGTGAAAATGATCATACTGCCAAAAGCAATCTACAAATTCATTGCAATTCCTATCAAAATACCACCATCATTCTTCACAGAACTAGAAAAAAAAATAGTAAAATTTACATG

Filled Site:

GAAAACTACAAAACACTGCTGAAATAAATCATAGAAGACACAAACAAAT**GAAAGCATAAC**AGAGGATCCAAGATGGCCGAATAGGAACAGCTCCGGTCTACACCTCCCAGCGTGAGCGACGCAGAAGACGGGTGATTTCTGCATTTCCATCTGAGCTTTGAAGAGAGCAGTGGTTCTCCCAGCACGCAGCTGGAGATCTGAGAACGGGCAGACTGCCTCCTCAAGTGGGTCCCTGACCCCTGACCCCCGAGCAGCCTAACTGGGAGGCACCCCCCAGCAGGGGCAGACTGACACCTCACACGGCCGGGTACTCCAACAGAACCGCAGGTGAGGGTCCTGTCTGTTAGAAGGAAAACTAACAAACAGAAAGGACATCCACACCAAAAACCCATCTGTACATCACCATCATCAAAGACCAAAAGTAGATAAAACCACAAAGATGGGGAAAAAACAGAACAGAAAAACTGGAAACTCTAAAAAGCAGAGCACCTCTCCTCCTCCAAAGGAATACAGTTCCTCACCAGCAGCGGAACAAAGCTGGACCGAGAATGACTTTGACGAGCTGAGAGAAGAAGGCTTCAGACGATCAAATTACTCCGAGCTATGGGAGGACATTCAAACCAAAGGCAAAGAAGTTGAAAACTTTGAAAAAAATTTAGAGGAATGTATAACTAGAATAACCAATACAGAGAAGTGCTTAAAGGAGCTGATGGAGCTGAAAACCAAGGCTCGAGAACTACGTGAAGAATGCAGAAGCCTCAGGAGCCGATGCGATCAACTGGAAGAAAGGGTATCAGCGATGGAAGATGAAATGAATGAAATGAAGCGAGAAGGGAAGTTTAGAGAAAAAAGAATAAAAAGAAAGGAGCAAAGCCTCCAAGAAATATGGGACTATGTGAAAAGACCAAATCTACGTCTGATTTGTGTACCTGAAAGTGACGGGGAGAACGGAACCAAGTTCGAAAACACTCTGCAGGATATTATCCAGGAGAACTTCCCCAATCTAGCAGGGCAGGCCAACATTCAGATTCAGGAAATACAGAGAACGCCATAAAGATACTCCTTGAGAAGAGCAACTCCAAGACACATAATTGTCAGATTCACCAAAGTTGAAAGGAAGGAAAAAATGTTAAGGGCAGCCAGAGAGAAAGGTCGGATTACCCTCAAAGGGAAGCCCATCAGATTAACAGCAGATCTCTCGGCAGAAACCCTACAAGCCAGAAGAGAGTGGGGGCCAATATTCAACATTCTTAAAGAAAAGAATTTTCAACCCAGAATTTCATATCCAGCCAAACTAAGCTTCATAAGTGAAGGAGAAATAAAATACTTTACAGACAAGCAAATGCTGAGAGATTTTGTTACCACCAGGCCTGCTCTAAAAGAGTTCCTGAAGGAAGTGCTAAACATGGAAAGGAACAACCGGTACCAGCCGCTGCAAAATCATGCCAAAATGTAAAGACCATCGAGACTAGGAAGAAACTGCATCAACTAAAGAGCAAAATAACCAGCTAACATCATAATGACAGGATCAAATTCACACATAACAATATTAACTTTAAATGTAAATGGACTAAATGCTCCAATTAAAAGACACAGACTGGCAAATTGGATAAAGAGTCAAGACCCATCAGTGTGCTGTATTCAGGAAACCCATCTCACGGGCAGAGACACACATAGGCTCAAAATAAAAGGATGGAAGAAGATCTACCAAGCAAATGGAAAACAAAAAAAGGCAGGGGTTGCAATCCTAGTCTCTGATAAAACAGACTTTAAACCAACAAAGATCAAAAGAGACAAAGAAGGCCATTACATAATGGTAAAGGGATCAATTCAACAAGAAGAGCTAACTATCCTAAATATATATGCACCCAATACAGGAGCACCCAGATTCATAAAGCAAGTCCTGAGTGACCTACAAAGAGACTTAGACTCCCACACATTAATAATGGGAGACTTTAACACCCACTGTCAACATTAGACAGATCAACGAGACAGAAAGTCAACAAAGATACCCAGGAATTGAACTCAGCTCTGCACCAAGTGGACCTAATAGACATCTACAGAACTCTCCACCCCAAATCAACAGAATATACATTTTTTTCAGCACCACACCACACCTATTCCAAAATTGACCACATAGTTGGAAGTAAAGCTCTCCTCAGCAAATGTAAAAGAACAGAAATTACAACAAACTGTCTCTCAGACCACAGTGCAATCAAACTAGAACTCAAGATTAAGAATCTCACTCAAAACCGCTCAACTACATGGAAACTGAACAACCTGATCCTGAATGACTACTGGGTACATAACGATATGAAGGCAGAAATAAAGATGTTCTTTGAAACCAACGAGAACAAAGACACAACATACCAGAATCTCTGGGACACATTCAAAGCAGTGTGTAGAGGGAAATTTATAGCACTAAATGCCCACAAGAGAAAGCAGGAAAGATCCAAAATTGACACCCTAACATCACAATTAAAAGAACTAGAGAAGCAAGAGCAAACACATTCAAAAGCTAGCAGAAGGCAAGAAATAACTAAAATCAGAGCAGAACTGAAGGAAATAGAGACACAAAAAACCCTTCAAAAAATTAATGAATCCAGGAGCTGGTTTTTGGAAGGATCAACAAAATTGATAGACTGCTGGCAAGACTAATAAAGAAAAAAAGAGAGAAGAATCAAATAGACGCAATAAAAAATGACAAAGGGGATATCACCACCAATCCCATAGAAATACAAACTACCATCAGAGAATACTACAAAAACCTCTATGCAAATAAACTAGAAAATCTAGAAGAAATGGATAAATTCCTCGACACATACACCCTCCCAAGACTAAACCAGGAAGAAGTTGAATCTCTGAATAGACCAATAACAGACTCTGAAATTGTGGCAATAATCAATAGCTTACCGACCAAAAAGAGTCCAGGACCAGATGGATTCACAGCCAAATTCTACCAGAGGTACAAGGAGGAACTGGTACCATTCCTTCTGAAACTATTCCAATCAATAGAAAAAGAGGGAATCCTCCCTAACTCATTTTATGAGGCCAGCATCATCCTGATACCAAAGCCGGGCAGAGACACAACAAAAAAGAGAATTTTAGACCAATATCCTTGATGAACATTGTTGCAAAAATCCTCAATAAAATACTGACAAACCAAATCCAGCAGCACATCAAAAAGCTTATCCACCATGATCAAGTGGGCTTCATCCCTGGGATGCAAGGCTGGTTCAATATACGCAAAACAATAAATGTAATCCAGCATATAAACAGAACCAAAGACAAAAACCACAGGATTATCTCAATAGATGCAGAAAAGTCCTTTGACAAAATTCAACAATGCTTCATGCTAAAAACTCTCAATAAATTAGGTATTGATGGAACGTATTTCAAAATAATAAAAGCTATCTATGACAAACCCACAGCCAATATCATACTGAATGGGCAAAAACTGGAAGCATTCCCTTTGAAAACTGGCACAAGACAGGGATGCCCTCTCTCACCACTCCTATTCAACATAGTGTTGGAAGTTCTGGCCAGGGAAATTAGGCAGGAGAAAGAAATAAAGGGTATTCAATTAGGAAAAGAGGAAGTCAAATTGTCCCTGTTTGCAGATGACATGATCGTATATCTAGAAAATCCCATTGTCTCAGCCCAAAATCTCCTTAAGCTGATAAGCAACTTCAGCAAAGTCTCAGGATACAAAATCAATGTACAAAAATCACAAGCATTCTTATACACCAAAAACAGACAAACAGAGAGCCAAATCATGAGTAAACTCCCATTCACAATTGCTTCAAAGAGAATAAAATACCTAGGAATCCAACTTACAAGGGATGTGAAGGATCTCTTCAAGGAGAACTGCAAACCACTGCTCAAGGAAATAAAAGAGGATACAAACAAATGGAAGAACATTCCATGCTCATGGGTAGGAAGAATCAATATCATGAAAATGGCCATACTGCCCAAGGTAATTTACAGATTCAATGCCATGCCCATCAAGCTACCAATCACTTCCTTCACAGAATTGGAAAATAGTACTTTAAAGTTCATATGGAACCAAAAAAGAGCCCACATCGCCAAGTCAATCCTAAGCCAAAAGAACAAAGCTGGAGGCATCACACTACCTGACTTCAAACTATACTACAAGGCTACAGTAACCAAAACAGCATGGTACTGGTACCAAAACAGAGATATAGATCAATGGAACAGAACAGAGCCCTCAGAAATAATGCCGCATATCTACAACTATCTGATCTTTGACAAACCTGACAAAAACAAGCAATGGGGAAAGGATTCCCTATTTAATAAATGGTGCTGGGAAAACTGGCTAGCCATATGTAGAAAGCTGAAACTGGATCCCTTCCTTACACCTTATACAAAAATCAATTCAAGATGGATTAAAGACTTAAACTTTAGACCTAAAACCATAAAAACCCTAGAAGAATACCTAGGCATTACCATTCAGGAAACAGGCATGGGCAAGGACTTCATGTCTTTGCCCAAACACCAGAAGCAATGGCAACAAAAGCCAAAATTGACAAATGGGATCTAATTAAACTAAAGAGATTCTGCACAGCAAAAGAAACTACCATCAGAGTGAACAGGCAACCTACAAAATGGGAGAAAATTTTTGCAACCTACTCATCTGACAAAGGGCTAATATCCAGAATCTACAATGAACTCAAACAAATTCACAAGAAAAAAACAAACAACCCCATCAAAAAGTGGGCGAAGGACATGAACAGACACTTCTCAAAAGAAGACATTTATGCAGCCAAAAAACACATGAAAAAATGCTCACCATCACTGGCCATCAGAGAAATGCAAATCAAAACCACAACGAGATACCATCTCACACCAGTTAGAATGGCAATCATTAAAAAGTCAGGAAACAAGAGGTGCTGGAGAGCATGTGGAGAAATAGGAACACTTTTACACTGTTGGTGGGACTGTAAACTAGTTCAACCATTGTGGAAGTCAGTGTGGCGATTCCTCAGGGATCTAGAACTAGAAATACCATTTGACCCAGCCATCCCATTACTGGCTATATACCCAAAGGACTATAAATCATGCTGCTATAAAGACACATGCACACGTATGTTTATTGCAGCATTATTCACAATAGCAAAGACTTGGAACCAACCCAAATGTCCAACAATGATAGACTGGATTAAGAAAATGTGGCACATATACACCATGGAGTACTATGCAGCCATAAAAAATGATGAGTTCACGTCCTTTGTAGGGACATGGATGAAACTGGAAATCATCATTCTCAGTAAACTATCGCAAGAACAAAAAACCAAACACCACATATTCTCACTCATAGGTGGGAATTGAACAATGAGAACACATGGACACAGGAAGGGGAACGTCACACTCTGGGGACTGTTGTGGGGTGGGGGGAGGGGGGAGGGATAGCATTGGGAGATATACCTAATGCTAGATGACGAGTTAGTGGGTGCAGTGCACCAGCATGGCACATGTATACATATGTAACTAACCTGCACATTGTGCACATGTACCCTAAAACTTAAAGTATAAT*AATAATAATAAATAAATAAATAAATAAATAAATAAATAAAAA****GAAA*GCATATC**CCATGCTCATGGATAGGTAGAATCAATATTGTGAAAATGATCATACTGCCAAAA

**Clone 85; PA3; SpIRE(97/622)-85: Filled site spans: chr11:29416064-29421725**

Empty Site:

TGAAGGGTAATCTTTTCCCTTTTACTCCATCCTAGTAAAGTATAAGTCCCCTAAATTATTTTTCTTAAAAAAAAATCAAACATTATTCCTCTTAAAATAAAAAGCCAGAGTTACGTAAGT

TTAGGAAGTATT**AAAAATTATC**AAGCAGGTGTCTTTGCTA

TAGGTATTTTCAGAAACTTGAGTTTTCTAATGTAATTTCCTTACAAATATCAAAGGGTACAAAAATAGCAGAAGCCATTTTAAACTATAAGCACTTCATCTGCCTTGTGGATTAACCAAT

Filled Site:

CATTATTCCTCTTAAAATAAAAAGCCAGAGTTACGTAAGTTTAGGAAGTATT**AAAAATTATC**GGGGATTGGCCAAGATGGCCGAATAGGAACAGCTCCGGTCTACAGCTCCCAGCGTTAGCGACGCAGAAGACGGGTGATTTCTGCATTTCCATCTGAGCTTTGAAGAGAGCAGTGGTTCTCCCAGCACGCAGCGGGAGATCTGAGAACAGGCAGACTGCCTCCTCAAGTGGGTCCCTGACCCCTGACCCCCGAGCAGCCTAACTGGGAGGCACCCCCCAGCAGGGGTGGACTGACACCTCACACAGCCGGGTACTCCAACAGACCTGCAGCTGAGGGTCCTGTCTGTTAGAAGGAAAACTAACAAACAGAAAGGACATCCACACCAAAAACCCATCTGTACATCACCATCATCAAAGACCAAAAGTAGATAAAACCACAAAGATGGGGAAAAAACAGAACAGAAAAACTGGAAACTCTAAAAAGCAGATCGCCTCTCCTCCTCCAAAGGAACGCAGTTCCTCACCAGCAATGGAACAAAGCTGGAGGGAGCATGACTTTGACGAGCTGAGAGAAGAAGGCTTCAGACGATCAAATTACTCTGAGCTATGGGAGGACATTCAAACCAAAGGCAAAGAAGTTGAAAACTTTGAAAAAAATTTAGAAGAATGTATAACTAGAATAACCAACACAGAGAAGTGCTTAAAGGAGCTGATGGAGCTGAAAACCAAGGCTTGAGAACTACGTGAAGAATGCAGAAGCCTCAGGAGCCAATGCGATCAACTGGAAGAAAGGGTATCAGCAATGGAAGATGAAATGAATGAAATGAAGCAAGAAGGGAAGTTTAGAGAAAAAAGAATAAAAAGAAACGAGCAAAGCCTCCAAGAAATATGAGACTATGTGAAAAGACCAAATCTACGTCTGATTGGTGTACCTGAAAGTGACGGGGAGAATCGAACCAAGTTGGAAAACACTCTGCAGGATATTATGCAGGAGAACTTCCCCAATCTAGCAAGGCAGGCCAACATTCAGATTCAGGAAATACAGAGAACGCCACAAAGATACTCCTCGAGAAGAGCAACTCCAAGAAACATAATTGTCAGATTCACCAAAGTTGAAATGAAGGAAAAAATGTTAAGGGCAGCCAGAGAGAAAGGTCAGGTTACCCTCAAAGGGAAGCCCATCAGACTAACAGCGGATCTCTCAGCAGAAACTCTACAAGCCAGAAGAAAGTGGGGGCCAATATTCAACATTTTTAAAGAAAAGAATTTTCAACCCAGAATTTCATATCCAGCCAAACTAAGCTTCAAAAGTGAAGGAAAAATAAAATACTTTACAGACAGGCAAATGCTGAGAGATTTTGTTACCACCAGGCCTGCCCTAAAAGAGCTCCTGAAGGAAGCACTAAACATGGAAAGGCACAACCGGTACCAGCTGCTGCAAAATCATGCCAAAATGTAAAGACCATCGAGACTACGAAGAAACTGCATCAACTAATGAGCAAAATAACCAGCTAACATCATAATGACAGGATCAAATTCACACATAACAATATTAACTTTAAATGTAAATGGACTAAATGCTCCAATTAAAAGACACAGACTGGCAAATTGGATAAAGAGTCAAGACCCATCAGTGTGCTGTATTCAGGAAACCCATCTCACATGCAGAGACACACATAGGCTCAAAATAAAAGGATGGAGGAAGATCTACCAAGCAAATGGAAAACAAAAAAAGGCAGGGGTTGCAATCCTAGTCTCTGATAAAACAGACTTTAAACCAACAAAGATCAAAAGAGACAAAGAAGGCCATTGCATAATGGTAAAGGGATCAATTCAACAAGAAGAGCTAACTATCCTAAATATATATGCACCCAATACAGGAGCACCCAGATTCATAAAGCAAGTCATGAGTGACCTACAAAGAGACTTAGACTCCCACACATTAATAATGGGAGACTTTAACACCCCACTGTCAACATTAGACAGATCAACGAGACAGAAAGTCAACAAGGATACCCAGGAATTGAACTCAGCTCTGCACCAAGTGGACCTAATAGACATCTACAGAACTCTCCACCCCAAATCAACAGAATATACATTTTTTACAGCACCACACCACACCTATTCCAAAATTGACCACATAGTTGGAAGTAAAGCTCTCCTCAGCAAATGTAAAAGAACAGAAATTATAACAAACTATCTCTCAGACCACAGTGCAATCAAACTAGAACTCAGGATTAAGAATCTCACTCAAAACCGCTCAACTACATGGAAACCTAACAACCTGCTCCTGAATGACTACTGGGTACATAACGAAATGAAGGCAGAAATACAGATGTTCTTTGAAACCAATGAGAACAAAGACACAACATACCAGAATCTCTGGGACGCATTCAAAGCAGTGTGTAGAGGGAAATTTATAGCACTAAATGCCCAAAAGGGAAAGCAGGAAAGACCCAAAATTGACACCCTAACATCACAACTAAAAGAACTAGAAAAGCAAGAGCAAACACATTCAAAAGCTAGCAGAAGGCAAGAAATAACTAAAATCAGAGCAGAACTTAAAGAAATAGAGACACAAAAAACCCTTCAAAAAATTAATGAATCCAGGAGCTGGTCTTTTGAAAAGATCAACAAAATTGATACACCGCTAGCAAGACTAATAAAGAAAAAAAGAGAGAAGAATCAAATAGACACAATAAAAAATGACAAAGGGGATATCACCACTGATCCCACAGAAATACAAACTACCATCAGAGAATACTACAAACACCTCTATGCAAATAAACTAGAAAATCTAGAAGAAATGGATAAATTCCTCGACACATACACTCTCCCAAGACTAAACCAGGAAGAAGTTGAATCTCTGAATAGACCAATAACAGGCTCTGAAATTGTGGCAATAATCAATAGCTTACCAACCAAAAAGAGTCCAGGATCAGATGGATTCACAGCTGAATTCTACCAGAGGTACAAGGAGGAAGTGGTACCATTCCTTCTGAAACTATTCCAATCAATAGAAAAAGAGGGAATCCTCCCTAACTCATTTTATGAGGCCAGCATCATCCTGATACCAAAGCCAGGCAGAGACACAACAAAAAAAAGAGAATTTTAGACCAATATCCCTGATGAAAATCGATGCAAAAATCCTCAATAAAATACTGGCAAACCGAATCCAGCAGCACATCAAAGAGCTTATCCACCATGATCAAGTGGGCTTCATCCCTGGGATGCAAGGCTGGTTCAACATACGAAAATCAATAAATGTAATCCAGCATATAAACAGAACCAACGACAAAAACCACATGATTATCTCAATAGATGCAGAAAAGGTCTTTGACAAATTTCAACAACCCTTCATGCTAAAAACTCTCAATAAATTAGGTATTCATGGAACGTATTTCAAAATAATAAGAGGTATCTATGACAAACCCACAGCCAATATCATACTGAATGGGCAAAAACTGGAAGCATTCCCTTTGAAAACTGGCACAAGACAGGGATGCCCTCTCTCACCACTCCTATTCAACATAGTGTTGGAAGTTCTGGCCAGGGCAATTAGGCAGGAGAAAGAAATAAAGGATATTCAATTAGGAAAAGAGGAAGTCAAATTGTCCCTGTTTGCAGATGGCATGATTGTATATCTAGAAAACCCCATTGTCTCAGCCCAAAATCTCCTTAAGCTGATAAGCAACTTCAGCATAGTCTCAGGATAAAAAATCAATGTACAAAAATCACAGGCATTCTTATACACCAACAACAGACAAACAGAGAACCAAATCATGAGTGAACTCCCATTCACAATTGCTTCAAAGAGAATAAAATACCTAGGAATCCAACTTACAAGGGATGTGAAGGACCTCTTCAAGGAGAACTACAAACCACTGCTCAATGAAATAAAAGAGGATACAAACAAATGGAAGAACATTCCATGCTCATGGGTAGGAAGAATCAATATCGTGAAAATGGCCATACTGCCCAAGGTAATTTACAGATTCAATACCATCCCCATCAAGCTACCAATGACTTTCTTCACAGAATTGGAAAAAACTACTTTAAAGTTCATATGGAACCAAAAGAGAGCAGGCATCGCCAAGTCAATCCTAAGCCAAAAGAACAAAGCTGGAGGTATCACACTACCTGACTTCAAACTATACTACAAGACTACAGTAACCAAAACAGCATGGTACTCGTACCAAAACAGAGATATAGATCAATGGAACAGAACAGAGCCCTCAGAAATAATGCTGCATATCTACAACTATCTGATCTTTGACAAACCTGAGAAAAACAAGCAATGAGGAAAGGATTCCCTATCTAATAAATGGTGCTGGGAAAACTGGCTAGCCCTATGTAGAAAGCTGAAACTGGATCCCTTCCTTACACCTTATACAAAAATCAATTCAAGATGGATTAAAGACTTAAACGTTAGACCTAAAACCAGAAAAACCCTAGAAGAAAACCTAGGCATTACCATTCAGGACATAGGCATGGGCAAGGACTTCATGTCTAAAACACCAAAAGCAATGGCAACAAAGGCCAAAATTGACAAATGGGATCTAATTAAACTAAAGAGCTTCTGCACAGCAAAAGAAACTACCATCAGAGTGAACAGGCAACCTACAGAATGGGAGAAAATTTTCGCAATCTACTCATCTGACAAAGGGCTAATATCCAGAATCTACAATGAACTCAAACAAATTTACAAGAAAAAAACAAACAACCCCATCGAAAAGTGGGCGAAGGACATGAACAGACAATTCTGAAAAGAAGACATTTATGCAGCCAAAAAACACATGAAAAAATGCTCACCATCACTGGCCATCAGAGAAATGCAAATCAAAACCACAATGAGATATCATCTCACACCAGTTAGAATGGCAATCATTAAAAAGTCAGGAAACAACAGGTGCTGGAGAGGATGTGGAGAAATAGGAACGATTTTACACTGTTGCTGGGACTGCAAACTAGTTCAACCATTGTGGAAGTCAGTGTGGCGATTCCTCAGGGATCTAGAACTAGAAATACCATTTGACCCAGCCATCCCATTACTGGGTATATACCCAAAGGACTATAAATCATGCTGCTATAAAGATACATGCACACGTATGTTTATTGCGGCATTATTCACAATAGCAAAGACTTGGAACCAACCCGAATGTCCAACAATGATAGACTGGATTAAGAAAATGTGGCACATATACACCATGGAGTACTATGCAGCCATAAAAAATGATGAGTTCACGTCCTTTGTAGGCACGTGGATGAAATTGGAAATCATCATTCTCAGTAAACTATCGCAAGAACAAAAAACCAAACACCGCATATTCTCACTCATAGGTGGGAATTGAACAATGAGAACACATGGACACAGGAAGGGGAATATCACACTCTGGGGACTGTTGTGGGGTGGGGGGAGCGGGGAGGAATAGCATTGGGAGATATACCTAATGCTAGATGACGAGTTAGTGGGTGCAGCGCAGCAGCATGGCACATGTATACGTATGTAACTAACCTGCACATTGTGCACATGTACCCTAAAACTTGAAGTATAAT*AATAATAATTTAAAAAAAAAGTGTAGGGATTGAGTAGTGGAAAAAAAAACAAA****AAAAA*TTATC**AAGCAGGTGTCTTTGCTATAGGTATTTTCAGAAACTTGAGTT

**Clone 89; PA3; SpIRE(97/622)-89: Filled site spans: chr12:60761320-60767018**

Empty Site:

AGAAACCAAAACCTTGCATCATCATATTTCAAGGCCAAGTTAACTTAATAATAATTAAGTTGTCACTGATAATTTATTTTTTAAAAGTTAAGCATGGAAACAGTGCCTCCTCTTTTAAAC

TAATTTT**AAAATTCTT**AAAGTCAGGAGCCAATTATTAAAG

TAAAGGAATATAATTCAACTGCTAATAATATTATCATGTGTAATAGATGCTATCTTAAATGTTAATAGCCAATATAGTTTTCCTCTCCTTTACACTGAAACCATCTGGAAATAAAATTCA

Filled Site:

TTAAAAGTTAAGCATGGAAACAGTGCCTCCTCTTTTAAACTAATTTT**AAAATTTTT**GGAGGGGAGGAGCCAAGATGGCCGAATAGGAACAGCTCTGGTCTACAGCTCCCAGCATGAGCGATGCAGAAGACGGGTGATTTCTGCATTTCCATCTGAGCTTTGAAGAGAGCAGTGGTTCTCCCAGCATGCAGCTGGAGATCTGAGAACGGGCAGACTGCCTCCTCAAGTGGGTCCCTGACCCCTGACCCCCAAGCAGCCTAACTGGGAGGAACCCCCCAGCAGGGGCAGACTGACACCTCACAGGGCCGGGTACTCCAACAGACCAGCAGCTGAGGGTCCTGTCTGTTAGAAGGAAAACTAACAAACAGAAAGGACATCCACACCAAAAACCCATCTGTACATCACCATCATCAAAGACCAAAAGTAGATAAAACCACAAAGATGGGGAAAAAACAGAGCAGAAAAACTGGAAACTCTAAAAAGCAGGGTGCCTCTCCTCCTCCAAAGGAATGCAGTTCCTCACCAGCAATGGAACAAAGCTGGACGGAGAATGACTTTGACGAGCTGAGAGAAGAAGGCTTCAGACAATCAAATTGCTCCGAGCTATGGGAGGAAATTCAAACCAAAGTCAAAGAAGTTGAAAACTTTGAAAAAAGTTTAGAAGAATGTATAACTCGAATAACCAATACAGAGAAGTGCTTAAAGGAGCTGATGGAGCTGAAAACCAAGGCTTGAGAACTACGTGAAGAATGCAGAAGCCTCAGGAGCCGATGTGATCAACTGGAAGAAAGGGTATCAGCGATGGATGATGAAATGAATGAAATGAAGCGAGAAGGGAAGTTTAGAGAAAAAAGAATAAAAAGAAAGGAGCAAAGCCTCCAAAAATATGGGACTATGTGAAAAGACCGAATCTACGTCTGATTGGTGTACCTGAAAGTGACGGGGAGAATGGAACCAAGTTGGAAAACACTCTGCAGGATATCATCCAGGAGAACTTCCCCAATCTAGCAAGGCAGGCCAACATTCAGATTCAGAAATACAGAGAACGCCACAAAGATACTCCTCGAGAAGAGCAACTCCAAGACACATAATTGTCAGATTCACTAAAGTTGAAATGAAGGAAAAAATGTTAAGGGCAGCCAGAGAGAAAGGTCGGGTTACTCACAAAGGGAAGCCCATCAGACTAACAGTGGATCTCTCGGCAGAAACTCTACAAGCCAGAAGAGAGTGGGGACCAATATTCAACATTCTTAAAGAAAAGAATTTTCAACCCAGAATTTCATATCCAGCCAAACTAGGCTTCATAAGTGAAGGAGAAATAAAATACTTTACAAACAAGCAAATGCTGAGAGATTTTGTCACCACCAGGCCTGCCCTAAAAGAGCTCCTGAAGGAAGCACTAAACATGGAAAGGAACAACCGATACCAGCCGCTGCAAAATCATGCCAAAATGTAAAGACCATCGAGACTAGGAAGAAACTGCAGGAACTAACAAGCAAAATAACCAGCTAACATCATGACAGGATCAAATTCACACATAACAATATTAACTTAAAATGTAAATGGACTAAATGCTCCAATTAAAAGACACAGACTGGCAAATTGGATAAAGAGTCAAGACCCATCAGTGTGCTGTATTCAGGAAACCCATCTCATGTGCAGAGACACACATAGGCTCAAAATAAAGGAAGGAGGAAGATCTACCAAGCAAATGGAAAACAAAAAAAGGCAGGGGTTGCAATCCTAGTCTCTGATAAAACAGACTTTAAACCAACAAAGATCAAAAGAGACAAAGAAGGCCATTACATAATGGCGAAGGGATCAATTCAACAAGAAGAGCTAACTATTCTAAATATATATGCACCCAATACAGGAGCACCCAGATTCATAAAGCAAGTCCTGAGTGACCTACAAAGAGACTTAGACTCCCACACATTAATAATGAGAGACTTTAACACCCCACTGTCAACATTAGACAGATCAACGAGACAGAAAGTCAACAAGGATACCCAGGATTGAACTCAGCTCTGCACCAAGCAGACCTAATAGACATCTACAGAACTCTCCACCCCTAATCAACAGAATATACATTTTTTTTTCAGTGCAACACCACACCTATTCCAAAATTGACCACATAGTTGGAAGTAAAGCACTCCTCAGCAAATGTAAAAGAACAAAAATTATAACAAACTGTCTTTCAGACCACAGTGCAATCAAACTAGAACTCAGGATTAAGAATCTCACTCAAAACCGCTCAACTACATGGAAACTGAGCAACCTGCTCCTGAACGACTACTGGGTACATAAAGAAATGAAGGCAGAAATAAAGATGTTCTTTGAAACCAACGAGAACAAAGAGACAACATACCCGAATCTCTGGGACGCATTCAAAGCAGTGTGTAGAGGGAAATTTATAGCACTAAATGCCCACAAGAGAAAGCAGGAAAGATCCAAAATTGACACCCTAACATCACAATTAAAAGAACTAGAAAAGCAAGAGCAAACACATTCAAAAGCTAGCAGAAGGCAAGAAATAACTAAAATCAGAGCAGAACTGAAGGAAATAGAGACACAAAAAACCCTTCAAAAAATTAATGAATCCAGGAGCTGGTTTTTTGAAAGGATCAACAAAATTGATAGACTGCTAGCAAGACTAATAAAGAAAAAAAAGAGAGAAGAATCAAGTAGATGCAATAAAAAACAATAAAGGGGATATCACCACTGATCCCACAGAAATACAAACTACCATCAGAGAATACTGCAAACACCTCTACACAAATAAACTAGAAAATCTAGAAGAAATGGATACATTCCTCGACACATACACTCTCCCAAAACTAAACCAGGAAGAAGTTGAATCTCTGAATAGACCAATAACAGGAGCTGAAATTGTGGCAATAATCAGTAGCTTACCATCCAAAAAGAGTCCAGGACCAGATGGATTCACAGCCGAATTGTTCCAGAGGTACAAGGAGGAACTGGTACCATTCCTTCTGAAACTATTCCAATCAATAGAAAAAGAGGGAATCCTCCCTAACTCATTTTATGAAGCCAGCATCATCCTGATACCAAAGCCGGGCAGAGACACAACCAAAAAAGAGAATTTTAGACCAATATCCTTGATGAACATTGATACAAAAATCCTCAATAAAATACTGGCAAACTGAATCCAACAGCACATCAAAAAGCTTATCCACCATGATCAAGTGGGCTTCATCCCTGGGATGCAAGTCTGGTTCAGTATATGCAAATCAATAAATGTAATCCACCATATAAACAGAACCAATGACAAAAACCACATGATTATCTCAATAGATGCAGAAAAGGCCTTTGACAAAATTCAACAACGCTTCATGCTAAAAAGTCTCAATAAATTAGGTATTGATGGGATGTATCTCAAAATAATAAGAGCTATCTATGAGAAACCCACAGCCAATATCATACTGAATGGGCAAAAACTGGAAGCATTCCCTTTGAAAACGGGCACAAGACAGGGATGCCCTCTCTCACCACTCCTATTCAACATAGTGTTGGAAGTTCTGGCCAGGGCAATTAGGCAGGAGAAGGAAATAAAGGGTATTCAATTAGGAAAAGAGGAAGTCAAATTGTCCCTGTTTGCAGATGACATGATTGTATATCTAGAAAACCCCACTGTCTCAGCCCAAAATCTCCTTAAGCTGATAAGAAACTTCAGCAAAGTCTCAGGATACAAAATCAATGTACAAAAATCACAAGCATTCCTATACACCAAAAACAGACAAACAGAGAGCCAAATCATGAGTGAACTCCCATTCACAATTGCTTCAAAGAGAATAAAATACCTAGGAATCCAACTGACAAGGGATGTGAAGGACCTCTTCAAGGAGAACTACAAACCACTGCTCAAGGAAATAAAAGAGGATACAAACAAGTGGAAGAACATTCCATGCTCATGGGTAGGAAGAATCCATATCGTGAAAATGGCCATACTGTCCAAGGTAATTTACAGATTAAATGCCATCCCCATCAAGCTACCAATGACTTTCTTCACAGAATTGGAAAAAACTACTTTAAAGTTCATATGGAAACCAAAAAAGAACCCACATCACCAAATCAATCCTAAGCCAAAAGAACAAAGCTGGAGGCATCACACTACCTGACTTCAAATTATACTACAAGGCTACAGTAACCAAAACAGCATGGTACTGGTACCAAAACAGAGATATAGATCAATGGAACAGAACAGAGACCTCAGAAATAACGCCACATATCTACAACTATCTGATCTTTGACAAACCTGAGAAAAACAAGTAATGGGGAAAGGATTCCCTATTTAATAAATGGTGCTGGGAAAACTGGCTAGCCATATGTAGAAAGCTGAAAATGGATCCCTTCCTTACACCTTATACAAAAATCAATTCAAGATGGATTAAAGACTTAAACATTAGACCTAAAACCATAAAAACCCTAGAAGAAAACCTAGGCATTACCTTTCAGGACATAGGCATGGGCAAGGACTTCATGTCTAAAACACCAAAAGCAATGGCAACAAAAGACAAAATTGACAAATGGGATCTAATTAAACTAAAGAGCTTCTGCACAGCAAAAGAAACTACCATCAGAGTGAACAGGCAACCTACAAAATGGGAGAAAATTTTTGCAACCTACTCATCTGACAAAGGGCTAATATCCAGAATCTACAATGAACTCAAACACATTTACGAGAAAAAAACAACCCCATCAAAAGTGGGTAAAGGACATGAACAGACACGTCTCAAAAGAAGACATTTATGCAGCCAAAAAACACACGAAAAAATGCTCACCATCACTGGCCATCAGAGAAATGCAAATCAAAACCACAATGAGACACCATCTCACACCAGTTAGAATGGCAATCATTAAAAAGTCAGGAAACAACAGGTACTGGAGAGGATGTGGAGAAATAGGAACACTTTTATACCGTTGGTGGGACAGTAAACTAGTTCAGCCATTGTGGAAGTCAGTGTGGCGATTTCTCAGGCATCTAGAACTGGAAATACCATTTGACCCAGCCATCCCATTACTGGGTATATACCCAAAGGACTATAAATCATGCATCTATAAAGACACATGCACACATATGTTTATTGCGGCATTATTCACAATAGCAAAGACTTGGAACCAAAGCAAATGTCCAACAAAGATAGACTGGATTAAGAAAATGTGGCACATATACACCATGGAATACTATGCAGCCATAAAAAATGATGAGTTCACGTCCTTTGTAGCGACATGGATGAAATTGGAAATCATCATTCTCAGTAAAGTATCGCAAGAACAAAAATCCAAACACCGTATATTCTCACTCATAGGTGGGATTTGAACAATGAGAACACATGGACACAGGAAGGGGAACATCACACTCTGGGGACTGTCGTGGGAGGCGGGAGGGATAGCATTAGGAGATATACCTAATGCTAGATGATGAGTTAGTGGGTGCAGCACACCAGCATGGCACATGTATACACATGTAACTAACCTGCACATTGTGCACATGTAACCTAAAACTTAAAGTATAAT*AATAATAAATTTAAAAAAAGAAAAAAAAAAGAATAAGAAATAACAAAAATACAAAATAAAAAATAAATTTTTTTATATAAAAACTAATTTCAGGTTAC****AAAA*TTCTT**AAAGTCAGGAGCCAATTATTAAAGTAAAGGAATATAATTCAACTGCTAATAATAT

**Clone 98; PA3; SpIRE(97/622)-98: Filled site spans: chr13:47378585-47384337**

Empty Site:

TTAAATGTTTCGTTCTGTAATAGTGCCTGTTCCAGATGATGAGAAGATAAGATACAGACTAGGAAAAAGTATTTGTAAACCTTTAATCCAACAATGAAATTGTGTCTAGAATACATAAAT

AACAT**AAAAAACTCAACAATAG**AAAAACAAAGTATCCAATGA

AAAGGGACCAAAAGTCACAATGAGACCTTTCACTAAAAAGAATATACAGTTGGCTAATTAGCATATGAAAAGATGCTTACATCATTAGCCTTTAGGTAAAGGCAAATTAAAACCACAATA

Filled Site:

TTTAATCCAACAATGAAATTGTGTCTAGAATACATAAATAACAT**AAAAATCTCAAAAGTAG**GTTGGGTTGCAGCCAAGATGGCCAAATAGGAATAGCTCCGGTCTACAGCTCCCAGCATGAGTGACGCAGAAGACGGGTGATATCTGCATTTCCATCTGAGCTTTGAAGAGAGCAGTGGTTCTCCCAGCACGCAGCTGGAGATCTGAGAACGGGCAGATTGCCTCCTCAAGTGGGTCCCTGACACCTGACCCCCGAGCAGCCTAACTGGGAGGCACCCCCCATTAGGGGCAGACTGACACCTCACACGGCCAGGTACTCCTCTGAGACAAAACTTCCAGAGGAATGATCAGACAGCAGCATTCGCGGTTCATGAAAATCCGCTGTTCTGCAGCCACCGCTGCTGGTACCCAGGCAAACAGGGTCTGGAGTGGACCTCTAGCAAATTCCAACAGACCTGCAGCTGAGGGTCCTGTCTGTTAGAAGGAAAACTAACAAACAGAAAGGACATCCACACCAAAAACCCATCTGTACATCACCATCATCATAAACCAAAAGTAGATAAAACCACAAAGATGGGAAAAAACAGAGCAGGAAAACTGGAAACTCTAAAAAGCAGAGCGCCTCTCCTCCTCCAAAGGAACGCAGCTCCTCACCAGCAACGGAACAAAGCTGGACGGAGAATAACTTTGACGAGTTGAGAGAAGAAGTTCTCAGACGATCAAACTACTCCAAGCTACAGGAGGAAATTCAAACCAAAGGCAAAGAAGTTGAAAACTTTGAAAAAAATTTAGACGAATGTATAACTAGAATAACCAATACAGAGAAGTGCTTAAAGGAGCTGATGGAGCTGAAAACCAAGGCTCGAGAACTACGTGAAGAATGCAGAAGCCTCAGGAGCCAATGCGATCAACTGGAAGAAAGGGTATCAGTGACGGAAGATCAAATGAATGAAATGAAGCAAGAAGGGGAGTTTAGAGAAAAAAGAATAAAAAGAAACGAACAAAGCCTCCAAGAAATAGGGGACTATGTGAAAAGACCAAATCTACGTCTGACTGGTGTACCTGAAAGTGACGGGGAGAATGGAACCAAGTTGGAAAACACTCTGCAGGATATTATCCAGGAGAACTTCCCCAATCTAGCAAGGCAGGCCAACATTCAGATTCAGGAAATACAGAGAATGCCACAAAGATACTCCTCGAGAAGAGCAACTCCAAGACACATAATTGTCAGATTCACCAAAGTTGAAATGAAGGAAAAAATGTTAAGGGCAGCCAGAGAGAAAGGTCGGGTTACCCTCAAAGGGAAGCCTACCAGACTAACAGTGGATCTCTCGGCAGAAACACTACAAGCCAGAAGAGAGTGGGGGCCAATATTCAACATTCTTAAAGAAAAGAATTTTCAACCCAGAATTTCCTATCCAGCCAAACTAAGCTTCATAAGTGAAGGAGAAATAAAATACTTTACAGACAAGCAAATGCTGAGAGATTTTGTCACCACCAGGCCTGCCCTAAAAGAGCTCCTGAAGGAAGCGCTAAACATGGAAAGGCACAACCGGTACCAGCCGCTGCAAAATCATGCCAAAATGTAAAGACCATCGAGGCTAGGAAGAAACTGCATCAACTAACGAGCAAAATAACCAGCTAACATCATAATGACAGGATCAAATTCACACATAACAATATTAACTTTAAATGTAAATGGACTAAATGTTCCAATTAAAAGACACAGACTGGCAAATTGGATAAAGAGTCAAGACCCATCAGTGTGCTGTATTCAGGAAACCCATCTCACGTGCAGAGACACACATAGGCTCAAAATAAAAGGATGGGGGAAGATCTACCAAGCAAATGGAAAACAAAAAAAGGCAGGGGTTGCAATCCTAGTCTCTGATAAAACAGACTTTAAATCAACAAAGATCAAAAGAGACAAAGAAGGCCATTATATAATGGTAAAGGGATCAATTCAACAAGAAGAGCTAACTATCCTAAATATATATGCACCCAATACAGGAGCATCCAGATGCATAAAGCAAGTCCTGAGTGACCTACAAAGAGACTTAGAATCCCACACAATAATAATGGGAGACTTTAACACCCCACTGTCAACATTAGACAGATCAACGAGACACAAAGTCAACAAGGATACCCAGGAATTGAACTCAGCTCTGCACCAAGCGGACCTAATAGACATCTACAGATCTCTCCACCCCAAATCAACAGAATATACATTTTTTTCAGCACCACACCACACCTATTCCAAAGTTGACCACATACTTGGAAGTAAAGCTCTCCTCAGCAAATGTAAAAGATTAGAAATTATAACAAACTGTCTCTCAGACCACAGTGCAATCAAACTAGAACTCAGGATTAAGAAACTCACTCAAAACAACTCAACTACATGGAAACTGAAAAACCTGCTCCTGAATGACTACTGGGTACATAACGAAATGAAGGCAGAAATAAAGACGCTCTTTGAAACCAATGAGAACAAAGACACAACATACCAGAATCTCTGGGACTCATTCAAAGCAGTGTGTAGAGGGAAATTTATAGCACTAAATGCCCACAAGAGAAAGCAGGAAAGATCCAAAACTGACACCCTAACATCACAATTAAAAGAACTAGAAAAGCAAGAGCAAACACATTCAAAAGCTAGCAGAAGGCAAGAAATAACTAAAATCAGAGCAGAACTGAAGGAAATAGAGACACAAAAAACCCTTCAAAAAAATTAATGAATCCAGGAGCTGGTTTTTTGAAAGGATCAACAAAATTGATAGAACGCTAGCAAGACTAATAAAGAAGAAAAGAGAGAAGAATCAAATAGATGCAATAAAAAATGATAAAGGGGATATCACCACCGATCCCACAGAAATACAAACTACCATCAGAGAATACTACAAACGCCTCTACGCAAATAAACTAGAAAATCTAGAAGAAATGGATAAATTCCTCAACACATACACTCTCCCAAGACTAAACCAGGAAGAAGTTGAATCTCTGAATAGACCAATAACAGGCTCTGAAATTGTGGCAATAATCAATAGCTTACCAACCAAAAAGAGTCCAGGACCAGATGGATTCACAGCCGAATTGTACCAGAGGTACAAGGAGGAACTGGTACCATTCCTTCTGAAACTATTCCAATCAATAGGAAAAGAAGGAATCCTCCCTAACTCATTTTATGAGGCCAGCATCATCTTGATACCAAAGCCTGGCAGAGACACAACCAAAAAAGAGAATTTTAGACCAATATCCTTGATGAACATTGATGCAAAAATCCTCAATAAAATACTGGCAAACCGAATCCAGCAGCACATCAAAAAGCTTATCCACCATGATCAAGTGGGCTTCATCCCTGGGATGCCAGGCTGGTTCAATATACGCAAATCAATAAATGTAATCCAGCATATAACAGAACCAAAGACAAAAACCACATGATTATCTCAATAGATGCAGAAAAGACCTTTGACAAAATTCAACAACGCTTCATGCTAAAAACTCTCAATAAATTAGGTACTGATGGGATGTATCTCAAAATAATAAGAGCTATCTATGACAAACCCACAGCCAATATCATACTGAATGGGCAAAAACTGGAACCATTCCCTTTGAAAATTGGCACAAGACAGGGATGCCCTCTCTCACCACTCCTATTCAACATAGTGTTGGAAGTTCTGACCAGGGCAATTAGGCAGGAGAAGGAAATATAGGGTATTCAATTAGGAAAAGAGGAAGGCAAATTGTCCCTGTTTGCAGATGACATGACTGTATATCTAGAAAACCCTGTTGTCTCAGCCCAAAATCTCCTTAAGCTGATAAGCAACTTCAGCAAAGTCTCAGGATACAAAATCAATGTACAAAAATCACAAGCATTCTTATACACCAATAACAGACAGAGAGCCAAATCATGAGTGAACTCCCATTCACGATTGTTTCAAAGAGAATAAAATACCTAGGAATCCAACTTACAAGGGACGTGAAGGACCTCTTCAAGGAGAACTACAAACCACTGCTCAATGAAATAAAAGAGGATACAAACAAATGGAAGAACATTCCATGCTCATGGGTAGGAAGAATCAATATCATGAAAATGGCCATACTGCCCAAGGTAATTTATAGATTCAATGCCATCCCCATCAAGCTACCAATGACTTTCTTCACAGAACTGGAAAAAACTACTTTAAAATTCATATGGAACCAAAAAAGAGCCTGCATCGCCAAGTCAATCCTCAGCCAAAAGAACAAAGCTGGAGGCATCACGCTACCTGACTTCAAACTATACCACAAGGCTACAGTAAGCAAAACAGCATGGTACTGGTACCAAAACAGAGATATAGAGCAATGGAACAGAACAGAGCCCTCAGAAATAACACCACATATCTACAACTATCTGATCTTTGACAAACCTGAGAAAAATAAGCAATGGGGAAAGGATTGCCTATTTAATAAATGGTGCTGGGAAAACTGGCTAGCCATAAACAGAAAGCTGAAACTGGATCCCTTCCTTACACCTTATACAAAAATCAATTCAAGATGGATTAAAGACTTAAACATTAGGTCTAAAACCATAAAAACCCTAGAAGAAAACCTAGGGCATTACCATTCGGGACATAGGCATGGGCAGGGACTTCATGTCTAAAACACCAAAAGCAATGGCAACAAAAGCCAAAATTGACAAATTGGATCTAATTAAACTAAAGAGCTTCTGCACAGCAAAAGAAACTACCATCAGAGTGAACAGGCAACCTACAAAATGGGAGAAAATTTTCGCAACCTACTCATCTGACAAAGGGCTAATATCCAGAATCTACAATGAACTCAAACAAATTTACAAGAAAAAAACAAACAACCCCATCAAAAATTGGGCAAAGGACATGAACAGACACTTCTCAAAAGAAGACATTTATGCAGCCAAAAAACACAGGAAAAAATGCTCACCATCACTGGCCATCAGAGAAATGCAAATCAAAACCACAATGAGATACCATCTCACACCAGTTAGAATGGCAATCATTAAAAAGTCAGGAAACAACAGGTGCTGGAGAGGATGTGGAGAAATAGGAACACTTTTACACTGTTGGTGGGACTGTAAACTAATTCAACCCTTGTGGAAGTCAGTGTGGCGATTCCTCAGGGATCTAGAACTAGAAATACCATTTGACCCAGCCATTCCATTACTGGGTATATACCCAAAGGACTATAAATCACGCTGCTATAAAGACACATGCACATGTATGTTTATTGCGGCACTATTCATAATAGCAAAGACTTGGAACCAACCCACATGTCCAACAACGATAGACTGGATTAAGAAAATGTGGCACATATACACCATGGAATACTATGCAGCCATAAAAAATGATGAGTTCATGTCCTTTGTAGGGACATGGATGAAATTGGAAATCACCATTCTCAGTAAACTATCGCCAAGGACAAAAAACCAAACACTGCATATTCTAACTCATAGGTGGGAACTGAACAACGAGAACACATGGACACAGGATGGGGAACATGACATTCGGGACAGTTGTGGGGTGGGGGGAGGGGGGAGGGATAGCTTTAGAAGATATACCTAATGCTAAAAGATGATTTAATAGGTGCAGCGCAGCAGCATGGGACATGTATACATATGTAACTAACCTGCACATTGTGCACATGTACCCTAAAACTT*AAAGTATAATAAAAA****AAAAAA*CTCAACAATAG**AAAAACAAAGTATCCAATGAAAAGGGACCAAAAGTCACAATGAGACCTTT

**Clone 100; PA3; SpIRE(97/622)-100:**

**5’ half of Filled site spans: chr14:65860272-65860472**

**3’ half of Filled site spans: chr14:65854868-65860286**

Empty Site:

TATCAGCAGCGGTGTTTGCAGAACAGCGGTTTTTCGTGAACTGCGAATGCTGCTGTCTGATCATTCCTCTGGAAGTTTTGTCTCAGAGGAGTACCCGGCCGTGTGAAGTGTCAGTCTGCT

CCTACTGGG**GGGTGTCT**TTACCAATACGTCAAGATTTGTT

CAACAGCTTCACCACATGGCCCATGGATTCTTGATTATTACCTTAAATACTGCCCTAAAACCCAGCCATCTTTTAAAGTCGGCAGTAAAGTTTTAGAAAACGCATGTGATTCGCTGTTTT

Filled Site:

GGGGGGTGCCTCCAAGATGGCCGAATAGGAACAGCTCCGGTCTACAGCTCCCAGTGTGAGCGATGCAGAAGACGGGTGATTTCTGCATTTCCATCTGAGCTTTGAAGAGAGCAGTGGTTCTCCCAACATGCAGCTGGAGATCTGAGAATGGGCAGACTGCCTCCTCAAGTGGGTCCCTGACCCCTGACCCCTGAGCAGACTACTTGGAGGCACCCCCCAGTAGGAGCAGACTGACACTTCACACGGCCGGGTACTCCTCTGAGACAAAACTTCCAGAGGAATGATCAGACAGCAGCATTCGCAGTTCACGAAAAACCGCTGTTCTGCAAACACCGCTGCTGATACCCAGGCAAACAGGGTCTGGAGTGGACCTCTAGCAAACTCCAACAGACCTGCAGCTGAGGGTCCTGTCTGTTAGAAGGAAAACTAACAAACAGAAAGGACATCCACACCAAAAACCCATCTGTATATCACCATCATCAAAGACCAAAAGTAGATAAAACCACAAAGATGGGGAAAAAACAGAGCAGAAAAACTGGAAACTCTAAAAAGCAGAGCACCTCTCCTCCTCCAAAGGAACACAGTTCCTCACCAGCAATGGAACAAAGCTGGATGGAGAATGACTTTGACGAGTTGAGAGAAGAAGGCTTCAGATGATCAAACTACGAGCTACAGGAGGAAATTCAAACCAAAGGCAAAGAAGTTAAAAACTTTGAAAAAAATTTAGACGAATGTATAACTAGAATAACCAATACAGAGAAGTGCTTAAAGGAGCTGATGGAGCTGAAAGCCAAGACTCGAGAACTACGTGAAGAATGCAGAAGCCTCAGGAGCCAATGCAATCCACTGGAAGAAAGGGTATCTGTGATGGAAGATGAAATGAATGAAATGAAGCGAGAAGGGAAGTTTAGAGAAAAAAGAATAAAAAGAAATGAACAAAGCTTCCAAGAAATATGGGACTATGTGAAAAGACCAAATCTGCATCTGATTGGTGTACCTGAAAGTGACGGGGAGAGTGGAACCAAGTTGGAAAACACTCTGCAGGATATTATCCAGGAGAACTTCCCCAATCTAGCAAGGCAGGCCAACATTCAGATTCAGGAAATACAGAGAACACCACAAAGATACTCCTCGAGAAGAGCAACTCCAAGACACATAATTGTCAGACTCACCAAAGTTGAAAGGAAGGAAAAAATGTTAAGGGCAGCCAGAGAGAAAGGTCGGGTTACCCACAAAGGGAAGCCCATCGGACTAACAGCGGATCTCTCGGCAGAAACTCTACAAGCTAGAAGAGAGTGGGGGCCAATATTCAACATTCTTAAAGAAAAGAATTTTCAACCCAGAATTTCATATCCAGCCAAAATAAGCTTCATAAGTGAAGGAGAAATAAAATCCTTTACAGACAAGCAAATGCTGAGAGATTTTGTCACCACCAGGCCTGCCCTAAAAGAGCTCCTGAAGGAAGCACTAAACATGGAAAGGAACAACCGATACCAGCTGCTGCAAAATCATGACAAAATGTAAAGACCATTGAGACTAGGAAGAAACTGCATCAACTAACGAGCAAAATAACAAGCTAACATCATAATGACAGGATCAAATTCACACATAACAATATTAACTTTAAATGTAAATGGGCTAAATGCTCCAATTAAAAGACACAGACTGGCAAATTGGATGAAGAGTCAAGAACCATCAGTGTGCTGTATTCAGGAAACCCATCTCATGTGCAGAGACACACATAGGCTCAAAATAAAAGGATGGAGGAAGATCTACCAAGCAAATGGAAAACAAAAAAAGGCAGGGGTTGCAATCCTAGTCTCTGATGAAACAGACTTTAAACCAACAAAGTTCAAAAGAGACAAAGAAGGCCATTACATAATGGTAAAGGGATCAATACAACAAGAAGAGCTAACTATCCTAAATATATATGCACCCAATACAGTAGCACCCAGATTCATAAAGCAAGTCCTGAGTGACCTACAAAGAGACTTAGACTCCCACACATTAATAATGGGAGACTTTAACACCCCACTGTCAACATTAGACAGATCAACGAGACAGAAAGTTAACAAGGATACCCAGGAATTGAACTCAGCTCTGCACCAAGCGGACCTAATAGACATCTACAGAACTCTCCACCCCAAATCAACAGAATATACATTTTTTTCAGCACCACACCACACCTATTCCAAAATTGACCACATACTTGGAAGTAAAGCTCTCCTCAGCAAATGTAAAAGAACAGAAATTATAACAAACTATCTCTCAGACCACAGTGCAATCAAACTGGAACTCAGGATTAAGAAACTCACTCAAAACCACTCAACTACATGGAAACTGAACAAACTGCTCCTGAATGACTACTGGGTACATAACAAAATGAAGGCAGAAATAAAGATGTTCTTTGAAACCAACGAGAACAAAGACACAACATACTAGAATCTCTGGGACACATTCAAAGCAGTGTGTAGAGGGAAATTTATAGCACTAAATGCCCACAAGAGAAAGCAGGAAAGATCTAAAATTGACACCCTAACATCACAATTAAAAGCACTAGAAAAGCAAGAGCAAACACATTCAAAAGCTAGCAGAAGGCAAGAAATAACTAAAATCAGAGCAGAACTGAAGGAAATAGGGACACAAAAAACCCTTCAAAAAATTAATGAATCCAGGAGCTGGTTTTTTGAAACAATCAACACAATTGATAGACCACTAGCAAGACTAATCAAGAAAAAGAGAAGAATCAAATAGACGCAATAAAAAATGATAAAGGGGATACCACCACCGATCCCACAGAAATACAAACTACCATCAGAGATTACTACAAACACCTCTATACAAATAAACTAGAAAATCTAGAAGAAATGGATAAATTCCTGGACACATACACTCTCCCAAGACTAAACCAGGAAGAAGTTGAATCTCTGAATAGACCGATAACAGGAGCTGAAATTGTGGCAATAATCAATAGCTTACCAACCAAAAAGAGTCCAGGACCAGATGGATTCACAGTCGAATTCTACCAGAGGTACAAGGAGGAACTGGTACCATTCCTTCTGAAACTATTCCAATCAATAGAAAAAGAGGGAATCCTCCCTAACTCATTTTATGAGGCCAGCATCATCCTGATACCAAAGCCTGGCAGAGACACAACCAAAAAAGAGAATTTTAGACCAATGTCCTTGATGAACATTGATGCAAAAATCCTCAATAAAATACTGGAAAACTGAATCCAGCAGCACATCAAAAAGCTTATCCACCATGATCAAGTGGGCTTCATCCCTGGGATGCAAGGCTGGTTCAATATACGCAAATCAATAAATGTAATCCAGCATATAAACAGAACCAAAGACAAAAACCACATGATTATCTCAATAGATGCAGAAAAGGCCTTTGACAAAATTCAACAACCCTTCATGCTAAAAACTCTCAATAAATTAGGTATTGATGGGACGTATCTCAAAATAATAAGAACTATCTATGACAAACCCACAGCCAATATCATACTGAATGGGCAAATACTGGAAGCATTCCCTTTGAAAACTGGCACAAGACAGGGATGCCCTCTCTAACCACACCTATTCAACATAGTGTTGGAAGTTCTGGCCAGGGCAATTAGGCAGGAGAAGGAAATAAAGGGTATTCAATTAGGAAAAGAGGAGGTCAAATTGTCCCTGTTTGCAGACGACATGATTGTATATCTAGAAAACCCCAATGTCTCAGCCCAAAATCTCCTTAAGCTGATAAGCAACTTCAGCAAAGTCTCAGGATACAAAATCAATGTACAAAAATCACAAGCATTCTTATACACCAATAACAGACAAACAGAGAGCCAAATCATGAGTGAACTCCCATTCACAAATGCTTCAAAGAGAATAAAATACCTAGGAATCCACCTTACAAGGGACGTGAAGGACCTCTTCAAGGAGAACTACAAACCACTGCTCAATGAAACAAAAGAGGATACAAACAAATGGAAGAACATTCCATGCTCATGGGTAGGAAGAATCAATATCGTGAAAATGGCCATACTGCCCAAGGTAATTTATAGATTCAATGCCATCCCCATCAAGCTACCAATGACTTTCTTCACACAATTGGTAAAAACTACTTTGAAGTTCATATGGAACCAAAAAAGAGCTCACATCGCCAAGTCAATCCTAAGCCAAAAGAACAAAGCTGGAGGCATCACACTACCTGACTTCAAACTATACTACAAGGCTACAGTAACCAAAACAGCATAGTACTGGTGCCAAAACAGAGATATAGATCAATGGAACAGAACAGAGCCCTGAGAAATAATGCCGCATATCTACAACTATCTGATCTTTGACAAACCTGAGAAAAACAAGCAATGGGAAAAGGATTCCCTATTTAATAAATGGTGCTGGGAAAACTGGCTAGCCATATGTAAAAAGCTGAAACTGGATCCCTTCCTTACACCTTATACAAAAATCAATTCAAGATAGATTAAAGACTTAAACGTTAGACCTAAAACCATAAAAACCCTAGAAGAAAACCTAGGCATTACCATTCAGGACATAGGCATGGGCAAGGACTTCATGTCTCAAACACCAAAAGCAATGGCAACAAAAGACAAAATTGACAAATGGGATCTAATTAAACTAAAGAGCTTCTGCACAGCAAAAGAAACTACCATCAGAGTGAACAGGCAACCCACAAAATGGGAGGACATTTTCGCAACCTACTCATCTGACAAAGGGCTAATATCCAGAATCTACAATGAACTCAAACAAATTTACAAGAAAAAAACAAACAACCCCATCAAAAAGTGGGTGAAAGACATGAACAGACACTTCTCAAAAGAAGACATTTATGCAGCCAAAAAACACATGAAAAAATGCTCACCATCACTGGCCATCAGAGAAATGCAAATCAAAACCACAATGAGATATCATCTCACACCAGTTAGAATGGCAATCATTAAAAAGTCAGGAAACAACAGGTGCTGGAGAGGATGTGGAGAAATAGGAACACTTTTACACTGTTGGTGGGACTGTAAACTAGTTCAACCATTGTGGAAGTCAGTGTGGTGATTCCTCAGGGATCTAGAACTAGAAATACCATGTGACCCAGCCATCCCATTACTGGGTATATACCCAAAGGACTATAAATCATGCTGCTATAAAGACACATGCACACGTATGTTTATTGTGGCACTATTCACAATAGCAAAGACTTGGAACCAACCCAAATGTCCAACAACGATAGACTGGATTAAGAAAATGTGGCACATATACACCATGGAATACTAGGCAGCCATAAAAAATGATGAGTTCATGTCCTTTGTAGGGACATGGATGAAATTGGAAATCATCCTTCTCAGTAAACTATCGCAAGAACAAAAAACCAAACACCGCATATTCTCACTCATAGGTGGGAATTGAACAATGAGAACATATGGACACAGGAAGGGGAACATCACACTCCAGGGACTGTTGTGGGGTGGGGGGAGGGATAGCATTGGGAGATATACCTAATGCTAGATGACGAGTTAGTGGGTGCAGCGCACCAGCATGGCACAGGTATACATATGTAACTAACCTGCATATTGTGCACATGTACCCTAAAACTTAAAGTATAATAAT*AAAAAGAAA*

**Clone 106; PA3; SpIRE(97/622)-106: Filled site spans: chr15:71792963-71798702**

Empty Site:

CGTTCAGCCCTTCCTGAGTGGGTGACTGGGGATGGAGCACAAGAAAGCCTGTTTTGAATTATCCCATGCTCCACACCATTCGTTATTCCAGTGTGTGCACACTCGAGTAACACTCAGACG

ACCTTT**AAAAGAAAATAATT**ATTTCACTGCCCTTCAAT

GT

TGAATTAAATAATGTTTAATTATAATATTATGGACATAAAACTAAGTAGAAGGGGTATGACTGATATAACTATCCCATCAAAATGAAATTGCTAATTAAATCAAAGCACAACTTCAAA

Filled Site:

CGTTATTCCAGTGTGTGCACACTCGAGTAACACTCAGACGACCTTT**AAAAGAAAATAATT**GGAAGATGGCCGAATAGTAACAGCTCCGGTCTACAGCTCCCAGTGTGAGCGACGCAGAAGACGGGTGATTTCTGCATTTCCATCGGAGCTTTGAAGAGAGCAGCGGTTCTCCCAGCACGCAGCTGGAGATCTGAGAACGGGCAGACTGCCTCCTCAAGTGGGTCCCTGACCCCTGACCCCCTGAGCAGCCTAACTGGGAAGCACCCCCCCAGCAGGGGCAGACTGACACCTCACACGGCCGGGTACTCCTCTGAGACAAAACTTCCAGAAGAACGATCAGAGAGCAGCATTCGCGGTTCACAAAAAACCACTGTTCTGCAGACACCGCTGCTGATACCCAGGCAAACAGGGTCTGGAGTGGACCTCTAGCAAACTCCAACAGACCTGCAGCTGAGGGTCCTGTCGTTAGAAGGAAAACTAACAAACAGAAAGGACATCCACACCAAAAACGCATCTGGAGATCACCATCATCAAAGACCAAAAGTAGATAAAACCAAAAAGATGGGGAAAAAACAGAGCAGAAAAACTGGAAACTCTAAAAAGCAGAGCACCTCTCCTCCTCCAAAGGATCGCAGTTCCTCACCAGCAATGGAACAAAGCTGGACGGAGAATGACTTTGACGAGTTGAGAGAAGAAGGCTTCAGACGATCAAACTACGAGCTACAGGAGGAAATTCAAACCAAAGGCAAAGAAGTTAAAAACTGAAAAAAATTTAGACGAATGTATAACTAGAATAACCAATACAGAGAAGTGCTTAAAGGAGCTGATGGAGCTGAAAGCCAAGGCTCGAGAACTACGTGAAGAATGCAGAAGCCTCAGGAGCCGATGTGATCAACTGGAAGAAAGGGTATCAGCGATGGAAGATGAAATGAATGAAATGAAGCAAGAAGGGAAGTTTAGAGAAAAAATAATAAAAAGAAACGAACAAAGCCTCCAAGAAATATGGGACTATGTGAAAAGACCAAATCTACGTCTGATTGGTGTACCTGAAAGTGATGGGGAGAATGGAACCAAGTTGGAAAACACTCTGCAGGATATTATCCAGGAGAACTTCCCCAATCTAGCAAGGTAGGCCAACATTCAGATTCAGGAAATACAGAGAACGCCACAAAGATACTCCTCGAGAAGAGCAACTCCAAGACACATAATTGTCAGATTCACCAAAGTTGAAATGAAGGAAAAAATGTTAAGGGCAGCCAGAGAGAAAGGTCGGGTTACCCACAAAGGGAAGCCCATCAGACTAACAGCGGATCTCTCGGCAGAAACTCTACAAGCCAGAAGAGAGTGGGGGCCAATATTCAATATTCTTAAAGAAAAGAATTTTCAACCCAGAATTTCATATCCAGCCAAACTAAGCTTCATAAGTGAAGGAGAAATAAAATCCTTTACAGACAAGCAAATGTTGAGAGATTTTGTCACCACCAGGCCTGCCCTAAAAGAGCTCCTGAAGGAAGCACTAAACATGGAAAGGAACAACCAGTACCAGCCACTGCAAAATCATGCCAAAATGTAAAGACCATCGATGCTATGAAGAAACTGCATCAATTAATGGGCAAAATAACCAGCTAACATCATCATGACAGGTTCAAATTCACACATAACAATATTAACTTTAAATGTAAATGGACTAAATGCTCCAATTACAAGACACAGACTGGCAAATTGGATAAAGAGTCAAGACCCATCAGTGTGCTGTATTCAGGAAACCCATCTCACGTGCAGAGACACACATAGGCTCAAAATAAAAGGATGGAGGAAGATCTACCAAGCCAATGGAAAACAAAAAAAGGCAGGGGTTGCAATCCTAGTCTCTGATAAAACAGACTTTAAACCAACAAAGATCAAAAGAGACAAAGAAGGCCATTACATAATGGTAAAGGGATCAATTCAACAAGAAGAGCTAACTATCCTAAATATATATGCACCCAATACAGGAGCACCCAGATTCATAAAGCAAGTCCTGAGTGACTACAAAGAGACTTAGACTCCCACACATTAATAATGGGAGACTTTAACACCCCACTGTCAACATTAGACAGATCAATGAGACAGAAAGTCAACAAGGATACCCAGGAATTGAACTCGGCTCTGCACCAAGTGGACCTAATAGACATCTACAGAACTCTCCACCCCAAATCAACAGTATACATTTTTTTCAGCACCACACCACACCTATTCCAAAATTGACCACGTAGTTGGAAGTAAAGCTCTCCTCAGCAAATGTAAAAGATCAGAAATTATAACAAACTATCTCTCAGACCACAGTGCAATCAAACTAGAACTCAGGATTAAGAAACTCACTCAAAACTGCTCAACTACATGGAAACTGAACAATCTGCTCCTGAATGACTACTGGGTACATAACGAAATGAAGGCAGAAATAAAGATGTTCTTTGAAACCAACGAGAACAAAGACACAACATACCAGAATCTCTGGGACACATTCAAAGCAGTGTGTAGAGGGAAATTTATAGCACTAAATGCCCACAAGAGAAAGCAGGAAAGATCCAAAATTGACACCCTAACATCACAATTAAAAGAACTAGAAAAGCAAGAGAAAACACATTCAAAAGCTAGCAGAAGGCAAGAAATAACTAAAATCAGAGCAGAACTGAAGGAAATAGAGACACAAAAAACCCTTCAAAAAATTAATGAATCCAGGAGCTGGTTTTTTGAAAGGATCAACAAAATTGATAGACCGCTAGCAAGACTAATAAAGAAAAAAAGAGAGAAGAATCAAACAGACACAATAAAAAATGATAAAGGGGATATCACCACCGATCCCACAGAAATAAAAACTACCATCAGAGAATACTACAAACACCTCTACGCAAATAAACTAGAAAATCTAGAAGAAATGGATAAATTCCTTGACACATACACTCTCCCAAGACTAAACCAGGAAGAAGTTGAATCTCTGAATAGACCAATAACAGGAGCTGAAATTGTGGCAATAATCAATAGCTTACCAACCAAAAAGAGTCCAGGACCGGATGGATTCACAGCCGAATTCTACCAGAGGTACAAGGAGGAACTGGTACCATTCCTTCTGAAACTATTCCAATCAATAGAAAAAGAGGGAATCCTCCCTAACTCATTTTATGAGGCCAGCATCATCCTGATACCAAAGCCTGGCAGAGACACAAACAAAAAAGAGAATTTTAGACCAATATCCTTGATGAACATTGATGCAAAAATCCTCAATAAAATACTGGCAAACCGAATCCAGCAGCACATCAAAAAGCTTATCCACCATGATCAAGTGGGCTTCATCCCTGGGATGCAACGATGGTTCAATATATGCAAATCAATAAATATAATCCAGCATATAAACAGAACCAAAGACAAAAACCACATGATTATCTCAATAGATGCAGAAAAGGCCTTTGACAAAATTCAAAAACCCTTCATGCTAAAAACTCTCAATAAATTAGGTATTGATGGGACGTATCTCAAAATAATAAGAGCTATCTATGACAAACCCACAGCCAATATCATACTGAATGGGCAAAAACTGGAAGCATTCCCTTTGAAAACTGGCACAAGACAGGGATGCCCTCTCTCACCACTCCTATTCAACATAGTGTTGGAAGTTCTGGCCAGGGCAATTAGGCAGGAGAAGGAAATAAAGGGTATTCAATTAGGAAAAGAGGAAGTCAAATTGTCCCTGTTTGCAGATGACATGATTGTATATCTAGAAAACCCCATTGTCTCAACCCAAAATCTCCTTAAGCTGATAAGCAACTTCAGCAAAGTCTCAGGATACAAAATCAATGTACAAAAATCATAAGCATTCTCATACACCAATAACAGACAAACAGAGAGCCAAATCATGAGTGAACTCCCATTCACAATTGCTTCAAAGAGAATAAAATACCTAGGAATCCAACTTACAAGGGATGTGAAGGACCTCTTCAAGGAGAACTACAAACCACTGCTCAATGAAATTAAAGAGGATACAAACAAATGGAAGAACATTCCATGCTCATGGATAGGAAGAATCAATATCATGAAAATGGCCATACCGCCCAAGGTAATTTATAGATTCAATGCCATCCCCATCAAGCTACCATCGACTTTCTTCACAGAATTGGAAAAAACTACTTTAAAGTTCATATGGAACCAAAAAAGAGCCCACATCGCCAAGTCAATCCTAAGCCAAAAGAACAAAGCTGGAGGCGTCACGCTACCTGACTTCAAACTATACTACAAGGCTACAGTCACCAAAACAGCATGGTACTGGTACCAAAACAGAGATATAGATCAATGGAACAGAACAGAGCCCTCAGAAATAACGCCGTATATCTACAACTATCTGATCTTTGACAAACCTGAGAAAAACAAGCAATGGGGAAAGGATTCCCTATTTAATAAATGGTGCTGGGAAAACTGGCTAGGCATGTGTAGAAAGCTGAAACTGGATCCCTTCCTTACACCTTATACAAAAATCAATTCAAGATGGATTAAAGACTTAAACGTTAGACCTAAAACCATAAAAACCCTAGAAGAAAACCTAGGCATTACCATTCAGGACATAGGCACGAGCAAGGACTTCATGTCTAAAACACCAAAAGCAATGGCAAAAAAAAGCCAAAATTGACAAATGGGATCTAATTAAACTAAAGAGCTTCTGCACAGCAAAAGAAACTACCATCAGAGTGAACAGGCAACCCACAAAATGGGAGAAAATTTTCGCAACCTACTCATCTGACAAAGGGCTAATATCCAGAATCTACAATGAACTCAAACACATTTACAAGAAAAAAACAAACAACCCCATCAAAAAGTGGGCGAAGGACATGAACAGACACTTCTCAAAAGAAGACATTTATGCAGCCAAAAAACACATGAAAAAATGCTCACCATCACTGGCCATCAGAGAAATGCAAATCAAAGCCACAATGAGATACCATCTCACACCAGTTAGAATGGCAATCATTAAAAAGTCAGGAAACAACAGGTGCTGGAGAGGATGTGGAGAAATAGGAACACTTTTACACTGTTGGTGGGACTATAAACTGGTTCAACCATTGTGGAAGTCAGTGTGGCGATTCCTCAGGGATCTAGAACTAGAAATACCATTTGACCCAGCCATCCCATTACTGGGTATATACCCAAAGGACTATAAATCATGCTGCTATAAAGATACATGCACACGTGTGTTTATTGTGGCACTATTCACAATAGCAAAGACTTGGAACCAACCCAAATGTCCAACAATGATAGACTGGATTAAGAAAATGTGGCACATATACACCATGGAATACTATGCAGCCATAAAAAATGATGAGTTCATGTCCTTTGTAGGGACATGGATGAAATTGGAAATCATCATTCTCAGTAAACTGTCGCAAGAACAAAAAACCAAACACCGCATATTCTCACTCATAGGTGGGAACTGAACAATGGGAACACATGGACACAGGAGGGGGAACATCACAGTCTGGGGACTGTTGTGGGGTCGGGGGAGAGGAGAGGGATAGCATTGGGAGATATACCTAATGCTAGATGACGAGTTAGTGGGTGCAGTGCACCAGCATGTCACATGTATACATATGTAACTAACCTGCACATTGTGCACATGTACCCTAAAACTTAAAGTATAAT*AATTAAAAAGAAAAAAAGAAAA****AA*CAGAAAATAATT**ATTTCACTGCCCTTCAATGTTGAATTAAATAATGTTT

**Clone 107; PA3; SpIRE(97/622)-107: Filled site spans: chr15:77260604-77266304**

Empty Site:

ATGAAAAAAGCAATATAATGGTGACAGAAATAGAGGATTTTCAAAAAGAACCAAAAAATAACTTCTAGAGATAAAATATATAGAGACAAATGAATATTTCACTAGACAGATAAGCAGTAG

TTCATACACAGAAG**AAAAAAA**TAAAAATAAAATACAATAA

AATTTAAAAACTAATCAAAGAACCAAATAATTTTACTCCTAGGTATTTACTCCACAGAGTGGGTATCATCAGAGGCCTAGTACGAAGCTGAAACTCCCAACTCGGCCCAGAACAGGCGCC

Filled Site:

TAGAGACAAATGAATATTTCACTAGACAGATAAGCAGTAGTtCATACACAGAAG**AAAAAAA**GGTGGAGCCAAGATGGCCGAATAGGAACAGCTCTAGTCTACAGCTCCCAGCGTGAGCGACACAGAAGATGTATGATTTCTGCATTTCCAACTGAGCTTTGAAGAGAGTAGTGGTTCTCCCAGCACGCAGCTTGAGATCTGAGAACGGACAGACTGCCTCGTCAAGTGGCTCCCTGACGCCCAAGTAGCCTAACTGGGAGGCACCCCCCAGTAGAGGCAGACTGACACCTCACATGGCCGGGTACTCCTCTGAGACAAACCTTCCAGAGGAATGATCAGGCAGCAACATTTGCTGTTCACCAATATCTGCTGTTGTGCAGCCTCCGCTGCTGATACCCAGGCAAACAGCATCTGGAGAGGACCCCCAGCAAACTCCAACAGACCTGCAGCTGAGGGTCCTGACTGTTAGAAGGAAAACTAATAAACAGAAAGGACATCCACACCAAAACCCCATCTGTATGTCACCATCATCAAAGACCAAAGGTAGATAAAACCACAAAGATGGGGAAAAAACAGAGCAGAAACACTGAAAATTCTAAAAATCAGAGCGCCTCTCCTCCTCCAAAGGAATGCAGCTCCTCACCAGCAATGGAACAAAGCCGGATGGAGAATGACTTTGATGAGTTGAGAGAAGAAGGCTTCAGACGATCAAACTACTCCAAGCTAAAGGAGGAAGTTCGAACCCATGGCAAAGAAGTTAAAAACCTTGAAACAAGATTAGACGAATGGCTAACTAGAATAACCAATGCAGAGAAGTCCTTAAAGGAGCTGATGGAGCTGAAAACCACGGCACGAGAACTATGTGATGAATGCACGAGCCTCAGCAGCCGATTCGATCAACTGGAAGAAAGGGTATCAGTGATGGAAGATCAAATGAATGAAATGCGAGTTTAGAAAAAAAGAATAAAAAAAAAGAACAAAGCCTCCAACAAATATAGGACTATGTGAACAGACCAAATCTATATCTGATTGGTGTACCTGAAAGTGACGGGGAGAATGGAACCAACTTGGAAAACACTCTGCAGGATATTATCCAGGAGAACTTCCCCAATCTAGCAAGGCAGGCCAACATTCAGATTCGGGAAATACAGAGAACGTCATAAAGATACTCCTCCAGAAGAGCAACTCTAAGACACATAATTGTCAGATTCACCAAAGTTGAAATGAAGGAAAAAATGTTAAGGGCAGCCAGAGAGAAAGGTCGGGTTACCCACAAAGGGAAGCCCATCAGACTAACAGCTGATCTCTTGGCAGAAACTCTACAAGCCAGAAGAGAGTGGGGGCCAATATTCAACATTCTTAAAGAAAAGAATTTTCAACCCACAATTTCATATCCAGCCAAACTAAGCTTCATAAGTGAAGGAGAAATAAAATACTTTACAGACAAGCAAATGCTGAGAGATTTTGTCACTACCAGGCCTGCCCTAAAAGAACTCCTGAAGGAAGCACTAAACATGGAAAGGAACAACCAGTTCCAGCCACTGCAAAAACATGTCAAACTGTAAAGACCATCGATGCTAGGAAGAAATTGCATCAACTAACGAGCAAAATAACCAGCTAACATCATAATGACAGGATCAAATTCACACATAACAATATTAACTTTAAATGTAAATGGGCTAAATGCTCCAATTAGAAGACACAGACTGGCAAATTGGATAAAGAGTCAAGACCCATCAGTGTGCTGTATTCAGGAAACCCATCTCACATGCAGAGACACACGTAGGCTCAAAATAAAGGGATGGAGGAAGATCTACTAAGCAAATGGAAAACAAAAAAGGCAGGGGTTGCAATCCTAGTCTCTGATAAAACAGACTTTAAACCAATAAAGATCAAAAGAGACAAAGAGGGCCATTACATAATGGTAAAGGGATCAATTGAACAAGAAGAGCTAACTATCCTAAATATATATGCACCCAATACAGGAGCACCCAGATTCATAAAGCAAGTCCTTAGAGACCTACAAAGAGACTCCCACACAATAATAATGGGACACTTTAACACCCCACAGACAACATTAGACAGACCAACGAGACAGAAAGTTAACAAGGATACGCAGGAATTGAACTCAGCTCTGCACCAAGCAGACCTAATAGACATCTACAGAACTCTCCACCCCAAATCAACAGAATATACATTTTTTTCAGCACCACACCACACCTATTCCAAAATTGACCACATAGTTGGAAGTAAAGCAGTCCTCAGCAAATGTAAAAGAACAGAAATTATAACAAACTGTCTCTCAGGCCACAGTACAATCAAACTAGAACTCAGGATTAAGAAACTCACTCAAAACCACTCAGCTACATGGAAACTGAACAACCTGCTCCTGAATGACTACTGGGTACATAATGAAATGAAGGCAGAAATAAAGATGTTCTTTGAAACCAATGAGAACAAAGACACAACATACCAGAATCTCTGGGACACATTCAAAGCAGTGCGTAGAGGGAAATTTATAGCACTAAATGCCCACAAGAGAAACCAAGAAAGATCTAAAATTGACACCATAACATCACAATTAAAAGAACTAGAGAAGCAAGAGGAAACACATTCAAAAGCTAGTAGAAGGCAAGAAATAACTAAGATCAGAGCAGAACTGAAGGAAATAGACACATAAAAAACACTTCAAAAAAATCAATGAATCCAGGAGCTGGTTTTTTGAAAAGATCAACAAAATTGATAGACCGCTAGCTAGACTAATAAAGAAGAAAAGAGAGAAGAATCAAATAGACGTAATAAAAAATGATAAACGGGATATCACCACCGATCCCACAGAAATACAAACTACCATCAGAGAATACTATAAACACCTCTACACAAATAAACTAGAAAATCTAGAATAAATGGATAAATTCCTCGACACCTACACCCTCCCAAGACTAAACCAGGAAGAAGCTGAATCTCTGAATAGACCAATAACAGGCTCTGAAATTGAGGCAATAATTAATAGCTTACTAACCAAAAAAAGGCCAGGACCAGATGGATTCACAGCCGAATTCTACTAAAGGTACAAGGAGGAGCTGGTACCATTCCTTCTGAAACTATTCCAATCAATAGAAAAAGAGGGAATCCTCCCTAACTCATTTTATGAGGCCAGCATCATCCTGATACCAAAGCCTGGCAGAGACACAACAACAAAAAAAAGAATTTTAGACCAATATCCCTGATGAACATCGATGCAAAAATCCTCAATAAAATACTAGCAAACCGAATCCAGCAGCACATCCAAAAGCTTATCTACCATGATCAAGTGGGCTTCAACCTGGGATGCAAGGCTGGTTCAACATACGCAAATCAATAAACATAATCCAGCATATAAACAGAACCAACGACAAAAACCACGATTATCTCAATAGATGCAGAAAAGGCCTTTGACAAAATTCAACAACCCTTCATGCTAAAAACTCTCAATAAATTAGGTATTGATGGGACGTATCTCAAAATAATAAGAGCTATCTGTGACAAACCCACAGCCAACATCATACTGAATGGGCAAAAACTGGAAGCATTCCCTTTGAAAACTGGCACAAGACAGGGATGCCCTCTCTCACCACTCCTATTCAACATAGTGTTGGAAGTTCTGGCCAGGGCAATCAGGCAGGAGAAGGAAATAAAGGGTATTCAATTAGGAAAAGAGGAAGTCAAATTGTCCCTGTTTGCAGATGACATGATTGTATATCTAGAAAACCCCATTGTCTCAGCCCAAAATCTCCTAAAGCTGATAAGCAACTTCAGCAAAGTCTCAGGATACAAAATCAATGTATAAAATCACAAGCATTCTTATACACCAATAACAGACAAACAGAGAGCCAAATCATGAGTGAACTCCCATTCACAATTGCTTCAAAGAGAATACAATACCTAGGAATCCAATTTACAAGGGACGTGAAGGACCTCTTCAAGGAGAACTACAAACCACTGCTCAATGAAATAAAAGAGGATACCAACAAATGGAAGAACATTCCACGCTCATGGGTAGGAAGAATCAATATCGTGAAAATGGCCATACTGCCCAAGGTAATTTATAGATTCAATGCCATCCCCATCAAGCTACCAATGACTTTCTTCACAGAATTGGAAAAAACTACTTTAAAGTTCATATGAAACCAAAAAAGAGCCCGCATCGCCAAGTCAATCCTAAGCCAAAAGAACAAAGCTGGAGGCATCACACTACCTGACTTCAAACTATACTACAAGGCTACAGTAACCAAAACAGCATGGTACTGGTACCAAAACAGAGATATAGATCAATGGAACAGAACAGAGCCCTCAGAAATAACACTGCATATCTGCAACTATCTAATCTTTGACAAACCTGAGAAAAACAAGCAATGGGGAAAGGATTCCCTATTTAATAAATGGTGCTGGGAAAACTGTCTAGCCATATGTAGAAAGCTGAAACTGGATCCCTTCCTTACACCTTACACAAAAATCAATTCAAGATGGATTAAAGACTTAAACGTTAGACCTAAAACCATAAAAACCCTAGAAGAAAACCCAGGCATTACCATTCAGGACATAGGCATGGGCAAGGACTTCATGTCTAAAACACCAAAAGCAATGGCAACAAAAGCCAAAATTGACAAATGGGATCTAATTAAACTAAAGAGCTTCTGCACAGCAAAAGAAACTACCAACAAAGTGAATAGACAACCCACAAAATGGGAGAAAATTTTCGCAACCTACTCATCTGACAAAGGGCTAATATCCAGAATCTAAAGTGAACTCAAACAAATTTACAAGAAAAAAACAAACAACCCCATCAAAAAGTGGGCGAAGGACATGAACAGACACTTCTCAAAAGAAGACATTTATGCAGACAAAAGACACATGAAAAAATGCTCACCATCACTGGCCATCAGAGAAATGCAAATCCAAACTGCAATGAGATACCATCTCACACCAGTTAGAATGGCGATCATTAAAAAGTCAGGAAACAACAGGTGCTGGAGAGGATGTGGAGAAATAGGAACACTTTTACACTGTTGGTGGGACTGTAAACTAGTTCAACCATTGTGGAAGTCAGTGTGGCGATTCCTCAGGGCTCTAGAACTAGAAATACCATTTGACCCAGCCATCCCATTACTGGGTATATACCCAAAGGACTATAAATCATGCTGCTATAAAGACACATGCACACGTATGTTTATTGTGGCACTATTCACAATAGCAAAGACTTGGAACCAACCCAAATGTCCAACAATGATAGACTGGATTAAGAAAATGTGGCACGTATACACCATGGAATACTATGCAGCCATAAAAAATGAGGAGTTCATGTCCTTTGTAGGGACATGGATGAAATTGGAAATCATCATTCTCAGTAAACTATCACAAGAAAAACCAAACACCGCATATTCTCACTCATAGGTGGGAACTGAACAATGAGAACACATGGACACAGGAAGGGGAACATCACACTCTGGGGACTGTTGTGGGGTGGGGAGAGGGGGGAGGGATAGCATTTGGAGATATACCTAACGCTAGATGACAAGTTAGTGGGTGCAGCGCACCAGCATGTCACATGTATACATATGTAACTAACCTGCACATTGTGCACATGTACCCTAAAAGTTAAAGTATAAT*AATAA****AAAAAAA***TAAAAATAAAATACAATAAAATTTAAAAACTAATCAAAGA

**Clone 108; PA3; SpIRE(97/622)-108: Filled site spans: chr16:63147076-63153346**

Empty Site:

TGTGGGAGTCAAAAATATGAAGATAAATACCACTCAAATCCTTGCATAGCACCTGAATTATGCCTGTGCTAGAGGGAAAAAGACAATAAAGCAGACAAAAGATTAGAAGAAATAAAGGCA

CTACATTTCTCAAATTTGG**TAAAAAAG**GCTCTTACATTTG

ATATGAAGTAATATATTTTTTAAAAGAATAAAAGATACATATTGCAATCTCCAGAACTACTATAAAAATAACTAAACGAAGCAGAATTAAAGAGCCAATAGAGGAATTAAAAGAAAAAAA

Filled Site:

AGACAATAAAGCAGACAAAAGATTAGAAGAAATAAAGGCACTACATTTCTCAAATTTGG**TAAAAAAG**AACCCCATAAACTTAAAAATCAAAAGAGCACAGGAAACACTAAACAGGATAAATACAAGAAGAATACATTTAGACATATTATAGCCAAAATGATGAAAAGCAAGACAAAGATAAATTTTAGAAATAATATATTTTTAATGAATTAATAAATAGTGTAGGAACAATTGGACATCAATGGACAAAATCTAAACTTTAAAACCTTACACATTATACAAAAGTTAATCCAAAATAAATCATGAATTTATTTTCTATTTTATTTTTATTTATTTATATTTCGGCTACAGGGTCTCACTTTGTCACCCAGGCTGGAGTGCAGTGGCACAATCTCAGCTCACAGCAGCCTCTACCTGCTTGGCTCAAGCAATCCTCCTACCTCAGCCCCCAAGTAGCTGGGACCACAGGCATGCGCCACCACACCCAGCTAATTTTTTTGGATCTTTTGTAGAGACAGGGTTTTGCCGTGTTGCCCAGGCCGGTCTCAAACTCCTAGCTGGTCAAGCAATCTCCTGCCTCGGCCTCCCAAAGTCCTAGGATTATAAGCGTGAGCCTCTGCCCCTGGCCTGGATCTACAGCTCCCAGCGTGAGTGACGCAGAAGACGGGTGATTTCTGCATTTCCATCTGAGCTTTGAAGAGAGCAGTGGTTCTCCCAGCACGCAGCTGGAGATCTGAGAAGGGGCAGACTGCCTCCTCAAGTGGGTCCCTGACCCCTGACCCCTGAGCAGCCTAACTGGCAGGTACCCCCCAGTAGGGGCAGACTGACACCTCACACGGCCGGGTACTCCTCTGAGACAAAACTTCCAGAGGAATGATCAGACAGCAGCATTCACGGTTCATGAAAAAACACTGTTCTGCAGACACTGCTGCTGATGCCCAGGAAAACAGGGTCTGGAGTGGACCTCTAGCAAACTCCAACAGACTTGCAGCTGAGGGTCCTGTCTGTTAGAAGGAAAACTAACAAACAGAAAGGACATCCACACCAAAAACCCATCTGTACATCACCATCATCAAAGAGCAAAAGTAGATAAAACCACAAAGATGGGGAAAAAACAGAGCAGAAAAACTGGAAACTCTAAAAAGCAGAGCACTTCTCCTCCTCCAAAGGATCGCAGTTCCTCACCAGCAATGGAACAAAGCTGGATGGAGAATGACTTTGACAAGTTGAGAGAAGGCTTCAGACGATCAAACTACAAGCTACAGGAGGAAATTCAAACCAAAGGCAAAGAAGTTAAAAACTTTGAAAAAAATTTAGACGAATGTATAACTAGAATAACCAATACAGAGAAGTGCTTAAAGGAGCTGATGGAGCTGAAAGCCAAGGCTCGAGAACTATGTGAAGAATGCAGAAGCCTCAGGAGCCGATACGATCAACTGGAAGAAAGGGTATCAGCGATGGAAGATGAAATGAATGAAATGAAGTGAGAAGGGAAGTTTAGAGAAAAAAGAATAAAAAGAAATGAACAAAGCCTCCAAGAAATATGGGACTATGTGAAAAGACCAAATCTATGTCTGATTGGTGTACCTGAAAGTGATGGGGAGAATGGAACCAAGTTGGAAAACAATCTGCAGGATATTATCCAGGAGAACTTCCCCAGTCTAGCAAGGCAGGCCAACATTCAGATTCAGGAAATACAGAGAACGCCACAAAGATACTCCTCGAGAAGAGCAACTCCAAGACACATAATTGTCAGATTCACCAAAGTTGAAATGAAGGAAAAAATGTTAAGGGCAGCCAGAGAGAAAGGTCAGGTTACCCACAAAGGGAAGCCCATCAGACTAACAGAGCATCTCTCGGCAGAAACTCTACAAGCCAGAAGAGAGTGGGGGCCAATATTCAACATTCTTAAAGAAAAGAATTTTCAACCCAGAATTTCATATCCAGCCAAACTAAGCTTCATAAGTGAAGGAGAAATAAAATCCTTTACAGACAAGCAAATGCTGAGAGATTTTGTCACCACCAGGCCTGCCCTAAAAGAGCTCCTGAAGGAAGCACTAAACATGGAAAGGAACAACCAGTACCAGCTGCTGCAAAATCATGCCAAAATGTAAAGACCATTGAGACTAGGAAGAAACTGCATCAGCTAACAAGCAAAATAACCAGCTAACATCATAATGACAGGTTCAAATTCACACATAACAATATTAACTTTCAATGTAAATGGACTGAATGCTCCAATTAAAAGACACAGACTGGCAAATTGGATAAAGAGTCAAGACCCATCAGTGTGCTGTATTCAGGAAACCCATCTCACGTGCAGAGACACACATAGGCTCAAAATAAAAGGATGGAGGAAGATCTACCGAGCAAATGGAAAACAAAAAAAGGCAGGGGTTGCAATCCTAGTCTCTGATAAAACAGACTTTAAACCAACAAAGATCAAAAGAGACAAAGAAGGCCATTACATAATGGTAAAGGGATCAATTCAACAAGAAGAGCTAACTATCCTAAATATATATGCACCCAATACAGGAGCACCCAGATTCATAAAGCAAGTCCTGAGTGACCTACAAAGAGACTTAGACTCCCACACATTAATAATGGGAGGCTTTAACACCCCACTGTCAACATTAGACAGATCAACGAGACAGAAAGTCAACAAGGATACCCAGGAATTGAACTCAGCTCTGCACCAAGCGGACCTAATAGACATCTACAGAACTCTCCACCCCAAATCAACAGAATATACATTTTTTTCAGCACCACACCACACCTATTCCAAAATTGACCACATACTTGGAAGTAAAGCTCTCCTCAGCAAATGTAAAAGAACAGAAATTATAACAAACTATCTCTCAGACCACAGTGCAATCAAACTAGAACTCAGGGTTAAGAAACTCACTCAAAACCGCTCAACTACATGGAAACTGAACAACCTGCTCCTGAATGACTACTGGGTACATAACAAAATGAAGGCAGAAATAAAGATGTTCTTTGAAACCAATGAGAACAAAGATACAACATACCAGAATCTCTGGGACACATTCAAAGCAGTGTGTAGAGGGAAATTTATAGCACTAAATGCCCACAAGAGAAAGCAGGAAAGATCCAAAATTGACAGCCTAACATCACAATTAAAAGAACTAGAAAAGCAAGAGCCAACACATTCAAAAGCTAGCAGAAGGCAAGAAATAACTAAAATCAGAGCAGAACTGAAGGAAATAGAGACACAAAAAACCCTTCAAAAAATTAATGAATCCAGGAGCCGGTTTTTTGAAAGGATCAACAAAATTGATAGACTGCTAGCAAGACTAATAAAGACAAAAAGAAGAATCAAATGGACGCAATAAAAAATGATAAAGGGGATATCACCACCGATCCCACAGAAATACAAACTACCATCAGAGAATACTACAAACACCTCTATGCAAATAAACTAGAAAATCTAGAAGAAATGGATGAATTCCTGGACACATACACTCTCCCAAGACTAAAGCAGGAAGAAGTTGAATCTCTGAATAGACCAATAACAGGATCTGAAATTGTGGCAATAATCAATAGCTTACCAACCAAAAAGAGTCCAGGACCAGATGGATTCACAGCCGAATTCTACCAGAGGTACAAGGAGGAACTGGTGCCATTCCTTCTGAAACTATTCCAATCAATAGAAAAAGAGGGAATCCTCCCTAGCTCATTTTATGAGGCCAGCATCATCCTGATACCAAAGCCGGGCAGAGACACAACCAAAAAAGAGAATTTTAGACCAATATCCTTGATGACATTGATGCAAAAATCCTCAATAAAATACTGGCAAACTGAATCCAGCAGCACATCAAAAAGCTTATCCACCATGATCAAGTGGGCTTCATCCCTGGGATGCAAGGCTGGTTCAATATACACAAATCAATAAATGTAATCCAGCATATAAACAGAACAAAAGACAAAAACCACATGATTATCTCAATAGATGCAGAGAAGGCCTTTGACAAAATTCGACAACCTTCATGCTAAAAACTCTCAATAAATTAGGTATTGATGGGACGTATCTCAAAATAATTAGAGCTATCTATGACAAACCCACAGCCAATATCATACTGAATGGGCAAAAACTGGAAGCATTCCCTTTGAAAACTGGGCACAAGACAGGGATGCCCTCTCTCACCACTCCTATTCAACATAGTGTTGGAAGTTCTGGCCAGGGCAATTAGGCAGGAGAAGGAAATAAAGGGTATTCAATTAGGAAAAGAGGAAGTCAAATTGTCCCTGTTGTAGACGACATGATTGTTTATCTAGAAAACCCCATTGTCTCAGCCCAAAATCTCCTTAAGCTGATAAGCAACTTCAGCAAAGTCTCAGGATACAAAATCAATGTACAAAAATCACAAGCATTCTTATACACCAATAACAGACAAACAGAGAGCCAAATCATGAGTGAACTCCCATTCACAATTGCTTCAAAGAGAATAAAATACCTAGGAATCCACCTTACAAGGGATGTGAAGGACCTCTTCAAGGAGAACTACAAACCACTGCTCAATGAAATTAAAGAGGATACTAACAAATGGAAGAACATTCCATGCTCATGGATAGGAAGAATCAATATCGTGAAAATGGCCATACTGCCCAAGGTAATTTATAGATTCAATGCCATCCCCATCAAGCTACCAATGACTTTCTTCACAGAATTGGAAAAAACTACTTTAAAGTTCATATGGAACCAAAAAAGAGCCCGCATCGCCAAGTCAATCCTAAGCCAAAAGAACAAAGCTGGAGGCATCACGCTACCTGACTTCAAACTATACTACAAGGCTACAGTAACCAAAACAGCACGGTACTGGTACCAAAACAGAGATATAGATCAATGGAACAGAACAGAGCCCTCAGAAATAACGCCGCATATCTACAACTATCTGATCTTTGACAAACCTGAGAAAAACAAGCAATGGGGAAAGGATTCCCTATTTAATAAATGGTGCTGGGAAAACTGGCTAGCCATATGTAGAAAGCTGAAACTGGATCCCTTCCTTACACCTTATACAAAAATCAATTCAAGATGGATTAAAGACTTAAACGTTAGACCTAAAACCATAAAAACCCTAGAAGAAAACCTAGGCATTACCATTCAGGACATAGGCATGGACAAGGACTTCATGTTTAAAACACCAAAAGCAATGGCAACAAAAGCCAAAATTGACAAATGGGATCTAATTAAACTCAAGAAGCTTCTGCACAGCAAAAGCAACTACCATCAGAGTGAACAGGCAACCCACAAAATGGGAGAAAATTTTCACCACCTACTCATCTGACAAAGGGCTAATATCCAGAATCTACAATGAACTCAAACAAATTTATAAGAAAAAAACAAACAACCCCATCAAAAAGTAGGCAAAGGACATGAACAGACACTTCTCAAAAGAAGACATTTATGCAGCCAAAAAACACATGAAAAAATGCTTACCGTCACTGGCCATCAGAGAAATGCAAATCAAAACCACAATGAGATATCATCTCACACCAGTTAGAATGGCAATCATTAAAAAGTCAGGAAACAACAGGTGCTGGAGAGGATGTGGAGAAATAGGAACACTTTTACACTGTAGGTGGGACTGTAAACTAGCTCAACCATTGTGGAAGTCAGTGTGGCGATTCCTCTGGGATCTAGAACTAGAAATACCATTTGACCCAGCCATCCCATTACTGGGTATATACCCAAAGGACTATAAATCATGCTGCTGTAAAGACACATGCACAGGTATGTTTATTGTGGCTCTATTCACAATAGCAAAGACTTGGAACCAACCCAAATGTCCAACAATGATAGGCTGGATTAAGAAAATGTGGCACATATACACCATGGAATACTATGCAGCCATAAAAAATGATGAGTTCATGTCCTTTGTAGGGACATGGATGAAATTGGAAATCATCATTCTCAGTAAACTATCCCAAGAACAAAAAAACAAACACTGCATATTCTCACTCATAGGTGGGAATTGAACAATGGGAACAGATGGACACAGGAAGGGGAACATCACACTCTGGGGACTGTTGGGGGGAGGGGGGAGGGATAGCATTGGGAGATGTACCTAATGCTAGATGACAAGTTAGTGGGTGTAGCGCACCAGCATGTCACATGTGTACATATGTAACTAACCTGCACATTGTGCACATGTACCCTAAAACTT*AAAGTATAATAATAATAA****TAAAAAA*G**GCTCTTACATTTGATATGAAGTAATATATTTTTTAAAAGTAATAAAAGATACATATT

**Clone 111; PA3; SpIRE(97/622)-111: Filled site spans: chr18:49771040-49776625**

Empty Site:

ATATACATTCTATTCATTGGCACATGGAACATTCTCCAAGATAGACCATATGATAGGACACAAAACAAGTCTCAGTAAATTCAAGAAAACTGAAATTATATCAAGTACTGTCTCAGACTA

CAGTAG**AATAAAA**GAAAAAAAAAGTCATATACAAAAAAGA

TACTTGCACACACGTTTATAGCAGCACAATTTACAATTGCAAAAATATGGAACTAGCCCAAATGCCCATCAATCAATGAGTGGATAAAGAAAATGTTGTATAGCTGGGCGTGGTGGCCCA

Filled Site:
[truncated: 416,253 more chars]
